# PacBio single-molecule long-read sequencing provides new insights into the complexity of full-length transcripts in oriental river prawn, *macrobrachium nipponense*

**DOI:** 10.1186/s12864-023-09442-x

**Published:** 2023-06-20

**Authors:** Cheng-Yan Mou, Qiang Li, Zhi-Peng Huang, Hong-Yu Ke, Han Zhao, Zhong-Meng Zhao, Yuan-Liang Duan, Hua-Dong Li, Yu Xiao, Zhou-Ming Qian, Jun Du, Jian Zhou, Lu Zhang

**Affiliations:** 1 Fisheries Institute, Sichuan Academy of Agricultural Sciences, Chengdu, Sichuan 611731 China; 2 Sichuan Academy of Agricultural Sciences, Chengdu, Sichuan 610066 China; 3 Chengdu Eaters Agricultural Group Co., Ltd, Chengdu, Sichuan 610000 China

**Keywords:** Long non-coding RNA, Novel genes, Alternative splicing, Alternative polyadenylation, SMRT sequencing, Oriental river prawn

## Abstract

**Background:**

Oriental river prawn (*Macrobrachium nipponense*) is one of the most dominant species in shrimp farming in China, which is a rich source of protein and contributes to a significant impact on the quality of human life. Thus, more complete and accurate annotation of gene models are important for the breeding research of oriental river prawn.

**Results:**

A full-length transcriptome of oriental river prawn muscle was obtained using the PacBio Sequel platform. Then, 37.99 Gb of subreads were sequenced, including 584,498 circular consensus sequences, among which 512,216 were full length non-chimeric sequences. After Illumina-based correction of long PacBio reads, 6,599 error-corrected isoforms were identified. Transcriptome structural analysis revealed 2,263 and 2,555 alternative splicing (AS) events and alternative polyadenylation (APA) sites, respectively. In total, 620 novel genes (NGs), 197 putative transcription factors (TFs), and 291 novel long non-coding RNAs (lncRNAs) were identified.

**Conclusions:**

In summary, this study offers novel insights into the transcriptome complexity and diversity of this prawn species, and provides valuable information for understanding the genomic structure and improving the draft genome annotation of oriental river prawn.

**Supplementary Information:**

The online version contains supplementary material available at 10.1186/s12864-023-09442-x.

## Introduction

Oriental river prawn (*Macrobrachium nipponense*), which belong to family Palaemonidae, order Decapoda, subphylum Crustacea, are commonly found in the low-salinity and freshwater regions of estuaries in China [1]. It has become one of the most dominant species in shrimp farming in China, with delicious taste and high nutritional value. The annual production capacity of oriental river prawn has increased to 228,765 tons in 2020 [2]. With the rapidly developing sequencing technology, genome and transcriptome information can serve as an effective tool in promoting the breeding process of oriental river prawn. In 2011, 15,806 bp (bp) mitochondrial genome from a single female oriental river prawn were sequenced, which is comprised of 37 genes, including 13 protein-coding genes (PCGs), 22 transfer RNAs (tRNAs) and 2 ribosomal RNAs (rRNAs) [1]. In 2013, transcriptome analysis of oriental river prawn androgenic gland showed that a total of 78,408 isosequences were obtained, among which 57,619 non-redundant transcripts and 40 candidate NGs were found [3]. The latest reference genome assembly (ASM1510439v1) of oriental river prawn was generated by using Illumina and PacBio sequencing, assembling ∼4.5 Gb of the genome, with predictions of 44,086 protein-coding genes. This assembly has a higher sequence continuity and accuracy because of using two sequencing methods [4]. Other transcriptome analysis mainly focused on revealing differential gene expression analysis and dynamic spatial gene coexpression networks [5–8]. Although these sequences can serve as useful genetic resources for shrimp breeding, the genome coverage remains incomplete. To date, most of the gene models were predicted in silico, and the information on untranslated regions and alternative isoforms are still lacking [9–11]. Hence, more precise genomic information is essential to improve the functional and structural annotation of the existing oriental river prawn reference genome.

Since the introduction of large-scale sequencing platform, transcriptomic sequencing has received considerable attention in the research of gene expression and regulation [12]. Next-generation sequencing (NGS), including the Illumina platform, has been widely employed for transcriptome and genome analyses in many species because of its multiple advantages, such as accuracy and cost-effective [13–16]. However, the NGS in short amplified fragments makes the reconstruction task more complicated, and increase the difficulty of accurate full-length splice isoform prediction [17, 18]. Recently, the PacBio (PB) Single-Molecule Real-Time (SMRT) sequencing, as a representative of the third generation sequencing (TGS) technology, can directly obtain full-length splice isoforms without assembly, thereby overcoming the limitations of short-read sequences and allowing the identification of rare or novel splice variants [19–22]. At present, PB sequencing has been widely employed in different species, including pearl oyster (*Pinctada fucata martensii*) [23], Cattle (*Bos taurus*) [24], rabbit (*Oryctolagus cuniculus*) [25], Chinese chive maggots (*Bradysia odoriphaga*) [26] and sedges (*Carex breviculmis*) [27]. However, PB sequencing still possesses some disadvantages such as low throughput and high sequencing error rates [28, 29]. Therefore, a combined strategy of SMRT sequencing and Illumina RNA-seq data to complement each other has became increasingly important [30–32]. In this study, we gained a full-length transcriptome from oriental river prawn muscle through PacBio SMRT and Illumina sequencing, and identified NGs, structural variations, AS events, TFs and lncRNAs. These data will improve our understanding of the structural variations and complexity of the transcripts, and provide a strong basis for further genomic research on this prawn species.

## Results

### Baseline characteristics of the SMRT sequences of oriental river prawn

**Fig. 1:**
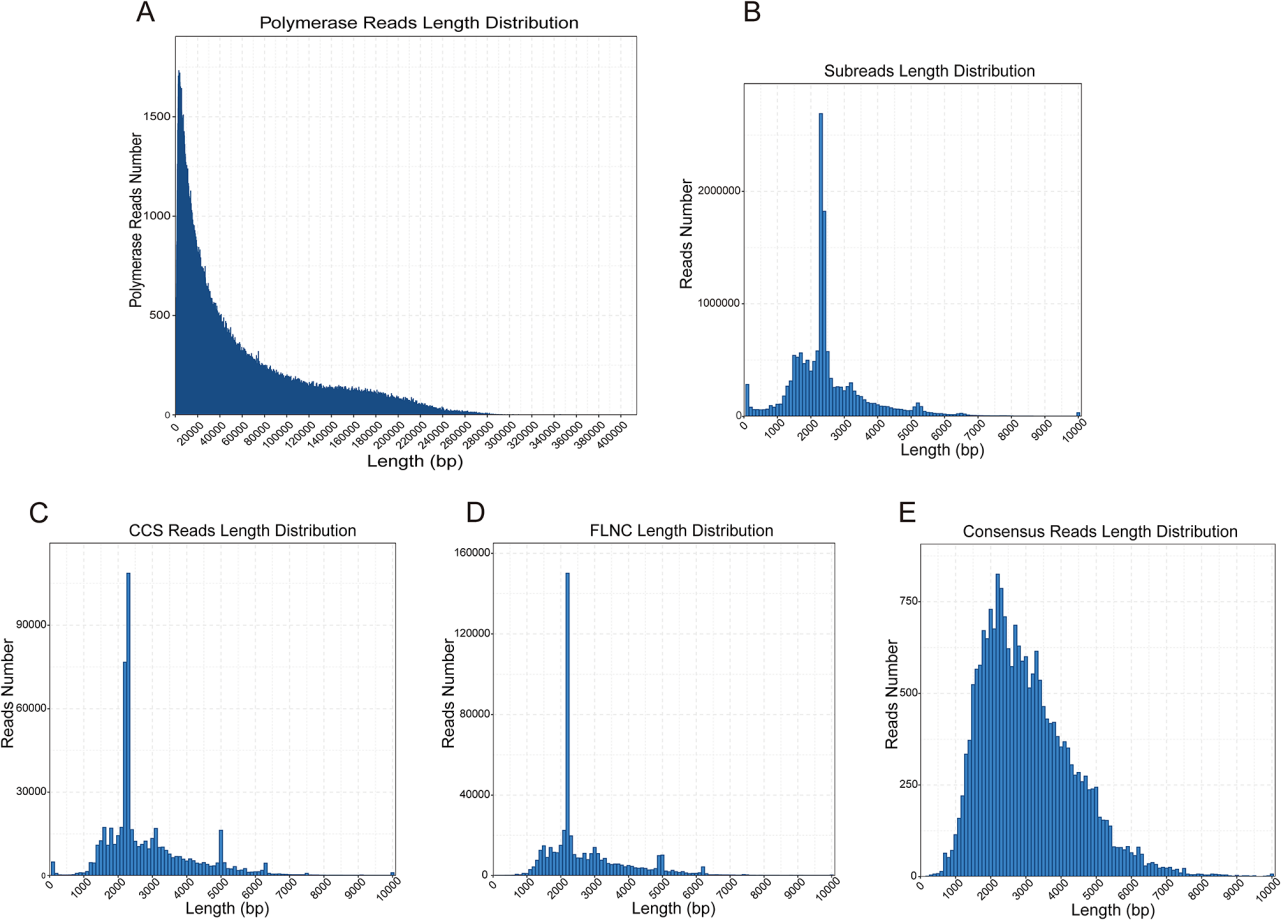
Length distributions of SMRT sequences. (**A**) length distributions of 667,816 polymerase read. (**B**) length distributions of 15,481,437 subreads sequences. (**C**) length distributions of 584,498 CCS sequences. (**D**) length distributions of 512,216 FLNC sequences. (**E**) length distributions of 21,008 consensus sequences

To further investigate the transcriptome complexity of oriental river prawn, its muscle tissues were collected to extract total RNA, and the SMRT library was constructed for sequencing by using the PB Sequel platform. About 39.47 Gb of raw data consisting 667,816 raw polymerase reads were obtained. In total, 15,481,437 subreads (37.99 Gb) were identified, with the average read length and N50 length of 2,454 and 2,393 bp, respectively. To retrieve more accurate sequencing data, 584,498 circular consensus sequences (CCSs) were identified from subreads that pass at least 2 times through the insert. Of them, 513,236 CCSs belonged to full-length reads, and 512,216 full-length non-chimeric (FLNC) reads with a mean read length of 2,701 bp were identified. Next, all FLNC reads were clustered to remove redundancy and corrected by Arrow software, which finally obtained 21,008 polished consensus sequences. The average length of polished consensus reads was 3,009 bp. To correct the high error rate of PB long reads, ∼634.2 million clean reads were generated using the Illumina platform. Next, LoRDEC software was used to correct the PB long reads based on the Illumina short reads. Lastly, 21,008 corrected sequences were obtained, with the average read length and N50 length of 3,007 and 3,396 bp, respectively. The length distributions of all the above sequences are shown in Fig. 1; Table 1.

**Table 1:** Summary of PB SMRT sequencing reads

	Polymerase reads	Subreads	CCS	FLNC	Corrected consensus
Number	667,816	15,481,437	484,498	512,216	21,008
Mean length (bp)	59,109	2,454	2,796	2,701	3,009
N50	117,297	2,393	2,942	2,776	3,396

### Genome mapping

**Fig. 2:**
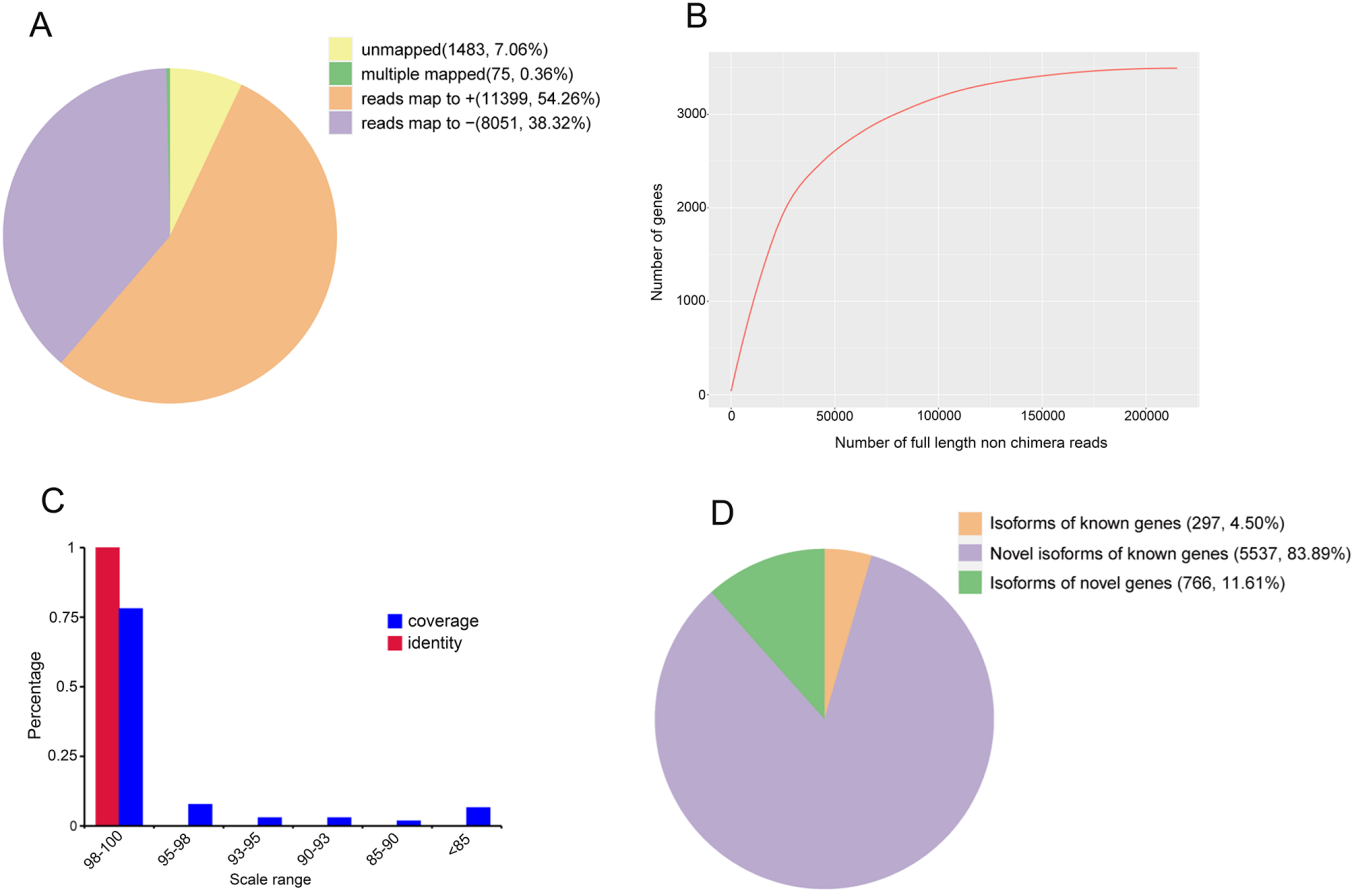
GMAP analysis of SMRT sequences. (**A**) GMAP mapping of the corrected sequences. (**B**) Saturation curve of the corrected sequences. (**C**) Sequence identity and coverage. (**D**) Classification of the identified transcript isoforms

All the corrected sequences were compared against the oriental river prawn reference genome (ASM1510439v1) via GMAP software. In total, 19,525 reads (92.94%) were mapped to the reference genome. As shown in Fig. 2A, these reads were divided into 4 groups: mapped to plus (+), mapped to minus (−), multiple mapped and unmapped. These four groups comprised of 11,399 reads (54.26%) mapped to the positive strand, 8,051 reads (38.32%) mapped to the opposite strand, 75 reads (0.36%) with multiple alignments and 1,483 reads (7.06%) without any mapping to the reference genome, respectively. A saturation level was observed in the curve of the corrected isoform numbers (Fig. 2B), and 75% high-quality reads with identity and coverage values of > 98% were identified (Fig. 2C).

After correction, the transcript sequences were mapped against the reference genome. The Genome Mapping and Alignment Program (GMAP) output file and genome annotation file (http://gigadb.org/dataset/100843) were employed for the analysis of transcript and gene isoforms. Reads that were mapped to different exons in known gene regions were defined as new isoforms, and isoforms that spanned more than one gene were excluded from downstream splice analysis. Subsequently, 6,599 isoforms were generated, which could be assigned to 3 groups: (i) 296 isoforms of known genes; (ii) 5,537 novel isoforms from known genes; and (iii) 766 isoforms from NGs (Fig. 2D). Additionally, 620 NGs (no annotation in reference genome) were also identified (Table S1).

### Functional annotation of NGs

**Fig. 3:**
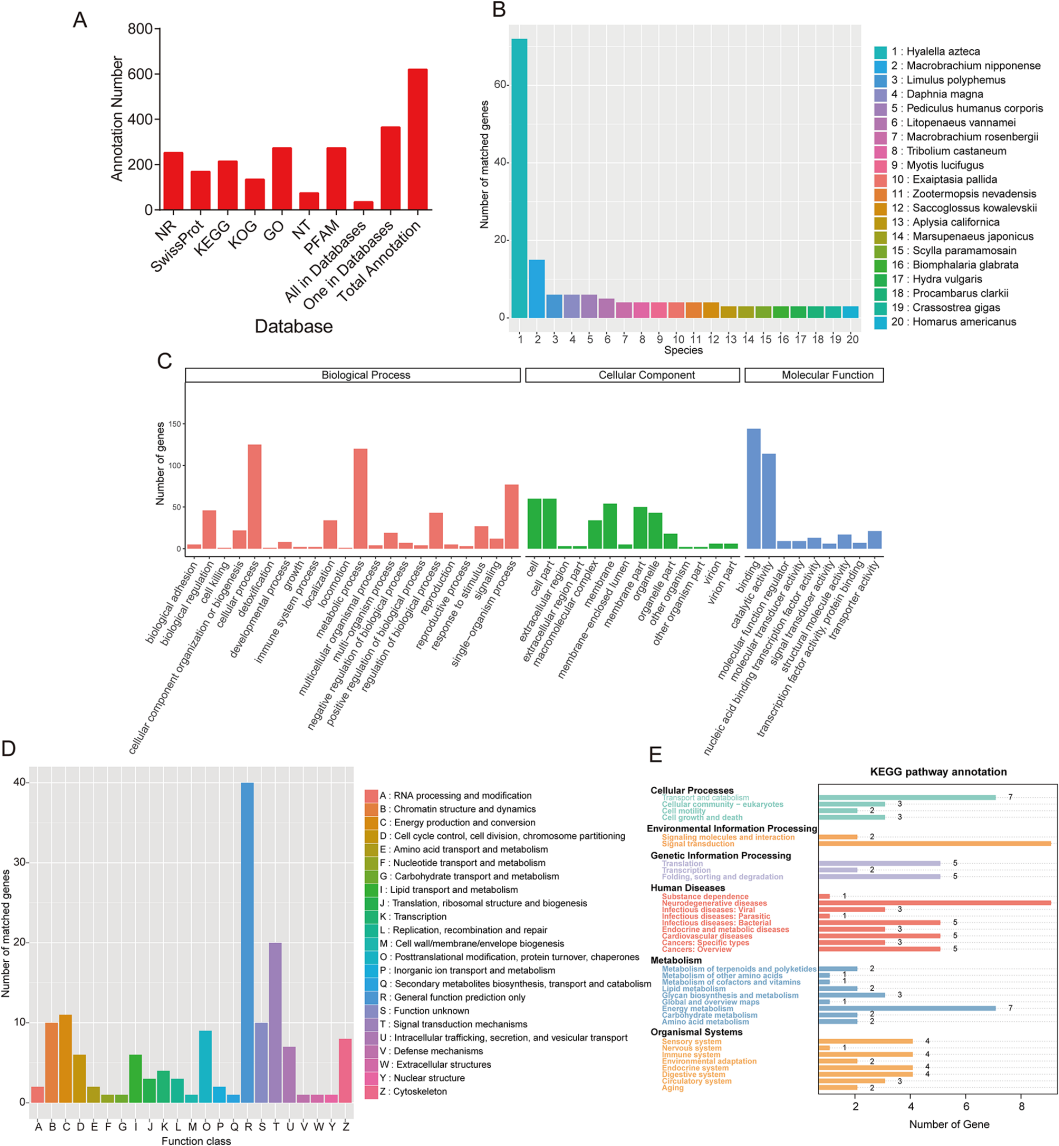
Functional annotation of NGs. (**A**) Functional annotation of NGs across seven databases. (**B**) Nr homologous species distribution of NGs. (**C**) Distribution of GO terms for all annotated transcripts. (**D**) KOG enrichment of NGs. (**E**) KEGG pathway enrichment of NGs.

To enhance functional annotation, 620 NGs were annotated by NCBI-Nt, NCBI-Nr, Pfam, KOG, GO, KEGG and Swissprot databases. There were 35 genes overlapped across the 7 databases, and 365 genes were detected in at least 1 database (Fig. 3A and Table S2). The NGs were compared against the Nr database to identify homologous genes. It was found that the top 5 NGs have homologues in *Hyalella azteca* (64), *Macrobrachium nipponense* (28), *Limulus polyphemus* (20), *Daphnia magna* (12), and *Pediculus humanus corporis* (10) (Fig. 3B). Moreover, GO analysis revealed that “cellular process”, “metabolic process”, and “single-organism process” were significantly enriched in the “biological process”, “Cell”, “Cell part”, and “membrane” were significantly enriched in the “cellular components”, and “binding” and “catalytic activity” were significantly enriched in the “molecular functions”(Fig. 3C). KOG analysis demonstrated that the NGs were clustered into 23 functional groups, and the “General function prediction only”, “Signal transduction mechanisms”, and “Energy production and conversion” ranked as the three most common categories (Fig. 3D). KEGG analysis indicated that the NGs were mapped to 110 KEGG pathways (Fig. 3E).

### Determination of alternative splicing (AS) and alternative polyadenylation (APA)

**Fig. 4:**
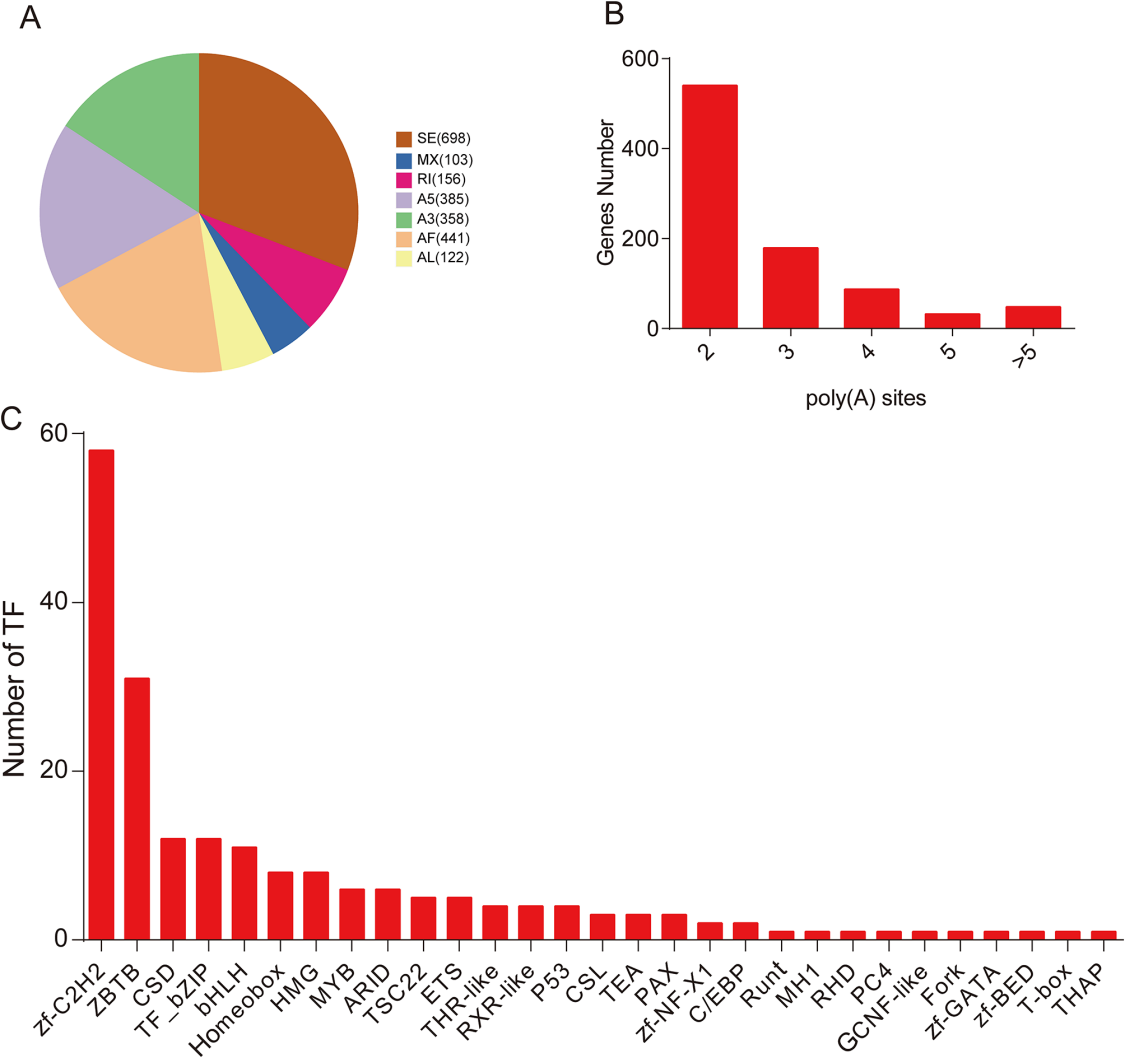
Identification of AS, APA events and transcription factors according to the SMRT sequences. (**A**) Number and category of the identified AS events. (**B**) The number of poly(A) sites in each gene. (**C**) Number and type of the identified transcription factors

In this study, SUPPA software was applied to determine AS events [33]. Seven types of AS events were identified, including alternative first exon (AF), alternative 3’ splice site (A3), alternative 5’ splice site (A5), alternative last exon (AL), mutually exclusive exon (MX), retained intron (RI) and skipped exon (SE). A total of 2,263 AS events were found from 3,605 gens (Table S3). Four kinds of events, SE (698), AF (441), A5 (385) and A3 (358) were relatively more common than other three AS events (Fig. 4A). PB sequencing also allows the determination of APA sites. A total of 2,555, poly(A) sites were detected in 886 genes, among which 80 and 540 genes had 5 and 2 poly(A) sites, respectively (Fig. 4B and Table S4). The mean number of poly(A) sites in each gene was 2.88.

### Identification of TFs and lncRNAs

TFs play vital roles in regulating animal growth and development. The animal TFDB 2.0 database was employed to identify and classify TFs [34]. In total, 199 putative TFs were identified from 29 families, among which 16 novel TFs were identified. The numbers of enriched TF families were as follows: zf-C2H2 (58), ZBTB (31), CSD (12), TF_bZIP (12), bHLH (11), Homeobox (8), HMG (8), MYB (6) and ARID (6) (Fig. 4C and Table S5).

**Fig. 5:**
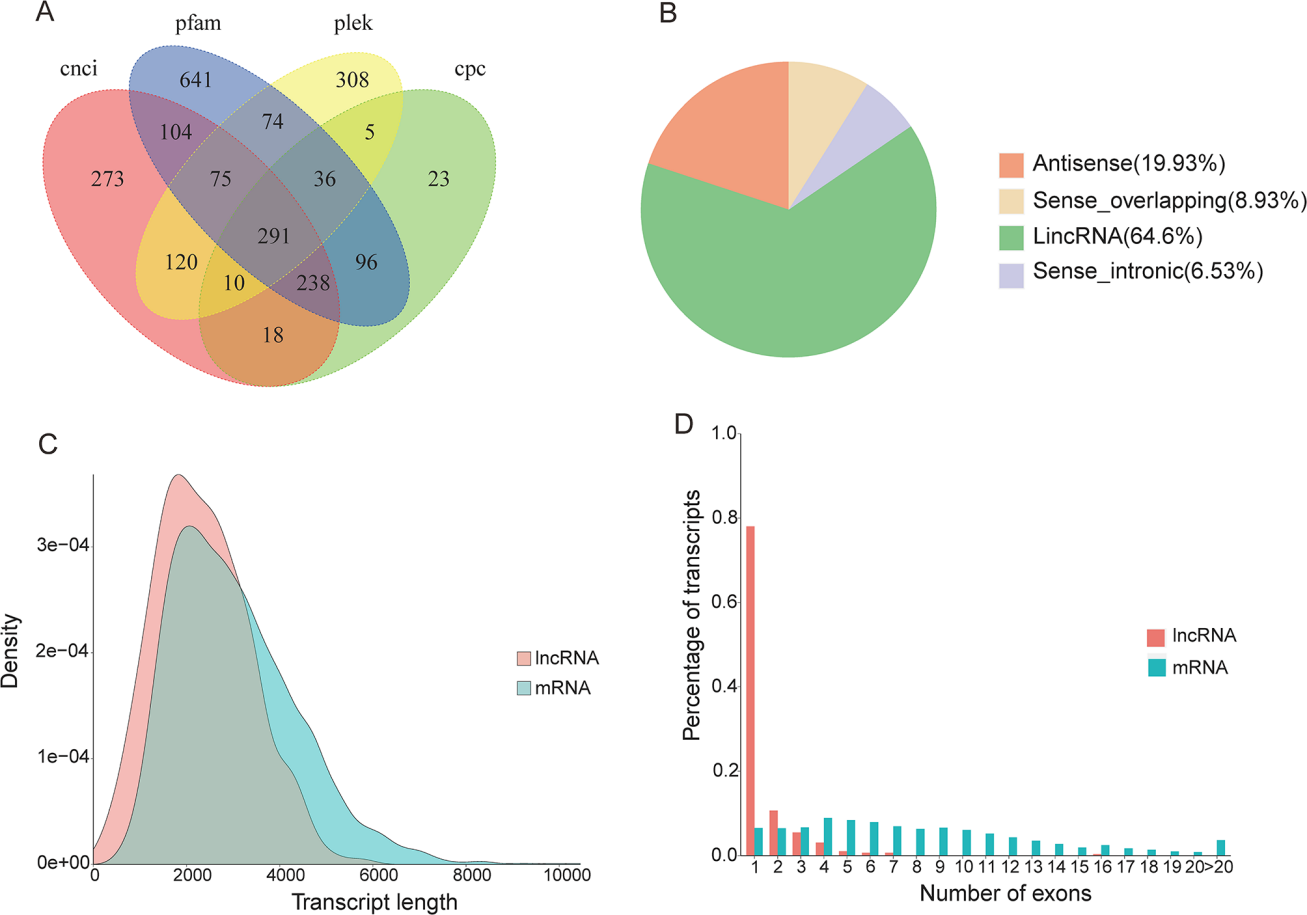
Identification of lncRNA according to the SMRT sequences. (**A**) Venn diagram of lncRNA estimated by CPC, CNCI, Pfam, and PLEK tools. (**B)** Classification of the lncRNA types. (**C**) Length and density distributions of the annotated lncRNA and mRNA. (**D**) Comparison of the exon numbers of the annotated lncRNA and mRNA.

According to the prediction results of CNCI, CPC, Pfam, and PLEK tools, 2,312 transcripts were regarded as putative non-coding RNAs. The 291 transcripts obtained from all four prediction tools were deemed as lncRNAs, and 203 (69.76%) of them were novel lncRNAs (Fig. 5A and Table S6). These lncRNAs were divided into 4 groups: antisense lncRNA (n = 58, 19.93%), sense intronic lncRNA (n = 19, 6.53%), sense overlapping lncRNA (n = 26, 8.93%), and lincRNA (n = 188, 64.60%) (Fig. 5B). Length distribution analysis showed that the lengths of lncRNAs ranged from 0.24 to 5.75 kb, and the average length was 2.34 kb (Fig. 5C). Additionally, The lncRNAs predicted have fewer exons when compared to the mRNAs and 228 (78.35%) single-exon lncRNAs were identified (Fig. 5D).

## Discussion

The NGS technologies have been widely used to construct genome and transcriptome with significant advantages including accurate, cost-effective and high throughput [13–16, 35]. Therefore, the data about oriental river prawn transcriptome based on gene expression profiling and genome were mainly produced by NGS sequencing [36–41]. However, the fusion transcripts, full-length mRNAs, AS events and APA sites of oriental river prawn have not been well characterized due to the lack of full-length transcripts. PB SMRT sequencing can be used to directly obtain full-length transcripts without further assembly, thus overcoming the above-mentioned limitations [42–45].

The transcriptome analysis of oriental river prawn mainly involves gonads and hepatopancreas tissues in the previous study. For instance, a total of 78,408 isosequences were obtained in de novo transcriptome assembly data of oriental river prawn androgenic gland tissues, among which contain 57,619 non-redundant transcripts and 40 candidate NGs [3]. Besides, by transcriptome analysis of oriental river prawn ovarian, a total of 63,336 unigenes were assembled, and among 9 key DEGs may be related to sexual precocity [5]. Hepatopancreas is the largest functional organ of shrimp so its transcriptome studies are the most common in oriental river prawn. These transcriptome sequencing produced large number of unigenes by using Illumina platform, and revealed differential gene expression profile and related signaling pathways enrichment rule under a variety of treatment conditions [46–48].This study is the first transcriptome analysis of oriental river prawn muscle using a hybrid sequencing approach. In total, 37.99 Gb subread data were retrieved and 584,498 CCS sequences were generated after correction. By detecting the sequences, 512,216 FLNC sequences were identified with an average length of 2,701 bp. After eliminating duplicate sequences, 21,008 consensus sequences were acquired. Moreover, the paired-end reads were retrieved using the Illumina platform, and were then employed to correct the consensus isoform sequences after quality filtering. Lastly, the combination of SMRT with Illumina data generated a total of 21,008 corrected consensus reads. After mapping the consensus reads against the oriental river prawn reference genome, the mapping rate was 92.94% (> 70%), indicating the quality of the sequencing data is good [49]. In the previous work, multiple isoforms of anti-lipopolysaccharide factors (ALFs) were identified from the ridgetail prawn *Exopalaemon carinicauda* and showed different function in modulating the in vivo bacterial and viral propagation [50]. Base on proteomics informed by transcriptomics, eleven different black tiger shrimp *Penaeus monodon* hemocyanin (PmoHc) γ isoforms and one PmoHc β isoform were successfully identified in black tiger shrimp *P. monodon* and showed specific expression patterns in shrimp different stages of development. The average identity of amino acid sequence ranged from 24 to 97% between putative PmoHc gene isoforms [51]. In this study, 6,599 high-quality isoforms were obtained based on the PB full-length sequences, among which 5,537 and 766 were classified as novel isoforms from known genes and isoforms from NGs, respectively. These isoforms may effectively enrich the diversity of proteins in oriental river prawn.

Previous studies have shown that eukaryotic transcriptome is highly complex due to posttranscriptional processing (e.g., AS and APA) of precursor mRNAs [18, 52]. Here, AS and APA events were identified from oriental river prawn by using PB sequences. AS has contributed greatly to enrich the functional and structural polymorphisms of genes and proteins [53–55]. In freshwater giant prawn (*Macrobrachium rosenbergii*), two crustacean hyperglycemic hormone (chh and chh- l) isoforms were identified and demonstrated to come from a Chh gene transcribed in an AS manner. The chh transcript contains exons I, II, and IV, whereas the chh-l transcript contains all 4 exons [56]. In addition, two Cactus (MnCactus-a and MnCactus-b) and four Taiman (MnTai-A, MnTai-B, MnTai-C, and MnTai-D) isoforms were characterized from oriental river prawns and proved to produce by AS [57, 58]. Cactus-a encodes a protein of 377 amino acids (aa) and Cactus-b encodes a protein of 471 aa [57]. The full-length cDNA of MnTai-A contains all exons (20) and encoded a protein of 1665 aa. The second to last (-exon2) and the third to last (-exon3) exons can be AS, and the deprivation of -exon2 or -exon3 produces MnTai-B or MnTai-C, respectively, whereas both exons are absent in MnTai-D. All these four isoforms were ubiquitous in a variety of tissues [58]. In this study, 2,263 AS events were found among 3,605 genes, which may provide more new knowledge about the complexity and diversity of isoforms of transcripts and corresponding proteins. For example, The full-length cDNA of twitchin-like contains a total of 9 exons, the variable splicing occurred on the fifth exon, eventually resulting in a splice variant containing 8 exons. PB sequencing is more effective than NGS for analyzing poly(A) sites [18, 59, 60]. In this study, a draft genome map of APA was constructed, which consisted of 2,555 poly(A) sites in 886 genes. These data may underestimate the exact number of APA genes due to the downregulated expression of proximal poly(A) sites.

LncRNA has been characterized in many species, which plays important parts in developmental and pathological processes [61, 62]. However, the lncRNAs identified by NGS are inaccurate due to a lack of poly(A) tails [63]. In our study, 291 lncRNAs were predicted according to SMRT sequencing data and 203 of these were identified as NGs, which could serve as lncRNA candidates for future functional characterization. In many species, NGs detected by full-length transcript sequencing effectively supplemented the reference genome data, such as Cattle (*Bos taurus*) [24], Gnetales (*Gnetum*) [42], and Perennial ryegrass (*Lolium perenne*) [64]. In this study, 620 NGs were detected when these transcripts were mapped with the oriental river prawn reference genome, which provided more comprehensive supplement data for genome sequences and gene functions in oriental river prawn.

Taken altogether, the current study represents an example of PB SMRT sequencing insights into the transcriptome complexity and diversity of oriental river prawn, which characterized full-length transcript and refined the annotation of the reference genome. These findings are beneficial for molecular breeding of oriental river prawn.

## Conclusion

In summary, we identified 2,263 AS events, 2,555 APA sites, 620 NGs, 291 novel lncRNAs, and 197 TFs based on the full-length transcriptome analysis of oriental river prawn, which provided a strong molecular basis for exploring the transcriptome diversity of oriental river prawn. In addition, these data can be useful for elucidating the transcriptomic profile, understanding the genomic structure, and improving the draft genome annotation of oriental river prawn.

## Materials and methods

### Sample collection and RNA preparation

Specimens of 1-year-old adult oriental river prawn were collected from a wild population in Minjiang river, Sichuan, China. 5 individuals with body weights of 11.01–13.45 g were selected for sequencing. Fresh muscle tissues of 5 individuals were collected and immediately frozen in liquid nitrogen before carrying out RNA extraction. Subsequently, total RNA from muscle tissues were extracted by using TRIzol reagent (Takara, Japan) according to the manufacturer’s instructions. The quality and quantity were assessed by agarose gel electrophoresis and Agilent Bioanalyzer 2100 System (Agilent, USA), respectively. The qualified RNA specimens were subjected to cDNA library construction and sequencing. We hereby declare that the study is reported in accordance with ARRIVE guidelines.

### SMRT library preparation and PB sequencing

First, the qualified RNA samples were equally pooled together. Then, full-length cDNA synthesis was conducted using the SMARTer PCR cDNA Synthesis Kit (Clontech, USA). Next, the BluePippinTM Size Selection System (Sage Science, USA) was applied for cDNA size fractionation and length selection. Subsequently, the PB library was prepared using the SMRTbell Express Template Prep Kit 2.0 (Pacific Biosciences, USA). Lastly, the PB Sequel platform was used for SMRT sequencing.

### Illumina cDNA library construction and NGS analysis

Total RNA was extracted from independent biological replicates and prepared for double-stranded cDNA library construction. The first and second cDNA strands successively were synthesized using a NEBNext® Ultra™ RNA Library Prep Kit (NEB, USA). Next, an Illumina NovaSeq 6000 platform was used to sequence the qualified libraries to generate raw paired-end reads with 150-bp read length. Quality filtering was conducted with NGS QC Toolkit v2.3.3 [65], such as trimming the first five bases at the 5’-end and removing reads containing the low-quality bases (QA ≤ 30) > 20% or ambiguous bases > 1%. Finally, the obtained Illumina clean reads were assembled independently using Stringtie v2.1.1 and Hisat2 v2.1.0 for correcting PB long reads [66, 67].

### Quality filtering and error correction

SMRTlink v8.0 software was employed to process the PB raw data based on the following parameters: minPasses = 1, minLength = 50, maxLength = 15000. CCSs were generated from the subread.bam files (parameters: min_length 200, max_drop_fraction 0.8, no_polish TRUE, min_zscore − 9999, min_passes 1, min_predicted_accuracy 0.8, max_length 18,000), and then these CCS.bam file were output. By searching for the poly(A) tail and the 5’ and 3’ adapters, the CCSs were classified into full-length and non-full-length reads. Full-length reads without chimeras were defined as FLNC reads. Then, these FLNC reads were clustered to clear redundancy by Iterative Clustering for Error Correction (ICE) and then corrected to obtain high-quality (post-correction accuracy above 99%) polished consensus reads by SMRT-Link built-in Arrow software (https://github.com/PacificBiosciences/pbbioconda). The LoRDEC v0.7 software [68] was employed to correct mismatches and nucleotide indels in consensus reads (parameters: -k 23; -s 3). Construction of a high-quality PB corrected consensus read dataset without redundant isoforms was then performed.

### Mapping to the reference genome and structural analysis

GMAP v2017-06-20 [49] was used to align the corrected isoforms against the oriental river prawn reference genome (ASM1510439v1) based on the following parameters: –no-chimeras, –expand-offsets 1 - B 5 -f samse -n 1. The genome annotation file (http://gigadb.org/dataset/100843) was employed for the determination of genes and transcripts. Genome-guided transcriptome assembly was then carried out. Structure analysis of the transcripts was conducted using the TAPIS pipeline v1.2.1 [18]. AS events were identified and classified by SUPPA v2.3 [33]. TAPIS was used to analyze the APA events. The animal TFDB 2.0 database was employed for predicting transcription factors (TFs) [34].

Because of the limitation of library construction, only polyA tails-containing lncRNAs were obtained. The coding potential was calculated using the CNCI [69], PLEK [70], CPC [71], and Pfam database [72]. The transcripts (> 200 bp) with at least 2 exons were chosen as lncRNA candidates. To ensure the accuracy of the results, only the lncRNAs identified simultaneously from the four tools were retained for further analysis.

### Identification and functional annotation of novel genes

NGs were defined as those (compared to GigaDB gene-build) that did not match any annotation in the oriental river prawn reference genome (ASM1510439v1). The identified NGs were annotated by 7 databases, including NCBI-Nr (NCBI non-redundant protein sequences), NCBI-Nt (NCBI non-redundant nucleotide sequences), KOG/COG [73], Pfam [72], SwissProt [74], GO [75], and KEGG [76]. NCBI-BLAST (https://blast.ncbi.nlm.nih.gov/Blast.cgi) was employed for Nt analysis; Hmmscan (https://www.ebi.ac.uk/Tools/hmmer/search/hmmscan) for Pfam analysis; Diamond v0.8.36 [77] for Nr, KOG/COG, KEGG, and Swiss-Prot analyses; and the E-value was set as “1e-5”.

## Electronic supplementary material

Below is the link to the electronic supplementary material.

Supplementary Material 1

Supplementary Material 2

Supplementary Material 3

Supplementary Material 4

Supplementary Material 5

Supplementary Material 6

Supplementary Material 7

Supplementary Material 8

Supplementary Material 9

Supplementary Material 10

Supplementary Material 11

Supplementary Material 12

Supplementary Material 13

## Data Availability

The raw bam files and Illumina RNA-Seq data have been deposited in the Sequence Read Archives (SRA) of the National Center for Biotechnology Information (NCBI) under the accession number PRJNA935961 (https://www.ncbi.nlm.nih.gov/bioproject/?term=PRJNA935961) and PRJNA902553 (https://www.ncbi.nlm.nih.gov/bioproject/?term=PRJNA902553), respectively.
