# Supplementary material for: PacBio single-molecule long-read sequencing provides new insights into the complexity of full-length transcripts in oriental river prawn, macrobrachium nipponense
Source: BMC Genomics. 2023 Jun 20;24:340. doi: 10.1186/s12864-023-09442-x (PMC10280851; doi:10.1186/s12864-023-09442-x)
Supplement: Supplementary file 9 — Supplementary Material 9 [file 12864_2023_9442_MOESM9_ESM.docx]

The amino acid sequence of transcription factors

>Novelgene0172_novel01

MPSKSSSNFYLHKLRGFHLKRLKRQKRQASTSLKFVNSSIPSLQTSGTILLETKESSPDL

CHVRSSASVVKRYKEIKPKNITTHTLKLQDGRPLMNTSNSDVKEVGKISGSSKNYFRHFC

TSSVKHLAQRSLLKSRLLLSRKRANLPDFETRGSDEGKITERKIVSLPNNLTVEAIHYKC

KILQLHDTKCNCSTPRLHIYLTTNHRCGDKCSAESYQYSDNISSNSPADIPRNKYIFAVM

TGKSKAKGQLKKITSDQKVRKTPRKKGVKSLTNTSTFNNKPQGRRKSLMTIRNEEPESEL

LGEECESLPTIMSTLSGDVEYIVLDDNTFNLNANMTNTSQGKDDPRSSNAVEGDVKPEIQ

SLVNAQTQKEGRSWEDILERCLSEVSYQSGRYKGGLNQLQDNKIKSEPAEEGTTTKNTEA

SSSLAEVCTNHCPSIRDIFGDVNIKDEPVDNIDENDMSVEAFSQEIMRLTDAEKSGNGNS

WSVDKSISLDQEISSNKGLFAGLLKEEIKVTKDESKEENKSITEVHIKEENDTNKESKDN

DGDDDYSSDSSLGPLQIDEGEQPPAEEEAVNVPVMPTPATSLILPSVNIISPFKIKIFFG

TPHRVHLEKPHELFEAATVSAPVTGASPIQNSVVSNKSSEKKPNVMEEEDSETDSSQALV

QSEPTPMPSLTCAYCQTVCSDIKALAKHFEDHQRDGLIICYFCQKSFGDKTSMKRHMRTH

TGEKPYQCKVCGKRFSLPGNFKKHRDIHEDIRTEPCEVCGKTFRRKEHLKYHMRTHTGEK

PYTCGECGTSFTARYSLQIHMNIHLGKKPYKCTYCTKAFSDKSTMRKHVRVHTGEKPFHC

EMCGRCFGESGTLAAHMATHRTERPYKCKLCSLCFKTTGGLRQHEKIHTGLKQYACRFCG

VKFLQKYNMTMHERIHTGEKPYSCSHCSRSFRSRSCLAKHVVLHGGEEERRYECEHCNSR

FYRKAHLRRHIDTHLGIKNYECDICSKKFCTRISLVNHLKTFHAQGARRFPCTWCGRVFK

RQVYVHTHECIRDPEKVEQYKNEMRAAAAAAAAGDNTQSKDDKNENSSDSTDYSNIDIKS

EPEELVEEVSGLGSIESVIGNANFDAPPDLKTEDPS*

>Novelgene0173_novel01

MEHPFLDELCNNDFKEKEDESVSKKQPTDVGPYLCKLCGKHFAHKGNLNRHMKLQVCDRP

FSCERCRKTFAQEEMLFKHREKHTRDRPYTCEVCGRGFTERSNLRTHMMYHTGERPFSCE

VCGKTFVGRGNLNIHIRKHTGERPYSCEICGKSFPQKEHLNKHIRCHTRERPYVCELCGK

TFIEHGHLTTHRKHHKEERPFKCEVCGKGFVERCNLKQHLRKHTGERPYSCEFCDKDFTR

KGILNIHLRLHTGERPFICDVCNRGFTRKDNLKLHKRQHTGELPFACDACGRCYAEKGKL

KTHMRKHTGERPFACKICGKTFTENGKLTRHMRQHTGERPFECGVCGMKFIQKNHLNKHM

RRHNGVGPVSRRNANTSGNSEPDTLTGKVEISEIISIPEELENIFTDGNIPDVDTELHCQ

SDLSLVNSLCENNEPEDCLSENACGTNLMRLVDPSEISREFIKRESEDTDISLAEVKVEE

SLFSDTLMNVSV*

>Novelgene0273_novel01

MDGKMKKYECEICGKHFMLKSSLKVHVLNHKSYGMFWDVDKSHVKPIEEMSPPCWRQEDD

LVAYHSQDSVQDFENADCTEKDKFYKCPFCGKLYSLKDCLKTHVVKCKIATSSGAGESTE

MSPLGHQCTTTESNRLDPDLKSLVPEDKEKFHVKQGDLISESIPIGIFSHSLNAKRLSDK

EQDLSTFDYPGKGLERKKTLVIGEPMNVNVALSFDNESIMKDVEVFSIQDTESNTSRNAG

KCLRQSNLGNEEKHYMKSHPDLVCPVCESIFSSKAMLSEHNEQRSHVCKVCSQSYFSCKD

LASHEKSHYKYSEVCDINFALYEDAVIHKRSHSSKQRCSTCKKLFSTSSVLHRHLEQESY

KCNICKEIFSSCKDAKIHRKSHLKSQCRTCHKTFAAVEKDSHTCHDYKTLSVHRGTRCKN

FCPACQKLFPSEDLLREHMQQDSYICEICLKDFTGCKHLKSHMESHKLKCKICKKQFLCP

DNLKEHELKHSDEDLTCKYCHKKFKSLGNVKRHIERSHYGIKDVQCDICGKMFHKSLFKI

HRRIHTGERPFQCIQCNATFVQKAHLTTHMKSHTGERNYPCNLCSKTFISKETLTIHQRY

HTGEKPYECDQCGKRFRSSQLLANHFRTHRKERPFKCNICLKDFKFRTSLWQHTKTHFPD

QLPCCKVCGKRFNYKYMLHAHYRRHSEEELQNAGMESLSLELEEKFHRPEFKTFDCKYCG

KHFYYKARLQSHERLHTGEKPHKCSFCEKSFRLPVSLKNHERIHTGERPYKCNICDKTFV

QQVHLKVHMVMHTGARPYECSFCDKTFAHQSNLLAHRKIHLRVTNADPVSAS*

>Novelgene0296_novel01

NDCGKAFSRNDTLKIHMRVHSGEKPFKCDDCGKAFSQKASLKIHMRDHTEEKTFKCSNCW

KPLTQKDHLSGHKKALVVTEFFARNLGRNVTLKTIICIPE*

>Novelgene0374_novel01

MKKQIMYKHSLPSLQDWESKRPAGPPSSLPVCVSAKPSCEGSTLPRNRVPDDFEAVQTLL

SFSQQHVMKHDSNFSHTANSYFPPSPPSSQGSISPMYQSLESDAEESGDHSPHKKSKFSD

SDLEKVLTNQMALPHMPCTPPRSPSPSFTGYCSRSSTPSSLAGVPVSVIVKATRQPKQHE

NKCAVVEPLKSPPHRIVHTAQEQIFVCSKDTDRELDVSSVSGNSSSPILPVLPPASVEPA

SKTMSKGMQNCNKLVAIAPKIPSVIPVKPGTPIILAQVNGPSTMIPVSNPGITHLIVAPQ

GSQISSSVFSPLFISAGPVQQGSQEDRRKTFECPFPDCDKTYYKSSHLKSHMRSHTGEKP

YKCSWEGCERRFARSDELSRHKRTHTGEKKFGCPSCSAKFMRSDHLSKHMKRHALRRIGV

PVAPKVSTLAPAISFIAVPTLPQ*

>Novelgene0514_novel01

MFHRFHDLKTGQDPEMVGGGSRSSTPSEPVDGRALFPSVIRSELSAPGNQLRVNPQYPIA

SSPGSGYVGQGPPLMSLGHNIMFPPGKLLATPSQEAHRYMTEAYRIAASELESSTVSYGS

PAGRPPVESQSSVTDTHKSPPDGLPVAAGNRHGSISNRPHGVADGHLPAGRDIYNPINLT

PQINSEPSQIDSDAPRGLAELSYNITRSPSNKVEGEPLSYEKSTYSGGVVSGVSQPPSAS

PKAKCPPISIGGPHLFQGPFTSAYEQYGDGITGNFKRPYYDDSSGRREGLSHPQSNVGNS

PQVIRGEGSPSSGDQVQESIGFNSGCPDVDLYSREGQSRTGSADPGSHLNLDLLTRATSS

QAGSLERPKLMKGRQLKAYKCDICELEFKNGTQLKNHAWRHTGEKPFSCDLCQATFTQQS

NLKTHLRIHTGERPYTCEECNATFTQISNLRTHQKIHTGEKPYECDICFTRFSQQSNLKS

HKLIHTGERPFSCEECGATFVQSTHLRNHKRIHTDERPYTCDQCGGSFRQLSNLKTHEKI

HTGERPHVCEECGSAFAQKSNLKSHMLKLHSNDGAPIRRGRKKKLEAIVPFICEECGAKF

TLMSNLKIHMRLHTGEKPFVCTICSASFAQRSNLKAHEQIHTDERPYKCLECPAAFRQKT

NLKTHKMKKHPVKSLKIKILSDTQYIMHDIMHDIIQSEDNEGEGPSQHIEEPIVQIREGK

DDHLLLGGGESIKSESGDRYEDHNESMAETADRYEDHSEALTESNAFRQDTMGAAAINPY

DSPNF*

>Novelgene0515_novel01

MNPELPEQVHVKSENIHVKSEKVLIKNEEVHEESETEDTRFIFPVKAEVKTEVEDFEDPL

SISELNREFSAETEDPLSVSPASHDGCSAISDDIVSGLFDPIVIVKTESEKCEGKEDPEG

YEENEGEVKYSCIIDKEDCNIIGNPVVIHTEREKLISSEWERAILKSNIKADPNICTGKK

PLTCLICGKTFGRKSHLKDHIICHTGEKPFRCSECGKGFSQKGHLTVHMNSHTGQRPHKC

PECGRAFSQKGHLTVHMNCHTGAKPFKCNECGKAYSHKNNFLSHMIIHTGEEPFRCGECG

KAFYARSKLLRHMNTHTGEKPYKCSKCPKGYADKRDLTKHMTSHNGE*

>Novelgene0518_novel01

MDSKEDTEPSANQPQDPVASADIDASSSSAENAQTDSTVSGDAKSSDRKEEVDDGIVEET

LKNSENINAGEDTRDSPEAQHEVSATGESSLNSIVSDSSPNTHAPESSLKKQTVRLHKKH

LYPLSVPSRTVIDKKIVSTKKKAPVIIRNISEIVAKESDNDDVDSESQVHASSRCEESSH

SKTIYISQSPNHEEFKENKQLTLVSHAENASTVDRSGPSKFKGNFVNDNESYKKGTLTPS

VGSSQSSPYNTRYAHLKIGTIFSVSNKSSGISRKKYDKDSVKVVKSHANKYSTGLRCENL

NVVEENSDNGVCFLCGISFDDDMGISLSVKKPLPLSNVDVITLLESFSISCAAVKAENLN

FVNICNVCHTLIIEGDSSYKHLLSVASEMREQWPYRAMKSNPVFSLSIPDSTLNQFNSPD

GVSERVAASEKQESNHSEYRKVRGRVGRPPKIKVKQEDDDDINWKPSQVKLERTTSTGRR

GRPPKRKLKREKRGRPRKVIVKGEISENESFTCGLCSEEFLEESDWSNHYESVHNTQLRW

VNYRSQKKLKKLILYNIQESFLNEDFTEATCHACDSKFFERHAFIEHLRCLHKMVIDDEF

IAALYKYSQGHIENPEELFDSDIEKDIKAASDGTIIKIERESLAFPGKLEKSLKCKVCHL

TLSSEQALKEHTDTNHFSLVVIGNDDSREVVVPEEKSEDGKAEIVSEKSEPLELTCKDDA

ENDDNVCEECEEIFESRHQLLEHKLNIHGSNDDRGQEKGLLRYECEVCGRRIYGLAALKV

HMTKVHYKTVSSFKFSCWMCVYKSQSRSQLGKHMRMKHGKEIMPSVQCETCGKMYGSSYI

AKHIATMHSTHTRNFGCNFCDMKFYDMAGLKFHIYHEHANKVWKCNKCPMEFKKYHQLRQ

HRIFTHSTKVHACMECPKTYKRKSDLTEHVKRLHLQRVVLLCPRCPKQYVNRFKLRNHLM

KHHDVPWEDTLARNYARHQRANNCRRTEEGLLEANKDQQLEEDIFIQESYDHFERQKEMQ

DVSNNSEEQIEEQEYRDHFNRHSEEHEYHVQYRQSNDGILHQQQQRTHHIRPIKMERDDQ

PEEYIEIMEATDTFAKDGVTYVIIEES*

>Novelgene0523_novel01

MNHIAGQPIPQAAGSQPALWYNYDVIMNHPLQVQVAVPSVQVISAAPQTPTPTPTPTPTP

TPTPAPPPTPVPVAVSMIPAKLKKVKKPPPEERERNYCCEVCGATFTNSSNLRIHTKIHL

GVRPFVCSECGKSFTQSSNLKVHNRIHTGERPYKCSECGQTFSRSSHLVGHKRIHTGERP

YICGICGESFYTSSHMRNHVRRHTGEKPYVCQVCGEAFSQSVELRVHFRRHTGEKAFKCR

ECDSVFVSGPELRAHRKVAHSSSKPFRCEKCDRCFRTAKFFVKHELKCSGPRPKRPKGRP

PKYPRDDGVEPSWNKSKKHRNRKPAAPTDRVSRSKSRALRIAQMKEEVLPEEQSYKNQKE

VIPAFEQASDILEDSGSSDLPSESPLKHEGLSESDDHISQLPMVHVMLDNFSSGADGIVL

HSVPSSTLHGLHSLTDESEQLGVMDLSGQQMHEAEHQNDTDPQTTLRLLPTQSMSVVEAD

HQLPLVTDQEHEVSLEHHQITSAMVSQHSMNNHQKLSVATTFQQSMSALPLSVTSSATTG

GSMNAIPQVSAHHQNATNLVSQVSSSQPTHSQQLSAMPAHQHSPAVGSHHQVPAVTSQQH

LPSVTAIHNTSSSHLQLPTINAHQNQVGIGVHQQVTPLPTSIENTVTAHQNLTMASTHQQ

ISTASSHQMLPVVSAHQQQQVSAGPTQQLSTTANTLLLTGVSHHHVVNSHHQLPVVSAHQ

QTVTNHLQLPTVSSRHTLPTILTAHQSSSGVQQHMVAVTAHQNVSSVPQQHLPAITAHQQ

IANTTDQQVSVAHLSNASAVASGVHQQGLVVSAHQQQVVPLLPVYKHSCNLGSIEDNSQV

VYEGGQSFVVDLSSPALTPQVSLVDPPATSHSTRH*

>Novelgene0524_novel01

MALPPNSVNMNSVYMDHFMQVGQTGGHAGWYDYNTFNNLLVSPPVQHPQIPVPQPHVHHQ

EIIPPAVQPKPKQPPVPRQKPRAAPMERTYHCDTCGATFSNSSNLKSHSRIHSGERPFTC

EMCNASFVQSSNLKAHKRIHTGERPYMCSECGQTFSRSSHLTGHKRTHTGERPYICGICQ

DSFVTSTHLRNHMRKHTGERPFSCSVCKAAFAQNASLQIHLRIHTGERPYKCTECAAAFR

SKGDLRSHRKLHTDERPYACCRCGKYFKTNQYLQKHLKKCGAPPTGKKRGRPRKMVQAEP

VLLSVTKKKVNCGSGRGKGRNKGKLVRTRAKSARLNRSVKVEEQDENVCDIHQRDRNEDL

RLVQGGTFVDIMDLEKISKCNILSDSSSSGEPLPQTLDSHMDPLSMQVQEPLPEIKECVE

QIDEIANSGLEQVADVNQTLQISHVEHIDHMPIEAMDRIVQGNPYQEHSM*

>Novelgene0530_novel01

MMHIKEELTLGIFEKMRAEDRSCPEPHFSDDVPRSGFSHSSDLVTVSNHHIMGDSFPDDT

SVTECKQEENIDVQIKEDVSDLTWSCSLCCKCFSQKSDLEMHMKECPDQRPFSSGLNRNS

DDSSSFQYQLNMCIDTPNFSSDFTEKNSKKPFPCEYCGKCFAKKSNLKIHKRQHTGELPY

ACEICKKRFIQKAKLRIHMRKHTGERPFFCEFCGKCFSTRESLKEHVRLHTGERPFVCKV

CEKTFVTRANLKQHNRTHTGEKPFTCPVCNKSFSHNGTLKVHIQQHSDERPFACKMCDKS

FKNKGTLKVHTRLHTGERPYLCEVCGKDFVESNNLRKHMVAHREERSFFCEACGKGFKHK

AALKVHMKLHAGDRPYFCDVCGKDFVNIKEHRKIHNGDRPYLCEFCEKDFVRKDKLQQHI

RTHLTDRPFPCEVCEKRFTLKKTLKVHMQLHSGACPFTCEICGKSFIVKSRLKSHMLIHS

DQPPVSCKICHKHFSSKGNLNKHMKCLHDDQVIRSSIFVDVLKVESEDIPIKQEVLDDSF

SSNELGLNFSP*

>Novelgene0556_novel01

MNPEHSLEFPLKKEIKEPSLPSLITCKNEDLVDYSTADNDGCSLDPMLEVKIKPELYDPN

TSKKEVSEEGSVHVWTGIQDEGDVYFESCKKQKGRLKKQQKIQKEKERFLSIVNGENFPR

KEVLENHVVIHSEEQLMNSELGKPCHESNLKTLVLSHPGEKPFKCENCGKKFGQKYTLKN

HMLIHTGEKPFRCSDCGKGFSQKIHLANHVRIHTGEKPFVCSNCGKAYAQKIQLTDHMRI

HSGEKPFSCNECGKAFSRKSFLRRHMNSHAGVKSFSCPDCERAFSQKIHLTKHMRIHTGE

KPFKCSECGKTFSRKYSLKSHLKCHNR*

>Novelgene0560_novel01

MNWSVLSFLAWCDKAFSEHFVRFCCSFTNLNSCNLQMTDLSSIMDKIRILRSSRRRTYPG

KIVNNNKTTSVTMTPVTNSETTVSDTNHRWVPFIVLDISQPDGNADSAEDKSAEDLKSKL

EDSPEAQKIPALTPPIQINTSAAPKRTTSSISTTSDKTVIKTTTTTKRSSPRKSKRIPVA

CTIMVTSKDIKNSSKVKTKTNQEGDVVTPALIQNKKDSSSAQELTVSLAKTVESSSVVTT

FAKSKKDVSSPMKSNKDASSSAKSNKDASTPTKSNKDIEVVPSKDANFNTTTLAPVGCVI

CSKHFSTLTEMQTHYLSEHKNRRGREHRKRPIEVVNTEPQKMPNLVTVQEKAVPDEPGFK

FPLYVNESDPKCPVCSIAFKNAAEVKNHVKLVHSYRCSECSDTFYTLFEFTGHKCYKGVK

KSKRIRKKSSRSPNVNQEVAKAMKSFVPIQPNLNKSPKSKDEGTSEVFVPPVHQKDADTS

FVPISSSSPTVSNLSNQRSGMQVNTSTKLQITSVPQQPEVSVPSIRTPSPELTIAPIDNK

PKSVKIRVLETKNLRCFKFKKDEIVSESPLSKSELIQEKITQLKNNPHVSVSWIPRLQFK

CDEDDSEYVCGRCNVICDDMDDYMDHIQDCLTITSVTLEKTSNPPRRILKLQKEISSFGF

QETNNNKYSVNKNLMKKLQEVIPLTEEDVRESSPTRTRYLEEVVEQDSSHLSDLMNESHS

DQVVASSTSLSELSLESSMLQIKEEPLDDYQDPSESVLSKAPSNVYHDGLPNIYQLSNSK

GTLPMVVIPVNQTSANKLLSGSELSVKKPQTTKPPSPDSSSSSSQWDASPCDLMIDTDDI

KMEVEEEVIDDYENF*

>Novelgene0569_novel01

MGKNKSQNEFFFYMVAMKPEVEKRLGKKVPMKEMASLVHADWKNLSEAEKQPYKVMSREA

KGSAEKKDCFGVPLSVLHRKEEEKRKKEGDMFKDIGNTIQFHRDGGALHHHRIFVISTSE

YCRTEIGSVPAELSVLCFTFNKGIEREHHVIFKANIPACYAYTAREESESTHCLLDGNVG

VTDLEEVLHGLMQFLLEDLDELPPVYTLEELRGMTEEVLISICGGSLDFKVYSLDYYFQS

MYSAALEHKIPMSIATDQLTNCALDYHPSMPCNWHSLHQKDAGKYCTLSCARRWVFNMCD

HINITNTFGIEPIEGKHLPRKYFPEGTLIDDPRPLVGEWKSVTPQESLFVSSKSSESSLL

PLGSSFQPKNPHPSSLASLKSREPVHKPNANNWSKVVSSTPSHKAASVTSGCSSTLDDTS

DDDFPALGAGYAKGDSKSQHRLGVLGGAGRGILRNPEYEVKDAKGDESQPRVAAFGGIGR

GLFAQINSDRKTADKKGIGHGLVDGKSLPCTPGNRKERWGNTLVD*

>Novelgene0593_novel01

MALTKSVSPVSQNAHTCIQCGKIFRNSVKYMIHKSKHTSHITFISNIRKKYLLNFRYRFC

WKKYHVCNFFRKAYCCGMCQKSFISERTVVNHLKNCPCNTKHKCLFCDYTSNSQSDIINH

TKQHADEQPYQCTKCDKKFVSLGELTDHELVHLPPRYLCFTCGKVFAEKNVLKNHLEAHR

GKQLCIQCGKEYSGEDYFCHTEVVSALCKKCGSRFKYLCLYEKHKKMHGEVVKKMKHRGK

HLCIHCGKEYLGKDHIFHMEFFDELSPCRICGLHFDYICMYEKHKKGHIEALENIKTCRT

RADLCQFIECSEMVVSQAPLIYGYNHVCMICGKGFKDFPSTLVHIEGHVKIESGMTGNVA

SSVCKASREPVNDFQGFDKQLNSNKGSSLTSKHVDVVVFFNATKRKKSSLESDMQLSLKP

REEVLKYNSEENIGLKVNRNYCSVNLSTRSLPHSSYDSQVDENLPGPSKSIKKKVMSSGY

SFSSSEDGSLTIVDHGLTFSKYKAVMAEVSQKISETGESKMKQSLVEPCSLEKVCPSVSS

TLGGDATSKSNRAGKSPIASPGKCVSDDLSRSRLILSEENPSSNNEGRVNIPKDRIALYR

T

>augustus-12798_pilon_pilon_pilon-processed-gene-1.5-mRNA-1-DNA_novel01

MTAIIHRSNVLQHQIMVAETVKYPVNYPALIPLSPTVTGLGGGYSCSSSMLHANFSPAHI

MDQKPSQQQQQPAPQGPPIGSPPQNGQDNALVGLGAPGLHLPSSLDLASHMRKKDIFTQR

KQREFIPDSKKDESYWDRRRRNNEAAKRSREKRRFNDMILEQRVIELSKENHIMKAQLNA

IKEKYGILGESLINIDQVIANMPQSDQIIAINKRTKFNSAIMALGSSPSVTSLLSPESGM

TSPTPTPSYHNPSDNPESPQNYSHYGNTHDDHDSHFDNNEQYPPSSSSDLYYRSSALNLS

THTSNSHSPMSPTTSQMEYSPASDPEICRRSPVGEAGSSLPHKLRHKSHLGDKDAAQSLL

ALQGIKAEPHDPNHEAAEDSVGSSDERDSGISYSSSSSCSDGYPRSSDTTSSSPTNCLMG

DSTSISSSRNQQSLHLQHYQHQQEQHQKQDLDDVAEYENTQLRSELERLASEVATLKYML

VRRPRSEGDSDGSR*

>augustus-12798_pilon_pilon_pilon-processed-gene-1.5-mRNA-1-DNA_novel02

MVAETVKYPVNYPALIPLSPTVTGLGGGYSCSSSMLHANFSPAHIMDQKPSQQQQQPAPQ

GPPIGSPPQNGQDNALVGLGAPGLHLPSSLDLASHMRKKDIFTQRKQREFIPDSKKDESY

WDRRRRNNEAAKRSREKRRFNDMILEQRVIELSKENHIMKAQLNAIKEKYGILGESLINI

DQVIANMPQSDQIIAINKRTKFNSAIMALGSSPSVTSLLSPESGMTSPTPTPSYHNPSDN

PESPQNYSHYGNTHDDHDSHFDNNEQYPPSSSSDLYYRSSALNLSTHTSNSHSPMSPTTS

QMEYSPASDPEICRRSPVGEAGSSLPHKLRHKSHLGDKDAAQSLLALQGIKAEPHDPNHE

AAEDSVGSSDERDSGISYSSSSSCSDGYPRSSDTTSSSPTNCLMGDSTSISSSRNQQSLH

LQHYQHQQEQHQKQDLDDVAEYENTQLRSELERLASEVATLKYMLVRRPRSEGDSDGSR*

>augustus-12798_pilon_pilon_pilon-processed-gene-1.5-mRNA-1-DNA_novel04

MVAETVKYPVNYPALIPLSPTVTGLGGGYSCSSSMLHANFSPAHIMDQKPSQQQQQPAPQ

GPPIGSPPQNGQDNALVGLGAPGLHLPSSLDLASHMRKKDIFTQRKQREFIPDSKKDESY

WDRRRRNNEAAKRSREKRRFNDMILEQRVIELSKENHIMKAQLNAIKEKYGILGESLINI

DQVIANMPQSDQIIAINKRTKFNSAIMALGSSPSVTSLLSPESGMTSPTPTPSYHNPSDN

PESPQNYSHYGNTHDDHDSHFDNNEQYPPSSSSDLYYRSSALNLSTHTSNSHSPMSPTTS

QMEYSPASDPEICRRSPVGEAGSSLPHKLRHKSHLGDKDAAQSLLALQGIKAEPHDPNHE

AAEDSVGSSDERDSGISYSSSSSCSDGYPRSSDTTSSSPTNCLMGDSTSISSSRNQQSLH

LQHYQHQQEQHQKQDLDDVAEYENTQLRSELERLASEVATLKYMLVRRPRSEGDSDGSR*

>augustus-13470_pilon_pilon_pilon-processed-gene-0.6-mRNA-1-DNA_novel01

MSSPHHLQRLLTPNHHHHHSPNSDPGMPRCYLVKKVVVANDGGGNKGCTAPTSPTAGTPA

PPTPPDMADQEAQTRVYHELRAADPSFSCTFQYYQTTPDHAYANIQPQTCLSTSQQTCIS

TDQQTCLTLNQEASVPLCQQSCLPASQQNYISSSQQSTITATTPQNCYATSQQQQQQQQC

HSPRQVCHLTRQQSSSQARQQPHCLPVTQPSAVTTSQESSCHQLTHQTDHTFTPLHLRPV

EETEAAHDLLELARSAPAYSYQPPTPASSCDSVEAASTSYSETNTADDTEAIYEDSEASL

TISPTPSPAGSSDTENVAPSFPLGLDDGRSRLRTGRSEGVRIGRHKPRYTCSECGKHYAT

SSNLSRHKQTHRDLDSGNARCCHVCGKAYVSMPALAMHVLTHNLTHRCGVCGKAFSRPWL

LQGHMRSHTGEKPFRCHHCGKSFADRSNLRAHMQTHSQLKNFRCKRCNKSFALKSYLNKH

YESACYRDGACGETCQSPGP*

>maker-10090_pilon_pilon_pilon-augustus-gene-0.16-mRNA-1-DNA_novel01

MAKEFSLDEDRLLIQNVEQYQAIYNTLSPDYREQSAKDDAWKQVSGRVGRSVEDCKWRWK

NIRDTYGKRKKMSKTDRRAKKKWHLETMMRFLDSVQYGRKSKNHSSSGMSEGIIDIIDTA

ISSAYEAKPNAACPGNKDSPKNVQRATIPPTSPMDTTPPLEDAKRTRTLKKNAVKDTEME

DLAETPKEQSRQSQTTEQTATLTSNVAGTHDDHVDLFFKSVAMSVKVLPRHLIGQAKLRT

IQMLIELETESSTVSHVSNK*

>maker-10090_pilon_pilon_pilon-augustus-gene-0.16-mRNA-1-DNA_novel02

MAKEFSLDEDRLLIQNVEQYQAIYNTLSPDYREQSAKDDAWKQVSGRVGRSVEDCKWRWK

NIRDTYGKRKKMSKTDRRAKKKWHLETMMRFLDSVQYGRKSKNHSSSGMSEGIIDIIDTA

ISSAYEAKPNAACPGNKDSPKNVQRATIPPTSPMDTTPPLEDAKRTRTLKKNAVKDTEME

DLAETPKEQSRQSQTTEQTATLTSNVAGTHDDHVDLFFKSVAMSVKVLPRHLIGQAKLRT

IQMLIELETESSTVSHVSNK*

>maker-10090_pilon_pilon_pilon-augustus-gene-0.16-mRNA-1-DNA_novel03

MAKEFSLDEDRLLIQNVEQYQAIYNTLSPDYREQSAKDDAWKQVSGRVGRSVEDCKWRWK

NIRDTYGKRKKMSKTDRRAKKKWHLETMMRFLDSVQYGRKSKNHSSSGMSEGIIDIIDTA

ISSAYEAKPNAACPGNKDSPKNVQRATIPPTSPMDTTPPLEDAKRTRTLKKNAVKDTEME

DLAETPKEQSRQSQTTEQTATLTSNVAGTHDDHVDLFFKSVAMSVKVLPRHLIGQAKLRT

IQMLIELETESSTVSHVSNK*

>maker-11506_pilon_pilon_pilon-augustus-gene-0.18-mRNA-1-DNA_novel01

MADALHSQSPQDGNEARSAACGAPVQPDISPSSNILSPGDILPPTAETSSSRFLQPPTFS

FTPATPSPPLPTPHEGIEDAVIRGLLSGGVNSLNSPPLGKDASIEPDPLSLPGVLEGIQK

VDSGSNAELPDMSENSLDTFSSIVNSTSLNFREISDCLEQPGDIPVSSASMMDSRNRNET

HSKDIGNSPTVPLVDSLEKQVDGTSERMSDLMNANDEGQKAVDPPLASPPAKEFDGGGWK

HSFASGEGTNCSNGVVQDSKTNSVGHSILQPEDPVVEPVCSELVAKELPSPLSEFRAEFA

MESQPENCLGSGGSNSENTLLNLLPSEMQNSSEDSLSLPNSTVADPSKDTVANSLLVCKD

VNMTDTGEKEKYLCGGTVAADLASLTENTDLLSQIENTSNTCETEAMDVDEVNDAEKLSE

LEKTLERMDEVHDKSEVQSAKEKTLPETSLPENPLPETSLPAESSLPEFATSKDGSNKTE

CGDGKAVSGVARKVSSVLLNDSDEPLQPVTIGGSLQPLEEVQEASILNDPSFTADLSLID

SSADEADSIGGLVINSVIGAADGVADFPPDDEMEAERQLTDISSSAPRVNQESCEVDSSE

DVEPSAKRRKLENGEKEGDKMDDPRSVVFKVLPVKNGHGLWDALKLKEVLLSKGTIHLRI

HTSATEFGNFEPEKCEIEVDFTCIMVSALEEECNSPLGNAVPDPDSCDPLAIVANAGAVE

TNSPPHYPRPFGAQTRGRSTALQPAMPRPPYRHLQSPPHAYNKNEVLGLPQGIEFEEPQN

ATEWSGAGSRRVTPRVQAEPHQPSRARTHLLPQPSTSVQLDSGKKMDQVDSEKGPNRKAK

EKEKEKEVEGEEEEEEEEEEEAEEGGGGGGGGGGGGGSRVEGMCALWLGL*

>maker-14770_pilon_pilon_pilon-augustus-gene-0.11-mRNA-1-DNA_novel01

MSEYCLRWNNHRPNLVTVFSELLTSEALVDVTLATDGHYIHAHKLVLSACSVYFKDLFGA

NPCKHPIVILKDIRIDDLKTVIDFIYRGEVNVAQDRLQDVLKTAESLRIKGLAENPRNYD

EIPCQSTRFPSSGLGPQVARQRSSLTDSREQSLSLEGEEDGEPGTPPSSKRRKITSSHDS

SESQHDEQENDNTNRVLNVKDEPVDDRENDKSHSGDTEQESNVNRVADAMLMLQGASQDS

GEDSGNAAGTSADATLSSSHSQGPADWSLQQLSPWWASVLPALTRHHHHHRSPALRPPPL

HPSMAHLPSMRSMAAASGGGGGGIGSCGRMLGTASGKTVDGGGGGGGVGGNVSPRPPVTL

PLDQMSLVAVLGKEVSPQMIQALQAVAARRESFSSAAKLYAVSVTTLWRYSKKLGLTESS

HK*

>maker-15862_pilon_pilon_pilon-augustus-gene-0.23-mRNA-1-DNA_novel02

EAKGESKNNTKTVDEKSKDANKENSSKEENKENRTSSVTSGKDKSSKDDKEKSDTDSVVE

METEEKKEEDLTHLGKWEPYYRSSLGKFILDLGLNQAQEFLQSDLLRMQKKKLEKMKTVP

SKEGIVSVKVLEKQLEMTRQKNSHLRVPLKTCKFCNFKTESEMVMDRHLESPHMVNYTYK

CNFCDFETRGPQVILFHMEAEHNIRGRLERAPAFFQCSLCPYEDNNKSKMTRHSFSCAKK

YKPEKNCELADWEPPAKIPKVHRGRPNTLGKAFEPVKMPNLLPKGLAGMNINLQSAVAAN

LLTTASANTAALAAGRGRGRPVGSYKTTGQIINQGRGASPASLLYSTRGVTTGNLVQQVG

GNAQQFTLGNQMFHLVNGHLVPMSSAAASVSSVSVSSQSTGRVPRILPSSSASNVSLIPG

LAASSSITIQSVQSKAGNTKSPQQPSISITPLPRGGQQPQNVTKGTTPTRDASGKPSFVI

CEICDGYIKDLEQLRNHMNLIHKVKIHPKMIYNRPPLNCQKCQHRFFTDQGLERHLLGTH

GLVTSSMQEAANKGKDAGRCPICGKVFQWKLLNHVSRDHKMTLKPAHLSYKCTVCTATFN

MYRLFENHVYSAHSVVNKNKGDNNKKQSSGGSSDPPLKINDEITIIPQPAAKSSKPDNMT

SPGKKSGNSPSVSKEITITKVGSRPTRRSGGSSVEVISLDESPKKADKRTNKDNAGNSAK

KVKASS*

>maker-21334_pilon_pilon_pilon-augustus-gene-0.6-mRNA-1-DNA_novel01

MAPNAAMAGVARALVRYMCQDLVAASSLDSNAFQRLVWTILNLGAAHGTFREEEPLPSAR

QLLNTYLTPMAGDSRSVIARSVLDQTNLCLSLHHNTDLRLLSGAIHFITPKFELRSYALG

AHRVIEQETEDEAVTGLLSEFFSDVWVPAALRDKHVTVVSDRGTPSESGVVGVRCVWHAV

EEVLDALTEEPSYRQICDDVAAILSFLADSDVSGVSSCGLQPHEVKRWDALLHALNFITG

HHDELCAVMAGCEAGATLLGERRFYQEVSELLTGVKRCLLTVRDSLRPTLNQAVLCRAKL

LQLCAAPSASLPLRQLKQHLASKFTDALALTPFHHVASFLDPRCKSLKVLTDTEKTEVHR

HVLEMMKSVAVTEANTGVLNLKTTRTTTTTTTTSTTYSSSNGHSTDASMPATTPNDVHPF

REYMDNPAPDTSVSDSEEIMAYVNMKVHLEDDDILGWWSGTSASGLSTLRLLARKILAVP

ATCAYAHDLCLNARQARQQMGIVEDSQLNNVLHLKYNMN*

>maker-24686_pilon_pilon_pilon-augustus-gene-0.23-mRNA-1-DNA_novel01

MKRTPCVDVGVQTEAEVMRRSRRKSSSPGRWEPYGSFGSSTRNSNKRKRTTETQTRLTHR

GNKKRNTDSCDQISSKATIGGVCVEGTMEINGNTESESSFLEVSADNCSNSENSLESLTL

QIDLPELISTQSSSGTQTSPRAGGLAHTSMHNISISLFDRACGGDEPTISPPSVTTITAT

QTPQHLDPFAGSHLLIEEDEDLLDVVEEASSSMNGHPPLLAHIRSSSIETQTDHDVLLSE

NAGTPDDVDEIILTNTETQTDTNNILNMSGNTSYEPSNPCQAAGTCDVHGSDSIWCTSET

QTYEDFSDIEQFMRSTIHTQTPDHSHSELFPELSFTHTQTQTSIDDPPALVTTHTQTPTH

THFPTDLY*

>maker-25064_pilon_pilon_pilon-augustus-gene-0.33-mRNA-1-DNA_novel01

DGISHCPKPSSRRHLFLGKGGMSSPYSGESASPYSDGVPSPPTVVAAYNRPYEVVHYDGP

YIQVLKQPQSKFRFRYKSEMVGTHGQLKADCSDKNKAAFPTVKLAKWNSGPAVIRLMLYT

AEENVNQRKRHVHELSGKNCDKETGICEVVVDEKCDYTAQFQNLGIIHIAKRDTREIIMR

RKREELVAHLRLRKPQHSLEEIRRSITQADLKRIDEEADEEAKSMDLNKVTLRFQAYQYD

KNIERYRPITLPVDSDIVYNLKNATTGELKIVRMSACSAPCTGGTEIWLLVEKVRRNNVQ

VKFFELDNNDREVWTAYGEFSDSDVHHQYAIVFRTPRYRFTNLNTAVRVKVQLERPTDRD

TSEPLDFTYMPDSLKRSRIHLETALEEGKGSYNDPPSKRFNFTPYDSQAIDLSNNKCNGR

GQEDSMPTPDILEFLAGELGGNANFNIEGQSQSPLHMVPSPVNSIDSSSQGLYSPMHPGS

TGTPNTEYITLGNSQLQVPSPVHHMSTSPHYGMMSPGDQSVGSPPYQGGADSGGGMTPQF

TDSQYNSPSPGMMVPSPSYQENTMQVVNQQYIQQQQVQQHQQYIQQQQVQQHQQLQLQQQ

QQQHQQQQNQQIQKQDIGFQHLIQGTEETLLVQQQTVSMQPSQQLVQDFNLPDCFSLGNL

GQESLLDMLEVASEQLDYGANELPVSNLKADYGGKSTDTKKKEKDQPFSSGQDVDEITKL

VSGVRIDDSRERQTQRDGGNRQTTPSVDVAFKVAINAAECLQAYAATGDISLLLATHRYL

LAVQNNQGDTALHTAVSNKNVDAFNKILKACEKIKPTDLLNAQNFSLETALHQAVSGNEL

TMVRRLVAVPGCDVSIPDARGNTPLHRAVQLQDHSCLEALLTRPINGARSAVSQAINAYN

YQGETPLHLAVISGNLNIVRILISAGAQVHMCEHKRGANPLHLCAMYGRHRIAEYLIRNT

SITIEAGLFDGNTALHLAAQARDPEMCRVLMRANADPEAKNYLRRRKKVSESEEEEEEEE

SSKKDEDEEEEESEEEAECYTPLDYAGDDREILAILRREVLDEPSVSEVANEVQEVKKLP

NAQFGGDSGIDISVSGIDISVTDIPCPDSSKDSENSVGLLSAGIRRSLANHLSGDTWRHL

AQLLDLDYLVPHLSREASPASLLLQPDNMKSVTIEKLRGCLEVLGLKDCLAVLDAA*

>maker-30664_pilon_pilon_pilon-augustus-gene-0.29-mRNA-1-DNA_novel01

MAPKMEGLSSESGDDSSTGRSSSRRLVKNTEEYRKRRERNNLAVKKSRLKTKMKTQQMMD

RVTQLKTENDELEENIKILTKELSILKDMFVAHAGHAHGVKLNDADLVKMLCEDSDVDEG

VSLLMSLSQGPPL*

>maker-30664_pilon_pilon_pilon-augustus-gene-0.29-mRNA-1-DNA_novel02

MAPKMEGLSSESGDDSSTGRSSSRRLVKNTEEYRKRRERNNLAVKKSRLKTKMKTQQMMD

RVTQLKTENDELEENIKILTKELSILKDMFVAHAGHAHGVKLNDADLVKMLCEDSDVDEG

VSLLMSLSQGPPL*

>maker-4596_pilon_pilon_pilon-augustus-gene-1.72-mRNA-1-DNA_novel01

MPSALSVPVVGSLLLPAVLPLPPASPRRAGEGERGTLLLPLSPRLPPAPHLSPSSAPPPA

NPSLHGVSPYCRPPSGAGHVIKKDPYESCINLSDLLNENQTLIHQHQAPSPSSIPSSASS

SSPPYGGAPYPDHPLLRGRLEETNPFQKISSPGGSTPPPSFEQGASLHNLPPHPLEEQGQ

APFLQKLSPSGGYKESDEGLSFLESLVDVKRELEDDVSVGSSCGSPAAPSSGCGSPLEVV

TVPSPQQHQHQHQPQVSAASCQRDILETVERLAQEHNRRDVQRVSDSLNIPVDPAQWSVD

DVRAWLKLQASQLNLPPLMLDFWNYGGPSLLKLTEQQFRLLAPVGGELLYAKLEIWREAL

KSSSKFAPPSPTPSSAAPSDPRQQVGGGAFLPLSHHQQIIDATNSLMLPSSLHAHHQLHL

THQASSGGAPPSAPGAQGSGSSSSSGGSSAPSSPPLPLVPALAQVPTPVAQVSSQQGSSN

SGSSPNTPSPPLQQQGTAVYLDESCMDMAALLQQQQQQQHPPSNGSPQGGASSPHSGHHH

HTSANHCSPQEDAYDSEDEVEEESCSSSGGSGRGGTHIHLWQFLKELLQQPQLYGSCIRW

LDRQKGVFKIEDSVRVARLWGKRKNRPAMNYDKLSRSIRQYYKKGIMKKTERSQRLVYQF

CHPYCL*

>maker-4830_pilon_pilon_pilon-augustus-gene-1.16-mRNA-1-DNA_novel01

MVLQNQRLASSEILEYDEAPLDLSVSNRTRCPSPPRSPYYYRESLSPDYRDYRDRDTSSP

HDSDDSDSQRLSHPNPAKAYKKAMMKRYLDTDGARVIGGHHGEGLHHHHHHHIHHHHLSN

NGGRSREQLLQDLLSAGQLMPNMHSSNHNGNNNNNNNNGPYSSAQTYQPHPAAHAGHRTA

YSASHPNTSVGGSLPPSPADSGVSDVDSSSGHTSNDESKTRMHLTVCGSRTPDSPSSGAE

AGGPLPSFHPNYSPQQLHMRSSSQPPLTPTFPRPQDSQVMSGYGGVGVSGYMDAGSPGGM

AGYGINSPHYTTHPSGLTSPLQPPNASHMGHLVSSGMGAAPSTASPSSSSSAEDIFLGDM

GFPPRMKKKGRKPKPVDANGQPQQPGMKRKSREGSTTYLWEFLLKLLQDKECCPKYIKWT

NREKGIFKLVDSKAVSRLWGLHKNKPDMNYETMGRALRYYYQRGILAKVDGQRLVYQFVD

VPKDIIEIDCTGA*

>maker-6028_pilon_pilon_pilon-augustus-gene-0.19-mRNA-1-DNA_novel01

MYSNNYYITSGRERSSSTSTDPSSVGDDANFLRTAAAAAARKRRGNLPKESVKILKKWLY

DHRYNAYPSDDEKMQLSRAANLSVLQVCNWFINARRRILPEIIRREGHDPDKYTITRRAK

KLKGISPRDRIDQEEYPPTRQSQNDYAPRPRPRWESTDLTDHDYDFSMDSRVATWVNEHH

RVPTKPHYEAVCPCGCGSESDHTPATFPSNNTAAAASPTAPAAPAVAVVAAVPSNGPPVA

VQQTTTAPNFVTEVVAPATSTTNQNLYTDAHSPAYTPNDSPLYPPSTPEHYVPQTDYNHH

SYSTPAQVTPPPTPPEDDADKFKCLYMLVDAALSQWEKQNASPPPQDAIDTTRTYLSL*

>maker-70184_pilon_pilon_pilon-augustus-gene-0.3-mRNA-1-DNA_novel01

MTLSPRHTTPFSVTDILSPLEEQRRALDLLDDRRIDLLDSGSYGRAAAAAAMQPAGMQPV

AYPPAPPPTTYCTPDVYDARASAPSWYQTSSSDPRFAISRLMGGSNMNMNMGNVTSLSTC

SMADNKAMQFPLSQRRKRRVLFTQAQVYELERRFKQQKYLSAPEIEQLAALIHLTPTQVK

IWFQNHRYKMKRAAKEKAMSEQNQNSSSNNQSVGSPRRVAVPVLVKDGKPCPSADDQDTP

NHANSTPSTTPITSTQPPTVHHAPTQLQDQHTPTSYSATVHHSGSHVISGTLGGGGCPSP

MMSEPGWAAS*

>augustus-23758_pilon_pilon_pilon-processed-gene-1.30-mRNA-1-DNA_novel01

MSGDHKKVIKGSPSQYVKLNVGGSLHYTTIGTLTKHDNMLRAMFSGRMEVLTDSEGWILI

DRCGKHFGTILNYLRDEWIPLPENQRELQELLAEARYYCMSDLVEVCEGAIKQKEPEKDP

VCRVPLITSQREEQMLIQSSSTPVVKLHVNRHNNKYSYTSTSDDNLLKNIELFDRLSLRF

SQRVLFIKDVIGSNEICLWAYYGHGKKLAEVCCTSIVYATDRKHTKVEFPEARIYEETLN

VMLYENRTGPDAELMQATSTRGAVAPVVCTSDDEEDRRGGVCSQSGLGRLRSNKNN*

>augustus-25716_pilon_pilon_pilon-processed-gene-1.17-mRNA-1-DNA_novel01

MNCVDRNSIPRGKHLLKFVISHISGSHIQGSGDLVKAVYKPPICSYRELNLTFHEEAEEA

IVVEEGADLNELQKSVVSEYRRKTGYYNPLKISQVCVKGTSNGEENEMLKIKVVQITSNT

DEILEGILDSNLDTHCLSRFPVHPLPLCELPSDGGPTGSLKVGEREENGGILLETYSDNN

REMFSVKFGEDDIMSVSRSAVDKFHKISGITDSLSAQRERIFTYVNNKEVPITIMYLFPD

PEVKKTETCILVKETARKRGRPRGRRKGVAKGRIMKEVRELNEYVSQFKHSKFRRRDNGI

PDQDKDLQIALEEEISDMEGDDLSTFGCETGIVVSGSVVSSSADSNDGDGAAKEDLPMLE

LQSSHEDMNASLNPGDIWKFLTQYASEDNRVQGLVTQFNNESWLQVKRMSNFYQIDGEVM

TDLSFKVVYRNDFSPITYFLRGFSRLLKTGELNTANDINNLFRFMSRDRKLCLGLKPSPY

QHSIDSNTRVRKFFQEKGQFLSTPFECLLSKSCRGIVKIIGTSPVSVFTRGSRTVHGVHA

LCRACALLGKKMNQLIKLSPKNSIMEADEMQEKVVGDSFLQLLRQPVKRSGMTYSALKYL

FTQSIPEILSDKELKSSQTFPDEADIDEETQTGAKRGRGNQKEGKSKSNICVTEEAFLQM

IDLQPPNSEGLGRSLLKKCNLCQFRAPTMLSLVTHMKGHLGTLKEKCSQCEIQLSSKEAL

EIHKKQMHMERSPVCSICEMDFTTNDQLFDHMNKHRNHLPFECPYCNHKLSNKEAYKKHL

RLTHKITSVKDLKFNCKTCNIFFYTEDHVLLHRVNQNHEGIEPFNCRACAFSCVTANEFR

HHIMEHAEEEREAVNLAICPECYKVFFSKYRLTFHIEMKHSKVKAKSDQLPEAIVESESV

PTVKKKKSKGPYLRYLRANEKYECAKCKRRFKTAKTLESHIKFAHAEKKACKSEHVCTLC

GRKFNALRRLGNHMKIHSGNRPVFQCEECGQIYGKKTQVLEHIRTDHAEQVERHQQQQEL

VQVDQSQDQQFVDIQVHQQNNCSIEGSASTSWSPIKSPFKVPTVEQTQEAVYSLLQLTDF

*

>augustus-3678_pilon_pilon_pilon-processed-gene-0.3-mRNA-1-DNA_novel01

MYQESSAVDNAEYHHIMYNYETLKLPPEAAAALKMTGVEADTVTLSASDSLPTVTLSAGE

SLVPSCISTSIPVTNVLTTTSLSEGVRLTAPSLSIATPLTCTSITNGIPLTAVTLTPTNS

PSVVSAAVPSGHFSDGSVPSTLTVATMSDVDSSSVSAREDSLEIGQIVHTTDDGVLENSL

VSSSSIDAVVTSMVASADCQGVVTNGRKIPSQVIAKLLSDDSLSLDMVRIITEQDVENES

HQPQGFNMLARPGMLVHTKLADNDIVSGNSINPYRHVEVLNVRPTIRLDNEGSTHCSVVW

TCAYCSMAFPSSEAVGQHHEKECTENQSITVATSSSATVESLISPLRQVDENTFELKLST

DKESQNGTEHDAHEEPVKDEEQHWSTEHESIWTCTICGGEFNKSEELGKHHLTHSIQDLS

ASLLKLTKPQRRAKVNKITAVPLPAVTASATVPSAVVLEQDNSNNCESAWNKSGLTAMEE

ELADNPGDTPPQTPPQESLTVKVKKKTGIKAPPVEKNDKKKGGKKTNKETKAPRQRKCKG

RVVGQGSRDPHTCNICNKVLSCRGNLAKHLVTHASDKPFVCKPCGIGFNAKRDQQYHYLR

YHTNERPNICEVCGKGYVFRQYLLKHMVFHGKERQFSCEICGKQFLTAKCVMRHKKRHKK

EKLFKCTSCLKSFTVNADLKAHIRKIHRNNKKTSNIKQQQDSQHKVIQSVQSLQQKVPDS

QSLKSKDITEFLLPSNATIQGQLLPAELEPATLSGTVSSKDEVFITPDPLGSIIPLSELP

PNEYSASVAPIGDHTTYVMINSTDEEVASQNVSAAHGLNIMYAAATTPAQQVGELSRSHL

SQGNLNQGQHMLVSSTLGDTTSQLSEGTLQHIESQTDLQHQAQETQTLAAVTEAGLDEGH

QGDMNVAAGTDDHGSELAAATHDSVVHSSHDPGSGFHLELLSQTIQLELQRTQTSTANYL

GISDR*

>augustus-526_pilon_pilon_pilon-processed-gene-0.4-mRNA-1-DNA_novel01

MPRGRPRGSLGGGMAIDKPRGRMTAYAYFVQTCRSEHKKLHPEENVQFQEFSRQCSERWK

TMSDVEKKKFHELAEKDKERFESEMSSYPGGGGGRRGRRGRKAPKDPNKPKRALSAFFFY

ANDERSRVRAANPDFSVGEVAKELGRQWNELTEGEKLKYEKQAEEDRARYDREMQIYKSG

GSPAKKMKASNGHPVPEDNDLDDEEVPDDDDDDDLDNGSGDD*

>maker-17354_pilon_pilon_pilon-augustus-gene-0.34-mRNA-1-DNA_novel01

VVFSEFSRKCADRWKTMTDKEKIRFYEMAEKDKDRYESEMKGYRGPRMPRGSRKRRNRKD

PNAPKRALSAFFWFCNDERPKVRAANPDMGVGEIAKQLGAAWSNTSSEVKSKYEAMAEYD

KARYEREMKAFKEGNFAAKKQKTVDTPDEDDEEESESEEDEEEEEEVE*

>maker-19178_pilon_pilon_pilon-augustus-gene-0.10-mRNA-1-DNA_novel03

MESVKLAGIGAHAPQILYSGGRQPPPGPQAAHPPPTSRHEGSPYKTPISPPMGSRAGAGG

YPAYPAAGETHVGGGGGSRYIQQPYIYPREPHYIRPASDRPVMLPHGPRRPTILGPYGPY

AAHGRADMMPVVAGNPLAVNTTMDSSKRIKLSQPSKQDSARVPLVIDTSDNNHSSRESTY

TPQVEAISPTLPSDPRDESPLRSTKDDLLKRIEQVDREIAKAESSIAKLRKKEGELEQAA

QKGDGPTAVEEDTKEPKHQSIAQIIYAENRKKAEESHNILVKLIPPTDVKSVLPLYHQPT

DTEVYMTNKRQYAGFKRRLMENFKRRLASKNERDNNLTQTYSKLMTEWLKKVEKVENSKK

HKEKVAKNRELFEKVFPELRKQREEKERFSRVGARVKSDAEMDEILDGLQEQENEDKKMR

AYAVIPPILLEERKRKHRYTNRNGVIEDPITEYKDHKNINIWTEQEKEIFREKYLLNPKN

FVVIGSYLERKSVSDCIQFYYSTKKKENYKMLVKRRIRRRQKPNNQPVVEVFGENSTRVL

TRGNLAAIKSQQPPATTRPGSASGIVDSNSIGPSASTPATSPGPNMGLDLATSGGPATSE

SSTTSNTCNQEVREKDKENLSASENNKAVEKPSRRERIRNKDDDKTKDAQDSSDEDNAAE

HGKGGPHECLVCGTQLEHFGQSRPVGPGQAAQYGLSESHLPANPRVCTNCRCRHLRRRAQ

CPIPTCPSHKWRGKRLRPLPSRWADLPAEIKDPIIREFQIPPDVTKCCSACHNRIMRKLG

PEAEEPVDPSRWSEEDVDALRSALREVGCHWSRVAERLPNKTEGQCKSFYFNFKKKYKYD

EIVAEYRKSRGKGDGPPTVTDEEESGSSTSSCDEGGTVPPPPATQDSASDTTSAPSPGPA

IHHNNIDEKREAENTEGGMKEPLPRHPPPPASVKEDYDSSATASADEGQSCVGRDSPPEV

QHLPNPNERPPQPIPSTIGGKTPPPPVSRPSSKGTENPCAGSANVRDLIFDTIERTLEKT

DEKPQNKGMPTISNILRSSYVQRPAPVPPPPPPVGQEFHRNQMPRFNDPRDGLPHNDNNP

FDSQDGQCEGLDLTVRKRDRSPPQPPAHQSLPVHHQPAQPPQQSHPPSRPTSSPREIPTV

RGGAEVTITSKEPPQPPPRAHANTSYSKPPHEPWGFPDPRNKSPAFIPDSRALSHHQPPT

LTSVSGGPSPLAYTHVRPPHTHSSKMKMAPPPPLTSVRSQHPPPQPVNTSISTPPRIPMK

IDKQVPPTHVGSITHGTPVNPPGSIVSVPLSQAGRFDMMPKPGIGKEGGSITQGTPMHPD

QKRSVAVTSIYKDGCPPFEARAGSGVEIIPRPALPSQYEREHIYRGPRPPISSAPSSTPY

YSSYAYTRPAYPEPQISSRQIIAHDYQMSQQMINRRGVEKESRVSPRARDHSPQQDPRDH

RTTVGTTVGRIIDNSTRSIDPRGLDIRQIELSRIDSRTVDPRLDHRGGLDPRVVERINPS

TDPRFYGASGPHGSYQPPSQSSAYSQERVSRPPSSPASGRDPTPTSPNSDMAQSPNDFRS

PGVSTPTPPRVGVIQRHPTSKPPSHLAPPTSASPRTDDRYSPRHSRGGSDTFNEFVNIAV

AQPGYPSAPHESRREGRPKVPHDTPQPEGLEKQLTDSMRIRTAAMDGRLPHPDDRRYYDP

RKKLPDVDNDAPQGGFVTCYVRDRKPSPSPTGNSNNRNSSETLTAANLIDAIITHTINHT

SADSGGGPNDQRPSRRHGETGSHMEIDKRVRSPATAPVKEKEVVMVDDSPDTRPPPNPLN

HKDGVGSSLHSAPHHQGSQPKSMHAHMDQIIHREYALGDGRKMEAPPHMPYARTLPSSSS

SLEILRTDGRPYVSSGGPPAVTVSQVGNYEPWKRGAEKDGKDRIDSHSPHRPPPSSQSGG

HHIPAQHLAPDERQIVRIKQPSPVSPRPDHKRRTPPSAQATSFPVETNSPPSSSAPPLSA

DPNHTDSNNQWSQYPKRPYQQKEVFDYVKNKIAEVMRTEIQEGDKRSVDDSRHTQPGLQV

SQLQHQQQLPPQHSVSQQNAHYDMEDPLKKRARLEISDRPPSRPRSTGGDMIKERREDAP

TRDDVADSPQSGEMVIDESPRDSHPPTSKSGDMGGGKMSSKGEPSDRSGYPPVSASSPYS

YSYPGFRGHGGPGLGGPGVPPSTTSSTTSSNSSGGRPLQGYEPHVEPVSDED*

>maker-21374_pilon_pilon_pilon-augustus-gene-0.22-mRNA-1-DNA_novel01

SIASVKHGGKLHLPYKAFEPCMIATMDTGLLSLKWNNHGTTFFHVLSTIRRKESYSDVTL

ACEGKFYPVHKLVLSTCSEYFEEIFNRTQCRQPVIVLKDIRHSELEALLNYMYLGEVNVL

QADLAGLIKAAECLKIKGLAVPDEAPSPRNASRDTKRSASLRESPQQSKRLRGDDDIHKD

SREGREHARFNMSRSVPSHSSDSVSNSLASQSGLESSRDQREVHERPPDSDRLSEVRQNE

APSSSPSQPVEIVSDEPVVKTEILDEPKEEDESNDNLLNSDSSLAYDPLTSGMQGEGVSE

GGQGFDHQILTSQPQTMEELVAQAMPGTSGMQGDSLVGWGGNQAGGGGGGGGSGSDLSRF

PLDAFQVDDSQGGSQGSGGPQQQQQQPMGRGGQTTADGVFKPFMCAYCPKRFSRRDNLTL

HLRTHTGEKPYHCPLCNYRTTISSNVYRHIRNKHQLDTDTSKPLFQRQSYLHMYRGASSS

SVSHSRGENQESSTQWLDKAFKSYSPPSISCYFLIF*

>maker-24702_pilon_pilon_pilon-augustus-gene-0.36-mRNA-1-DNA_novel01

MEHNVSTAGSAVTHVTGVGSIAGVTGVTSVQGVGNVQPLNAVGNVASVDSVAGLQGVTWY

NYSPVGQLITASGPAVQVELGSPDIARQFNQQVQTQYGNIQLQVQTVPQVPITKAHAVIP

EQNIIVKSDPGVIVKQPKPRVRQSGGERVPCPECGKTVSCNATLRDHMRTHTGERPFVCG

ECGLAFAQRSNLRMHKRLHTGERPYMCGICGKTFARSSHLPAHMRTHTGERPYECHECNH

AFITAQQLKNHFRVHTGEKPWKCDLCDAAFTHSSSLSTHKKKHTGQKPHQCEKCSKAFFF

RAALDKHMKVHSRVRPFKCDNCEKAFKYKESLTVHKEKYCGKEVSKKPRAPRKPDAKKPG

PKPGSGRKYVQKRKGRPRGRPRKRGRWGKRGRPRRYIKDEEDEEEFDDEFEEKDSIIDTT

DEVLDTEQIIPEATIDPLKIEKITDDIEFEHEQKLEAQQDELQQHHHQIHADHFPHTAHE

IHIEDATALTIVSPEEAEQQAAVVDAAAAGGVQLVTLHSEVPIQIVSHDPQVQVTHQLIT

RDGVQMWYPRQYQ*

>maker-24702_pilon_pilon_pilon-augustus-gene-0.36-mRNA-1-DNA_novel02

MEQAQNTTSMVWYNYTPVDGLITTTNAPVQVELGTVDIARQINQQVSLQYGNLQAVPTQP

QQIIVSHHPQQPPAVATPEPAVTPKPPKARKAPTNGERVPCPECGKTFSCNANLRDHVRL

HTGERPFICSECGMTFAQRSNWRLHKRVHTGERPYMCGICGKTFSRSSHLPGHMRIHTGE

RPYSCELCEHSFASHQALKNHSRTHTGEKPFVCEYCQTAFTHSSSLSSHKKRCTGEKRKR

GRPKGSGRKMCKKKPSGRPRGRPRKKGRYLNRRKRKPVRTSEDDDSCKKDLPIKSELISV

DEESENQIDIVQAKEEIEDIPTVEVDPDTIQIDGADPLDTHSETENMIAHAQESISVTEG

HTIHHIVPVEEVVTVAAAAAGLDDTSSLHQEAAVAMAALHEGAALNLAHHQLPTETYVER

DTSHTMWYPKQHY*

>maker-2590_pilon_pilon_pilon-augustus-gene-2.52-mRNA-1-DNA_novel01

MSGSGKSVTGEVKVSASVKIKRNNMDDNDAAAFELMADEIKIEEENEPLYSSETIEQKDD

DEEIAAAPVNAADSAQQLRHQTDNDDPSPPDDEQMNVEVEDMDETTQEAEVLGIEKDSDV

EDPAGQTTFRIAKVYSVAKEEGEEKRDEEKQAREVKADSARVPGKKSEPKTEVQTSKTEE

NKKNAKEEKILKPKTTMATLAKVPTLTGGVGGVRLLSGGVRGTNMPLLVSMTAGSTGIPL

FPAGLPPGRYVILPGTSATTKTTCATVNTTSASMAPRLATGVSPPVAATAAVAAVVGQAT

SVAAGAGAAPAVATAMAVRSVTPTLQVPPRQHTRDRGRRKSYTTGEKLAMIEAVEAGQRK

STVADRFGVAPSTLACILLQKHKIRAEQDNLTRRRFRHVKYAGEEGGRRVGTTSRAHTSV

TPTLQSLAPSQDQPFTHFLASLTGPTPAVIDTQPEEIITVPDLMERIDGGTGSDEVSGGE

GPDGNAGEGKSQREGETEEDDAESGVECLGVVEGSGPSSMDVEYQQGSASPHDKPSSSSS

GLFARRRHPSAPQAQPDDDAVDDSFKQDISDSAPSPTSQGLTGPYLLKKEAHCTTVLDQL

LRDDTFTDVTLTAEGQSLRAHRIVLCLASSYFRQVLSREQNVQSVVLLRDVKFAELRNII

HFIYTGEATVDATDLESFLRTAEMLEITSLSEGQKYISSRGPSQVGAAAGFSIGDFERLV

GAKRPNREAQSPPRKSQKVSGESSSSRSPSAEPTAISSVPLVLMKEDPDSEPPSTSQGDR

SMNEKSSSDNVTSQESITSGIGKPGREIAKARRRRDSAESIGGFAAIAGVQKASEVDDEV

FSQTPKPECQQLADHDKSLNSEANNISLPGRCPYCPHVNQKFEGAPMMRHLLVSHPCKPA

FPCPCCWRVFVKRSHFKSHLLNCQQSY*

>maker-32102_pilon_pilon_pilon-augustus-gene-0.18-mRNA-1-DNA_novel01

MQRMDITEAAVVQPYKKKPEDPPFLTVGTEVSAKYKGAFCEAKVHKVVKVVKVKVTFKLG

LGSAVVSDDQVKGVLKIGAQVKASHPDKNQYVDAIVNKIQDCSQYTVVFDDGDITTLRRT

SLCLKSGRHFAESETLDQLPLTHPEHFGTPVGGNRRGRRSRQNPDGDDSGEEEEDNVHKR

HRSRREEWEADIGKVVCVELGDKKKMKDNWFPGLVVAPTAQDSVKIETKKDYLVRSFQDG

RYYTVPKKEASPFSREVGNKVSQSALKTAVEKALLFMTNDELPPHWDRDLLFGMDVSDED

SDRDGSDSDSSDDEPREEKDHFVAQLYKFMEECGTPINTGPTVGNKDLDLYKLFKVVQKL

GGFNRVMNQNTWKNVSTKMQLQTLGSQAPNHIKNAYKKYLQSFEDFYRKLGCTMVSNPRG

SRTRHRSGRSLIRDCDRTPRTLKKKENDSCDSTEDETEKKDDDDEKDAKKPEV

>maker-32102_pilon_pilon_pilon-augustus-gene-0.18-mRNA-1-DNA_novel02

MQPEDPPFLTVGTEVSAKYKGAFCEAKVHKVVKVVKVKVTFKLGLGSAVVSDDQVKGVLK

IGAQVKASHPDKNQYVDAIVNKIQDCSQYTVVFDDGDITTLRRTSLCLKSGRHFAESETL

DQLPLTHPEHFGTPVGGNRRGRRSRQNPDGDDSGEEEEDNVHKRHRSRREEWEADIGKVV

CVELGDKKKMKDNWFPGLVVAPTAQDSVKIETKKDYLVRSFQDGRYYTVPKKEASPFSRE

VGNKVSQSALKTAVEKALLFMTNDELPPHWDRDLLFGMDVSDEDSDRDGSDSDSSDDEPR

EEKDHFVAQLYKFMEECGTPINTGPTVGNKDLDLYKLFKVVQKLGGFNRVMNQNTWKNVS

TKMQLQTLGSQAPNHIKNAYKKYLQSFEDFYRKLGCTMVSNPRGSRTRHRSGRSLIRDCD

RTPRTLKKKENDSCDSTEDETEKKDDDDEKDAKKPEV

>maker-32102_pilon_pilon_pilon-augustus-gene-0.18-mRNA-1-DNA_novel03

MTNDELPPHWDRDLLFGMDVSDEDSDRDGSDSDSSDDEPREEKDHFVAQLYKFMEECGTP

INTGPTVGNKDLDLYKLFKVVQKLGGFNRVMNQNTWKNVSTKMQLQTLGSQAPNHIKNAY

KKYLQSFEDFYRKLGCTMVSNPRGSRTRHRSGRSLIRDCDRTPRTLKKKENDSCDSTEDE

TEKKDDDDEKDAKKPEVKKEKEETSKKEVKKEAERKEPEKKEAEKKETEKKEEKKEPEKK

KEKEDNKSTKSVKQEVNKSPEREPPKVRERSCEPLLTREKSREPAVTRERSREPVVGKDK

PREKSREPSASKDRNRESASSTKEVPSEPATPKEKREPRTPRSEAKTRAAVKVESTEVKT

RSS

>maker-33754_pilon_pilon_pilon-augustus-gene-0.38-mRNA-1-DNA_novel01

MRNLNIGIGSESSNTPPQVEESTSAARMRGVSRKRPGRARNGGWGQQRDCIEEKQGKEWK

NRQVKTECQDEQQLSKATKPVKERNGASNKQWSNVNSESKTTSEVTPISRSEGLIEQLSR

GLYECMVCCDRIKQPQAIWSCPGCYNCFHLGCIKKWAKTSAGDGGWRCPACQGFTAKVPN

TYLCFCGKVREPEWNHRETPHSCGEICGRKRKIEWCKHKCNILCHPGPCPPCSAFIRRSC

PCGTETREVRCSMTESFLCGNPCNKDLNCGGHKCPTKCHVGPCQECAEIVKQSCYCGKED

REVPCTAESFEILKYECGKECSRILECGNHKCSSNCHAGDCQRCEKAIDVVLRCPCDKVP

LEKIYERDGVIPRKSCSDPVPTCGQICSRKLKCGVPGSYHECQAMCHEGPCPPCPLSTPV

KCRCGSLDQEVSCSELTTKADEVRCKRACKKMRECGRHKCSTRCCIDIDHICTLICGRTL

TCGLHKCEDTCHRGNCRRCQETSFEELTCHCGAQVTFPPIPCGTKPPECNNPCSRPMPCG

HTPTHNCHPEDNCPPCTILVDKPCFGNHKTMKSTPCYLKALSCGSVCNKPLSCGQHNCLL

VCHEQPCVAPGSSCPQKCLKSRDSCGHPCGSACHTGPCPDVTCKEMVIITCGCGHRTSML

KCSENDREYRAMTTSLLASQMRNMNSGCSVDISEIFTATARPDKLKRLTCNEDCKTLERN

RRLAMALQIENPETRETLGNSSNLYSDFMKEEARKDPSLAKMVHDTLTELVMKAKESKQK

SRSHSFAPMNRDKRRFIHEYCEHFGCQSQAYDEEPKKNVVATAVKGTCYLPPISVLTQLQ

REQGQKKVPGPVWSRKAVTS*

>maker-3888_pilon_pilon_pilon-augustus-gene-2.10-mRNA-1-DNA_novel01

MVVLESKGMLTAGSNQYLQPDYLSPLPTTLDAKKSPLALLAQTCSQIGADSSKPLPNEKK

KEEEKKEKSTPTPIDATRRSPAFKPYESATKKNMAEETTSSEEKGHDRKTPSSQPSSARS

PSNSSNGGGSSSGTTKEESGRRSSEAGATSSASTTPIIRSGLEVLAGHPKDVPLGTYRTG

VPPMLSHGYPGLESHPALRGAHPGFMQGLGLPGLPSSLASVYPSAAAAALYSSAGLTSTT

GLPPSLLASPYLTYTRVKTAGGGEAVVPVCRDPYCTGCPYSVQNNHLLQSGGACPPSCTQ

CEQAKAALSALPGLSSLPPSSLVPASSILPPTSVAAAAAASLYSPSAASSLLTGPPRPYV

CNWIAGDTYCGKRFSTSEELLQHLRTHTNMSTDSSSLSLLSNPLHAHAALLGSAHSALHR

PGMPYPPPALSPLTAARYHPYSKPSALSTSAALTPTLPGLSPYASALSAYPHGYPSPYAG

LYPRPPL*

>maker-4390_pilon_pilon_pilon-augustus-gene-0.43-mRNA-1-DNA_novel01

MDPQWNLFQPPISEPAVLDYNGGSLHHGRYNGFYDSSNYNRNQSRSVPPISPSYTAPAQH

SVFSPHMNNQSNPQPSHQSNHHQSSHHLNQNSQQATQTPPTQGPPRSPQLTSPSSNYYTS

NSSSQYTPPVADFKIGTFTLNGFNSDTNTTTNQETNNFSHTNGVGNSQSARETGIIEKLL

QTYGFIQCCERQARLFFHFSQFDGNTEHLRIGDPVEFEVTFDRRTGKPIASAVTKISPEV

VLSEERVIGIVTTELRSESGGEHQGRITYENRGECFFLPYGKEDVEGYVTLKPGDKVSFQ

IATNQRTGNLAARHVRLENPAQPVKYQGIVNTIKDSYGFIERADVVDEIFFHFSEAKNIP

SGLKLGDDVEFYIQTRNGKEVATNMVKLNPGTVVFEDINPELIKGQVLKPVERNPRHSQK

DPLTGRIRYRGKDRSEVEVKFGERDQKGDFTLRHGDWVQFNIATDRRDHLQKATNISLLD

ESFTVSGEKREQGIIVAAKEDFGFIKCADREARMFFHYNELLDPEGDITLNDEVEFTVAQ

DNSSQSPNRQSAIRIHRLPVGTVKFEVLLKEDLQGTIITEAPASPRNSFKNGRTSPSEKV

QEPCVISYTLDGVKQNVNCYAINNCDKPPVVGDKVEFNLYQVKRSKELVAMDLRIVRGQP

NARQPTVISNNNSTPVIILPAGKMKMNASNASNCNGNANLSTNGNVVNGTSNMNGCGSGP

RSSSTLHQGFVAALKDTFGFIETIAHDKEIFFHFSNVEGDANNLELGQEVEYTLSSRTGP

GGKVSAENVRTLNKGTLPMPKVMGDIFEGTVLRSMRSINPDQQEYCGLICIGNEDEGLAQ

YEFGITGLVNKRELLQPGDQVTFQLDESRRAVLIRAIRKKLIATVDAVKGQYGFLNYETE

EGKKLFFHQSEVKDGTSLSPGDEVEFVIITNQKTGKSAACSISRIMSKNAPTQRPDRLIS

RLKNLNCDSSKPRLSVLRQPKGPDGSKGFKLQRTTSDSQNLAVS*

>maker-4390_pilon_pilon_pilon-augustus-gene-0.43-mRNA-1-DNA_novel02

MFFHYNELLDPEGDITLNDEVEFTVAQDNSSQSPNRQSAIRIHRLPVGTVKFEVLLKEDL

QGTIITEAPASPRNSFKNGRTSPSEKVQEPCVISYTLDGVKQNVNCYAINNCDKPPVVGD

KVEFNLYQVKRSKELVAMDLRIVRGQPNARQPTVISNNNSTPVIILPAGKMKMNASNASN

CNGNANLSTNGNVVNGTSNMNGCGSGPRSSSTLHQGFVAALKDTFGFIETIAHDKEIFFH

FSNVEGDANNLELGQEVEYTLSSRTGPGGKVSAENVRTLNKGTLPMPKVMGDIFEGTVLR

SMRSINPDQQEYCGLICIGNEDEGLAQYEFGITGLVNKRELLQPGDQVTFQLDESRRAVL

IRAIRKKLIATVDAVKGQYGFLNYETEEGKKLFFHQSEVKDGTSLSPGDEVEFVIITNQK

TGKSAACSISRIMSKNAPTQRPDRLISRLKNLNCDSSKPRLSVLRQPKGPDGSKGFKLQR

TTSDSQNLAVS*

>maker-4390_pilon_pilon_pilon-augustus-gene-0.43-mRNA-1-DNA_novel03

MDPQWNLFQPPISEPAVLDYNGGSLHHGRYNGFYDSSNYNRNQSRSVPPISPSYTAPAQH

SVFSPHMNNQSNPQPSHQSNHHQSSHHLNQNSQQATQTPPTQGPPRSPQLTSPSSNYYTS

NSSSQYTPPVADFKIGTFTLNGFNSDTNTTTNQETNNFSHTNGVGNSQSARETGIIEKLL

VLSEERVIGIVTTELRSESGGEHQGRITYENRGECFFLPYGKEDVEGYVTLKPGDKVSFQ

IATNQRTGNLAARHVRLENPAQPVKYQGIVNTIKDSYGFIERADVVDEIFFHFSEAKNIP

SGLKLGDDVEFYIQTRNGKEVATNMVKLNPGTVVFEDINPELIKGQVLKPVERNPRHSQK

DPLTGRIRYRGKDRSEVEVKFGERDQKGDFTLRHGDWVQFNIATDRRDHLQKATNISLLD

ESFTVSGEKREQGIIVAAKEDFGFIKCADREARMFFHYNELLDPEGDITLNDEVEFTVAQ

DNSSQSPNRQSAIRIHRLPVGTVKFEVLLKEDLQGTIITEAPASPRNSFKNGRTSPSEKV

QEPCVISYTLDGVKQNVNCYAINNCDKPPVVGDKVEFNLYQVKRSKELVAMDLRIVRGQP

NARQPTVISNNNSTPVIILPAGKMKMNASNASNCNGNANLSTNGNVVNGTSNMNGCGSGP

RSSSTLHQGFVAALKDTFGFIETIAHDKEIFFHFSNVEGDANNLELGQEVEYTLSSRTGP

GGKVSAENVRTLNKGTLPMPKVMGDIFEGTVLRSMRSINPDQQEYCGLICIGNEDEGLAQ

YEFGITGLVNKRELLQPGDQVTFQLDESRRAVLIRAIRKKLIATVDAVKGQYGFLNYETE

EGKKLFFHQSEVKDGTSLSPGDEVEFVIITNQKTGKSAACSISRIMSKNAPTQRPDRLIS

RLKNLNCDSSKPRLSVLRQPKGPDGSKGFKLQRTTSDSQNLAVS*

>maker-4390_pilon_pilon_pilon-augustus-gene-0.43-mRNA-1-DNA_novel04

MDPQWNLFQPPISEPAVLDYNGGSLHHGRYNGFYDSSNYNRNQSRSVPPISPSYTAPAQH

SVFSPHMNNQSNPQPSHQSNHHQSSHHLNQNSQQATQTPPTQGPPRSPQLTSPSSNYYTS

NSSSQYTPPVADFKIGTFTLNGFNSDTNTTTNQETNNFSHTNGVGNSQSARETGIIEKLL

QTYGFIQCCERQARLFFHFSQFDGNTEHLRIGDPVEFEVTFDRRTGKPIASAVTKISPEV

VLSEERVIGIVTTELRSESGGEHQGRITYENRGECFFLPYGKEDVEGYVTLKPGDKVSFQ

IATNQRTGNLAARHVRLENPAQPVKYQGIVNTIKDSYGFIERADVVDEIFFHFSEAKNIP

SGLKLGDDVEFYIQTRNGKEVATNMVKLNPGTVVFEDINPELIKGQVLKPVERNPRHSQK

DPLTGRIRYRGKDRSEVEVKFGERDQKGDFTLRHGDWVQFNIATDRRDHLQKATNISLLD

ESFTVSGEKREQGIIVAAKEDFGFIKCADREARMFFHYNELLDPEGDITLNDEVEFTVAQ

DNSSQSPNRQSAIRIHRLPVGTVKFEVLLKEDLQGTIITEAPASPRNSFKNGRTSPSEKV

QEPCVISYTLDGVKQNVNCYAINNCDKPPVVGDKVEFNLYQVKRSKELVAMDLRIVRGQP

NARQPTVISNNNSTPVIILPAGKMKMNASNASNCNGNANLSTNGNVVNGTSNMNGCGSGP

RSSSTLHQGFVAALKDTFGFIETIAHDKEIFFHFSNVEGDANNLELGQEVEYTLSSRTGP

GGKVSAENVRTLNKGTLPMPKVMGDIFEGTVLRSMRSINPDQQEYCGLICIGNEDEGLAQ

YEFGITGLVNKRELLQPGDQVTFQLDESRRAVLIRAIRKKLIATVDAVKGQYGFLNYETE

EGKKLFFHQSEVKDGTSLSPGDEVEFVIITNQKTGKSAACSISRIMSKNAPTQRPDRLIS

RLKNLNCDSSKPRLSVLRQPKGPDGSKGFKLQRTTSDSQNLAVS*

>maker-4390_pilon_pilon_pilon-augustus-gene-0.43-mRNA-1-DNA_novel05

VTKISPEVVLSEERVIGIVTTELRSESGGEHQGRITYENRGECFFLPYGKEDVEGYVTLK

PGDKVSFQIATNQRTGNLAARHVRLENPAQPVKYQGIVNTIKDSYGFIERADVVDEIFFH

FSEAKNIPSGLKLGDDVEFYIQTRNGKEVATNMVKLNPGTVVFEDINPELIKGQVLKPVE

RNPRHSQKDPLTGRIRYRGKDRSEVEVKFGERDQKGDFTLRHGDWVQFNIATDRRDHLQK

ATNISLLDESFTVSGEKREQGIIVAAKEDFGFIKCADREARMFFHYNELLDPEGDITLND

EVEFTVAQDNSSQSPNRQSAIRIHRLPVGTVKFEVLLKEDLQGTIITEAPASPRNSFKNG

RTSPSEKVQEPCVISYTLDGVKQNVNCYAINNCDKPPVVGDKVEFNLYQVKRSKELVAMD

LRIVRGQPNARQPTVISNNNSTPVIILPAGKMKMNASNASNCNGNANLSTNGNVVNGTSN

MNGCGSGPRSSSTLHQGFVAALKDTFGFIETIAHDKEIFFHFSNVEGDANNLELGQEVEY

TLSSRTGPGGKVSAENVRTLNKGTLPMPKVMGDIFEGTVLRSMRSINPDQQEYCGLICIG

NEDEGLAQYEFGITGLVNKRELLQPGDQVTFQLDESRRAVLIRAIRKKLIATVDAVKGQY

GFLNYETEEGKKLFFHQSEVKDGTSLSPGDEVEFVIITNQKTGKSAACSISRIMSKNAPT

QRPDRLISRLKNLNCDSSKPRLSVLRQPKGPDGSKGFKLQRTTSDSQNLAVS*

>maker-4884_pilon_pilon_pilon-augustus-gene-0.51-mRNA-1-DNA_novel01

ATPTSLHQPTGSSGAQHTMTLLPHNHNNATHPSTPTPPQPAHYNAQPHALTNLSPQNTHA

HNQNSHAHVQSPAQTPNSHAHGHNHAQASTSLEAQGPLPGPVVGEKRKLDDTGSPYSAAS

GANPAHSQPSSTGLNGFSPDYLQSSGESPAKKTDSKSKKSSDTPGVKKKKTRTTFTAYQL

EELERAFERAPYPDVFAREELAVKLNLSESRVQVWFQNRRAKWRKREPPRKNYIPPGGMG

GALGGTFNSLNSLNTLTSFNTGDAWSYSSYDPAHLNLLGPTSYTFSANHNPGYSYPMLSQ

PMINDSLFPNPIGQMRTGEFQSPTGMRDFGNGHLKTYEYLQEVKTEDFLHEKRDANMNSV

RHQLPTKENKDSSYTTLPSFLS*

>maker-4884_pilon_pilon_pilon-augustus-gene-0.51-mRNA-1-DNA_novel02

MKRARLQEEAQAYEAQISQAYTPIVAPPSQTINQPPSAMPSVAPPTPIVSQSPGNSVAPP

TPMGNQSTSVSASGSNRQLLCCEDSSYDVGSSQAYDQPVPLHKYDSPPILKISHSHHPPP

PLEKYDPSPQPSYKYEGHHSQQHDYDSATTSSGPAHDYDPLPPQVPVASDEEPLVYPNVG

VESHTGPPSMADPHMPPGYFSHYPLESQENLDSQDPLDYSVLEALFEGGSYAGQGESGNP

EAYQEYQQY*

>maker-5142_pilon_pilon_pilon-augustus-gene-1.56-mRNA-1-DNA_novel01

VFADLTVSLVTAAPGIILSLNLPESKDGESYMPTRRLEKCMKTEALSDSGWPAALSVGLY

SAWERQLFTDLVIKTQDEVIHAHKVVLAAASPYFSAMLSGGLMEGLTQTLVLEDIPGELI

RIVLIYIYTGQAPLTESNVQEVLTLSDYFQIHALRKLCCYYLANNLTAENCLGVYELAKL

HNCHDLATSSLFVATSQCSEIISHPTFLSAHPETVMKLLDQSYLSIDSEDELLKAVIEWV

SDSPSSRAEYLCKMLTKIKLDLVASDAKTRSLETLRERKLLTKEVKETIIPYDTVDANSD

EERLRNDHLEEVILVMAGESNGRVMRNIECFKMGLPSWQCSLPYVALRVENQIDSSLVLP

CMNDARAYCAVASYNNLVYIIGGQTNNAFLSSVERFNIRKNKWDKLSPLPLAIHGTAAAF

LEGCLYVVGGKTAVQHENRSWMYDMEKNMWVSREPMTQPRVHHGLAALHGSLYAIGGISP

LGANHQVHTSCEKYDPGSDSWMPIASLSQARAYLGVAVINDYLYAVGGYDGSYWLNSVER

YDPLQDQWTSVSSMISLRSSFGITVSSGRIYCIGGFSGESNLNTVEKYNPSTDTWHCVQS

MQLRRYGGVAATVLVPFLPKPNP*

>maker-6258_pilon_pilon_pilon-augustus-gene-0.38-mRNA-1-DNA_novel01

MVTCIAWGCRNRSQGLNGGGGGDVVGFHSFPVRNPALLQKWLDALQISSDALNKYCRVCS

DHFLPGDYKRDFRHELIGTRRKRLLREGAVPTVGICRRRRNDTSDVMMEESTAAAVVAPE

GLVLNDVPTVSNAASAVNCSNSGAEMDRDNDERNVHDPLSDPLAEDSVEILPEFEIKEEP

YDFYEDNITDTGSPCWISDGVTLIGENDDDIELEDGIQLEDNSDPFDYIEKLNNSHEDIM

DVSPIQKYPNKGRKWKQKPYGYTRKILKNFVPYEVDVDDDMDDGLMVTVENAAAFKDDLE

EFGWRHKRMTDADLARRRREFETHLMRCKVNIKVMQARLDEERGKMLRIQNSLMNIRRRQ

KRHEMKRMQYGGSVSEVMDDPAVGIRYSKLGSEGKNSLALNIEDGEGKKESLVAMKMRMA

RLREIKAKKALEQKRSGVVDYQKELENKLRMARLREIKAQKALERKLNLEPEPGRAKLKP

LKIPKPEECINGEDYEKVLEAKLRMARLREIKAMKALERVNNEDSDKTDEFKIKKTKVKR

LKMKIKTHKPYDELEMKAKMAKLREIKAQKALERKKLEESGEVPIKDKM

>maker-7682_pilon_pilon_pilon-augustus-gene-0.12-mRNA-1-DNA_novel01

MSDEERDIDIESDEEETCTLGPRGEIQFASQAEKRAHHNALERKRRDHIKDSFCSLRDSV

PSLQGEKASRAQILKKAADYIEFMRRKNSAHQQDIDDLKKQNNLLESQIRSLEKTKYTGN

FAAMASNNGILNNKSGSLVEFEGSGSDTSDSEVPNNVHRKKLKT*

>maker-7682_pilon_pilon_pilon-augustus-gene-0.12-mRNA-1-DNA_novel02

MSDEERDIDIESDEEETCTLGPRGEIQFASQAEKRAHHNALERKRRDHIKDSFCSLRDSV

PSLQGEKASRAQILKKAADYIEFMRRKNSAHQQDIDDLKKQNNLLESQIEMSADQRQHLP

AAALSPVRSLEKTKYTGNFAAMASNNGILNNKSGSLVEFEGSGSDTSDSEVPNNVHRKKL

KT*

>maker-8134_pilon_pilon_pilon-augustus-gene-1.12-mRNA-1-DNA_novel01

MGTQMPTKKAPTPKQHGPPVTESGAQDRISESYHLFENEDGSDVILMVGEGSYRERVPAH

TWVLAGGNQYFRALFQGPFADRHKKTYDIPNDPKGFQNLLRWLYRHECGIQSMDSALITL

QVAIEYLCPELAELCVEYIGKHLRVSNVLKVLQFTWRYCPSAQMADDSQASAPLSPAPSA

PSLEAMFGPDQGGLHQFGAADRLQQLDISQNPTHNTCQLPNPSEETEFQDPTACCSDLYN

VCLEVVDSATESVLVSEPLEELDYRTLQLILKRSTLNIKNEVLILQAVQRWSTAECKRRM

LPLTSENRRAVLGDLLYHVRFLNMTNEQLQQTSSLLTADEFNYLATRISGHTPATVPVTL

NPHLPMMATPRHHKPPTLPTTESPSKSKKKSSTGKRKYTKKELMLDVVSCLAVIFD*

>maker-8134_pilon_pilon_pilon-augustus-gene-1.12-mRNA-1-DNA_novel02

MGTQMPTKKAPTPKQHGPPVTESGAQDRISESYHLFENEDGSDVILMVGEGSYRERVPAH

TWVLAGGNQYFRALFQGPFADRHKKTYDIPNDPKGFQNLLRWLYRHECGIQSMDSALITL

QVAIEYLCPELAELCVEYIGKHLRVSNVLKVLQFTWRYCPSAQMADDSQASAPLSPAPSA

PSLEAMFGPDQGGLHQFGAADRLQQLDISQNPTHNTCQLPNPSEETEFQDPTACCSDLYN

VCLEVVDSATESVLVSEPLEELDYRTLQLILKRSTLNIKNEVLILQAVQRWSTAECKRRM

LPLTSENRRAVLGDLLYHVRFLNMTNEQLQQTSSLLTADEFNYLATRISGHTPATVPVTL

NPHLPMMATPRHHKPPTLPTTESPSKSKKKSSTGKRKYTKKELMLDVVSCLAVIFD*

>maker-886_pilon_pilon_pilon-augustus-gene-2.3-mRNA-1-DNA_novel01

MDVLPSGGIFKELQLVHDTGYFSSACSLEERWQQNCYEMDRYLKDEPRVKGGGRKLGGCE

TDAPWSAYYEEYTHDGSSSPVRLFTKLGRPIKGARASRFLVKVKSETLSAELNIKREPFD

PDTCFKDEPLDDSISVKEEPLEDLDDPLSQDDLCGSERSLDSVSVSSSSSSASSLASLEG

DLDTSVSAGGQSPTRGRSLISWASSEDMVTDTRLEAGGRRGSGSPFYAATKLLTAKSAPN

IATLTPPSSPETSPVTHTHTLLKVHARSQVPKFISFATSLPLKSSNNSSSLSMGVRGAMS

VGVHHPRTADTSPDAKRRVHKCLFPGCKKVYTKSSHLKAHQRTHTGEKPYRCSWEGCEWR

FARSDELTRHFRKHTGAKPFKCNHCGRCFSRSDHLALHMKRHQ*

>maker-9550_pilon_pilon_pilon-augustus-gene-2.45-mRNA-1-DNA_novel01

MTALNSLFSFTSPAVKKLLGWKQGDEEEKWAEKAVDSLVKKLKKKKGAIEDLERALSCPG

QQSKCVTIPRSLDGRLQVSHRKGLPHVIYCRVWRWPDLQSHHELKPFEHCQYPFSAKQKE

VCVNPYHYKRVESPVLPPVLVPRHSEFAPGHSLLSFQQVPEPMPSSVSYSQNEPMPNNVS

YSQNGFVHSQMGGASHSQMSGGNIAGINQPPSPVGSVVSPASQSPHNSYGSIPGGGSLTG

LNQPPSPGISPASQAPQSPYGSIPADTPPPAYSPSEDIKQTQPAEPMDTVGTNVTPITYQ

EPKYWSSIAYYELNNRVGEVFHAQTHSVIVDGFTDPSNNSERFCLGLLSNVNRNSTIENT

RRHIGKGVHLYYVGGEVYAECLSDSAIFVQSRNCNHHHGFHPSTVCKIPPGCSLKIFNNQ

EFAQLLSQSVNHGYEAVFDLTKMCTIRMSFVKGWGAEYHRQDVTSTPCWIEIHLHGPLQW

LDKVLTQMGAPADRISSVS*

>maker-9826_pilon_pilon_pilon-augustus-gene-0.15-mRNA-1-DNA_novel01

MSLELLLDAAKFLEQQEESCKDEETTEDAVSEESDKREVVYRRTLETYSRTAPITASPTT

TTTQHHYHRHHHHHNSNHGSVLATSPPPPPPPPARTTPTSPAHAVTPPVVGVRASPGTLG

VNAVQSHQQQTSPRTSLSYPGSPASSPPRVVLPPRGSSSPLGLLSALSQDSSSPPMGGTS

SDPGLSRLPHESLHSHLNNGIRHNGITKSLSLSPPSHNFTHHGVYSPEDNSFKEDKKRSG

STPSREVHNKLEKNRRAHLKECFETLRRQLPSMDDKKISNQTILKAAHRHIQNLKRKERE

YEHEMERLAREKIAHQQKLSALKKELSARWDHIDFNTLLPDMASLEQHRDKETKSTTTAS

EQPDLDDDAPRLDASNGQVSSAAVVASCPLSPRLSTNQPQGTSFTTIASSGNSIGATGLA

TLREHFTDSNQPLNLSTSIVDTAKVSPSGQTLVTQSVSLHQNGEWGTRQASPHYTQAQYH

ASTNKEEETWSATVVTSVTPTATGDTATTVHSEQHLPIVGSTGGINLSAQLVAGGMQLNG

APGGLLGQPAIQVITPEGYKLLPADHAPNGAKPLTITLAHPAEGLERADSADEVKSSPGA

MMLALNKNGVSALHLAVPTPPRSLPHSQGTNGTVTTVPLNLSVGGTATVKVTSAATSLIP

SYIPITHANGITQLVPGLGTSVGTLVSGIGTPLVAPLVVSQPQRPVVPGSATTAGNKGLK

ATAVAGSTNSMPLVTAQYASSLAGGLVKPVVVVSAPTAVAGMVTGAPAGASGKP*

>maker-9826_pilon_pilon_pilon-augustus-gene-0.15-mRNA-1-DNA_novel02

MGGTSSDPGLSRLPHESLHSHLNNGIRHNGITKSLSLSPPSHNFTHHGVYSPEDNSFKED

KKRSGSTPSREVHNKLEKNRRAHLKECFETLRRQLPSMDDKKISNQTILKAAHRHIQNLK

RKEREYEHEMERLAREKIAHQQKLSALKKELSARWDHIDFNTLLPDMASLEQHRDKETKS

TTTASEQPDLDDDAPRLDASNGQVSSAAVVASCPLSPRLSTNQPQGTSFTTIASSGNSIG

ATGLATLREHFTDSNQPLNLSTSIVDTAKVSPSGQTLVTQSVSLHQNGEWGTRQASPHYT

QAQYHASTNKEEETWSATVVTSVTPTATGDTATTVHSEQHLPIVGSTGGINLSAQLVAGG

MQLNGAPGGLLGQPAIQVITPEGYKLLPADHAPNGAKPLTITLAHPAEGLERADSADEVK

SSPGAMMLALNKNGVSALHLAVPTPPRSLPHSQGTNGTVTTVPLNLSVGGTATVKVTSAA

TSLIPSYIPITHANGITQLVPGLGTSVGTLVSGIGTPLVAPLVVSQPQRPVVPGSATTAG

NKGLKATAVAGSTNSMPLVTAQYASSLAGGLVKPVVVVSAPTAVAGMVTGAPAGASGKP*

>maker-9826_pilon_pilon_pilon-augustus-gene-0.15-mRNA-1-DNA_novel03

MVKMGLKELDSSEKRWSHPPKKKWIYDYLADDEETTEDAVSEESDKREVVYRRTLETYSR

TAPITASPTTTTTQHHYHRHHHHHNSNHGSVLATSPPPPPPPPARTTPTSPAHAVTPPVV

GVRASPGTLGVNAVQSHQQQTSPRTSLSYPGSPASSPPRVVLPPRGSSSPLGLLSALSQD

SSSPPMGGTSSDPGLSRLPHESLHSHLNNGIRHNGITKSLSLSPPSHNFTHHGVYSPEDN

SFKEDKKRSGSTPSREVHNKLEKNRRAHLKECFETLRRQLPSMDDKKISNQTILKAAHRH

IQNLKRKEREYEHEMERLAREKIAHQQKLSALKKELSARWDHIDFNTLLPDMASLEQHRD

KETKSTTTASEQPDLDDDAPRLDASNGQVSSAAVVASCPLSPRLSTNQPQGTSFTTIASS

GNSIGATGLATLREHFTDSNQPLNLSTSIVDTAKVSPSGQTLVTQSVSLHQNGEWGTRQA

SPHYTQAQYHASTNKEEETWSATVVTSVTPTATGDTATTVHSEQHLPIVGSTGGINLSAQ

LVAGGMQLNGAPGGLLGQPAIQVITPEGYKLLPADHAPNGAKPLTITLAHPAEGLERADS

ADEVKSSPGAMMLALNKNGVSALHLAVPTPPRSLPHSQGTNGTVTTVPLNLSVGGTATVK

VTSAATSLIPSYIPITHANGITQLVPGLGTSVGTLVSGIGTPLVAPLVVSQPQRPVVPGS

ATTAGNKGLKATAVAGSTNSMPLVTAQYASSLAGGLVKPVVVVSAPTAVAGMVTGAPAGA

SGKP*

>augustus-18946_pilon_pilon_pilon-processed-gene-0.8-mRNA-1-DNA_novel01

MCSLKSHMKTHMGEKPYTCSLCQRSFSQLGLLKKHMRTHTGEKPYSCSICQRSFAESGYL

RRHMRTHTGEKPYTCSICQTSFAESGYLRRHMITHTGEKKFTCSVCLRGFSQSCDLKRHM

RTHTGEKPFTCSICQRSFSQSCDLKRHMRTHTGEKPYTCSICQGSFSYQNTFKIHMRTHT

GEKPYNCSVCQRSFSISGTLTKHMRTHAGE*

>augustus-9322_pilon_pilon_pilon-processed-gene-0.6-mRNA-1-DNA_novel01

MRIHTKERPFDCELCSKSFTQSTTLKNHLKTHGNDKISGAVYDSEDSANDQSSSKEKSKK

HRCELCGQYFALKNTLKIHMMRHTGERPHKCDVCPKSFTQSSTLKIHMRTHTGDKPYACS

MCDARFAYNYVLQKHIIKHKEAGEMGEEEHSEQEEYEEYSEQEDSEDNVKLRTDDRKLVK

QSYNSAVKHEESNRNVNSKKIDNGETNITFNGGEDQEKNGKKITPMDFLAVFQSSLKASS

N*

>maker-10886_pilon_pilon_pilon-augustus-gene-1.12-mRNA-1-DNA_novel01

MDMYMRRKCQECRLKKCLNVGMRPECVVPESQCQVKREQKKAREKDKKDYPSAGSPLVED

NKHSPLSPCKPKGPSSASVPSYKSNNGTGSFSITPMDWPQEKESSEEEGRDSKLPLSRGG

GTGAKPLTHEQQELIHTLVYYQEEFEQPSEADMKKIKFTFDGEDTSDLRFKHITEMTILT

VQLIVEFSKQLPGFDTLQREDQITLLKACSSEVMMLRAARRYDASTDSIVFGNNYPYTLD

SYESAGLGESAGAIFQFCRNLCKMKVDNAEYALLAAIAIFSERPSLKEPHKVEKLQEIYL

EALKAHVENRRTPRSSVVFAKLLNILTDLRTLGNLNSEMCFSLTLKNKKLPPFLAEIWDI

HSCDQY*

>maker-13752_pilon_pilon_pilon-augustus-gene-0.1-mRNA-1-DNA_novel01

SHLWLAVAKGGCPSQRPSSSLDFSLPFCLSPAANMLTASSPSSGYFPKEYLRPYEYRPSS

YHKSKSHSLKRHRHGIMTVTLLQCEGVKMSLFQDLKLKRRKVDSRCSSDGESLAESSSCS

PDSGSSGGETSSCSPVQPQATYLPPESPRASPHLHPHIERSSPDSAGNEFGEVKSSRDYE

QEEDPRDAHVFDGGGTQPVSSSGVSVMSHSSGMQDAHPSDEVDPPERMSTTPPPPDTSEH

LPEISSVSTGTPLPPPLVSLAPPLNGLGAMPATPYTLAVSMAGLAPSIASSAMGLRCGAP

VSVAPPYWVGGGGSGGGGSVAGPGASRVNGVRPELISGGIVQSSPAAPPPAAAPADSSRA

GYSPRLQRTPTVIMGEAGGVKTMFWTGPSESITPVSQAPREVISCPSTPCVYGADTSDAV

RASVDALMSLGQDRRGSPPQLSPSSTLSLSPSARSPITPSSRSPHTNPPISRSPYTVAPS

SPGYQVVRGLGSPQSSPGDLHLLQNSSYVHSPTSVETQRHPSAPLNMERLWAGDRSQLPQ

TQPDSQAALNLTTVGMWGARANGMDTGVGVPAQPTQTEEDEDDQPMICMICEDRATGLHY

GIITCEGCKGFFKRTVQNKRVYTCVADGDCEITKAQRNRCQYCRFQKCLRQGMVLAAVRE

DRMPGGRNSGAVYNLYKVKYKKKNKKNGQMKPGAPASEKKLSLDPLMTSGTTSLANSPIP

MPISSALTPPVTSPITSGLNSGHILKAALTNPSEVAHFRQRLDNNVTSTRERLLPYPVAE

AMIRLLIECDDFEDIATLKNLDDLLDHKSDLSTKLRQLGDSISIKLVQWTKRLPFYHELP

VEVHTRLLTHKWHELLVLTTSAYQAIYGLQKLGSRSSDGTEAEFHQEVSNNLCTLQTCLN

SMMGRPITMDQLRQEVGVMVEKITHVTLALRKAKIQIEEYVCLKVITMLNQVARSGHSEL

EVIQERYMNCLRTYCETHYPFQPNRYQDLLVRLPDIQAAAAKLLETKMLYVPFLLNSTIN

R*

>maker-18582_pilon_pilon_pilon-augustus-gene-0.35-mRNA-1

MATAKTIVITLPKNMSGTPMVLPAPSPATTAIAGKQLTNILTGNYVSVIKEEVPDMEGNN

ADGKPPRKRQRLDHLTMEEKIMRRKLKNRVAAQTARDRKKLRMDQLEAQLAELTEHVNEL

TDLTSLLTEQNAELAEKNEALQTMLSKCVCGLNSSQVESNHSISETQKADVFEGVMVPVS

TQIVGSAESSPLQRAISVLAVLRIMVLSSLCQHWVNTAVLSLSLAHSSSIPTNQLQRMPS

KHTPQNKWWGPHQQSWNPVGT*

>maker-22642_pilon_pilon_pilon-augustus-gene-2.9-mRNA-1-DNA_novel01

MAENTISKIVEDLLIADSEKEAVSEHNSGLLFAHMYVKKELHRRMELLDSKRPKKKRTSA

KKGGEKDDPVSESEEKGQSPSSPQTRDNSKEYSPLVIPGTVKEELSETEEFSDTEDVSTR

GSSPPARKRTAEEIDIEIKEEEIDDFEFFPCDVSPKDHDEEDDDDTDEEEDEDYELKDDE

ALVIDEDYAPTASSEESLMPQLLQHLLASPSSALSVDEVKSREFLASRLLMNIAQGATLS

EVSRLAANEASPLDLRLTSKSGDVAFIPIAPKIDILKLQQNAKAAALLLDHPCKSPLQQN

TESDALLLDSPCKSPLSGSPPLKESSSSVMTDQELVSLTVRELNRRICRLPKALQNDLKR

RRRTLKNRTYAQNSRTKRTQYQQHLKTIIKHLRKENSETREEVNRLSQQIILYKSENTLL

KDDILNLKIQLRTTLNELERVKQQSSRCHKCKSACPDAVLEKDQPESASEIPTELSSEDT

TED*

>maker-6178_pilon_pilon_pilon-augustus-gene-0.45-mRNA-1-DNA_novel01

MEEPLPCALPASSISQEEDRDLFTKPAPILAGFSCERCGTNFSENHNFLAFLQSSEQRFS

CETCGKEFDRKEMQGIIIGKTTGEQAYACEICYMTFKGKGNLNRHMRKHTGERPYECEMC

DKRFSQKGHLVKHTRCHTNERPYVCNLCGKSFIASDYLSTHIKEHAGVRPFDCEICGRGF

TQKGNLNKHKKRHFGERSYSCEICGNSFTQKEHLNKHMRRHTQERPFECEFCGKTFADKG

YLGIHQRQHTGERPYSCEICGRAFARQETLNVHLRQHTGERPYKCDVCGRRFTENGTMKR

HMMRKHTIGKQFTCEVCGKIYPEKGILKLHMRKHTGERPFSCEICSKTFTENSILTRHMK

KHTGERPYSCPICGKNFTRKGVVDKHMKLHGSDEPKKSTEISTVSLELNPEMVKEEVKDV

DIDICIKEEDISDETILSEAPSSS*

>maker-992_pilon_pilon_pilon-augustus-gene-2.19-mRNA-1-DNA_novel01

DHITRLQDFVSAMSKLQPDEHEYAYLKTIVLFQCEHVSGSKRNQIERLQERAAQELRHHV

EDAYAETPERFTKLLLKLPPLRALHPQVMEELFFAGLIGNVQIDSVIPYIMKMEMTE*

>augustus-10212_pilon_pilon_pilon-processed-gene-0.12-mRNA-1-DNA_novel01

MAAIELFRHCKQGHLEEVRNLVEQRDVDVNIRDKWDSTPLYYACLCGHLQVVEYLIGVGA

RCEANTFDGERCLYGALTDEIRRVLTQHSLVTTHTIRRDAYDEFLRRVFESGEYSDVTFV

IQGQSFLLHRCLLSARSEYFKDMFLTRWHHKDKVTINRDLVDPASFSAVMKYIYTGRFEC

PQELVERCIMIGVNCKLPNFKSMLDDALKKAVSLQMGKHGLVKVTTVVVEPDTCIGAGSE

GVGSDLRELTQATLPKHLRFWPTEMPFCPTQDQANFADVCFLVEGHQFMCHKMFFCPRSE

YFKALLRDHFKEAEWKDSSSDTRIPVVTLHNISAEVFAVLVHYLYCNQSMVNLENAMDVI

VASDMLLVPGLKRQCGVYLGTQLHTDNVITVLRLSRMFELPRLEDQCIAFMAKNLEQVLE

EEDFRDLILADAEEVVGRQETDTVTVVDELRYYISSAVETISEIHEARTKLAALEAVLED

LGIDA*

>augustus-13364_pilon_pilon_pilon-processed-gene-2.2-mRNA-1-DNA_novel01

MECVLCEELFPSKVKLVQHFRETHSVAKHRPRRSKVVEGIRQITLKEFDESEGEDSEPNP

VNPFTCSDCGDQFATQVSFEDHICDNHDSSMQQIKFAVSCIYCGQKCKNSRGATIHQVFC

EQRINGFGFKRIHHECPADDCGAIFKNQHQLIKHIKNHHAGESVKVISSPILSRTTHDGE

NKVPAVQLVQQQMTNSPMVTVTMKPHIGKTDGLQDLSLLITPTKGMEESTANDTSENTSS

QFMSKNLQESASITVEECWTMEGEVRGEGETVLCVAEDGSVSHTPGNNINDIIDHEKTFL

LITDSNILNDKCMRSYECRICCELFLEKGSLNEHMDKKHRSIRSTATAFIKEEPEDDFED

CAKRKDEDHEWEFVSFQKNKDDMDVSICKGKFEYVCKLCNRGFLSELSYQGHIKNEECRT

KKFKLPRMFSCHFKRCDSSFFKLTELQKHWQIAHKFAMASENHEFEDEKAFFNWVSCEEA

KHKVRFTCDVRRRKPHRSERLMVCHRFHSLRTAAARKAAKREYSDKHNWVHKIQPCLCYA

RMKVYQDFNKDTCDYTGKIRVVYYYEHSHPESRVTEEEENILEHILQRNKRNRNGSFQEL

KGSEKVQHRQKLEKFARIAGREYRASVRNSKTSVVRRSRKGVVNGGILNTSSVSGVHVNG

NSILESDEFFDSPVEMVLDGQFDMSHEVLAGPAAANLDTLAIFDGSKVKQDCDETSENDV

AVSQEDSEDLESEDSTIVLPASADDWGKLFHFARSRVEKEDDSALKAEASLVLPYEKVIG

LISPSEQIPIFQQLWELAYPALNSEEVIQVVKINVVR*

>augustus-1854_pilon_pilon_pilon-processed-gene-0.3-mRNA-1-DNA_novel01

MSQITGDPIIKTLDGPSDGFHLLTYLEDFNDHSLMDRLGIGVPVDGDAPVQTMAENNSKL

LDLNASQYATLQAGTPRPDRANNAAQTGYYTIDIDELPGCQGNAEVSDPLAIDSAGSFAM

DSYDETFVQSNATDKTTRIVLHTSDNIAGESNDPLGLNTSSSTEQKIILAQEKPNQYFII

TGDHCQVPINLLETHTTLPLVSLVSPSPVVNTIALEAAPGNDGVPYSDNQPNTFGTVTPP

GKSSQPKPRASRRQRPKKPKKYEMTQELHDPAEEKKRLNAINAKRNRDRKKDKLKELGEK

VEAVTKERDALLKEVEELKRRERQLRELLLSKHNIML*

>augustus-30786_pilon_pilon_pilon-processed-gene-0.3-mRNA-1-DNA_novel01

MFKCTYCNKAYTAKSSLNVHMLSHSGKKLYACMFCDKRFSRKNYLPIHMRVHTGERPYKC

TVCGKTFRQNANLRKHEKLHLPGPFSIHIGEWPYKCSLCQKTFRNLGSLTRHNKLHDEIK

CLSCSICSKQFSEKSLFENHFQQHSCSKSKGYSQIGNGTTREKIHSGHQQYHCANCEKSF

SQKQDFEKHCRTHLAVPAYNCQLCNKGFTRLKSLHKHAQKIHSNQISFGCGFCHRNFTDT

GTFTNHMKYYFVHGYCEDDHIKVNSDNGKTQDIEQVMFPKANIFRVEDDLIVIVISENDV

NEGEMCHALKHVKSKFGLLTAKKISSNEKSCISDNEVSGLLTEKQLHIISNIPNLKGKIS

GPFCKKTWKTFNKLPNANGKHKFGCDVCDAKL

>augustus-5578_pilon_pilon_pilon-processed-gene-0.4-mRNA-1-DNA_novel01

EDLKTYSSPDLLICGNCRELFSNLSDFIDHRRTYCKLRFTCKCSSHNGTARMEESALLMC

ALCKENFASAWDLMVHAQAAHMVNIYQLASKDTDKDNGSCNAAGDNTAGSTEGSDVGMDS

SADGGEASGDGADAPHNDDEPQPKQNGVGGLDDGEGGHGLSGAEEIEGNSTDIMNGASPP

TLPTDVVVPSSLKTEAVVTQ*

>maker-27068_pilon_pilon_pilon-augustus-gene-0.4-mRNA-1-DNA_novel01

MTVAWRSAGDMATEVGGMGGMGGGGEHPGELVKTGAPNILCTALPTHWRSNKSLPMSFKV

VALDDVKDGTIVTIRAGNDENFCGELRNNSAVMKNQVAKFNDLRFVGRSGRGKSFNLSII

ISTAPVQMTTVTKAIKVTVDGPREPRNKSREWIVGHPGWGFPLGYHPWLDSHLSALGYNL

GLTAFNPLAHTKALELARVGGNGWLDAWRGASGLVGGLGMGVGGSPLPGATLSGLSAHSL

PTLPTHNPPQSHALASITNSEIPRSLLSDTRCLRCRGCESGGPCSSILHHSSPVSSSPSA

IASSSSSSSSSSTSSSTTTSTSSITNSSSGGGSSSSSSGGSRPPPLAPIDGLSAFSIPRS

LGTAKTTFPSRDSSFQPVKPSSERSPETKVWRPY*

>maker-30146_pilon_pilon_pilon-augustus-gene-1.10-mRNA-1-DNA_novel01

MYECASGGVPPVRSGESTTKPFDDEHHRRINRNTTTELGAVAYQEHSQYDALDLNMNSGF

SLYPQYGSDDFNQDSPRYTSPKPGGAYGDAYFDGGVDVWGATGGGAGGAAAAGAGAVGGS

YPYPSPSMMGPSSHLSQPHYIPQHLQDPIGYDHMPSGLPPMSTFRGPGASSTTGGGSVAT

SSPLYTHSPVPNHTQATPTANTGDALGKALSSIYPGEQTSSSYSSNPGTPVSATSPPPLT

SGASSGAPQQWPQPHTPTSPHYDRSLPMTSRIDERLEDAVNVLQRHAETSGGLPAGPHLG

PPTHGGSLPPYPAGLDSHLAGPPNSTFTTLGSHSQDLKALGVPGLGDGVKDKLDVKEELS

THLPTTTVASSIATGTKSGKRSRSSCSSTEEDECADPETKALREKERRQANNARERIRIR

DINDALKELGRMCMTHLKTDKPQTKLGILNMAVDVIMSLEQQVRERNLNPKAACLKRREE

EKSDGSKLPPPHLPHPSSMAPPFPNLPGDHLSHGGAGPH*

>maker-5726_pilon_pilon_pilon-augustus-gene-0.9-mRNA-1-DNA_novel01

MFNMDFHHYETTNGMYSGRGGMYTPTMQDVSATPTNMWPQQVDYGMQDLGSMKSPSTPPL

SFPPRVYTTAQVPQRQSCPIPANAAIPSLNQLQGSSMPLSRSTLGYSGYEMATGSWAAAA

DAYGTYAPQVRNAISAVKGAYETVTSEASLYNCRERRECANCAASRTPLWRRDSSTGYYL

CNACALYTRTNGINRPLGKAPTRRLSGSRRAGQQCSNCHTTVTSLWRRNPQGEAVCNACG

LYYKLHNVNRPLTMKKESIQTRKRKPKKNGDSKSASSTGSSTSSSSTSVSSSAVPSTTPS

SSASSNVLGGGVSSMTSTVATSHSGASLSSSKYNHQCSSPTTSVKNELSGAHYATMYGSL

SSSAYSSHAPPSSSSSSVSLGSSLPPLLSSYTIPGLSKSSPSTLHYGSVKDEPLSPIANA

MGELSALSHAHNVTHAHSASHASSSEAGSSVASPTGLSSSAVSPREPLSGTTPSSSSSSS

SSSSSAGQMIPTPSQMSPVPLYAPASMLHPHIVNSGSLSCVSLGGTPPAEHAALQQTHSP

PMTPTTHQQHLEHLSWKAK*

>maker-58340_pilon_pilon_pilon-augustus-gene-0.3-mRNA-1-DNA_novel01

MLAGSLTPPDKLNGEGHQGPPHNHHHHPHALMPVMPTLHTPPSPLPTPSPPVYDRYAPKD

QRLTREAMQRYLRERGDQILVILHAKVAQKSYGNEKRFFCPPPCIYLYGDGWQRKRHELQ

RAGASEQDSQLCAFIGIGNSDQDMQQLDLNGKHYCAAKTLFISDSDKRKHFMLTVKKFWG

SGADIGVFHSRRIKVISKPSKKKQSLKNADLCIASGTKVALFNRLRSQTVSTRYLHVENG

NFHASSTQWGAFTIHLLDDNESESEEFNVRDGYIHYGSTVKLVCAVTGMALPRLIIRKVD

KQTALLDADDPVSQLHKCAFYMKDTERMYLCLSQERIIQFQATPCPKEPNKEMINDGASW

TIISTDKAEYQFYEGMGPVRAPVTPVPIVNSLHLNGGGDVAMLELTGESFSPNLRVWFGD

VEAETMYRCQESMLCVVPDISAFRRGWQWVRQPTQVPVSLVRHDGIIYATNLTFTYTPEP

GPRQHCPQADDIMRPPAHRLPPAALQEVPTSAHHHHASPPANPLIPLQFEPSQPIHTSTQ

QQQQQQQQQQQQQQQQQQQQQQQQQQQQHL*

>maker-58340_pilon_pilon_pilon-augustus-gene-0.3-mRNA-1-DNA_novel02

MLAGSLTPPDKLNGEGHQGPPHNHHHHPHALMPVMPTLHTPPSPLPTPSPPVYDRYAPKD

QRLTREAMQRYLRERGDQILVILHAKVAQKSYGNEKRFFCPPPCIYLYGDGWQRKRHELQ

RAGASEQDSQLCAFIGIGNSDQDMQQLDLNGKHYCAAKTLFISDSDKRKHFMLTVKKFWG

SGADIGVFHSRRIKVISKPSKKKQSLKNADLCIASGTKVALFNRLRSQTVSTRYLHVENG

NFHASSTQWGAFTIHLLDDNESESEEFNVRDGYIHYGSTVKLVCAVTGMALPRLIIRKVD

KQTALLDADDPVSQLHKCAFYMKDTERMYLCLSQERIIQFQATPCPKEPNKEMINDGASW

TIISTDKAEYQFYEGMGPVRAPVTPVPIVNSLHLNGGGDVAMLELTGESFSPNLRVWFGD

VEAETMYRCQESMLCVVPDISAFRRGWQWVRQPTQVPVSLVRHDGIIYATNLTFTYTPEP

GPRQHCPQADDIMRPPAHRLPPAALQEVPTSAHHHHASPPANPLIPLQFEPSQPIHTSTQ

QQQQQQQQQQQQQQQQQQQQQQQQQQQQHL*

>maker-58340_pilon_pilon_pilon-augustus-gene-0.3-mRNA-1-DNA_novel03

MLAGSLTPPDKLNGEGHQGPPHNHHHHPHALMPVMPTLHTPPSPLPTPSPPVYDRYAPKD

QRLTREAMQRYLRERGDQILVILHAKVAQKSYGNEKRFFCPPPCIYLYGDGWQRKRHELQ

RAGASEQDSQLCAFIGIGNSDQDMQQLDLNGKHYCAAKTLFISDSDKRKHFMLTVKKFWG

SGADIGVFHSRRIKVISKPSKKKQSLKNADLCIASGTKVALFNRLRSQTVSTRYLHVENG

NFHASSTQWGAFTIHLLDDNESESEEFNVRDGYIHYGSTVKLVCAVTGMALPRLIIRKVD

KQTALLDADDPVSQLHKCAFYMKDTERMYLCLSQERIIQFQATPCPKEPNKEMINDGASW

TIISTDKAEYQFYEGMGPVRAPVTPVPIVNSLHLNGGGDVAMLELTGESFSPNLRVWFGD

VEAETMYRCQESMLCVVPDISAFRRGWQWVRQPTQVPVSLVRHDGIIYATNLTFTYTPEP

GPRQHCPQADDIMRPPAHRLPPAALQEVPTSAHHHHASPPANPLIPLQFEPSQPIHTSTQ

QQQQQQQQQQQQQQQQQQQQQQQQQQQQHL*

>maker-66710_pilon_pilon_pilon-augustus-gene-0.0-mRNA-1-DNA_novel01

MITSEGSSLPIVHIRVVYEQSCKVYPCDYCFKTFTSEKSLSSHICAHHKSVDVQDVFVCS

ICNCCLLSQSDLLEHAALRHQLQLLGSDASFQLCQYCGAQLPNHETLGKHIEKDHAGAFL

KKGCSSSDIIELTLSEISNIGMGDKNCRTLFKVIEIPLEEKENSYCEDPKSLVKGTDDRE

AYKVKSSGETEVSRVRQDIVVDQKCSKIEGPLNKDFRTKVNMKKDAVLQFDCSKQELWNE

ASVRSEGEEHENSKLAITVLCEPDESAFPSAKGELVLTVESEEKLVWSKDVVSKVISSSG

ILRKSSLNDKMLDLLPVSVSREEAEVVTQLYSGTDQIDGNKRLSPANSNVETHIVVGCVD

GMENCIAEDKGTDCQKVIKTRDCCICGKRFTNMGELERHEGSVHGVRIKCDLCNDTFAFS

KSLKTHYLRKHSNSKSFPCEFCGSCFKSRGNLWSHRLSHHGAKRHECPQCPQRFCTKSKL

NNHLQTHSGKKKYQCGECNKGFIYPGLLVHHMGKHFSEERWDDAPENETFYLVEVKTEDA

DAPSTSDNQLQ*

>maker-9268_pilon_pilon_pilon-augustus-gene-1.14-mRNA-1-DNA_novel01

MESGARSLLPDRMEGPKPHFPIRLDDSATSMLPDRGKSPLTLAIDATTTQLNSVAESFKT

HFPHGTDGTKSFLLQNLEGTMALLAARGVDAPYPIALDGLRPFLPYGSSGESVKSYLLER

LDKPHKSVRDRKDIKCSGSDRTTTTTSSSSVATSVGGDIKRFHHHHHHDRSREHKSSNSH

SIKKVPLPKSQLSNPRHDANLTHAIRELHDEEFGLDAVLLAEGGHVGAHCAVLAAASPFL

KTILLHANDHPAIISVTGTSFSTLQSLVTCLYTGNIPSGASLFHLIEAAKLLKMDELAAV

LQKTYFAQGGSLDLPPKSQQLISSSQRSTTTTTASLQQGLITSQEQQPEKFGKKVRSNRL

DQILYQKLNVNHLAPSDIPEQPPPHPPPSLVPPSQESSRVPPLFDASRVPILEAPRVPPP

DHGGILGELLTKADPLRSIFSNPSIYSGIPDLSFEASRASTLKPLNLSKSSKAETLTSSA

ASTVSSSTCTTTAILSSTTTSCSPSSTMASSLASQTDLTLAGLTSLAPTLPGNMPGLLTP

LTPSNISSMISPSALVSLSTTSALASFGAQHSAITSKPKSSCGNGTSRSRSTTKSICSGR

SSSSSSTNTTRPGDNSGTPSHSLPLLYTLRSGMMTPEMAKEVYEQLKINPQLSTLAGFNI

NSIPNLASLSSSASLQNLANLHNLSSLQSFPTIFPFLASATENSQINTVESSGSVVHDNK

VITLSPTEPPSNDYNVTVNSTSVNANNLSSSVMTAEDGVLNLSSSAQTVRSESPGVTTSV

AVVAATSASTEEATIQTSDSVDQPTVVNVSPSETKEEQTDSSPPEVTNVSTGCQTVGLID

PPENCGTVVVASSDLNCTLSNGCSVSEETKASILSGQLDPSNLPLLLMTSNGPLTSISQT

QTNIVENIETTPDVQAVLSKDPSLMSSEGADTGDDTSSIEVVDEIPGISQALQNRKAMFH

AGHVSNLKMGRKRKGCGDCEGCQVVEDCGQCRFCRDKAKFGGPNRLKQVCVYKRCVHAEL

EPEDSKKKRKSSGKKGRGKCGGCDGCQRTTDCNECYACLHNAATQPPARRKVCEMRVCEQ

QQMEEVRATLSVAGEPSPYSTDSLMAEGIISSGTSSPTPDGQPSTHNDKMKLMRKMLKKK

FAQPYSRVSPSKVRTKYYCGECPGCQTTTPCGNCLYCEDMPKFGGPGRYRQKCVKQLCVY

HPRLQALKLSNRSKITYDEQHILPETLNHLGGNVVQSDTRNQL*

>augustus-1990_pilon_pilon_pilon-processed-gene-0.11-mRNA-1-DNA_novel01

MKNLARKTRQCIPSYWVSSQFKISLRKTKPELSLIMEMKMGLVNPSMMNRFMRSLISRGK

LDQNLKRKAGTSEVSNEEEAVSEVSNEEAVPKVILEDHARFTCEQESDGVIGAAIDKQAQ

NVHQDQNVVASEQENETYNQPLPDDTPQAETGRENEWVTCENPFECPVQETKFDLQRKVA

LGNANDKWTYELPNRQPVQGVTHGGQDKVTPKYDNVASSELANKQLQSGVFEDKSVMLPV

QRYEVENHGLYNKHPAQDTSLNQGKVLDHQNKQMSSEFPDRQIVQESFPEIQRKMAFSHG

NERVQNEFSNKQPGQDAIFEYQRRIALGHGSNYQQSAEEVAQGVERTAAPTGYMFVKENS

GVNIDSNRMTGSDYHRGHAFQCKVCKLTFYQEHQYNKHMETYHKEGNDIAHKATSDNLTN

KSYGKLYNLTDGMACFPPGTSLGKPAYGDFPVKGDHFSFNTMVYKCKYCNVVFGNCEAFQ

WHLFHAHSKNSFYECYLCRTKYTDRGQFTNHIQSHTQREMHTCSFCKEGLHICEIGFYSE

AYANKLRDGCVYNEQNYNLLYNHELRYPAERAGNVGNQHPNSSVVCNVVGETESSYNQKQ

YNYGRYNLENNAHENYANSNMSQIHKGSLKASEDLRQLNMFSPETSAQRIHENVINVMFE

EFVKVEENCFYSGQENFGYERRLFSVVNHNFADNSHNPCGSGNIPVWTDNEMGNEEFTQV

FDILYNTGQGSGGVIGKNDSEFNIPLKKKRKTTKSDRNGEGKKRGRGQSSKKVPQSTEGG

GGPNPLESGDPSNENTTISYNARLYECNVCHRKFQHDCNLHLHMKVHESNPDEKEED

>augustus-2330_pilon_pilon_pilon-processed-gene-1.2-mRNA-1-DNA_novel01

MLANQLKTTREYWEEEKEKMSLARNEMDIENGSQVTLLTPLTTANSYTTLSEPLSPVRGP

PPQQATHSVIVPNSAVLHQQGGQQICTISIPSGNLLDPILGHSGGGDRAAPPPHDEDKAG

LNQTSSGLACTICGDRATGKHYGAHSCDGCKGFFRRSVRKNHSYSCRFNRGCTVDKDKRN

QCRYCRLRKCIRAGMKKEAVQNERDRISTRRPSYDESALNSGISLSAILNAENYSRQFTP

AFGEISVMNKKVAGVMDVCDSMKQQLLVLVDWAKYIPAFCDLVLDDQVALLRAHAGEHLI

LGAAYRSMNLTDMILLGNDCVILRNSSVELEITRIGHRIMDELILPLRQANMDGHEYACL

KTIVFFDPVIRGLHNVEKVKSLRHRVQLLLEDYINDRQYEPRGRFGEMLLTLPALQSITW

QMIEQIQFAKLFGGAKIDNLLQEMLLGGTGNDGPNGMSGNNASSGGYGGGDGGIGIGVPA

PPPGGTGLPIPLGAATAASGGMALCLSSSDINRGTYGGGLGLEGVTLGGTCDDGGDRDGD

EAKTFLGSASPISSHCLSPYSGSEGSNSHMSVIMRDVTKEANIQLTPLKNFKQENSDSG*

>augustus-2330_pilon_pilon_pilon-processed-gene-1.2-mRNA-1-DNA_novel02

MLANQLKTTREYWEEEKEKMSLARNEMDIENGSQVTLLTPLTTANSYTTLSEPLSPVRGP

PPQQATHSVIVPNSAVLHQQGGQQICTISIPSGNLLDPILGHSGGGDRAAPPPHDEDKAG

LNQTSSGLACTICGDRATGKHYGAHSCDGCKGFFRRSVRKNHSYSCRFNRGCTVDKDKRN

QCRYCRLRKCIRAGMKKEAVQNERDRISTRRPSYDESALNSGISLSAILNAENYSRQFTP

AFGEISVMNKKVAGVMDVCDSMKQQLLVLVDWAKYIPAFCDLVLDDQVALLRAHAGEHLI

LGAAYRSMNLTDMILLGNDCVILRNSSVELEITRIGHRIMDELILPLRQANMDGHEYACL

KTIVFFDPVIRGLHNVEKVKSLRHRVQLLLEDYINDRQYEPRGRFGEMLLTLPALQSITW

QMIEQIQFAKLFGGAKIDNLLQEMLLGGTGNDGPNGMSGNNASSGGYGGGDGGIGIGVPA

PPPGGTGLPIPLGAATAASGGMALCLSSSDINRGTYGGGLGLEGVTLGGTCDDDGDEAKT

FLGSASPISSHCLSPYSGSEGSNSHMSVIMRDVTKEANIQLTPLKNFKQENSDSG*

>augustus-2330_pilon_pilon_pilon-processed-gene-1.2-mRNA-1-DNA_novel03

MLANQLKTTREYWEEEKEKMSLARNEMDIENGSQVTLLTPLTTANSYTTLSEPLSPVRGP

PPQQATHSVIVPNSAVLHQQGGQQICTISIPSGNLLDPILGHSGGGDRAAPPPHDEDKAG

LNQTSSGLACTICGDRATGKHYGAHSCDGCKGFFRRSVRKNHSYSCRFNRGCTVDKDKRN

QCRYCRLRKCIRAGMKKEAVQNERDRISTRRPSYDESALNSGISLSAILNAENYSRQFTP

AFGEISVMNKKVAGVMDVCDSMKQQLLVLVDWAKYIPAFCDLVLDDQVALLRAHAGEHLI

LGAAYRSMNLTDMILLGNDCVILRNSSVELEITRIGHRIMDELILPLRQANMDGHEYACL

KTIVFFDPVIRGLHNVEKVKSLRHRVQLLLEDYINDRQYEPRGRFGEMLLTLPALQSITW

QMIEQIQFAKLFGGAKIDNLLQEMLLGGTGNDGPNGMSGNNASSGGYGGGDGGIGIGVPA

PPPGGTGLPIPLGAATAASGGMALCLSSSDINRGTYGGGLGLEGVTLGGTCDDGGDRDGD

EAKTFLGSASPISSHCLSPYSGSEGSNSHMSVIMRDVTKEANIQLTPLKNFKQENSDSG*

>augustus-5412_pilon_pilon_pilon-processed-gene-0.6-mRNA-1-DNA_novel01

MTSNSNARTSQIVYTSVDDDMTVTEIICEYDCDDMVTEAVCEVEPPESSDGQIYLAEPSH

NDNMPVIKEEKIYIDSESFQHEQNFKDDGIYIDDNSDDPVHQIFAEDKKFKCGVCCEQFD

DLAAISRHMEEHSGEKKYECAECGKQFKNINNVMRHMVVHSGERKYMCPICKKPFTQVGN

LKRHMFVHSGEKKFPCSTCGKGFTQMNNLIRHQAIHSGVKNFECNICHRLFNQASNLNRH

LLIHKGIKLYSCDTCGRSFSQLTNLQKHRERHLRGKKYKCEVCGDGFDHKLMYNKHLENH

YGKPRFQCNVCGRVVTQKSTLSQHMLLHFENKKYTCHQCNKCYAQKGSLAKHMEAHNLTK

YFCKICNRYFYDEGGLLKHESTHTDKAKSIASMVQKEIRVLDYNLQFCEFCGKSFILKRS

LRRHMEKTHNVLLEIVRKNCRENPCEECGKIFVHRKSLKKHMLRKHNAEIEPDAERSSPV

IKTEKEDDMSDEENEADESSLAILDKDKITETLLQIKQENVIEADTTFEESEKVWNDISN

CKVKEEPEERTSNAMVHLCEICGRGYYLVSNLHRHMEIKHGVTGKGKENVNGKFSSSASV

KMVNNFILKETGITVKKLTGDSRSFVNQVASSDDELLEDQTVARGVGVRRRGRNVSTGIE

KKGIAKVAEDPENKPKGKRGRKRKFSFFSDADICDICGRGFLTPFDLLLHMLEHSKAKKN

ELLSIVNEAAQEFQSMKKSEVELNVTEKTKGLVSHFRKCSRNQSNEISSIFSIGETYEQV

PFSGDVSPIRHCAVSLSDCEQESITTECVDFGAESKQEVLEINEFDYV*

>augustus-8670_pilon_pilon_pilon-processed-gene-1.24-mRNA-1-DNA_novel01

MGDSVIEEAMIASSLMEDAIIDGTYDDYADMSSFSANLCDVVQEDMGMYDAPDEQDMYNS

KTKRLICKRCGKAFRNKILLKKHTAMHTKDNVFDCEECDKKFSRKESLTRHMLVHSGMKF

YECFECGRMFNQNGHLRTHMLTHTKEKNFQCDSCNMKFARKDNLKRHYLLHSRKKSFECS

ICAKRFNQNGHLESHMLTHTQEKNFECEQCFMKFARRDTLKRHMLVHTREKAFECDSCSA

KFVQNSHLKAHLLVHTREKKFNCPDCDKSFARKGSLKRHMCVHTGERNFECTECGKTFVQ

KTHLNKHQSIHSGTRDKMNDENVDEFLENINKVEVHILSPSKVGKNGKPVKRRNRRCLKN

SQAGKGGGSKKGTSNSATNNSVNTQAGEASKADPSLSADGTACKVKDESAAVSKPGSGGK

PASGDQNASKNQSASGAQPVSGSGSASGNQPVRAQQGTMTNIQQQNSLQPVGVNNVHCPP

GDVRSPPVNIESENPDGSQMQPNNLLANSNSVMALALLDHFLTGNESQENVAAAEESGGK

GKGNNQWGQKTQQELLAAQTLSAIGQRASVVNENHQGYPQAPCINPQMPSCNMQPVPQGV

LASGPAAAMKIRPPAGAQWIPQDKALVHPQNIVYNVPPPAHEIFRYYSPANMPLHHPSNR

YPDGSCIPADMRATPRKPKYPQKVLLSPGEWQQMPMHPGGMNATMPMYAHTKHPSDPPRM

LDRP*

>maker-1110_pilon_pilon_pilon-augustus-gene-0.31-mRNA-1-DNA_novel01

MEVAPNTFDGSASQLLLLTFDKTKDHTTRVRTKKLNFRCDICQKPFSKNAYLARHMVTHT

GEKNFECHICGKKFSQNPHLRTHMKIHTSEKSWICSVCGKGFFQKSNLTTHMVTHTGERN

HRCEVCGKGFSQKTNLNTHMVSHTGGYEFICDYCEKGFSRFSHLKRHLISHTGEKNFRCD

LCGKNYTMNSSLERHKLTLHNNEWVENQRKRKAARKPMKCKGSKENDLNDLLVKRVENVQ

ESTHNSLSITIKTEAETFVIETDPSLFHDDDDDISNLDIEHNDHENLHIKTETDEEQIIA

NVCSNGESCEQRISKNGGSEKECGVDHINESIHKRRVF*

>maker-11302_pilon_pilon_pilon-augustus-gene-0.4-mRNA-1-DNA_novel01

RARARVGNIKERGCTQITTHFPSFTIMADPENTVTSPEEPKAPAKEKEVLATKVTGTVKW

FNVKSGYGFINRHDTKEDVFVHQSAITKNNPRKYVRSVGDGEDVEFDVVVGEKGNEAANV

TGPGGEAVKGSPYAADRRRGYRRRYYRRAAPGEEGEGEGEGVMAPPMRGRGRGGFRGRGP

RRFYRGRFFGRGRGRGGRGGYDQAGYMNYGEEVIDGDGMSMRGRGRGRGRGRGRGGRGPR

GYFRRYYAGSRPQPEGMMGGPSEGSRRRFRRGGRGRGRGRIGSGPETKGDGQGQEGQSAG

SGQAQQAVENTTAESSHKLSHPPLQSSTKANEAHYLGYPDKVT*

>maker-11302_pilon_pilon_pilon-augustus-gene-0.4-mRNA-1-DNA_novel02

RARARVGNIKERGCTQITTHFPSFTIMADPENTVTSPEEPKAPAKEKEVLATKVTGTVKW

FNVKSGYGFINRHDTKEDVFVHQSAITKNNPRKYVRSVGDGEDVEFDVVVGEKGNEAANV

TGPGGEAVKGSPYAADRRRGYRRRYYRRAAPGEEGEGEGEGVMAPPMRGRGRGGFRGRGP

RRFYRGRFFGRGRGRGGRGGYDQAGYMNYGEEVIDGDGMSMRGRGRGRGRGRGRGGRGPR

GYFRRYYAGSRPQPEGMMGGPSEGSRRRFRRGGRGRGRGRIGSGPETKTSETQTQVAAAE

GQKAS*

>maker-11302_pilon_pilon_pilon-augustus-gene-0.4-mRNA-1-DNA_novel03

AGRARARVGNIKERGCTQITTHFPSFTIMADPENTVTSPEEPKAPAKEKEVLATKVTGTV

KWFNVKSGYGFINRHDTKEDVFVHQSAITKNNPRKYVRSVGDGEDVEFDVVVGEKGNEAA

NVTGPGGEAVKGSPYAADRRRGYRRRYYRRAAPGEEGEGEGEGVMAPPMRGRGRGGFRGR

GPRRFYRGRFFGRGRGRGGRGGYDQAGYMNYGEEVIDG*

>maker-11302_pilon_pilon_pilon-augustus-gene-0.4-mRNA-1-DNA_novel04

GRARARVGNIKERGCTQITTHFPSFTIMADPENTVTSPEEPKAPAKEKEVLATKVTGTVK

WFNVKSGYGFINRHDTKEDVFVHQSAITKNNPRKYVRSVGDGEDVEFDVVVGEKGNEAAN

VTGPGGEAVKGSPYAADRRRGYRRRYYRRAAPGEEGEGEGEGVMAPPMRGRGRGGFRGRG

PRRFYRGRFFGRGRGRGGRGGYDQAGYMNYGEEVIDGDGMSMRGRGRGRGRGRGRGGRGP

RGYFRRYYAGSRPQPEGMMGGPSEGSRRRFRRGGRGRGRGRIGSGPETKVSEDYSKHFGE

VN*

>maker-11302_pilon_pilon_pilon-augustus-gene-0.4-mRNA-1-DNA_novel05

RARARVGNIKERGCTQITTHFPSFTIMADPENTVTSPEEPKAPAKEKEVLATKVTGTVKW

FNVKSGYGFINRHDTKEDVFVHQSAITKNNPRKYVRSVGDGEDVEFDVVVGEKGNEAANV

TGPGGEAVKGSPYAADRRRGYRRRYYRRAAPGEEGEGEGEGVMAPPMRGRGRGGFRGRGP

RRFYRGRFFGRGRGRGGRGGYDQAGYMNYGEEVIDGDGMSMRGRGRGRGRGRGRGGRGPR

GYFRRYYAGSRPQPEGMMGGPSEGSRRRFRRGGRGRGRGRIGSGPETKTSETQTQVAAAE

GQKGDGQGQEGQSAGSGQAQQAVENTTAESSHKLSHPPLQSSTKANEAHYLGYPDKVT*

>maker-11302_pilon_pilon_pilon-augustus-gene-0.4-mRNA-1-DNA_novel07

WFNVKSGYGFINRHDTKEDVFVHQSAITKNNPRKYVRSVGDGEDVEFDVVVGEKGNEAAN

VTGPGGEAVKGSPYAADRRRGYRRRYYRRAAPGEEGEGEGEGVMAPPMRGRGRGGFRGRG

PRRFYRGRFFGRGRGRGGRGGYDQAGYMNYGEEVIDGDGMSMRGRGRGRGRGRGRGGRGP

RGYFRRYYAGSRPQPEGMMGGPSEGSRRRFRRGGRGRGRGRIGSGPETKTSETQTQVAAA

EGQKGDGQGQEGQSAGSGQAQQAVENTTAESSHKLSHPPLQSSTKANEAHYLGYPDKVT*

>maker-11302_pilon_pilon_pilon-augustus-gene-0.4-mRNA-1-DNA_novel08

KSGYGFINRHDTKEDVFVHQSAITKNNPRKYVRSVGDGEDVEFDVVVGEKGNEAANVTGP

GGEAVKGSPYAADRRRGYRRRYYRRAAPGEEGEGEGEGVMAPPMRGRGRGGFRGRGPRRF

YRGRFFGRGRGRGGRGGYDQAGYMNYGEEVIDGDGMSMRGRGRGRGRGRGRGGRGPRGYF

RRYYAGSRPQPEGMMGGPSEGSRRRFRRGGRGRGRGRIGSGPETKGDGQGQEGQSAGSGQ

AQQAVENTTAESSHKLSHPPLQSSTKANEAHYLGYPDKVT*

>maker-12620_pilon_pilon_pilon-augustus-gene-0.9-mRNA-1-DNA_novel01

MWDLRLSFAEKHFPQSLQAKGFSPVWITTCLLRVDLSENDFPQSLHTKGLSPVWITMWVL

RYDLRENDFPQSLHTKGLSPVWITMWVLRFDLLENDFQQSLHTKGFSPVWTTPCGSPDAI

YVRTTSHSHCRRRLSPPAPPWIAMLVLMGNDPHFMGERLPTDPCRRRARPSCGSPCVS*

>maker-14298_pilon_pilon_pilon-augustus-gene-0.4-mRNA-1-DNA_novel01

MTETIKAERGGRVSVCIGCGGVINDQYILRVAPDMEWHAACLKCVDCHQFLDEFCTCFVR

DGKTYCKRDYVRLFGAKCDKCGRMFEKTDLVMRARTKIYHLDCFRCSACGKQLLPGDEFA

LREDGLYCRHDFDIFEKKANAENNNTTNINNNELKVSGEVKSVRKERREGKTTRVRTVLN

EKQLHTLRTCYAANCRPDALMKEQLVEMTGLSPRVIRVWFQNKRCKDKKRSILMKQMQQE

KVCEEKQMALQGITGVPMVASSPIRHDGAPMSIGMGGPLDVTAYQPPWKSLTEFALTHDL

ERLDPTTPQYQQLVQQMHGYGAEMCSATELLGSQPPPQQPPHPQQSQQHQQHPHSDSKYD

PGPFDDSVHSDSYVSYLESDDSMTAPLSTTTSP*

>maker-20952_pilon_pilon_pilon-augustus-gene-0.10-mRNA-1-DNA_novel01

MECGGYESEGKGKRKQHCTYCKNHGKKSRKTNHKCDHEHCVCLLCKLTRLSRLIMRHQQR

LWRHLKDSKRRQNAAAADATGAGAGGGAGEPAGGEHTTDSSGAANGTSSTKLQKCDMCRN

HGEIMSKRAHKNACPYQDCLCELCSLTRKRRYIMRLQQRVRRSQVTSKQHNEVWEYVTKA

TAELESLGLQDIQQTTDDPNASSSSSDASSSPSPDSSSSSTSGGSLSLKPEVAPPNPVTP

AAPAEIRSKDTASSPVPMDMPPLEPLPECSPTRYSPRPPAPPLTNVPPPAATSRTKTSCY

PSPPPPNPLPPNRVVTNVPQMEIEPAWLNLKREREEFLKQNSRVQTREENLFGHLDFMKD

HHHHHHHRTMTGRLEPPVPPEASRSFHSTAIPMNFGTSPIPPYKSLVPMIDTSHHPPPPL

QKSRADCNLHFQYRSMLWGNLPAEPSVGFVPEEFQQHCYRSFLASNTDLRIQPPPGLIQI

PQPQHPLRPRPLPPVRPHTLPVSVECDHVNLHYLAYFLQNQSRDTLSTLSTSRSFGLPRE

ALFLHHPVP*

>maker-2194_pilon_pilon_pilon-augustus-gene-0.27-mRNA-1-DNA_novel01

MAAEGSALPKTEQQKENLKEGFTERKVDGSLNLEPSTEVKEEPEFIGCDDCDVDYSCQFV

KSEGNCQNGEEGFVSCDKKSGKKIIGEGNVQLHMVVTDDSDSLSCENIFCEKFPYEANEN

NCKNVEMFSANTEMEGEPGNNIEYQSIVPPSVEVGIPLRKRKINIPVTCSVCQKICSNKG

ILKVHMRTHTGEKPYVCSECPSKFSHSDSLNRHMRTHTGEEKPYVCSVCPKRYSYLNSLK

IHLKTHTGEKDFTCPKCQKSFAIKNTLRKHMTTHTSEKPYTCSECQKSFAMKCILTRHMR

IHSGENLQRRGKLFVCTECSGTYSCVDSLRRHMRIHSGEKPFTCTSVNVVSINRVSSSSI

*

>maker-2194_pilon_pilon_pilon-augustus-gene-0.27-mRNA-1-DNA_novel02

MAAEGSALPKTEQQKENLKEGFTERKVDGSLNLEPSTEVKEEPEFIGCDDCDVDYSCQFV

KSEGNCQNGEEGFVSCDKKSGKKIIGEGNVQLHMVVTDDSDSLSCENIFCEKFPYEANEN

NCKNVEMFSANTEMEGEPGNNIEYQSIVPPSVEVGIPLRKRKINIPVTCSVCQKICSNKG

ILKVHMRTHTGEKPYVCSECPSKFSHSDSLNRHMRTHTGEEKPYVCSVCPKRYSYLNSLK

IHLKTHTGEKDFTCPKCQKSFAIKNTLRKHMTTHTSEKPYTCSECQKSFAMKCILTRHMR

IHSGENLQRRGKLFVCTECSGTYSCVDSLRRHMRIHSGEKPFTCTSVNVVSINRVSSSSI

*

>maker-22838_pilon_pilon_pilon-augustus-gene-0.13-mRNA-1-DNA_novel01

MEPVYCEQEYYDIPSDSQVLVDKTVIEFDGTTVLCRVCGDKASGFHYGVHSCEGCKGFFR

RSIQQKIQYRPCTKNQQCSILRINRNRCQYCRLKKCIAVGMSRDAVRFGRVPKREKAKIL

AAMQSVNARSQEKAVLAELEDDTRVIAAIIRAHMDTCEFTQDKVAPMLQQARAHPSYTQC

PPTLACPLNPRPVPLHGQQEMVQDFSERFSPAIRGVVEFAKRLPGFQQLPQEDQVTLLKA

GVFEVLLVRLAGMFDARTNTMLCLNGQLLRREALHTSVNARFLMDSMFDFAERVNCLSLS

DAELALFCAVVILAPDRPGLRNSELVERVQRRLVNCLQAVISKHHPERPNLHQELLCKIP

DLRTLNTLHSEKLLKYKMTEHTAAANGAWDYRSWNSEQESSIGSPSSSCAADEAMQSPVS

CSESVIGESGSSSESLCGSEVSGYSELRQPFPLARRRHDLSEGASSGDEATESPLKCPFI

KRKSESPDDSGIESGTDRSDKLSSPSVCSSPRSSIDEKSEEDQEDMPVLRRALQAPPIIN

TDLLIESVYKPHKKFRALRREDEPHSSQPTPSILAQTLSQPLQQTPPPPPPPVASSQSTV

SSSNSTLAATLASSLESTLSTDETKGDPTQSRLVNKHSTLARTLLRGTNKTTEDHMRRAD

YLHTMIMRHEPPREQLTPVSNSSSVSPAPYHVPQAALDRLQPPVSSWPYASSRSCPSSSS

SNSCVSTLSPMQPTVTAQPRLHLLTTPTPSRYCEPRVSVTPVGLGAQPTSSPIQIPSSQS

VTVTPVGMGMGVQPQRASLSPVVELQVDIADSQPLNLSKKTPPPTPQEFTMEA*

>maker-22838_pilon_pilon_pilon-augustus-gene-0.13-mRNA-1-DNA_novel02

MKMPEEDLPILKGLLQYGVAPHYMSVRFGRVPKREKAKILAAMQSVNARSQEKAVLAELE

DDTRVIAAIIRAHMDTCEFTQDKVAPMLQQARAHPSYTQCPPTLACPLNPRPVPLHGQQE

MVQDFSERFSPAIRGVVEFAKRLPGFQQLPQEDQVTLLKAGVFEVLLVRLAGMFDARTNT

MLCLNGQLLRREALHTSVNARFLMDSMFDFAERVNCLSLSDAELALFCAVVILAPDRPGL

RNSELVERVQRRLVNCLQAVISKHHPERPNLHQELLCKIPDLRTLNTLHSEKLLKYKMTE

HTAAANGAWDYRSWNSEQESSIGSPSSSCAADEAMQSPVSCSESVIGESGSSSESLCGSE

VSGYSELRQPFPLARRRHDLSEGASSGDEATESPLKCPFIKRKSESPDDSGIESGTDRSD

KLSSPSVCSSPRSSIDEKSEEDQEDMPVLRRALQAPPIINTDLLIESVYKPHKKFRALRR

EDEPHSSQPTPSILAQTLSQPLQQTPPPPPPPVASSQSTVSSSNSTLAATLASSLESTLS

TDETKGDPTQSRLVNKHSTLARTLLRGTNKTTEDHMRRADYLHTMIMRHEPPREQLTPVS

NSSSVSPAPYHVPQAALDRLQPPVSSWPYASSRSCPSSSSSNSCVSTLSPMQPTVTAQPR

LHLLTTPTPSRYCEPRVSVTPVGLGAQPTSSPIQIPSSQSVTVTPVGMGMGVQPQRASLS

PVVELQVDIADSQPLNLSKKTPPPTPQEFTMEA*

>maker-24312_pilon_pilon_pilon-augustus-gene-0.8-mRNA-1-DNA_novel01

MSAGETNSLIQEAFNDGIILYSKQTLNYECVLCKKSMTGDAPLAQHLESKKHEEAAFRYA

VSHVSSCPESLKKFFTPEIKRAVEEGIIEVSQSPGGVYGIYCKTCNKDVNGYAPLTQHLM

GADHRKQLQGVQVRIPSFVTGASNNRLEEARSVTSPLLAPGSEGFVKFPPRLTSTCTSAI

SSTQTTPIPTLTFRNDQRHVPNGASLLEPSYPRLPEPVGLYRSQPSHRETSPTRFPTPRS

PLQSVSLSMSAINSFPKRSESREVTPSSPLPRHSLPLINPGALHLDPQNSIVLQAINDDV

LVFNSETLKYTCRVCGIFFPTQQQTEDHLKHKIHREASARIVVNQQGHGPPSLLELLPQN

IIEAAAASTITVTEGNTFKCVVCGTSSTGVIPLKQHLESREHLKKKGISSGQPALPHLSN

AYARSGTESSSLREPPGNSHTADDVNVNALAVAFERGLVFCLDNNSSVFKCETCGVTLNS

LRNLDQHLNGAKHKKQFQKNSLTPGGQPCLTSPQSSEDPNTPRGFQKNSLTPGGQPCLTS

PQSSEYPNTPRGFQKNSLTPGGQPCLTSPQSSEDPNTPRGCNVQRQPYEVNTEPRGLVYI

FNYKFTGRKTNPKERKGAEQDTVNLMKTFTDMGYKVIPMENMTATETRDSMEEIRQSRDL

ERVAALIFFFLSHGNEPYKFYGEDGEMLDLQQDVWNKLTNTSCPSMKNKPKIIFANFCRG

FDTEMNEYDAVVRDVPRDLVTIHAATEGITARRNPQLGTHFVQSLCDALRWCAHDSDLRG

IYEVLDTISKSRGGTQPKWEDSRFKTFYFNPVW*

>maker-25660_pilon_pilon_pilon-augustus-gene-0.29-mRNA-1-DNA_novel01

MEERIQNIYQCAVCLKEFSRKSTCKSHMKLHTGERPFECETCGMTFASKSNLTNHVLTHT

HQKCTTCGKTFTSKPLLKSHMIMHSSERPHECNLCGRCFKRKGELRSHMVTHTGEKRWKC

EVCGNLFTERHSLKTHMRLHTGEKPYKCSVCEKTFSRKSSYNSHSRMHTGVQPYKCFVCG

KKFAHRSVMVCHSRKHMIRECIMCGMNFMKENELTSHVREQHQDDWPHRCNICGLLFTNN

AKVRKHMRLHTEEKPFRCNVCSRQFLHKSALQSHMLSHSESEGRPTCNICGKSYTAKLSL

EIHMRTHTGEKPFACSVCGKKFTRRSGCISHMRLHTGEKPYDCHICGKRFAYSSGIRSHM

KLHSRAECNLCGVKLIGGIDPKNHVTHHIVAVGAKCGLCDIEFKSLNVFKSHLYKHTVAE

REEECRKNAETMGIALGQTDNKDDIDGNHTTQPPELPVLVEEDAVDKKVNIPEVAMLEAF

PDQEDLLLSPEIKENERKGNMSTVMQSVVKVKQENIEMEDPEFFDTAVLEVTKCSESQAG

ITTVRVTTKQEKNNSDDDVDKACHDFDTHPQDINFDTAAPVSPLRDIDSLQSSLSPSSLS

NPPSPLPKLTEQVASINSSNIKESITVNDRDFFTGSSGSPGKVITISTSSPSKTPFVCVG

STNEKSLIAKASSYVVTLQKSSSANSSLKANMKTTPTEVISPKNVYLKEIKSNRSVLSVI

KVTAKGKKQEVPSTKSPQGDDVKPRLASATKKLFDSEDMNLANSYSAYVKKMLNLINPPE

KQMALIKALNHTMDDFIEKYKSWK*

>maker-27444_pilon_pilon_pilon-augustus-gene-1.23-mRNA-1

MILQECEMARRFDAEDSEGSNDAMSPSKAKDGMVNNITLWQFLLELLLSNQYKHIITWTN

SEGEFKLLNAEEVARLWGLRKNKTNMNYDKLSRALRYYYDKNIIKKVLGQKFVYRFVSFP

EIVKTESKIPFHVKMESLGTNNTCKPAGGMSPYSPGASSLQPGAPSPSSSSHHGGSRLPS

PAHAPPHHLATPPPAHIGSYTSIGPLAHMYSTAFSHIAAASLGGYLSALARRPEPETPTT

PTQPPSSLWPPDFGHRSPSPPSEDSKDGILSAPLHLVTEEASRRRREEEAEERRREEQRR

PRSRSPQSHSPYAHSPVQRMRSPIRPRSPLPSPPPLSPLTPPPIREHRTPSISVSAAASS

ASSTPSIAGNKSGVGKVRSGPKPKPSPLSIEAITGSPPVLSPRQFSASSLNTPLVNLPSP

PYSGVGTKSPYLPLHLWSSLSPLASLSPHYGSPRNQHHFTFPALPQYSPLPPPVVSPLLP

TTAFDFSALTSPSERTASVPVQQ*

>maker-27444_pilon_pilon_pilon-augustus-gene-1.23-mRNA-1-DNA_novel01

MVTFYQSDSMTGDGEEVSAMEDVVEYTDGMVNNITLWQFLLELLLSNQYKHIITWTNSEG

EFKLLNAEEVARLWGLRKNKTNMNYDKLSRALRYYYDKNIIKKVLGQKFVYRFVSFPEIV

KTESKIPFHVKMESLGTNNTCKPAGGMSPYSPGASSLQPGAPSPSSSSHHGGSRLPSPAH

APPHHLATPPPAHIGSYTSIGPLAHMYSTAFSHIAAASLGGYLSALARRPEPETPTTPTQ

PPSSLWPPDFGHRSPSPPSEDSKDGILSAPLHLVTEEASRRRREEEAEERRREEQRRPRS

RSPQSHSPYAHSPVQRMRSPIRPRSPLPSPPPLSPLTPPPIREHRTPSISVSAAASSASS

TPSIAGNKSGVGKVRSGPKPKPSPLSIEAITGSPPVLSPRQFSASSLNTPLVNLPSPPYS

GVGTKSPYLPLHLWSSLSPLASLSPHYGSPRNQHHFTFPALPQYSPLPPPVVSPLLPTTA

FDFSALTSPSERTASVPVQQ*

>maker-29982_pilon_pilon_pilon-augustus-gene-0.40-mRNA-1-DNA_novel01

MTSADIGIMDFLFEKDDSSPKGLTLSGDGFKNMIFSSSDKLDLDTPDIKEELDWKMENDW

DADELLRSVLNGSDVVSAPLPLNIHGPTLASSPAPLGSSAPMSVPSLGGTSPLSTPTLKN

DDDIDDLKDLVGLPFDVVLDSGASDSGMSSDSNFDQQMSPLSGCSVASPGSFVDMGLGIG

GFSSSESGADTVSEVSSVSDLGSSSPHSASTLLAPGSPEMLLGADDFITQTDSGFSSPAP

QIVPELFTTNVTSCPVSTYNTTVGSRTPTRVSNRPPNSKVSPVVSTVSNIVGSSSPILKT

VSVAAPSSGTTSSVSTLGGQTIVVTQASKGGPTGQLVKGGTLLKSNPTSRSILLPVTVKD

FNQVRTIKIISTGGSSNVGVNGRGVRTVVSTVRQPNASAATVTTSLSNPAAIRTALLTTV

RGQDSKSLLKGGAATTVVTSARPSGASKVCATLASPTSTKTDFDDDDEDDDESRTSFQQL

TLSDEEKRLLKKEGITLPTHYPLTKHEERELKRIRRKIRNKISAQDSRKRKKEYIDGLED

RVKACTEENQQLQKRIRSLEAQNESLLVQMRKFQNAVIGGSNNGRSQVHASTALMMLILS

TALFVVPSLRQDTKVSDNELSLPSTKISNPGHRTLLEAGSGAIVPSTLEALETLDDEIAS

DLDSPLVQALTDHDYTPATKRQRTLSKNNGYYVPPLDDFPPQLTKTDSGGGFPPDHGSGS

KIAARIMDAVVDHSVNNFSDPGGKHANPHTVVVNLGKAQKRLRDDLELE*

>maker-29982_pilon_pilon_pilon-augustus-gene-0.40-mRNA-1-DNA_novel02

MTSADIGIMDFLFEKDDSSPKGLTLSGDGFKNMIFSSSDKLDLDTPDIKEELDWKMENDW

DADELLRSVLNGSDVVSAPLPLNIHGPTLASSPAPLGSSAPMSVPSLGGTSPLSTPTLKN

DDDIDDLKDLVGLPFDVVLDSGASDSGMSSDSNFDQQMSPLSGCSVASPGSFVDMGLGIG

GFSSSESGADTVSEVSSVSDLGSSSPHSASTLLAPGSPEMLLGADDFITQTDSGFSSPAP

QIVPELFTTNVTSCPVSTYNTTVGSRTPTRVSNRPPNSKVSPVVSTVSNIVGSSSPILKT

VSVAAPSSGTTSSVSTLGGQTIVVTQASKGGPTGQLVKGGTLLKSNPTSRSILLPVTVKD

FNQVRTIKIISTGGSSNVGVNGRGVRTVVSTVRQPNASAATVTTSLSNPAAIRTALLTTV

RGQDSKSLLKGGAATTVVTSARPSGASKVCATLASPTSTKTDFDDDDEDDDESRTSFQQL

TLSDEEKRLLKKEGITLPTHYPLTKHEERELKRIRRKIRNKISAQDSRKRKKEYIDGLED

RVKACTEENQQLQKRIRSLEAQNESLLVQMRKFQNAVIGGSNNGRSQVHASTALMMLILS

TALFVVPSLRQDTKVSDNELSLPSTKISNPGHRTLLEAGSGAIVPSTLEALETLDDEIAS

DLDSPLVQALTDHDYTPATKRQRTLSKNNGYYVPPLDDFPPQLTKTDSGGGFPPDHGSGS

KIAARIMDAVVDHSVNNFSDPGGKHANPHTVVVNLGKAQKRLRDDLELE*

>maker-29982_pilon_pilon_pilon-augustus-gene-0.40-mRNA-1-DNA_novel03

MNAQDFCELLMENDWDADELLRSVLNGSDVVSAPLPLNIHGPTLASSPAPLGSSAPMSVP

SLGGTSPLSTPTLKNDDDIDDLKDLVGLPFDVVLDSGASDSGMSSDSNFDQQMSPLSGCS

VASPGSFVDMGLGIGGFSSSESGADTVSEVSSVSDLGSSSPHSASTLLAPGSPEMLLGAD

DFITQTDSGFSSPAPQIVPELFTTNVTSCPVSTYNTTVGSRTPTRVSNRPPNSKVSPVVS

TVSNIVGSSSPILKTVSVAAPSSGTTSSVSTLGGQTIVVTQASKGGPTGQLVKGGTLLKS

NPTSRSILLPVTVKDFNQVRTIKIISTGGSSNVGVNGRGVRTVVSTVRQPNASAATVTTS

LSNPAAIRTALLTTVRGQDSKSLLKGGAATTVVTSARPSGASKVCATLASPTSTKTDFDD

DDEDDDESRTSFQQLTLSDEEKRLLKKEGITLPTHYPLTKHEERELKRIRRKIRNKISAQ

DSRKRKKEYIDGLEDRVKACTEENQQLQKRIRSLEAQNESLLVQMRKFQNAVIGGSNNGR

SQVHASTALMMLILSTALFVVPSLRQDTKVSDNELSLPSTKISNPGHRTLLEAGSGAIVP

STLEALETLDDEIASDLDSPLVQALTDHDYTPATKRQRTLSKNNGYYVPPLDDFPPQLTK

TDSGGGFPPDHGSGSKIAARIMDAVVDHSVNNFSDPGGKHANPHTVVVNLGKAQKRLRDD

LELE*

>maker-3820_pilon_pilon_pilon-augustus-gene-5.30-mRNA-1-DNA_novel01

MSAMSQIMDATYCIGGDMDFGRMFDQYLHKPAYGSYGSEENYSDLQGLTSTLNQQEPDTT

SAPHSHPHHPHSMYQHQMGVTGAMGSPNPQFPPQNLYIPPISQNSHMASVHLYRTTGYMV

HPACGPPTPTYTNASLAMHGAGPPQQGHMMAGVGGMMGGYYPRPPMARNGGSPNSGGSPS

PGSEDSDDSTPLAQMGRGKRPSSDSVEGPARGKKPKVSKKGKKKRDPNEPQKPVSAYALF

FRDTQAAIKGSNPNASFGEVSKIVASMWDSLDADHKNVYKKKTEAAKKEYLKALAAYRAS

LVSKGGENENVYGAPGYGQFAPGFGYPPTTTAPGHPQVQSPTQMSPLAKKSPLLSSLMAD

PPPPMMTPPMSQPMPPSGPPMMPHGSMPPMSAASMAPQPQQGPPQAAHQPPTPQPHLTPM

TNVPTTSPYMNQQAPGSMTSPGGGGSPSSAQQPQQHQNPQQQAAAAATQHQQQQAGQQQQ

QHQQSGQHGQQQQQQQQQGQQQQGQQQQQQQQQQQQGQPSGQGSGGQQQGMGQPGHVGCP

TSGPSCIRNGCTNPAVHNPEWEDEYCSNECVVSHCRDVFTTWVASNHHQQTPPSYPAPVK

*

>maker-3922_pilon_pilon_pilon-augustus-gene-2.44-mRNA-1-DNA_novel01

MNEEEFLLKWNNHQTNFVDVFNHLLRDDSFVDVTLVCGGEAVLGHKMVLAACSPLLHRIL

RDNPCKHPVLILSDVPAEDMRAMMKFIYQGEVSVSQSELASFLKTADNLQIKGLAEDKEK

DKEHKSKEERKRKDKEDRSEWRSSNKRQRLSDSRATETCSPLLSGSFKTISDSHSSHKSK

SKTSNDDHSSKSSGVGGVSTLHVPSSKHASSKTSSSKIVVHGVPNKVLNTCSTKENCTIQ

TNSSISMEENNLLKANLSEDLTIKTEPLDYPGEMVWTEGHGAINSDLLDDPQPIMPLGAI

SGALESVAVALTASPNLPVLETNEDGRCDEESRGDMSTASDDINANTTAGEVEEVEDETT

TTGSPSKFRGGRKACCFCGKTFCDNYTLRRHMVTHLSRERQFECPMCSAAYSRKDHLMAH

IRRKHPISDDVEKIEYNTGQAS*

>maker-5736_pilon_pilon_pilon-augustus-gene-5.22-mRNA-1-DNA_novel01

MPKHSKKDSSDSDSDSGPEDRAPPPKKSKSGGGDGAAKTNDSGESYFDIDGLRRVTVREF

KGRVYVDIREFYEKDGKVLPGKKGISLSTSQWGKVKSLMDSIDGAVEQLR*

>maker-6504_pilon_pilon_pilon-augustus-gene-4.16-mRNA-1-DNA_novel01

MGDGMLSLSWNNHKATFCHILANLRDKERYTDVTLACEGKFYRVHKLVLSTCSEYFEVMF

ENTPCKHPVIVLNHIKHDEIEALLSYMYAGVVNVAQNDLARLIKAAELLKIKGLAVPDEP

PQESEVRKSHPSPWSSRDERTSPLPKRRRKDDSGTPQGGSQTSNSPASSPRASPFMDNSD

YGERSRTHSEGQISDHRGERTDLRSDPLSTRLDDISDKQIDHRSSTEQSPAQAIVDETLV

KEEILDTADDSRDNMLDSGLDYGSLGAENRLDSSNDASNTVLPNKFDTPMMPSQTQLSEP

VVEALAGPSGMQGWLGDMGGGFSSVESFRGEGSQQDMRQPLPTQQPHQMVVLNRAGQRTV

NGGALGLKNHHIIPHRKLLKCSYCPFTTHCHNNYKRHMSTHLNNNNNNNKKPYAAITALT

NLIQMI*

>maker-6504_pilon_pilon_pilon-augustus-gene-4.16-mRNA-1-DNA_novel02

MGDGMLSLSWNNHKATFCHILANLRDKERYTDVTLACEGKFYRVHKLVLSTCSEYFEVMF

ENTPCKHPVIVLNHIKHDEIEALLSYMYAGVVNVAQNDLARLIKAAELLKIKGLAVPDEP

PQESEVRKSHPSPWSSRDERTSPLPKRRRKDDSGTPQGGSQTSNSPASSPRASPFMDNSD

YGERSRTHSEGQISDHRGERTDLRSDPLSTRLDDISDKQIDHRSSTEQSPAQAIVDETLV

KEEILDTADDSRDNMLDSGLDYGSLGAENRLDSSNDASNTVLPNKFDTPMMPSQTQLSEP

VVEALAGPSGMQGWLGDMGGGFSSVESFRGEGSQQDMRQPLPTQQPHQMMGMSFGGGNGG

GVGDGVGVGGLRMRFGNRSGGATAGAPCIYTSSHKLHHCPYCTSYTRKASYVSSFVLSAT

GHHLSA*

>maker-7896_pilon_pilon_pilon-augustus-gene-1.40-mRNA-1-DNA_novel01

MPPVEIQWCDHSSVLLEYLRQLYEEGRFVDVGLECEGRQVKAHKAVISACSPYLEGLLKG

NPARSHLIKFNGYSYTSLRAVIDFMYNGKVMVEQEQLAAFLKLGRALQVRGILDIAAQCE

EAGLTTAEETKSNAQYSTTVQQPPKAAPQTPAAKAVQQSGATAAETKKYAFLLDKGSTNP

TKKPGPQFRKVMPNIIGTVDQQGNIAKVQGIGAQQVAQQQTHHAQTVQQAQPTQTVQQVV

QQGQAVQRQQVQQATEETVGDDQSQQQQQQEAAPTTLEELQQALSSGQAILQTADNGSEQ

TLQMVEGQCVVTGSGQTYVVVQEGDNISFVPAEMVQGDLGPEVNGGQVVEGLDPVEQQLA

QMEGQVLSHNQVIEGSGQQQTTGNTGQAGSGANAGGDSGSLDTMSVVESLVKLGEKQPTE

NENGSGEEATEVGADMPSLDGDGTDDATEVGASMPCIGEEDETGAGEGEEEMETDVATEE

DTGKRRRQPTQKMIEAMGMLKRKIPESPTQGGRGKKMK*

>maker-7896_pilon_pilon_pilon-augustus-gene-1.40-mRNA-1-DNA_novel02

MPPVEIQWCDHSSVLLEYLRQLYEEGRFVDVGLECEGRQVKAHKAVISACSPYLEGLLKG

NPARSHLIKFNGYSYTSLRAVIDFMYNGKVMVEQEQLAAFLKLGRALQVRGILDIAAQCE

EAGLTTAEETKSNAQYSTTVQQPPKAAPQTPAAKAVQQSGATAAETKKYAFLLDKGSTNP

TKKPGPQFRKVMPNIIGTVDQQGNIAKVQGIGAQQVAQQQTHHAQTVQQAQPTQTVQQVV

QQGQAVQRQQVQQATEETVGDDQSQQQQQQEAAPTTLEELQQALSSGQAILQTADNGSEQ

TLQMVEGQCVVTGSGQTYVVVQEGDNISFVPAEMVQGDLGPEVNGGQVVEGLDPVEQQLA

QMEGQVLSHNQVIEGSGQQQTTGNTGQAGSGANAGGDSGSLDTMSVVESLVKLGEKQPTE

NENGSGEEATEVGADMPSLDGDGTDDATEVGASMPCIGEEDETGAGEGEEEMETDVATEE

DTGKRRRQPTQKMIEAMGMLKRKIPESPTQGGRGKKMK*

>maker-7896_pilon_pilon_pilon-augustus-gene-1.40-mRNA-1-DNA_novel03

MPPVEIQWCDHSSVLLEYLRQLYEEGRFVDVGLECEGRQVKAHKAVISACSPYLEGLLKG

NPARSHLIKFNGYSYTSLRAVIDFMYNGKVMVEQEQLAAFLKLGRALQVRGILDIAAQCE

EAGLTTAEETKSNAQYSTTVQQPPKAAPQTPAAKAVQQSGATAAETKKYAFLLDKGSTNP

TKKPGPQFRKVMPNIIGTVDQQGNIAKVQGIGAQQVAQQQTHHAQTVQQAQPTQTVQQVV

QQGQAVQRQQVQQATEETVGDDQSQQQQQQEAAPTTLEELQQALSSGQAILQTADNGSEQ

TLQMVEGQCVVTGSGQTYVVVQEGDNISFVPAEMVQGDLGPEVNGGQVIEGSGQQQTTGN

TGQAGSGANAGGDSGSLDTMSVVESLVKLGEKQPTENENGSGEEATEVGASMPCIGEEDE

TGAGEGEEEMETDVATEEDTGKRRRQPTQKMIEAMGMLKRKIPESPTQGGRGKKMK*

>maker-7896_pilon_pilon_pilon-augustus-gene-1.40-mRNA-1-DNA_novel04

MPPVEIQWCDHSSVLLEYLRQLYEEGRFVDVGLECEGRQVKAHKAVISACSPYLEGLLKG

NPARSHLIKFNGYSYTSLRAVIDFMYNGKVMVEQEQLAAFLKLGRALQVRGILDIAAQCE

EAGLTTAEETKSNAQYSTTVQQPPKAAPQTPAAKAVQQSGATAAETKKYAFLLDKGSTNP

TKKPGPQFRKVMPNIIGTVDQQGNIAKVQGIGAQQVAQQQTHHAQTVQQAQPTQTVQQVV

QQGQAVQRQQVQQATEETVGDDQSQQQQQQEAAPTTLEELQQALSSGQAILQTADNGSEQ

TLQMVEGQCVVTGSGQTYVVVQEGDNISFVPAEMVQGDLGPEVNGGQVVEGLDPVEQQLA

QMEGQVLSHNQVIEGSGQQQTTGNTGQAGSGANAGGDSGSLDTMSVVESLVKLGEKQPTE

NENGSGEEATEVGASMPCIGEEDETGAGEGEEEMETDVATEEDTGKRRRQPTQKMIEAMG

MLKRKIPESPTQGGRGKKMK*

>maker-7896_pilon_pilon_pilon-augustus-gene-1.40-mRNA-1-DNA_novel05

MPPVEIQWCDHSSVLLEYLRQLYEEGRFVDVGLECEGRQVKAHKAVISACSPYLEGLLKG

NPARSHLIKFNGYSYTSLRAVIDFMYNGKVMVEQEQLAAFLKLGRALQVRGILDIAAQCE

EAGLTTAEETKSNAQYSTTVQQPPKAAPQTPAAKAVQQSGATAAETKKYAFLLDKGSTNP

TKKPGPQFRKVMPNIIGTVDQQGNQCLV*

>maker-7896_pilon_pilon_pilon-augustus-gene-1.40-mRNA-1-DNA_novel06

MPPVEIQWCDHSSVLLEYLRQLYEEGRFVDVGLECEGRQVKAHKAVISACSPYLEGLLKG

NPARSHLIKFNGYSYTSLRAVIDFMYNGKVMVEQEQLAAFLKLGRALQVRGILDIAAQCE

EAGLTTAEETKSNAQYSTTVQQPPKAAPQTPAAKAVQQSGATAAETKKYAFLLDKGSTNP

TKKPGPQFRKVMPNIIGTVDQQGNIAKVQGIGAQQVAQQQTHHAQTVQQAQPTQTVQQVV

QQGQAVQRQQVQQATEETVGDDQSQQQQQQEAAPTTLEELQQALSSGQAILQTADNGSEQ

TLQMVEGQCVVTGSGQTYVVVQEGDNISFVPAEMVQGDLGPEVNGGQVVEGLDPVEQQLA

QMEGQVLSHNQVIEGSGQQQTTGNTGQAGSGANAGGDSGSLDTMSVVESLVKLGEKQPTE

NENGSGEEATEVGASMPCIGEEDETGAGEGEEEMETDVATEEDTGKRRRQPTQKMIEAMG

MLKRKIPESPTQGGRGKKMK*

>maker-7896_pilon_pilon_pilon-augustus-gene-1.40-mRNA-1-DNA_novel07

MPPVEIQWCDHSSVLLEYLRQLYEEGRFVDVGLECEGRQVKAHKAVISACSPYLEGLLKG

NPARSHLIKFNGYSYTSLRAVIDFMYNGKVMVEQEQLAAFLKLGRALQVRGILDIAAQCE

EAGLTTAEETKSNAQYSTTVQQPPKAAPQTPAAKAVQQSGATAAETKKYAFLLDKGSTNP

TKKPGPQFRKVMPNIIGTVDQQGNIAKVQGIGAQQVAQQQTHHAQTVQQAQPTQTVQQVV

QQGQAVQRQQVQQATEETVGDDQSQQQQQQEAAPTTLEELQQALSSGQAILQTADNGSEQ

TLQMVEGQCVVTGSGQTYVVVQEGDNISFVPAEMVQGDLGPEVNGGQVVEGLDPVEQQLA

QMEGQVLSHNQVIEGSGQQQTTGNTGQAGSGANAGGDSGSLDTMSVVESLVKLGEKQPTE

NENGSGEEATEVGASMPCIGEEDETGAGEGEEEMETDVATEEDTGKRRRQPTQKMIEAMG

MLKRKIPESPTQGGRGKKMK*

>maker-8134_pilon_pilon_pilon-augustus-gene-1.13-mRNA-1-DNA_novel01

METGLLWAKLFYQQQGSMKIQLTEQKAKESYARSVANSRWCADTMRNLVYYTVMGPCKWV

QLSLHLIRGRSYNLFENEDGSDVILMVGDGSYRERVPAHSWVLAAGNQYFRALFQGPFAD

RHKKTYDIPNDPKGFQNLLKWLYRHECGIQSMDSALITLQVAIEYLCPELAELCVEYIGK

HLRVSNVLKVLQFTWRYCPSSQTSDDSQGSSVISTAPSAPSLEIMDDPEDDDDDTNSFGA

ADRLQQLDLSQHTVHSMSQLQKLSEEAEFQDPTACCSDLYNVCLEVVDSATQAVLASEPL

EELDYRTLQLILKRSTLNIPNELPIFHAVQRWSTAECKRRMLPLTSDNRRAVLGDLLYYV

RFLNMTNEQLQQTSSLLTADEFNYLATRISGHTPATIPATLNPHLAMMATPRHHKPPTLP

TTDSTNKNKKKGTTGKRKYTKKELMLDVVSCLAVIFD*

>maker-8134_pilon_pilon_pilon-augustus-gene-1.13-mRNA-1-DNA_novel02

MRQILKRMVCGTQSKLRKYKAEMQKLLSVNTEQKALCDPARIEFLLPRRVNSASSTPELC

YKTSSSSSSSSPSSGPSSMHLFIRSYNLFENEDGSDVILMVGDGSYRERVPAHSWVLAAG

NQYFRALFQGPFADRHKKTYDIPNDPKGFQNLLKWLYRHECGIQSMDSALITLQVAIEYL

CPELAELCVEYIGKHLRVSNVLKVLQFTWRYCPSSQTSDDSQGSSVISTAPSAPSLEIMD

DPEDDDDDTNSFGAADRLQQLDLSQHTVHSMSQLQKLSEEAEFQDPTACCSDLYNVCLEV

VDSATQAVLASEPLEELDYRTLQLILKRSTLNIPNELPIFHAVQRWSTAECKRRMLPLTS

DNRRAVLGDLLYYVRFLNMTNEQLQQTSSLLTADEFNYLATRISGHTPATIPATLNPHLA

MMATPRHHKPPTLPTTDSTNKNKKKGTTGKRKYTKKELMLDVVSCLAVIFD*

>maker-8134_pilon_pilon_pilon-augustus-gene-1.13-mRNA-1-DNA_novel03

METGLLWAKLFYQQQGSMKIQLTEQKAKESYARSVANSRWCADTMRNLVYYTVMGPCKWV

QLSLHLIRGRSYNLFENEDGSDVILMVGDGSYRERVPAHSWVLAAGNQYFRALFQGPFAD

RHKKTYDIPNDPKGFQNLLKWLYRHECGIQSMDSALITLQVAIEYLCPELAELCVEYIGK

HLRVSNVLKVLQFTWRYCPSSQTSDDSQGSSVISTAPSAPSLEIMDDPEDDDDDTNSFGA

ADRLQQLDLSQHTVHSMSQLQKLSEEAEFQDPTACCSDLYNVCLEVVDSATQAVLASEPL

EELDYRTLQLILKRSTLNIPNELPIFHAVQRWSTAECKRRMLPLTSDNRRAVLGDLLYYV

RFLNMTNEQLQQTSSLLTADEFNYLATRISGHTPATIPATLNPHLAMMATPRHHKPPTLP

TTDSTNKNKKKGTTGKRKYTKKELMLDVVSCLAVIFD*

>maker-9386_pilon_pilon_pilon-augustus-gene-0.9-mRNA-1-DNA_novel02

MEDDVMIPDDLGDEDLLSNMNWTDLDLDDIKDINLDWNEGDVLGQGTSLLEHTDPTPVTT

TNHSNTSTPTHVPSQVSVVNSNATLSGFISVSDAVNKGISQLKSGGVPLQVKVTAPQHVQ

VQKHQLIATTACGNQMRQVAHVAPRVEHASLINIPHTVVSTSGTLTQTPQLQQILTQANG

NIQQRQVEHIQTQPIQVQQSQIQQVTIPHQIGKRMATGGVSILQHPTVIQQLVLPKTDNG

TIVSTTSGQCINMPQTVLYKATPTIATIAAPVQGLDTSTVVTGIPVVFDSDRIPVARVVS

SGKPLVIPPKGEKRNSHNAIEKRYRCSINDKIVELKNLVAGEESKLHKSQILKKAIEYIR

YLQTQNSRLRVELNSYRMRDGNQKITDLLLGPYTPPPSDTSSPARSPISDSSLPPSPPIA

KIELKDEDAASPCNSPSYIPMARNMADGSRMVLCMMMLALLAFNPMSLLNKRSHSALSDT

IGEAPGRSILDFESDNDAFSFQSLFYSSLMVWLVNFVIISVFLIRIFIYGEPIMRRKSPV

AESFWRHRKQADLDMAKGNYGSACRQYGLALHVIGRPLPSSWVEKSCSVMWQFTRQIMQR

MYLGRWLAKHAGGLFLESSIREEIRESIRECSYVYHQLHKLHLMGHVEDGSNLLGLYLAL

TSINLGECGSVPGGDMADILVLGALRAKESLPELCRFIAKILMGKARWCSVLYKKNNIQW

LFSPSGNRFFVSHKWSYDAKRISMFSQLTDKADPVGYLLLLYREHLLERAITTLVNPGVK

ADDVCEESAHRRTKTSDVLDYIALLTETYNGYEASFVVSCRDEVSCWWGTWCQVIAHWLL

GEDDHAESVYAALENVPQVLKASDDPLPMAVLNAARARRQLGHGSPSSILRLCERSASAL

SDSIHQASYKHPNQMVLSVQLIIVDLLLGVRTVVWESESATDCDTLTVAPAHILRGFQND

LALLRKLVQHLPGVLARVFLHEATLRMMAGASPARTQQLLDRSLRHRYGKSSVICGKDKK

IDSFSGERERATALMLACRHLPSQLLSSPGERAGMLSEAAKTLEKIGDKQRLQDCYNMMK

NLGTGVSSYC*

>augustus-28854_pilon_pilon_pilon-processed-gene-0.27-mRNA-1-DNA_novel01

GPGNTRAQCPLSTSQGSDREGSAGISGLEKRQFKGKRSRKRTRQSRTNLRLLKRHRDESK

AKHLKSSSDNVESNAGSKSISENMISISSKYSQKFSKKRDADIQNIGKENDLTDDEQEAC

VAVKTDYNNVEISSGGKRDYEVIVSGKVTGDEPYDSANRDAHVNDTASTVNSPNDSFESD

VDQDGTNDDKSSENEIQESSCDISLDVDCSHADRESGVTDKVASHGAVCEVNSETPLGAV

ISEESIDGDNSCTLVIHEEETSESLSRDTPVEADKSLLQEEVKRDRDEGLPAEKTVLNQK

ERTSETPDIQFDFYSYMKDKTNTSESFVTPNQEISQSASDGQHNEYNDDNGEEDSDDDSS

SVTSSSSSSSSSSSSSSSDDDDRINFKQRVERAAQAAREAEASAAALKSKSGIHQGGVRE

NEVSLQTRPLIHMTSVCPSSSSVEQSDNDNSLTNSVAAPASQIFAPPSSSSHALAPTSML

CMKPNEVGQNHPSLNPGLNCPEPEEIRHCGRDPADLQAAQGLADLQRGQDLVLNSSVKTT

PSSMNYTRNQPMETPFEIRRCAGGKRVECVVCGKTYGRLIKLKQHHLTHTGERPWVCNTC

GKTFTKSAYLKDHCLKCHTPADQKRFSCYVCHKGFGVKSDLNAHLATHATNKPFACDICG

QTFALKFTRDTHKAQHTGSRPWACNVCGRLYVTKYKLRAHMKAHTGELVCSVCGRHFTSQ

DSLGRHQRLHENSGKSNDLPACHICNKKYATPSRLKRHVLTHERQNSFKCSICGEEFMNH

QLLAAHQKRHPQSGVQPSHRCHICNKLYVRESDLARHIAKHNATGFCQEANSSSVPVPQN

KKCNTDAADGGNPHSLNTLSEPNAACFIPNKKTELYTQSQTTSLMTNNALAQQTLGTNSY

LDMSQSQPHSAPLAQAQHSSGITQTPLAGHVSQSHPLARTLAQSQHVNVLTQSQHSGTVP

SQPLANPISQPQLVGGMPHPHSVVMSPSHQITQPQQQLARPVSQSQHIVIMAQTQHTQAI

ASVQHPRGMPQTQQLTSGISQVPHSRPLPQQQQTLHMPNQQLNSVVVGQPQQQESLLDSR

TQSNSQTSTLSAPVYMSSVQYNSDILRANMQENSYQHPPRQK*

>maker-10900_pilon_pilon_pilon-augustus-gene-3.27-mRNA-1

MTSPEFQFIFPPEAPVFKPTWEEFQDALGYINKIRPIAEQSGICKIIPPPDWQPPFAVDV

DNFKFTPRIQRLNELEAHTRIKLNFLDQIAKFWELQGSSLKIPVVERKALDLYSLHRHVW

REGGIDQVTKERKWNKIASQIGFPSNKNLGTLLKSHYERILLPFDIFNKGKEIPSLKCEV

KTEVSDKDYKPHKIPSRMAGKIPESEKNGRRSRRHPDAGDIKADTQVGFGSDDEEEGKGS

KELKRLQFYGAGPKMAGFEKASPEDKGKTRGKKVDYSDLDPLAKYICLNCGRGDAEDQML

LCDGCDDSYHIFCLVPPLLDIPKGDWRCPKCVAVEVSKPVEAFGFEQAQKEYTLQSFGEM

ADKFKQDYFSMPVHLIPTEMAEREFWRIVSSIDEDVTVEYGADLHTMDHGSGFPTKNSKN

LLPADQEYAISNWNLNNLPVLEGSVLGHINCDISGMKVPWMYVGMCFSTFCWHNEDHWSY

SINYLHWGEPKTWYGVPGGEAEKFEAAMKGAAPELFQAQPDLLHQLVTIMNPNILMKSGV

PIVRTNQHAGEFVITFPRSYHAGFNQGYNFAEAVNFAPPDWLAIGRECVLHYSNLKRYCV

FSHDELVCKMASTPDELDLTVAAATYQDMLRMVDLEKKLRRSLLHWGVTNAEREAFELLP

DDERQCDFCKTTCFLSAVTCTCTHDRLVCLRHYKLLCECPPENHTLRYRYTLDELPPLLQ

RLKVRAESFDHWAVKVKEALEAKPDDKLELSDLKELVEEAEAQRFPDSELLQTLIQAVTE

ADKCATVAAHLATKKVRTRTRGSGEAKSRLTLEELRLFHEQIESLACTIREGEAVKELLT

RVTAFQEEATELLNQQMPESETIAKMVDTGCSLDIDLPELPRLKLKLQQVEWLEEVEETL

DDSSSLTVECLRKLLDAGISLPPHPRIERSLAQLQQLLNNMEMWEEKANQALNAKPGLSV

SEGEELVRQAEEVPAHLPSVIALKDAVKRAREWTNKVTTLQNGETAPLLETVEGLVSRGR

PIPLHLEPLADLEENVAAAHSWLEKTARTFLKKNSHLTLLEVLVPRTDIGVVSGKRKRVK

REEMSAQQQQAQLLDISFEDTANPATVVKAFKECEERELGAMRALRAHNRNKRDGEAVQE

GATFCICHKAPQPNMLTCSLCLDLFHSGCVPSSHRTKVKLSSEGRFLCPSCERSRRPRLE

TILSLLVALQKLPVRLPEAEALQCLTERGMSWQDRARKLMKTDELTAALGKLSVFSQKLI

EAQAKQHKPTSSAVRDEEKMEVEDSTNDDSLMSEDGDKECRADNSKIDSEKEPNKKSEEG

SWEHAYSSNKGNREKKTEIDKARWSRLVASVMENPNGPLITLSPNIRSQVQDLLVEGELL

EVSLDETIHLWRVLQAANHPPAMLEDTLLSLNSTSNTNEDKAKKAVPKKRKSQGEGSEDM

QPKSPQKSPTKKTKLIKPGALNADGKITKGIIKKKAIAKLAGTGGGGGGGGIQPTTGMGL

SGGLDKKKREGLMPNKDGVKKKGCGKKKRAVQSTPSDDDDDDNDEACAAVRCFHPTGQNV

DWVQCDACEGWFHYICVGLTSADVIEEEEYICKRCLPKRVNKAAGTKASGAAKAVAPKPG

ELTEEESVDILLSLASSLPPKE*

>maker-10900_pilon_pilon_pilon-augustus-gene-3.27-mRNA-1-DNA_novel01

MTSPEFQFIFPPEAPVFKPTWEEFQDALGYINKIRPIAEQSGICKIIPPPDWQPPFAVDV

DNFKFTPRIQRLNELEAHTRIKLNFLDQIAKFWELQGSSLKIPVVERKALDLYSLHRHVW

REGGIDQVTKERKWNKIASQIGFPSNKNLGTLLKSHYERILLPFDIFNKGKEIPSLKCEV

KTEVSDKDYKPHKIPSRMAGKIPESEKNGRRSRRHPDAGDIKADTQVGFGSDDEEEGKGS

KELKRLQFYGAGPKMAGFEKASPEDKGKTRGKKVDYSDLDPLAKYICLNCGRGDAEDQML

LCDGCDDSYHIFCLVPPLLDIPKGDWRCPKCVAVEVSKPVEAFGFEQAQKEYTLQSFGEM

ADKFKQDYFSMPVHLIPTEMAEREFWRIVSSIDEDVTVEYGADLHTMDHGSGFPTKNSKN

LLPADQEYAISNWNLNNLPVLEGSVLGHINCDISGMKVPWMYVGMCFSTFCWHNEDHWSY

SINYLHWGEPKTWYGVPGGEAEKFEAAMKGAAPELFQAQPDLLHQLVTIMNPNILMKSGV

PIVRTNQHAGEFVITFPRSYHAGFNQGYNFAEAVNFAPPDWLAIGRECVLHYSNLKRYCV

FSHDELVCKMASTPDELDLTVAAATYQDMLRMVDLEKKLRRSLLHWGVTNAEREAFELLP

DDERQCDFCKTTCFLSAVTCTCTHDRLVCLRHYKLLCECPPENHTLRYRYTLDELPPLLQ

RLKVRAESFDHWAVKVKEALEAKPDDKLELSDLKELVEEAEAQRFPDSELLQTLIQAVTE

ADKCATVAAHLATKKVRTRTRGSGEAKSRLTLEELRLFHEQIESLACTIREGEAVKELLT

RVTAFQEEATELLNQQMPESETIAKMVDTGCSLDIDLPELPRLKLKLQQVEWLEEVEETL

DDSSSLTVECLRKLLDAGISLPPHPRIERSLAQLQQLLNNMEMWEEKANQALNAKPGLSV

SEGEELVRQAEEVPAHLPSVIALKDAVKRAREWTNKVTTLQNGETAPLLETVEGLVSRGR

PIPLHLEPLADLEENVAAAHSWLEKTARTFLKKNSHLTLLEVLVPRTDIGVVSGKRKRVK

REEMSAQQQQAQLLDISFEDTANPATVVKAFKECEERELGAMRALRAHNRNKRDGEAVQE

GATFCICHKAPQPNMLTCSLCLDLFHSGCVPSSHRTKVKLSSEGRFLCPSCERSRRPRLE

TILSLLVALQKLPVRLPEAEALQCLTERGMSWQDRARKLMKTDELTAALGKLSVFSQKLI

EAQAKQHKPTSSAVRDEEKMEVEDSTNDDSLMSEDGDKECRADNSKIDSEKEPNKKSEEG

SWEHAYSSNKGNREKKTEIDKARWSRLVASVMENPNGPLITLSPNIRSQVQDLLVEGELL

EVSLDETIHLWRVLQAANHPPAMLEDTLLSLNSTSNTNEDKAKKAVPKKRKSQGEGSEDM

QPKSPQKSPTKKTKLIKPGALNADGKITKGIIKKKAIAKLAGTGGGGGGGGIQPTTGMGL

SGGLDKKKREGLMP

>maker-10900_pilon_pilon_pilon-augustus-gene-3.27-mRNA-1-DNA_novel02

MTSPEFQFIFPPEAPVFKPTWEEFQDALGYINKIRPIAEQSGICKIIPPPDWQPPFAVDV

DNFKFTPRIQRLNELEAHTRIKLNFLDQIAKFWELQGSSLKIPVVERKALDLYSLHRHVW

REGGIDQVTKERKWNKIASQIGFPSNKNLGTLLKSHYERILLPFDIFNKGKEIPSLKCEV

KTEVSDKDYKPHKIPSRMAGKIPESEKNGRRSRRHPDAGDIKADTQVGFGSDDELAKYIC

LNCGRGDAEDQMLLCDGCDDSYHIFCLVPPLLDIPKGDWRCPKCVAVEVSKPVEAFGFEQ

AQKEYTLQSFGEMADKFKQDYFSMPVHLIPTEMAEREFWRIVSSIDEDVTVEYGADLHTM

DHGSGFPTKNSKNLLPADQEYAISNWNLNNLPVLEGSVLGHINCDISGMKVPWMYVGMCF

STFCWHNEDHWSYSINYLHWGEPKTWYGVPGGEAEKFEAAMKGAAPELFQAQPDLLHQLV

TIMNPNILMKSGVPIVRTNQHAGEFVITFPRSYHAGFNQGYNFAEAVNFAPPDWLAIGRE

CVLHYSNLKRYCVFSHDELVCKMASTPDELDLTVAAATYQDMLRMVDLEKKLRRSLLHWG

VTNAEREAFELLPDDERQCDFCKTTCFLSAVTCTCTHDRLVCLRHYKLLCECPPENHTLR

YRYTLDELPPLLQRLKVRAESFDHWAVKVKEALEAKPDDKLELSDLKELVEEAEAQRFPD

SELLQTLIQAVTEADKCATVAAHLATKKVRTRTRGSGEAKSRLTLEELRLFHEQIESLAC

TIREGEAVKELLTRVTAFQEEATELLNQQMPESETIAKMVDTGCSLDIDLPELPRLKLKL

QQVEWLEEVEETLDDSSSLTVECLRKLLDAGISLPPHPRIERSLAQLQQLLNNMEMWEEK

ANQALNAKPGLSVSEGEELVRQAEEVPAHLPSVIALKDAVKRAREWTNKVTTLQNGETAP

LLETVEGLVSRGRPIPLHLEPLADLEENVAAAHSWLEKTARTFLKKNSHLTLLEVLVPRT

DIGVVSGKRKRVKREEMSAQQQQAQLLDISFEDTANPATVVKAFKECEERELGAMRALRA

HNRNKRDGEAVQEGATFCICHKAPQPNMLTCSLCLDLFHSGCVPSSHRTKVKLSSEGRFL

CPSCERSRRPRLETILSLLVALQKLPVRLPEAEALQCLTERGMSWQDRARKLMKTDELTA

ALGKLSVFSQKLIEAQAKQHKPTSSAVRDEEKMEVEDSTNDDSLMSEDGDKECRADNSKI

DSEKEPNKKSEEGSWEHAYSSNKGNREKKTEIDKARWSRLVASVMENPNGPLITLSPNIR

SQVQDLLVEGELLEVSLDETIHLWRVLQAANHPPAMLEDTLLSLNSTSNTNEDKAKKAVP

KKRKSQGEGSEDMQPKSPQKSPTKKTKLIKPGALNADGKITKGIIKKKAIAKLAGTGGGG

GGGGIQPTTGMGLSGGLD

>maker-12382_pilon_pilon_pilon-augustus-gene-0.17-mRNA-1-DNA_novel01

MAINPRKDGGPNAKYYESTETVSQFENVKTWLMKNAKKYVAADPPNNKGLAQLVVQLLQF

MEDNFGPKVTKAPMTKLPMKCFLDFKPGGGLCHILSTVYKFKSDQGWRRFDFQSPSRKDT

NIELFKEIEKSLLSNKCWAKPSVFIHGEVDKELRNELKDIVKRHDGKIVEEEEEASHVVF

DEQDPLDDEFARAVMRRDRQILFHFYYMPDSYDSWASGVELDYDPPESPQFFDGQWKVCA

NWLQDLESYNEWMNEEDYEVDDNGTKKKHRGCLSVDDTMGLSDPDKKKKAKKRPRSPPSP

RTKRKGARGAATPGKPVKKIKTDEEEEDLTRDMEDPVAEPTVTEVTTSSKIQPSKAGTYM

DLDEESAEKTEKPEGQPTPDGEDTATDQTHHIIVPSYAAWFDYNAIHAIEKRGLPEFFNG

KNKSKTPEIYLAYRNFMIDTYRLNPTEYLTSTACRRNLAGDVCAVMRVHAFLEQWGLINY

QVDADARPTPMGPPPTSHFHVLADTPAGLQSINPPKVNQPSAAKQLMDFDKKPVDKKPEI

NIGTEFGLKLDQYSKKTGALKNKSAASQAREWTEQEILLLLEALEMYKDDWNKVCEHVGT

RTQDECILQFLRLPIEDPYLEDPEVGNGPTASALGPLAYQPIPFSKSGNPIMSTVAFLAS

VVDPRIAASAAKAAMDEFCRIKDEVPAALLDSHIKNVADHAASNEGKVDPTAGLAKSGIA

GTEPEGEEKKESEGEAEKKETEPKKDAEKKEEEKKEEKEPEKKEEPMEVDKSSE

>maker-12382_pilon_pilon_pilon-augustus-gene-0.17-mRNA-1-DNA_novel02

MAINPRKDGGPNAKYYESTETVSQFENVKTWLMKNAKKYVAADPPNNKGLAQLVVQLLQF

MEDNFGPKVTKAPMTKLPMKCFLDFKPGGGLCHILSTVYKFKSDQGWRRFDFQSPSRKDT

NIELFKEIEKSLLSNKCWAKPSVFIHGEVDKELRNELKDIVKRHDGKIVEEEEEASHVVF

DEQDPLDDEFARAVMRRDRQILFHFYYMPDSYDSWASGVELDYDPPESPQFFDGQWKVCA

NWLQDLESYNEWMNEEDYEVDDNGTKKKHRGCLSVDDTMGLSDPDKKKKAKKRPRSPPSP

RTKRKGARGAATPGKPVKKIKTDEEEEDLTRDMEDPVAEPTVTEVTTSSKIQPSKAGTYM

DLDEESAEKTEKPEGQMHVFNLQPTPDGEDTATDQTHHIIVPSYAAWFDYNAIHAIEKRG

LPEFFNGKNKSKTPEIYLAYRNFMIDTYRLNPTEYLTSTACRRNLAGDVCAVMRVHAFLE

QWGLINYQVDADARPTPMGPPPTSHFHVLADTPAGLQSINPPKVNQPSAAKQLMDFDKKP

VDKKPEINIGTEFGLKLDQYSKKTGALKNKSAASQAREWTEQEILLLLEALEMYKDDWNK

VCEHVGTRTQDECILQFLRLPIEDPYLEDPEVGNGPTASALGPLAYQPIPFSKSGNPIMS

TVAFLASVVDPRIAASAAKAAMDEFCRIKDEVPAALLDSHIKNVADHAASNEGKVDPTAG

LAKSGIAGTEPEGEEKKESEGEAEKKETEPKKDA

>maker-12770_pilon_pilon_pilon-augustus-gene-2.36-mRNA-1-DNA_novel01

LYCTFRVKIEVNQREQINNWLLLFCCRLFKLHGSSIMDEEMLELIWTNHGPIFRYWFTEH

REKERYTDATIGCEGKLYAVHKLVLATCSNYFDEVFEHTEGKHPVLLLHDVKQHELEAIL

CYMYTGSANVSQLNLSRFMMVAKALQIKGLAVPDGDSSDPLVAVENPQVDDNKGSSSGGY

CWQESEMGSMQSMSDSSELHEDMDNVRQGAGGVGKKEDMDRKKSDTKDQYSFPGFKYKSV

EGANDGDLIKKREVLEKIISQTERQFITDGKQGITQEKAGSLQQDSAPTIKTEVDEYIDQ

KKPMKKEKLSPSECGSSKIFKGTMKRNLNGQKKSSISKVKTSKFKSMKKHLLSGEKSYKC

LQCPASFTRKYALRRHVQNLHVDVEGFVCTYCHAKFASKEFLTLHMETHDKSKPYVCTLC

HASFSRKDKLTVHIKNHETEGKGKVIAQVKNFSSKVFVCNECPATFENHSLFVEHSQMHA

GERQFPCTVCPAAFAKKVHLSRHFLTHKGFGSKDKLQDCN*

>maker-16102_pilon_pilon_pilon-augustus-gene-0.61-mRNA-1-DNA_novel01

MMECNWNSSSLPWLARDEQDFPLGAKDASYLPSATNIGLNHDVADLPDWMESRDIMVHPD

DILSIVPRHPEQMEVDCGSQGFGGYLDTDEDYGLSINPDDMLVPLSELQPEKCLSQNTIL

PAASASKVSVRVSKVRVPKISVEHSGIQNTTEMMALQPYRPTSVQSTGLVEQIDDLQNYN

ADELLDELVSLANREPNHLASLIESLDSSLVQAGMEELLSQLNANSANQEMPAPLSPVCI

PSPVPSVGSLSPQSTVDYSFHQLVSPVYYGNEVTCKSEKVTVVSSPSPVSHTDYGSSVSS

SDIEETLSIASPAIEDNLSIASPAIEDNLSIASPAIEDNLSIASPAFEVNLSVASFVKDE

EEVEVECDYIETAMHTYSRSPRKIRSKRKSSAGSAYPESRKERKKEQNKQAALRYRQKKK

QEDDELMTKIHAEEERQKQLKKKCSNLKQELTYLKKIMREVLIAKGTLSPEAFKK*

>maker-17854_pilon_pilon_pilon-augustus-gene-0.20-mRNA-1-DNA_novel01

MSVSRVPSPLPPENNMPVAENWCYTQVKVIKFSYMWAINNFSFCREEMGEVLKSSTFSAG

ANDKLKWCLRVNPKGLDEESKDYLSLYLLLVSSNKSEVRAKFKFSILNAKREETKAMESQ

RAYRFVQGKDWGFKKFIRRDFLLDEANGLLPDDKLTLYCEVSVVADSVNISGQSNAIHFK

VPDCRLSDDLGVLFESQRFSDVTLSVGGREFQAHKAILAARSPVFAAMFAHEMEERKHNR

VEIQDVDHEVLREMLRFIYTGKAANLERMADDLLAAADKYALERLKVMCEEALCTNLSTE

NAADVLILADLHSADQLKAQAIEFINTHATDVMETQGWKSMIQSHPHLIAEAFRALATQQ

IPPIGPPRKRIKASP*

>maker-17854_pilon_pilon_pilon-augustus-gene-0.20-mRNA-1-DNA_novel02

MSVSRVPSPLPPENNMPVAENWCYTQVKVIKFSYMWAINNFSFCREEMGEVLKSSTFSAG

ANDKLKWCLRVNPKGLDEESKDYLSLYLLLVSSNKSEVRAKFKFSILNAKREETKAMESQ

RAYRFVQGKDWGFKKFIRRDFLLDEANGLLPDDKLTLYCEVSVVADSVNISGQSNAIHFK

VPDCRLSDDLGVLFESQRFSDVTLSVGGREFQAHKAILAARSPVFAAMFAHEMEERKHNR

VEIQDVDHEVLREMLRFIYTGKAANLERMADDLLAAADKYALERLKVMCEEALCTNLSTE

NAADVLILADLHSADQLKAQAIEFINTHATDVMETQGWKSMIQSHPHLIAEAFRALATQQ

IPPIGPPRKRIKASP*

>maker-20258_pilon_pilon_pilon-augustus-gene-0.40-mRNA-1-DNA_novel01

MTTGGGGSGNNDASPKKKGWPKGRKRRLLPKDSNAPKGPLSGYMLFMSDQREILRQTNAG

LAFHEITKLVAQKWSQLDTSEKTKYLEAAEADKERYQKELEEYQKTDAYKNFMQKQNQDD

SPTPPKKPRKEVVEERKEEEEEEVAPTPSSLEIPIFTEEFLNHNKIREAELRQLRKSNTD

YEEQNAILQKHIESMKSTVDKLESETSQQRSNNAALQQHIETLRKTLAKAFGSLPLPGTQ

ETPTEESIDSYMVQLHSVIVNNPQQHQSLIQTVRGIVSQLALT*

>maker-20258_pilon_pilon_pilon-augustus-gene-0.40-mRNA-1-DNA_novel03

MTTGGGGSGNNDASPKKKGWPKGRKRRLLPKDSNAPKGPLSGYMLFMSDQREILRQTNAG

LAFHEITKLVAQKWSQLDTSEKTKYLEAAEADKERYQKELEEYQKTDAYKNFMQKQNQDD

SPTPPKKPRKEVVEERKEEEEEEVAPTPSSLEIPIFTEEFLNHNKIREAELRQLRKSNTD

YEEQNAILQKHIESMKSTVDKLESETSQQRSNNAALQQHIETLRKTLAKAFGSLPLPGTQ

ETPTEESIDSYMVQLHSVIVNNPQQHQSLIQTVRGIVSQLALT*

>maker-20258_pilon_pilon_pilon-augustus-gene-0.40-mRNA-1-DNA_novel04

MTTGGGGSGNNDASPKKKGWPKGRKRRLLPKDSNAPKGPLSGYMLFMSDQREILRQTNAG

LAFHEITKLVAQKWSQLDTSEKTKYLEAAEADKERYQKELEEYQKTDAYKNFMQKQNQDD

SPTPPKKPRKEVVEERKEEEEEEVAPTPSSLEIPIFTEEFLNHNKIREAELRQLRKSNTD

YEEQNAILQKHIESMKSTVDKLESETSQQRSNNAALQQHIETLRKTLAKAFGSLPLPGTQ

ETPTEESIDSYMVQLHSVIVNNPQQHQSLIQTVRGIVSQLALT*

>maker-20258_pilon_pilon_pilon-augustus-gene-0.40-mRNA-1-DNA_novel05

MEGTAIMTTGGGGSGNNDASPKKKGWPKGRKRRLLPKDSNAPKGPLSGYMLFMSDQREIL

RQTNAGLAFHEITKLVAQKWSQLDTSEKTKYLEAAEADKERYQKELEEYQKTDAYKNFMQ

KQNQDDSPTPPKKPRKEVVEERKEEEEEEVAPTPSSLEIPIFTEEFLNHNKIREAELRQL

RKSNTDYEEQNAILQKHIESMKSTVDKLESETSQQRSNNAALQQHIETLRKTLAKAFGSL

PLPGTQETPTEESIDSYMVQLHSVIVNNPQQHQSLIQTVRGIVSQLALT*

>maker-20516_pilon_pilon_pilon-augustus-gene-0.24-mRNA-1-DNA_novel01

MSGRGKGRGRGRGRGSTSTSDEGFWGKPNSRPEAQLRQNDLGNSAKHPAGLEEDRKVWER

KFADACQEIQKNVDKYIATHQLDNVDSDEENDDDDLEEDGIIEKLYQGYNGERSSSRTDQ

FLKDACKTGAQVCLICIDNIKHADSVWSCKECFCGFHMSCVQKWAKDSIYQLAEAHLESD

PHFNKSTLRWCCPKCRTEYAQSDIPSRYYCYCGKAEDPKFDPWLVPHSCGETCRKLLKPE

CGHRCFLLCHPGPCPPCPSMVKSKCYCGRQPAQTRRCSAKAWSCGKPCGGLLSCGQHTCE

EPCHADECKPCPRQSLQPCMCGNEKKKRFCADPTWQCDKVCGKTYSCSHHECKKVCHSGD

CGKCPSSGPRTCPCGKASHILPCTEKIPSCGDTCGKQLACGIHYCVDRCHKGSCAACLQF

CVKKCRCGAREKEMACSKEFTCEIKCKNIKDCRRHNCNRKCCEGNCPPCEHLCGRTLSCR

NHKCKSRCHPGPCYPCSLTHTISCSCKATTITVPCGREKNTPPPKCSKLCRKPPACHHPE

RAKHKCHSGECPQCRMMCSLKLNCGHICPAPCHDAVPVKIVSHKKRAGPWEPVEGPRIEI

QKKPCPPCQVPVPTTCFGIHETVNWPCCDIRPHSCGRKCGRLLACTNHTCARECHVVEGA

VNDKDAGKNCAPCEELCSKPRPDGCSHVCGKPCHHGKCSFCKQLVKVKCHCGINNLYIEC

HTLNSAATDEKEVLCSCKNHCNKSISCSHRCMKECHSGPCSDPTSCRKRVAIRCPCRRIR

RDALCHLVISGQIIVECDEVCLEKIKALEEEKRREEEELKRKELESLEKLNEGRKHKKKN

RKHVEAEVAESQWTINKWMMLGIFILIFNVFIYFIYNFDFN*

>maker-4754_pilon_pilon_pilon-augustus-gene-0.39-mRNA-1-DNA_novel01

MSSSAETVREAALYLEYFLKGLSECNVVGSYTVQFLELRSFNVRLEASLADHLKCLVINM

KGLLMKLDSSFIPYCNTGAQTEDILLCSLCNSHSTIYSSPEKDSKKSFEEVEKTCESPEI

DQLLTSDPLIQLKQEIPCLNAATSRSVQYSRKENIANIIHIDSTDEKDPLALVEHDTSPG

IKTEKCSVALSLTALDDNLNIGYATAPQMEEGQGEILLDHQDGSDSTHSEGLMYSIYTMN

SCGGLQLVSRVLSKNNSSKDGLIEPVEKAMIEINNLKEELYSPNNLAALALQENNNQDQG

SSLTEEGIAITDSSLGNPAKPYSKIISKIEKPARLKQIKKVEGMDTCIPKFSEIVKLGPE

DLGNGTWCNVCEKEFPSVGSLKAHVSRTHSKVKWCNTCSKFITSEDMNSHVQDYHSELPY

VCNVCGQSLRTSGAFQRHMDIHKGLRGSACEVCGQTFSRTEYYREHIRIHTGEKPFKCET

CEKTFSRSSNLYAHMRIHNGEEQRHMCKLCLKSFARADKLKEHTIRHLQVKRFACRLCSK

SYRERRDLIKHLGKIHASDRSSNTD*

>augustus-55760_pilon_pilon_pilon-processed-gene-0.1-mRNA-1-DNA_novel01

MVTEGKITGLVIEDSVSVLNQRLGQLNQLRKSRHFCDVVLQVGSSEIHAHRSVLACASPY

FFELFTAEDDRKTAREGKLLYKLNGGFERDSVECLVHYAYTGCLDVQHPLVKSVFIAANR

LKMETVSHACGEYLVAHLTPESCLGIRAIKGIASNAKLAERIDEYIQQQSDLVLVTRDAM

GVPKLQVSVIHITQDEANITGRALCNLVLEWLKKQVIEEDLQLELLKEKKHMLYLNMDNS

LHDCTDIQSGELNDSDIVQDYKKMSRKLSQTNIKVRRKSTTPQPVKPRLMLYSRSISDKD

DSEQDTEWKLIAYSQVADGAWVAVITLKGSVAVLSVQQKMGNASPTSTPMSSRPSSVEKV

DFYTVIPHMASPKCATGTGNLNGRLLVCGGYDRGECLRTVETYSPETNIWTAMPAMRQGR

GRFDLTVLDGKAYAVGGCDGSKELSNVEVLDETASKWISVAPLPLARSNTGQSSIMESQL

AKCGQETGTESFFVLAMIICHLNKLQADLLANFADVHVPIS*

>augustus-55760_pilon_pilon_pilon-processed-gene-0.1-mRNA-1-DNA_novel02

MVTEGKITGLVIEDSVSVLNQRLGQLNQLRKSRHFCDVVLQVGSSEIHAHRSVLACASPY

FFELFTAEDDRKTAREGKLLYKLNGGFERDSVECLVHYAYTGCLDVQHPLVKSVFIAANR

LKMETVSHACGEYLVAHLTPESCLGIRAIKGIASNAKLAERIDEYIQQQSDLVLVTRDAM

GVPKLQVSVIHITQDEANITGRALCNLVLEWLKKQVIEEDLQLELLKEKKHMLYLNMDNS

LHDCTDIQSGELNDSDIVQDYKKMSRKLSQTNIKVRRKSTTPQPVKPRLMLYSRSISDKD

DSEQDTEWKLIAYSQVADGAWVAVITLKGSVAVLSVQQKMGNASPTSTPMSSRPSSVEKV

DFYTVIPHMASPKCATGTGNLNGRLLVCGGYDRGECLRTVETYSPETNIWTAMPAMRQGR

GRFDLTVLDGKAYAVGGCDGSKELSNVEVLDETASKWISVAPLPLARSNTGVCSVDGRVF

CIGGWNGQYGIKQCDVYDPKLNQWTTIAPLHVGRYQAGVASLAGKVYAIGGCDSWNCLNS

VEVYDPETDMWRFVAPMSTPRRGCGAEVFKGKLYVVGGSDGTQSLCSTEIYDPETNAWMP

GPPMTTCRANVGVAVVKNKLYAAGGFSGKNFLNSAEYLDPDTDEWTNFTPKPELLKSIQD

KGQSITGNEHSKTSNGLNHCDSEEVFERDTITTHLKMENGCNGITNGIHPIAANGQSL*

>maker-20720_pilon_pilon_pilon-augustus-gene-0.23-mRNA-1-DNA_novel01

MNRNANKSLKRKVIEYQDGEIDEDGRASKEAKRDTSNSSISSSFSTLALRRLVPEDWNPS

PISRKQDEENEDSFEKTGDRTVDRGTLWSGKDVCQEPQGISQLYMACNRSVKKSSSCDCE

TQTYYFVPNSHDTRNEVSKDKGLVRECCVIGCKYNSEEDSSVSYYGLPSSKAERSLWADT

LGLKFASASKGEIQEICSRHFDASISPQSSLPKVSHTPKSKTRKKKTCDILIAEEAEKVK

QSMATGRAKRTPKPNRNYDWAELMPLIKSETEDYEMEAAMSKNPGKRRKQLLDYIKKGKK

GNERSFMEMHVNLDDEVEESDSESDEYDVGNISLPSLPRPPPPKGKGRPHRIQDTCTQTE

SSLNSRGPLREVKVQCNLTAEPLVSCSSCSSIDWRSLDEELSQIMSVIECEIVNPILSRC

LSNALSEHAIPSHAANVLKMIAGLPRDYLEDRKDIHIVPGEDEFDEVEIEKFIHQEEGPI

VGIREIIAKDWNKKAKTVRTTASKRDEDILDDPDSFGLKEATDKDDDEFNPEGAAVEEDD

DDYEEDDDWDIKKDDSGASIQKKKTRLKAKKELKGEMAAVSCRYCRKLFLNKEQFLAHGC

KGQRTDKGRKFVCTICGRSFAQKIGLQYHNVYIHNGERKAACPHCNYWAPDKAKLSLHLR

KHSKIRPFVCEFCNASFKFRSTYKNHVARHTNSGKHVCTQCNKTFLILSELQEHSQIHSL

KRPYVCSQCGMAFKQKKRLRVHLRAVHYQDKHFACNICGSTHINNWNLQIHMKTHSYVNI

GYPNVCIYCGSTFRGKAGLAAHMRMRHPMVLVGVSLDNEELIEHILDPSPPVDPLMVEVF

SCSLCGLLFESLDLLTDHTTTEHEVFIYSSAQ*

>maker-20720_pilon_pilon_pilon-augustus-gene-0.23-mRNA-1-DNA_novel02

MNRNANKSLKRKVIEYQDGEIDEDGRASKEAKRDTSNSSISSSFSTLALRRLVPEDWNPS

PISRKQDEENEDSFEKTGDRTVDRGTLWSGKDVCQEPQGISQLYMACNRSVKKSSSCDCE

TQTYYFVPNSHDTRNEVSKDKGLVRECCVIGCKYNSEEDSSVSYYGLPSSKAERSLWADT

LGLKFASASKGEIQEICSRHFDASISPQSSLPKVSHTPKSKTRKKKTCDILIAEEAEKVK

QSMATGRAKRTPKPNRNYDWAELMPLIKSETEDYEMEAAMSKNPGKRRKQLLDYIKKGKK

GNERSFMEMHVNLDDEVEESDSESDEYDVGNISLPSLPRPPPPKGKGRPHRIQDTCTQTE

SSLNSRGPLREVKVQCNLTAEPLVSCSSCSSIDWRSLDEELSQIMSVIECEIVNPILSRC

LSNALSEHAIPSHAANVLKMIAGLPRDYLEDRKDIHIVPGEDEFDEVEIEKFIHQEEGPI

VGIREIIAKDWNKKAKTVRTTASKRDEDILDDPDSFGLKEATDKDDDEFNPEGAAVEEDD

DDYEEDDDWDIKKDDSGASIQKKKTRLKAKKEHTLKAGSEQGSSQAVEKKKEGTVMVSGT

SNKTKIQQNRRRRRKAGDTRWIRKEHRCTDCNIVFSTQRKYNLHYSHVHLGVAPWQGHHK

CDDCGKVFTQKVTLKVHRMFKHGAPRRFRCTKCVYEGPTKEYLKRHMKVHSNERHYVCSA

CGKGLKTLDSYRNHMVLHTNEGRFFCTLCKKAFNHKGAYTDHMHCHEESRNYACYCGASF

KVYKHVARHIRAVHLNDKRFICDICGTQHMTGFNLKNHLKKHGDVPYLPYSYECSTCEAK

FRGHQGLATHLRVIHNSKIVNISPEEIKPTEPVHPTRRPTKYRYSLLSDDSTVMSNDSAA

EDNRKLLVACEEGYILSEIKAEEGEEVVYNVSAREVDLNQEVDYEFVSGNDEREVIHIFS

CNICSSMFSSESLCQEHMAKVHLSE*

>maker-25210_pilon_pilon_pilon-augustus-gene-0.14-mRNA-1-DNA_novel01

MLTQLELLNASGASSNDDDSGMSLSPLDGLSEYSIVKTEEFEYFESNLGSDSYYDISPTQ

ELQDSLPPPPCLTPTAGTAPSAFGSATLYDTSISSRGDGFRGHDDDVKVFDEDLKPLNEA

LESSSDFSSGLYDGNVELTDVKIKIEEETSTYDAFSTDVYDNSIATEGFNLGSWYDTFDA

SLHASETTETKATWDEAASKGKIGPLQRRALDSIFNVTEKPSRGLVLHIASELGLHHITV

KNFFSNGRRRLRRAAARLNDPERTRRENERRKEKRRLAARASAAALSNGDTNKSCSGSGS

SSSSSSSSSAGSLAATKSSMPTTVESPPPKETTFVSLERRALMEKLADKVQRSKAQRSLT

SDPGVMQSTTSSTSTTSQDFLSQSTQDLLIHPISDILSQASQDILSHSSQGLLAELPHDF

LAEPTHDLSQTSSLLNPNTDPWNFF*

>maker-31912_pilon_pilon_pilon-augustus-gene-0.5-mRNA-1-DNA_novel01

MGSGMGACPQGAGVSSGLGAAPPATSVGALSVAPPALSPQDYGGDCSSSVSSPGSPDPHS

PLQVTEPESARASQGRDVAAGRGLNEKFLGSASSARATALPTAAAGDLKSEVRFDYHISP

NDLALATVPGQDFDPRTRCFSEEELKPQPMIKKSRKQFVPPEMKDEKYWARRRKNNLAAK

RSRDARRLKENQIAMRANYLEKENNAMTVELQKAQAIVESLKKRLAMYEAV*

>maker-46212_pilon_pilon_pilon-augustus-gene-1.13-mRNA-1-DNA_novel01

MLRLDSATKEKALQTMSSMSSAQIVSASVMHNKGSLPLPPTPVSYPSPFWQGGIQAGTSQ

DVKPFAQSPFGMGGETKPGAGGMSGLLQPAAPSITVPPWEGRSIATQKLRLVEFSAFMES

QRDPDTYQKHLFVHIGGPTSYTDPILEAVDIRQIYDKFPEKKGGLKELYDKGPQNAFFLV

KFWADLNVNIQDESGAFYGVTSQYESPDNMTITSSTKVCSFGKQVVEKVETEYARFENGR

FVYRIHRSPMCEYMINFIHKLKHLPEKYMMNSVLENFTILQVVTNRDTQETLLCVAYVFE

VSTSEHGAQHHIYRLVKD*

>maker-46212_pilon_pilon_pilon-augustus-gene-1.13-mRNA-1-DNA_novel02

MLRLDSATKEKALQTMSSMSSAQIVSASVMHNKGSLPLPPTPVSYPSPFWQGGIQAGTSQ

DVKPFAQSPFGMGGETKPGAGGMSGLLQPAAPSITVPPWEGRSIATQKLRLVEFSAFMES

QRDPDTYQKHLFVHIGGPTSYTDPILEAVDIRQIYDKFPEKKGGLKELYDKGPQNAFFLV

KFWADLNVNIQDESGAFYGVTSQYESPDNMTITSSTKVCSFGKQVVEKVETEYARFENGR

FVYRIHRSPMCEYMINFIHKLKHLPEKYMMNSVLENFTILQVVTNRDTQETLLCVAYVFE

VSTSEHGAQHHIYRLVKD*

>maker-46212_pilon_pilon_pilon-augustus-gene-1.13-mRNA-1-DNA_novel03

MLRLDSATKEKALQTMSSMSSAQIVSASVMHNKGSLPLPPTPVSYPSPFWQGGIQAGTSQ

DVKPFAQSPFGMGGETKPGAGGMSGLLQPAAPSITVPPWEGRSIATQKLRLVEFSAFMES

QRDPDTYQKHLFVHIGGPTSYTDPILEAVDIRQIYDKFPEKKGGLKELYDKGPQNAFFLV

KFWADLNVNIQDESGAFYGVTSQYESPDNMTITSSTKVCSFGKQVVEKVETEYARFENGR

FVYRIHRSPMCEYMINFIHKLKHLPEKYMMNSVLENFTILQVVTNRDTQETLLCVAYVFE

VSTSEHGAQHHIYRLVKD*

>maker-47178_pilon_pilon_pilon-augustus-gene-0.7-mRNA-1-DNA_novel01

MSKNQGEATCGTNTEKSSNNSSSSGAATTTTSGPGTSSSSSSSSQHSKMGVENKLSSSSR

EMRNMAEKMRRDKLNNYVAEIAGIVPLGSGASANKRIDKTSVLRLAANYIRMHKILNDDD

EPSNKMTPDFGKEITHALAEAVGGFLLVVTSSGKVVYITETVDQFFGHTQVDLLGHSIYN

VIHPDDHDIFQQQLVPKENCRRSFYCRMMEKALSRNDPGRYEIILVVGQLRPLPQMQTCL

PSCSMTSPASSSHGETSTSSDHDDEESSDNEGEMRPLKVTNRTGTHMLVCFVRVVKDRPI

TELTLVESTQDEYITRHGMDGKILYTDHRISFVTGLMPTEVVGTSAFNYMHQDDILWSIV

AQKLMFTSTQGQGVVSYRLRCRDGAHITLRSRGFLEVNKQTGQVESFVCINTVLSINEAM

EEIKNQRRKLLPIITCHETEDHLGSISSSLPPELMMMLKQVMNPETMRKMIESVSSNQLA

PPESYESAKAPSISPLPENAEHDDTINPFHTKPKRHCSEISTCKSVDTWISQKGEDQEEW

HKVTKKRSNVDEFITVASKKFSFQPDSPCPDLLHPNYKSPSNQNYSSVTSPQPSFSPTTS

SVPENLNNFPSGTCHQYSSNIHNCDSSSHSHSGDSVIPPSMSQQYLKVDAVPPEELWQPI

NPSALTPQRSGQDYLIPEDEHKTSKNMMPSPSRNNPRTPSGIANSPNFEQSRDAVFSPGL

RDSRITDNEHPLNNHHQRIEGFSPPGFDSLSGTGGTAVGCNVLQLRCETVSLPSGQNHGI

GSYNQSTSLGISYPSSYSQNTCQVSPPVRNYECDEKKLIHGPQDTDQSACKVISSTAPSH

CSYQFSASSLKDHSSLMSPKLYPQYQPSTPGINYSVSQHQYQYTIPAQDQGYTVPPQTLS

STSSGYYTPDFSPQVQKQQQGLQGRQLPPNQDSSLHAVPITTSYSVSLSEPGTSGKLL*

>maker-47178_pilon_pilon_pilon-augustus-gene-0.7-mRNA-1-DNA_novel02

MMMLKQVMNPETMRKMIESVSSNQLAPPESYESAKAPSISPLPENAEHDDTINPFHTKPK

RHCSEISTCKSVDTWISQKGEDQEEWHKVTKKRSNVDEFITVASKKFSFQPDSPCPDLLH

PNYKSPSNQNYSSVTSPQPSFSPTTSSVPENLNNFPSGTCHQYSSNIHNCDSSSHSHSGD

SVIPPSMSQQYLKVDAVPPEELWQPINPSALTPQRSGQDYLIPEDEHKTSKNMMPSPSRN

NPRTPSGIANSPNFEQSRDAVFSPGLRDSRITDNEHPLNNHHQRIEGFSPPGFDSLSGTG

GTAVGCNVLQLRCETVSLPSGQNHGIGSYNQSTSLGISYPSSYSQNTCQVSPPVRNYECD

EKKLIHGPQDTDQSACKVISSTAPSHCSYQFSASSLKDHSSLMSPKLYPQYQPSTPGINY

SVSQHQYQYTIPAQDQGYTVPPQTLSSTSSGYYTPDFSPQVQKQQQGLQGRQLPPNQDSS

LHAVPITTSYSVSLSEPGTSGKLL*

>maker-47178_pilon_pilon_pilon-augustus-gene-0.7-mRNA-1-DNA_novel03

MMEKALSRNDPGRYEIILVVGQLRPLPQMQTCLPSCSMTSPASSSHGETSTSSDHDDEES

SDNEGEMRPLKVTNRTGTHMLVCFVRVVKDRPITELTLVESTQDEYITRHGMDGKILYTD

HRISFVTGLMPTEVVGTSAFNYMHQDDILWSIVAQKLMFTSTQGQGVVSYRLRCRDGAHI

TLRSRGFLEVNKQTGQVESFVCINTVLSINEAMEEIKNQRRKLLPIITCHETEDHLGSIS

SSLPPELMMMLKQVMNPETMRKMIESVSSNQLAPPESYESAKAPSISPLPENAEHDDTIN

PFHTKPKRHCSEISTCKSVDTWISQKGEDQEEWHKVTKKRSNVDEFITVASKKFSFQPDS

PCPDLLHPNYKSPSNQNYSSVTSPQPSFSPTTSSVPENLNNFPSGTCHQYSSNIHNCDSS

SHSHSGDSVIPPSMSQQYLKVDAVPPEELWQPINPSALTPQRSGQDYLIPEDEHKTSKNM

MPSPSRNNPRTPSGIANSPNFEQSRDAVFSPGLRDSRITDNEHPLNNHHQRIEGFSPPGF

DSLSGTGGTAVGCNVLQLRCETVSLPSGQNHGIGSYNQSTSLGISYPSSYSQNTCQVSPP

VRNYECDEKKLIHGPQDTDQSACKVISSTAPSHCSYQFSASSLKDHSSLMSPKLYPQYQP

STPGINYSVSQHQYQYTIPAQDQGYTVPPQTLSSTSSGYYTPDFSPQVQKQQQGLQGRQL

PPNQDSSLHAVPITTSYSVSLSEPGTSGKLL*

>maker-71460_pilon_pilon_pilon-augustus-gene-0.14-mRNA-1-DNA_novel01

MQSYMQLSKPPSMQSYMHSSRRNKIDCDSGEDEIHSEMQVIKSEGNHRAYKAKPKEDGIS

QASPEDRRKILGKGVREIICLRAPTPTDIDLPSSREVESDKVLLQDFSCNKIISFLPTYE

ILPDFSQRKYLESSSDLGVQNNVVQKDSMQSQQLLDMQEGLQKAMTNSNIIRKRKKAPIS

YRKDKKNNESIISRTRKISQNNNDEEDGKGHHHLRRPQHKVLGFMEDHAEFTSDWEEASK

YQGRYVSSVLESTVDGSIIKGARRVTGSRRMTPAEFTSCIESAIETGDVDEDPLKALDEW

GLKTELHKYKCSLCEMVSDHRIDVEQHIDELKGACHSDAVVEVLLGSKAQGRNCTLCNVR

LGRTEYFTHIREEHKEVFPLHCGYCAFQGQDYNQLRHHIQKKHKTSDFDIIDMTLSQEAS

DYLEQKDSVNKCEMVNEEEAEEEGTAELPFTCPLCEKSLKTLRNLKKHVMQHSRQKKQCI

LCPFTTNFPTQIRSHYLRMHPNETPKWKKVVVAEGGSDALVEDASPFLKMVSVKYGVRKP

KEKNWVRQYRCPHCSYTCNFNSSYNRHLRVHNGVKPFKCGYCSFHGREAYVVKRHCIKAH

KGKDILVENGQDFKAPYRYAKRIKKEAVEWDESCQVVEKGANNLASQSSNDLSLNIKVEV

QSEELSKSNTLKESSDSQIQFKDGDKKYSENKAAENSDGSDLYQTKDSTVYQCLVLSSGK

NKDDCTEKPVINQNDSASCFSVYSRDDCKKNIEKPLDDECQIPLENSKKDTINISPLLGP

VVKGSSFIHTQECATSKVEKCFPELENVRNLPEGYSSAQHQMIHTIKVKTFRGNTESSIA

PLKEKEDIFEEGNPSHMQIHCDNDRTLSTIDFSSSYVIDPSADSFPNSVFEAVVINLYAN

ESYTPRELITHNSTERLEDGCVFNEKICTISDHEDSPLNLIVSDFEVQLSDRRKTFCNPE

ESSKPNPVQLVKSSEKCVKVSGESLDGISFTIEKSSDLPPEGLENTLHNHITFLSDSLNQ

RVSNRISAPPGVMTRDLLCLDYSNSDYFSSEKVRTMMIKKKGGLVMCAECGVTTKYRAFY

KHAKKHFNIKPFRCGYCLYRSIEKSKIRVHNAFCHPSKPCVILKLSPDSAKVSDATHHSL

SSNTSDVSASKHILEGENSLSENGEIYPSLLKSTKTSEHDN

>maker-940_pilon_pilon_pilon-augustus-gene-0.36-mRNA-1-DNA_novel01

MERLHIPYPGKFHRINQMGLVDHQQVAQAHPGSFISFQIQEHPNPGPKEQIPLQIPTPNA

VQNAIQTTIQNAIQAPVQTMHTATNTVQAAVQTQQQNQQVQTTPVIPEKPYECNRCSKRY

AQKSHLTSHMIWHDREKNFVCKICNKRFSMESHLNGHMLEHSREKKFECEVCGKRFSMEN

YLNSHMHVHISEKKFQCEVCDKRFAMQNHLDSHMLVHCTEKKFECGICRKRYTQRSHLNS

HMLKHSKERNFECMLCGKRFQMECHLKSHMLVHSDEKKFECVLCGKRFHMEVQLNSHMVV

HMSEKKFECSICDKKFIMESHLNSHMLIHNEKTFECELCHKRFNMESHLVSHMLVHNEKT

FACEICGKKFNMLSHLNSHVMVHSMEKNFECTVCEKRFAMENHLNSHMLVHSEDKKFACQ

ICEKRFILEIHLTSHMLVHSEEKNFECEICNKRFNMESHLNSHMLVHNEKTFECMLCGKR

FLMESHLKSHMLVHGEKKFQCGLCSKRFHMESHLNSHMLMHSEEKNFGCPMCGKRFNMES

HLNSHMLMHSTEKKFECNVCGKRFNMECHLNSHMLVHNEKKFVCGLCGKRFNMESHLNSH

MQVHGDKNFECDICGKRFGQSSHLNAHRFVHTEQKKYECQVCRKKFAQESHLGAHMFVHS

DEKKYECETCGKRFIQEMHLNNHRFTHNTEGKIEYQQWGNRIAAKNHANMLNFLHQDDKV

LECEECGKKFAHESHLVSHRFVHKEEKRFECDQCGKRFSQESYLCSHKIVHSEKKYECNQ

CGKKFVQESHLNSHRFVHSEKIEVEYGKEIIRDLPNLQNGQIIVQEMEKKPFEDHNAQLV

TDDKKQYQYEQNAKLLIQQTNQSPYAYAQWEHVRTLWSL*

>maker-9718_pilon_pilon_pilon-augustus-gene-0.31-mRNA-1-DNA_novel01

MEGLTLDVTNLGEAGAQMITLVEATALESPQHNVHILTHQLERDDGNPLHEEHQDHDPLD

PDPKLPLGNSSPHCLVCNAKLGVSARGAMELFSEKAKTSHRQIEVHVLLSNIVNQEIQHK

CIHSSIVCKKCYKLIDDIDSLEGQLISMKQVVTNKYMRTLAIVKQDTLQGEIENVLDEDA

LHLEASTLTKDDRDFKVYMGSGVNGRGRRGRRGRGRGRAPVVKLEVKQEEPVELIHELTA

TDNVESLKQQGLLASTDSNDGLGGRIEDDIENDGGTDILMVEEEILEESAVVEDVMEVDG

LDLEDNEGCVQIGGLTLSTLNSHELNVNHDNSAFQEEQHLLEDSIEKYKCRFCSLKMNVL

ADIQKHMREAHPDRLYECEVCQERLPTKAELVNHLEQHMASGEKPYECTMCPRRYALPRQ

LKEHVRHHMSKTYSCSRCPRRFRSATALQEHFNGHTGNRPHACDQCDKKFTSRHILKTHM

KTHGVRQRPYQCRACGKHFLTSHHLTDHMNLHQGKKNFICETCGKAFATQRNLDLHAITH

TGVKNFACSICNKMFARKGEVEDHERTHTGEKPFQCEICGSTFSQRSNLQSHKRTTHYQE

KRYQCSRCSKAFKRKRLLVYHVMSVHTGERPYKCEQCNAGFVYPEHYKKHLRIHTGEKPF

RCDICGKNFNSRDNRNAHKFIHSDKKPYECTLCGAGFMRKPMLASHLQQHGHTENIEAYI

KVNPPTIVANESGVSSVSSPVGSVRTVKLDDDQSLDPSLDGSVQLVRSTVRDGETVEVMS

RPVHIIDADDLPRYIIHAANSDRPEEGVGHFFASLQGQVVEVRADDIERYSDLTADQMTQ

VAQVAAQVVSSQSAGTSIQQVAVSGDIRPFHIQLEPATRDITLQTQNGQAREVTATIGLP

TATTVSTRDGTLSTGNHGTAVLVGSRQMHYDSGALTQSPLRKVLTEKDDIRTTTVLNITT

NSETRSAVFRPWPQHTIDTNSSNFIGN*

>augustus-6192_pilon_pilon_pilon-processed-gene-1.4-mRNA-1-DNA_novel01

MELLREKREPRLLDTASSSRSTTSFLIDDILFHRPKRVGTNDSGNEEGLVGEDSHWMRLN

APLYPVTSRDLTSVSSSIVRASFTESYAAAAAAAAGVLPSTAAAAAAAAAAYLHPHPYLH

KPEAHFLFPGASLGFGSLFGGSEGALKACRRRKARTVFSDHQLGGLEKRFAAQRYLSTPE

RVELATALNLSETQVKTWFQNRRMKHKKQLRKHSEDKTPGSSSSSSSSSSSSGSEGEAHL

AGQAQQAHGQQQLSRSMCPSSPMTGVASPCTTTTARDSDISHSDYEDEIDIVGVPADLRH

GHAAHAHSQPPH*

>maker-10196_pilon_pilon_pilon-augustus-gene-0.3-mRNA-1-DNA_novel01

MVKKLKIDDSDQGQQQPFQQQQQQQQQQQQQQQQQPYLQQQQQLQQTVPTQGPKTNFEDN

LITNQGASGNTIIQQDLFQTLLTSTNDVTSRFQNSSISTSQQFIPPCQQQSAQNNMTNIS

STFSNSAASLSSANHFLTMKTAQYVPGPPLHHPSTNAAPNSNSYSLETTQSALTANQSNN

MYNHCDQPSTSASNLAPTYSHNLPVTSALPTSFQSKGSVAIYKPPEGGAPPSIRDSMYSS

RMTTYQVGNEWAVPKSYTENEGGNRSDRGHTDIDELIQGKGIEPSRGPPVAQFPQRNSFI

GIYGLHVIFPGESPKLVLKEDAYGNRHLFTELDHCITTVVRHSADYSAAMRFLIVFSKDC

EATQPVFPCKNHQKPEHPNHMLEVRSGSGTTIWEEEPHPSIVVPPSPSSEGKYSVQLKFL

CRNSCIMRKNLTLICQLEREGQVVGRQCLELKISACPRRDAKVSKNKEVPEIDTVDGAMG

SEQVQLGGILTPNDTLSTNTSVLSTNTSVFSIVTQSINQSINEAVSSALESGRRAAYFAY

MEKRFQELQPEDYASAKADFDRWWDSPKDVI*

>maker-1054_pilon_pilon_pilon-augustus-gene-1.16-mRNA-1-DNA_novel01

MKTGTLETRGELPTFSMTRKNITNQSGTWSRHISPKKNHLQESSVLKENPLSLVMDSILT

GTDLMSDLNTPDIKFEDFDFHSGNPTSSYNRGNVNVESSRDAMEPSVGVVCNQDDDSLLV

EAPTTGVAYTLFDMSPGSRGLLEALSPSTQEDLHADQQEQRINLEEYQESASTLNVRSDV

ITYNDGIFTNPIPELDHCMQETLIPVEDCPQPAQMEMLVTHNNPPQLSISEGDLEFSIPQ

NLLEQMESLFPEEQAIESSLIQSSNVCTTELDMFLGQQDFNSDLLSESLEQSGLLDDELS

SFVCEDTCNDDFSNIHINSLMPNSDAVQSSIAPQVVQPNTLDLEPVGLPAEVAERSTFLL

TKDFLSTPVTIHPAVSATFNNENDFKTTFIVNASSTGAVPKTPVKKRQHQEDTTPVINPV

KRQRRASRKPLRFRELYTDEDFTDSGSSESFADQESRLWTDPSDIDIKNVNVDVASSSNV

SARRRSRGSVEELTEEQKYHRIRQLNNEASKRCRLKRKLTVKQMEAQMEELVKRNLVLRE

KHEALKQLKDKFQGFVSDYFKTRLMEKSTNK*

>maker-11076_pilon_pilon_pilon-augustus-gene-1.5-mRNA-1-DNA_novel01

MASNMTDHSGLKSEKSRGSSNVINVTTSETMRLDGVGDRTNLHIQPVGGGTGGGGGQGYQ

QKKKNSSFQITSITSRLSNDPGDDSADDTEDISDMGDVSRVTDYDQETPSYSEDYSKTEE

VFFGPAPVIPTSSQYGITALVQQTGTGGVMTARLPQGVTVNVTEGGIALAKADGDSDDVD

HWQNRFKIVKIDSNEPFKRGRWICLDFMDSPAVSATSTQKKDESMTVVINSVGEKDDPAD

HQPQTVLQVPVPVTQQSQTTTQNQSHPATAQYQVPHSSQAAVPIASSAQPTQGSVPLQQS

SPHMTAPATTQGPGNMQYPSQGAPMQGQLQPTMSQGYANATQAPQASVHPSPSAQTPPQA

QPPLQPQLQQQQQQTAPSLQQATIQQPPASQSGVQAPMQATSIPQGGVTQATVQQSMVPS

NSHAQPQHQQPVPTQSTMAQPSINTQSIPQQGMATQPVSIPQSVAQPSVMGHAGIPAQLI

SLM*

>maker-11076_pilon_pilon_pilon-augustus-gene-1.5-mRNA-1-DNA_novel02

MGMMGLGGHGDVSGSGEGFVGGGGGNVGGGGGGRPRRRRRTPPKAKIDINDNSLYSVYCI

FKSILSASTASTVAIDNKIEQAMDLVKSHLMFAVREEVEVLKEKISELMERINQLEYENT

VLRQYATQEALQQLQQPHHSSNT*

>maker-11076_pilon_pilon_pilon-augustus-gene-1.5-mRNA-1-DNA_novel03

SPLAGVEESGSTFLSSLTRSHRTLVSLTLRRPSEGKMLCSDIYDYQTYANALVKSIVVDS

ASTASTVAIDNKIEQAMDLVKSHLMFAVREEVEVLKEKISELMERINQLEYENTVLRQYA

TQEALQQLQQPHHSSNT*

>maker-11076_pilon_pilon_pilon-augustus-gene-1.5-mRNA-1-DNA_novel04

MQMDALHCGYPHASAGMGGGVPITPPATPTQEMSRTPTFSTLSSCSESDYSKHEYAPTDY

ADNQYSSYSTEEYTPTHSLSPPESPMTTPKSTPTHTPTFSPMSSNPSPTSTSSSSSSSSH

AFKKPTSKINRNRKNSFVYDVRLTDEQFLFFVPDFDLVKSHLMFAVREEVEVLKEKISEL

MERINQLEYENTVLRQYATQEALQQLQQPHHSSNT*

>maker-11076_pilon_pilon_pilon-augustus-gene-1.5-mRNA-1-DNA_novel05

MQMDALHCGYPHASAGMGGGVPITPPATPTQEMSRTPTFSTLSSCSESDYSKHEYAPTDY

ADNQYSSYSTEEYTPTHSLSPPESPMTTPKSTPTHTPTFSPMSSNPSPTSTSSSSSSSSH

AFKKPTSKINRNRKNSFVYDVRLTDEQFLFFVPDFDLVKSHLMFAVREEVEVLKEKISEL

MERINQLEYENTVLRQYATQEALQQLQQPHHSSNT*

>maker-13448_pilon_pilon_pilon-augustus-gene-0.5-mRNA-1-DNA_novel01

MLGDMVEAVVVIEEEEKKNVKRKEEVLRKVFDGGTSSYRCGLCNTYHSTAEELAVHKAEH

NQVLECVKCQKSFLSVKQLKRHMTVHVPCKDDEKQSNKGMNEHQCLLCGKVFGSESALSR

HSSTHARSEGRHQCKVCSRRIGTRAHLSEHMARVHKIHSDSKKMQCPMCDRHFVSSFHLN

LHLITHGEVKPLYSCKVCNRNYLRPLSLRRHMATHDQNFVCKTCNESFPSARRLSLHSRT

HDASYKSKVQCPKCPLAFPYKSQLEVHLRTHTGEKPYVCETCGKSFKRLQHCTVHLRVMH

GNEKQPCKECGKLFSDKANLLRHRLKVHYHLNRWVCGVCAQSFAYSEDLRRHLKKKHNLI

FKRLVSTNKKCMSEVYVIPELGTREISANTRAAIECICEMEKQKVKQIFSGAFPDLTKPL

PKPVVNDTDRNDDPDNSAPQPGDTVAAGSVCIPSATVGLPTIISSGQLAAGGQSSMNFQT

CYGTGEIPGLVSLPVTGVKCTECGVDVLTPQQCGICGVLLCTQTHLDSHIASVHQVLFQC

SVCGQHYSSQSECIAHVTNCHSSHFLTVQGTAATYLPASQTQTLQSTGAFVQPQLFLPFP

NSQSSPQCILQVGSCPPQLPGLVAPAGNAVGSQQLSAPNCVLPYTNVVNGWSQATALGSA

SGIGKQTVYAMTNIFSDQQSVPNIQSAVSHIIKKEPVAETQVCTITLPHGNPESLSTHSE

GRLSAKPCSSVLPTILPRTNSSGEEPQFIVGNSEPSEHQDKNSQMLLVNSRTNHSSGGDV

THPSLLHERVLEGKLSHHHTNTSVASVLLTLAETPQDDQTSMLVPGNALRIPDAHVDQVL

SPSIISDMSLDEVTKDSTITTGNTGFLQTIDLPEQPKDELVHNPSFGLSQVNEESTDCKS

PEAISVEIIQPQLLRPKSELTQKSLPKKSSSRTKRKKVISSEENETCECKICGKILNSSS

QLKRHKTTHGVRRFKCQVCDKAFTEKYNLKIHTLTHTQERPHECNLCQKKFRYLRDLAEH

KRTHEGTRPHVCDVCQKTFVRQRDLTRHQREQHDAKRYQCKICGFYFKRLLYLKSVHMRI

HHPLLIEANDESKPKENAPAKPEAKQSHICKICGRNFARARYLTSHLRTHSKRASYVRCP

LCPRMFSTEQTLKVHKETFHDHEARSLGTVNENLEMEGDIQGEPIVANFDQPHTENLVIS

ESQDLSENATSLVISTQNGASHVSKHQMLLQSDSSAQPSYTTAGTSESLEVSLQSGRISH

FDVNIEECDPISYLNPSFST*

>maker-13608_pilon_pilon_pilon-augustus-gene-0.15-mRNA-1-DNA_novel01

MITMDIPPTPAPHQVVHAQTIMQQQPSPEESKKKLTGSYYFGENSTKGHGGVNQLGGVFV

NGRPLPDMVRQRIVELAHNGVRPCDISRQLRVSHGCVSKILSRYYETGSYKAGVIGGSKP

KVATPVVVEAIARYKRENPTMFAWEIRDRLLAESVCSQENVPSVSSINRIVRNKAAEKAK

HAMPGTLSPVSQATSVIAHAPPAPHETALQRAGSYSINGILGIPPHTDPNGNINKRKRDD

TDEHRDMNGHPEDDLKRQRTQYNTDPLYTNMIWSKQWPTLKNEDAKTLLPDLGSSVAGGA

SSPYSSVQSFVDHPSSYPSVSASSVSTDALYETMSMTQSNTNHVYSPPLATSLGTASVGS

MVDYTYSSPYTQYSSTYPTYGYGTGGLLSK*

>maker-13608_pilon_pilon_pilon-augustus-gene-0.15-mRNA-1-DNA_novel02

MITMDIPPTPAPHQVVHAQTIMQQQPSPEESKKKLTGSYYFGENSTKGHGGVNQLGGVFV

NGRPLPDMVRQRIVELAHNGVRPCDISRQLRVSHGCVSKILSRYYETGSYKAGVIGGSKP

KVATPVVVEAIARYKRENPTMFAWEIRDRLLAESVCSQENVPSVSSINRIVRNKAAEKAK

HAMPGTLSPVSQATSVIAHAPPAPHETALQRAGSYSINGILGIPPHTDPNGNINKRKRDD

TDEHRDMNGHPEDDLKRQRTQYNTDPLYTNMIWSKQWPTLKNEDAKTLLPDLGSSVAGGA

SSPYSSVQSFVDHPSSYPSVSASSVSTDALYETMSMTQSNTNHVYSPPLATSLGTASVGS

MVDYTYSSPYTQYSSTYPTYGYGTGGLLSK*

>maker-13608_pilon_pilon_pilon-augustus-gene-0.15-mRNA-1-DNA_novel03

MITMDIPPTPAPHQVVHAQTIMQQQPSPEESKKKLTGSYYFGENSTKGHGGVNQLGGVFV

NGRPLPDMVRQRIVELAHNGVRPCDISRQLRVSHGCVSKILSRYYETGSYKAGVIGGSKP

KVATPVVVEAIARYKRENPTMFAWEIRDRLLAESVCSQENVPSVSSINRIVRNKAAEKAK

HAMPGTLSPVSQATSVIAHAPPAPHETALQRAGSYSINGILGIPPHTDPNGNINKRKRDD

TDEHRDMNGHPEDDLKRQRTQYNTDPLYTNMIWSKQWPTLKNEDAKTLLPDLGSSVAGGA

SSPYSSVQSFVDHPSSYPSVSASSVSTDALYETMSMTQSNTNHVYSPPLATSLGGGGSAY

TPMSTSQPTSNPGYATIDTQPTHYADQTPVSEGVTPLESLVVAPCSSPLKADTPCTTALT

VLQPASHAHAATHHAHVSTPVHTSTHAHVPMHAHVASTPDPTYTTLPPITHYTGTASVGS

MVDYTYSSPYTQYSSTYPTYGYGTGGLLSK*

>maker-1570_pilon_pilon_pilon-augustus-gene-0.13-mRNA-1-DNA_novel01

MGYIYKGNASLPDVEMAVMVYKLASKYQMSSLQSVCSKYLQSHVDLKNLALVYEMSFLYD

DSKLKQKCRKVVSESHEILSSPDVGQLSVQCMEDLLQQKLPVQSEVAVFEGLLQWGTLQS

ENSSTSAKCPKGLRQEIEMLLPHIRFLTMTLDDIVEHVMPSGVLTPEESTAIMMNIKSVG

NISLPAFCSPSRDTRVSYSQLHHIFLRMGTNVFEEPTIINPEWAPEKVDFGLFKNETSIG

KMVFMRNALLCAHF*

>maker-16102_pilon_pilon_pilon-augustus-gene-0.62-mRNA-1-DNA_novel01

MILGIPGPSALDFKARKISGKYPPPEQLAGPVMYSYQSRGRPRKNWTTATGLPTMIPITH

PPMNHSDDADDEESKPFKCNLCGKSFKLKGGLVQHERTHSSDRPYVCPDCGKLFRQPTHL

QQHLRIHTGEKPYECAFCDKTFRQRTILNQHLRIHTGEKPYACMECGKQFRQKAILDQHF

RTHLGDKPYACPHPECRKHFREMATLISHMKCHKDVPDPRIVLQQAKRLVKEERDDLIPG

NLNLNREQHGEGSREIVQDIMEQNRGMGLERVGQDRRLSMDSVTQERNMVQDRVGQERNE

QDQRNNRCGGSEQGRYQGSQEQNRQNHHSFPGHSSSEESSAQKSPHNVPQHSSGDQSNER

VPSHSQPQISPNMVPTPQMAMANLIPYPHSIFPATSYMMPPPDPRQYNPHSQAGSSHPRP

SQ*

>maker-16102_pilon_pilon_pilon-augustus-gene-0.62-mRNA-1-DNA_novel02

MILGIPGPSALDFKARKISGKYPPPEQLAGPVMYSYQSRGRPRKNWTTATGLPTMIPITH

PPMNHSDDADDEESKPFKCNLCGKSFKLKGGLVQHERTHSSDRPYVCPDCGKLFRQPTHL

QQHLRIHTGEKPYECAFCDKTFRQRTILNQHLRIHTGEKPYACMECGKQFRQKAILDQHF

RTHLGDKPYACPHPECRKHFREMATLISHMKCHKDVPDPRIVLQQAKRLVKEERDDLIPG

NLNLNREQHGEGSREIVQDIMEQNRGMGLERVGQDRRLSMDSVTQERNMVQDRVGQERNE

QDQRNNRCGGSEQGRYQGSQEQNRQNHHSFPGHSSSEESSAQKSPHNVPQHSSGDQSNER

VPSHSQPQISPNMVPTPQMAMANLIPYPHSIFPATSYMMPPPDPRQYNPHSQAGSSHPRP

SQ*

>maker-16102_pilon_pilon_pilon-augustus-gene-0.62-mRNA-1-DNA_novel03

MILGIPGPSALDFKARNVHEYFRISGKYPPPEQLAGPVMYSYQSRGRPRKNWTTATGLPT

MIPITHPPMNHSDDADDEESKPFKCNLCGKSFKLKGGLVQHERTHSSDRPYVCPDCGKLF

RQPTHLQQHLRIHTGEKPYECAFCDKTFRQRTILNQHLRIHTGEKPYACMECGKQFRQKA

ILDQHFRTHLGDKPYACPHPECRKHFREMATLISHMKCHKDVPDPRIVLQQAKRLVKEER

DDLIPGNLNLNREQHGEGSREIVQDIMEQNRGMGLERVGQDRRLSMDSVTQERNMVQDRV

GQERNEQDQRNNRCGGSEQGRYQGSQEQNRQNHHSFPGHSSSEESSAQKSPHNVPQHSSG

DQSNERVPSHSQPQISPNMVPTPQMAMANLIPYPHSIFPATSYMMPPPDPRQYNPHSQAG

SSHPRPSQ*

>maker-18230_pilon_pilon_pilon-augustus-gene-0.22-mRNA-1-DNA_novel01

MVRLGIATGSRGPSGENTGHRGVVSADQHLYPYPVIRTEGETHQSQQIILNPSQSSPLQL

RIDNQYENILPPDTRISPLRSRNEVSLSSTSVSPVASSSSSNIVNMTNKSHLSGWPRAVR

DSGESVIRSQKRTYRETEDTPEGHETYTKDVKWCPMCRSPVHPGVVHHCPATVQHGINFY

GLGGLKEKGYVPDHHGSSHTSSFSDNIHHSMSAVEEGLNFSNRVPGLRVPVVSVSPHSIS

PGTSNSQTIPQEDHSPSTCNPSVLKACAAPPTERIDPPRSPRGISPTQPSTSYDRSSSME

RPGHSSPVWSEGNYVPTSSATSDNWETPTSLISRLSARLTAGTNQEDKGYRNCPSPSMSE

TSSVSMSSSYISHQESAQPYDPFNMAHQELVEEYSKTNGPPDVCRQSTKPHMTEKSKARP

NQKRGRRYTSSESTSSDINVGDLSLQGSQDSDLEEEDCSSGYLDENGKLPDLDTLLKYIR

DPDLSPYSLDKSSIEKFLEPHEIGHINHLIKSVSDAMLTISLPETLYKSKGFTPLDYMDI

HLNCVLRHFFFVFKNEDFLNLVMHDQIALLDGCTLRAIQCTSLYLFNKDAGCWHIPGGTN

KANHPVIHVSDLMQVFPQSFVTRTYELHCIAAKLGFDWPMGVIASCILMYTPLKRVIQEM

EKIDVLRNKYVNLLLKYITWKHGTYNASLLFPEILKLLDALLLHVEDLASITLKLNEEEV

IAVEERLATLTISQMAPYGGQSSKFKEKVASWSTLDCVKLGDIQKRLCMAMHMSMMESAA

PHYSSNSMFYGSGVSQDEQIITQCRTVPQLMPPSGMECSDDRTLERSAYFGSKMLHQNRE

MRGISALAHKDLMLLRKFLEEVTNREGTLVVQNIKEKMDSNQIKKIINKLCS*

>maker-18284_pilon_pilon_pilon-augustus-gene-0.25-mRNA-1-DNA_novel01

MKVPPLNLTPEINRVGMALPFSPDLLWRYPLSLPQSASPSSPMLDVKNQMPTHLAADPRM

WSREDVSAFLRWFEREFELPSIDLSKFCMNGKALCMLNKSDLADRAPGAGDILHNTLQFL

LRDAPQVPQSPITPHHPLLSPSPLTSGPGQPWPIMNPEMHTSFSHFLHQGGSVTLSPAPS

EQSGGSPRHPDTASSTSSTSTAAYHHSSAASTGSAHSGSQSDSEDSTRDSSSPQRSPLPP

SSTAAVSSFPAHALASLKHLSDYQRAAAAAAAAAAAAAQSASSHQENLQHQQHQHQHQSQ

SQPQPQQGPTGPSEEPVETGTNGRLLWDFLQQLLNDPQQRYSRYIAWKNRETGVFKIVDP

PGLARLWGIQKNHLSMNYDKMSRALRYYYRVNILRKVQGERHCYQFLRNPSELKSIKNIS

MLRTAPPQSPRNKDAPSAVQPAPMEEEEGPTDLSMSSIHHQYHSQPPAPMDTQPQNLKRE

PHSLSVSIKTEEYLEHSYE*

>maker-22516_pilon_pilon_pilon-augustus-gene-0.42-mRNA-1

MTMFPRVGEKRRIDDIKIEDEEAIGSLCRLGGRPVSPKTAMEQEKRIRREIANSNERRRM

QSINAGFASLKTLLPHHEGEKLSKAAILQQTAEYIYQLEQEKTRLLSQNCQLKRLLNQQQ

HHADAEGSPAAGDNACDDGVTTVGDTVVSTQVDNGPATEELRREMIDLRVQLDRERRHRM

SLEEHIRSQDAQTFPDRIKDLSQPKSDIREVKVETPPGPVVTETNVTIPPPPTAVEVSTL

RQAPVQVVKTEAEIKMESPRQSPPRHHAPLSIQSPVTTTTITTPAAAIATATTITQPAPR

QPATAVLSRQTVTHTQHNPIQGTIVSAPPATTIVTVVPSAKGGVDALPSVFEALTASGRF

GAKVEVEPAPRVPSPEATIEYVDDNRNRTVYIVNSTNAACQKSLDTICEAIRHLEGDHMF

QTEEQHPRIEEEIITEETLTIEDHAGQQVTLTGSQVPVTIGSHVTLTGGDEPITLHMVGE

PQEVPLELTTHRPSQQSSSHSNLHSSPQSTISLTTRPSHQSPSHSSTTTTSSKDDCTPVC

SISTTTSSHSSPSQSLLQATRPGVIVVKHP*

>maker-25608_pilon_pilon_pilon-augustus-gene-0.5-mRNA-1-DNA_novel01

MEQLQSAHLVSTTAPVTSVPSSSGNSYSYSGLEEFLVPTPVQVEVSNGVARQSSQPLHSV

RFTYVPQVQPVQLTAPSTTAPVRSTQSPTPAPSSQAGAEMEQQQQQQQPQRQPKTSPKPR

VRNSGGERVVCEVCNKSLACGANLNEHMRIHTGERPFLCDECGASFAAKSNLRTHKRLHT

GERPYMCGVCGKTFSQSSHLPSHMRVHTGERPYECPECPKSFSSSTTLRNHLRIHKGDFP

FRCESCGRGFVCKSWLQDHYKVHTGEKPFQCDICRKWFKDKSYITKHKMKYCGNDGYKKR

WRRPGVKKPPGRKPNTAKMKMRMLLEEEILHQPKRARGRPKGSKNKKGKKRKSAATSKSG

QNAVTDSDTEFEVMLQEQEVESPKVEIEEADPLRDDHPKKKEYQSRNQFASESDSSLQSG

SHHGKTHFAVGLQPVSGMHLVAMPQPSVQTHSQQQSHLEQDVQSVTQPQNLVQPQNLTQS

QSIAQPQNLTQQSQSIMQPQNLTQPQSLIQPQNLTQSQDLLQPQNLTQSHSIIHSQSIVQ

SENVRESHDLMQSQALLQPENLSQSQSVNQSLSQPQNLSQTPSHSQPHNLSVAQNLSQPQ

NLSLATKIVQSTDLHQIQNFSQPQNLSQPQNLSQPQNLSLPHNLSQATALSQPQNLSQSH

SLSQAMSLSQPQNLSQPQNLSHDDGMSRAQNLSEVQNLSLSHSLSQPHASLHANSTPIGS

SSHHSQYNIMVFYNS*

>maker-2858_pilon_pilon_pilon-augustus-gene-0.7-mRNA-1

MLDNDLLAKGALSDGQQQPPPLTNPYTQFQQYQQSMAGYNNMGYGFPAMYQNGYGYHLSG

YPHAPSPPTDVTEKPEGGEVRVTAKGKKIRKPRTIYSSLQLQQLNKMFQRTQYLALPERA

ELAAKLGLTQTQVKIWFQNRRSKYKKLYKAAQNGQLPGVDAADIAADLGANLSQMVGPES

PGSPPASDHHDQPPPSVGGADGGPLSPTTDHQDRPQSLPPTVSSHSDMTTPMMTAPRDMM

TSPPAHVQSKDMMAMNQVMSEQRREQMAMMPPHHQWDPAHYMSYWNHYGDMAAHQMNHQI

MT*

>maker-3916_pilon_pilon_pilon-augustus-gene-0.11-mRNA-1-DNA_novel01

MAGVSTMYAPSPYTDSIRLWNIAGVFSYLSGDKSGSGEKGVSPQTPSDEAVTPTNAPVSP

PTSALTPTSAITSASDPSMGDDAEFLNGASVPCSVCNKKFANIHRLQRHMISHQESDVLR

RFKCDECGKAFKFKHHLKEHKRIHSGEKPFECEHCGKRFSHSGSYSSHMSSKKCQLGRGR

TTNGLLPRTPPQYPGTNSKRPVLNPSSYLPILPKDKSPTPSADLGYSSPTRPISRDSLLM

SPPTLVSPSGLNSSSLTSGLPQGFPQGLSQGLQSNLQGLPPYHPLNPLILAAHIHPTLLS

FTMTQAALNKAQNDGLDEKGNSAEAQEDVDDDPEPGELVIKEETREDEEEKSPTIQEECK

KEEREICFPSRDRESMSTLKRMLESVNTTVTKQQFEEKISSANSGSLLSSTAIADDLTCK

LCGAMSKDRLEAISHAYRQCPRLSEVTHASERYKGCLIEGLAARLHVIAESQQHQQQQHH

PQHPSLLMSRQHYTTMNRLNTGKEDESDADSGHVIDDEEMTNDGKKARVRSHFREEHLVI

LRSHYALNPRPKKEELSNIAEKIGFPVRVVQVWFQNNRARERSEGRSVTPTPPLYSHSSY

PSEFSPSSYTASNYPSNYAPTCYPTPTSQASSRALSITPYYPSLLAPGLMYPVGPGRTSP

APADRADELDDDQPLDLSTKKSSPSASPKPASILSDSDADSTSLAISYKSESRTPTPTSL

NNNLTECKEGKESSVAPASTVLPSSVSEQQSKLAQILQGAKLGIPSLYSEHGDHNDKRSR

DDDSGDESRKRRREDEGGSFQCDQCDKSFNKPSSLARHKYEHSGQRPFKCDLCPKAFKHK

HHLTEHSRLHTGEKPYQCQKCLKRFSHSGSYSQHMNHRYSYCKPYRPDGTVLISESSTPV

PSPSESGNGVSSTHGEGVSVIVTATADLKAVSPLETTTDEAPTSAPLASSPEPLETASCL

VKEELVAKPQEL*

>maker-4582_pilon_pilon_pilon-augustus-gene-0.9-mRNA-1-DNA_novel01

MKNLHGQNIEMERTYYKLKSDRHALEFVSLLDDFRVQELYCDATIACDGKKYPVHQVVLS

ACSPFFANIFASVSCQNPLVVIQDVSSWELEGILTYAYRGEVHVPYCNLQSFLKAGRSLF

IKGLMFPQAEKRLGSEPRAETWHEPYYSHGKRTAGLHTHTSAHTDVHGHIHPSGRMQRRD

ETVSYHHQHHSSVHSPVPENSIRVSDHLRRENTVERSFYSGHDIGQQKRHYLADYPQDLR

VSSEMGVERRSSPDSETSDCSSGDRSRWENESSSQASRNTTGDSTARMASPEARKRRRYD

AAERREEPISSHPRFNMPESQGTFQELISGDDERIIKEEPEDDIIDCNSKEYVKDNQGES

DEDEEESPLQIDIKEDPVPDSGCNSSPEPQNEPSSYSRIPEPYSTMDSSSSSSPGHTSPT

SHLRKELLRPVTQNTSVLLQQQQQVPAIPLSIPHPVIALREPQSKPQPPLREPPPLMKIA

QNIPTMVEECEVGSREPPRGLVKDMQASRGNGHLMVTAPPSYHSHNMSFEIHNQHLGPVL

VNAGSPIQISSTSAFSQPKRQNNGTAKNHNNNIFKGDVIWYDSEKPLNLSGVQLSPVKNP

DYSNSEDPFLGKIVTNRKKRLRGPKSWEFLVRLLKDPTTNPNLIRWENEANGIFRLVQPA

IIAQRWGRRTGKHASENLSYENFARGLRYHYATGALKPVSEKSFVYRFGPKALKSLQEGN

NVVFSQTVV*

>maker-5992_pilon_pilon_pilon-augustus-gene-0.8-mRNA-1-DNA_novel01

MASGLLSLKWNNHRSTFFHVLSTVRSKESYCDVTLACDGKFYPVHKLVLSTCSDYFERMF

ERTNCKHPIIVLKDIRHEELEALLNYMYVGEVNVLQNELAGLIKAAECLMIKGLAVPDEA

PSQTKESKRTYHSSGDSPHAKRRKRDDDGRLSSHNQHLHREGREVSHSQRHSSSASTSTP

SSVGRVVPPSSSPSPSVGSASDAVSEECSQSQSETNEAQETSKHPGGEPSSQSKQSVPEV

ILDEPAVKEEPAEIEEEVTDSKEDVVSQFHFDPLQEGDSRSDPGGGGSGGYDSQMLTSQS

QSMEDLVAEALPGSSGLQGNSLWEGEGSLQGFPMEGYSGEGTRPPQMGRGRRQLLSGVDE

EQSATPHRISSSSSYQSSKWQSGGSGTRHACPYCGKTFYKKFNLVTHIRIHTGERPFACP

YCPYRANHFSHLKSHVLKLHHRPLNRGLNLQI*

>maker-62220_pilon_pilon_pilon-augustus-gene-0.4-mRNA-1-DNA_novel01

MSSDVNYILELTGSEWENLLNDRALDFQVRNYEGDGAQNGCQVSNEVGAPQNETSLPVQS

TYGNLYDEFGILNVSPYPPADIFDGQDQESTPQQQQSFNEQPMSTQPPVVVPSEDESGQI

TVSDPLGQMVQHSSAGIPVTGSQQSNTFQGPRAVSEISVPLYAVPQSTAVVIPQQQQTFT

AAKTGGVVDVPALTDMEGDYGFSVTIEEKERTTKSPMWLMSSVTNKLYTNINKAVPFEIQ

LRKKVPSDKSFYIRAVAVFSSAQFLRTNVTRCPNHASTADMTNHGFPYPTHVVRADHPEA

KYEESLTGRLSVVVPLDKLVDGSDFTPILLRFMCLGSCVGGINRRPIAIILTLENGLGQV

FGRKVIDVRVCACPTRDIRTDELALSNKGGKRRGSSHQEKTLPPPRKKPKVEPKVEPQDD

DNKLFTIVVRGRKLYDFLTLVKESYLKTHPECADRYHDPRSFDFLN*

>maker-62220_pilon_pilon_pilon-augustus-gene-0.4-mRNA-1-DNA_novel02

MRFKKAPLKAPGGESSDIIIKEEFESYSDVTNEEMPNTTHAEESSHLGGLGRPLVQGVSG

ILPALQPARTPESYLNKDEETQAAQAKLRQYEALGLIKVEVDEPSEDESGQITVSDPLGQ

MVQHSSAGIPVTGSQQSNTFQGPRAVSEISVPLYAVPQSTAVVIPQQQQTFTAAKTGGVV

DVPALTDMEGDYGFSVTIEEKERTTKSPMWLMSSVTNKLYTNINKAVPFEIQLRKKVPSD

KSFYIRAVAVFSSAQFLRTNVTRCPNHASTADMTNHGFPYPTHVVRADHPEAKYEESLTG

RLSVVVPLDKLVDGSDFTPILLRFMCLGSCVGGINRRPIAIILTLENGLGQVFGRKVIDV

RVCACPTRDIRTDELALSNKGGKRRGSSHQEKTLPPPRKKPKVEPKVEPQDDDNKLFTIV

VRGRKLYDFLTLVKESYLKTHPECADRYHDPSFQNEDSSEDEVKNSNSSQGLEAGDSDSV

SWDSGCNFTQQVGVGFCNDSEVDLVSCPKNNSPLSKLKPNISISPSSLNLRLWQEVPSVQ

SSQDECPELHQSSKAGESICNSETVGISVKSDSLLLSKSSITYSHLSKKDFSVSKHIPVM

QVESYQPGALTVKPKPMQLFKSNFAHSKGEKDKKISVKKTLRETVSLASFEQTESSFDTS

PRKPYMSPDSPEMLAANVLAEGFKGKTVFKTSVMK*

>maker-62220_pilon_pilon_pilon-augustus-gene-0.4-mRNA-1-DNA_novel03

MRFKKAPLKAPGGESSDIIIKEEFESYSDVTNEEMPNTTHAEESSHLGGLGRPLVQGVSG

ILPALQPARTPESYLNKDEETQAAQAKLRQYEALGLIKVEVDEPSEDESGQITVSDPLGQ

MVQHSSAGIPVTGSQQSNTFQGPRAVSEISVPLYAVPQSTAVVIPQQQQTFTAAKTGGVV

DVPALTDMEGDYGFSVTIEEKERTTKSPMWLMSSVTNKLYTNINKAVPFEIQLRKKVPSD

KSFYIRAVAVFSSAQFLRTNVTRCPNHASTADMTNHGFPYPTHVVRADHPEAKYEESLTG

RLSVVVPLDKLVDGSDFTPILLRFMCLGSCVGGINRRPIAIILTLENGLGQVFGRKVIDV

RVCACPTRDIRTDELALSNKGGKRRGSSHQEKTLPPPRKKPKVEPKVEPQDDDNKLFTIV

VRGRKLYDFLTLVKESYLKTHPECADRYHDPRSFDFLN*

>maker-786_pilon_pilon_pilon-augustus-gene-5.6-mRNA-1-DNA_novel01

MHPNMTSKEAEVPPEQVEPVDLSVRTTKTSADSQHTNCKTLKDHPGVVLKVPTFPSFALQ

SLGLDLSLKAVSESPPSPVAPFSVSLEALRTGTTTLLNIQKLHETSLALCGKRSLSESPS

PPPVTSSGPPAAHLSSAVSVSMSSPAAVAAAAAAASLLSPSHSFISSSSSPSSSPPTPPA

GARPPGRPPPPPADYTDALRRRKVHRCDFEGCEKVYTKSSHLKAHKRTHTGEKPYQCTWD

GCMWRFARSDELTRHYRKHTGQKPFKCQLCQRSFSRSDHLSLHMKRH*

>maker-8938_pilon_pilon_pilon-augustus-gene-0.28-mRNA-1

MYEEEYLYLRSRGMAPAAPLGLHPYLGGGLSANLTDHLERSYRLGFHGGMAGGSALAHGF

PGDYLKPHGQCGLPAPGLPGGKPDPRIKVRLENETLWNQFDKFGTEMIITKLGRRMFPTV

KMSASGLEPNTKYFVLMDIVPADDSRYKFQGKEWVVAGKAEPHMPGRLYIHPDSPASGAQ

WMRHPISFQKLKLTNNNLDQQGHIILNSMHKYQPRIHIVAAPDLVSLHWAAFNTFAFPQT

CFMAVTAYQNERITQLKIDNNPFAKGFRENGQLRSKKRSGGASPPATDITSAPEKSTEAE

DATLDKRARLNSVDSGDLDVSDLDDRPASSMSVEKDPSDEKVDIESPPTPPPPFLPPPQI

IKSEAEETPTSIVSPLMSAASMMAAAAASAETAKERTPPAPVEPQRPLHEVGMLSPAAPR

PCLPSPTYSAGLPGGVPAGMAASSLPYPYLYYSHMMSPYLLSRAPQLHATPLEKATAAAA

AAREMYTYPTYLRTPTAHHSSVPTTPSRPAPIHPYSYALPPQVPANL*

>maker-940_pilon_pilon_pilon-augustus-gene-0.32-mRNA-1-DNA_novel01

MEEPQIQYPTNKINKDTQTGLDGPQIFQYPVTTVYGNVHFQDGPTVTKEQKLRTQIEQWL

QSSDKPYECDQCSKRFVQKSHLNSHMLVHTNEKKFECDLCGKKFTMESHLNGHLLMHNTE

KKIECEVCGKRFLQKSHLNGHMLVHSEKKFGCELCGKRFILESHLNSHMHIHSEKKFVCN

ICNRRYTHKSHLSSHMLVHCEKKFECEMCGKRFFQRSHLNSHMFVHTEEKRFECEMCYKR

FAQESHLNSHRIVHTAERRFECEVCRKRFYMKSHLISHSFVHSEEKMFECKECGKRFTQE

IHLNSHRFVHTEEKKIENQTWGKRFTHESYLNGHKFIPYDMKKFVCEECGKRFLLKSHLN

SHRFIHREERRYECDICGKRFTQRSHLSTHKFVHSEEKVYECDECGKRFSQQVHLSSHKF

VHSAERKFECEECGKKFIQESHLNSHRFMHGDRKGISYDDNIKLLTQKSQMNTVLMNADD

KVLTYEDTSKLFQQQAYQPAYTYGHLTGQLAGQLTGQLTGQLAGQLTGQLTNQLAGQLSG

QLTEKVRTLWPL*

>Novelgene0085_novel01

MNIMLRICLASFVIRDCPQNPVLKNICNCIKYKCMTLTKPLNVRFARKFGKKECLKIHLH

RRHSGEKPTFTCEICNENFESKTALARHFRQHKDELQYKCDECGKVFRSKKVLGNHKKRK

HCDELTHNSHENSVSVSNIREYINPIEDLSDNIMEKSPMEGIESNIVEKQYTCSSCGKQL

NSHKSVLNHMLMHAETVLYSCKVCGIAFSNKTLLAKHEGKHDNFNEADGCTKVLCEAVES

QEKITLYIKNDDSSPNKFNNKRKVFMLEERGPHLDQTGKIVNKKWRQGGERDKKEDVMVI

NEDVVIEVEEVIIAEKEDDNTESTGVVLDPLL*

>augustus-1290_pilon_pilon_pilon-processed-gene-0.3-mRNA-1-DNA_novel01

MASSDFVTEVEVQPEIQEVEIEAIPVDMPVESMEQYDGQPIIALQQLPEPGREEIIFQTQ

EEVVGEQEIPYEIPVPVANDVMVESSPGPSKKSHKKGQKRRMKDIDDFATIASGGQKWEQ

KQVQIKTLEGEFSVTMWSSGVDEDDLSNPEPDPDYTEYMAGSNKKFAGIPGVDLSDPKQL

AEFAKIKPKKSSTDDIARTIACPHKGCNKMFRDNSAMRKHLHTHGPRVHVCAECGKAFVE

SSKLKRHQLVHTGEKPFQCTFEGCGKRFSLDFNLRTHVRIHTGDRPYVCPFDGCNKKFAQ

STNLKSHILTHAKAKNNMRAAANTTPQYETDYNSTQQFVQVEMPSPEDQQFVIYTS*

>augustus-25620_pilon_pilon_pilon-processed-gene-0.20-mRNA-1-DNA_novel01

MASDLHNKPKFYSYFKDSHDCKVRLRLHGNPSSNKQQNVPPKTYLGKINKIPPVPSKSPV

EAKSGATPPLKENGEKAQLLVGPQSSVGVIPGLPPPALKENGVKAQFVMGPQSSVGVISG

LSPPTLKGSGELVPVMIPKPKKPPVAVKSGSLPPFKRNDDIIPFKTITKVHVATSTVDPA

MPQTVKGTNGVVPFVTKKVPVVSYKSPIHVKPGMPPPVQGSGEMIPFLVASNPAVEVKPG

TSVPLKGGGEMIPLVAVSKSSEVKSQVAPPPNQGNMVPYMMTSSVDGKSGIPSPMTDVWI

RKGSIGTISKVSPMGASKSIFLPVHVLNSHAQKPLPQMQLVPSQVQNPHPQVQLLNSQVE

KRSPQLQSSQSQVQLLASKAKVKKPNPQAQLLTSQAQTSVPQMVTTSQKYIVAPSSTQFL

GPTQVLTTSHSQLIAAPTVVSSVNGNVATVLRTVASPRIVTTSAQQIGSGLNSLPKVITG

RLKPVQIIMHDVIDRAPTNIPLVDLKDEELYGSEDKFPKIFQCAECNLHFSNKKELERHE

DNHLGEVNYICDQCGKEFSTKRSLEDHLKVHASEKKFECDVCHKRFDYRDNLIFHMRIHG

DVKNISIKCPICQKKFGNKSMRDNHLKRHTVEERNKHKIVNSETTQASSPEKKETSFDCD

VCDKTFSDKVALENHKVVHIKFDRGPKLEVKKKAATVEETTTSTTTFQCRSCLTVFNSDQ

SLRNHASTCGKALTDTIVCAFCKKHYGIFKQSKDDGSQGMKFICEPCCTFLAQKSQVSGF

TPRLENRFICNKCDRCFSNELQLREHLRSHLPGKPVRCDLCSRNFESLSALQIHRAARHT

KRAPLVSQTSLKIGFVTNVHNSKTVETASKSPPAVASSVRRFRGRRRKKRKRIERSKARN

RQERLRYEMRNKEKVLERPTESQVPRKRARYDFGKVADKGSSTEDADESISEADEGKTNS

YEDEDGEYDSDLALGVPYEEDDDDDDDDFIEPHLNVPPSDDDDDNGDDEDVGDDNDEDLF

NKEAMKDLEVTIKQEPIDHDENKTKVMENCTVFNAVPSKGKVSATALFIPDRVIKEEKPD

DYEELDVQIKQEKETMEEMVIKEENVTIKEENLFSQEENDLDSLQFPIKEEKLDESELPM

EEEFEFTPFGGCVKEEPLGL*

>augustus-41828_pilon_pilon_pilon-processed-gene-0.9-mRNA-1-DNA_novel01

MGSIGCPLCCRHDFISVVALHDHLLYYMYRPLQCAICSTHVGGIQELTHHLERHLGDGVS

TTTFHPKPAFDQNTESGNTPFIENGSSCYDDLHDQGRWKNLSENGDIVSHQTREEPIELT

LRAFFCQTCGAKIVGKDSYFLHIKQHISRPSNLTSNNPQPSRSSYQQENSSCGPSPSLVS

INTVPANILTDSETVTTEETHEATESPETQLSDCMEEEHVANQLLKLREWQLGKYGRRFR

KHHLSFSSSLGASDKNSVEGKLVSANPSPNINPGSVEKVTSSHTNDLASVDQTSVEKEPL

PSFWPSSVSYLSDGERNTEISGKCSVPSTPHTLVISEKVDSAVSAVCSEDVLSPCISTPG

EQNEATYHEDEFVQLDDCAVSPLGRINESFNTGYLSDDKIARFPPQESKTLEGSEWSQCS

ISQPGKRSDGGNFSSGGPQPSYDKSSQDMAKQTTVDSNLGISTMNIPFSSDPKSLDTLQD

SYQVPFEGARDFSDNPRFTDRTDEIISDTLTQTENQLFIGDEDKLQSSTHSNSVSNHIVC

EEVFK*

>maker-32052_pilon_pilon_pilon-augustus-gene-0.19-mRNA-1-DNA_novel01

MDLYLPSTEPGLTLQEMIDCDIKCGIDFNEGMDAMITSSDLNSLDLSGMSSLEAELDLKS

FEPLFDPLEKLEDMDTVDDHTADELNAVNSWMNMSSMSNYNIDLENGIMVNPSAVLPTTL

PSTVVKEEVITSSATTSTLTKPTLLIKTEPQQSVTTTVQSPQSQPSNGATVTTQVTVMRP

QTTIGQAIKQQSPLVSSQPVSQTAALTTVAAKPTLTTASQLVVQASQKPSVQMSLSSSTP

NTPTTPVITARTTTSHNIINNRYKTTGKPRKDLRPEEKVFPKPAYSYSCLIALALKNSTT

GSLPVSEIYSFMCEHFPYFRTAPNGWKNSVRHNLSLNKCFEKIEKPVVAGSNQRKGCLWA

LNPAKAHKMDDEVQKWSKKDLQGIKDAMQYPEVLDALEGGEMKFEYSNCGVSSCSEDEDE

DEEVDPLSVDASSSSTVGSVKVNSLTSTVVPNQRVQTSVNHVRHTVQNVRTSLPSNLTHT

TQSQANIRPAFTTQSIKRSQVTSSDETELILPDSTMTEINLQTNGSVVDDLGNEIKVEEE

PSSITSSSPRGLVVRKLPGITPLRSATVRANYVYTPTISSPHQQHHTLNRLVLTNGKLS*

>maker-5902_pilon_pilon_pilon-augustus-gene-0.35-mRNA-1-DNA_novel01

HYLNVYGRWKWIRSFKSIVLTMDGSQFCILWNNYHGSLVNTLSGLRRESDLVDITVVCGD

GSKVRAHQLILAACSTYFRDFLRDHPTKHPIVLLPVEIRYPELKAMIDFMYTGQVCVSQD

RLQSFIRGARYLRIKGLEDNADDSDIDDLPPDLDDKASIDVSGSVGGLVAANGETTTATT

TVKGRRVKDHRYTQQVDLGDSDDNREKWEVVERKPVILPRAQPLPQAGSGIRRRRRARSA

IRKRAMHLSNNIVCYEQRTEGSDGGQERSEPINKIRRLGSPGHFHDDDLDICTCTSRKIW

SVRDTEALLKVWRETLTQVPPSYFIRSMALCKRVAKRLQARGIQKNWRQCQVKMKNLRRE

VRLYKVSVGRNSSRKPSEVATACEKLLPDIEEIFNKEEEIRREWYQMRAEERLRAEGRLV

DGEAAPADILAQVAVLADSDLCDEDTDAQECVVADGLETEGETAFTEGDGFTEGEGGFDG

HVSTHPHVSTQADTAVHHIVENVTLQVVTKQEPQSSTDM*
