# Supplementary material for: PacBio single-molecule long-read sequencing provides new insights into the complexity of full-length transcripts in oriental river prawn, macrobrachium nipponense
Source: BMC Genomics. 2023 Jun 20;24:340. doi: 10.1186/s12864-023-09442-x (PMC10280851; doi:10.1186/s12864-023-09442-x)
Supplement: Supplementary file 10 — Supplementary Material 10 [file 12864_2023_9442_MOESM10_ESM.docx]

The transcript sequence of LncRNA

>chr44:+:14268197-14268252

GTGAGAATGTCATTCAGGATTGGTGTTGGAGAGGAATAAACCAATTGGGTTAAAGT

>chr44:+:14270093-14271650

CAGTCCTGGATTTGCTCCCCAGGTGAAGGGGGTTGATGCGTGAGTGACATAGTAAGATCCAGCCTCACCTGAACCAAAAGTGGAATAGAGTGTACCTTTTGTGAAGTGAATTGGTGTTCAAACCCATTGCTGAGTCTTACCAAATTATAGGTTTTAAGTTTGTGTGAACGTCTCCTTAGGTTTAAGTTGGATGAGTAGCGCCTCCCTGAAGTGTATATTTTTTCTGTATATGTGTTTATACATTAAGTTTTTGATTTTTCTTTTTATACATTTTATGCATTGATGCTTTGAAAAATTGGTTCATCTCTGTTGAGTGTTCTGCCTTGTGTCTTCAGCAGTGGGTTTCCATCAAAAGTCAAAGTCATTGTAGCTATCTTTAAAAGCCTTCAGGTTATTAACTATGAAAAAGATCTCATAATGCCTTGCTGATGAAAGATCTCAATACACTACGAGAAAACTTCAAAGTAATTACAGTACCTACAATATTGTAGCTTAAATGACTTTGAGCTATAAAACCAAGTCCACTTCAGTACCTACCTGTAATTAAAAGTAAAAAAAAAAATATGTACAAAGTTCGTCGTTTTGGAGATATGGAATGGGTGGTAGTTCTTTGTGGCACGATCATTTAGCGGATAGAAGTGGATTAAAGAATTATTCCGTTAAGTCGACGACAAAGCCTGAGGTACTACTACTTTGTATCTCCTCTTACGAGGAACACTTAATATTTCTAGCTTTGCCACCATTTTTGGAGGATTTTGAATTTCAGATTTATTGAATTTGTTTTCTGCTTGATATATGTTTGGATCATTTAATCAAAATTTATTATTGAGCTAAAGTCTTGAAAGTAATGATAATTCCCTTAGATGGTGCTAATTTTTGTTCTATTGAGTTTTTAATGCACTAGAACCAGCTTTCAAAGCATTTTTCAGAGTATTTTATTATTCCGACATACTAGACATTGTAATTTTTAGGAAAAATAAAATATTTAAAGTAATTTTATTTTTATTGTTGAATACCTTATGTTCATTTTGATAAGGAGGCACTTTACTTTCATATTAAAGAAGCCACTCCAAACATATCCACTATTTATTTGCCTGTACTTTTGAATCCAAAAATGTGGCATTGTAGAGCACTTGATTAGTGGTTAACCCTTTGCAAAATTCATAGGAAGATTAGTTTATTACTGTAGTCAGAATGTTTATACTATAGCTCATGTGTGAAAGTGGATGTGTATGTCGTGACCATGAGGTTGAAAATGCAGATTGAGTGTTTGATAAGACTGGTAACTCTAACAATTCAGTTATCCCAGTCCTTTCCTCTTTGGAAGGAGCTGAAAGTGGTTTCATGGTACTTGAATATATGACTAGATGGTTGAGAGGTGTGATTGGCTTGTGTAATAGTAGTCAAAGTGTGTTTTAGAACAGGATAAGATTTCATATGAAAAACACTATTGTATTTTTTTCAATTAAGTGCACGGCCTTCCTTATTGAGGAGAAGCCTGTAGCTTTTAAGAATAAGAATCGTGTCTGTTTCAATCTACAATAAAATCTTTGTTCAG

>chr5:+:128381259-128381305

AACCACGACTCTTTGTAGGCATGATCCTCATTCTTATTTTTGCTGAG

>chr5:+:128381596-128384818

GTATTGGGATTGTATGGCTTAATTGTTGCCATTTACTTGTATACTAAGACAAGTAATTAAGTGGAAAGGAATATAAAAAATGTGAAGACTGTGCCAAATAAATGGCATTAATTAAGAATGGAAATTTGCAAGTGGTTGGAAGCTAGCAGGAAAAGAAGAACAATCGTTGTCTCATTGATTCCTGAGTAATTTCTTCAGAACAAGGCAGTTTTTGTTGGTGTGGTGGTTGGTGTATCCATTATAGGTGCTCAGAAATAGGAAATGGAATCCAATCATATATTTTCTTATTTTTATGAGGGTTATATTTTGTGTGATTTCTCATAAGTTGTAGGAACATGAAGATGTCTTTGTAATAGATTTATCACTGGGTGAAACATACCTGTGCAACAGTATTCTGGCTATAATGTCGATTGGAGTCCAGATCAGATTCTGACATTTTGTACAGATGTGGGTCTATGACATTAACATTAAAGGCTTTGCTCTGTAGAAACTAACCTCAGTTTAAGTATGAATTTGTTTTTTATTATCAGTCACTGGTTTGCAAATGACTAGGTAACTGAATCAGTTGAGAATTGGGTGAATGGTAACAAATTAACTAAGGAGAAATGGGTAAATAATTTTATTTTCGACAAGTATGTTCAACAGTGAAATTTTCAGGGCCATTAATTATTTTTTAAAAAGATGCATTCGGCTGTTAAAAAATTTCTTGTCTTCAAAGGCACATCAGCCTATACTATTGATGCACAGTTTCCATATTTTTCCTCATAGAATATGATAGTCTCCTGATGTGAAGCTTCTTATGATTTCATGACGGGTTTGTTTCCTAGTGTGATATGAAAAAATGTTTATCGTTGGATTTTGCAAAATACATTATTTGCTGAAGTTAAATGTTGTATAATGTAAAATTTCAATCAAGATTCAAGGTATGTTGCTAATTTAAGTTTGCAAAATATACAAGCTATTTGTCCATGTGAAGTTTGACAGTATTTTTGTGTTGATGAAATTTTACAGAATTTTTTATTTATTTAAAGGATTTGAAGAATTGATAATCAGAGTTTGCAAAGAACATTATTTGCCAAGAGAAAATTATACATGTTTTAATGGGTGGTACTTTTAGTGATATTTAATGTGAAAACAGTCACAAGTAAGAACTAGAGATGAGATGGTTGTGATAAAAAGGGATGGTCATTTGTGTGTAAGCTGCATTAGGAGGAAATTATCTTTACTGAGGAGTATAACCAGTGAGACCTTAGTAGCTGCCATGCAGGAAAACAAGGTATTATAACAGGACATAGCATAACTTAAAGTGATCAGCAAATACTAAGCAGTATTTCCTGGTATCAGCTTCTGTTAAAGTGTTCAGACAAAATACTTTATCTGTATAATTTGTATGAGAATGTTAATATGTATATTTATTTTGTAAACAGGGAGTCATGTATTTTTTGTAGATTATTAAGTTAATGTTTATTTGTGTATAGAAAATCTATTTAAATTAAGACTATTTTCATAAAGTTATTATTGTGTAAATTTACTGGTATCTGTGTACCATGTACCCCAAATTCCTTGTATGTCCAGGATGAAAGAGATTTACTGTAACATTTTTATCCTGTAATATGGTAACTGAAGCACCTCAGTGGCATGGTCGGTATGATGTTGGCTTACCACCTCGGTGGCCACGAGTTCAATTCTCCAGCATTCCATTGAGGTGTGAGAGATGTGTATTTCTGGTGATAGAAGTTCACTCTCGACGTGGTTCAGAAGTCATGTAAAGCCGTTGGTCCCACGCAAAGTAAAAACGCCATACAAACAAACAAATAAATATGGTAACTGAGTCATTAGACCCTTTGACTTTACACGGGTTATATTTCTTAGGTGGGACTTGGGTTTAAACATTTTTGATATTTCTTAATATAATAATCAAGTTTGGCAGAACTTCCTAAAAGTAAACTATAGTTGGATGTAACTCTATAACAGCTTTTGCAGCCTCAGTGTTCCTGTAATCTTAACAAATATTCCAGAAGTGATTGTTGTTTATTTTGACAGCACAGCTTAACACATTTTTTCAGCCAGCATAGCTTGATGATTATTTCAATTTGCTTGTGTAGAGATGCACTCGTACTCTATACTTCACTGAATTAAGACTATACAGTTTTCTCTAGTTCCCTAATTGACTGTTGAGTATCTTTGGTATGATGCATTTGATGTAAGGTGTAAAAAATTAATAAATTTTGTAGGCAAACAAATTTAAAAAGTTAGTATGATATTTAAAGGGAGTATATTCGGTGAAAAGCATATAAAGATTGCAGGACACAAGGGAGAGTTTTATTTGTGTTCCTCCATAGATTTGCAACTGTTTCTCTGTTTTAATTTTGAAAAATTATGGCATCAGGCCTTTATCATGCATACAAAATCTTTGAACCTAAGTTTGATTACCATATTTTTTTTTTTATCAACTATAAATTTTTCCTTTTAAAGTTTATTCATTCTACTTGAAAAATTTTTGTAACTAGATTACATATGAGGATATAGATGGATTTTGGGGTTAAATTTGCATATGGTATATATATCAAATTCAGTAGGTGAAGTGACTTGGCAGAAGGTGGTTGGCTTACATATCATAGGAACCATCTGTTAATCTTGCGATTTTGCCAGTGCACCAGGAATACATTGTGTATCTACATTGGTAGCATTTTCATTAGCATTGAAGCACAATCTAGTGAATTACGAAGTTTAGATTGTAAAAAGTTTCCAATTAGGTACATTCCAATCTTTAAAGAAGGTGGTGTTAGGTAACTTTTTGGAAGACAGAGTAGGTTTTATTTTTTCTCATATGATGTATGTACTGAATGGTGTGAAACTGTGAATCCTGTACTTCATTATTTCAAGTACTATGTAGTACCATAGAACAAAATGTAAAAACTTGGAACATTTTGTGGCTTTATCAGTGTGCACTAGACTGTAACAAAAGAAAACATCACTCTTTAATTTCAAATTAGTGTTGGGGTCTTGTCTGTAAACATTACTACTCATATTTATGTCAGTGTTACCATGTGATCAAAATAAGTTAGTTGTCATCTTGCTCCCAACCATTGAAGGTTAGTATTGGTTATAAGTGTGTTCAATCAGACCAAAAAGTTAAAAAGAATTAAAAGAAGTGTATGTATGTACAGTATATTAATAGAAGATTGGTGTAAAAGTTGTTCAAGTAAATTATATGGATGTC

>chr5:+:128381729-128384818

GGTTGGAAGCTAGCAGGAAAAGAAGAACAATCGTTGTCTCATTGATTCCTGAGTAATTTCTTCAGAACAAGGCAGTTTTTGTTGGTGTGGTGGTTGGTGTATCCATTATAGGTGCTCAGAAATAGGAAATGGAATCCAATCATATATTTTCTTATTTTTATGAGGGTTATATTTTGTGTGATTTCTCATAAGTTGTAGGAACATGAAGATGTCTTTGTAATAGATTTATCACTGGGTGAAACATACCTGTGCAACAGTATTCTGGCTATAATGTCGATTGGAGTCCAGATCAGATTCTGACATTTTGTACAGATGTGGGTCTATGACATTAACATTAAAGGCTTTGCTCTGTAGAAACTAACCTCAGTTTAAGTATGAATTTGTTTTTTATTATCAGTCACTGGTTTGCAAATGACTAGGTAACTGAATCAGTTGAGAATTGGGTGAATGGTAACAAATTAACTAAGGAGAAATGGGTAAATAATTTTATTTTCGACAAGTATGTTCAACAGTGAAATTTTCAGGGCCATTAATTATTTTTTAAAAAGATGCATTCGGCTGTTAAAAAATTTCTTGTCTTCAAAGGCACATCAGCCTATACTATTGATGCACAGTTTCCATATTTTTCCTCATAGAATATGATAGTCTCCTGATGTGAAGCTTCTTATGATTTCATGACGGGTTTGTTTCCTAGTGTGATATGAAAAAATGTTTATCGTTGGATTTTGCAAAATACATTATTTGCTGAAGTTAAATGTTGTATAATGTAAAATTTCAATCAAGATTCAAGGTATGTTGCTAATTTAAGTTTGCAAAATATACAAGCTATTTGTCCATGTGAAGTTTGACAGTATTTTTGTGTTGATGAAATTTTACAGAATTTTTTATTTATTTAAAGGATTTGAAGAATTGATAATCAGAGTTTGCAAAGAACATTATTTGCCAAGAGAAAATTATACATGTTTTAATGGGTGGTACTTTTAGTGATATTTAATGTGAAAACAGTCACAAGTAAGAACTAGAGATGAGATGGTTGTGATAAAAAGGGATGGTCATTTGTGTGTAAGCTGCATTAGGAGGAAATTATCTTTACTGAGGAGTATAACCAGTGAGACCTTAGTAGCTGCCATGCAGGAAAACAAGGTATTATAACAGGACATAGCATAACTTAAAGTGATCAGCAAATACTAAGCAGTATTTCCTGGTATCAGCTTCTGTTAAAGTGTTCAGACAAAATACTTTATCTGTATAATTTGTATGAGAATGTTAATATGTATATTTATTTTGTAAACAGGGAGTCATGTATTTTTTGTAGATTATTAAGTTAATGTTTATTTGTGTATAGAAAATCTATTTAAATTAAGACTATTTTCATAAAGTTATTATTGTGTAAATTTACTGGTATCTGTGTACCATGTACCCCAAATTCCTTGTATGTCCAGGATGAAAGAGATTTACTGTAACATTTTTATCCTGTAATATGGTAACTGAAGCACCTCAGTGGCATGGTCGGTATGATGTTGGCTTACCACCTCGGTGGCCACGAGTTCAATTCTCCAGCATTCCATTGAGGTGTGAGAGATGTGTATTTCTGGTGATAGAAGTTCACTCTCGACGTGGTTCAGAAGTCATGTAAAGCCGTTGGTCCCACGCAAAGTAAAAACGCCATACAAACAAACAAATAAATATGGTAACTGAGTCATTAGACCCTTTGACTTTACACGGGTTATATTTCTTAGGTGGGACTTGGGTTTAAACATTTTTGATATTTCTTAATATAATAATCAAGTTTGGCAGAACTTCCTAAAAGTAAACTATAGTTGGATGTAACTCTATAACAGCTTTTGCAGCCTCAGTGTTCCTGTAATCTTAACAAATATTCCAGAAGTGATTGTTGTTTATTTTGACAGCACAGCTTAACACATTTTTTCAGCCAGCATAGCTTGATGATTATTTCAATTTGCTTGTGTAGAGATGCACTCGTACTCTATACTTCACTGAATTAAGACTATACAGTTTTCTCTAGTTCCCTAATTGACTGTTGAGTATCTTTGGTATGATGCATTTGATGTAAGGTGTAAAAAATTAATAAATTTTGTAGGCAAACAAATTTAAAAAGTTAGTATGATATTTAAAGGGAGTATATTCGGTGAAAAGCATATAAAGATTGCAGGACACAAGGGAGAGTTTTATTTGTGTTCCTCCATAGATTTGCAACTGTTTCTCTGTTTTAATTTTGAAAAATTATGGCATCAGGCCTTTATCATGCATACAAAATCTTTGAACCTAAGTTTGATTACCATATTTTTTTTTTTATCAACTATAAATTTTTCCTTTTAAAGTTTATTCATTCTACTTGAAAAATTTTTGTAACTAGATTACATATGAGGATATAGATGGATTTTGGGGTTAAATTTGCATATGGTATATATATCAAATTCAGTAGGTGAAGTGACTTGGCAGAAGGTGGTTGGCTTACATATCATAGGAACCATCTGTTAATCTTGCGATTTTGCCAGTGCACCAGGAATACATTGTGTATCTACATTGGTAGCATTTTCATTAGCATTGAAGCACAATCTAGTGAATTACGAAGTTTAGATTGTAAAAAGTTTCCAATTAGGTACATTCCAATCTTTAAAGAAGGTGGTGTTAGGTAACTTTTTGGAAGACAGAGTAGGTTTTATTTTTTCTCATATGATGTATGTACTGAATGGTGTGAAACTGTGAATCCTGTACTTCATTATTTCAAGTACTATGTAGTACCATAGAACAAAATGTAAAAACTTGGAACATTTTGTGGCTTTATCAGTGTGCACTAGACTGTAACAAAAGAAAACATCACTCTTTAATTTCAAATTAGTGTTGGGGTCTTGTCTGTAAACATTACTACTCATATTTATGTCAGTGTTACCATGTGATCAAAATAAGTTAGTTGTCATCTTGCTCCCAACCATTGAAGGTTAGTATTGGTTATAAGTGTGTTCAATCAGACCAAAAAGTTAAAAAGAATTAAAAGAAGTGTATGTATGTACAGTATATTAATAGAAGATTGGTGTAAAAGTTGTTCAAGTAAATTATATGGATGTC

>chr35:-:28836642-28838528

GATTTCTCCCCCCTCGTAACTAACTAATCTACGTTTCGTTTCCCGCCTTTTGCTAAACACGGACAACGATTTGTTCACAGCAAGCAGCAAATTCTTAGACATCACTCGCATAACAAGAAGATAAGCACAAAATAACTCAGCCACGGAGAGGAGTCTGGTGAGCATTGCTCGTAGTGTGGAAGGTAATATGCTCGCGACGCAAAAGTTTTTCAACTTAGAATCTATACGCGTATTATAGCTACGAAAATACAAATGTCAGTCAGCTCTCCATTCGCTGTTCCGAAGGCGACAAGCATCGCGCACTCTCTAAGCTAGGTGTTCAGTCAATTCTGCATTGCGTAACACACTCAGAATAGATACGAGGTTGTTCTGATGCGTTATAGGTGAAAACTTGGTGCTGAATCTCCTGTTGCTTGTAATCTGCTTTAATGGATATCTTATTAAAGACAAAATGGTTCTTGGAATCAGATATCTTTTATTCAGAGACGAAATTTGCTAGCGACATTTTGTCTCCACATTTCGTATACAGATGAACACATGTTACGAACCTCAGTGTGTGTGTGTGTGTGTGTGTGTGTGGACGACTTGTTTATCTATTTAAGCCCGCCATGATCCCTGTCACATTGAAGCTTAATATATCAGAGCAATTTCATATATCAGAACGTTGTGGTGCCGACCTGCATATACTCGCTAAATGCTTTCACGTCGATTTGCTTGTCAAAGATACCAATCTCTCGCTCTCTCGGTTTCGTTTCGCTAGACCTAGCACTAGGAGGCATGCAGCCCAAAAACAACACTGAAAAGTGAGATAGAAGAAGTTGGTCTCTCCTCATCACGACCGCTGCGTCCAAACTCATCCTGGAATCTGGAAGATGACCGCTTTGTACCACACGATGACTCCGGTGCTTGCTTTTCGACCTCGAAGGAAAAGCAACCGATCTCCTCAAGACTTCAGCATTCGAAGCTTGCTGGTATCCAACGGAAAACAACCCCCACACACTGGTTTCGAAATGGATCTAATAAATACAGGCTATCATTTACGAGCTCTCTCATCACCTCGCTTGGACCGTCTGAGTGCGCCTACCTTCACTCCCCAGAAGTGCTGTTCTAGAATAGGCATTGGCTGTCAAAAGGAAAACACCAACTCCGACTTGCACACTGACACCTGTTTCCAAGACTTTTTTCCTTTCGTTTCTCCTCCACATCCACAAGAGACCATGACATTTCTAGCACGTTGTTTGGTCCACCTGATAATCAAATACAGTATTATCGAATACAAGAGGCTCTGTTTATTCGATCTAGTAACTGACTTTATCTCTGTCAGATAAAGGGTGAAGAATAAAGTGCCAAATGACACCTAAAACCGGATGCGATAGTTGAAGAAATCACTGTCATTTGGATAGTAAGCCAGCAATGAAAGTTTCCTATGCAGAATGACAGCGAAATGTCTCTCTACCCGTGCCAAAACTATTTGTTTCTGTGCCAAAACTGAGGAACAGATACAGAACAGATTACAAAAGAAATTACAGTATTTCCTGGTCCCATTTAAGTTCAGACAACTGGACAAAATCGCGGCGCGTTCTACTAAAGAACACATTCGCGAATTCCATTTTATTTGAGACTGTAAGACCTCCATTTTAATTCTCTGTGACTGGAAGACTGCCACTTTTCGTTGTGACAAGAGACAGAATCTTTTGAAATTTCCCTGAGCACAGTTACTCAGATTCCTCATCCTTTAGATCAAAAAGACTCACGGACATAAAGTGGAAGGCCAGTGACTGTGCCTTACAACCCAGGATGACCTTGCTAGGTCAGGGTCATACACAGACCACTCAATGGTGATCCCTGTAACTGTCAGGCACTGAAAACAATAAAAAAGGAACCATG

>chr24:-:16105019-16105852

AAGAGCTCAACAGATCAAGACCACATAATTTTCTTAAGCTCACTTGGTTTTGTTGTTTCCACATCAACAGTAGTTATTCCTCGGAATGGCTTCCCTTCTGGGATGCCCAATGATAAGTTCATCAGGTTCTGTTGCATTTATTTATTGTTAATAAACTATTTCGATAGGTGAACTAAAGACTTATGGCATAGAATATAATTAAGGCACATATGTTCACAATCAATACTAAGTCAGTGCCTTAATTTTATCTAAATCTTTATCTTAGGATAAGAATATATAAGCTTTACAGAGTAGTCTAAGTATCCGTAAGCCAGTGAAAAATGGACAATTAACTGTGGGGTCTGTTACTAAGACCAATGGTATGGAACATGTTCTCTATCTAATGCTACAACTTATTGAATCTTTCATGTATTGTACTTACTGCACATACAAACAGATGCAAACTGTGAATAACTATCGAAAAATAGTGTATATATACGAATTTCATAAATGTTTATTTAATATCTTCTTCTTCCCTATAAACACTGAAAACTTGGCTATGCTTGCTGAGTCTATATAAATGCTATGTGTCTTCTGTCTAAAGTTCTTGTGTTTGTGTATTATTGGCTATTAGTTCTTATCAGATTACTTTTATCCCATAGAGCTTGCTTAAGGGCCAATGAAACTCAGGATGGCCCTGTAGCTAACCTGTTAATGGATATAATTGTGAAATTTACTCTTGTTAATTAAAAAATTGTGCTTTAGAGTATTCGTTACAGTATTATAAAGAAACGTGTGATTAATCATCAGATGTCATGTACCTCTGTCTTTGGGGGCTTAACATAATAAAAGAGG

>chr3:+:52408289-52411077

ATCTGAGAAAAACAATTAAGCTGATTATACAACAGTTATTGTTTTTTACTTTTAATTTGACTTCAGTATATGGAGAAACATAAGTTGGCAAGCATGTAAGTTTTTAACCATGCTGTATCTCATGATTTTCTGTATTTTGGGGTTGTATTATTTGCATTGATGAATTAATGCCCTGTTTTATAAGTGAATCATGTGCCTTAGCAATTAAATAAATTTTATATCAAGATGTAGATATTTCACATGAAGGACATTATGGAAGTAATGAAAATGAGATAATTTCTTTAACTGCTGAAATGCTTTGCATTGATGGATAAAGTTTTGTCTTTATTTTGTAGGTCTAGTCGGTTATGTTCTTTTCCACCTTATTCTCGTCTTGAGTGTTGACTCAGAAAAATGCTGCTTTATCCCATTATTAGGTTTGAAGCTCGCTTTCAGCAGTATTATGCTATGTATAACTTACTCTATGCAGTCTATGCAATATTTTGTGATTTGCTCATGAACTACATTTTGGGATGACAACTTGCAGCTATGAAACTACTGTATGTACATGCTGAGGAATGATTTTCATCACTACTATTTTGTCTTGCATGGTCATAATGTTAACTCATTTTCCAAGGTATACATGTTCCTTAATGTGAAATTTTTGTGATTAAATATCATTCTGCACTTTCCGTCACCACAAGGTTGTACCAACTGATACTGTAACTGGGACTTGCATAATATATTTTTAACATATTTATGGAGGAGTTTGCAACCAGTTAGCATGAATTTTAATAACCCCTGGTGTGTTAGTGCTTCATGTCCAATCTGCAAGATCTTATTTGCTCTTATGTAAATTGTAGTGTAGTGCTGCTTTACTGGTTTGCACTATTCATTCATTTTCAGCAAATGGGCAACAGAAGCAACACTGTGTAAAGAGGTTGAGGTTTTTTGTATTGTGTTATTATCTTGTTTTTCTGTAAGATGGATTGTATGAAGTTGTATCATGTTATTCTGGTTAAAACTTGGGAGCCACCAATTTAATATAATTTTTGCTAAAAGCATCATTTGCTGCAATATGAATGAGTGAGGAGTTCTAGTAGCATGCTCAGGAATTACATTACCACCATTGACTGTAAGGTGATAGGCTGATTATCAGGTAACCATGAAGACACTTGAGAATGCTCCCAGTTTAGCACCTTCCGAGAGTTAGTCTTTTTATTTTTCTGTAAACTTTTGGTCTTAATTTGAAATTTGTTTTAGAAAATATATATCCTATAACCATTATAGCCTGTTTATATTCATAACTGCATAATTCATATTCATGCATCAGTATTTCATATTCACTTGTAGAATAACATAAGAAATAAGTATGAAGATAAAATCTTGATAGTGTAGGTCATAGATTTTGTGTATGGTAGTTAATTTTAGAAGCCTGAAAACTAATATGCAACTCTACGAAGACTGACTTTATTAAATGCCTCTGGGTAGACCAAGACGTTTCAGTGTTATTGTAGTTTTATTTTAAGAGTTGTCATATTGCCATCCTTGAAAAATGTGTAATAGGAACCAAATTATATTCTATTCTTTTTCTGCTAGCTCAGTGATTTTAGTGGAAATATTTTTTGTATTTTTGATGAAGTGTAATTTTACAGATATTTTGTATCAGGTGTGCTATACTGTTTAAAACTACTTGTATTTCCCAGAATGAATTTCTGCAAATAGGGAGAAAAAGTAAATTGCACTGCATATTTTGCTCCCAGAAAATAAGTGCCTATTAATTACTAATATACTGCTTTTGCTGCAGATTTAATCTTTTGCAAGTTGTGCCTCGAATTTCATAAATATTTACAGAAGTGTCAGTCTTTGGTTTTTATAAATTGTATAGTCATGTAAAATGTTAGGCTTGTTTTCAGGTCTTCATGTTCTTATCAAAGTTTATTTAGTTTTAAGACATTCCACATGTAGGATTCCTCATAAGTTTGAATTATTAACAGGTTAGCAAGGGAATTATGATTTCATGGAGAGAATGATGATCATAACACACCTAATTTAATGATTCGTCAGACATTATTGGTCTATGATATATCCAGTCTTGTTGTATCGGATGTGTTAATACTTGCCAGGTTACTTCTGCAGTAGTTTTGTATTTTTTAAGGTTGTGATTTTGTTATGTATTTCAAAAGTTAAATTAGTTTCTTGTAAACAGTCCTAAAGGTGTGCACACTCATGTTCTGTTCATCATTAATTACTTTTATAATTAGAAATGGAAAAAAAAAAATTTACTTTGGTTTGTCACAGCATCACAGCAGACTTCCTGTTAATTTTACATTTACATTGTACTTTGCTGTGTTGATGTTACTATTTGCATCCACCCAATCTATTCTTATAGAGCAAGACTTGAACATTATAAACCTCAACTGTTACACAGACCCAGGCTGTTTTTTTCTTTTCTTTTAGCTTAAGCCATTTCATCCTCTTGCTTCTATTGCAAAGGTCTTTATTTATTCTTCCAACTAGTGTATGTCAAGTTATGTAATATTAGCAGAATTAATTTACTAGTGCAAAGTTTGTGTATATACTTCTGTATAATATCCAATGGTTTGTTGCATTGTGGCCTTGTATTAATAAGTTAGGCACATAGTACTGCTTATTACATAATCAGAAGCAGGCTTTACTACCATTATGTTCCTTTTTAGAAACACAAATTGAAGAACCTTTTGTGAACTGCTTATGATACTGATGTATAATGGATTGTCTTCCAGAACAAAAAGAAAAAATCCTGCTCATTATTATATGCTTAAACAATC

>chr18:-:49359629-49361983

TGGAGCTAAAATGTAGGTGCTTATGCAAGAGAAATTATTGCACAGTATTGATAAAGTGGCTTTTTAAAGCAAATGTTAAGCTAGACTTGCTGGAAGGAAAGAAAGTCATATGTCATGACATATTTTGCAGGGATTTGTGCCATGGTAGTTTAAATCTTATGGTTCTTCCATTTGCACTGAGGAAGTTATTCATGGAAAAGTTTGGAGTCCTTCCATGACTGAGTAAAAGGGTGGTTTTCATAATTATGATTAGTATACTATAATAAGATTCATAAGTATTTAACTAATATACTAGTGTTGTATTTTAGTTGCATATGCCTATACAAGTTAGAAATGACTGATAATACTGTACAGATTTGCAAAACCTCAACCTAGTATTTATGTAAAGTGGATTACAATACCGTTTTAAACACTTCCATTCTATTGAAACTGCTGTACTTGTCTTGTAATGTCTTAAGTTTTAAATGAAATCTTCATACAATATTTTTCTAGTACATGTTTAATATATCAAAAAGTTTTAAGATGTTAATGGTAGTAAGCAGTAGTGAGTTATTGTAGGGAGCTTTATACATTCACTCAAAGGAAATCCTTTGAATTGAGATGTTAGTGCTTGCAGATATGCAGGAGATTTCTTAGAGCTATTTTGTTTTTCCGTCTTAGAAATTATAATGAAAAGTTTTCCAGAAGCTTTTAAGTATTCATTTTTTGTGTACATAATCTACTAAGCTTTTACCTTAAAGTTTAAAATAGAAAAGGCTTTTCATTGGCAATTCTGTTCATCAGCTTTACTGAATTTGTTATTGTATGGTTAGTTTTATAGTTTTGTAAATGTTTCTAGATTCACAGTATATCTGAATAACCTGTATTATCTTCATAAAATTGAGAAATGAGAGTAGTCATTCCATTGTTTCTAGTACTTTGGTGAAGTATTGGTGAGTGCCATCTTCAAGGATAAAAAGTTCTTTGGAGTAGTAAGACAGAATAGTAGCATCTACGTAGTTCACTGTAGTTTTTTAATATAGAAACCACATGAACAATTATTTTGTTAGAAATGTTTGAAAAACAGCTCAATTTGTATTTTAAAATAATGCTATTTAGAGCTGTTGCAGATTTAAATGAGTATAGGCATGCCTCTAATGAAGGACAAAGTAAAACCTTTACATTACCCAAGTATCTGAAACTTTGACTTGGCTTCCTGCATTTTGACCCAGAAACAACTTTTGGCAGTGCAAAGAGTTTTCCACCTATTGCAGCAACTGGTTGTGGACCAGCAGGTCAGTGTTAATATACTGTGTCATGTTACCTTTTCCCCCTCTTTATGTAGGACGTTCCTTTAAACTTGAGTACTTAATAACAGTTGACATAATGGTATTTTTGCTGTACAGGTGGCAGTCTTATGTTTAGTGTACAGTACTATCTTTTAGTTAATACCATGATGTGCTGTTCCCTATCATGTACATTATTTCTCATCTATATTTTGTTTTCCTGTCATTTCAGATACTTTATTGTTAGGGTATTTATGTGTATCTAACTCAGATGATGTATAGTTTCTTTCCTCAAAATCAAGTTGGACCTATTTTTGTGCAAGATATTCAATGGAATTCCATTTAAGAAGTAATTGCTCATTTAGGACCAAAACACACATTCCATACATAGGTTTCCCATCTGAACTTTGCTTGAATATTTTTTTTATCAACTTACCACAGCCTAATCAAAAGTTTTATGCAGATTTGTAGTACTGAATTTAACAGCATAGTATCTGTAATTTGCAGATTACTGCGTAATTTTTTCCATAAAGTGCATTTCTTGCACCTCATTCTAGGCAAATTGGTTCATTTGAAGTATTGTTAGCATTTGTTGAAGTTCAACAAAAGTTAGAGCATCATGTGCGGTGTACTTAACTTACATTTTAACCTAAGCAAAGGAATGATAACATATTTTGATCTCTTGCTTTATTATTGGTTGGCAGTTCAAACATTCATAATTCATCAGATTGGCATAAACTATTGTACATATAGAATAGTAAAATTTATAGATATTCTAGTCATAAGATTTTTCATAATGGAAACAATTGGACCAAATCTTTATTACTTTGTTATTTGTTTGGTTACTATTTTTCTCTGGTTGTAAGATTATTATATATTGACATTAATCTGCTAAATTTCTAATTATTTACTGTAGGTTGTGTTGCCAAGTGCTTCTATGTGTTTGTCAACTAATCATTTGATGTTCTACATCATATAAATTTCCTTATATTTTTTATTACCCTTGGTATTTGATTGTATTATTCTATTCTGCACAATCACTTGTAATTTTATTTAACACTAGTCAGTGTGTAAATAAAGAAGCCTGTTT

>chr39:+:52165606-52167018

ATAAGATTTGGGGCTGAGCGAAAGTTGGATGTTTTCACCTTTGGGAAATAATTCATGACTATCTCACATTTATTGTTGTTTTCTGTCCTTGATGCTGCTCGGTTGTTGCATTGTGTCGAACAAGACTTATTACATGTGACTCTTCTAATTTAAAAGTGATAGCATAGTATCCTTACAAGAACCTTTTCGAATGCAGTCTTAAAAATTGTTTTTCTTTCATTCGTTATCTCGGTATTGTATTTTTTTTTAATTCATTATTTATCTAGGCATTATTCTGAAAGTATAATGACGGCAACCCATGAGTTTTGTCATTTGTATTAGCTGGAGTCGTGAAATAGTTTGTCGATAGCAATTCTACATACTTAATTGTTGATGATCCCTCTGGAAACGTTTGACAATTACCCTGTAAAAGGGGCGGGGCTATGTGTTAACTTAAGAGTAACATTCAGTCATGTTATGTAGATGCAACTAAAGCATATTTGTCCTGTATTTGGTCTGTAAGACTGGGATTCTGGAAGATTTTGTGTAGAACATGATGTTCCTCATCATGTGGCATCTTTGGCCTTTATATACCAAGATATAATTGAATGTGAATGTTATATAAGTAATATTTTTTTCGTTTCTGTTTCTGCTGATTATCTTATGTAGGTTTACATTAATGTTCTTATTAGTGTCAGAACGCCAAATAGCAAATTGAAAGCTGCATAGTCAATATGCATTGTCTGTTTACCAGACCACGTAAATCATGAAGTTAGTAAAAATCAACCTATTTTGCATAAAATCCATTTTCTTTGATAAATATAACACATATTTCAATTATTAAAAACAGAAGATTGCTCGTTTAACGTTAATTTACCGCTTAGCATTCAATTTGCTTTTAGTTTAGGTGATAATACAAAATGCTCTTTGTTACGTCCAAAATAGACAATCAGTGTCATTCCTTTTGAGGATTACTTGTCTTTTTTAATAAAATTCTGCCTCAAATGAATAGAAATGTTATTTTGGTAGCAACACACTAATGGATTATAAATTGACATATGGCTCGTGAATATATGAGTTTTTGTTTTGTCTGACATGTAAGTAAATTTGCTAAGTGGGCAAAAGCATTGTTTAAAATGTAATGGTAAAAGAAGCGTTGTGGGTAAGTTTTAAAGATATGATTATGTATTATTGTTTTGGGTTCAAGAGTCAAAGAATTCTTTAATTTGGATGCTCAAAAGGGCATCAACAACCTGTTTCATAACATGACAAGTCGAAGTTGGCCCCCACGCAATTTTCCGTGAAGAAAGGAAACAGATTTGTAGAATGTCGTCTTTGCTGCAAATGTGGTTTTTATGAAATTTATAGGAAGATATTTCAGCCGCAGAGCCAGTTTTTATTGGACTTCAGAAGCCGATGTTGTGATGAGTAACC

>chr24:+:52871534-52876004

GAGAAATATGGTCCTACTGTTAACATCCATTCTGTCGGATGGCTGGTAACAGAAGAGCCAAAAGCAGCTCTTTTATTTTGTAAAAGAGTTGAGGTTCATATAGTAAACTATTCACAGAGTATATGTATATAAAATGAATTTGAATTGTAAAGTGACAGTTGGAGACACACAGTTAGGACATTCATGGACTCTTGTATAAGGTTATTTTATTTGTTACCCTGGTATGACATTGTAAATTGTTAGAATGTAATATATTAGATTGAAATATTGTCAGAGATTGTCATTGATAGTATATCAGTAGTCGTTATCTTTTGTTACATAACGTTACCTGTTTCCACAAAAAGATATGCTGAGATTGTGTTTGGAATGGTTCTTGAAGGTCAACTTGATCGATCATATTACAAATTATGCAAGTTTAGTAAGTAAAAAAATAACAGGGATGATTCACGGTATGTTTATATGACTGGTAGATAGCCATTGACATTTGAGATGGCATTTAGTTTTGAGAAGTCATTAGTTAGTTTTTTTTTTTTTTTTGTATAAGGGACAACACCTATACAGTATTTGTTTCACTTGAATGCTGTCTTCCACAGAAGTAGGATGGTTTGTGCAAGTCTCTTGTTACATTGTTCTTTCTTTTGTACTGTGTTAAAATATGCTTCCTGCTTTACTTGGGTAGTATAGGAAGAACAGTCTAGTTAATTTATGATTGGTATTGTCCTCTTTTGTTCCTATTGTTGTTTCTCCCATCTTTTTTGAGAGATTTATTTTTATTTAGCTGTGACGAATGAAAAATTAGTTGCTCTATGTTTAAAATATTCAGCATTTTCAACAAAGACATTCACCAGGACTCGGTCCTGGAGGTACGCTTAAACAGGCACACCTTAAATCATCCCTCTTTTTAAAGTGAATTAAACTTGATTAGGGGCCTAAGTTCCCAACCATATGGTTATCTGGCCAGATTTGAAAGTATGAAATAACAGTGGAAAGATCTCATCTGGTCATTGTCAACATTCCTTCCACTCTGTCTTTTGGCCATATCACAGTAAGAAGATAGCTTCCTTATGCACATAGGTTATTAGTGGTGGTAGTAATGTAGATAAAATTGTGTAAAGATATTACCAACACTGATGGCTTGAGGAAGCAAGCAGATTCAGGTAAAGTGTGTGACAAATGACGTGTACTTTTGAAAGTCTGCTGTAGCATCCCATTGATCAGTGTATTGTAAAGATAAACTTGTGCATTGGTAGATCATTTTCATTCCTCATGTGAAAATTCATTGTGTATTATAGAAGTGTGTGTGGAGACTGCGAGATCATAATATCATTTTGTTATTAATGTTGTCATTACTATTACTGTAATAATTTTTGTCGAGATTACATCTTTCATTCATCTGAAGTCAAATCAGAGACTTCAGTGGTCCTTTTTTTCCAGTTCTGTTACTTTTTCATCCTTACCATATCATGCATGAACCTTGTCATAACTAAGAAGCAATCTATTCCCCCCCATATTTTTGTTGTTGTTAATCCCATTTTAATTTAATATTCTCTTTATGTTGAACCAAATGGGTAGTGGTCATGTTTACTGCTATGTTCAGATTTTCCTGTCTATTCTCTCTCCCCTTCCATGCTGGACAACAGACAGGTTATTTATGAAGCTTTGATTTGCAGAGTTCAAGCTCAATTTTTTCCCCTCCAAGATCAGTATCCTTGTAAGTACTCTTAGTATTCAAGAAATCTTGTGTTCTAAGGTCTTCTCTATACTGAATATGGAATTGCACACACAACCATGAAATAGTATATTGCCTCTGATGCATGGTGTAAATCTGTAGGTTATTGCATCCTATAGTGTAAAATATACATATAATTCTAAGTTCAATTAGGGAAAAGTTATGCCTTTTCATGTTTACGGAAATGTTAAAACCAACCTTTTAGTTTCCACCATTGTTAAAAGTAGATAAATGAAAATAGATGAAAATGGTCATTTGGATGGAGGTTGTTTTACATTTGAGATTTTACCCAGTTTCACCCTTAAGCACTGACTTTCTTGCCTTGAAGAATGGCTTAAGTGTCATAAATGTATTTAAAGTAATATATTCTATATTGATGACTTGGTCTTAAAGGATATAATCTGTCAAAAGTACAGGATAATTTGGTTTTGATATTTGTCATTACTTAGACATAAATGAGTGACTTCTAGGCTGCCATTAACAGCTTTTTCAAACTTTTTAATAACAATGCTATTTTGAGCATCATTGTAGACAACCTTTGTATGACATGATGTCACTGAATATTTTATTTATTTTGGGACTCTGATGTACAATTTCTGATCTTGTATTTGTTATAATTTGAAGCTGGACTTTAAGTTAGTATGTCTGAGAGATTATATTTTCCCCCATGGAGGTTTTTATAGTTCATGTTGCCTTCTAAAAGTTTATTCTTGTTGTGACTGTGGTGTCAAAGTGACCTCTTTGAGATTCCCCAAATTCTTGGTAGGACCGGCATCGTACGATCTTTTTGGAATTGTTGTAAGAAGCCAATTAAGTATTTGATCACCAGATACTATTTTCCAAAGTCCTATCACTAGGGCTGGTGCTTGAGGGTGTGTTTCACTCCCCTCCCTTTTTATAACACCAGGATTCACATGCATTTAGCTGGTTTGCATATGCTCCTGATCTTAGTTTCTTTTAAAATATTTTTTTTTTATTTACGATGTAATTTTGAAATTCAGTTAGCATTAGAATTTGGTTTCTTTCTTCCCTTTGAAATATTGAATTTAATTAATAAATAGCCTTCATAGATAATTTGATTTATGTAATGTATATAGACAGTACAGCATATAATTTTGTATTTTGATATGTTAGACAGATTGGGGTTAAAGATTTTGGGTTGTATATTGTGATAAATAGCTGATAACCTTTATTTTGAGTAGACATTTTTTTTTTAGTTTTCACCACTGCTTAGTAAAGTAATTTCTTCAGCAACTGTCTTCTCATGGAATGCCTTGAGTGTCAAATGAGATCGGCTTTATTCACATAATAATTTTGATATATGCATAGTTCCTTAAATTCATTTTTTTTTTTTTTTAACTTTTTCCCATGGTAGTTGTATGATACAGAGGCAGCTATCTGAGGCCACATGCTTGTGTAATCAAGGGTTTCAATGTATCTAGGGAGCTGCAAGTGAGCAGTTTTGTTTTTGATTTTTAGGTTTGGAATTTTCTTGTCTTTTCCTTGTCCTTGATATTTTTAACTGGACATTGAGTTAACAGCACACATTACTTTTAAGACTTAACAAATAAAATAATTGAACCACATCAGTAATACTGTATAAGCATCATCAGTTCTTGTGACATTAGATGCTGCGTGTCTAATCTACTTTTAAAAAGTGAGAAATGGGGTTTTGAATGGGTTATTTCTTTTAAGTATTCTGTAGACATTCCAGTCAGGCACGTAGCTATTCATTTTTGGCTTTACTCTAGTACTGGCTTCCTCAATATAACAAAGTGCTGTTAACCTGTTCTTACATTGGATGAGGTGTTGCTGTTTTCTCTGTTGTCTAAGGTGATGCATTAGCATATCTGAATTTTGAACCATTGAATGTCTTCAGTATAATTTAAAGCTGTGTGAAAACACACCGAAGAAACCAATTTAGTACACGAACAAAGCTCTTTCGAGTTGAAGATACAGTAATCTCAAGGAGGATGTTAACTAGTTTTACATTGGGTCTTTAATTCTGGTCTTGTGTAGCATAGGTGTGAAGTGTCCCTTAGTAAGGTATGCAAGGGTATTTAGTTGTAAGTGATGGCTCAGGAGGGCGACATAGTGTGTTCGTCTGCCTTCAATGCATCTGCCTGCACAAATTTGCTGGCAACAAATGTTCATTGACATTTAAGTCCACCTATGTTTGACATGATCTGGAAGAGACAGATTGTAAATACAGTAAAGTACAGTAAATCATTGAATCAAGTGGGTGTTGAAATATTAAAAGCAACACTGCAGAATTCATTTTATGAAATGTGCAGTGTGTCATCCAAAGGAAGAAACATTCGCTTGACTGGGATTTCCTATCTTTCAAAATTATGATTGCCGGTATTTTGACTTCAGAATAGGTCACTGGAAAGCCTCAAGAAAACAAGAAAATATGAAATAGATCAGTGCTTTGTCTTCTGCATTTCATTAAAACTAGTATTGATATGAAACTTATCTACTGTTAGTGAGGTGTAATAGTTAAAGGGAAATTATCATCAAAGTATGTTCTAGCATTCGAGTTCTGGCTCAATTGTATTAGGAAAAATGAAAAGACTGCACAATTAGCAGGTGATTGTTCATTATCTGGAATTGAATGCTTGGTATGTCCTCATTTTATTGCCATACAACATTGTGTTTTCTGGAGATTTTCCACTGATGTCAGAAATGTGTTACACACTGAAATTATTGTAAGAAATGGTTATTAAAATAAATGTGTATATT

>chr42:+:12701951-12702211

ATGATGCTCGATTTCACCAGAGCTCTCTGTCCCCGGAAGACGCCGCCGCCATGAGACTCGACTTTTAACGCGGAAGCTTCTCTTCCAAGAGCCCCCTCGGGATGACTTTAATTGGATGGAAGGCAAGCAAGCGCTAAGGAGAAATAATTATACGATGCTGAGTTACAACAAAATTCAAATACAGAGATGATGGTTGTGACACACACATTCAGTGAAGCTTCCTATTCCGTAGAAAATTATTTATCTTCATTCCACCTGCAG

>chr42:+:12705856-12707762

AGGAAAACCCACCAGGGGCGAAGAGGGTCTGACCATTATGCAAATGGCGCAGCCAATCACGAAGCATCATTCATCAAAGCCTCACCATCTTCGGCCCTTATCCATTTTACATCAGCTACAGCAGAGAATGATATTCATTATACATTTACATATTCTGACAGAGAGATTTTTGCCTGTAACGAAATAATACAGCCCCGTCTCATCGTGTGATGGGATCTGAGCGTCAGGGGGCCATCTTCCGATTTTTGTCTCTGGGGTCTTCGTTCAATTCCACCACCCGATCACAGATAGTCAGTCTCATAACATGACGAGAAAATGTTCCTCTCCACGTGGGAAAATGCAATTTGTCCTAATTATTGTGGTGGGATTTATCCTCAACTTGTGATCAGATTTTTCCCCTCCAACAATTCGAGCCCTTCAAATAATAGGCCTAAAACCTTTTCTTTTTTTATTCATGCAAGTAAAACAGATTCCTTCAAGACTGGAATATGTTTAACGCCACGGATTTTATTCTGAAAGGTTTCTCTCTTCCCCCGTCACCTATTTTGCTTTGCATATTCTATGACGTCATTAGTCCTGTCTTTTTTTTATTTAATATTTTTTTTAAACTCTGGATAATACTAAATCGTTACTTATATCTGTTCAACTTTATTTCTTAATCGATTCTTATTCATGGTTGTTCCAGTGCATCCATTTTTGCTTTTGGAATGACACTCTAATATTTGTTTACTGAATGTGTGTGTGTGTGTGTGTGTGTGTGTGCGCGCGTGTGTGTGTGTCACTGACCCGCGGTCTCCCTGTGAATGAAGCCCCATTGCTCATTTCATTCTTAAATTACATGACAACTTTTCGCAAATGATGACGACGTCGCTTATTTTACTATCCATTGGAACTAGTTTTAAGTTTTCTATTTCGAATACTATGAATGTTGAACTTATAATAATATGTGCTGTCTAAATGTCCCTATGAGGCTGGACATTTTATTCCTTTTAATGTAGTTTTCGTTTTTGCTTTCCTGTGTTTAAATCGCTCACACAAAATATGTCACTGTTGACAACCCTTATCGTTTATTTGCTTCACTTATCGAAAATGGAAGTATTTGAATGACATTTTATTATGTAATTGTTCGAACACGGGCTAAATGCAACCTGTGATCATCAATAATTTGATCCATGTACTTATGAAGCCTAAAGATTGATATAAATCGCATAATTATTTCCTATCGACATTTAGTTTCGTTGACAATGGCATTATATGTACTAGTTTGTTTATGAGTCTTATGTAGTTATAAAAAGTCCTATAAAAACTCCACTCTATGTCGTTTCTGGTGCTTTAGGATTAGACTGTCTATAAAAATAAACTTATTTTCACCATTTATGGATAAATATAAATTCCTATATCACATTTACGTAGCTTTACATTACACACTTTTACAAGCAGGATACAGTCAGACGGGATCCTTTCCTCAAAAGTGTCATAAAGTTCCAATCGTATTCTTTAAATTGTTATTCTCTGCATTATCCTCACTGTCTTTTTGGTTGAAGTTCTTCATACATTTTTCATATCTACGTATGAATATCTGCAAAGCTGTGTGTGAGTTTGTAAAGATAGGTGTGTGTGTATAGTATAATAGAATATAATATATAATATACATAATTAACAGAAAGGACTTCAATCAAGTCACTTGCACATGATTTCGTTACTGGTTAAAATGAAAACTGAAACTCAGTGAACTTGGACTTACAATGCTATTGTTAATGATTTCGTTTTATTTCTAGTTAGTATTTTTTTTCATCACATGTTCGTATGAAAACCATTCTTAGTAAGCCTATTTTTTCCTAGTCAACGTGGAAGATTTTTCGCTTCTATGATATAATTTGTCGCCAATAAATTGTATTTTTGTATAT

>chr2:+:158920590-158923656

ACTCACCCAAGGTGGCTGTCTGGCAGAATGCTACCCATAAAGCATACTGGTCATTGCTGAAAGTTACTAGTCTGCAGAAGTTGGCTGAATAACTAGAATTTAACTTATTAATCCAGAGGAGTCAGATGCCAGGTTATTGGACTTATACTGAACTCCTTATTCAACATTTAATTGGCATCCTTTGGTAATGTACAAAATTTTGGCTTACACAAGAAAAGAAAGGGATGTTACTTTTACCAAAAGATTAATACCTGATGTTGTTATACCTAGATATTTACATGCTCATAAAAGTTTAATTATAATTTTTTATGCTATACTCCCTTGTTCCTCTGAGAGGGTGACTCCGGATGTGGTAAACCTTCTTAATTTCAGCAAGCCTTTTTGAGATTAGGTTTCCCACCGCAGCTAAGACCCACTCAAGTTTACCAACCTTCAGGTATCCAACCACGGGTCAGTAGGGCCGCAGTCACTTTTTCATGAAACAAGCCAAGCTATTTGGTAATAATGGATAGGAAGAAAACTAGAGGAGGTGAAAGGGAAAATCCCAATGTATTACTTACAAACAGTCTGGTGACATGAACTCCAGAGAGGTGTTCATACCACCAATGCACACCAAAAGATAGATTTGTCCTTTATGCCATCTTGCTAGTAATTTAAATTTACTATGCCACTTCATTCTGAAGTCCACTACAAGGAGTTACCTTTTATTTGGTTTTCAAGACCCAAAAATGCTATTTTTGTATACTTCTTTAAAAACTTCATACTTGTATTTGCAAGATGTTAAACATCAGTCCGTTGAATATCATGGTGACCAGTGTTTTTGTATCTTGTGACTGAGGCTATGATAAATTTATGTTGATATTTTAGAATGAAACGCTGACCTATATTGTAATTGCGTGTTTATGTATTTCCTTCATTTGTAGAAATTCTTGATCCTTATATGCTCTGGACATTCATAGTGATAATTTGCCTGTTCCTATTGCTAGTAATGAGTAGTCATTTAATCTGTAGTAATTTTTAACTTTTGTGACATCAAAGTAGTTTATAAAACTGGTTTTGAAGTTTCAGTGTTATATATATTGTAACATCAATCAGTGAAAAGATTTCTTATTAGTAATGGTAATGAAGACATAATGGTAATGAAGACATCATATTGTGCTTGGTAATTTGTATTATGTAAGAAAATTTATTTTTCATGTACATTTGTGTGTGATACCAAAAAGCCTTGTGGAAGGTTTTCCATTGCATGTAGGTAATGTGAGGTAATGTGTTTGAAAGTAATAAATTTCTTACTTTCTAACTGAAAATGTTATACAGTGTAGTTTTTTCTGTATATATGCTCTACATGGTGTCATGTGGTGTAATTTAAACAAAAACAGACTTAAAAGTTTAGAGTCCATGGTAAAATTAAACCTTATTTTTTTATAAATACATATACATTCATACATACACTTACACAAACACATGTGAGAGATGCATGCAAGATAAAAGTGAATTGATGAGTGTGGGTAAGGGTGAGTCTTCTTTGTAGGGGTATGGAGACGTCTCTTTGCCAAAGAAATTATCATCAACCATGGCTGTTCCACTGATAGCACAATGTCAGTAAAATGTAACGTACACTACCAGGAAATGTCCAGAACACAGTTAGTATTACCCCTTAACCAGCTGAACTTGGAATGATTGTCATAGTAGACAGGCACACTTGTCCAAGTAAGTTCTGTGACTCCTGCAAAGGATCATATATCTGGATGATAAAGATTCATCCACTGAGGTATCTCATTAGCCAACCATAAAACAGCACTAAGAAAAACACTAAAAAAATCTGCATGATTCCATCTCACAAGAAATCTTGTCACTGGGATGGAACTCATGAAACATGAGACTAACCTTGGCTGTCAGTCCCTAGTGGAATCCCTCTTGTGTCACGTCCTCATATTTGTTGAATGCTAAATCATCTATATTTTTCATGTTTTGTCTTTTGGCTCCCTTTGCCCTTTACCACTTTACTCATTCTTTCATGCCATTTTTTATGCAAAATCACTCTACCTTTACCTATAAACTTTCATGGCTAGCTTATTGGCCCCAGTCATAAAGAGTAGATGCTCCTCATATACTTACTCAATGTAAATGTTTATAACCCTCACCTATTTTTCATTTCATTGTCTCTTGGCATGCTGTCTTACTTTAGTATCAGACTCACGTGTCATATTTGCACCAACAGACACTGTAAATCCTTACTTACTGCCTTTTGGAAACATCCATTTTTACTATTACTTTCAGCCCAGCGACCAGCACATATAGGTCATTATCATCTTGAGAAGGGTTTCATAACTATAGACTGTATATATTGCTAATTATGTTTATGCATAAAGTAACTAAAATGCTAATGTGTTCAAGTTGGAAGTGAAAGGACTTTCATTTGAAATCTGTTTTTGCAGTGATAATTTTTATCTACTGAAGTGAAGCATCATAATGTTTTTATTGTGTATACTGTACACTTAAACTAGTAATATGCTTAATTGTGTGATTCTGATCTTGTTATTCTAGTTAGATTTGTTTAATCTTGAAATCAACTGTTGAAAAAAAATTTTGAATTTCCCTTTCTATACCTCTACCACTTTGTTTTTTTGCCATTATGTTTTTTTTTTGTTAGTCCAGTGTACAGTGCATGTTATTGGGTTTTTCAAAGTATATTTTGTTTTGATTCCAAATTCAGTAATGTTTCTAACCTTAGAAAGACAAGTACTTGTGACTAAGCTTTAATACTTTTGTGTTCCTTTATGTTATGAAATAGAGTAGTTGATATGTTTTGTATATTACAGTACATATTTTTTTTTCTCATTTCCATTCACTGCACAACAGAAAAGATATTTTTGGATCAGAGTACTTTGTGCAAATACAGTGTTTATTTTTCCAAACATTTACTGCAATGTGATATATAAAATTAAGAATGGATCTCTGAATGCCATAGTTATAGTATTATCCATATGTATTTAGAAATTCATGCTGGAAGTCCTTATTTAATAAACATGCTTTATTAAAAAGAATTTGATTTAAGCAACAATCT

>chr17:+:58561591-58565108

GGGCCAATGAAGCATTAGCCAAAATTGGCCGAGGATTGATATCTGGCAGGGCTATTCTTGAATTCCCAGAAGAAGATGAAGAAACAGAAGAGTAGAAGAAGATGTGAAAGTATTTCAGTGTGCAAAAAACCATTTGACAATTTTTCTGGGCATTTTCCTAATTTTGCATAAAAGCTTGGGGTGCCTGTTATGTCTATACATCTCTGAATATAAATACTGTTTGTGTATATGTGCATTTAAACACTTCCATAATCATACTCAGCTGGGACTGAAGAGAGAAGTGCTTTTCTTGGTTACAACATAAACCAAGATTTAATCCAGAGAAAAGCTACAATCAGTTTATATAATATCGTTGGCCCTGTTATCTTGTGTCTGTGAAGTACTTAATTATAGAATTTGCAATTATGCATTGCTCTAAAATGTGATTGTGGGCAACAGTATTCATTTGCACATGTGTGCCATGGCAACATTGCAGAATGTTGTAAGGGGCTGTAGGAATTGCACTGTGAGCAGATGTCAGTAATATTCATACAATTTTCTTTATCATTATTAACATCAGGTGTGATGCAATTTTTTTCAATAAGGTTATTTCAGTGCTTTAGGTCATGTGCAGTCAGACATCATAATTGTACATTTCAGCACAATTCTACAATACTGTAAGGGTAAAGCAACCACAAATTTTTGGATTTTGATATAACCACATCAAGTACATATTTTGCAAGTTCCACATTTGTATTATTTATTTCAAAGGCTGTGATATTTAGGCATTATAAATATGATCAGAGTACCAAACATAGTGCTGTTCATTATTTCCTACCATCTTTATCATAAGAAATAATGTACCATCAGAACCATGTAATCATATTGTACAAATAACCCATCTACTAAAAACATCTTACCAAGCTCAGTAGAAGTGTACACCAGATTCTTGACATGAAGTCAGATCTCATATTAATGGCCTCAATATCTGTTGCATGGATTATGGTAATGAGAAATTAAATCCCAAAATGCATTTTAAACCCTTTCAATGTATATTGTACCTGTATGTATTTCTACTGATATCTTCTTCATTGTGTTCAGACAACAGCCATCATAATTCAGTTTCCATCATGCTTCCCAAAAGATGCATTTTATAAATGTACTGTTGTAATTGACTGTTGTGAAATGGAGTATGTGTGATGTTAAAGACACTCATTATAATGTAGAGTTGCTTAAGTCACTGCTGCCTTAAAATAATAAAAATGCAGTGTTAGTTTGACTTGCTTACCTCATGCATCCCAGGAGCCAATTTTTATACCACAAAATGGTGAAAGGTATTTAACAAAGTCGCTTATGAACTTCGTGGAATTATGAGGACTATCACTTTCAGATGTGTGCACAGTCTACACAACATTATAAACACCATATACACACACACATATACACCTCATTCATAAGTGAAAATATATTATATATAAATTATTTATGAAGTCACCTGATGCTGGATAAAGTTTCAAGTGTTTTCCTAGGAACAATTTTGCCATTTCTTATTCATGTTCAGGAACACTGCAAGACAGCGTAAAGTGAAAATGTGTATGTGAATATCAACAACAAACTTCCAATTCATAGAAACCCAAGAATATTTGGTGATATGCCACATATATTCTTCCACAAAATTGACAATGTAAGTGGATAAGATTTCTAGTGAATTTATAGACCATAGGCAGATCCACACATGGTAATGCTCTCTAATTACAAAGAACCCTTTACTTTCAATAACAGAACTCTGCAGTTATCCTGAACTGGCAGTTTTTGTTTGATAAAAATATTTACATTATCAAGTATAACTAAATGTGTGGTACATATATTTTATCTAATAAACCAATACCTTATGGTGTACTGTACTTTCAGATCACTAATGATATAATCAGGACATAATTATCAGTTTTTCATAAAACATTTGTGAAAAAGTAATTTTCAGATACAGCTGGGCTAAGAGATAGCAATCAAGCCAAACATTTTTTCCTTAGAGTTACAATCTGTGTTGCCGTTTTGAGGAATTTTGTCTACTAGTGTGGCTTTCTAGTGATATCTGGCAACCCTCTGTGGATATTCTGACCACAGCCATGGCGCTGAGGAATAAAATTCTTCACTTTGCCTTTTAAATATATTGTGAAAGTATGTAATCCATTTTGGGGTTTTAATTTAAAAAGAAATAGAATTGTAGGATCTCATTTGAAATAGCAAATTAGTTTACCATAAACATTTTCTTTGAAAAGACAATGTCAAAATGAAAACAATTTAAAGTAAACTAGTCTACCATAAACTAAGATTTCTTTGAAAATCGGAAGAACTGCTTATGGTAACACTGCTAAAAGCCCAAGTTGTGAAGAAAAATGGCAACGCTGGTTAAAATATAATTTGAGCTATCTAAGTAACACTTCATGACTGGGAATTACATTTTACGAAAAAGGTGACATTAAATGAAGCATTATTAGTTTATATTTTTTTCCAGGAATCATAAAATACTCGTAAGTACGAAACTTGAATATTAGTTACTTTTAAGTACTATGTGTAAAAAAAGTTTTTTCCCAAGTAATGTGTACAGTACTTGTAATTTTGTTTTAACTGTGTGATCTGTACTGGTATCCTTACGATTGGTCAGTCTTCAAGCTTAAGGTACGAATTAGCAAATACAATGTGATTTCATTTGTTTCAATCATTCCTGTTACTGAAGCTTTATTTGTGAAAGCAAAAATAGAGGAGTAAATATGTCTAGTTTTTTAGGTGTCAACAGGTTCGCAAGAACAGTGTCAGTGCGAATGTATTGATGGCTACTTTATTTAAAAAATTTTCACACTGCATTGGTTACAGCAATGAGGCAAACAAAATTTGTTTGCAGGCAAGAAAGCTCTTAAACAATGATTCTTAATTTCAGGTTCATGTTGTGGATAGCAAGCTATCAAATAAATTTGTATTCATTGTTATCATAGTGAAGCTGGCTATCACAGATGATTGTGAATATTTTTTATTTTAAGGGATGGTTTTATCATAATTTTGTGGGTGTAGATACAATACAGATGAAAATGTAAGCTATGTATAGTTATACCTTATACACCCCACATAGAACTTATTTTTTTACATAATTTTCTTGTATATATACCAATCACTTGCCTAGGTCTATGTAAAATTATATCCAGTACATATATATACCAATCACTTGCCTAGGTCTGTATGTAAAATTATATCCAGTGAAATTAACTGAATATGAAATGTATCAAATCTGTATAAAAGCATAAATTATATTATCAAACAACACTTATTTCATGGCAAACTTATCCAAGGAAATTTGTATTTTGCTGATTTGTTAGAAATTTTTATTTGCCTTAGGATAAGATCTGTATATAGCTATCTTTCTTGTATGGCTGCTGATGTAAACACTGTTGAATTGTATAGCTGCTATATGTGAGTATATTTAAAACTAGAATAGTATTATGTACTGTATTATGACTACTGTAAGCAGTCAATTAATAAAATTC

>chr14:+:26881464-26883929

GCATCACTTCTGTAACTAAAGAATTTCAAACATTTAGAATGCTCAAAGAGAATCTGATAAAGAGGCTTTTCAAGAAACTCATTTAGTAGAATTCTCTTTGAATAATTAGCAATAAACAACCACCCTTCCTTTACAAATGGTGACATGTGGTAGACCTCACAATTTCCCCACATTGTATTCTTGAAGGAAGAAAAAGGCCTGGGTACCCTTATTACGTATACAATTGCCAGTGCATTTATTATCTTGCAGTGCAGGCTTGAAGTGTCATCATGGAAGGAGGTTTATTTCTTTTAGGTCAAAGTTAAAACTGTATCCCTTAAACTTAGTAAGTTCATGAGAACTCTGTGACTGGTAGGCAGTTGTTCTGTGTCTGTTAGGCAGTTTTGTGATAAGATCTTGTACTTACTCGGCTTTTCCGGTTACCAGTTATGTGAAATAATTACTTAACCATTTGATTTTCATTAAGAGGAATTAGTAGATGCAAAATCCAGAGTTGTTATTAAGTTGTTATCCCATTTTATTTTAACCAAGAGTGTGTTGAGGCTTTCTTCTCCCATTAATTAGGTTTTTGAGGTCATATCTAAGAAAAGGAAACTTGTGTGTTTACATCATTCCATCTGCAGGTTCTCTGTTGATATCAGTGATTGGAAGCACTAGTCATTTGCTTTTGGTCAGTGTACCATACATTGACTATGGGTAACTGCTTGAAGTAGAGCATATGCACCATTATTTTCCTCACTTCTGTGATCTTTTAGAGACAGAAGAGCGTACTTCTCACTATACTTTGTATTAAATTGATGTGCCTTGCCATAAAGAGCATCTTAAATAGTAAGGTAAATGACAGATGACATCATGTCTGTATGGTGAAAATGGTTGTATGTATTGTGGTGCTGTGGTATACACACACAGGTGATTTTGTTATAATGAAGATGGCACAGATTCTTGCTGCCTCTGCAGCTTTACTTGCTGGCCATCACGTTTGCTACTTCTGTAGCCTTTAGTTGAGCCCAAGTCTTATCAATATTATTATATGAAATTAGTGTAATGAAACATAATGTCACGTTTCTAGACGTAAGGCCCATCAAACAATTTTTTCATGTGAGAAATGAAAATCGAAGCAAGGAACATTTCAAGAAGTTGGGCAGTTGGGTGGGAAGACAACAAAGGGAAAGGTTGAAAAGTAAAAGGCAGAACACTGCAGCTAGGTCTGAAGTGACATTGCAAAGAACGTATGTTACCATATGCAGTGTGCCATGCAAGGCGCACAGTTGCCTTCCAACAGGATTCCAATCATTAAGTTCTGGCATTTAACATTTCAATTCGAGCAATCCTTTTTGTCTTAGTAAAGACATCAGTATTTTCCTTGCATACATGAAGCGATGTGCTGTACTATACTGCATTTCAGTACTTAATGGTTTATATTTGTTTTGGAATGAATTTATAAAACAACAATTTTGATGTTTTTTCCAATATACTAATCCATATTTCACGTAGTGTACCGTCTTCCATCCTTATAGGTCATAGTCATGTGACAGTATATGAGGGGGTTATTTGTCCTTCACTCTTTGGGCCAGCTCATTCTCACTTGTTGATTTTGGTTAGTTGCCTCACAATGGTTATCGAGATTGAAATGTTTTTTGTTTTCAGCTAGACACACATGAAGTTACTTTACGTTGCAATTAGTATGTTAGGAAACCAGAATTATTTAAGAATTGTGTTTTTTCCAGAGGTAAAGGGTTGTGAACCTTTGAGACAGGACTTGATGTCAGGTAATATCAAGCAGTGAATGGATGGAACTGAACTTGATCAGAGATAATGATGGAGTTGTCTCGCAAATAACACGGAATGCTACGAAATTTTGGCTGGTAGTTTTGTTTGTAGATAGCAGGGGATGGAACACTTCCTCCACTTTTAGCTTGCTTGAGCATTTTTTAAAATATTGGACTAACTACCTTTTACAGTTTGAAGGAAAAATATTTTTATCTTCGTGTTTTTGACATCACAGTTCCCTTGTTTTGTCTCTACTTCCTTTCAAGAATCTGACAATTTGATGGATATTGCAAAGCATATATGGTAAAAATGCTAAGAATGGCATTGTGTTTGTATTTGCATTTATGAAAGAACCAAAAAAGCAGTAGAGTTCCCTTTTTGTTTCTTGAAGTTGTTTACTACAGTGCAGCCATGAATATATAGTTGTTTCAGCTCTGTAAAAACTCCTTGAACCTTCCAAAATCATTTGTTGAGCCACTGCATCCAATTTGACAGTTAATAGTTATCTGTAGTATGAATTATTTTGTCTTGTGATTTATTGAGTTTCTTTTTTTTTATTTCACAAATGTTAAACATCCTTTGCTAAATATGTTTTATCTTCATTGCTTTATATTTATCAAAAACATTTACTAAAGGATTCATAATACGTACCTTTATTAATGTTTTTCTTTTATTTTTGTGAAAATAACAAATTTT

>chr18:+:49340435-49342359

TCTCCCATGAAGTACAATCAATCTATTGTGATGCTGTAGGAATAGCTTCCTTCCTAAAATTAAGTTTTATCTATGCACAAATTTAAAGTTGTTTGTCAATATAACTTGGTGGATTCCAATGACAAATTAGTTGAATGCTAATCCACAGTTTGTAGTGTACATTGTTCAGTATTCAATTTATTGACATGATCAATTAATGGCATCTTTGGTATCTTGGACAGTTCTTCTTTTGCTAACTAGAAACAACTTTGTCCCTTTTTCAATAATAATACTGTATAGTGAAAAATTACTTATTCATTAAGTTCACTGTGGCACACTTTTCAGGAAGCTACCCATTTTTTTTTTTTTTTTGTTTCAGAGCCTTTTAGACAGGATGAGGGTAGCCATTATAGGTCAGTCAACCTTTGCAGGCGAAGTTTACAAGCAACTGGTGAAAGATGGCCATAAGGTGGTGGGCGTTTTTACGGTACCAGATCAGGGAAACCGAGAGGATCCATTGGGTGAGTATCCTGTTAATATGAAGGAGTTTATATATTCTGTATCCCACTTATTTTTCCTTTTTGGTAATACATCTGCTGCTTTGGTAAATTCATTTCATGTTAAATACATGGAGCACAGATTTGTCTGCATTACTGTTTGTGTGTGTTTTATGCATTTGTCTATTATTATTTAGTTGGACAGCAGGAATGGGAAAATAAAGTTTATTGCACCAGCAAGAGTGGTAAAATATGCACTCAAGAACAAAAGCAGATGGAAAGTTTTACAATTGGATAACCCTGCTGGGAAAAAAATCAAAGTAATTATACATTCTGTGATTGAAGCGCCAACTTTGGTACACTTGTAGCAAAGGTATTCCTTAAAAATGTACATACCATATGTGGGACTATCATGAATTTTTGTTATGTTACCCATGTCAGTCTTTTTCAACATAGTATCTGCAAGCAGGAACTTTCTAAGTCAGCATTAATTTCAGCAAAGGGTATTTATGATACTTTATTTATGGGATAGATTTATCATAAAATTTCTAGTGAATTTTAATTCCATAATTCAGTTGTCATTGGAAATTCAAGTTGAGTGCTTGAATGCTATGAAATTTGGAATTTTCATAAAGTTGTACTGAGTAATCCAATTAAGATTTTCTCTGTTCAATCACTTTGATTTTGTCTTACAGACTTTAAAAATACAGTATAAGATTTTAGGAGTGATCAGCTTCCTTTAATATTTCTTAATTAGCTAACAATTCCCTCATCAGAACCCATCCTCTCATCCCTCTTTCAGGATGGAGAAGGATGACAGGCTATATAAGAGAATTGCTGCTGGATTTGAAAGTTTCCAAGTGACTTTGTGATATTCCTTATTACATGCTGTGTTTAAGGGTACAGTCATCACTGCCATTTTTATATCCCATTTTTATGGGGTCTTTTGGCATACCTTGTATAAGTAGCAAGCCTTAAACTTTCATCCTTTATTGTATTTACAGTTTTAAAATTTTGTTGGAAGTATTATTTAGCACTCTTGAAATGGTGGAAGGAGGGCAGAGGGAGACATGGCACAAGTTTCTATAGGCTCTTGGCTAGATAAAAGGTTGCCTGAGAAGTTTGTAATGCAGCATATGCATTGGCTGGTTGAGGCTATTTCGTGTCTACGAGACACTCCTGTTGTGTGGAATGTGTAACAAATTGATATTGGTAGCAACATGGCGCACCTTTGTGTAGTGGTTTAGCAAACAGCCAATGATCTTGCTGGGAGAGTAAAGGAGGTTTGATTATTCCCTTCACCAACAAAAGAAGGAAATTGCAGTGCCATGGATAGAATCCTTAGCTGTGGTTGAACTATGTAGTCATGGAGGGCTTACTGCTTTTTCCATTTCAAATTGCTACTAGATGTTCCCCATTAACTTGAGAATACAATCATAGGATATGACC

>chr14:+:33246749-33248058

GGGGCGCCCCTGGTTCACAAGGACCCCCAGGACACTGCGAGTACTGTAATCCAGCCTTGGCCTATTCCCATCAGCAAGCTGTAGCCGGCAACGTCAAAGGCCCGTGAGGATCCTAGTCCAACACTCCCAAGGATGTCGTGACATCCTTCAAAGGGAGAGCGTTCTTTTTTTTTTCAATTTGCTGAGGTCAACGTAACTCGATAAAAAAAAAATCTTCGAAATGCTTTTAGCTGTCACATCAGTGTAGGAAAAAATTCCGTCCTTCTTACTCCAGTCTGAGCATTATATGAGAGCTTCGTGTTTTCACTTTTCCTTTTCTTGATATTGCGAAATAAGATTGATTAGTTATTGTCATTACTTTTATATGGAACGTCTCTCAAACTTACACTAGGAATGTATCGGTGCATTTTTCTAAAGTCCCTCCATATTTGATTGGCTGGAAAGGTATACATGAGTGTTTAGATGTGTGTCAGGAGTCTTACTAAAAATGAGTTAGGCAAACCAAGAGAACATATCATGAGGAATATATACCCTCCCCACCTCTCTCCCTTTACTAAGTAGGTCCCAAAAAACAGACATTGATTTAATGTCTCTTCCTCAATCACTGCCATAACCTCTCTTAGTTTGTTTGTGCTGAATGAATGACTGATTAGTATGTTAGGTAGAATAGGAAACGTTAACTTAGAATAATTAATATCAAGTTTCAGAAAATGTAACACGGGCAGTTGAAAACCAGCGGATAATCTAACTTTTTTTTATGTTTTATACAGCACATTTATATCTAATACGTGCATACATACGAACAGACAAGTGCGTTTGCTTATTTGACCGAAAGTAAAGCAATGAGATTCCCGGTAATACAAACGTATCATCGGCATTAAAACCAAATACATCCATTAAATTTAAAGATACTCAAACAATCTCTTTAAAATTATACATTTTACGATCAACCAGATCCTGCCTAGTGCAACTAATGCTGACCAATGGTGCTATTACAATGAAGAGAAGAGAGGAATAAGACTGAATGAAAGGAGAGAGAGAGAGAGAGAGAGAGAGAGAGAGAGAGAGAGAGAGAGAGAGAGAGAGAGAGAGAGAGAGAGAGAATAAGCTTAATAAGTGAGGGAGAATAAGTCCTGTAAGTGAATGGCTCTGAATCCGGGATTATTCCATGGTGCTACTGCGGGTACACACCTCCATCGCAACACAAAAATAAATAAATACTGCCATGTTGTATATTCTTTGTAACTAACATCTTATTGTATATAAAAATCGTTTTGTATCGAAATTAATAAAGAGTTGGCCAACATATT

>chr2:+:140277313-140277329

GAAGGAAGAAGCCAAAG

>chr2:+:140277586-140279041

AAAAAGAGGCTGAACCTGCGAAGGAAGAAGCAGCCAAAGAGGAGGCAAAGGAGGAGGCGAAGGAAGACGCAAAAGAGGAGAAGAAGGACTCTTAATACCCCCCGTCTCTTCTCACATTTAGCGAATAGTGTCTTTCTAGCATCCTAGGTCTTTTCTAACATCCCCGATCCTCTTTGGGATTGTAGGACCGTCCCGCGGATTTTATAAATGTTTGGGGTCTTTCCAAGGATGATCCCAGGGCCTTTTTAGGATTCAAGGATTCTCCTACGATTCTATGAAATCTGGGATTATGGGAAGGGAGGGGAATGTTTTATATATATAAAGTATACGTTTGAACTATGCTCAGTAGTTGTAGTGATACAGTATGAATGGTCTTGCATTGGGTCAATGCAAGTCGAAGCGAAAGGTTCGTTCATGCGTTAGAAAGCGATTCGATATTTGTAAATTAATGACTGAAATCTAGTTCGTGTTTGGTTATCCAGTGTGATTTAATATTTGTCTTGGTAAATTTGTATTGTAAAGGATTCATGATGATGATGATGATGATGACTGCAAGGTTTGCATGGGTAGGTGTTGAAATGCTAGAAAGTTTGTGTTCCTAGATGCGTAATGTGAAGTCAAAAATGCATTGCCATGCATTTTTTTTAGCAGTAAGGATTTTTATAAGCAGACATGTACAGCTTTTTGTGTGACTTCCCGGTATTATATTCCCCTGAAATTTTAACCCAACAGAAATAATATCCGATGTATTTTTCCCACTAGTTTCAATGTAGTTTCAAAACCGCCCGTTTTTCTCAACATATCAAAATCCTATGAATAAAATAGTAATTTTATTTAACTAAGGTGGAATTCCATCTAAAAATACAATTTATTTTGTATAGTCTTCAAAGAATTTCATTTCTCCAATACCCCGCGTTTATTCATCGCCTAATTTGGTGTCTATAATAACATACAGTGTAACTTTTACAAGAAAATTTACCCTAACATAAATTGCAGATTGGTTGGCACCATAATATCACGGTTTTTGCCAAGTTGTTCTTCATCTCCTGCTACAGTATTATGTAGAAACGCTAGTTATGCCGTAGAATATAAATCACTGCCATTTTCTTGACTGAACCTTCTGTTTGTGCATCCTTCCACTTTACATGACGAGCTTTAAGTGTAGCCAGTATTCCAGGAAGCTTTTATAGGTGTGGCATAACTAGGAAACTTGTAGGCAACGATCGTTTTGCTTTGTAATTTGGTAAAGTTGAAAATTTGGCTTGGTTGTGTCACTTACTGTCAAACTATTTTCAATACTGTAACATTAAAAGATCTATTGTCTTTTCTTCATGTGTCTTAGGGAATGCACGCAATATGCTTATTCCACCTTTCAGCAAAACAGTATCCAAAGTCTTCCATAATTTAAGTCGTAAACTATCTAACATTCGGAATAGTGTTACAACCCACCTAAATG

>chr30:+:7230967-7233691

GGATTAATAGGGTGCTCTGGTATTTCTGGAGAATAATGATAGATGAACAGAATAGCTGTTTTTTGTGATTAATTTCACATTCCTCTAGTTAACTGTAGAAAATATCTGAAACAATGGAAGGGTAATGTCATTAATTACATACATTAAAGCTGGAAAGGACTATTGTGTTCAATAATTCTGGAGATGATAAACTTTACAGTTTTCATTCCAGTTATGACATAAATACTAAAGGATATTTGTCTTCTCCGGAAGATGGAAAAAAAGGTTAGTGAGGTATTTATCTTTTTCATTTTCAGCTCAGTAGTTGATCATCATTTTCAAAGTTAGTCAACTGATTTCGGTGAGCAGAACAGTCATTGCATATGAAGTCTATGATAAATAGGTTGTTATATGTACATGTTATTGCAATCTCAGTCTAAGATCTGGAATCAAAGTAGATTATCACTTGTCACCTGTGATATAATTTGAGGAAGCAATATACAGCATAAAAAGTTTTTTATTTCCTGTAGGTTTACCACAGTACAGAGAGAAAGTATGATGTACTGTATTGTGAAAAGTGTCAGTATTTTGGGTTTTGTTAATGTTGGGTTGTCTTCAAGAAATTGCAAATGTTGAGATTCAGTAGTGCTTGTTGAAAAGTACCATATAAGTTTTAGAAAAATGGTTTTCAGTGTTGAAATTTGAGAATAATTTTGTAATTTTATGCTTTGTAAAGTACATATGGTTATATTTCTTTGAATTTTCCACACGTGATCATCACAATTCATTTTTTTGAGTGTTGAGTGTATCATGCTATTATCTATGGATAAATGTTCTTTTCTACATCTGTCTATTTGTGAGAGTTTTATTATTTATGAATAATGAAAAGATATTTTCTTTGATATATAGCAAAATATAGTAATGACTGAGAAAGTTTTGTCATTGTAGTTTAGTATTGTTTTCTGTATGAATGTCACAGGCAATCAGGTATTTTAAATAGACTCAGTATGTCATCTTTTGTGGATATGTTATAAAAGGAAAAAACTTACACAAACTACACTTTTCAGTTGAACTAGTATTTATAATTGTAATGAGCACTAGTGGTTTTGGGGAATTTAGTTAGGAATTTAGAATTGCACTCATTTGTAGTGTATTACTACAAAATTTCAATTTTACATCTGTGGTTGTAAGCCCTTTTGTAGTACATGGATACCTATAAAGGTTAGTCAGCAAGATATAGTTCAGCTAATTTTGTTAGTAACTTTGGTTTCGTAAGTTGTCCTAAGGTATTTTTATTTAAAAAGGGTTCAGCCAGACCACTTAGCCAAGAGCTCCAACTCTTTCAAGTGTTGATTCATTTGGGCAAGAAATTTATCTGTTGCAGGAAGTAAGGCTTTTTCTAACTCGAACTTTTTCTGGTGTAGTGGGTTTGGCATCCTCACAGAAAACTTCAAAGGTTTGGGAATAATAGAAGAGTATTGTGGATTTATTTTTTTTTAAATGTACTGTATACATTCCAGTATATCATTGTCTCAACTGTGGTTTGCTGTAAGTAGTTAAGTTTCCTCTACATTTCTTTATATTGTTTGCTTTCCAGGCACAGAATTGCACAAATTGATATGGAATTAGTGTGGAAAAAATTTGCTTCAGCATATGAAGTAAATAGATTTGTTAACTGCTTCACATGGGATAATTAATGCACTACAATATTTTTTATCATATATTTAAGGTTTGTGATAATTTTTGGTTTTAGTACTGTAGTAACACAAGAAAATCAAATAATTTAACCAGTTTATGGAGGCAAGATCTTTAAAATTTGAGGGCTTGCCTTATTTCTTGCTTTTATAGTTTACAAGTACTGTGCTTCTTTGTAAATCAGTAGAAAATTAGCAAATATGGTGATTTTACAAGGGCCATAAGCACAACACAATATATTGGCATTTTGTACAGTACAGATTGATGTTGTTAACATGTATTTACAGCATTCAAATTTTGTGATAACTAGGAAGTACTTATAGTATGTACTTGCACAGCAATATAGAGTACTATGCAATATTGCAGTTTTGAAAGTCTTCTTTTATAGTACAGTTCATTTTAAAGAGGGCAGAACTTAGCTGTCTGCAAGTGGAGATGTTCATTTTATAGCATTCTGCTTATATAGTCCTATCTCCATTTAGGTTTATCAAGGAGGGCAATCAGTGAGATGTTTGTTTCATAGCATTCTGCTTATATAGTCCTATCTCCATTTCTGTTTATCAAGGAGGGCAATCAGTGATGATCCATGGTTTCAGCTTCTGGGAGATTTGTCAGTATTTCTGATATCTAACTACTACATTGTAACTGTACGTCCAGCATATATTTTGTGAAAACACCAGTTACTACATTCTGCTTATGACTTTTACTGAATGTGGAGCAATGATTGATTATATTTTCATTTGATTTGCTGAACTTCTAAAATATTCATTTGTGAAAAGCTGGAGAAAATGCAGTAATCTGTAAGTACTTTACAATAATCTCAATAAGAAGCCAAGTAATTATATCATTGTAATTTATTATGTATACAGACAGATTAAAAAAATTGGCTTTTTAGCATGAATTTAGAATGAAGCTGAGAAAAGAATCTTGAAAATATTTTATCAAAGAAAAAATATATCCAAGCATGCAATAAGTAACATAACAGTGCAGCAATGAAGCAATTGTATAATGAATGCATATTGTTTTTTATGAATAAATTATTTTTTACTGT

>chr24:+:6407233-6408787

AAGCATTTTCCATTTTGCAAGCAACTTTTTTTGTTTACATAAAGATCAAGATAGAAAATTCATTTATAAGAATGTATTTGTAATTGTGAAAACCATTCACAAGAGCTTACTATAACAGTAAAGACTTTTCATAAGAACATACCTTGAGTGTATGGGAGTAATGAGAAAAACTGCGATGATAAAAATACCATTTATTTGCCATCATCTTTGACATATCTACATGCAGGGAAACAGTAATTTGAAGAGAACGCCAACTGATCTGCTGTTAATGCCTCCTGAAATTTCATACTGCACATGCATGGTCAGGGTTGGTTTCCTGGAATACCTAGGAAAATATACACTAGTAAGAACATTGGTGTTGAGGCAGCAAAAGGTTACCACCACATTGCAAGAGTTTGTTTACATTCAGCTACTGTTACACTTGTACACATTATAAGTAACAATAACAGTCAGTTTATAAACTATACTGTAGAACACTTAATGTAATTTGAATAATACTGTAAGTAATGTTTTAGATAAAAATTAAGCATACCAATGGAAATATATCTTTCCTAGTTTTTTTTTCAATTAAAGAAAAGGTTTTGCAGGGATTTATTGGCTATAGTATCTACATATTAAGTATATCTGAAATGTTTAAAATACATTCTAATGAAATTCACATTTCCTACATGTTAACTTTGGATAATAGCAATTACTTTGCCAAGGTTACTTGTAACCTTTGCATTGGATTTGGCTGTGCAGCAGCATTAACACAGAGGCACTCACTCACACCCCCACTCCACTGCCTTCTGCTGCCTACAACAGGAAATAGACTAACTGATTTTCAGCATAAAGTCCCGACGTATCGTTGCACGTCAGCTAGCCTGAGGGAATGTGCATTGTGCCAGTATTTTCATGTGCAGGCAAAATTAAGTGGATTTGGTAATTCATGCTGCTCTTATATTTATGAGACTTTGTCTACTTGTAGGCCTATCAGAAGGTGAGGATCTAATTTGCTAGTTTGACAGTAACTCACAAACAAATTGATTAGAATGAGATTGTTTTATATATAGGCTATATATGTGAAGTTAAGACAAGGTAATTACAAAAATAGGTAAATTGGAAAAATCCAAAATCAGGCTACTTTTATACATATACTTTCTAACCAAAGTTTTGTATTGAAAAGGTCTTACAGTACCTTAGTATTATAGGATAAGTATTAGTTCCTGCTTGTAATACTTGGAGAACTTGAAGTCAGGAATTGATTGAAAGAGCACTACTGGTCAGACATTATTTAAATCTTATGACATCAGCTTGGTTGTCAGTGTTCCATTATTCTGTAATATCTGTGTATTCTATTGTAAATATGATATAGAAGCAAGAGAAAATAAATTATTGGTAATATATTATAATAGCCTTTAATGTATGGAAAGTAGGTTGTTAAGAGAATTAGTAATGTGTTTTATTTTCATTGCAATAATATTACCGAGGAAATGTGTTGTATAATGGAAAACATGTTAGTGAAAACTTTTTTTCTAAATCAATGTAAACACTTGAAATAAAACTGAACAAAGTT

>chr13:-:43103318-43103501

TTCGTTCGTTCGTGGGGCAGCCACGCAGACTCCAAGCACATCACACCTACTTGATACGACTTCCTCGCATGAAGTTGTTTTAGAGTTTTGGGTTTAATATTTTGCAGAATACCTGGACATGTCTTTGAAAAGTGTTGTGACTGGTGCTAGGCTGTGTGTCGCGCCATTAAGGCAAGCGGCTGCT

>chr13:-:43098952-43099035

GCAAGACTAAGCACCAGTGCCAGGCATGGTGTCAGCTACTCCCCTGTGCCGAAGCCCCTACAGGGGAAAATGAAATTGTTCCAG

>chr13:-:43096803-43097689

ACTGACAACGGTCTCCCAGTGCACGTCAGAGGAGGACCTCTTGACAAAATGCTGTACGCAATAACGGCAGTTGTCTGCACTGTTGGATTAATCGAGTGTTTCCACGTCTACTACGTCCTTTCCTACACTGCCAAGAAGGACTAGTCGGGATACAGTTTCATTCCTTCATTTCAACTGCAGTAAATCTCACCTCAAAGAAATTACTCTCCTTTCCCAAGGCATAACAGATATTCTGCCAAATATTTCCTGGTTTCAAAAATATTCTTAGGATAACTTCTTTCTCGCGCTTTTTTTTTTTTTTTTTTTTGACATGAATGGTTGTAGGCCTGATGAAATTCCATCGTGGTAACAGCGACTGATCTAATGACAACACGAAACGTTTCTTTTTATTAGTTTGACTCGTATTCTGATGATGACAGATTTAAAGAGATGTTATGAACTCTGGTAGGTAACATTCCTCAGTACTTACAGATACTAAGGCCTCCTACATTAGGTATCTTTAACTTATTTCACGTAGAATAAGTAAAAGTGAACTAGAAGTGATGTAAATATTCTCCTTCATTTTTTCTCCGAGGCTCTCAGGTGGTCCATATGTTATGTCATTGCCAGCAAGTCTTCCATAAGCTAATTGCCCATGATTGATTCTACAATGACTTTAAGAGGGTCCAGAGTTTGAAGCACTAATAGCCTTTGAATGTGCAAGGGTTCTTGCCTTGAGTTTATATATTCCTGCGATTACAGAGTTGACTATCATAATTAAGCTTCATTTACTCATAACCATAATTCATCTGGGTAGACTTAAAGAACTGTGGAAGACTTGTTATGCCATGCCTTCTGCCATATCTTGAGCTGTGAACATAAATGGGTCGAATAAAGTTAAAAATGTT

>chr5:+:82888534-82891945

GCTGTACTTTTATTTGATACTCCGTCTCACAGGAAAATACAACAAGAAGGAAACAAGACAAAAAAACATCAACTTTGGTTCTTCAGTGTGCACTGGTATAAGACTGTGCAACTAAGCCCGGGCCAATAATTTACTTTCTAAATTGCTTGCCATTAGAAAAATACGATACACCAATAAAAAAGTAAGACCTAAAGATTATATAAAAAGTAACTTGTGCTATGAGTAAAATTGGTACTAGCGTCTATGAAATTAAGCGCTAATAATTTACTAAGACTTGAGATGAAAAAAAAAAAGTTGTAAAATTAACCTCTGCTAATACAGCAGAGATGATATTTCATAGTTGGCTTTCTTTTACATTTCATCAGAGACCTTTGCTTAGGCACAGTCTACACTAACCACCTTTTTCTTCTGATATCATAACACATACATATTCATTCTTGACAGGAACCATGCAATGAAAAAAAGAGATGAACTTTGCTCATACTCCAGCAACCCCTTAATCAGCATCCTGATTACAAGAGATAAAAAGCGCAAGATACCAAGTTTTAGGCACTGTACAGCCAGATTCATTGGAGGGCAATATGCTAGACCAAATTTCAAAGTTACGATAAAATGGAGTGCAAGTTTATCAAAAGAACTGAACATTAAAAAGTCAATAACAATACTGTTAATACTTATCATACTAAATGATGAAACTGCCCACACTACAAAATTTATTCATCAGCATAACCATCATCATTCCACCTCACATGACAAATGACTGAGGCATCAAGAGGCAACAATAGTGCAGTGTGTATCAGCTTTATTCCTAACAATTATCTTGGTTTGACACAATAATGTGGCTTTAGTGTGATAAAACTTCAAAGCAGCATATGTGTTAAAGAAGATTTTCAGCAGCCCCCAATACAATAACTGGCAGAAATAGCATTTTTTGGGGGAGTACAGCACTGTCATCAAGCGTCATCAGATAACACGTGTGGCAATCACAGAAAGACACCCAGACATTATGCGGGACATCATGATGCCTTACCAAAATAGATATAAATTTTCAGACTTAGGCATTATGGCAGAAATATTAATACAGTATGATTGTTTTCATGGACACCTTTACATTCAGCACACATGTATAATGAAAAAGGTTGAAACTATGCATAACATGTCCAAGTTCAGCTTTGACTTTCGAATACCCTTTCTTTACTTTTGCACAATATAACAGTGGTTGCGACAGTTCTTGTTAATTTGTATGGCACAATCAATTCAGTTTTACTCAAAACTAATTTAGTTTTGTTCCATTAGCCACCCAGTAGCAATGACAGAGGGGATGGGGACGCAATAAATGGCACATAAAGTACACAAGCTTTGATCATTCAAAAAGATACTTTTTAAAATAACATCTCTGAAGTCCACTGAATGCATCAAGAAATTAGTGTAGTAAATGTATGTAAGCATTAACTTGGGCACAAAATTGATGGTCATGCAACATTTTGTGCACACAATAATAAAGGATGTCTGAAATATTGTCATACAAAACCAAAGGCGATAAACTAAGAGTGAGAGGAACCCATACCTTAATAGCACTCGCAGTGAATGTAAAATTATGCATATGGGGACCTAACAAAATAATAAAGGCAGGCTCAACACCATGGGTCTGAGAAATATGTCCATAGCCACAATACTATAAGAAAGTTTAAACATACAATATATCATAAAACCTCGACCTTGTATTATTAATATAATTTAGAAATAACACAGGTACTCCACTGCCAAAAAAGACAAAAACAGAGAGAGAGCACTCCTGATATTGGGTACTAAAATGGCAGTGGAACCTTGAGTCAACATTCTCATAAAAAAATCCTCATGACACTTTGGCACAAAGATGGAGGAGAGAATAGTCGTTGAATACACACTGAATGCAAAGGCAGGGAAAACTTGACCACATTCATAATAATTAAAAAGCGTAGCTGTGTGGCAGTTCCCATATCCATATTTTCTCCCGACCTTTTTAACGTTTACCTACATTATTAATACCAGTTTAAAGATAATTTTATAAAATCCCCTAGCACTTACATATGAAAATCAATCATCCTAAAATTACAATCCTAGTTATGACACTATAATAAAAAATACATTATTTCCTTTACATCAAAAGTAATTCTAACTACATAATGGTAAACCAAAATATTTCATTAATCAACCAAGCAAAAAAAAATCATTCTCAAGTACTTTACAATAAAATAAATGATTTCATAACAGGGGAATTTTTACTCATATGTACAATATTATGCCTGTCAGGAGTTTACAATGGAATATGGGAAAATCAAACACCTGCCAGCATGAACATGCAATTGACAAAATGTAAAAAAAGGAAAAAAATGGAACTGGAATACACTTAATTGGTATGTAGAATGACTCAGAACATCCGACAGAGGAAAGTCAACTCTCCGCTAATTAAGTACAACTAGTAAGTACAACTGGGATCGTTGAAAAATTACAAACCATACATGTTGCTACACCTCGTTCTTACTTATTCTGACAGGTGAGCACCAGACAAGGAATCTTTGTGCAGGTAACACGCAGTACATAAAGGAGGACCAGCAGGAAAGAACAGTCTCCTTCCTGTGCTGTACATTTGTTCACTCCACACAGACCCACAACACTCTCACAGCCCCAGCTTCAGTATAAAAAGTTACATCCCTTGAACTCTGTCCCGTGACTACCTACATAATAATAATAAAAAAGGATGTAGCCCTTCAGTTTAAGTTTAGCAGGTTCACTTACCAATTTCTCTTAAAAGACAGGGCTGAAAGTTAGAATATCCTGATGGTGGGTCAACAACAAAATCCACACCATAAGCACCTAGTCCTTATGTAGAAACCCACCAGGGGCTGATCAATGATAAGGACATGAAAAGAAGGAGATGGGACATTCCACTGCAATGTGAAGCTGGGAGTGTGGGAACTATTAATCTGACCTATAAACAGAGAATCTTATGTGGGTTAGGAAAGGAACGGGGATGGAGAGGGATCATGCTAAGTGGGAGTCTTAAAGAATAGAGAGTAGAGAAGATAACCGGAATGCTTGGGATGGATGAAAGTTGGAGGTATTATTGGTCAAGGTCGGCTATGGAAAACAATAAGAATTAGGTAAAATAATTCAATGTTATGAAAAGTTATAAAATGATGGGAGTTTATCATTGGCCAACACTGGTTATTATTTAATTAGGATTAAGGTTTTTTTTTAGTGTAAGAGGTTAATTGAGGTGAGGTTAGGTTGTGGGAGGTCACTGGGATGACTGAGGATCACACTGCAGCAGACGAACAATTGAGGGATCACTCTTTGCCCCTTCAATAAACTCCTCTAAGCTAAGTTTCCCATCTTTATTTTTATCCATTTGTCGGAATATCTTATCTGT

>chr3:+:99166676-99168643

GGATACGCTAACAATATTAACTACGGTGCTGGCATGCCCTCGTACCAAGGCTACAGCATGTAAGCAATTATCACCGCCATTCCCACGCCAGTGTAAAGTTTTCAGGTTTGTTTTCATAAGACAAGTGCGTAGAGTTATTAGTAGGGCAAATTGCTACATCTAAAAACTGTTCATACCAGTCATTTTTTTTTCTATAAAAGGTTGGTTTCAGAATAAGTGTTAACTTGTTTCCTCAAAGCTGCAGGCTTAACTAATAATGAGCCTCATAGACTAATGTAAGTTACACAAATACTGTAAAAAGGAAGTTGACTTATCCGCATGGATATGTGTCCAAATTCTACGTAGAGGAATCTGTAAGTTTACAAGTGTAAATGGCCAAGTTGTTTGGTAAAGAGCAAAGAAATAAGCTTATAGTGTGTAATAGTGAAAGGGCGAGTGAGTGAGATTTATGAGGCATTCAGGTTCAGAGACCTGGCTGCTACTCTTCTGCAGATTGTCCTGTTTATTCAGTAATGTCTATTAACAGAATTTTTTTTTTCTTTGAGAAATTTGTGGAGGTGTGGGGAAGACATGCTGCCTCACATGGTTAATTTTTTTTTATGTTAAGGAACTTGTCTGAATGTGGAGGTATAAGCCCATGTAAATGCTTTTTCATTGTATATAGTTTTTCCTGTGGCGATTCCTGCTGAATGTGATGTAGCATTTTTTGCCTATTCCTCTTCTGATTTATAATTGGTTTCTGGTGTAGAGACATTGATTCTGTAAGAATTACGAAGCTGAATTACAGTATGTATATATAGATTCAAATATAAGACATAATAAGTTATAAAAAGTTTTTGCAATGTGTAAACTTTGATTTTTTTTTCTCTTAGACTAGATGATGAGATACTGGATTGCTTTATCAAACCATTCCTAAACCTATGTGATGGGCAACTTCCCAGAATCTCATCTTCAAGTCTGAACAAATGGAGATTGAAAGTGGAAAAAGCTTTGTCCATTGTGTGCCTTCAGACAGGAAAAGAGAAGGCGGCGGCTTTACAGTCTACATCATGATTGTTAATCAGTGAGGTGATACTTTCCAAAGGCCACTAGAATGTGTTCAAATTATCTAAATGGTTGTTCATTAGAGAATAGAGTGTCACCAAATTGATAGGGGTGGAGCAGCTTTATATTTATAGACAAACCTTTGGATGAGTTAACCTTGTTGACAAAGTAAATAACATTGTTGCTGGTTATCAAGAACTCATAATGCCATGATGTTGCTTGGAGTGCATGGCAATTTTTAGTTTTGATAATTTTCACCTTTCCCCCCATTCAAGATGTGAATTATGGATTATTGATAGCTGGCACTATGATAGAGGCATTGGGAGGAGGAGGAAATTTTTGTTGAACGGAACTTAGACAGTATGCTAGTTTCATTGAAAAATTATTTTTGACAATTTCCATTGTTTTGTGGTCAAATTCAAGGATTGGCCTATTTTGTCAGTTCCTCATATCTGTTGTATGAAATTTTTTCCGTACAGCACTTTTTCCATGGTATGACTCCATGGCTATGTATGACTGTTTAGAACTCTGGTACTGTACTATGCATTCTTCTCTATCACCTCCACTCATTTTTTTTTTTCATTTTTGTCTGAAAGTACCATGGAAATGTTGGATTATTTTTCTTTAATATTTAACAATAGGATCATTTGCCCTCTGGGCAGTATTTAAAAACTGCTGATATAGATATTCATTGTATTTTATTAATTGTTATTGAGGATAGTAATTTTATTTTTAAGTTAGGATTTGAGAGTTTTAATGTTTCTTTGTAAGAAATAGCATGTACATTTCCTTGGTGATGGAGCCAGCAAGTGCCATCTTCATCAGGCTTCAGGAGACAACTGTTGACTCCCCAGTGGCTGATGCCTCTACTTAGAGCAGATGTACTGTGGCTACATGTCAAATTTAAATAAACACCTCCCACAT

>chr27:+:23236233-23237133

GTGGAGAACCCCAGCCCCAGTCCAGCAGGTTCGAACACTACGAAAACATGTTCAACTATTACGTCTACCCCCCACGCCAGTAGAATTCGGGATCGATTCGCTCCTCTGTTGCTGTCGAACCTCCTGAAGAGCGGAAAACAACAACAACGCTATGCTGCTTTTTCTTCCGTCGTTTTTACTCAGAGTGCAATGATGCCGATTCTTCAACCGTCGAGGAAACCTAGACTCATATGCCTAAGGATTTGATCTAGACTCATATCCCTAAGAATTTGACCTAGACTCATATCCTTAAGGATTTGACCTAGACTCAAATCCCTAAGGATCTGACCTAGACTCATATCCCTAAGGATGTGATCTTGATTCATACCCCGAAGGATTTGACCTAGACTCAAGCCAAAGGAATTGAAAGGCTCTATGAGACTATACATTCGACTTGGGCCATGTTTGACGTTGGTGCAACTTCTCCATCCTTTGCCACGTTCCTGTTTTGAGTATTTTCGCGACATGACCTCACCCTCACCTCATCCAGAGCTCCTGGCTGTTCAAGGCTGCTGCGTCCCAGCCAGGGGCCCTCGGGAGTCTATTGTACAACGTGAACCAGAGCCAACTGGAGATTCTCAGTATACTCACTCAGAAGGAGAATTGTGTATACATGTCAACCTCATCAGTATGAATTGTAAGATCGCGTAAGTCAGAAGTATTCGATGTGTTTAGATTCCAAACAGCAACTACCAAAAATAAAACTGTTCATAAAGTCATTTAACTAGACATAAGGTCAACCACTTTAGTTTATCACAAGCTGTCGATAGTTTTGCATCCAAGAAGGAAATAAACATTGACAACCACGACGTCCAAATTAGCATTTTTAGGTTTGTTTCATTGGGCACAATGCTTCTGGAGC

>chr2:+:156438240-156439777

ACACACAAAACTAGGCATGCATGTGATTAATGGTGTAAGAAAGGACTGTTTTACATTATGAAAGTTAAGAATTTATGTAATTTCAAATGTTTGAGTTATATAATTATCATATGACCAGTTGGAACCATGAAAGATGCTAACAATGATTGTTAAGGTAGTGAATGGTTATTATCAAATCCCTTCCTTTTTGGCTAGGCCTATATGTTCTCAAGTTGATTACAGTATAGCCCACAGTAATTTAATATACAGGACCGTACGGTAAAATAGTGCAATATGCAAATTTCAATGTATGTAATGTGAGGTTATATGTACTGTTAAAGAGGCAGTCTTTCTGAGATGGTAATCTATTATAAAGGTATTGTTTTGCAGCACTTTCATAGAAAATTCAAGTAGGAATAAATGGTATTAGGGATGAAGTATGAAAATTTCTTAGCAGAACACCCTATTGCAATAATGGTACCTGTGGGCACAGTTTTCCCCAATAATATAATCCACCTAACCCTCCCCCCTTTCCCATGTTGTAGCTAAATGCACAGTGCATAAATTGTACTGGTCTCATAGTTAAGATGCTATGATTTTTTTTTAAGAGTTGTGCACTTTTGTGTGTTGATTACTTTGTACAGTATGACTTTCCCTGTATCTGAATTCAGAGCACTTAACGTAATGACTAAAAGGAGCATGGTCTATGTTGTCACTAAAGTACCCATTTTCAGTAAATTTGTATGGTTATTTATGTGCAGTATTGAGAAAGGAAGTGATAATGCTAGTGAAGCCACCTCCTAGGTGCTGAATTTTTATGGTTTGGTAATGTATAGGAATAATGCAAGAAACTTTTGCCAGATTATTTAATTTATTTGTTATAAGGGTTAGGTAGCATATATTGGTTTTTATAGGCTGTATATAATTAATGATAAATAATTTTTTTTTAAGTACTGCCATTATTATTTTGTATTGTATGGTACCGTACAAGCTTAGTAAATAAATAGAACGGACTTCTTAGGCCTCTCTAAATATACTCACATTTCCCTGTTAGTTTGCAAACATAATTTCCAAGTTGTATTGTTAATGTGGTTTCCTGCTGTTTAGTATATTATGTATTTGGTGATAGTTTAATTTGCAAGTAAGTTTATGTACTTAATGATTTACTTACAGCTAATATACACGGGAAAAGTATTGGATCCTCTTAATCAAATATGTATTCCATCTTTCAGCCTTTTGAGAGATTAATATGTGATGATAGGTTTTGCATATTTCTTTTTTACATTTCACATGTGTAGAAATATTATGTTATTTATAATATCTGCTATTATATGTGACAACTAGTAATTAAGAGTGCATCCTGTTATTTATGGCATGCAAATGTATGTATGTACTATTAGGACACATATGTACCTCAGTTTAGAAAAGCTTATCTTACAAATTGCTCAGTAATGGTAACGTTGTTTATCGGAGCTCAGAAGAAGATTTGGAGAACAGCGTAAAGAAATATTGCTGAGCTTGAAGGTTGTTGCTTTATGCTGACTTACTGCTTTATACCC

>chr1:+:144479952-144481717

GGGTTGCGCAAATAAAATTGGTGGTATTTGTGATCTTCCCAATATTGATAATTATTTTTTTTAGATGAGAGTATTTTAATGCTTGTCCTGTAATGATGTTCTGGTAATACATGATAAGCGATAGGTATAATTTGGTATCCAGAGACTAATGGTTTGTTCTTCCCTAATCCTTTTAAAGAGCTGAGTAGGGATTTGTTAGAGTGCTTCACCAGGAAATTTTATCCTCTCTTTATTTGAAAATGTACCAAGCAAGTTAATTGCATAATTAGTATGGTGGTATTTCAATATTTTGTGTATAATCTTATAAACAGATTTGACATGAACAGTTGTATAGGGCTTTTTTTTTATCAGCTTGAACTTTAATTTGAGATTTATAGATATTTTTCCTTTTATTTTTTTCTGTCATTGAACTCCTTATTCAAAGGGGTAATATTTTGAAATTTTTTTTAATGGATTTATTACATTGAATTTGTTTTGTCTTGAATTGCTTCTATTTTAGTCATGTTCCAGACAAAGCATTTTGAATTTGACTTTTTTGGTTTATACAATTTATGTAGGGATTGGTCGCAATTGAATATTTTTCTTATGGGAGCTAAAATAACAATACCTGAGGGTATTGCAAGGTCATTGTGAAAGTTGTGAGCGCCATATCCTGTGCCCTGGTCTTCACATATCTTGCTCACTTGTTGTACCATATCGCCAGCCAAAAATATCATTTAGAATAACAGCACTTGCGGGGTTTTACCTTGTACTTGAAAGCATTGTGGAGGTTAGCTTTTGTTTCCCTTTTGAGGTATTTTTTCCTCAGCGCAGTACACAACCTTTTTTTTATGCTTTATACAATCGACTAGCAATTTTTTTTTATCTGATGTGTGTGTATCATCTGATACAACCGAGATAGTGTATTTTGGATTTATTTTTGTATAATTTTAATGTGACAGGCTCTACTTTGCTGTTGGCTTAAAATGCTTTGAAAGCTACTGCACTTATCATTTTTTGTTTAAAACCAAAAGTTTTATTTGTATCATGTTTGGGGAAACACCCTGAACACTGAAAAGCACTGTGGAATTTGCCTACAAAATGTTGTCCACTTATGGGAAGATAATTAAGATTGCTATTATTTCTTGAAGTGAGGTCTTCAGATTTCAAGTACAAGAAATATCCCAGGATCAGATTTATATAATTCTGATTTACTTTTGGGGAGTAACGTAGACATTTGTTAATTTTTTTTTTTTAGTTAGTGTTTTACTGGGTAATCCAAATATGTAAAATTATTTTTACTTCTAAGGATGTACCTTTTTGCTTATTTATTTGTGGAGAAGGCATTATTTGTATATCTATGTTGTACTTCTTTAGTTGTTGGTGTCTTTTAGATGAGTGTGCTTTAGGGGATTTGCATGACTTTTTTTTCTTTTTTTTTATATTATAACCTGTTGTTCCACAGTTGAACTTTTTTTTATATAACCTGTTGTTCCACAGTTTTATTTTGAAGTAAATATGGTTAGATGATAATTTTGCTTCCAGATATGATAATGATTCTACAAAATGTTGTTTGAATATGGCATTGTTTCATATTCATATTTAGTTTTATCCCATGCTAGCATATCATACTAGTTTATTGCATACATATCTACCATATCATATAATTTTGTATTTAGGAAAATTCAGGCTACCCCACAAAATAAAGGATTTAACTTCAATTACAATATATGTGGGCACAGGATATGATGGTTTTGGACAGAATGAATAAATTTGATTTTTTTCTC

>chr8:+:31026316-31026771

AGTGCTGAATTTGGACGTTGCAGCTGTAGCCTAGACTGAACCAGACTGCGCAAGACATGAGAAAATACTGTCAACATTGAGAATAGTTAGTTACACACTATTTTGATGCAGGAGGATGACGTCTGCTAAATATTTTTTTATGCGTGGATCAAGCGCATTCAAGCAGCGCAATTTGTCTTGAATGAAAAGTAGTGAAGTGACTACAATAGTTTTGTGTTAGAAGTGGCATTTGCCCCCTCCCCCCTCCCCCCCACAGAAACTTTTCGTGAGACAAGAATTTAAGTTTTATCGTATAAGATACAACAACGGTTTTAGGTCGGGCTTTTTAGACAAAAATTGCAGGTTGAAAACGTTACTCCATTCGGTCGCTTAAGGCATCAAGGAGGAAAGATGGAAGTGTACAGACGAAGCCATTTCTTCTTCACACCACCAGCAATTCTCAAAGTTGTCGAAATG

>chr8:+:31032020-31032208

GTAGTTTTGGTGACCACCATGATATTGTTTTTGGTGGACAACAAGTGTTCAAGCGATCTGAATTTTATCATCGTTTTTATTCTACCCTGCGTTGCTTGTACTCTTGCAACTCTTGTCTCTTACATATCAGCTGTTATGGTGCTTATTAGCAGGCGAAATCCACTTACAACTCCAGCTTGGGTCAAAGGG

>chr8:+:31033525-31033629

GATGTCTGCTTCAATATATCTGCACTTGTACTAATGCTTGTGGGTTCTATAATCACCCTCACGAGAGAGGAGTGTGTCAACAGTTCTATTACAGTTGCTGCTCTT

>chr8:+:31053331-31054756

GCCCTGGGTTTCATAAGCGTGATTCTTTTTGCTATAAGTGGAGCAGTGACGTACTTGGTTTTAGTTCGCCACCAACAAGAAGTGAAGGAGACTCAAAGAGCTCAGCTAAATGCTAGAAGATTAACTATGAGTGAATTAGCATAGATGCAGAAGAACTAAGTATATGTAAATAAAAATCTTGATAGGATTGCATTGACTGGGGAAGTATGGCATTTATAATAAAGATTCAATATAAATTAATTATGCAAGAACACTGGAGTAAAAAAATTAGTGTTCATTTCCCAAGATCCTCATAAAAATGGGAACTTAAGTAGTTTTCATCCCAAAAAGGCAAACTGGCACACATAATTTTGTGAAGTAGCCAGCTTTTTTTTTTTTTTTTTCAAACTGTTTCAGATCATTCTGCAACAACAAGGAAAATCTTTTATCTTTTCATAATAAACAGGTAATATTTTGATATCTTAAAAGCTTAGCCTAAGGTACTATTGTTAATCATAATGCCTGCAAGTAATAGTTATTACTAAACTATTCTCTCGAGTTGAAACCTTCAAAATGAGTAGTTTATAGTTAATTCAGTTTTGCTCAGTAAATAGTTTCATAGTTGCAGTGTGATTTGCTTGAAGTTCATTTTTCAGAATGCTTAAAATGTTTTTATAGATTTTGAAATGCAAATAGTTTATAGCTTAACCATAAGTCGTTGTCCATGGAAGCTTGTTTATAATGTCAAACTACCTTTGCATATTTTCTTATTTATTTGATTAGCACCTTTCTTATTCATTTTCAAACTACATATCTTCATGTATTTTCTTATTATTTTCCTTACCAGTTTTTCTTATTCATTTTCAAACTACATATCTTCATGTATTTTCTTATTATTTTATTACCACCTTTTTCTTATTCATTTTCAAACTACCTTTATGTGTCTAATACGTATACCTTAAAATATTTATCGTAGTACATACTTAAATTTTACATTATTTATATCAGAAAATAGCATAAATTTTCTCCCCCTTTTTAAAATGATTGATTCGGTGTTCTCATAACTTTGTTTTTCATAAATGTAACTGTTTACTCCTAAATAGTATATCACTTGTCAGACAGTTTCTGAAATTAAACTTTTTTTTATTTTTTTTTTTTCCTAATGAGGTCAGCACAAGAGAGCTTCTTGTTTGAAATTAAGAAAAAGAATAATTTCTACTCTTATAGCTGAGTCTAATAATAGTTTAGTATTGCACTTTCCTGTAAGTCTGATTATTTTATTTATATTTTTTAATATGACTGATCAATTTTTACTGCTCTTTCACTCTAAACAGTAATTTATGTTGTTTTAATTTTAGTAGTTGTCAAAATGTACTCAAAAAGTATGTATCTTTGAACTGTATACTGTATTGAGCAATAAAATAAAATAAAAATATTTTCACTGGTG

>chr11:+:9438647-9441028

CTGGTCAGAGTTTGCTCGTTGTGTGCATCTGCTATACTTTGCATAGAACTAGTGAAGTGTGTGGTAGAGCACACGCTGGCTGACTTGGCCTCCATGTTGCAGATCAGTGAAATGGTGTAAAGAAAGTCAAAATAGTACCTGACATTATTCTAGTACAATTATTGTAGGTCATATGTTATTTCTGAAAATTACGTTTGAGTATCAATGGACACAGCACCATGGTAGCAAAGATGGAGTAAAAGTCAACCAGTGTGTGGCCTCCTGATTATGAATGTACTGTAGTCTTGGTCCAGGTGTAGGTGTCGGTAGTGGAGTAAGTTGACAGGGTGGATGGTTGGGTCACTTCTTTTAATCTCCCATTTCCATCTTGGAACTTGAGAATTAGATTTATAGTTACTGACCATTTTATAGAAATGATCATGCTGGAAATCATACAAATACATGCATTCTGTTGCAGCTTACTTGTGTAGTGTGGAAATTTTCAAAAATTTGTTCCTCCAACCCTTGCAAGTTACTCCATACTGCAACTTATAATAATGCTTTATCCAGTAAGCTGTTACATACATAATTCTTTTAGCTAATGGGCATTCATTAATTAAATGCAAAGTATAGATGTTGGATGATCGTCGGATTATGTGATGGGCTGCTCACAGGAGTCAAAATGGAAACATTCTAGAAGTATTTAAGATTTTCATAATTCAAACAGATTTTCCTTGGAAGATTTTAGTTTCATGTAGGGTCCACTGAAGTTATATCTACTGTATAAATTTTAAGTTAGGTATTCAGATTTATGTACACTAATTTCCAGTAATGTAAAGTTTATTACGTTGATGGAAATAATAGTTGTATTTATCAATTTATATTTAGGCATGCTGTTTGACAAACTGTAATCTGCTTTTCGAAGAAACAATAACTAATTAAAAGGGATGAATTTGTACTGAGTTCATCATAAATAAAGTAACAGTGGAGTAGAAATAAATGCATTTTCAAACAAGCATAGCTAATAACCTGTGAGCAGCAAGGCCATACCCCAAAGAATATTAAAGAAACTTCAGAAGTAAATTTCTGTTGTGGGTGTGGTCTGGAATGTAAAGCCCATTGTCTGCGTGAGACAGCACACAGAGGGATGAATTGAAACACCTTGGTGGTATCTACAATTAGCATGCTTGAGGAACCTTGAATTGGACCCTTAACGTGTGTATTGGTGTTTCGTTCAGTAGGAATGCCCGCAGAGTGTGTGTGGTACTGTTGTCATCTGTCTGTGTGCTGGCTTTATTTTTTTAATCACCTTGTTCAGTCTCCATCAAATGTTGCACAAAACACTGGGTTTTGCAAATGGGTATGTCAAGCCTGTTCAGAAGTGTTCATCATTATTATTATTATTATATTGCATGAGTGTTAGTGCATGTTGTAAGTCTAATAATAATAATTTGTTATTTTACTACTTTTAATCCTTTTATATTTTGGGTTTATCTTTTATCAATTTCTGTTATAGATATATAAATTAATTCTACAATTACTTTATATAAACTTTGATGGAGACTGACTGAGAGTGATTTCTCATTAGGTGTTTCACTCCAAACAAGTGTAAGTCATTACAAACTTCTCAATGATGGTGTCAGTTTTGTTTGGGTGTCATCTAAGAGCTTCATAGATTTACTAGCCAGTCCCATGCATCTTTTGGGGAGAGTTGTGCTCAAATAGTTCTCATTGCCCAGAACTCCACAGTAATATTTTTAACAGAGGGGACAGTATTGGAATAATAGCGAGGTGTGTTTTGTGGGGTCAGCTTTAGTAAACCTTTGAAGCACTGAAGTATATATATATATTTTTTTTGTGAATTTCTAGTACAGTACAATTCTTATTTTGCGCAGATTATCTTTTCACTATGGCATCCAGAAGTAGAGTGCAAGATGGCTTGCATTCCCTTCGACTTTTTATTTTGAGAGGATTTAAAATTCTGTCCTCTAAATAACTGTGTCAGCTATTGACATGCAAGTGTATTAATTGGTCAAAGTCTAAATAAATATTTTCTTCGATATCCTCTGTAGGTAATATAGAACCAAATATACACATCAATTGTCAGAAACATGCAACAAATGACAGTAATCCACTAGCATGTCCATAGTTTATACATTGTTTATAATATTTCATTTAAGCTTAAGATATCAAATCTCCAGTTACATGTTTAGAGAAAACTGTATAAGTAACATCTTTTAAAAAGTATGAAATGCTATAATTGGCGGGGCACTCAGATACTATGAAAAAGGATGAACAACGTTTCAGGATTTATGTTGTTAGTTGAATATATCTTTGTGTCTTTCCAATATCCTCTATTTATTATGTTATATGAGTCTGCACCATGATAAATTTGCCAAACTC

>chr28:+:26565617-26568513

GGATTTCGATGACTTGGATATGGCAAATGAATGCGATACCCTGGGAGATTTCCTCAAATTGACAGATGAACAGAAAAAGGACATAAAAATTGGTATCACCAGTCTTGTACCCGACGATGAGAAAGCCAAGCACCAAGACAGCCAACCTTTGACTAACGGTGTTTCAACTAGTCGAGAGGTAGGCGTGCATTAAAGCACCTCGCGCTGGCTTGGAGTAAGGGACTCATTGCTGCATGTTCTGCTGCGCCAGTTGGGGTTACATAGGAGAATACAGTTGGCTAATGTATTTGTATTATGTATAGATTTACTACTACTTTCATCAGCTGATTGGAACTAACAAAAAACTCATGCAATGTGAATATGCTTAAAATGTTCTCTTTAGCGTAGTCTTTAGATACGATATTACACATCAGCTTTAAAAAATACGTTTGTGACTTTGTCAGTTGTTCTATAATGCTCATTCTTATTTAATGCTTTTAAGTTTAAAGAGATGCAATACGTAAATTGTGAAAGATGCTGTACTCCTTTTAATCTTTGATGTAGTACAGGATTTTGGAGTTTTCTTCTTTTTTGTGTTTTTTCTTAAATATAATTCCTGACCTCTGCATCTGACTGACAAAGTGGCAATTTTACAGCGTGCAATTAACATTCCAGCTTGATGAGTCGAGAACTGAAGTATGGAATTGTATTCTTTATTTGTGTAGAACATTTTAAGTCTGAAGCTCAAGGTACAGGTATTGTAAAAGGACTTAAAAAATATTCGTACTTGATGATATGACCTTCATTTTTTGGCAAGTAGGAAAGTTTTAGAAATAGAGAGCTCATTTTGTCATGAATACCATTACTAACTGTGGAAGTTGATTTCTTCTTGATCACAATCAACTTTGACTACTGGAGTAGAACTTAAACAGCGAAGAACTATTACCCTGTCTGATCAGGGTCAAAGTACTTCACAAATGAAGTTTTTTAGACGTAGAGTTTTTTTATCTATTGAATTCGTAAGTGCATTTCTTTTCAGATTGTACATCAAACCCATGTGATAAATAACTTAGGAAATTAAACTCGTTAGCAATGAAAAATAAGCGTGTGGAACGTTTCTATAGAAGCTAATCACTGAAATAAAACAAGCAGATGCTGCGTAGTTCAATTGCAGAGCATTGTTTACTCATGATGGCTCATTTCAGGTCCCAGTTTCAATCATTTATGTAATTTATGTATCCCAAAAGTAGGTTATAGAGGTTATGTGCATACATTGTAAGACATATTGAGATGTAATCATTTGGGTCTATGAAACATTACTGTAGTTTTGAGGGAATTAAATTTTATTTGTAGTTACTATTTAGTGCAGTACACACAAATGCCAAAACCTGTACTTCTTTACTGATTAACTTTATAGAAATAATTTTAGCTTTAATTTTTGTACTGTACAGTAGTTCATAATAGTCTTGTAGTGCTGTGTAAACAAAACAAAGTCACAAGCCCATGGTGATACTACTGTATTTTGTGCAGTAGGAGCTAGACTGAAAATCTTTATTGCCTTATAGATAGACTGTCAATCAGCAGTAGACAATCCTAGTTTTTCTAATCCTTATATAAAAATAGTCAATTGTACTACCAGTACTTATGTAATGCTAAATATAGTTCATTGATTTGGGCTAAGTACATCATGCTTCCTACTAATAGTTCTACATTTGCAGGTAGTAAAATTAACTATTGGCAAATGTGTCTTTACATTCACAAAGATTTTGTAACGATGTAGTTTCTTCTCCTCCTCATTATCTTTTGTTTTGCCTTTTCCATATTTTTCAAAGCTTATCAAAGCACCATTCAGGTACTGACTTTTTGCCAAGATCCTCTTTGTTCAATTGATCTCGTGAAAACCAGTCCTCTTCATCCATGTAAAAGACAGATTTTTGGTGGCCTACTCCTGATCACTGAATTTTATGGCCCTTTTATCTGTGCATCAATAAGATATTAATAGCAATGACTTAGTGTCTTACTTTCATCACATTTTAAGATATTCTCCCATACTCTTCTTGTTTGAACTCCATTAATTTTGTATTCATTACAGTCTGAGTCACATTTTAAGCTTATGAATCTTTGTAGTTACTAGAAAATTTAGAAAATAACTTATAAATTCAAAACAGGGTTAAGAATGCTCAACAAAGTCAAACCTAAGTGCTGTCTCAAGCACATTACTACTATCATCAGTTCCTCATACTATTATTTATTGTTTCTTTAGTGAAAAAGATTTCAAACTGCTGTGTAAATTGTAAAATCATTTTGCATTAGTGACATCCATACTCGTTTACTTCATATTTATGCCCGGGCCACAATATATTCAAGAGTTTGACATGGATCTTTGGTGATTTTGCTCTGTTGTCATCAAAGCTTTGAGAAATTAATGTTTATTACTTACATACAGTCATCTTTAGAACAGATTAAGTACCGTATGTAACACTGTGTTTTTCTAAGTATTTTTTTCTAGGATCTGAGTTATTATCTGTTATTCTGTTTTGTATTGTTTCCTGTTTAGGAATATTATGTCTATAATTTCATGAAATTTCTGTAAATTTGTTTGGCGTTTTTTTCAGTCTTCTAAGGTACTGCACTAATCATCAACTAGTCAGCAACAGTTTCTTGTGCAGTACAAAATCTGTCTGCATAATTAGTTTATTATATGGAAAATATTTAATTTGTAAGGGACTAAAATAAATAACATTTCCTAATTAATATACTGTACTATTATCAAAACAATCATAGTATTTTTCCAGTTCTTTAGGTACTCCACTCAAGAAACTGTTATTGACTAGTTTTTCAGTACTGCTATTGTACTGTATTACTATACTCATTCTACATGAAGATAATTGAAGATTAAAAATAAAATTAGAATCACG

>chr8:+:105951829-105951890

AGTGTTCCAGAAAGTCTCGTCAGCATCAGACATCCCAACGAGTGACGACTTTCCAGCAAGAT

>chr8:+:105954798-105954862

GTCCACTCTCCCCATGAAGACAGTGTTTGCTATTGCTCTGGTGGCCTTTTCCTTGTTCCAGATAG

>chr8:+:105955076-105955161

GATATGCCATTAAATGCTACATTGGTGTGGATGACCTAATGACATTGATTGCACTACGTCTTGTGTAAAATGGGAGACTAATTCAA

>chr8:+:105956436-105956964

AATATGGAGACATTCTGCAGTATAGCTGTTATACGAAAAAGGAGACGGACGGTTGCACGACTAGGGTAACACCAATCCCAGGTATTGACAAGGACGCCACCAGAACACAGTGTTTCTGTAACACTGACCTCTGCAACAGTTCCGGCGTGACAATGTTGTCCCTACCTGTGCTTGTGGGCTCCTTCCTCCTGAGGATGGTGATCTAAAGTCTTCCAAAGAAGAAGAAGAGGAAGAAGAGAACTTTCCTGAAGGGAAATAAAAAAAAGATGCTGTAATTTGGGTACTTAAAAGTTTCTATGAATGCTGTTCTTTCATTCGGAATTAATTGTAATTCTTGTTTGATCATGTAAACTGTTGCCATACTCAGTTATTGATTTATTCACCTAAGTTTTAGAGGAATGATATTTCCATATCTCGAAATCAAGGTAAAATATTCTTTTTTATCATGAAAACTCCTTGTTTGTTGACTTCTATTGTAGTAGACCAAGGTTGAATGAATGATTCAGAATAATAAAGAGAAAGTGAAACG

>chr42:-:35346245-35348056

TACAAGGACAAGTGCAGCACAATCGTTGCCTTGGGTTCCCCTGACGAAGTCTTCTCCGAAGTCCAGAAGGCTCTCAGCGCCCTGCAGTAGACATCTTCGTCATCCTTCAAGATCCAACTTCAACGGGAAACAATTTCCTTGCAGCCAAAACGGATAGAAACTTTTTCTCACGATCTGAAATTAGATCAAAGCTGTCCACCACATCTTCCTAGTACTACTTTCGTCGCTATGATTAAGCAAAGCTTCCGGTGATACATACATAGACTCAAAGCAGAATCAAGCTTAGCGTTCAAAGCTTCTCTTCCAGCGAAGGGGGGCGGGGACGTTTGAGGCAGCTAGCTAGAAAGAAGTAGCTCGAAATCACCTTTCACTATGGCCTTAAATTTTCAGCAACACAATCAATAGAGTGGCTATTTTCTTACTTTGTAAGTGGAATCATTTTGCAGAAGCTGAGGTTTTGGTTTCCAATAGAGCGGATAATATGTTTTTTAACCCATATTGGGTGTTCAGCCAGATGACGACATTCTCCTGTCTCCTCCTGCATGAGGTAGCTTTTACTATTCCGGAGAAGAGTGACAGATTACTTACTTCTATTTTATTTCCCATGCTGGTTTGATGTCAGTCTTAGAGCGCGTTGACATTCTGAAACTTTTGTTTCTCTTTCTAGTGACTATAAAATATGTTTTCCATCAATGATTACGACAAACGGACAGCTGAAACAACTCTAGTCAATCAGAATCAGTCGCTCTGTTCTGTTGTTTTGTTGCCTTTTTTTGTCAAGAGGTGAACCAGTCTTTCGTTGTTTTACGGTCACGTGACTTCACCCAACGGAGGCCTTGCCATTCTCGGTCTTTCATATGAGGAACCGGTAGTCTGAATTCAGGCTTCTTAGAGAAGTGTCCTTTCAGTCGCCTCCTAACTTACTTCCCAAGTAGTGCATCTCGTGAAAGGATGATAACTGAGACGATTTTTCAGGTTGAGATTATAGATTCACGAAGACCCTCCTCCCCACACTTCGATCTTTGTGTTTTCATCCGAGTTTTCTTCACAGCACTTGAGTAATTCCAGTTACCCTCTCCATTCAGTAGAGTAGTGAATATAGTGCCCTTATAAATGGGAATCACATTCATATACCACTTTTAATGTAGCTGCCAAAGTGGCTTTGTAGCCTCTCCAGTGAGACTTACTCTTCCAATCGCGAACTATGCGTTGTCGAACCGAGTCTCCTTCGGTTTACCTTTGGTCTCTGCCACTTAGTACTAAAGAGTAGCCGAGAGCCGTTTGACTCTCTCTCATTCGCATGACATCCCTCACTCTTGTAGGCTGTTGATTAATGGCAAAAAAGGCATTTTTAGGTCTTCCTGTGGATGTTAATCTAATCCTAAGAGAGTCACCTGTTGCACGAATCACCATTTTGATTGTTATCTTAGCCCAGAACGTTTAGAGCAATGCATATATATAGAAGTGGATATTGCATTTACTCGTTATAAATGAGATTTTTGATTGTTGTCTTTTGAGAGCCTGATAATTATGGTAATGTTTAAACTTAAGGCAAAAATACTTTAAAAGCTTCTGTTCTCGCACTGTGAGCGAAATCCAATCTTCCAGAAATGCTCAGAAGTCATTGGGAAAACCCGACACCACGTAACTAAGACTATCTTGAGTCCATCTCAGAGTGATCGTAGTCTCTTTCATGACTGTTCAAGAAAGCATTGACTAGTCCCTGTTGTGAATGTTGTTATCTATACGGTTCATTTGTTGATTTATACAAGACATGAATGTATTTATAAATTTTTTTCATAAATGTCACGT

>chr14:+:20899813-20900050

GGGGGGCGTCTCGCCGTGGATCATACTGACATCATGTCTCTCGCCATATATTATAAAAATAACATAACATTTATATATATACGTATATATACGACTCGATCGTTTACTGCCGATATACTAGTTGAGATCAAAATAATAATAATGATAAGAATAAGTAGAGTACTTTTGTGGAAGGATTACTCTCGAGTACTTTCAGTGTAAAGTGAAAGAGAACTTTGATAAAAATAAGACCATCTTT

>chr17:-:81283443-81283591

AGTCATCGTCGTTGAAAGAAACGTAGTTGTCATCAGGTGTATTTGAGGCTGTGCTTCGGTTCCCGTCGTCTTGTCGGTCATTTTTTGATATTGTTACGCGATGTCATCCAACGAGGACGTAGAGATCAAAGGCGGGCATCCTCCTGCGT

>chr17:-:81253556-81256964

TCCTCTCAAGGCACCAGTTATGATTCAGACGCAGGTTATGGGGAAACCAGAACGAGACTTTCCTGTAGCAGCAGTAAAACACACACATGAAAAGCCTGTTCCTACACACGAGACACGTACTGCTCATATTTCGAAGCCTTCTCAAATCCAGCAACCAAGGAAATAGATTATGGCAGCATAGTACTTCACTCAAGAGCCTGTGGAATTCTGTATTATTTTAAGAAAAATTTTTTGTGATGTTTGAATGTGTCTTGGATCAGCTGTATTTTTAAGGAATACTTGGTAAGTAATAGAAGCAATGATGATTTGATGAAGTACAGTAATGAACATTACTAATTAAGTAAGGAATGAGGCAGTTGCTCTGATAATTTCACAGAAGTTATTTTTCATTGCTCACGAGAACAGAAATAAGGGACGTACTAATTTTGTGGTAATTGGTTAGTGCCATGGTACTTCAACCTAACATTTAGGAATTTTGCTGTTTTGTTAAGTATTTGATAACTATCAAGGTTAGCTATCAGTTATGCATTGTTTATTTTGAGAAATACAGACCACAGAGCTTTTTGTGACTGCATGGGTCTTGTTTTCTGTATATTATGGTAAAATGGGTTTTCAAGGATATCTTCGTGGGGTTTGTATATAACTCGCTCAAATCATCAGGGGATTACATGTAAAGTTAGATTGGTTCCATAGGGCTTAAAATTAAAATGTTGCCATTTGTTCATGCTTTGTATTTGGAATATAGTTGAAATCTTTTATGTCTGACATTTACATTGAAGTTATGCAAAGTTATATTTATATAGTATGTTTAGAAATATGCTGTGGGCAAGGAAACTCATGTAGTTTGACAATTTAACATGGTTTGTATTAAGGCTTGCAATTGTTTTGCTTGATTAGTAATAGAGATATGGAATTTTCAAGGTGGAAAAAGTTTTCTTTAGGTGTAGGATGTTTTGAGAATATTTAAAAAGATTTTAATGAAAATAAAACCAAATAAAAATTAGCAGAAGTTAAGGCTACATAAATAAGTACAGAAGTCAACTTACCTCCTTCAGCTTTTATACTCTTTAAATCATCATAAAAGTCTGATGCTTACCATGAAGTTCTTCTTTAGGTAATGTTTAAAGTGTTAACATTAGTGCATTTTTCATTACCTACCCACTCTGTTCTCAGTAGAATTTATCTGCATAACACTAGCGAACATAGATATTTTTGTTACCCATTAAAGTGGCCTTTAGATGATACTTGTTAAAATATAAGAATTAACAAATATATGATGTTGAGTTTTGACAGTTCATATAGTTATAGGAAGTCACAACTGTCGAGTCTTAAGGCAGAAGATTATTCGCTTGCTATGGACAAACTGAAAGTGGAAGACTTGATTTGGTCTATATTTCTTTTACATTTTAACAAGCCTTAAAAACTCAAGTAAGAAATGTATAATTTGCTTAATAGAAGTATCACGTCACTGAAAAGTGAGATAAATTTAAAGTGCTTTTAAATGCTCTGCTTTAAATTTTTTTCTTTTGTTAAGTTGACATTTCATGTTTACTGGCGGATATTACTGGTTAATGGCCTGAGATCCAAGTTTAAGATGTAAAAAATTTAATGAAGCTTTGCAATTATTCAGTTACGAAATGGACATCAGAGTACAAATCTCTTTCAACTACATATAACAAAAGTTATGAAAACTTTAGTAATAGCAATATGTATGTCTTTCAAAAAACAAAAGTATTTGGTGCAGTTTTTTATTTGGTGCATGTATCAGATTGAAAAGTTGTCAGGGCCCTCAGTGTCAGTGGATACTTATGAGATAGTGTTTCAGGTATCAATAATTTATTTCCCTTTGCATATGTGATGAATTAGAAGTAGAACTTGTACTTTTATACTGGAAAGAAATTCTTTGTGATTAAAAATCAGGTTTCAGTAACAAATAGAATATAGGTGTTTTGGCCATGAATAAGTGCTCGTCTCTGAAAATATCGTTATGTAAGGTGAGTGAATGCTGTGAAAACTAGAGGTGATGCATAGACTGAAAGTACATGCAGTTTATAATTATGTCGTCTTTCACAGGGCACAAAAAAGGGATAGTTCATTGTCGGTGAGATAGCTAATTATATTTTATCTGAAGAGCTAAGTAACTTGTAGGTAGTTCAGAAAATATGCAAAGCAAATTTTCTCCGCTCTTATTTTAAACTTATTTTTTCAGTTAAGTTCAAGTTCCTCTACGTTATACAGAAAAATGTATATTCATCTAGTAGCTCAAACTTGGGGATTACACAGTATGAAAGATGATAGTGAGTTCTTTAAGACTTGTGCCCTGGTGAATGTTTGGGGGTAAAGCTTAAATATTTCCACCTTTATACTGTACCTATAAGCTTTATAGTTGTTAAGAATAGGTAAATATAATGAAAATTATGAAAAGTTGCTTCCCTTTTTGGTTAGCAAGAACAAGAAATGCCATTAACATTTGTGCTTCACAGATTTCAGGTACTGGTACATTGAAGCCTTACCAAAATTTAATTTGTACAATGTGAAGTTTTAGTATGTTCTTTTAAGTTCATACTGCTTTCCATGTAAAACAAGCAGTTTTGCTTGACAGTAGTTAATTTACTTTCCACAAGAGGACCACGTTGGTAAATTTCTTCATAGCTTTAGCATTCCAGTTTATTATTTGGGATCATAAATGCATTTCTACATACAGCTTTCTTTGGGCTTTGTTAGATTTCCATAAATGTAAAAAGGTTGTGGGTGTCAATTTAAATAAATGGATAAATAAAAAAAATCAGTATATTTTTATACATTAGTTGAAACCTACAATACTTATGGATACTTCAGAGAAGAAATTGTTCCTTGCAGTCTTGAGTATGTATCAGTATTTTTTTTTAGTTTGGACCAAACAAAATTACCCCTTTTTTATTTTCTTATGTTCCTGAATGGCAGTAGTACTTATAAGGTGAAGCATATTTGCAATTGTGGACTAAGCAATTCCCTTCAGTAGTTTCACTAATGACTTTTGTATGTGCAATTTGCCTAGTCTGGCTTATATTATTTGCTAGTCATTAATAATTTTGTTTGGTTCATTGCACCTGAGGATGTCCATATTGGTTTTAGAAGTAGACAGGTTTTGAGAACAATTTCCAGTTGTAGATTATTGTAGGGAACATAATTTGTTTAAATTCTTAAGTTGGTTTCAAAAGAATTGCCAATGAACTTGTAATGGAACTAATATGATGGTTGTGGTAATTATTTCATCATTTCCTTTTGGTAGAGGTTGAGTTAGTGTATTTTCCTAAAAGTATTGTAGTAACTTAAAAAATTGGGTATAGATATTGGTACTAGAGCTGTGTGATGAGTTTCTATTTGGTTAAGAATTTTTATTCACTGGAAATCGCTTTATTAAAATGCT

>chr17:-:81253556-81255705

GAATTAACAAATATATGATGTTGAGTTTTGACAGTTCATATAGTTATAGGAAGTCACAACTGTCGAGTCTTAAGGCAGAAGATTATTCGCTTGCTATGGACAAACTGAAAGTGGAAGACTTGATTTGGTCTATATTTCTTTTACATTTTAACAAGCCTTAAAAACTCAAGTAAGAAATGTATAATTTGCTTAATAGAAGTATCACGTCACTGAAAAGTGAGATAAATTTAAAGTGCTTTTAAATGCTCTGCTTTAAATTTTTTTCTTTTGTTAAGTTGACATTTCATGTTTACTGGCGGATATTACTGGTTAATGGCCTGAGATCCAAGTTTAAGATGTAAAAAATTTAATGAAGCTTTGCAATTATTCAGTTACGAAATGGACATCAGAGTACAAATCTCTTTCAACTACATATAACAAAAGTTATGAAAACTTTAGTAATAGCAATATGTATGTCTTTCAAAAAACAAAAGTATTTGGTGCAGTTTTTTATTTGGTGCATGTATCAGATTGAAAAGTTGTCAGGGCCCTCAGTGTCAGTGGATACTTATGAGATAGTGTTTCAGGTATCAATAATTTATTTCCCTTTGCATATGTGATGAATTAGAAGTAGAACTTGTACTTTTATACTGGAAAGAAATTCTTTGTGATTAAAAATCAGGTTTCAGTAACAAATAGAATATAGGTGTTTTGGCCATGAATAAGTGCTCGTCTCTGAAAATATCGTTATGTAAGGTGAGTGAATGCTGTGAAAACTAGAGGTGATGCATAGACTGAAAGTACATGCAGTTTATAATTATGTCGTCTTTCACAGGGCACAAAAAAGGGATAGTTCATTGTCGGTGAGATAGCTAATTATATTTTATCTGAAGAGCTAAGTAACTTGTAGGTAGTTCAGAAAATATGCAAAGCAAATTTTCTCCGCTCTTATTTTAAACTTATTTTTTCAGTTAAGTTCAAGTTCCTCTACGTTATACAGAAAAATGTATATTCATCTAGTAGCTCAAACTTGGGGATTACACAGTATGAAAGATGATAGTGAGTTCTTTAAGACTTGTGCCCTGGTGAATGTTTGGGGGTAAAGCTTAAATATTTCCACCTTTATACTGTACCTATAAGCTTTATAGTTGTTAAGAATAGGTAAATATAATGAAAATTATGAAAAGTTGCTTCCCTTTTTGGTTAGCAAGAACAAGAAATGCCATTAACATTTGTGCTTCACAGATTTCAGGTACTGGTACATTGAAGCCTTACCAAAATTTAATTTGTACAATGTGAAGTTTTAGTATGTTCTTTTAAGTTCATACTGCTTTCCATGTAAAACAAGCAGTTTTGCTTGACAGTAGTTAATTTACTTTCCACAAGAGGACCACGTTGGTAAATTTCTTCATAGCTTTAGCATTCCAGTTTATTATTTGGGATCATAAATGCATTTCTACATACAGCTTTCTTTGGGCTTTGTTAGATTTCCATAAATGTAAAAAGGTTGTGGGTGTCAATTTAAATAAATGGATAAATAAAAAAAATCAGTATATTTTTATACATTAGTTGAAACCTACAATACTTATGGATACTTCAGAGAAGAAATTGTTCCTTGCAGTCTTGAGTATGTATCAGTATTTTTTTTTAGTTTGGACCAAACAAAATTACCCCTTTTTTATTTTCTTATGTTCCTGAATGGCAGTAGTACTTATAAGGTGAAGCATATTTGCAATTGTGGACTAAGCAATTCCCTTCAGTAGTTTCACTAATGACTTTTGTATGTGCAATTTGCCTAGTCTGGCTTATATTATTTGCTAGTCATTAATAATTTTGTTTGGTTCATTGCACCTGAGGATGTCCATATTGGTTTTAGAAGTAGACAGGTTTTGAGAACAATTTCCAGTTGTAGATTATTGTAGGGAACATAATTTGTTTAAATTCTTAAGTTGGTTTCAAAAGAATTGCCAATGAACTTGTAATGGAACTAATATGATGGTTGTGGTAATTATTTCATCATTTCCTTTTGGTAGAGGTTGAGTTAGTGTATTTTCCTAAAAGTATTGTAGTAACTTAAAAAATTGGGTATAGATATTGGTACTAGAGCTGTGTGATGAGTTTCTATTTGGTTAAGAATTTTTATTCACTGGAAATCGCTTTATTAAAATGCT

>chr7:+:93735025-93737970

GCGTGGAAATTGATAATTGCTTGGAAGAACAGTATGGCCACCAAGAAAGGACAGTAATTAATATTACGTTTTTATATTCTTCAAGAGCATTGTCAAAAAAGTGGTTAAGAACTGGTTAACAAAGTGTTAAAATTTTTGTACCGTACTTATTTTGAATTTAAATTTTGAATGTTAGTACTATAAGGGAAATTTCACACTTTAGGATTTTAAATTTAATTAATATATATATATATACACACACACAGGCAGTACTTGGTTATCGGTGGACTTGGGTAATGACGATCCGGTTTTATGGTGCTTGTCTAACACCATAAAGTCACCGATTTATGGCAACATAACAGGCCAAGTTTTGGTTATCGGTGCCACGATGAATGGTTGTTTTGCTTATCGACACCCCGCTGATAACCGATGACTGCCTGTTTATATATATATATATATATATATATACATCTTAAATTTCTAGTGCACATTTTATAAAATCTAGCTGTTTTATTTTTCATCAGCCTTGGTGGATGTGCAAACTGCTTTTAAAATCCTCATCCTGCCAGTGTTACTTCCCATGGAAAACTTCTGCAAAGTTTGCTAATTTGAACAAGTTAATGACTTGCCTTCTAGTTCATTGAAGAACCAGTGGAATTCTAGTTCTTATTGTAGAGAATGAGTAGTTCTTTTAATATTTAACAATATTTTTACAGATGGTTAACTTATGACTGTTATTGATGTACTCTATGAATCTTGTTGTACAACATATCATCCAACAAGATACAAATTGCTGTGTGGTACAGTGATAAGGATTTTTGTAAGGAGTCAATTTTTCTGTGGTGGTAATGTATTTTTATAAAATATTATTGTATTTGTTTTTATAGAGAGGATTAATGTTATGTGCATAAATATTGGTGCAAAATATTTTAATATGTTTTATGGCCAACAATGTGCCTTTATATGATTGGGTTAAGATTCTGGTATAATTAATGTTAAGTACTGTTAGGGTAAATGACACCTCATTATGTGTATCTTTCATCAAAGGAATTCATTAAAACATTTTATATGGATTACAAAACAGTAACCAACAAATCGAAACAGTGGAATGAAAATGGACCAACAACACTACAAAAATATTTGTATCTGGAAGGGGTAGTGGTTTCATCTTTGTTTATTGTTTTATTATTCTTTATTTGGGTGGTTGCACCCCACCAAATAGAAAATCTAAAATTATGTTAATTTTACTTCCCAAATTATTTTGGGTTTATAAGAATTAAGTTTTGTTATGTTGCATCCCTACCCAGGGTAGCAATTTGTTCCTATTAACATACATTTGTAAGTTATTTTCCAGGACACTGAATGTTACAAACAAATCTAGGTGATTTGTTCAAATTTCAGTTCATTTGTTGGACTCTTGATAGAGCAATTTTTCTTTGCGGAATTTGGAAGTTAAGTTACTTGTTGTGGTAGTTAGACATCAGAACACTCAAGTTAGTAATTTAATTTTTGTATAGTATTTGTACTGATATTCATTAATGATTCCTGTTATTAAGCAACTTGAAAAATCATCTTGTGCCATCTCTTCCTTCTGTCTAAGATAAATCAACTTGGTTTTGAATTACTTGAATGACAAATGCTAACAATATTCTAATTCATTGTTAAATTTACTCTTCTTGCCATGTGTATGTTCAAGTCCTTGCTTTTATTTTAGCACCTTTTCCCTCAAATTATTATAATCATATTTTTAAATTGACTTGTCTTGGTCGGATACCTATAAATTATGAAATAAATCACTATACATTTGGCAGTATTTCAGATTAGGAAATCATGAGGCATTGGGATTGAACTGGAAGTGTGACATATATATTAATATTTTTGGAATAATATATTGTATATTGGGAAGTGAAACACTTCCATTGTACAATGTATCATATGTATATATTAAAATCATATTTTCAATTCAAATTACCATGGCTTGAAGGTAATTTGATCTATTATGTGGTGAACTGAATATGGGAAAACATTTAATGACGTATTAAATATCAGTCAATGCATTTTTGACAGACATTTTTTAATGTCCTGTATTAGTGCTCATTACTAGTTCATAGATTTTTATGAAGTTTTAAATCACTCTCATAGGTATTTTTTCTTTTTATCTTGGACCTGCACATTTTATACCTGTTAATCCTTTCTTTTCCAAGCAGTTGTAACATTTAATAAGAAAAAAAAGTCCTTTACAAACTTAATCTTGGATAAACTTTGTTCTGAACACACATTACATTTGTTTCCCTTTCATTGGGGTGATGTGTTTACCTGTGGTTCATTGCTTAAGTAAACATTTCTGCATGGCAGTTAAACTGGAAACATTTGTATTGCAATTTTCTAAGACTAGAAACAAGAAAATTACAAATTTACTCAAGTTTGAGAATGCCATCAGCAATGCATTTGATAGTTAATGCTTGTTCTTTTCTGTGAGGAATGCAAAAGTACTCTTGAATGCTTTATTGTCAGGTTTCTATTTAATATCAAAACCAGATTTTTGGTGAGTTTCTTTGGTATGTTTACAGTGAGTAGAATTGAATGGGGAGATGGTCAGATCTCAAATGAGTCCATATTGTGTTCAATATTTTTAAGTTCCTTTGGTGCAGATGGCAGTGTTGAAACCGAGTAAAATAAAGAATTCAATATTTATATAGATATGTTTTACAACATTTAAAGGATCTTTGCACAGGTAAAATTTATGAAACTGGTGTATGAAAGCAATTAGTTGTAATTGCCACTTAATGCTCTCTAGTTTGAGAACAAAATAAGTAAAATTTGAAAGTGAACCTTTGTGAATGTTATTGCCTCTGCATTTGCCTCAAATACTTGGAGTGTGGCCAAGCTACAGTTTTCCAGATTTTCTTTCCTATTTTTATTTCATGTATGTTAATGGCAGACTTGTTGAAATAAAATTTGTAATAT

>chr1:+:107449150-107450362

AGACCTGATCAAGAGTCTGCCTAAGAACACAGTAGCCGTGGAGAACCTCCGAGCCCGCCACGCCAAGTTGTTTGAGAAGATCAGGGAGGAGCACGCCTCCATCGCCGCCGAGAGGAAGGCGCAGGAGGCGTTAGACACTGCAGAGGAGGGAACTTACCAGTAACCGGACAGTAAATGATAGCCTCCACGCTTACAGAGCGCATACCATGAGGCAGCTTCCTTTTTTCTGTAGTCTTTACTCTCGTCCTCATGTAAGAGTTAATATAGAGGGTTTTTGGGTTGGGGAATTACGTCTACTTGTTTTAGATAAGCATAAGATCATAGTACGTGCTTACGGCTACTTGTTACAAAAAAGTTATATATATATGTCTGCTTACTTCGGCCTTTTACTTTGATCGTTTTTATCGAATAATCAAACAAGATTGGCAAGTTACTTGGAAAGAAAGCTAAGTTACTTGGAAAGAAAGCTAAGTTACTTGGAAAGAAAGCTATTGAGTAAAAAAAAAATAAATAAAACTCTCCGGAATAATGCCGACTTACATTCGACGCCGAGTCATTCTAGGCCGAATCCACTTCCATTCTGTTATATATCTAATATGTCTCATCTCTCATGACCAATCAATAAGCCCGCACACGTTAGGTCTGAAAAGAGACTCGAATAGTATCTAAAACAAGGGATGACTCAGAGTACACGTTAGTTGGTGAACGCAGGTATGGGAAGGTCGACTAATAGTTACTTAAAATTGGAGTTATATATCACTTTTTACACTTCTGTTTGGGTGAGAGCTGATCAAATGCTGTCTCAATTAACGAACACCATATAATGTGCCATATTTGTAACTGGTAATAAGCTTCTTCCTGCAAATGTATTGGTTGATTATTACGTCCTAGGAGACTATATAGAGGTAATTTTTCTTGTCCTCTGATGCCTTATTCGGATGCAATTCCATGACATATTGCAATTAAAGCACCTCTAATTTAACTTGAATCAGAAATACCTTTAACAACATTTTCCTTGTGTAACTTTTAAAACTATTTAACCATAACATTATTTGTGGCTTTTTGTGACGTTAAATTTTTTCGTTTTTATTTATTTATTTCATCAAAATTTAATCTGATAATGTCTTATCGAATGTCCTGTTGCAGCTGTTACGTCGGTTTCCTGTAACTTCATAATTACGGAATTGTTCAAAACACTTCAATAAAATATAGG

>chr35:+:30453191-30454260

AATGTATAGTTTTACGTCTTTTTATTATGAATATATAAAGAAGAACTGTCAGGCTACCCATATAAAAGTTAAGAATGTTAGGTCACTTCTCAGGTTAAATTGTTTGATGATAATGACCTAGTGTTTAATGGTAATAACTCAGGGCAATAACAACTTTATGAAAATAATTTCTCTCAAAGAAGGACAGAATAGTCACATTTTTTTACTTGTTGCTGAAGTAATAAGTTTTATGGACTCGGATTATTTAATGGACAAGTTGTCTTGTAAGGTGTTACAGTTGTTATCAGATATATGTATAATACTACATGTTTTATTACTTCATGTGTAATTATATAGCTCTGGAGATTTAGGATAATGAAAGTGGAGGGAAATGAATGGAATATCTGTGATATGTAAGAGTGTATTCTAACAAGTGCTGATGAAATAAAAGGTCTGTGTACAAAAATTGTCAGTTTTGTCAGTATTAAGGTGATTATTGTATGATGATTGTATTGCGGTTGCTTATCAAAGTGATCATCATTATATAGAGAGTTCTTTTTTAGGATCTTCAAGCAGTTTCACTTCGTTCCTTAAAAACTTTTAAAAATGGATCAGAGTGATTTATAAAGAGGAAAACCAAAGCATGTAATCAGGGAAACTTAATTTTTTTTGTTTATAAAAAGGCTTTTTTATAATTATGCATGTTATATTGACAAGAAAGGATTTTTTTTTTTAACTACGGATGTTACTGATATCTAAGGTTTGAATTATTCAGGCATAGGCTACTAGGTTTTGAACTGCAATTAGTTTTACTTGAACTCTAACAATAGTGGTTTGAAAAATTCATTTTGTGATTATGATCAAGATTTGCTGAACAGGCTTTTATATACCTATAGAGTAAAAAATGTGTTTATTTAAATAGTAAATAGTACTAGCTTTAAACTGACTTGGTTATTGTGTGCAGTGTGGTATTAGAGGAGGTGCTTGTCTTTCTTGAGACAAATTGAATATTTCACAACTAAAAATATTTCACAACTAAATTTTTAACCGGATATAATCCTTTGTGTTAGAAAACAAATGAATAAATGAAC

>chr3:-:31352167-31352264

GAGTTCGGTATGTGAACCGTACGTTTTGCCATGTGATTTCCTTTTCCTGGCCTTAACCAAATTTGTTTACGATATTATTTGTGTTCGTTAATATACTG

>chr3:-:31311487-31311666

GGATATGAAAAAATTTCATCATGCCAATTAATACAGCAGAAGTACTGTCTTTGGTGACCCAGATTTGTGAGGAGGAAAAACTGAGAGTTTCTATCAAGGAAAGTGTCAAAGGTGGATTTATTGCTGGAGGAGCTACAATACTAGGAGGATTACTTGGAGGTCCAATAGGCCTTGCAGTGG

>chr3:-:31305773-31305894

GTGGAACTGTGGGAGGTTGTACTGCTGCATACATGTCTCAGGGGAAGTTTAAGAGTGTTGTTAGTATTATCAACAATGATTTAACATTTGCACAGAGGGAAAGGCTAGCTAACTCTGTAAGG

>chr3:-:31267299-31269617

ATGTTCCTGCAAAATAGATCTGTGGATGATCTAGCTGCAGCCTCAAATCTTCTTCTTAGCCCTTCTGCTAAAATGGTGTTTCTTCAGATTGTTATACAATTTGTTATTAGTGCCATAGGAATAGTATTTCAGTGGCAGAGAAGTAGAAGAACCAGGGAACAAGAGCAAGGTGAAGTACCACTTTCTCCAAGCTCTTCTAGAATATAAGCCTTCAAATAGTTATCGCTAATGTTTACAAGAAGCTACTGGCATTACAAACTGAAATTTTTGTGTTGTGATTATAAATATAATCGGTTTTCATAATTCAACATAGAAAAGTAGATGGTGTTTAGAAGGTAAAATCCCAAGGGTTTCTTTGAAAGAGGATATGTCAGTTCTCCATAAATTAAGCAAAATTGTCAAGGCACTACAATTTTTTAATTAAGGGCTGACATAACAAGAATTCCGCTAATTTTTTTTATTTTGATGTGCAGCACAATTATGGCCTATAATGATGGACTGTTTTTTTGCAATTATAAGTGTCTGCATAATGTATTTATTTTATCAGATGGATAATTTGGGCATAAGCTGAAGTAGGTCTCTGCTTGCACTAGTCTTATAATAAGTTCTCTGTAGTCTCTGCTTGTACTTGTCTTCTTATAAGCTGTCTCCAAGCATTGGATTTCATAAGCCATCCATTCATCCAACATGGTGAATAAAATTAAGATACTAACCTGCATGTGGATAAAAACAGTTGAGTGTCTTGTTTAGATACAAATTGCACTTCATATACAAGATAAACCAGTTCTTTGCCACACAGATTTCCACAGGAAGCACAATTAGTTTATTATTGTAGCACAAAAGATACACAAGATTAAATAGTTCTGTACTACAGAGTTTTGCACAGGTTGCACAACTAGTTTATTGTTGTAGCACAATAAAGAAGGTTGCACTGATAATTAGCCTTCCCAAAAGATATTTATGGTACATAAAGGACATAGTAGTACTAAGTTCATATGTAGTTCTAATCTCCCTACTAGCTTGTTACATTCAAGCACTGGTTAACAACATTCGGATTAATTTGGACCAGTACTTGTGTAACTTACTTTGCTAAAGCCAACACAAAATTAGACAAATCATCTGTTTTCATGATTTAACACGGAAAGTAGGTGGTGGTTACTAGGTGAAATCCTAAAGGTTTCTTCAAAAGAGCCATAGGAAGAATAGCATTATCAGTGTAATTTATCTTTGCAATTTTTAGTCTTGTCACTAGTATTAACAACATTTTAAAATCAGGTAGAATAAGTTGGGTATAGGTGTTCATGTTCATCGTTGACTTTTTTATGAAGCTTAATGAGGCAAATTATATCAAAAGTTGCACCCAAATTGTTTTGAACTAGTCTACTTCTGTTCATCAGCTTGTATGATGCACTATGGCTGTTAGGTAGCTGGCGGCAGTTAGCATCCTTTTCTTTCAGTTAAGACTTGTGCAGTAATTGAATACCTACGCCCTTATTTATGGTAGTGAGCTATCAGAAGTTCTGTGCTTAGTAGCCTTATAGGTGTACTGGGTGGTGACCATGTACTACAGCTTTTACAGTTATGGGTCTGAGTTCTATCGACTCATTTCTGTCTTTCCATTTATATCTGTATGTTGATCTCTTATCTACCTAGATGTTTGCCTTTAGTTTTACCTGGCAGCAGAGTTTACGCCATTTAAGGACAAATCCCTTGTGTAAACTGTGAGTGTAATGATTTGACTTTGGAATTTCATTAAATCAACCTCTAGTAATAATATATCTGTTTAGGTTCAGCCAGCCTTGTTCTGGCACAGGTTCTAGCTCTTCAGCAGCCTGAAAGATAGTAATAATAGCCTGCATATGTTTGTAAATTATTGTTTTCATTTGCTATTAGTTATTCAGTGTTGTATTCCTCCTAACACCAAAATTTTTGCTTTTTTTTTTATTCTGTTGAAGGGTGCTGGGCAAATAAAATAGCATAATTGAGCTTTTTATTTATCAACATTTTGGATCATTATGTCACTTTAAACTTGTCTTTGAGAGAACAGTTTCATGCCTTAAAAAAGTAGGTGTTTTATTGAGAAGATTTGAGTACTTTTTAGTCTGCTTATGAAAACTATATTTGGTCATCTCTTGATTATGAGGTGTGCTATATCTTTTGTCTCCCATCTTGATATCAGGGAATTTGGCATTTTGCTTGATCAGTCACCCCTCTTTTTCTTCTCCTGGTTCTCTGCTGTGATGCCATGTAGCTTTTGTGTTACTCATTTGTAGGCACTATTTTGGCCACTTTTATTGAGGTCACAGATTGAGACTCCTC

>chr3:-:31264445-31265161

TTAGTTGGCTTCTTAACACCTTAACATCTCAGGCTATTGCTTCTGACATTTTCAAGGTGAGTGCTCTGCTTGTTCCTTAGCCAAATTTTAGCATTTCCCAGAGTGTAATACCTCCCTGCCATCAATAAGCAGGTTTATAGTTCTTTTATTTTTAAAGCCTCTTTATTTTCCTTTTTTTCTTTTTCTTGTGTTCCTCAAACCTAGCCAGGATAAGGACTTTTGATTGCTTTTTCCTATGGTTTTGCTGTTTATAACAAATTAAACATTTGTGCACCCCCATGTAAAACATGATTTTCGCTAGAATTATGAAAAAGTATATCTTAGTTTAACAGACCACTGAGCTGAGTAACAGCTCTCCTAGGGCTGGCCTGAAGGATTAGATATTTTTATGTGGCTAGAAACTAATTGGTTACTTACCAACGGGACCTACAGCTTATTGTGGGATCTGAACCACATTGAGAAATGAATTTCTATCACTAGAAATAAACTAAAATTATGAAACAAAGGCTGATCATGTTCACTGAATTTTGATATTTTTCTTTGGATAGCATCTGACTCTGTATCTGGATAGGATGATAATGAGTTTTGATTTTGGAAGTTTGTAATAGTATGTAGTTCATAATCCTGACGCTTAGATATTGTCCATAAAGTTTTTATTATTTGTTGTATATTTCCTTGTAAAATATGTTTTACTCAAAATATACACCTTTTGAAAGG

>chr38:+:17746407-17746499

GTGGCCGCTTCTTTGGACGTGGTCGTGGCCGCGGTGGAAGAGGTGGCTATGATCAAGCAGGATATATGAACTATGGAGAGGAAGTCATTGACG

>chr38:+:17756424-17756641

GTGATGGAATGTCAATGAGGGGAAGAGGAAGAGGCCGTGGCCGCGGAAGGGGCCGTGGAGGCAGAGGTCCTCGTGGATATTTCCGCAGATATTATGCTGGTTCACGTCCCCAACCTGAAGGCATGATGGGAGGACCTTCAGAAGGTTCACGCCGCAGGTTCCGCCGTGGTGGACGTGGCCGTGGACGTGGCAGAATAGGTAGTGGACCAGAAACAAAA

>chr38:+:17758994-17760069

GGTGATGGACAAGGCCAGGAGGGACAGTCTGCTGGCTCTGGTCAGGCCCAGCAGGCAGTGGAGAACACAACTGCTGAAAGTTCACATAAGCTATCTCATCCTCCCCTGCAATCAAGCACCAAAGCTAACGAGGCCCACTATTTAGGTTATCCAGATAAAGTAACCTGAACAGTGTTCTTAATTTAAGGGTGGATAGGGCTGCTGCTGCTGCTGTCATACCCCCTCCCAGCCCATCGGAAGCCCAGCAGGAGCTCTGTCCACCACCCGGAACTTCCAGTGACAAACAAACCACCGACAAATGAAAGGGACAGTAATGCAAGAGCATAAGCTTACAAAAACCAGCTGTTGCTCCCGGTTACCAGAAAGTCGGCTCTGACCACCTTCAGAAGGAAGCTAATGTGTCCTCTCTCCGGGTCCGGTAACAAAGAGGTCACGCAATTTGCAAAGACGAACCCACGGTCAAGGGTTGGAGTCTAAATGGCCCTTTCTTTGTCGCGCAACACCTGAACACACCATGAAACAAATGTACATTTAGGGTGGGAAAGCACAGTTCATACATAACAGATTAAATAAAAAGATTAAACAAATCACAATGGGTGGTAAAATAATAAAACCATAAAAAAAAAAAAAAAATACTGACAGCATTTATATCTGAACTTTAGGATCTAAGTCCGTGGTGCTGAGGTGTGTTTGTTGCTGGTCTGTTTCCACCAAAAAAAGTGTAAAATCACAACTAAACCTAGTGCACATGATCATTACCCTGTTAGGCCCACATATCCAGCTAGAAGTTGCTGCATTCGTTCTAACCTAGACGTTGCAGTTGGTTACATTGCCATTCACAAGTTGAGAATGTAATGATGTGGGTGCCTCTGGTTAACTTTTTAGGTTATGAATGGAAATTTAGTAAATTTTTATTTGAAAGGGACATGCTGAATGTAGCATGGTTATAGGCAATACTAAAGTGAATTGATTATTTGATTGGCCAGTTTTTTGATTACCTATTTTGACATTTTTCTACATAAAATTTTTGTAGGAAATAATTGTGCCACAGACATGTGCATTGCAATAAAATCCAT

>chr28:+:10922020-10925349

TATAGAATGACAGTGTGTTATATCACTAATGTTTTCTCTTCTTGTTAAACTTTTGTTATATATATTTTCTTGATGCAAGAGAAGTAGCACTGGATTATTTTTTTTTTATAAAGTGATGTTTCACTTTTAATGACTTGACTTGATTTGTAAATACAGAAGTTATGAAATAGTATTTTCCTAGGTATATTACTAAAGGAAATTTGGTAGTCTGTCCTTTTTATTAAATCAGACAGTTGTAAATTGTAGGTTGAAATTTTGTAATGAAAATGTGTTAGCACAAGTTTCTAAGCAGTTTCTTTTATTATACAGTATACAGTATTTTGATTTTCAATGCATGTGTTTTGTAACTAAGGATTCGGGGCTAAAAATATTGTTTAATGTTGATCATTTAGTACACATATACGTAGTTTTGTTTGAAGGGAAATTGGACACTGTGTAAATTAAGTACAAGTATCTTGTCCAGTAACAAAACATAATGCCTTTTTTCACCAAAAAATATTAATGGACAATGCTGGGCAAAAAAAATGAGGAAAGTTATGGTCTGAAACAGTCAGATTGCTTGCTTCTGTTTGTGTGCCGTTTGATTATATTCATGATACTGTATCTTTGAACAAGTTGAGGTATATGGGCTGCGTGGGAGTTGTGATAGCATATAAGGCGTAATGTTTCCTTCATATAAGATTTTTCTTGTATTGATGCTAAGAGATTTTCCTTTATTGCACTTTTAGTGTTTCTGAAGTATTTTCCTTATATGCCAAAATGGTTTGTCTGCCTAGTGTAATTTTCCTCACGAACAAATATATTGAATGAAGTGCTAAAATAATGCCTCCTCTCATCTGATATTCAGGTCATTTATTGTTTACCTACCTAACTCTTGTTTCATTGCATAGGAAAAATCAAAAGTCCTCTTATATAATATATGCAGGTGGTGATGTGTATCTAACTAGTGCCATTATTCTAGCTATTAGGAAACCAATGCTTCTCAGAATTTTTTGTATCCGCCTAAGGGAATTGTAATCTGGAAAATAACTTTTGCACTTGAAATATGCAATAGAAATCAAGCAGTGCTTTGTTTATAGTCAAATCCGATATAACAAAAAGCTACATATTGCCAACAAAAATTGTTACTGAAGTCTGTTTTTTTATGTTCAAGTAGGAAGTGGAATTGTAACTAAGTCCTTATCTAGTACATTTCTTGCACATAATGGTTTTGTATGCAAAACCAAAATATCGTATACTACTAAGTTCTTACTTATTCAGTCTATGATTTAACTAAAAATGTAAGTTACTTTTTTGCCATAAAATTTTCAATAAGATTTTAAGATATTTTTCATATTTAGATCCCTTACATTTTACCCATTGCTTGAGGATTCCGATCTTTCACTTGGTTACCATCCATCTTTGAATTTTCCATTGGTCTAAAATGCCTTTTTCATCAGTTATTAATTAGAATGTGGTATACCATAACTTAAGGAGTGAACTAACTAGTCAGCTAAAACTATATCGGTGTAAAATGAGTAAGTACAGTATTCCTCATAAGCCCAAATTCACTGGAGAATTGCATGTAATTTAAACAATAATTGATCTCAATTAAAATTAGAGTTTATATGGCACTTCTTATTTAATTATTTTCTTTTTTGGTTTCCCAGTGTATTTTATCAGAGGTCTATTTTCCGAGAGGGGTAATATTAGGCACTCGTCATAGTCAAATGATGTTGTACTTAGGGTTTGCTAAATCAGACAAGAAATAGTCTTGTTACGTATTGGACTATATTGTGAGGGTCTTTTTGTATGATTGGATAGAATGTTCTGGATATCGAAAGGCTTTATTGCCTTCAAGTGAAGTTCATTTGCCAGTATATTTTTAATCTTTATACACATAAGGTACAGCGATGAATCCAGGAGAAATTTTATTACTTTGTAATTGTGGATGTTGTAATTGAAAATTTTACTGTAGGGAAATTTGTTCTTATATTTGTGATCTGAAATGTCATATACTGTCATAAAGATAAGTTTCATTGGGTCATTGCTTTCCTTCATGTATTTATTTTTTTATGTTTGTATATTAGCAAATGCAAGATGCAGTCTGCAATATTCTGCAAATTGCATAAGTTAGAAGACCTTTTCTTGTAGGTACAGAAGTGTTATTTTTGCAGTTTTCGTAGTTCTTGAAATTGCATTTTAAATAAGCATATTTTATATCAACTGAAATATTAACTTTAAATTTCTCTCTTTTTCGTGTGAAATGCAGATATTCTGTTGTGAAATTAATTGAGGAACAAGAACATTAAGTAAAAGCAAAAACATCAGACTGATGTTTGTGGAGAGTATAATTGTTCCTAAAGTAACAGTTTAAAAATATTTTTTTAATTGTTAATTTTCTGAGTACAGTATAAGTGAAGTGTGTAATTTAGTTACTTTTTGCATAAAACGATACTGTACATGACTGGTTTTAGATTGTATTGTATATTGGTAATTTTTTTTTTTTTTTTTTTTGTCACCATCGTAATGTTCTTTCCTAAACAGCTTAATTCTGGAAAGATTATGTCTATTTTCTTCTTGAGATTCTTCTCTTTGGTCCAATTTTGAACATTCCTCTTGTAGCTCAAAAAAAGAAAAACAACTCTTACACGACTGTGAAGGCATTACAGTATTTAGTAGTCGTCTCTTATTTATGCACTTTACCCCTTGAGTCACTTTGCTTTATGTTTGACACACTTAACACTTTTTCTTCTCTTCTTGTTATTGACGGTGCTTTATGATTAATATCATTTTGTAGATTTAAGTAGTTCTTTGTCATTGATTATTGTTGTTCTTCTGTGTGATAGTTATGGAGAGACATATCTTTTCAATTTCCTTTGCAAACGTTAGTAAAACTCACTGATTTATTATTGGTATGTGATACACATCAACAAATATTCGGTACAGTTTATGATGAACAGCATGAAGTGGTGGTGGTTGTATCTATGGATATTTATTTATTTTTTATTATAGTTATCTTCCATTGATATCATTGCTATTTCCCTCTGAATTCTTTTCTCACTCGGAGATTAGTCTGATAGATGTACATCATTTTTTATTATACAGTATTTACCTCCTGATTTATTTTTATAGATCCATATTTTATTCTTTTTATTCAAGGCAAGTGAATAATCAAAGGATTACTGGTATGTATGGAACCAAGCAAATCATTTATTAACCTCCACCAGCTTCATTTGGTTTTATAATTGTAAGCAATAAACAGTGCTTCATATCCTGTAACACTTAATTTCTTTGCTCTTCTGTACAAAGATTTTAATTTGAACCAAATAATAAGATTATTTGCAATTT

>chr32:+:60111232-60114134

TAGTATGTCATGTTAGAAACTGTGTACAGTATATTGTTGATTTGATTTGACAGCTCATTAGTAAACAGCAAAGAAATTCTAAATGTCATGGGCAGAAAGGACTGTCGATGGAGAATCGTGGCGGGGCGTGTCCAGCCCCTCCCCTCTTAGTCTCACTAGCTGCACCAGGACCCTTTAGGACACCTCTAGGACCCTCAAAAATCACGACAAGTGCCAGGGCACTGTCGTGAGTGCAGCGTACAGCCGCTGCCCCAGCCATTCCATTTGAGACTGGCGAATGCTGTCGTCATGCCCGGTGCAGTGGCGCAGCTTAACCTCTCATCTATTGTTTAATTCATAAATAAAGTAAATTATAGTCAAGGAGCTGTGAATTTGCATTGCTCAGTTACGTGATTTGAATTAGTATCTAAACTTTACTGAGCGTGACGCAGTAGAACAAAACTAAAAGGATATTTTTTTTTAAGATTCAAGAACCAACAAATTACAAAGGTTTTGGTGTCGAAAGTGTAGTGTGCATGGTGGGCGTGGCCTTCCTATGTCTCGATATGGCTACTTGAATGGGTATTAGAAAAAATGTGGTATGACACTGAGAGTCCTATCTTTTAATATGCCATTGCAGCTAGCTACTGATTTGTGCAGGGCAAAGCCTGGGAAGAACCTCACCCTCCATACATACATACATTCATATTTACATACACATCTCTGAAGGCGAGTATTTTTAAATCACCTCCTTGAACATCTTGGGAGAGCGTAACCAAGAACAAGTACATACTCGCTGATTCACTATAGCAACTATTTGATACAGAGTAGCCATGCATTGCATATGCTTTTTTATGATTTTTGCTAGGGCTGTGGATGGTTGATATAGCAATATCTGACTAATTTGTATATGCATATGAATCGGTGTAATGAGTCTTTCTGTTGCGCACGTGTCTTATTGTTAATTTGTTATACAGATGGTTAGGTCTTAAAGTTGCCGTCGGGTAGGGAGTGGCATCACTGCCATGTTTGGCCCTTGTGATACCTGACGCGCACAGATGCAGTAATAGGGCCTTGGCTTACTAGCGATAGTGCTCACCTTAATTATGCTACACCTGGCAGTTAACCCTCACTCACTTCATATGTGTTTGTTGCTTTCTACAAATGGTTTGCAAAAAATAAAAAAAGAAAATCTCAGGGCATATAACCAAATCTATTTTTGTTTTATTTTTGGTTACTCCATGTATTAGGCCCATTCGTGGAATAGCGGGAGATAACCTGGATGGTGGGGAACCCTTGTGGATGGTGGTGAGAGAAGCTGCCTTTTATAAAATGGCACCCTCTACCTAGGACCATGCCTCCCCCCCCCTCCCAACCTCCAGGGACCACTTAATAAAAGGTTAAGGCTCATTCGCATCGCTTGCAGCTACGACTGCTTGTAACCAGACAATCCAAGTAAACTCTAAAGGTGCCTCGAGCCATTGTAACAAACTTAACTGCCACATTGGGGTATACCAGAATTTGAGGGCTGGGCTAGTCCCTACCCCTTTATCCCTCTGGACAGACCCTCTCACCCCATCCCTAAGGCACCACCACCGTCGCCGCTTTGACCTAAACACCCGACCCATCACCTGACCTGCCCTGACCTGACCTGACCTGACCTGCAGCTCACTACATCCCTCTTGCTCTCAGTATTTGCCTTATATGAAACTGAACAGGGTCGAGCAGTGTATTACAATAGAAATGTGTACACTTAATATCATATGTCTTTCAGTACTGTTTTCGGCGACGTTGTTTTATACTGTTTTCTCGTACGGTATCTACTTCTGACGCCTGAATTTGTGTCCACGCAGCAGTGAGGTGAGCTGCTGGTGACACTGTTCACTGGTCCTACAATGACCCCAAGGCTACTGTTACCTGGCTCTCTCACACATTTGATCTCCTAGGCTAAAGTGCCACCACACCTGGGAGCCAGCTATAGCATTAGGTTTGGGTGATACAGCCAGCCTTAGCACCATCGCTAGTCAGCTAGTTACCATTCATAAATTATATATTATATGATGGAATGCTAAGGCTCGGTGAAAGGCAGCCCATTTTGCGATGAAGTAGGATCAGTGCGCCAGTGCCACTTGCAGGCACAGCATGTGGGCCACACCTCTCGGTGAATATCTTCAGCGTTAGCATTACAGCTAGTCAGTGCAGGAGAGGTTACTTGTACTTTACAGGGGGATCGTGTATTAGGCACAGATTTACATATCATTGTTCTTCACGTGTCACTATGTTCGCGTGTAGTTTACTAGTTGTATGCGTGGCATTGCTTTGCCCCCATATGGAAACTAACAGTATTACTTAAGTGCCAATTATATAATATGTTAACCTAGTGCCAAGTTATTGCCAAATCTTGCACTAACAGCATTGATAAAATGCTGGGTGCGCTAGTTTGTCCATGTTTGGGGAGAGTGTGCCACGAGTGCCAGGCAGCCTCTCAGGGCTCGCTTACTGTATGAGCTTAGTTTAAACATCCATATCATATCATGTGGGTACTGTCTGAGCCATACAGGCTTGGGTTAGGGTATGTGTTTCCTCTAGGCCTTCCATAAGATTCCCACAGTGTGGGATGGTGGCCGACAGAGGAGTGACCTGGAGCTGACTCTACTGCGCAGTTCCTTGCAAGGCAAAGGGCTGTTCTTTTAGAGAGGACTAGTACAATCAGAGGATAGTGAAATGCCTCATTAACACAAACAGCTCTAACCAGCCCTGACTGGCTCCCTGACACATTCGCCATCTTTACTGAGAAAAGTGTTTCACAGTTTTAATGTTATGGCAATAAATATTTGAAGGATGTAAGCAGTTAATGATTTTTTTATTGATTGTGCATATGTGTCTGTCTGACACAAGTATTGTTTGAGTTTCAATAAATCTTC

>chr34:+:5390401-5392882

GGCTGATATAAAGTTACAGTGTGTAACACATGGCATTTGGGGCTGCCTACCCTTCTGGTAATTATTACCAGTGTCTCCCCTGGGTTCTGTGGGGGGAGATATGGTTGGTGTTTTCATTCCTGTGTATGATAAAAATATTATTACTGTACCTTCACAAGGTACTAAATCCAAAATATAGAACTACTGGTACTAGCAGCTAAAGCCGTTGTATAAAGACTTAAGAAAGACTTGTAAAGCAATTCTTGCCTAATTATTATATAATGAATGAAGCGGTGTGCCTCTAAGCCTGTCACAACCACAAGAATCTGTCTGGTGGTCAAGTTCTGGCATGTGTGTGTGTGTGTGTATTAACCTCACACAGCATTTGTGACCTTGGGCTTAGGTGCACTACATCACCCAGGCTGTGTAAGGAAAAAACCAGCTATTGAAAGTATTAACTTTCAAGGGGGTGGGGGACAAAGCTGGTGGGGCGTGCGTTGTGTCCTGTATATTTTCTTATTTATTTGTTAATTTTATAAACACTTGCCTCCAATGCTATTACTATATCATGTCTTAATGCTTGTTACCCTCAGTGTTTCCAGTTAAGGCTAATCACTTTTTAAGATTTTATGCAAAATTGTTTTTAACTAAAAAGGGTAAGCATCGCAAATTCAAGTTGCTTCTCAGGAGTGTCACGTTACGAACAGTGTAAGCCGAATCTTGCTTCCTCAGAATATCATTTTGCCCATCTGTGATTTATACTGATTTCTTTAGCAGCTTTAGATGCTTTATTATAAGGGTCCCTGATATTTTTTGTAGAGTATTTTGGATTTTGTACAATTATTATGATTATTAAAATTTTTTTTATCATTTTCATGCCAATAAAATCTTGATTTCTGGCAGGTCTGACTTACTGTGGCCAAGAAAACACAAGGGCAAATGGTCTTTTGTAGCTTATCCACAGAGGATGAGGGTCAACAAGCCTCAGTATATAAATGGGTATTGTTAAATTACTCGGTTATAAGTACTCTACATGTATTACTAATGATTTAGATGCAGTGTTGGGCTTTTGGTACGAGTACATGTTGCCCATTTATTCTAGAAAAAGGAATGTAAGTTGATGCAAAGAGATGGTATTTATATTTCAGTGTTAACCATCATATCCCATTGGAAGTCCAACATTTTAAACCTAAATGCCAAAGCTTGAAATTTTTTCTTTGTATATATATCTTAAACTTAGTTTCATGACTAAATAAGAAAATCTTTTTATTCCTCTGTTATTAAGGACTTACTACTTCTCCTTTTATCCCTAACTCGATTGTTACTCCTCTGTGCATTTCAGATGCTGATGAACAAGTGCACTTCCCATTTTGATGCATTGTAAGCTTATGTTTTGAAATTGCTTTTAGTGGTGGCAATGCATATACACTATATATTCTGACATTCGAAGGGTGCCCTTGTTCCAGTACAGGTGAATTGCATTACAGGTGTACCAAAATACTGATTTATTTGTAGGGAGCCACAAGTGTGATAAGTTGTGCTTGGGCTAAAGGGGTGGGTAATTGTACATGTTTAGAGGGACCTTAATGGACATCGTTTGTAGCAGCTTTAATGAGTGGAGAAAACTTAAAATATGCCAGTGAGAGGGATATGCTACTGACTTTAGAAACTGAAGTACATTATTTATAATCTATGTAGCCAAGTTATTTTGTTTACAGCAAATGCAGTTTTACTCAAAGGGTACCCAAGAACAAGGTTCTCGCTTAATTTTGATCTTTTTAGAGCAACAAACTTATATGGGAAGCACAATTCAGAGCTGTATTTGCTCTAAAAATAGATATCTCACTAAGGTCCACATGCTTGTTGACTTCCATGAGCTTCGTTTATGTAGAATGAGTATAATATCATTAACAAACAAAACCTGTTAAAGAAGTATAAAATTATTACCACCATTATTCTTGGCAACAGTACTTCATGGGATATCAGACATAGTACCTAACATTTTGGGAAATACATAAGATTTTTTGGGTATCATGTCTGTTTAAGAAGAAATAATCTCCTAATGTATATTTTTTTATGTTTGCTTGTTTTGGGGACCTTTTTGCAAGTGATTGGAAAAATTATAAATTAGAATATGGTGATATAGGAATGCCAGATTTGTTATTGTTTATATGAAATTACCTCAGCAATGTTCTTGTTATAGTCTTATTTCCTCTCTCTTTATTTTAGTATTTTCTTCAATGAAGGGTAATATAGGCAAAACTTTTAAAACGAGTGTTTCTTTCAAGAAACTGTGATCCTTTGGTTCTAATGCGACCAAACGATCATGGAGATTGAGGGTAGCATTCTTTCAGATGTTTTGTTGCTAAAGTATTGCAGTGCTTTCAGTATGTTTTCCATGAAAATTTAGCCAGTGAAATTTTTCTTTGTATTATAGGATTATTTTCTCAGTTCGCCATAGTAGAAAACTGTAAAATATGGAAATATAAAATTTGTGATAACT

>chr21:+:57312959-57314680

AACTTATGAGATGTAATGAGCAATAGGAATAAAATAAGCTGGGGAAATATCTAAAGTACAGTGAAAATATGATGGAAGACAACTTTGTATTTGAGAATTACAGGTGATAGCTTGATTGTATTTTAATAGCCAAAAATTTGACAAGAAATCATTGCTTGCCATACAGTGTGCACAGTTAGATTTAGTTCCTCCACAGTTAAAATTAAGATGGCAAATTGGAGATATGTACTCAGATATAAAATATATCAAAATGTTAAGGAAAGGTGGAAGTTTTCATGAATCATTTAATGAGATGAAATTTTTCAGTAGCCTACACAAAATAAAGATGTCCTGAAACACATTGTATAGTATTTTAATGACTTCAAAGTGTCTTCAGATGCGATGTGTAGGATGTAATAGAAAACTAGGTTTAATCAGAGAGAGTCTTATTTTTAGGGTGTATTTTAATGACTTCAGAGAGGCGTCATATTTGAGCTGTAGGCTGTAAAAAAGTTGGTTTAATTGGAGAAGGCTTGTTTTTATGGTAAAAGTAGCTTTTGTCCTTTTAAGTCGAGAGATATTTCATTACAAGGCAGACAGAGGCCAAATCTTTCCCAACAAATAGAGGCACTATTGTTTTAGAGGAATCTGGTTCAGTATTTGTACTTCTTTTCAGGTAATACAAGTTATCTGGTTGCATAAAACTTTTTGTGCTTTATGTTCATTGGTCAGTGTTTCATAAAAGAAAATGTTTATCTTGATGAAGTATCTTTAAACTGATTTAACTATTGTGGTTAAGCTTGTTCTTCCAGATGTATTTTTTGACTAAAATTGTCATAGCACCTTAATATGTTTCTGTTTCTATAGTTCAAATATTGTGCATTACATTACTACTTTGTAGCAAGTGCTCCACCAAGACCATTTTTACAGACTTGTGTTACTTGGTCCATTTTCTTAACATGCATTTAGCTATGAAAATTAGGCTTTGTGCATCTCCTATAGCATTTCTTGTCGTACTTCACTCTCACAAGACCTTGCATCAAAATTTTGTCATCCCCAATTTTTTTTTTTATTATTATTAAAGAATTGTGGGCAGCTAAATATTAATGAAACTAATTTTTGAATTTTTGTTCAGCTGTTGTTGACATTTTGCTTTGTGCTAAATTATTTATAGTCCCTGAGAAAAAGTACTTTTTATGTGCTGTTTGTTGGACCTTTGCACATTTGCTAAAACGGTTTTTAAAAAACCACCATGATTTTCATATAAATGTTAAGTATATACCCACCGCATTTATAGTTTCCAGATAGGATTTTTAAGTTGAAAAATACTACAACGTGGGTATACTCTAATGGCTTTTATGCTTATAAATTTTTTGCATTTTCCGTTAAATATTTTACAGGACTTGTTTGCCAAATCAAAAGGTATTTATTAGTTTTTTTACTTGAATGATGTTTTTGTCTACCTATATATTTTACTGGGATTACTATTGTACTGTATATGATAGCACCATTTTTCAAATAAAAGCTCATGATTATCATGAAGTAAATCATTCTAGTTGTCACATTTTTGTTACACTACAGTATGTTATAGCCACTGTGTATGGTCAATGTCATTTGCGTTATGTTTTGTATGAGTAAATGGTATGTATGTATATACAGTATATATATATCTTGATGATGTAAATAATGGTTACAACTACCAACATTTTGTATCTGGGGAGTTGAATAAATGTATGTTGAACT

>chr3:-:81034822-81034877

ACTTCTTCCCTGTTCGTGTGAGTTTCAGCTCTACTCGGCCATACTGTGACCTCAAG

>chr3:-:81031606-81033187

GTCTTAGGTGTAGTGAGCACATCAGATGGTGCTCCCATCACTTTCTCTCAAGAAACGCAGTTGAAAACAAATGTGTACGAAATTGATTAGGGTTGTTACTTGTTGGTTATGGTGCACAATAACTATTAAATTCAGATGTTTTATTACTACAAACTTAATCCATGTACAGAAGGCTGTAGGAATAGGTTTCCCAAGCCATCATAATGTAGTTTATTGTTATCTTCATTCCTATTCAACATTTTCACTGCTGATTACCGGTAAGTGTATCAGTTTCATGTGAGTAGTTAGCAATCTGGAATGACAATACATAAATGCTTTCATCCTAAAGACAGGAATTCAATAAGTGCTTAAGATATATTGTACAGAGAAGCCATAAGTTCTGTATATATGGTACACCATCCCTCTTTAGTTTAGTTTCTCCCTCACACTCCATCTATTCTCTTAACAATACTTGTCATGTTTTGTGTCTTCTTAATGCAGTCTTCTTTTATTTCTGACCACATAGTATGACACCAGTTAGTGTCAAGTTGTTATCACTGTGACTTAAATTTTCCCTAATTTTCTTAATAATATGAAGCACCAATGTTGGTAGCTATATTGTCTAGTCATGATTGTCAGTCATCGTTGGTTACGAAAGTTGTTGAGTCATGGTTTTTGAAGAGACCTCATAAGTCATATACATAATGAGAAAAGTATCTGATGTGAAGATGATATTGGCAGTTTTAATATCTTTATGGCTTATCTTGAATCTTGGCATGGATAATTCAAAGGATTGGTTTGGGCTGAGGTCTTGAAGTTAATGTTTTTCTATAAATTTTGGGTGAAAACTTTGAAGATATGGTCAGGAAATAAGTTTTGACTGTCTTTCTTGCGTTATATCCTGGTATTAATTTAGGCTTTTTCTTTTGATGTTCCCAGTATTACTTGTTCTTTTTTTTTTCCATGCTGAATAGGCTTCCTCCTCAAGGATTTAGATGGGATGAGAGGGACAGATGAATTTGATTATAGGTGGCGCATGCTAACCACATTTGATGAATACTATTTCATCTCATATGATAAGGATGTATATAAAGATTGCCTGGTAGTTAATGCACTTTTCTATAGTACCTTGAAATGTGAGTTTTGTGACAATATTCTTTCATTTTTACTACTGTGAAATGGATCTATTTTTATTGTGGGGTACTCTATTTTAATTTACTGTTAAGAAACATTTACTAAAAGTTTATAAATCGCCTGAGGTGATGATTTTGGTAAATTGCTGGAACTTTTAATACCCAGCTTGTTTGTTGATCAAAAGGAGTCTGACTAGGTTTAATTTGGAGCTTTGAAATAATACTCTTAAAAATTGTAATTTTTACCAGTTATTGTTGCAGAACGTCCATTTAAGGTACACGTATTTAATTCATGGCTTAAGGGAAATGAGGTACTGTAGGTACAAGTTCTGAAGCAGCCTTTGTCCAAATGTTGTATTAAGGTTTAAGTTATGAAATTGTTGGTAATTTATAAGGAAATTTACTGGTAAACTTGAAATATTTTTTTTAATATACATTGTATATGATATTCATTAAAGATATGAAAGCCA

>chr49:+:13373809-13375373

ATACATTGAAGGGATTTTTAAAACATTCCTTTGTCCTTGGCTGCAATGATTTCTGCCTTCTGCATCTCATCTGCTCATCCTGTTTTGCACTGCACAGTCCAAATGTTTACTTCTCTTTTGCCACATGTGCCTTGTGCTTCATCAAGAGATGCTCATATCCCCTATCTGCTCTTCCTGCACAGTTTCTGATGCTGTAACTGACAAGAATCTCATTGAATTCAGTTTAGTTACTTGCTTGATACAGATATCCCAAGGTCCTCTTTTTGTAACCGTATGAGTAGAATTGTTTTACAGATCTGTGCAGTAGTGAGGTCACTTAATCCCAAAGTTTATCAGACTTGAGCGTCATCATTTTGCATCATTAATTTATTTTTCTTTACAGGATTTCCACCCGATATTTTTTGTGAATGGGGTCATCTAATATTTGAAAATGCCTTCTCGTTTCCAACCTTGTTCTAATTGTTAACCTGCAAGTTGACAGTGCCCCATTATTTATCTTTGCTGTGCATTTATCAGCATTCTCATAGCCTCCAATGCCATGCATGCAGTATAATGCCTTTGTTCTTTCTAAATTTGTAACTCACAACTGCTTTTTACTAGTGTCCTGGAATCCTTTTTGATTATCCCCATATATGTAGTTGATCATGTTCCATGGAGAGCAGGTAGTGTCTCCTCATTTGAATAAAGTTTTGTATCTTTGTAGAAATCCAACTAACATTTCCTGTAATAATTTTATACTGTGGCAGGGTATTTATCATTTTACAGTGGATGTATGCTTCCTTACGTAGGGTAATTCACTGCATGTAGGAGAGTGGTAGTATGGGTTAACAGCACTAAATGGAACAATATACTTGCATGTTACTCTGAAACCATAACTAAATATCTCTTGGCTGTAATCTTCACTTTAATTATCACATATGTAATGTTCTGTATCCTGGTAAAGTTCTGTGGAATTTTACGAACAAGATTTCACCTGAGAAATCAAGAGTGGAAGTTAAGTATTTCATATTTTCCCATTATTTTTCAGTGACAGAGAACCCCTTCTTCCAAGTGGAGGTGATGATTCTGTATTTACCATTAGTGACTCTGTACCTGCTCCAGTACACCTACCTCAACAACACTATCCTAAACCTTTCAAACAGCACCAATCTCAGCATGTTCCAAGGCCCCTCCCTCGTAGGACTTTAAGCCACAAACCAGAGGTTTCTCTGATAGCTGAATTACAAAATGCATTAATGGCTTCATCACAGCATGTCCCTGCACTGCCTTCAGGAAACATTTCCACTTTGGAACCAAAATTTCTTCCAGGAGCCAAAAAACAAGAAACTTTTAAGACCCCTTCACATCTCCCTGTTTTACCTTCAAAAACCTTTTCCGCAACAGAACCACGGCAAAAACTTATCAGGGCTTCATCACATGATGTCGCTGAGCCACTCTCAAAAGTCACGTTTACAACTGACCCAGAATTTCTTCCATACACCAAAACTCAAGACACTTTTAAGGCCCCATCACATTTCCCTCCACTGCCTTCAAAAAGCATTTCCACAACAGAACCACAACTTCCT

>chr15:+:21919793-21921020

GGGCCGGGGGTAGATGCATCACCAAAAAAAGCTCCAGTCAAGAGTAAATAACATCCAAAACACGTATGCTTTTTCTCACTTGTTTATCAGTTTTTGATAAAAGGTGATCTGTCGCATGTACTCCCAAATGCCTGCCTGTCTGTCTGCTGGGTTTGTCTAAGACCAGTGTATGAGAAAGTACGTTTGGTGCTTGATATTAAGTAGCTATAGGTCAGATTTTTTTTATTCACTTCAAACATGTGATGAAGTCTAGGTTCATAGGTCATAATTTGCCACACGGAATCCTTTTTCAAAGGTCAATTGTATATCATGGCTCAAATTTGATCCTTAACAACAGTAACCCTTATCTGTATTATCAGCTGTCCTCGGAGGGAAGTCAGGAATTCCTATCGTCTTGATCAAGCTTATGTAGCAGTCTCATGTTTACATATAAGCCATATGGTCCATCCCCCTCCATGTAGGCTTAATTCTTGGAATAAGCCTCGTAGTGGGATACATTGTTTTACTTCTGTAAGGTTAAATGTATAAGTTGTTGGAGTCATTAAAGAATGTTATTTTAACTTAACTGAATTATATAACGTGTTTTTGTGAGCAAACTATGTGTTAGATAATAAGGAATTTGCTGGTAGTCCATTATTGTAATGTGACCATTGTGTACAAACAGGTGTGCAAGCGAAACGAATTGTGGTGGCAGTAAAATGAAAGAATAGGACACATGACGTACTATAAACAGCAGCAGCAGTTCTTCCAGAAAAAAAGGTTTTATAATGGGCATCACATAATTGAATGCCCGTGTAACCCTTATTGTGTGACATACCATAAGAACAAATGTGTCGGTCACAATATTAAGTTATGGTTTTGCATGCTCAGCCGACCTTTTATTTGTTACGTTAGGTGTGGTCTGGAGTACACCCATTACCTTAGGCCTGGTTGCGTGGCGCCTCTGTCCTCATTTGAATTTGAATGGGTCCCTCCCTCCCTCCCTCTGTCACTTGTTAGGTATGTCGAACTCCAGCAGAGCCCAAACCTTGCTATACCCTACCTTTCCTCCCCCTCCCCTCTCCACCCCACGACACATCCCAATTTCAAGTTTTTCACGAAATGATTGGCCAAGTTTCGGTTAACAGTAAACGTCACGTCTTGTAAAGTAAAATCATCTTTTCAAAGAAACCTCGCAAATCTAACTTCTCAGATTGATAAGGGATTCGAGTGTCTTAATTGACTACGT

>chr19:+:74055970-74058362

GGGTTGATATTGTACTTGGACTGCCCACTTATTCCATTTGGAACACTTGAAGTCTATTGGATGTCTGTTTACAGATTGTAGAACAATCATTTGATGGATATTGTGCAGCGTTGTTGTGTACATAAAATTTGGAGCTTTTGCTCTGTTGTTATGATTATACGTACATTTAATTAAGAGGGTTGATACGAGTTCCCGAAGGTGATGTTTGTCGTACTGTTAAAACCATTTTTAGATGACACTACACTTTTCTTTGTTCTCTGTCTTAGCTGAAGACTCCCTTTCCCTCCTGCTTAAGTACTTTGAAGCGGACAGATTGCAAGTTTCCTGTGTATGTATTAGTATGCTGTTGTCTTGCTTGAGATAGATGTAGAATATAGAATCCCATAGTTAAACGTGAGTAGTGAGCAGCCCACTTGGTACGAACATGAACTGAGAAACTTGTTGATGGATTAATTTACACTCGCTGCATTCCTTAGGTTTTTACATTTTTAGCCTGCATTCCTTGTATGATATAGTTTCATAAGGCCTTTGTATTATTTTCATGAAACTTACAATTTTGAAGAACTTAAGCAAGGCTTTCTTTTAGTTTTTGAGATTAGGTAGGGCAGCAAAACTTTTTATTTTATTTATGTGTACATACTATTTATTTGTTTATTTATGGATTTATTTGTAATAACATATACATATTAGTTGTGGTAGCTTTATCACAAGGATTTGACTTTGGAGGAATTTTCATTTATGCATTTTATTTATGGGGGATCTGATGAATGCATCTTTTGTTGGTGCATTAATATTTAATCCTCTGTGAACTTAGTTGCTGGATAGAAAGAAATCTTTACATTTTTAATTGACTTATTCAGAGCTTTGAGTATCAGTTTTTGTAATGTGCTTTCCTTTGATATTGGTCGTTTGTGTTACACAAGAACGTATTTTAGATACTATAATGATTTCACATTTTGTATTTGTAAAGTGAAGTAATTGATGGTGGATGGTCATCATTTTGGTAGGAAATATTGTTTCATATCAAGGTTACTGTTAGCATTCAAAGTCAGATAAGGTAGGGAGCTGACATTCAAAAACAAGAAAAATCATTATGCAGTCGTTATCTGATAAGACTGAATGAATCCATAATTTGGGTAGGGAATTTTTCATGTTAGGGTAACAATGAACAAATTTGAAATTGAAATCTATTTGGGACTCTTTTAACATAAAATTAAACTAGAATTAGTAACCTACATACAGTATGTGGTGGTTTGCAAATATTTTTGTATTTCTTTCATGTCCGAGATTTGTTAGCTACGAGCAAATGCTGAGTAGATTCAAGTTAGGTGGAGCCAGATTTCAGTAAGGAGATTAGTGTCAATACTTTTAAAGTTTTTTCACTTATTAGATCATCTGTGAGTCTTGAAAGTATTGGAGAATTATTCATATTAAAAGCTGATGGTGTACTTTTCCAACAATATTGATTCTTGATCTTCTGATTGTCATTGATGTGGCTGGCTCTTTTAACTTACATCATTTGATGTAAATTCAAGCATATTTTGTTTGTGAATGTAGGCAGGCTGCAGAAATGTCCTGTTTCATCTTGTGCTAAAATGTATCTTTTCTAACTGTACACAATGAGACGTTTGAATATCTGTTATTAGCACATGATGTGGATGTGAGTGGAAAATATGTTGAAGTATTATTTTTAAAGACTTCAGATGATTTAAGGGGTCAAAATTCCCTCTTTCACTCTCTGTCCTGTGTTACTGTTCAGTTTCCTTATTTTTATATGTTGTTACTATTATGGATATTAATATGTAGCGTGCTTTTTAAAATTTTATTTCGTTCAATCCTGCTAATGTCTTTTACTCCGTTTTATTGCTGTAGCTTTCATTTTCTACTTTGCACATTTTCTCTAGATTTTTCTTTTGTTTTTAAATATTTGCTCAGGTATGGTTTTATTCCTTCATTTGCCATTCTGTTCTGCATCCATTTGTCAGGTGTCTTGGTTGAAAAATTAAATGATTCTTTTGGCAGGGTATGTATTTCAAGTACTCTCTGACTTAAGCAGTGACACTTTGATGATTGTCATTCACTGCCCCTCCATTTAAAGTGCTTCTACACCGTTCTTGAATATTTTTGAAGTGTTGGTGAGTATGAAAATTAATGATTGATTATTTTACTTTGAGATTTGTTTTTTAGGGAGGATAGATAATTATCACCAAAAGAAAATGACTAGTCATGAAAGATTATGCACAGTTTTTTCAAGTTTAACATGGAACCCTTTATCCATCCTTGAAAGAATTCATAATTGAGTTCTTATCATCACTAATGTCTATGGTTTTTGTATTGGTCTACTGGCATGTACACCTCTTCACTTTTAACTAATAAACACTAACTGCGTTATT

>chr44:+:32578615-32579175

CAAGAAGGCTGGTGGCATCAACCTTGCCCGCTACCAGGAACTGTATGCTCAGTTCATCTCCAACCCCGACGAGAAGTGCAACGCCGTCTACCTTTTCGGACCCCTTAAGGAAGTCAACTAAATAGGACGTTTAAGGAAACTTAGACGCACCTAACGCGCGAGGCCCAGGCGCTTTGGGTGTACCCACCGCACGCGCTCCCTGGGGTCAAGGCTTCTCTTGTCCTCTTCTCCTCTCCTGAGGGGACTGGATAATGATGGGCCTGCCCTAACACACACACACACCCACCAGTGCCCAGTTCCTCACTGCATCATTTTTATACTCATCTCCCTCTGATGCCAAGCCGCACCGTCAGGCATCAACACCGCCTGCGGCAGTCGACCCGGACTCATGGCCATCCTTAGCCGCAAATCATATTTTTGTATTGCTGGAGTTGGGATCCATGCGGTACCGCCCCCAGCAGGCCTCCTGCCCTCGTTGCGGGACCAGTGCTGGAACGCTATCGGGAATGGTCGACTGCTGTTGCGATCATCTCTTAACCAATAATAAACTCTACTATGTTT

>chr35:-:25619339-25619405

AGATAATATCGAGCAACAGCAACGGTATTACCTTTAAGTGCGAATTTTGCTTCCTTGAGTGCGTGAG

>chr35:-:25618916-25619033

CTTTTTGATCAGTGGAAGATGGATAGAAAGAGTGAGGGCAAGTATGCTGGTACTGGCGACAAGGCCGATTTGGACAACCACTCTAATCAGTGCAACCCTACCAACAAGGAGTATGCAG

>chr35:-:25618696-25618779

GCCACAAGTCTGAATTTGGGGGCAACAAAGCCGATGCCGACAACCATGCCAACCAGTGCAACCCAAATAACCCAACCCATAAAG

>chr35:-:25617966-25618397

GAAGCAAGTAAGCACTGGTAATTGTACTTGGAGTTTCACAATCTTCACCGTTACCATCAGGCACTCGACGACCATATTAAAAATTAAGATACTAACTTTCCTGAACTGCTACTCCATCAACAGCGTTCAGTGAACTTCAATCTAAATAATACAACTATTGAGAAATAAACTTATTAGCTTTAGTGTGATTATTGTGTAAATTCAAGTAGCTGCAACAACCTTTCATTTTACTGCGTTAAAGTCGAGCTGCCACTAATTTGCAAATTTGAATATTTTTAATTTTGAGACTGTAATTTACTATCTTGAATACTTAATGCAAACATTAATGCAAACATGTATTTTACCATCTTTAGTACCTTGTGCAAACATGTAATTACCATCTTTAATATGTAATGCAAACATGCAAATTGATTTATTAAAGTAATGTTATTT

>chr27:-:7427287-7427368

CGACCGACGACGTAAGCAGTAGCATATCACAACAGAAGTCCGTCTGCTGTAGACGTGTGAACGGTGTCTGCCAGATTAAAAG

>chr27:-:7400767-7403294

GACTTGCAGAATGTACACTCAAAACTGATTGATGACCCCATGATGGCTCGGTTCTCATAATTTAAGACAAAACAAGATATTCAACTTTGCCATTGCCTGTATGAGTTTTGTATTGGTGAAGGGAATGCAGATTTGGTGAAGGGTGTTCTTGTTACTTTGTTTTACTCAGTCACTGAATTTAAAAAGAATTATTACCACTAATGTAGTCATTATGGTGCTTTTATTACCAAATGGAAGAGTTTTTAGGTATGGATAAAGCTTTGTTTTGCTATTCTTTTCATTAAGTGGTACACTAGGTTTGGTAGTGATGGAGTTTGGAAACTTGAATGGAAGTGCAGTGAATGGCTATTGCTCTTGCACAGGTTTACCATTGTGTTTAAATGTCCCATTGTTATTATTTTCATTCATAGAGGATGGATATTTGTAATATATGACAGTTCATATCTGTGTCCAGCGAGAACAGGAAAGTTGTGAGCACCCACATCACTGAGGGAATGATTGTGATATAGCTACTTAAATATAATGGATCTCATTATTATTTGCTAAAATTGGAATCTCTTGGGAATAGTCTCATATCCCACAGTTCCTTGTACGATTCACTGTGAATCATGCTACTAGGTCAAGTTTCTCAAAATTTTGTAACTTCAGATGTGCAAATTACTTTTCAAGACTTCTAATACTGTTCCAAATGGAAGATAAGCTTGCTAAAGGACTTGAAATTTTGTAAAGATGTTAAGTGTACTTATTGCGGCATTAGAACGGGTGGTGTTTCTCACTTGTTAGTGACTAGCTTTAGGTTTTATTTGTGGCATTATTTGTAATGGTCATTCATTATAGTGCAATAGTATTCATTAGTATGGTTCAGTTTATTCATTATATTTAGTGATGCAATATACTGTTTTTTGCAAGCCTTGGAAGAACCTGTTACAACTGCAGTACAGTCCCCCCATAAACTATCAAGAGTAATCTTTACTATGCAAAAAAATTACCGTATTTTAAGGATTGATAAACAACCATGCTGAAACTTGAATATTCTATCTGCAGTCTACCCATGTATCATCAAGTTTCTCTGATGTTTAAGTAGATTAACCAGACCACCGAGCTAATGCTCTCTGAGGGCTGACCAAAAAGGTATGTCTGTACTAATGATATGATTACATTTTTTAAATTCTCAGTACTGCTGTGAGTTAAGCAAACTGCCATACTGAGTGTATTTTTTCATCTTACTGCTACTAAGAGTCTTAACTAATTAAATATCAAAATTATCTAAATGTTTGCAATATCACCTCATTCCTTTTCTCAGTTTCTTCATTTTTCTTTATTCATTGTATTTGTCTGTGATAATTGTAGTGATATTTGTGATTAGTTGAAAAATTTAATCCAGTTCCCTTTATTTATGTAAATTCTATATATTTAAGATTTGGAAACTTAATTTTTTCAAGTTTTCTGGTTTGGAAGGGATGGTATGTAATGAATACTGGATGTGGTTTGTTGTATCTAGGCAGGTTGCTTGCCTTGCAAAAGTAGTAGGACTAGTAAGTTTGATGTACTATTTTTGTACAACGTAGTTATACTGAAAAATATTCATACTGATATTCTAGTCCTAAAACATGAACTGTCCAATGGTGTAGATTTTTTAAGGTTTGTATTGCCTCTTCCAAAATGTTCTTGTACTGTATGTATATTTTAATGAAGCACAGTCCTTTATGGCATTTACTGAAGGATGACTGGAAAATTTTTGAGTAATAATGTATGTGCAGTATAGGTATATATTTTTTAATGCTTACAGAATGTCAGCATAAGATTAGAAAAATTATTTGACATAACTCAAGATATTTACAAAGATTTTTTGTACTCTGTTTTGAACATAATTCCTCGTAAGAAAAGAATACTTTAAGAGACATTTAGGCGTTCAGGCACAAAATACACAGGGCAGAGGGGAGAAAAGTTGTCTCCCAATGAACGTACATGTAATAACTGAAAAAAAATGTTCATGATAGTGATTATGATGTACGTACTTATATCAGATGTGAATAGTCAAAAGAACTGAAAATAATACAGTTGACATTAAGAGATAGGAATTTTAAAATAAGTCAATAGCTAACCTTAATTGTTTCTGAGATATCACACTATGCCCATAAAAGACACTAAATGTATTTTCTCCTACATATTCCAGTTATGATTCTTAATATAGAAACCTGTATGTACTGCAAATATCCTATGCAGGAAATACCACATGGTTACAGTTTATATAACTAGAGGTGGTGTTTTCTTTCAGGCCTATTAAGGTAAGTATATATTCATTTATAATTTTGAAATCTATAATATATTCAAAGACAGAGGTTTCCAGCTGTGATTATGTAAAAATTATATCCAATTTTAAGACTCGGTTCTGCCGTACATTTTCAGTTGCACAGTTATAATAAACATATTCCATTCCAGGTGTGTATTAGAGTATTTCCAAATTATCAGATATAGTAATTGCTATGTCATGCCTTACCATGTTAAAATATATGTTAATATATTGT

>chr9:+:13743169-13747475

AATCTGCAACCAGTCTCTTTCGTCAATGATAGTTGTATATTTTATCCTGAAAAAGGATTTGAACCAAAACTTACATACAGCACTCTCCCTTTCATACAACATTAACAGGGCTGGTGTGAATATTTTACTTTTAACTAAATATTATCCAGTCTGTACTGTTTATAGTATGTAATGCTTAGAAATCTGCTGTCAGTGTATGATTCAAAACGTTTAACCCTATGAGCTAGGTAAACCTTCCTTTCTTAAGAAGCAAGTGGAGTTAATAGACAGGTCAGCCTTTGGCGTTAGTGAAACACTTTTTCTCTGTTTCCCTGTTTTATTATGCCATTTTAAGCATGCATGTCTTTCATGGTATGCTGTAGTTTTTTTTTTTTTTTTTTTTTTTTTAAATATTCTTCCAAATAAAGCATTAGTTTTATTGGAACTCTTCATGATCACTGTGTAGTTGGGTGTACTGTGGACATGATTTATTTAGTGGGTTGTATTTCTTATATTCTTTTTGTTGGTTTGCGTATTTTATAGTGTGCCCGAGAAAAAAATACTGATATAGGTAATGATAGTCCTTTACCTTGTGATACAGTATTTATTGTAGCCAATCCAAAGAAAATGTGTAGCACTGTAGTCTGTATAGCACTGTTGTCTGTAAAGCTCTACCCCAGTCTTTTTTTCCTTTCTAAAGAAAGAAAAATTAAGAGGATGATTTTTCTTGACATCTAGACAAAGGCATGGATTACTATTTTTTTCTATTTATGCCTTTTATTTTTGGTTGTTAGTAAGGAAAAGACTTGATTGATGTAGAACTTGAATAGATAGTTTCATCAGAGAGAAAGTGATATGTACTCTTAATCACTAGGCAGGATGTGTGCATATGGGTAGCAACAGTAGTACTTAATAGGCCCGTACCTAAACAACAAACTGTACCAAACCTTTCAGAGCAAGGGGGAATTAAAACTGAACAAAAGCACATATATGATAAGGGAATGTTAGCGTAAATTGATGAGCTACAGAGAGGAAAAAAGAAAATTAGCTTTAAGATTATGGATCAGCAGAAATGGACACCCATGAACATGAAACAAGGTGCTCTTTAGTTTTTATGATATAATGAGTATCATTCTTTGTAAAATTGAGGCACTCTGGCTTGTCTCTGAATAGCCATCTTGATAGGAAGTACGGGAATTTTGTTGAAAGTAAACCCAAGTCATTTCAGTTATTACTTGTGGTTGCTGTCCTACAAGATTCATCACACACCCACTCAGTGTGTCATCTGTTTTCAGGGATGAATGAATTTTTTCTCCACTGGCAAAGCCCACTATAAAGTATATTGCTTTGCTGTGATGCATAATTTATTTTTCTAGTGGTGGCATTTCCAATTGCTATGTATATGGTGCCAATTGAAGGCTGGTTGCATGGCTTTGAAATGTATAAAACTGCATTCAGTGTTTTCATAGTAAAACAGTATTTTGACAAAATGTGGTTGAGACCAGTATTACTGATTTTTACTGGTTGCATTATTTTGTGAAATAAACTACTGCTCATGACTGGATTTTCATTATTGGTTAGATTGGCTGTTTTACAATTTCTTTTGCCATTATTTAGATAGTTTCTTTATAACCTTATGCTTGTGTGAATCAGAAGAGAGAGAATGCCGAGTTGATAGTTTGATATTTTTGTGTATTCTCTCTTTCAAGTTGACAGGGGACAAAAAGAGGGAATTAGCTTTTATTTGCTTTTTTTTAAGAAGTAAAGGTGGTGCCATTGTACTTGTCCTAATAAGAATTAGTATTGTAGTAGTAATGTTTGTTTTGGAAGCGGAAGTAAGGAAATATCTTTTGGTTTCATAAATTTGACACCTTAAAAGTGAAACTTGATTTTGTGGTTTGGAGAAGCAGCCACTTACGACCTGACACCTATAGGCCAGCACTCACATGTAATTATAGTTCAGGTATCTTACTTTTATGGCAGGACATTTATTCATTTATTATGAGGGAGGTTCAGGACTATTTAGAAATTGCAGTATTGAAATGATTTTACAGGGAAGTACTGAGGAAAGTGTTGCATTACCTTTCATTCATTTTTTGGTGAAATCTCAATGGGCAATTTTGTAAATACTGTACACACCTTATTACAATGGGATTTCACAAAGAGGCACATATAACATTTTTATTAGGAATAGATTAGCCTTTCTGTTCCTGGTTGGTGTTTTCGTAGATTTATCTCTCAGTCATGTATTATTTTTATTGATTTGGTAGCTGATTTTGTTCACTTGAAAATATGCTGACACTATTCTATTTATGTGCAAGAGATGGATTTTCCGACTATCCTTAGGCTTCTGTTACAGAAACCCCTCTTTGACTGTAACCCTTGTATTTTATTTTTGTGTTTGTTTTAACAAGTTTTCCATGCAAACTATTAGTTATACATTCCTGAAACAATTAGGTTTCCATTTTATATAGTTTGTTGGAAAAACTTTTCTCTTATATTTGGGTATACGATTTCTAACGTTAAAAAGTATGTCATTCCAACAGGAAAATGGAGCAGAATACTTGTTGTAGAGTATTTTGTTTGTTCATTATGACCCAGACTTAAATGTTGTTTGATATTGTATAGTTATGAGATCATATGTTGTAGCAGAAGCCTACTGGATAGGTTGTTAGTGATTAATGTACTATTGGAGGTCTTTTCATAATTTTTCGTGTTGAAAAATCAACAACTTCAGCACTGTACTGAGAATTGTGCTAAGCAACTTTTGAAAGTTTGATCTTGCTGCGAAGCATTCTTGAGTTTTTCAGAAAGTTGTTTGCAGGTAGACTGTGTTTCTTATCTCAGTCTTGATAATATCTAGTTCCTTATCTTTGTGTTTTACTTCTAAAGTAGGATTTGTTGGTACATCTTTACTGTATCTTTATAATTTTTGTTGTCTTTTATCTTTGCCATGTAGCTCAGGAGACTTCCATTGTGTTTTGATTTGGAGATTAGCATTAAGAAAAATTGGATAAGTACATTGACACCATCTTTTCATATTGTCTCAAATAAGAACATTTTTCTTCTATCTTTCCTGTCATAGTATTAATATTTGGAACATGCCTTTTTCATTTTGAAAGAAGTACTTTTTGAATAAAGTTCAACCCACAAAAAGTTCCAAAAACTCATAACCTATGGGTTAACTGTCATACACAACTTTGTTTCTTCAAACTGCCTATTTTTTTTTCATTTAATTACAAAATAATTCAGCAATACTTGAAGATGTTTTGTAATTGTCATGCTTGTGCAGGTTTCAGATTTTTGTGACCTTACACATGTTCAGGTTTCACAATTCATTATGTAATTTGTTCTGTCTCGATTTTCTTGAAAGAAACTTTGGAGATGGAAGTAACACAAGGCTGGAGAATTATGCAGGCTTATTTTGTTGATTGCAATACTATTCATTTTTTCTCCATCTTTGTTAAGTACTTGGTATTTTTATTTGTTTTTTATTCTAGTCACATACATTTGAAAGCTAATAGCATTCATTCTGGAAAGGATGATGTGATTTGTAGATGAATTCAGAATGTATGCTAGTTTATGAAAATAGTGACAAGATAAATTTTTTTCAAGTAGGTAGTGTCAAATTCTGTTGAAGATGTCCTTAGCTATAACAACATTCTGGGCAATCTTAGTGAATGGAAGTTATCAGGATACACTAAATAACTGGTTCAGAAAGATGAAAAACTAATGATTTTATTTTATTTTATTTCATTTTTAGATGCTAAAACTTGTCAAGGACAAAGTGTATTCTTGTTTTGCAGTAGCAAATTTTCTTTTACCATGAATGGCTAATTGAGAGTATGTTGTAACCCCTAGGAAATTTGACATTAGCAGCCTCCTGACTTTTTCTTGTGTTTTTGGTAGACAGTACTTGTATTGGTCAGGTGAATGAGGTCCTTGTGAAAAGGAGACAGCTGTGATATTTTAGAGATTTATTTGAAGTTAAATGACCCTGTCCAAAAATGGCTGATGGAAGATACTAAATTAACTTTTGTCTATGTGACAGGGGGTATTGTGGAAATTAAGAGTATTCTAGGTGTTTATTGCTAAATTTCATAGTTGGTATTTCTGTTGAGATGTACTACTGTTTGTTTTTTTCTGACATTTATGAAGTTGCTGTATGATTTTGTAAAGTACACATGTATACATGATCACCAGCCCATGTGTTTATTTTTTTTTATTTTTAATGAAGTTGCACAATAAAGTGTCAGCTATCACCTGTTGATGGACATGGTTGTGGATATATAATGCGAACACTTATATTTAAATATAAAAGATGAGTC

>chr9:+:13745665-13747475

TAACGTTAAAAAGTATGTCATTCCAACAGGAAAATGGAGCAGAATACTTGTTGTAGAGTATTTTGTTTGTTCATTATGACCCAGACTTAAATGTTGTTTGATATTGTATAGTTATGAGATCATATGTTGTAGCAGAAGCCTACTGGATAGGTTGTTAGTGATTAATGTACTATTGGAGGTCTTTTCATAATTTTTCGTGTTGAAAAATCAACAACTTCAGCACTGTACTGAGAATTGTGCTAAGCAACTTTTGAAAGTTTGATCTTGCTGCGAAGCATTCTTGAGTTTTTCAGAAAGTTGTTTGCAGGTAGACTGTGTTTCTTATCTCAGTCTTGATAATATCTAGTTCCTTATCTTTGTGTTTTACTTCTAAAGTAGGATTTGTTGGTACATCTTTACTGTATCTTTATAATTTTTGTTGTCTTTTATCTTTGCCATGTAGCTCAGGAGACTTCCATTGTGTTTTGATTTGGAGATTAGCATTAAGAAAAATTGGATAAGTACATTGACACCATCTTTTCATATTGTCTCAAATAAGAACATTTTTCTTCTATCTTTCCTGTCATAGTATTAATATTTGGAACATGCCTTTTTCATTTTGAAAGAAGTACTTTTTGAATAAAGTTCAACCCACAAAAAGTTCCAAAAACTCATAACCTATGGGTTAACTGTCATACACAACTTTGTTTCTTCAAACTGCCTATTTTTTTTTCATTTAATTACAAAATAATTCAGCAATACTTGAAGATGTTTTGTAATTGTCATGCTTGTGCAGGTTTCAGATTTTTGTGACCTTACACATGTTCAGGTTTCACAATTCATTATGTAATTTGTTCTGTCTCGATTTTCTTGAAAGAAACTTTGGAGATGGAAGTAACACAAGGCTGGAGAATTATGCAGGCTTATTTTGTTGATTGCAATACTATTCATTTTTTCTCCATCTTTGTTAAGTACTTGGTATTTTTATTTGTTTTTTATTCTAGTCACATACATTTGAAAGCTAATAGCATTCATTCTGGAAAGGATGATGTGATTTGTAGATGAATTCAGAATGTATGCTAGTTTATGAAAATAGTGACAAGATAAATTTTTTTCAAGTAGGTAGTGTCAAATTCTGTTGAAGATGTCCTTAGCTATAACAACATTCTGGGCAATCTTAGTGAATGGAAGTTATCAGGATACACTAAATAACTGGTTCAGAAAGATGAAAAACTAATGATTTTATTTTATTTTATTTCATTTTTAGATGCTAAAACTTGTCAAGGACAAAGTGTATTCTTGTTTTGCAGTAGCAAATTTTCTTTTACCATGAATGGCTAATTGAGAGTATGTTGTAACCCCTAGGAAATTTGACATTAGCAGCCTCCTGACTTTTTCTTGTGTTTTTGGTAGACAGTACTTGTATTGGTCAGGTGAATGAGGTCCTTGTGAAAAGGAGACAGCTGTGATATTTTAGAGATTTATTTGAAGTTAAATGACCCTGTCCAAAAATGGCTGATGGAAGATACTAAATTAACTTTTGTCTATGTGACAGGGGGTATTGTGGAAATTAAGAGTATTCTAGGTGTTTATTGCTAAATTTCATAGTTGGTATTTCTGTTGAGATGTACTACTGTTTGTTTTTTTCTGACATTTATGAAGTTGCTGTATGATTTTGTAAAGTACACATGTATACATGATCACCAGCCCATGTGTTTATTTTTTTTTATTTTTAATGAAGTTGCACAATAAAGTGTCAGCTATCACCTGTTGATGGACATGGTTGTGGATATATAATGCGAACACTTATATTTAAATATAAAAGATGAGTC

>chr16:+:63992011-63992966

GACGACGCTCTTGTAAGCAGTTCGGCCATCTTCTTGCGTTTCGTCATCACAGGGAAATGTGCGCATGCGTACAACACCACCGCAATCGGAGTGTCTTTGTGCAGTGTACAATCATTCTGTGGGGTACATCCATCCACAGACATGTATAAATGTAATATCATATTCCACTTAGAAATACGAAAAGACAGAGAGTTATATTTAGAAAGGAAATATAAAAAAAATAATGTTGATAATCATAAATAGACAATATTGAAACGATCGTGAGCCACCCATTACATGCTCATTTTTTTGTCCTGTCGGGAATGCGTGCCGTTTCGTGTGTATAAGTGTGCGCGTGATTGCGTAGTGTTCCAGGAGATCCCATGGATCATCTCTATTCCTACTCTCATCCACGCCGGGCCAAGAGGGGCTCCTACGGACGCCGCTACCTGTGTTGAGCAAGTAGACCTGAAGTGACCACTGAATGATCCGCTGTTAGTCCGCCCCGCCCCCCCTGTGTAAACGCCATCTCTCGGAGGAAGGCGGGGGCTAACGAGCCATTGAACAAAAGCTGAAAAAAAAAGCGACCGAGCCAAAGGCAGCGCCCCGCCACTCCCACCTTGCCACCGGCGGGCGCCCTGGACTCGGAACATTAGCCAGAAGAAGAAGAAACATCTAAAACGAAAAACCATCCGACTTTTCAATATTTGGTAGCGTGTCGGCCCATAGGTTCCCGTCTGGCCCAATAGTGCCTCTGAGCCAAGCCACCTTTTCTCTTTTTTTTCTTTTTTTTCTTAATCTTATTTTTATGCTAAAGGAGGCTACCAGGGGGAGTGTCACACAGTCAGCCAGCCAGTCGGAAAGAAGTCGACCAGTCAGTCAGCAAGGCCCCAAGTCAGTCAGTCAGTCAGTCCGCTTGAATGGCATCGTTATCAGCTGGTGTCTGTTGGCACATTGGTTAAGTTAACTATCGTCTC

>chr9:-:90798711-90798738

AGTGTGAACTGTGCCACCACAGTGAAAG

>chr9:-:90796837-90797219

TGTTTTTTGTGTTTGATCGTTGACGTTTGAGAGCCTCGTCTTAAACAGCAACGTCAGTGCGATGCCAAACCTCTGATCAGAAAGAGAATAAAAATTTGGGCAGATGTGAGACATCAGCGGGAAATCCTCCTGTCTCTTTGGCACAAGCAACAGGTGAATGGATTCTTCGAGGGAAGAAGAACCTGGCTCACGTTCCGCCCCCTCCTTCTAAAGAGGTGCCTCCTCCTGGGATGAACTCTTATCTGTATCGTCTTCTGAGACGGGACTGATGACGGATGTCTGTCAAATGATGCTGTGAGAGACTCGATTCGTTCGGGGTTTGGCTTCCTGTGGGGAAGTAATGCAGACAGCGAGTGCTGGGAAGCGGCAGCAGCTCTTTGATG

>chr9:-:90652471-90652511

ATTTGTCGTGAACAGCGCTACCTTATTTGTCGCAGCCAGTG

>chr9:-:90560380-90560505

GATTACTGAAGCCGATCTGGAAACCTAAGAAGATTAACGAGGCTTAAAAAATAAAATAGGTTAATAAGATAACTCCATCGGATTGTAATTACCTCAGGACATTTCATCAGACAGCCATATGGGCAG

>chr9:-:90541182-90541372

GTGCTCCGCTCCCGTATGCGTGTGGATTACGACTTCGGCACTCTTCTAGTCAAAAGATTGCCCGGCCTTGACGATGATGCGTCTGTTGTTAAAGATGAACTGACTGGTCGGTGGTAGGTCAGAGGTTAAACAGTCAAATAGAAGACTTCGGGAGCAACAAAATTGCATCGGTGGAGGTCTTTAATGAACAG

>chr9:-:90530158-90530289

GTACGCGTGAGGCTGTCATGGAATACTAAATTGGACCAGAATTCCAAAACGGGCGAGAAACCACACTTTAATTAGCAAGATAGATTCTTCTTCTTCCTCCCAGCTTTATCCCTATTCGGGGTTGGATAGCAG

>chr9:-:90514809-90515196

ATGTGGATTGTGGCCAAGGAGGTAGAGAGAGAGAAAGAGAGAGAGGGCCCCTCTGCCAAACCTCAAGTCATGCCATGCCCTCCCTCCATCATCAACAGAGAGATCTTGCCCTCACTGACCAACTGCCAAATATGAGACTCACCTGCCGCCAGTAACGCTGTCATTGAGATTCACAGTTCACAGTACACGAAGGGGATACAGTGTAGAGATTGGATATATAGGACGTAACAGATAACGCCCTGAAATATGGTCGAGCCTCAGAAGTTCGAGCTCTTTGCGCGTAGCGTCTGATACGGGAATTCGCTCTGATTTGGGGCTCTCTTTCGAGTACTGCTCAGTCCTTCGGTTGTGGTTTTTATTCCAGGTTTCAGGTTCGCTGAGTATCGTT

>chr22:-:32002759-32002846

GCCCACGTTGCCACAAACGACCTTCAGTCAACACACCACGTCTACACTCCTGCGTTGCCCCCACGCACGCCCACATCTAGTCAGCAAG

>chr22:-:31994623-31994858

TATGCACCCGCCTGTTGGAACAGGATACTCAGGAACTTACCAGTCTTCTGGAGGATACGGAAGTACTGGAGGTTATACAGGATCTGGTAGTGGATATACAGGAACTGGTGGAGGATACACGGGATCTGGTGGAGGATACACAGGATCCGGTAGTGGTTACACAGGATCAACCGGAGGATATACAGGAACAGGTGGAGGTTACACAGGATCTGGCACTGGGTACCCAGGATCAACCG

>chr22:-:31985029-31986932

GTTCCCGCAAGATGACTATCCGCGACTAAGCTTGTCGAAGGAAATAGTAGGAAGCTGGAGATGACAATAAAAGTGATTGATATAATATCAGTAGACACAGGCAGAGAATCTAAAGGTGTCATACGTTCATAAATCTTTCGGAAAGATCGCAGACTTAACATTATACACATTTTTATTCTTTACTCGAGGTACTATAACACGAACAAACATGCAGGCATCACACATACGATAAACAACATTTTGATGTCATCCGGAGGGATTTCGATATCAGTTGAGGTACACTGCATCTGAGAGCATCATGAAAATGCCAAGTCCGGCCGCCTCGTGTTTGTTCGTGATATGGTAAGTCGTCACCAACCGACACTCATAATACTCAATTTTCTTCAATATTTACTCTGTATGTGGGGACCACGCAGTTATAAAAACATTATACATACACATGCTAACTTTCATGGAATACACGTACACTGGCAACGTGAGTCTACAATTAATTTAATTCTAAACAAGATATGAAGATATGGCTTAAATTGAGTAGAACTGGGGAATGCAGCGCTTGTAGAAAAAACTACTATGATTTCCAAAAAAACAAAAAATGTGTGACATAATTCGAAGTTATTTACACTGTTTAGGGCAATGATGCAGATATAGATAAAAACTGAATTGTAAGCAATTTTTGCTTATACAGTAGGTAAGCTATCTATACACGTAAAGTTTATTTGGACATCTTGTTCCTTCTTAGTCGCGATCATTAGATAAGTAATCATTTGGAAATAGTAGTTGGCCTTTCTTACACTACACACACAAACATATACATAGGGCTTCACAGTGGAGAGGTTATAAAGTGCAGAGAGAGAGAGAGAGAGAGAGAGAGAGAGACGTTATATGAAATGATAAAATGGATTTAACTTACTTTAATGAACCTACACAACACAGCCCCAGGGGGGACTTAAGCACACTTCAGAGAATGGAAAATGCCCTCAGCCCTAATAAGCAACGCAATGAAATAAACACCTATATGAGTGATCTATCAGCATTCATTGATACTCTACCATAAATGGGTAAACTGGCCTTTATGGCAAATGGTCGATCACAAGAACGCATGATAAACACAGTTTATGAAATGTAAACTCACTTATAACCTCCCACGATGATTACACACAAAACACCGATACGTAACAATATCAAAGCCCAACCAAATCGGATTAAAACGTTGACGACCAAATGCTTGTTTATTGGACATGGGCAGTCTCGGTTCGTTTTCTTTCAAGCATAAGCCTTCTTCATAGAAAATGGTCACATTACTTAGATCGTGAACGTCGCTTTGAAGCCCCAAACTATTATAGGAAAAGGAAGTAGATAACCAAATAAAAAACATATATTTTAAGAGAATAAAGCTCGGGGATATGGCTATGGGGATGTCATCGCTAACGTGCTACATTGGAAGTAAAAAACGAAAGGAATAAATAGCTTAAGGGAAAATAAAAACAGGTTATTTGAGCCTGCTCACTATGAATGTTTTTTACAGAGTAACTTTTATTATTATAGGTCTTGTTGTTAATTTAATATCCGTACCTCCCCACGGAGCCCCCCATATCTCTGACCAGAAATTAGATTATGCCTTTTTGACAATCCTGGCCATATTGATATTATTTCATGTAATGATGTTCATTTTGTGTTGCCTCTGTGGTTGTGGGTCGCTAGAGTTGTAGCTTTTATAATAGTGAAGCTGGAGATAAATATTATGGTGTCGATGTTGATCTTTCTCGTTGTTTATTAAACCGTTAGCCTGCTTAAGTTTACCCTGGCCTTTTCCGCTCTTTGCCTCTTAGAGTGAATTATGTAAATTAGGTGAAAAATAGAATCAATGTATCAATAGGTATGCAATAAATACCCTAAGCAATGTG

>chr22:-:32002807-32002846

GCCCACGTTGCCACAAACGACCTTCAGTCAACACACCACG

>chr22:-:31985029-31986452

TCTACAATTAATTTAATTCTAAACAAGATATGAAGATATGGCTTAAATTGAGTAGAACTGGGGAATGCAGCGCTTGTAGAAAAAACTACTATGATTTCCAAAAAAACAAAAAATGTGTGACATAATTCGAAGTTATTTACACTGTTTAGGGCAATGATGCAGATATAGATAAAAACTGAATTGTAAGCAATTTTTGCTTATACAGTAGGTAAGCTATCTATACACGTAAAGTTTATTTGGACATCTTGTTCCTTCTTAGTCGCGATCATTAGATAAGTAATCATTTGGAAATAGTAGTTGGCCTTTCTTACACTACACACACAAACATATACATAGGGCTTCACAGTGGAGAGGTTATAAAGTGCAGAGAGAGAGAGAGAGAGAGAGAGAGAGAGACGTTATATGAAATGATAAAATGGATTTAACTTACTTTAATGAACCTACACAACACAGCCCCAGGGGGGACTTAAGCACACTTCAGAGAATGGAAAATGCCCTCAGCCCTAATAAGCAACGCAATGAAATAAACACCTATATGAGTGATCTATCAGCATTCATTGATACTCTACCATAAATGGGTAAACTGGCCTTTATGGCAAATGGTCGATCACAAGAACGCATGATAAACACAGTTTATGAAATGTAAACTCACTTATAACCTCCCACGATGATTACACACAAAACACCGATACGTAACAATATCAAAGCCCAACCAAATCGGATTAAAACGTTGACGACCAAATGCTTGTTTATTGGACATGGGCAGTCTCGGTTCGTTTTCTTTCAAGCATAAGCCTTCTTCATAGAAAATGGTCACATTACTTAGATCGTGAACGTCGCTTTGAAGCCCCAAACTATTATAGGAAAAGGAAGTAGATAACCAAATAAAAAACATATATTTTAAGAGAATAAAGCTCGGGGATATGGCTATGGGGATGTCATCGCTAACGTGCTACATTGGAAGTAAAAAACGAAAGGAATAAATAGCTTAAGGGAAAATAAAAACAGGTTATTTGAGCCTGCTCACTATGAATGTTTTTTACAGAGTAACTTTTATTATTATAGGTCTTGTTGTTAATTTAATATCCGTACCTCCCCACGGAGCCCCCCATATCTCTGACCAGAAATTAGATTATGCCTTTTTGACAATCCTGGCCATATTGATATTATTTCATGTAATGATGTTCATTTTGTGTTGCCTCTGTGGTTGTGGGTCGCTAGAGTTGTAGCTTTTATAATAGTGAAGCTGGAGATAAATATTATGGTGTCGATGTTGATCTTTCTCGTTGTTTATTAAACCGTTAGCCTGCTTAAGTTTACCCTGGCCTTTTCCGCTCTTTGCCTCTTAGAGTGAATTATGTAAATTAGGTGAAAAATAGAATCAATGTATCAATAGGTATGCAATAAATACCCTAAGCAATGTG

>chr22:-:31985030-31987053

ATCAATCGCGAAGTTCGGCCCAGGGAGACATTCACCTACAGCCAGACATTCGTGCCACGTCGCTCGGGTGACCGACGCCTGGTGGCAACATTCACTTCTCGCCAATTACATGACGTCACCGGTTCCCGCAAGATGACTATCCGCGACTAAGCTTGTCGAAGGAAATAGTAGGAAGCTGGAGATGACAATAAAAGTGATTGATATAATATCAGTAGACACAGGCAGAGAATCTAAAGGTGTCATACGTTCATAAATCTTTCGGAAAGATCGCAGACTTAACATTATACACATTTTTATTCTTTACTCGAGGTACTATAACACGAACAAACATGCAGGCATCACACATACGATAAACAACATTTTGATGTCATCCGGAGGGATTTCGATATCAGTTGAGGTACACTGCATCTGAGAGCATCATGAAAATGCCAAGTCCGGCCGCCTCGTGTTTGTTCGTGATATGGTAAGTCGTCACCAACCGACACTCATAATACTCAATTTTCTTCAATATTTACTCTGTATGTGGGGACCACGCAGTTATAAAAACATTATACATACACATGCTAACTTTCATGGAATACACGTACACTGGCAACGTGAGTCTACAATTAATTTAATTCTAAACAAGATATGAAGATATGGCTTAAATTGAGTAGAACTGGGGAATGCAGCGCTTGTAGAAAAAACTACTATGATTTCCAAAAAAACAAAAAATGTGTGACATAATTCGAAGTTATTTACACTGTTTAGGGCAATGATGCAGATATAGATAAAAACTGAATTGTAAGCAATTTTTGCTTATACAGTAGGTAAGCTATCTATACACGTAAAGTTTATTTGGACATCTTGTTCCTTCTTAGTCGCGATCATTAGATAAGTAATCATTTGGAAATAGTAGTTGGCCTTTCTTACACTACACACACAAACATATACATAGGGCTTCACAGTGGAGAGGTTATAAAGTGCAGAGAGAGAGAGAGAGAGAGAGAGAGAGAGACGTTATATGAAATGATAAAATGGATTTAACTTACTTTAATGAACCTACACAACACAGCCCCAGGGGGGACTTAAGCACACTTCAGAGAATGGAAAATGCCCTCAGCCCTAATAAGCAACGCAATGAAATAAACACCTATATGAGTGATCTATCAGCATTCATTGATACTCTACCATAAATGGGTAAACTGGCCTTTATGGCAAATGGTCGATCACAAGAACGCATGATAAACACAGTTTATGAAATGTAAACTCACTTATAACCTCCCACGATGATTACACACAAAACACCGATACGTAACAATATCAAAGCCCAACCAAATCGGATTAAAACGTTGACGACCAAATGCTTGTTTATTGGACATGGGCAGTCTCGGTTCGTTTTCTTTCAAGCATAAGCCTTCTTCATAGAAAATGGTCACATTACTTAGATCGTGAACGTCGCTTTGAAGCCCCAAACTATTATAGGAAAAGGAAGTAGATAACCAAATAAAAAACATATATTTTAAGAGAATAAAGCTCGGGGATATGGCTATGGGGATGTCATCGCTAACGTGCTACATTGGAAGTAAAAAACGAAAGGAATAAATAGCTTAAGGGAAAATAAAAACAGGTTATTTGAGCCTGCTCACTATGAATGTTTTTTACAGAGTAACTTTTATTATTATAGGTCTTGTTGTTAATTTAATATCCGTACCTCCCCACGGAGCCCCCCATATCTCTGACCAGAAATTAGATTATGCCTTTTTGACAATCCTGGCCATATTGATATTATTTCATGTAATGATGTTCATTTTGTGTTGCCTCTGTGGTTGTGGGTCGCTAGAGTTGTAGCTTTTATAATAGTGAAGCTGGAGATAAATATTATGGTGTCGATGTTGATCTTTCTCGTTGTTTATTAAACCGTTAGCCTGCTTAAGTTTACCCTGGCCTTTTCCGCTCTTTGCCTCTTAGAGTGAATTATGTAAATTAGGTGAAAAATAGAATCAATGTATCAATAGGTATGCAATAAATACCCTAAGCAATGT

>chr22:-:31985030-31986919

GACTATCCGCGACTAAGCTTGTCGAAGGAAATAGTAGGAAGCTGGAGATGACAATAAAAGTGATTGATATAATATCAGTAGACACAGGCAGAGAATCTAAAGGTGTCATACGTTCATAAATCTTTCGGAAAGATCGCAGACTTAACATTATACACATTTTTATTCTTTACTCGAGGTACTATAACACGAACAAACATGCAGGCATCACACATACGATAAACAACATTTTGATGTCATCCGGAGGGATTTCGATATCAGTTGAGGTACACTGCATCTGAGAGCATCATGAAAATGCCAAGTCCGGCCGCCTCGTGTTTGTTCGTGATATGGTAAGTCGTCACCAACCGACACTCATAATACTCAATTTTCTTCAATATTTACTCTGTATGTGGGGACCACGCAGTTATAAAAACATTATACATACACATGCTAACTTTCATGGAATACACGTACACTGGCAACGTGAGTCTACAATTAATTTAATTCTAAACAAGATATGAAGATATGGCTTAAATTGAGTAGAACTGGGGAATGCAGCGCTTGTAGAAAAAACTACTATGATTTCCAAAAAAACAAAAAATGTGTGACATAATTCGAAGTTATTTACACTGTTTAGGGCAATGATGCAGATATAGATAAAAACTGAATTGTAAGCAATTTTTGCTTATACAGTAGGTAAGCTATCTATACACGTAAAGTTTATTTGGACATCTTGTTCCTTCTTAGTCGCGATCATTAGATAAGTAATCATTTGGAAATAGTAGTTGGCCTTTCTTACACTACACACACAAACATATACATAGGGCTTCACAGTGGAGAGGTTATAAAGTGCAGAGAGAGAGAGAGAGAGAGAGAGAGAGAGACGTTATATGAAATGATAAAATGGATTTAACTTACTTTAATGAACCTACACAACACAGCCCCAGGGGGGACTTAAGCACACTTCAGAGAATGGAAAATGCCCTCAGCCCTAATAAGCAACGCAATGAAATAAACACCTATATGAGTGATCTATCAGCATTCATTGATACTCTACCATAAATGGGTAAACTGGCCTTTATGGCAAATGGTCGATCACAAGAACGCATGATAAACACAGTTTATGAAATGTAAACTCACTTATAACCTCCCACGATGATTACACACAAAACACCGATACGTAACAATATCAAAGCCCAACCAAATCGGATTAAAACGTTGACGACCAAATGCTTGTTTATTGGACATGGGCAGTCTCGGTTCGTTTTCTTTCAAGCATAAGCCTTCTTCATAGAAAATGGTCACATTACTTAGATCGTGAACGTCGCTTTGAAGCCCCAAACTATTATAGGAAAAGGAAGTAGATAACCAAATAAAAAACATATATTTTAAGAGAATAAAGCTCGGGGATATGGCTATGGGGATGTCATCGCTAACGTGCTACATTGGAAGTAAAAAACGAAAGGAATAAATAGCTTAAGGGAAAATAAAAACAGGTTATTTGAGCCTGCTCACTATGAATGTTTTTTACAGAGTAACTTTTATTATTATAGGTCTTGTTGTTAATTTAATATCCGTACCTCCCCACGGAGCCCCCCATATCTCTGACCAGAAATTAGATTATGCCTTTTTGACAATCCTGGCCATATTGATATTATTTCATGTAATGATGTTCATTTTGTGTTGCCTCTGTGGTTGTGGGTCGCTAGAGTTGTAGCTTTTATAATAGTGAAGCTGGAGATAAATATTATGGTGTCGATGTTGATCTTTCTCGTTGTTTATTAAACCGTTAGCCTGCTTAAGTTTACCCTGGCCTTTTCCGCTCTTTGCCTCTTAGAGTGAATTATGTAAATTAGGTGAAAAATAGAATCAATGTATCAATAGGTATGCAATAAATACCCTAAGCAATGT

>chr31:-:31510211-31513174

GGGACATGAGCGTCAGAGTGTGGGGGCTCTAAGCCTAATCTCCCAGATACTTTGTAGGATGTTGAAAACGTCTTCACAATTAAAAATGAATTCAGTTCTCCGGAGCAAGAAAAAAGTTGCATGGAGTTAAGTGACATTTATAGCTCATGGTATACATTCCACAACTTTGGGTATCTGTCTTTTGGCAATATTCAACAACACTTTCTTTTACATATAAGTTCTAAAAAATTGGATAAGATATTTCCCAGCCATCTTGGTACAGCTAGTCTTGGTTATTTCTTGCTGGGCTGACTTAGCAACTTACAGGATTGCAGCATGACTCTTATTTAGATGCTTCAAGGGCAAACATGGGCTCCTTCAATTTTTAAGTTTATTTCCCCTTCCATTCATTTGTGGAACTCTGTTCAACCGCCCTGTTACCTAGAGTCAATATCATACCAATCATTATATTAGACGCTTGTTTTGAACTGTTAAACCTAAATTTAAGCAAATAAATATTTCACAGCTACGTAAAATACAAAGTTTATTCCATGAGCAAGGATGTATCAAAAAGCTTTAAAAACTAAGGCTCATACATTATGAATAACCAAGATTTCCCCCACAGAGAACTCACTTGCTCACTTTACATACATTCCAGCACGGTACGTAAAAATAGACAGAAATATGCGTAAGCAGCTTAAGAACTGCTGCTGGAGGCAAACCTAGAAATTATAGTTCCACTCTTGATATTTACTGCCCCGGTTACAAATATGAAAATAAAATGATGTCACCTTTATTGTATTAGTGTAATCATTAGTCTATATTCAGTTATATAATGTAATTCATTCTATTGTACTTATAGCACTGCATTTCCTATTTATTACTACTATCTTTCATTGCTTGACTATCTGAAACCCTTCTTGTTGAAATTAACCATTAGGAAGACATTAAAAAGAAAACGCCCTTGCTGTGGTAATAAAATCCTACATAGTCAATAAAACCATTAGCTGATATTTTCCAGCATTACAAGGATTTTTTTTAAATGGCCTTTAGTTACAGACAAACAAATAAAATCAACAAAATGCAACTTCTTTACTGTAGAGGCCAAGTGGCCAAATTCTTAACCAATATCATATTATAGCTACAATTTTGGATTGAAATTATGTAATTTTAAACATACTCTTTGTTCCTTATGTGATTTTTGTAGATTGTTGTAATTATTGTTATTGTTTTTAATTATTCTGCTGAGTAGCATAGTGAAGATATGTTTAAGATAGTCATATCTGGATACACTTCACGGTAATACTGAGGAAAAAAAAGGAAGAAAGATTAAATTTGGGTGTTGTATAAACTACATTTGAGGAAAGCAAAATAAGTAATGACATACACGTTAGATATTTAGCTATCTTTTATAGACAAATGGCTCAAACAAAACCACGAGACTAGCTAAACATCAGGATAACTTGGCATTTGAGTTGGGGGCCTAGGCTCAATAAGCTAAGCAAGGGTTAACGTAAGTATACAGTAATTTGTCTTAAAACTGCGATACTTTTACATCGAAGTGCAAGTTGTTATCCCATTTCGGTGAATAAGGTTATTTGAAATGCTATTGGTTACAGAACTATTGCTTCACTGGAACAAAAGATTAGTGCAATTCCAGTAGATTTTTGGCAGAAAACTTAAGCGGATGAAGTCATATTTCCTTACAATGTTACTCTGCTGAACTGAGAAATTTAGAATAAGATAGGAAACACTTAATAGTAGAGTTACTTTAAGGTCTTGGTGGAGAATTTTGCTTAGCTGTGGGGGATAGTTAGGCAAAGCTGATCTACCTTCTGGGGTGCGCTGTTTTCCTCTGCAGGTCATTAGCTCTCTGATACTTACAAAAATAGAACAAAAACTAAAGACAGTCTTATGAATTGGGAAGAACTTCACGGATACCTAAAATGTAGGGAATAAATTATCTTAAAAAACGAATTCATTACAGTCACACTGTGACAGTTTTCTCTGGACTATTAATACAAAAGCATCAGGTTAGTTAAGGCTGGTAGTTGTGGAACAGTAACCCAGTAAATAACTTGTGCAAACAGCAGACAAGATGCTTCCTGGTACCTAGAGGTGGTCTTCCTTCCATGTAACTGTACCTGCTCTCTAGTGGTGGAAGTGAAGCATTGGTTAGAATTTTACGGACTTTTTTCTATTTCATGTACCCCTTCATGGGAATTGTATATGTTTTTAAGGGTTATTTGTGTGTTTTATATAGCTGGTTGTAGAGGATCAATAACTTATTAGATTATTTGAATGAAATGTTATTTTCCCCTCGTACTAGTAAGGTTTTTCCCATTTCCTCCCATTATCTGTACTGAATAATAAGGCTGGATGAAACGTGTATTTAAAATTTGGCTCTTACATGAGCGCATTGTTAAATTCTCGAAAGCAAATATCTACATGAGCAACGGGACACTTATCTCTCGCTGTTACGACACTTCAGTGCAGGTAAACTTGATAAAAAAATATTTTTACATTGATTATACTGATTCTAATAACGTAGAACTTATCCAGAACAAAAGGAGACCTAATACTTCAGCCTTATATTTATTAAACTCAAGGAACCGTGGGTCTTCTGCATCCCTTGAACCTAATGAGAGAAAGCAGCATTTTGGGAGACGCGGGAGAATGTAAGTCTCTCAATTGACATCCAGGTTTTTGCTTTGTAGCTCTCTATACTCTATATTAATAAAGTATCTTCAAAGCCCCTCTATAGCAATTTGCTTTCTTCATCTTTACTCGCTTCCTCTCAGTTAAACACTGTACTATATCTACCCCCTTAATCTTTCATAAGTATAAAGGAAAACCAAAAATATCTAGCCCATCTTTCGAAAGATGATGAAGCAGCTCACGCGGCACGTTGTGATCAAACCTGATAATACCTTTGTTGATTTATTTGTTTCACGGTAACTAAGGGTCCAAATAAAGAGTTGTTAG

>chr21:-:1963070-1963129

AGTCCCTTGGGCGAATTGACTGCACACGCGACTCGCGTCTCTCAAGCCCAATCTTTTTAG

>chr21:-:1955076-1955112

CAGTTCAGAAGAAGAAGAAGACACCATGGCGGACGAG

>chr21:-:1946655-1949676

AAGGCAAAAGCGGCCGAGGTACTTGCTAGACAACGAGCTTTGGAAGTATGTTCTGAGTGTCACCTACTAACTGCATGCGGGGCTTAATTAAAGTAATTTGCAATTAAGAGTCAAAAGATTTCAGTTCTTGAGCAACTGACTTTTGTATTGCTGAATATATAGATCAAAATCATTTGCAATAATTGTGAATTATAAGTACCACTGTATTCGTGAATATATGAAATAGTTGATAATAAGTCTGTGAGAAATAATCAGCCTTCTGCAGTCATTCACCTGAAGATATTCCGAAAGAGTTGTGGACGCGTCCATATAAGCCTCCACGTGAGAGTTCACGTGACGTCACGTAAAAAATGAGGTCATGTGACTCCATGTGAGGCCACGGCTGTCCCAAAAAGCGTCGCCCCGAGAGTTTCTTTGGTAACTGGCGCAGTGCCTATCTCTCACGAGGTAGGGCACTCGCCCACCACACCACACGCCCCTACCACAGCCCATCGAGTCCTCTCCCAGATCTAGCTCACCTTCCCCATACTATATCTCAAGACTGATCTCAGTGCAAGAGATGATAATTACTCAAACCAGGGCCCCGCCGCGAGCATAACAACGCGGCAGCCCTGGAGCGAGCACACTAACATCTGTTGACTCAAACGATCTGCATCAGATGATAAGAGCATCACCCGTTCACACTTGTGTTGTGACTATCATCCGCGAGAGATCTGGCGGACTGAGATCAACAGCAAACCCGCATGGGCAAAGAAGCTCATGTCGATGTGATCTTTAATACATCTCGAGATAGTCATGGGTCAAATCAAGATCAAGGGAGATGTACCCAATGGCTTGTGCGTCAGGGGCAACACAATCCCTTCCACATCCAGCGGCCGTCTAGGCGACGGGCGGCCCCGGGTCGGAAAAACCGACTAGGCCCCCCCCGCCCCCTTTCACTTCTAAAACTCACCCCCTCCCTCTTGATTTAAAAATGATGATGTCCCGTCGGGAGGAACTGAATGTTACCTTGTCCATAGATCGAACTGTCCTCGTTCATAAACGCAACATTTCTGTGTCTGTTGAAACGGCCTTGGATTTACCTATCACTTTCCTAATCAATTTTTTTTTTCTTTTATAACGATGGTCATTTAATATCTATAATTTCAGATGATTATTTTATTCTATGCATTTTTATTCCTCTTCACCATTAATTTCTTCCATAACAAGGATTTCATGTTCTGTTTATGAATTTCCTGATACATTATTTTCTAATTATTATTTCGTAACTAATCTCCCTCCCTAATCCAGGTGAAGTGGATTACGTTATCTCTGGACGAAAGGTTCAAAATAAATGTACCGCTTTCATTTCGGGCGTTTCTATCGGTCGCGTCTTCGGAACGGCGTCATTAGTAAGTACCGTGAGTTTATCTCACGGAAGAACAATTCCTCGACTTTTATGCTTGACACTTTTAGATTTCCGTTAGTCTTTCAGTTTAACGTCAAAACAGTTCCTTGAAATTTTACTCCCCCCACTCCCACAAACAAATTTTCATGGGTCACGGGTTCCCCTCTGCTTCAAAATAAATTTTTTGGTCCCAGGGCGTCGGACGCTCTCTTTTGATATTCGTTAGTCTTTCGGTTTAATGTCAAAACGGTTCCTCGAAATTTTATTTACGCCCCCTAAAATTTGGGTTTCATTGGTCCCCCTCTGGTTCATAGAACATTCTTTAACCCCTGGGCATCGTCGGTCAGCTCCTCGGCCCTCCGTCAGAGCGGGGGGCTGTGGCGAGGTTACCGAGGTGAAACTTCCCATTTAGCCCCCCGACCTCGCAGGATGGTGGGAGGTCAGGGCGCCATCGTATTTCTTGGGCGTGGGTGTTCATCTCGAACGTTCATTGACTAAGCTTCTATTTTTTTTCTTCTTCTTTTTGGCTGATGAAGCGATCGCCCTAGCTCTGACCTCCATATTCCTCAGCATTTTATATAATCAAAATTACCACATGAATTCTATCACTAAAAATCGTCTATACCTATTTTTCGTCATATAATTCTTGTCGCAACGAATAAAAGCAATAATCTTTTTTTCAGTAAAATTTTACACCGAAACAAATACAATTATTGTTTCGGTGACATTTGAATGTCCATATTTCATTTTATAAACGCAGCCCTTCGTGTTGACAGATATACAATATTTCTGAATTTTGAAACGTCAGATTTTATTTCTCGAAGGCACCCTTTAAGGGTGCATGGGGGCAACGTCAGTCATTTACGAATTGTATTACAAGGTTGTTTATGGTTCATTCAATACTTTATTAAAAAAATCTGACAAAGCGAGTTATATGGACGTTAGCTGTCGCTTGAGTCAGTCGTCTTTCTCAATTCACTGGGGAGATTTTCGGTGGCGCCCTGAAAGATACTTAACACAAAATAGATTAGAATTTATTTTTATTTTCATTTTTTTTTCTAAAGTCTCAAAAGCGGTTGGATCACGCGAGAATTGCAATTCATGTAGCAAGTGGTTGTTGATTACGTTATATTACTTTTATTTATTTTAATTGTTCTTTCTTTTATTTATTTTATTGTTTTTTTGGTTCTTCTTTTATTTATTTATTTTTTTTTTTGGCTGCACGATTCCATAATGATAACTTCTGCCTCTTCAAAAGCGTTTGTCTTGATTTTCGGCGACAGCTTACTTTGTGGCTTTCCACCCCCACTACTCCCCCTCCCTTTTTTTTGGCAACTCCACCCCCTCCCAAACTTTTGTTTGGCATAAGCTCGCTCTCCTTCCGTCTCTGTAATAACCTTTTATGCATCTACCAGAGGTACACAACCTGCCAACAGAGACCCCAAACCCAGTAAGTCAACTAATCTCTTGGTTTACAAATTTTAATTTTTCGTTTCTTCATTCATTAATCTTTTTCTTAAACCCCTCATATTATTCATTCTATGCCTCTTTGCAGTATTGCACCACAAAAGCTTAAGCTTCTTGGAAAGGCTTTTTAAAAGCTTATTGACCATTTTCAGTCTGATGCCGCAGATG

>chr16:-:53628069-53628145

AGAGTAGCTGAACTCCTCTCAGTGTTCAGTTCCCATTATTTGCTTCTCTCAGCTCTCAATAATACTACCATCTGCAG

>chr16:-:53622109-53623488

GATGTGGCGTCTGACAGCGGCTTTGCTTGTGGTGGCTCTAACTTCAGCTTCCCCACAGGGTTACAATTATCCAGCTCCTGACAATGGTTACTTACCTCCTTCACCTAGCAACCCCACCTGCCAAGTGGCTCCTATCACTTCCGTTCGCTACGACACACGTGTTCAGACATCTGTTAGGGTTCAAACAGTAAACCAGTTCAATACTCAATATGTTACCACAACACTTGTCAGGCAACAAGTTATCCCAACTACTTTGTACGAGACACGCGTTTTGACTCAGCCACAGTACCAAACCAGTATTGTACAGCAAACTTCTGTTGTTTACAATAATAGAGTTGTATCTCAGACTATTCCAAGTCCCCCGATCCAGTCTGTAGTATATGTCACAAGTACCAGAGTCGTACCTCGTGTTGACTATGTCACTCGCACTCAGATCCAGACTCAAGTTGTACCTGTTGAGGTTTTCAACACCCAATATCAAACAGTCAATCAGCCAGTAGTTAACTACCAAACCCAGTTTCGGCAAGAAACTAGAGTAGTTTCTATTCCTGGCCGTGATGTTATCCAAACTCGTGTACAAACTGTTGTCCAGACTTCCATTATCAGAAGCCAGGCACCAGGAAATACCCGTTTCCTCACTCAGACTCAGGTGCAACAACAGGTGCAGACCAGCGTTCTTAGAGGAAGAGATGAAATTGTAACTAGTTATGTTCAAAGACAACAAGTCGTCCCATACACAGCAGTCAGTACTAGATATGAAAATGCTTTTGCAACCCGTGAGCAGGTTGTCACTAGGACAAATATTGCAACACAAACCCAGGTCAGAACCCAGTATGTGCCACAGGAAGTTGTTAGCACACAAGTTGTGCCAAATACCATCTACACAACCCTCTTTGAAACAAGGGTTCAGCCATTCACACAGGTGCAGACTGTTATCAGGACCCAATACAATACTCCAGCACCTGTAGTCCAAACACGTGAGGTAACCAGTACTTCAATCTATCAGGCTCCTGGTAGAGACCAGATTGTGACCAGGGAAGTAGTACAGACTCAACAACAACAGCAGATCGTTTACCAGACTGTTAATCAGCCACAACAAATTGTTGTCACCAGAACTGTCACTGCTACTTGCTCTGGTTATAATTATGATGAACCTGCTATTCCTTTCAACATTGGCAAATAAACAAGCGTATTGTTATTTCCCTGGAACATCTAGGAAAACAATTCTTGATAAAACAACCATAAGGTCTATATAGATATAAAAAGAGGGCACTACATGAAGCGTTTTCATCTAGTTATAAACGCAGTATTTTAACATCTTTTTACTGTTAATAAAGTGAATGTATTTATGTAAATGGCAAATACACTTTAACACATGTT

>chr26:-:39200970-39201175

GAAGCTAGGAGTCTCTTTGAGTGAAGCCTAACACTTAGTTTCCAACTGCTGTATTGAGAGCAGCCTATCACATTCTGAGAATAAATGTGAAAGAATATGAGTGGTAACTTTTATAAGTGCTGTCTTACTGCTCACTAATAGTGCTTCAGTGCTTAGTGATGATTTAAGCAAGTCGTTTGTCCTGATCTACAGTTAACTTTCAAAAG

>chr26:-:39158728-39158787

GGACCCATGAGGCAACTCACATTACTGAGCAGCAGCCAACCTCTCTCTCCCACGACCAAG

>chr26:-:39106841-39108239

GATCACCAGGAGGACGGAAATCAAAGACGGGGAAATAAAGGAAGATGTCTATGAAGAATACTAAGGAGGATTTAGAGAAGGATATATTAAACAACTGTGTGCGTTTCCTCTCATTCCTCCTTAGTGCCTATCTGCCTTCCTGCCTACCCTTAATACTACTGCCATCTCCACCAACAATACTACACTAGTGTCATCGCCACAAACACCCCTTCTTTCCTTTTAATAATTCCATCAGGAATTTATTTGAAGGCCTCATATGTATTCATTTTCATCATCAATTCACTTTTAAATCCCAGTTAATTGATGGCTTTTTCCTTAATTATAGTAACATTTTTTTTGACCTAATTTTCAAGAACACTGTCCCTTGTAAGAGGTTTGTTAAGTGGTCAGTGTTGTAAAAAAAAAAAACCAGCAGCAGCAGCAGCAGCAGCTATTGACATATCGGTTGTCAAAGGATCTAGTCAGGACCGATAGCACTTGAGAGAAATCTAATTTTTTATGAGTGAGAAAATTTTTAAAAAATGTGACCTTTCTTGATAAAAAATATACAGTTTTATGTTACCTATAGAATTATGTTCAGTATAACCATGATATGGTTACTCTACTGTAATTACTATAAATGCCATTTTTAAAATGTCCATGAACGATCACATATTTGTAAAGGTGCACACACCCTCTTGAACCGTCAAGGTGTTCGTAGATTAAGTGACGTTCCCTCTGTGACATGGCGTGTTTCATCCTAGTATGACACTTATTATTACCATTAAGAATATGTATTGCTGAAAAACCTGTATTATAAAACTTTAGCCACGGGATGAAATAGGAGGCCAAGGTTCCAATAGAAGCTACTGTAATCATTTTATTATTCTGGCTCTTAGACAGGTAGAATGTTAATATTGTGGCTTATGAAACATCCAGTTATGGGCATGTTGAATAAGAATGCTATATTTCCACAAATTTTATGTATCAAATCTTTAAATTTGTTTTCCCATTTGTAAGCAACTTGAGGCAAGATTAAAGAATACAAGCCTGGGATAGGGCCTTGTGTGGTGTAAGAAACTAAAATAAAATACAGGATAATTAACAACAGTTTCCCAGCATTTAGGTAACAAATTGTATCTTTCATGTACTTGTTTCCCCACATGGTCATAAGTGGATGAAATAATATATATTGCCTTAGCACCTTATTCCTAGATTATATTCTCTGGCCAACAGTTGTTATTATCTTGATTCTTTTGAGTTGGTCTTGACTTAAACTGCTCTGTGAGGTGGGGAGAGGCCTTTCCTGCGATAATATATTTCTTAGGCCTACTCCTTGTCTCTTGGGTTTAACTCAAGACTTTGTAGCTCCAAATAATGTGGTCACTTATAAAGTTTTCAAAATAAAATGCTTAATGTG

>chr18:-:75818350-75818442

AGTGTGTAAGTTGAACCCGGTTAGGAAAGGTGTACGCGCCACTGCTCAGGTTGCCTCTCTTGGACTACAACTCGCATTTATTTACCTTCAAAA

>chr18:-:75796243-75796314

GTCCGAATGTCGGCCCCAACTACTGTTGTTGTAGCAGAAAATCTGCCACCTGGCACCTGCACTGTCTGTAGG

>chr18:-:75792582-75794604

AGAGGGAAGATCAAGGACAGTCCATCATGCTGCACATGGATCCTGTGTCTTTTGCTTCTCCCAGTGGGAATCTTCCAGGGATCATCGCTTTCTGCTGCTGCTGCCGGAAACCAAAGTGCACCCACTGTGGCTACACAACCTAGTCTTTAGGTTGAATGATTGAGGTGCTGTCATGGCCATTTTGTGAAATAGTAGTAGCTCCCTTACTATTTGTAATGCGTGCCTTGACCACTAACTTTTCAATTCTACTGTGTCTTGTTTGTTGTTTTCATGATTCTCATTGAAGTGTGATTTACCTTTTTGCACCATCAGTGGAAAGGGGCATACATGTCTAAATGATTTCAATTAGAACTAAATTTTTTTTTTTGCAATACAGTATCTCTAGACTTGATAATTTTCAAGAGAAAAAAGGTCCTATTATTGGTGCTTGCCTAGTACATAAATTCCCTCCTGCTGTAGAGGCCATACAATGATTTCCCTGTGCAAATTTAGAATATTTAATATGGAATTATTAAAGTTATCTCATAGAGTATAGTAGTACTGCCTTTTACACTTGAAGTCATTCCCCTAAGCCTTCATCATTAATTGAAGTAATTCCCCTAAGCCTTCATCATTAATTATAATAATTATTAACAGGAATAAAATTCATCTTGACCACGCATAACAATATTTGGGTCAGATGTATGGCAGAATGCTTTTTAAATAAAATAGAAAGGTACCTGTATTTCATGATAAAATTATGATTATATAGATTTTACTTCCAAATGGAGCAGAGAAATTATTGTATGGCCCTATTAGAAAAGTTTAGCATTAGCTACAAGACCAAAATGTACTGCATGAACCATGATGAAAAGAAATATAGATAAAGTAACCAAAGATATAGCAAACATGCCCCCCATATTTTATCCCCCCCCCTCTCAACTTATGAGTGTGTGTTCAGTTTTAGGTAGTTATATAGAAATTTTATTATTCTTTTGTACTCATTTAGATGAAGCTGCAATCTTTGTATTTAAGAGGTCATTGTCACATGAAATCTCACATTTTATAATGTATTTAAGATTTAAGACACCAAAGTGTTATAGGACATTGCATTTAGTTAATACAATGTATACCTTTAGCTATTACTGATTAGTTTTCCATTTAAGACTAGTTTTGTATGTAAAGCTATTTGAACTATTGTTAGTAATCTTGATACTGAAACAATTTACCCAAGCTGTAAAAAATGTATACATTTCAAAACAATATGAATTTCACATAATCCTTGTGAAGCTGCTTTTAATATTACATATAACAATTAGTACTGATGTATTGTCTCAGGTCTCCTTTTAAAGCCTTTGTACGCCAGCATAACTTACTTTTATGCAAGGAAAAAAATGAAAAACTCATGAAGTTTCTGAGAAAATTTGTATGATAATTCTTTCTGGCATTTTTAGGTGTTTTCAACCATTCATCTCAGCTGCCTTTTTTAGTTGATTACAGTACTATTACCTATTGGTGTATTTTTTCTATTTTTTTTTTCTCAATTCTTTAAAATTTATTAAGGAACACTCCCTTCAGAGTATCTGAACTCAAAATGACTGAAGTTGGCAAAATTTAATTTCAAGCTGGTATTACCAACAACTATTACTTCATTAGATATTTGGTTTTTTGTGTTGAATTTTCATACAATTGTTAGGTATTGAATAAAGAAACTATTACTAACATCCCACCGTTCGTCACTTGTTGTAGAAGGGTCTTCGTTACTGGAATTTGGTTGAAATAAACCATTCAGTAATAATCACAATTCTCGTGATGGGCGCAGAACTTGGTGCCCTTAAACTCCCAAAGCAGCATTTATGCTTGTGAGAGACTAGATTGTCTTAACAGGGTTAATTTTACTTGAATGTTACTTAACCAATTCTTGCTTGGTCAGACTAAATTAATTTTGTTTTATGGTAATGTTAATATTGCATTGGAGGTTTTATTCTTGTGACTCACTTGATATTTTTTTGTTTACTTTTTCTGCTAATAAAGCCTGGTATAC

>chr1:+:31270128-31270929

GGAGAAAGGGGATGGCGATGACATTGTCTTCGAGGACTTCGCCCGTCTGCGCCGCGGCATGAGCGTCGACCAGCCATAGATCCTCCTCAACTGGTCGTAAGGCAGATCACCTCCCCAGCTCTTGTTTCCCGCGCGCAGATGCATCTACTAGTCCCAAGCCTCTGCCGTCACCTCCTATTAACAAGTCCATCTCTTCCTCCTCCTCCCGCAAGCCCTTGGTCATTTCAACCAACCTGGAATTGGCAGACGAACCAATCGAACAACAGCTTTAATGCCGCCATCTCCTGTCTGTGACCATCCTAACAATTCACCTTTTGCGAACGAACGAAGTGTGGGATTTCAAACTTTTTTCCGAAACGACGTCGAAACTTCAGATGATTTTTTTCCTTCTTCTTCTTCTTCTTAGTCACTTACATCTTCTTGATTTGTCTTTTTTTTTCTTTATCTTTTTCCATATGGTCGTCGCTGCTGTTGTCGTTTCAGGAGGCGACTGCTCGGGTCTAGCAGCTGCCCGAACGCTATCACAGTGGTAAGCATAGACGAGGTTACTCCATAATAATATTTAGTTCAAAGAAGAATTTACTGTTAAACTAGAATCAGCTGTAACGCAGCAGCAGCGACGGGTTTTCTTATTTCCTCTGTAGTATTTAAGGCTTTGTGTTGATTTAAGTTATCTTTGTAAAGGATTTCTTCTACCGATCCTCAACAAACATCTTCGAGCAGTTGTCTTCGTGAGCTGTTGGTTCTAAATCGGCAGTGTGATATAATTGGCTGTTTTCTTTGCTTAAGTAAATTTAGTTAC

>chr31:+:8277087-8279674

AGTCCTCTATACATTTTAATTGTTTCCAAGAAACTAAACACAAAGTGTGCACACAAGAGAATATGAGATAGCGATGTAAAACAAACATGCTTTACAAGAAAGAAAAGTGCTCCTTACCACCCTAGAAATTCCTAACCTAAAAGTTCACAGATAACGAATAGTATAGGGTAGATAAAACAAGGCTTAAAAATGAACATTGTAATAGGATGGTACAGAGCCCAATTCTTTCAAAAATAATATTTGAAAGTGTAACAAACTAACAAGAATCTATTGTATTGGCTGTTCAGCTCAAGTGTAGTAATACAAAACTGGGTAGAATCTAAAATTTCTTATCTTATTCTAAAGCTCCACCCACCTTATCTTCTCGAAGCACCGTCCATGGTCGTGAACGATACGTGAATGCTCTTTGTAAACATATTTTGAAGACAAAATTATATGAACTCTCTACAATGTACTTATCCTTGAACACTATAAACATAACTCTGCTAAACAGTAGCAACTAAATGCCCCTAGTAATCCGCCACTTATATGGCCAGCTGAACAATGTAGTGAAATAAATTCTGCAAGCAACATTAGTTTTGATCATCAAAATAAAAAAGATAAAAACTTATATATATCAGTCCAACTCAAAAAAGAAAAAAAATACACTAAATAGCTTTTCTTTCTAGTTGACAAAAATGCAAAAAACAAAAAAACAAAAAGATCTACCAAATATACAGAATCCTAATCATCCAAATATTTCTGATTCTGTTATAATAATATATATTCTCACTGTTGAGCATCAAGTGTATTGACGATCCATACACTAATTTAAAATGGATACTACACTTGGTGATATCTATGTTATTAGTCCCATGAAAGTAAATTACCATAAGAAATGTGACAAACAAAATATAAGTTATTCTTGTCAATGCTAAGAAACGCAAAAGCCTGGTACATTACTTCCTATCACAAAACCCTTAATTGTTTTGCCATTGGCTACAATATTTTTTTGTTTTTGTTTACTGTATTTACAAATAGATTTCTTCTAAGGTTTTATAATTATATTTTACAGTTGCCAACTTTACAGCAATTTCAATGATGACATATTTCCCACTATCAAATATATTCAAACACAAGTGACAAAGAACTTATACCTTACTAAACAAAATCCCTGATCAATTTCCTACAAGCCTACACAATGCCATTGTTACTCAGATTATGAACATCAAAAAAAAAAATTAAAAACTACCACCACTGTTCTTCAAACACAATTCAATCTATGTTTTAATTTTAAAAACTTCCATGAGACTTACTTAATTTATTATGGCCACCTCTTTGACAGTTTAAAGATTCTGTTCTTTGCAGATTATGATAGCCCAAATATAAAAGTTCTCCATCTTCCTCAACTGTTCACCTTGCTGTAATAAATACAGGCTTAAGCCACTGACAATCACACATTAGTGGGGTAAATTTCACTTTGCATTGTCTTTTAAATGTTGCTTATCTGTATGAAGCAGAGCAATACTGATGATAGCCTCGCCTTTAGGGAAGTACACTGTAATCTAACAAAGAAACCCCATTTCACCTTTTTGCTTATGATGCATTCACAAATGTTTTCACTACTCTGAGTTGACTGCTGAAAACTGTACATTTGAATTAGTGAATGTTAATTTTCATGAATGAAATTTTAATATTATATCCTGTGAGGTTTTCCAAATACAAATCTTCTAAATTATACCATGAAAAAGTTTACTGATTCACAAAGTAACAATGATAACTTACAAAGTTATCATACGTAAGGAATGTTAATCTGGCTATATATAGACAAAAATATATACAAGTACATACATATATATATGCAAAATTGTGAAAAGAGAAACTACTATAGTATCATGTACGTGAATCAGGTATAAATTCTCCTTAATGCATTTCAACACTGGCAATGTCCTCATAAGGAAATTTGCTATCAAAAGATTTCTCTACAAAAATAAATCCTTAGAACAAAATTTAAGTCATGTCCCATAACTGGAAAAACAAGGTTTCCTCCTGGCCCTCCAATATAAGAAATGATGATTATTATTGAAGAATAAAACAGCGTCATTAACCACTCACAAGATATTAGCCTCTTATTCGTGCAGAACTATAAAAATTATCTGATGGTGCTTGACATTACAGAATTGGCAACCCCTTTCCTAGGAAAAACAATACCAATTCAAAATACAGGTAACAATAGGCAAGGGACTGTTATAAGGAGCCCTACAGATGTAAACATTACCAGTTAGAGCCTTGTCTCAAATCTTAATACCATATACAAGCAGCAATTCACCAAATTAAACATACTTGCCTGGATTACACTCGCTCACATCAATAAATTACTTCACACTCAACACTGACTTAAAAAAAAATTGCCTCCTAATCCAACCTACATAGTTTTTAAGCAGATCATCTATCTTAAACAATTTCACCTTTTCAAACCCATCAGTAGAAGAGTAAGTTGGAACAAAACAAGCTGGAAATCAAACTAATGATACATATTATATAAACGACCTCAAATGCGCTAAAGTTTATGCATAACCTCCTTTTACCTTTTATGATACTATCAGCAC

>chr16:+:21259361-21260924

GGCACAGGGATGAAGTCATTATGAGAGTATTTATTGTAACAGAATGTAACTGTTTTCAAGTCCATTATATGATAACTATTCTTATTGGGAAGAGTTCTCCACAATCATATAATTACATAAAGTCACAAAAGTATACATAATGGTTTTGAGTCACAAAGTATACTTTCTGATCTGTTAACTTATATTATTTAAGAAGGATTGACATTGCATGTATGCTATAACTGCAAACAATAGTAAGGTAGTTTTATTTCTTTTAGCACCAAAGATCTCTCTAACTAGACCAGAGATGTCAGACTTGCATATTACAATAGCAAGTAACATTTGGAAACCACTGCAGTGTTAGATGAAGGCCATTGTGTGTTGTGATCATTTAGTGTGTGTCTTGCTAGAGAATATGAAGGAGCCAGATCAAGGTCGTGCAGGCAATGTTTTAATTATTAGTTGAACTTTGATATGATGTTGAGATTCTGCTTTAGGGGATGCACTTACTTTGTGATTATAGTCATTACATTTTTCATTTGTTAGGTTTTATGTGGATGTATTTATTGAGGTTGACCTATGATGATGGTATATCTATATCCAGTGACTACTCAAGGGAATATGCTTTTTGTTGTCCTCCATTTTTTGAAATACATGGGTCTTTCCACTCCATACATTATTATTGGTTTTTTGTTTTGATTGATTGGAGTAGCTCTGAAATTTCAAGTACATTTCGGCAACCTTCAGAATATGGATACCAGGTATTTAGTCCCTTTACCATAATATTGTCAGGTGGTGTTTTGTATATTTCAGTATCCTAAAATGATCTCGATTTAGGTAGAATAGGATGAGCACAGGAATGGGTCAAAGTAATTCCAGACATTTTGTAGAGATAATCATTATATTGCCTAAAGGGTAGGGCATCTGAGACACTTTGTAAGGTTTAGTAGCTTAAGTAATTAATATTTTCTTAAGGAACATACATAAAACCTCCATATATCAACATATATAGCTTTATATGAAAGTTATTACTGATAAAGTGAGTTTTTCACTTTTGCTTATCTCTTACTTTTAGAATTCAAGAGTTTTTTCTCATGATGAAACTAATTTATGGTCTTCACAACTGTACTGTCTTCCCTTTGTGATATGATTTACCTTGCAGTTTAAATCTTATTTATTATACATATATATTCAACAATCTGTAAATAATTTTTCTCATATCCTCAAGTGATACCATATTTTCACTTTTCAGCTTATTACATCCATCCAGCTTATCTGTCTCCGAGTAAAGTGAGGTACTGTAATGGTAAATCACAAACTGGGATAAAGCTGAATGACTTTGAAGTAAACTTATTTTTCCTATTCACACTTGGTCATTTTAAAGTGCACTTGAGCATGGTATCTCGTATCCTCTTCCCATTTCATGTACATAATAAACTGTAGGTGACATCTAATCCTTTAAAGGTTGCTGTGCATGTTTAGAAGACTGTAAATTACAGTATTTTTCATATTTTTGTCTCTAAATGCTTGAAAAAAATTGCACTGATGAGGTTAAGGCCTCATAGAAAATTGTAAGTCTCTCTCT

>chr4:+:22504957-22505335

GGGTACCTCGTGGTGTAGTGTCAGCGCGCTCGGTTCACCACCGAGAGGTTCTGGGTTCGATTCCCGGACGAGGAGAAACGATTGGGCCCGTTTCGCACACCCGTCGCCCCTGTCTACCTAACAGTGAGTAGGTACCTGTTGTCAGTCGGGCTTGGTGGGCCTGCTTTCAGGGAATGGTTTCATGTGGTACACTAATCGCATGTTGAGAGTGAGAGAGTTCAGAAAGGGTGTTGAAGAGTAAAGAAAGATAAAAAAGGCAGTCTAAAAAAGCCCTCCGGACTGGTCACCCGGATGAAGGCGAAAGAAAGAAATAAAAGGCGAAGGTCCATGTCCCAGTTGCAAAGAAAAATGAAATGAAAAAAGTCGGTGTTGTCCTCCT

>chr4:+:22504957-22505296

GGGTACCTCGTGGTGTAGTGTCAGCGCGCTCGGTTCACCACCGAGAGGTTCTGGGTTCGATTCCCGGACGAGGAGAAACGATTGGGCCCGTTTCGCACACCCGTCGCCCCTGTCTACCTAACAGTGAGTAGGTACCTGTTGTCAGTCGGGCTTGGTGGGCCTGCTTTCAGGGAATGGTTTCATGTGGTACACTAATCGCATGTTGAGAGTGAGAGAGTTCAGAAAGGGTGTTGAAGAGTAAAGAAAGATAAAAAAGGCAGTCTAAAAAAGCCCTCCGGACTGGTCACCCGGATGAAGGCGAAAGAAAGAAATAAAAGGCGAAGGTCCATGTCCCAGTTGC

>chr16:+:19468322-19470734

GATGCTTGCACATTAGGATACAGTCTTGAGTCAAAACTTTGTTTTCAATGTTTTTGAAACTCGGCTGTGTATGGTTGCATTTGTTTGCGAATAACTTAAAATGCCTCAGTCAGTGTTATCCTGACTCATAAAATATAGTACAGTATTGATTAGTTTACAAGTTCTGTGGGTTGGTAAGTTACTGGATAATGGGCTAGAAGGCTTATCTTAGAATCTAATCATGCTTTCATCATTCCAAAATCATTATCAGTATGTTATTTAAAACTGTACAAATATAGGTGTTACACATAGTACTGTATTCGTTTTTAAGGCATCATAGATATATTTCTATCATTTATATAAAGATGAGCGTCAGTTGAAGATCTGTTTCATTTTATGTAGTTTTATGCAGAATTGTGATAAATGTTAATGGCTAAGAATAAATGCTGATAGTATAGTTTTGTATTTATCTTTTTATGAGAATGAATTTTAATGACTTTCCTGTTTAGAGCTATAGTTTATCAAAAATAGATTATGTATGTATAGATGGAAGTTTATATCAAAAGTCTATTAATAATGGTAGTCTAAGAATGATAAGTATATATTAATGCATTTTTATACCAAACTCTTAGGAGTTGCATTATTTAATATTGTCATGGAGAATGTTATCCAATCATTATTAATGAAAGCAAATTGAGACATCGTCAGGTTTTTTTGCAGTCTGATGATTGTAATTCATTTCCATTGCTCTACGATGAACACAACTGTCTTGTATTAAGAGTGAGCAAAGAAGGCATGATCATTTATATTTTCATGAGCTCTACATTGCTTTAATAATAACTGGCTTCAGCAGTTTATTACATGAGGTCTATTAACTCCTCAGTAATCTCATATTAAATACTAGTAGCCTCTACTGTAATTGCTGATTTGTAAATGTAGGTGCCGTTACCTACTTAAAATTCATACAGCATTTTTTTTTCTTTGACAGCAGAATGTTTCTGTATGCATGTTAGAACTACCTTTGTGCTTAATATTTTTTTTCTTTTTTTGTTGTGTCAAAAATTATTTTCATGTTGCTTTGTGACAATTAACATGAGATAAATCTTTCAAAATATTCCATGTTTTCTTTCTTTTGGGATTATTTCAAGCACAAAATACTGAAACAATAATGCTAAATTCCCTGGCTAATATTTTTCAATTAAATTTTTGTATAATCTTAAATGCCACTATCACATCAGTTATAGAGGTGGAATTAATTTTTCATATTTATGAAAAGATCGTTTGCATATCATCAGTATTATTTTTGCATCAAGTATAGGTGGCCATTTTATATAGTTTTGCATGGTCATTGAGAATTCAGATTAAATAAAGAAAAGAATGTCGAGAAATTTTACTTCAGTACTGTTAATTGTGACCAAGCTAATAATTATGAAATTTGTTGGTTTTGCTCTCTTTCCCTTCCTCCTCTACTTGTGACAAAAACTAATGAGTGTTGTATGAAACATAGTGATGACATTTTGTGATCAAGTGGCCACAATCATAATGTAGTTGTTATAGTTAAAATAGTTACAGTATTAAAGGTGTGTTTGATTGTTAAGTTTACTTTTGCTAAAAATATTATGGGGTAATCTACAATTTAATTAAGCTCATGACTACCCTGGCATTAACTGAGAGTGTAAAAGGTTTCATATCATCCCCATTTTGGTTCTGGTTTCTCAGAGATTTTTTTAAACAAAATTGTTATGGTAGATATTTTTTCGGCTTGAGTTGAGAACTTGATACCAAGTATTAATCATGTATATGGAAGCCCAATATTTTATGTCTCGAGAAGTAATCATTTGCAAGGCATAGAACACTATGTACCTTCTGGAAAAAAAATTATGTAACTATGAATTTAAACATCTTTTGCTGGTTTTTTCATGTAATATACATATATGTATGCTGCTATTATAGATCTGAAATCCATGATTTCCAATTTTTCTTGGTATTCACTGGGTAAGGATGTGATGCAATGCCTTTGCAAGTAACACTTGTTGGGTCTGTTTGGGTGGGTGATGGAAGGGTTGTGATTTGCACTTTTTTCTTATTTTGGTATTCTTGACAAAATTTGATATCCCTTCAACAGCTCGACCTGACCTACATATCATTCCAAGGGGTTTTGATGCTGAGTGGATGACTGCTATTTGTATATACTATGCATAATTTTTTTCACTTGACTCTTGACTGAATGCATTTCTCTTATATCTCAGCATCTAATTTAGGTCTGATTATAGTATGGTCACACTAATGTTAGTTATGGAAATACTCTTTGTTCTGAACTTCTTACTACAAAATTATATAAATCTTTCTCCAGATATTACCAGTTTTATGGCATAGTTCTACATACTCACTAACATTAGCACGTAGTCTTTGTAAATAAAATGTATATATCTGG

>chr21:-:51280016-51280057

ATAGAGAGAATGGGATTATCCCAGAAGAAAACGAAAAACCAG

>chr21:-:51270671-51272896

AGTCTGGTGAAGGAGACCAGTTTGAAATGGATCTTTAAATGCCAGTGTTACTGGACCCCAGCATCACCAGGTCTCTGCCCATCTCTGCAGTCGTTAAAAGGTCAAGTTACGCTTCATGGATAAACAAAAGTGTTTGTTATTTAACAAACACCTCTTATTTGATTACGTTACTGTGTTGTAGTAAGATTGTAACTTACTATGAATTAACTGGTAACAGTAAAATAAAAGGCCAAATTTTTGTGATAAGACATACCCATATATGCATACTTACTGAGTTGTAATGGGTATGAAGTTGATGGCACAAATTTACTAGTGAAGTTTACATGGTCACTAATTATGATGCAACTTCGTTTTGATGCTCCTAATTTATGCTCAAGGCTAGCACAAAATGTGTCAGCAGCAGAATGCAGGTCTTAATGTGTCATATACACCTTAGGGTGATTTTTTATACAGTATTTGAACTGCTTTTATATTGAAGAGCAATGCACTTGATTAAATTTACAAGAGGCAAGTGCCATTGGGTGGGTGGAACTGGATAGATGTAGGAAACGGCACTAGCTCCCAGTTGTTAAGCTTTTTACAGTTTCTTTGATGTTGAACTTTGTATGTAATCTCTTTTTATATATGAAGTGATTAGGGATAAATAGAAGTGCAATTGTGATTAGAAGAGAATTGTTTTTACTGCTTTATTGAGACACTCAAGTTGTATGCATTCTACAGAACCATTATTCAGTTTTTTTGTTTTATTTTGATGTCAGGTAAGGATATATGTTATGGTAACAAACCAAATTAAATAATTTGTTTTATGTGAATGAGGTATACAGGCATCGCTGAGCTATTGTTTTTTAAGTTTGTCCAAGTTGACATAATGAGAAAGAAATCAAATTCCAAGTCATGACCATTGTCTTTAGGTTGTGCAGTATTGATTCCAATTTGGTATAGTATTACAAGAATGTGTTCACTGTATTTCTTTGTTTCTTCGTGTTGCTGAAAGCATAGTCCTCTGGACGAACCCCAGGGATATTTTAATGTATGAACGTATATTTTTGTATTGCTAAACTTTTAGATTTGTAAATTTAAATACGTGACCCAAGAAACATGGTATATTGTAATAGGTAATGATATTGGCTGTTTTAGTTTTACAAATTGCTTATTGTAATTTTTTACCTCTTATCAAAAGTTGTATGGAATAGCATACTGTTTTCCTCTGAGGCAGAGAACGTGATGCTTTTTAATTGCTGCCTAATCTCTTCTACCAGGGTAACCTAATCTTCATACTGCCTGTCTGTATTCCATCTCTAGGATATTGCAGAAATCAAGTAACAGATTTACTTTTTGATTATTGTACTGTAGTTCATATCAATTTAACAGGACAAGCTTGCTGAGGTCATGTTAACCATTGAACAGTGCTGAATTTCCTCCTTTTTCTTTAATGGTCAGCACATGGGTTGCTTTATACTGTGAAAATCTATTAGTAAGAAGCAGCAATACTCAGGACAGTTTTGTGGCGAGATACCTTGAATTTTTTCTTGATTTAGAATTATATCAAGGATCCTGGACTTGAACAGACTTTGCCAAGATTCTTGACTTTTAAGTTTGAAGTTTGTCCTTAATTTCTATGATTTTCTACCTATAAGGATCTTGGTTTTGTATTTCAGCTTTGAATTTTAGCTGGATTTGAACTTCTGATTCACTTTGTGCCAAGTATTTGCATTCTAGTTTTCATCATAAAGTGCCAATGATCATTGCCTTGCTTTCAGAAGTGTCTTGGGTTGGACATACTCTGATACAAAATAGTATTTTAATTTTTTGTTTTTTAACCTAAGCTGCTAAAGTTTTTCAGTTTTGCAGTGTGAATTAATAGAGAATTAGGATCCAAAGTAATATGTGGTCAGAACTAGTTTCTTGATTTTGATGTTACTATTAAGGTCAGAATTTAATTTTAATCCATTTTCCCCGGTTACCGTAGGCTGTTATTATTTTTAAAAGTTTTGGACTTCAGAAACGTCCAATTTTATTTTGTCTTGTCGTTTTATATCAATGAATGACACGGAACAGTTAAATAATTTAAAGAACGTGATGAGCTGCAAGGTGTGCTCAGTGTTTTATTCGTGTCTGAGAAACTTAGGTATTCTCAGGTATTTTTTTCAGACTACATACTAAGGGTTTTCAGCATCCACTGAGATGGGGCATTGTGAATCATGAGCTAATATAATTTATGAAATT

>chr28:+:68629010-68630694

AGGACTGATATAATAAAAAGGCATCCCAAGTGAAATAAACTCTTGTTCAGTAACAATTTAACTGATAAAAACAAGCATATTACCATTCCATGAAACAATTAAATTTTAACGATCCCTTATCATTACAATGCAATCAGGATTAGTTGGGATAACCTTGACACTACCAGACATTAAACACTAACATCATCTCTCTATAGTAACATCATCTCTCTATAGTACTGGATTAAACATACATCAAAAATTCCCAGTCCACGTAGGTAGAAGAAACTGTACCAGGAAATCATGTACCTAAAGACAAGCCAACAGAGAAAGCACAACTGATACTTGGTAATGCTTTTCTTAACAAAAGTGAGGATATAACATAGTTGTTAAATAAGATGAAAGTATATCAACATTATATTCATGCTAAGGGGATTTGGATGTACCTCCTTTAGAGGCAATATGTATATACAGTATCTTTAGTATCAAACCCTATCATCATAAGATATCACTTTTCCAACAATGAACAGCAAAGGGTTGTAAGTTGCTCTTGAAGAACAGTTTCAAATTAGTGTTCTGTTACATTCAATAAGTGCTGCAATATATCTGCATTAAGTACTGTACTTTCAAAAATACAAAATAAATCTCAGACTAAAATAATAAAAAACTTTTTTATATTTTAGATTTGAAAATAAATGTTGTGTATAAATGCCATTCTTATTTTAATACCAGTAGGTGCAACCCTTTGATGTTCTATTTTAAATCCTTAACACCCAAGACATCAGTAAAAAGCCACATACACTACTTAGTAAGAAAAAAAAGGCCATGCCAACAAATGATGCACAAACCTGAGGTTGTCTTTCACATTATTCAAAGAAAAAACTAAATGTAAGATCAGCGTACAAAGAACTTGCTTGATTTTGTATGCCAAGAAAAATTTATCACAAAATCTGTCATATAGTATTCACTTGATGATCAGCAATAGCCACAACTTCCTAACACATGTACACCATAAAAAAAAGAGGAATATGAAAATTTTTTAATCAAATTGTATTTTTCATAGCTAACAAACCTGATTTCTAACCAAGTGATATGAGAAAAATAATTAATACTTCTACGAGGGCGTACCTGTGAATCATAATTTATTTCGTGTTCTCAATGGAAGGATCACTGGTTTGCAATGCTTTAGAGACTTTGAATAGAACTCAACAACAGTTTTCATATCCCAGCATCAACACAGCTGTTGTTAACTGAATATTGCCTCATTATATGAGACAGTCCTTTAAGTTTAAATCTTAAGTGCATCAATTGCTGGTACTACATAACAGTATAATGTATATATATATACAACAAATTTTAATACACTGTGTAAACAATGACTTAGTTTGAGAATATCAATGTTAACAGGTAAATATACTAAAACAGATCTCAAGATTAAGAGCAGCTCTGGCACAATACTCCAATTAATTTTTCCTGTAAGTATGACATAACTAGTAATGTCCCTTATGATTAATAAAATCCAATATGACTAAATCATAACTAATTGTGTGAACTTATATTACCATCAGATAACTCATGCTCTACATAAACCGGCTATTAACACAACCTCACTTAATTTTGTTTCTTTACTATTACTTGAAAATTCGTATTTCAAGCTGGTCAGTTTCTACAATTTAGGAAACTTGAACAATTTCTTCTTCATTTAC

>chr39:-:12164478-12165941

ATGAGGAGAGATGGACCGCCGAGGGAAAATCGTCTCTCAAGTGAACGAGGAGGCCCCGTGCCATTCAGACCCCGTGATTCTGCCAGACCGAAGTCACCAAAATGAAGAACTCGACTCTGATTTGAATGTGGTGATGACGACGAACCAGAGACGGCTGCCCTCCAGGCTGGGGAATCACTTACAGGGAGCTTATTTAAATGGAAGGAATAATCATAAGACAATATGGTTGGTCTTGCCTTGTCCCAACACAGACCAACCCATTGCAAGCGAGGTTGTTGCATTTGCATTGCTAGTTTTGCTCTCCCTGGTGATTTCTATATTAGAACAAGTGTTGTAACCCCAGCCTGGTGACTCTGCACCCCACGTCCGGCCCAATTAGTGCTGTAGCACGTGGCTGCATCATCCTTCTCCAGTGCCTATGCTGCCTGCCATTATTATATGTATATAGTGAAAACATTGTGCGCTGTTAGCTCCGTATGCAATGTGCTCGACTGGAGAGTCAATTATGACCGGTGCCAAAATTATGGCAGTTTTGCTTAATTGTACAGTTGAGAGTTTATTTTTGACAATAATAGCCTTGTAAATTAATGAGAAAATTTTGGAGCCATTCCTCAGAACTTGGTGTGGCATTGTGAATTGTGTATAGCTACTTATATTAAAAGAGTGCTTGCCTGTGACTTGGCTGAATCAAAAACCATGAACAGCCATTTGAGTGTGTGATGGCAATGATTATTACTTGGGGACAATTCCTTGTCGTCGACAAGGGCTCCCTCGTTGGTGCTCAAATGAAAAACATTTTACAGGAATAGAAAAACATAGGAAACAACAAACCTTTAGTTGCATTATTGGCACCTCCCATTATTTTTGGCTGTGACCTAAGAAGCAAGTTTCTTCAGTGAAGAATATGTGGCTTGCTTGCTTTACCTAGAAATGTATCTTACATCATTGATGAATATATGAAGAGGAAGAAGTGTTTGTTATTATAGCAAATTCAAGGAGCTGCTAGTGAGTACATTGAATATCGAAGGGCTTTGTATGAGTGAAGTGCATTTAATCTACAGTGATAGCAATTCTGAATGCAGAGTTGTTTTATTTGAAGCGCGAATGGAGCAAGTGTTATGTGATATGCAAGTGTAAGTGTTTGTTGTTTGTAGTGAAGAATGGATAATGAGTGTATGCTGTGAAAATGGTTTGATCAAATATTGGGACTTGTGCCTGGTTACTTTTACTCTGTCTTTCGAGTGGTTCATGCTTGGTTGTGTAGCCCCTTGATCATCATTGACTACATGCTGGCATAAGATTGGGATGTGTGTATGCATTGCATAAATGGTGTTGATGTACATAAATGTGCTAAAGCTATAAGACTGGCAAGTAAGGTAAGGTTACCTCCTCTCATCCTGTGTAGAGACCCAGGTTGTGAATGAATGTCTTATGGAGTGTGACAAACTTTCTGATTAAAGTGAG

>chr4:-:137784550-137784565

GTTGATATAAAATTAC

>chr4:-:137774842-137778134

CTGCATCCCCTCCTGTTAAAGAAGGAGAGGCTGGGTACAAAGACTGGGTCTTCATAAATTACACTTACAAGCGATTTGAGGGTCTTACCCAGAGGGGTCTTGTACGACCTAGTAAGCAGAAGTAGACCTTGCCAGACTATAATGATAATCTGTATTTCAGAAAGTGACATGTAAGATAAAAAAAGATATTAAAAAGGTTTGTGACTGGGAACTTGAAAATCCTAGAGCCTGTGATATAAATGGTGAAGTGTTATCCATAGTTTCCATAGAACTTTTTACAATGGCTCCTAGGATTTGCTAAGTGTGACGACAAAAGGTTTTTGTGGTAAATATTTTAGACCTGATAAGATTTGCTGGATGCTTCACTGTAGGAGTGTTGTCCAAGAAATCAACAGTACACAGATCATAATTTATCTTAAATAATCCATCAAGAGCACTAGTGTAGTGTGAAGCAGCCTGTAATCAATCAATCTTTGTTCACAGGATTTTCTTAACAGAGCTTGTGTAGTCCTGTACGCCAAGCTAAAGCAGAAGTATTACTTTTCTCCCTCCTTTTAAACTGTCTCACATTTAAAGAAGGCTGCAGCAATATTTCGCACTATGTGGGAAGTAGCGAGGTCTTATATATTTTATTAATATAATTAGTTTTTCCTGATGGGACCCAGTGAAAATTTTTTTTCATGATTAGACTTGTTTCTAATTGAAACATTGAGTTATGGATCTTATTATTATGGTTTTATTGTGATTTTAAGCCTTACAGCTGAAGACAGACTGTGATAGAATATTGGCAAAACAGTGGTAGGTCTTTTCAAGTATGCATACATACTTACTGAAAACAATTTTTTGTTTTTTTACTTTTATTTTATCTTGGACAATACAATTTCTGTATAGTTTGGTTTAGCACTGGTTTTGTGCTTGAATTACACACACACACACACACACACACACATACACACACATTTTTTAACTCTGGAAGACAGATGACATATTTGTGTGTGAAAGGGCAAAATGATGGAGAATTAATCCTCAGAACTTTGTGATGTTGCCTTCAGGTGTAGCATTCTTAGCCTTGTCATATTATCATGCATTATTTTGCCATTCCTTTTCATTTGAAGGCTTTTATCATATCATCTCATATTTGGTATTTATTTTTCCATACTGAATCATTGTTATTCAAATGATATAGGAGTGGCACTCATAAATTAAGTACAATAAAATACTGCTGTACAGTACTTGGCAATTCATCTTGATGTGCTAGATGCTGTAATACTGCCTCTGCTGTTTGAAAGGTTGTGAACAGTTTCTTAACTACTTTCCTCAGATGTTTAGTAAAAAAAACATTTGTTTACTTGGTAATAATTATCCACATCAACATAGCATCAGATAGTTTGCAGACTAAACCATTTAAATTTTTTAGTTAACTTCTGACAAAAGTAATAACACTTGCATAATATAAATTCCCAGGTCCATAAAATGTAAAAATGCAATTCACAGTGTCTTTCACAAGTTGAAGCATTGAAAATCTTTCCAAACGTTAGTTTTTCCACAGTCATTGGTCATTTTCCATAGACAGTTTGAAGCACACATCAGTTAAGTACAAGTGGGTTGTCAAGCTTGTTTATATTCTCGCTGTGTTTAGTTTGAGTTGGGTAACCTGTACTTAGGCTATGACCTGTACTATTATGCACTCTGCAAATGTGTAATTCACTAGCATTGTAATAAATAATGTGATTTTCCATAAGTTACTTTCTCATGCTGTACCTAAGCGTGCTATGTTAACTTGATTTTATGAGCACTATGATAATAGTATCCAAGATACACAGAACTTTTAGTTTCAATATTAACTTTGTTAGTTAGGATCATGATATATCTATTTATTTTAATGGACTGTAAATATTTTTCCTGTTATGGATTATCTGTCGTAGAATGATAAATGTGTTACATGTTATTAAATAAATTTGTAATTCAGTGTAAATGTTTTTAGGAGTTTGCAGTTTAAGTAAGGTTTTTGCTTTTATGTTTATGTACTATTATTGTGTATATATACAATTATGCTGTATAAGCATATACTACTAATTTACTAAACCTGTTCTGAGGAACATTTTCTCAGATTTTGATCCTTTTAGATTTGGTTTTTTAGTTTCCAGAAAATTTTTCAGTAATTAAAAGGTTTTATGTAGGCAGCATTATGAACCTAAAAGAATTTTAAGAAAAATAAACAGATTCTTTCTCCTGAGCTAAAGTACAGACAAAATTGTACAGTGTAGGTGTGTGTTCTTTCTGCATTAGTATGTGGTTTTGAAATCCTTCATATGCATAATAGTCTCAGTATTCAAGTGTAACACTTGTGCAAAATATAGCTTATTGCTGTGTTTTGTCTGAGGTACAGTATCAGGAATTGAAAAATTCAGCTCCATAGCAGAGTGAAAGTAGTTTACGATAGTTCTATAGAATACTTAGATTTATTTTTTTTTTCAATTGTATTAAGGGTAATAACCACATACTTGGTGATCACTAAACCAGTGTGCTAGCCTTTAGATGCATACAAGTTTGAGGTGTATTTATATAAACTTCATTAGAAAATTGACTTCTTTTAGTCTTGTCTAAATTACACTTACACTGTTTTGCTGCAGGTTCTAATGTCTTAAATATACATTCCTCATCACTTGTAAGTAAAGGTCCTAAAGATATATGCCATATTGTATTGGCATTGTCAAGTAAAATTTCTCTGGTTTTTAAATCTCTTAACTGTATAGCTCTCCTGAGAGTGTGAAAGTACCGTCCGTGGGACATGTATTAAATAAAGGGGTAAAACAGTTACTTTGTTAGAGGAGTTGAATACCCCTCACATGATCAAAGCATTATAACCTGTTTCCTTATGGTTTATTACTGATCACGGACAGGTTTCCAAGTTTTGTTTCTAAAATATGTTCAGTCACTTTTTGTATTTAGATAGATGGAATAAAATGGACTAGATGTTTTCTATTTCTCTAGATCTTAATTTGTGATTGAACTCCTGAGTTGTGAGTGGTATAATTATCAGGAAAAATTTTTAAATTTTTTCCTTGCACAACTAAGTTGTAGAGGCATTTATAATGCTCTATAAAATAATTCTGGAATAGTACGTAAGAAAATTGTGTATTAAAAGTGCCCATTGCGACTGTTGAACCCCGTAGGATCAAAAGAGTTCCTTTTTACTGTCTGAAGAACAGTATGGAAGGGTTCTTAACTGTGGGTTTCCAAGGGAGATCTCGTGTAAATATAAACAGCAGTAGCTCCTTTAACTAGTCACC

>chr3:+:121674885-121678039

GTTTGAGAGGGATTGCTTGGGTTTCCACCTGTACATTGACTCAATTTTTCTGATGCTTAGTTCAGAAAGTAAACTTCTATGAAGATAGAATTAGTATACAAGTATTTAGCAAAGTAGGGGAAGAGAGCTGCACAGAAAGGTATATACTTAAATACTTTTTTATTCTTGAAATTTGTGGTTGAATTCCTGAAAAGTAAGGCGTAGATACTGTATTCCTTATAAGTTATAGTAGCTCATATTTCACAACCTGATACACATGCTTAAAAGAAACCAAAGTTTATAATACTAGGACATACTAAATTTGATTTCATTTTTGCATTTATGGAATGGTGCCGGAAGAGAAGTTCATACGTATTCAATGATGACTGTACTAGTACTTGGATTGTGAGCAAATAACAGCGTTCTCTTAGAAGATAGAAAATACAGTACAGGGGTAGATTTAGCAGACAACAAAAGCCTATTTTTGTATTTAATGAATTCAAGGATTGATTTAAAAAGAAGCAGCAAATGAGGTTTTTTTATGAAGTGTTTACTACTTTGATATCTATTTCTGCGTAACATGCATATTTGGTTGCATTATTTTTATGATCTCTTGTTAATACTCTCCTTCTGTTAGATATAAAGAAAATGATAAGTGCTTACACTGGCGCTTCATGAGTAAAGTAGTTTTGTACAGCATGTTATTGGGACCAGCATGATAGTTACAAGTCAGTGGATAGATTGGTGTTGACCTGACGGTGTGTTCAATCAGGGAAATGGGTGTATGCATTTCACACATTTCTGTTCTCATTTTGATACAAGTAAAGCTAAACGAATAACAGTACTTGTATCCATTTTAGAGTTGAGAACTTGTAAGTCATATTGTGATTATAACATGACTGGCAAATCAAAATTGATAGAAAAATATAAAAGATATTCTTAAGCATTTTTTTACTTTAGCTTGTTTTCTCTCTTTCTCTCTCTTGAGGCAGTCACACAAGAACCAAAGTCTTATTTTTGTTTGTTTGTTTGGCTATGAATCATTTCAATATACCTTTGAAGTTGTAATGTACAACCCCATAGATCTGTAGCAGTGTTGTGGGGTGACGGTTGTTGCTGTAGCTGCGGCTGGTATTTCTGAACTTGACTTCACTGTAAATATAACACAAACACACTAAGGAATAGTTTCCCCCTCTACCAAGATGATAAATAACCATCACATTCCTTTAGAAGCATGGAATTAATGAAAGTGAAATGAGCATATGATATGCAGTGTGCATTTACACATCCAGTATAAAGTATATGCAATATATGCTTCTTCAGTGTATATGGAAATGCATAGTTTAATTGTATACTAACTTTATAAATTTGTAATATATAGTATACTTTAACTTATTGTACATATATACTGGTGATGTCTTATTGCTCCTTATAAAACAGAGCATCTTTGCTGTATGTGTCTGCACAGAAGTTTACATCGCATATCATAACCTTTTGGTATCGTGTTGAAATTGCTGTGCATTTATCTCAAGTGTTCACAGATGTTAACAAAATTTTATGCACATTATGGTTCTGGTAACTGCTATGTATCAGCATATAATACTACATTTATTTTGTGTATAGTAGATGTTTGAAGTGAGTGAATAACAGAGTGCAGGAAATATTTATACCTGTTTATGCCTTAGCTCCAGTTTGAGGTCAGTGATAAGTATACACACATTTATGTATTTATTGAATTAAAAAGAAATGCAAAAAACAAAAGTTGTTCATTATGTATGGAAAGAATTTTTTTCTCCATCACATACTGTGTCAGAGGCAGAAGTCTGGTAATTGATGTTTTTTTGAAAGTGTTCCCTTACCACATGGATGTCTGTTTCTATTATTTAATTTTAGAATTTGTCTCCTTTGTAAGGTATTTAGTGCCAATGCTGTTGAATATTCAAAACTCAGATTTTTATAATTTATTTTAAGTACATATGATTCTTTTTTCATTTTTTGTGAAATGTTGAAATTTTTTGTGCAACAAATTTCTGTTAGAATGTAAGTTAGTAGTCTAATATTCCTGTATTTTCTGAATATCCTAAAATTAGGAATATGAACCTATTATTATGAAACTGTGCATCTTGGAAGTATTTTTTTTTCATTAGTTTCAAAATGATTTTGAAGTGCTGACATTCATTACACAAGCAAAAAGGTTATAGGCATTTTGCATTTACTTTTATTTTTGTAGATCTTGGTAAATTATTAAGATTACCAACATTTCATGTTCAAGTTTGTACAAAGAATATATTCATTAGTTTTCCTTTAGATGTATTTAAATTCCATATTTAGCTTGGGCCATAATTTTAGATAAACTTTTTTGGATGAGGTGATATAGCATTTGCCATTCTAGTTTTCTCTTTAGTCTTGACAGTTATTCCAGGATGCACCTCTCCTTTGTACATAAAAATTTGTGCATCAAATTGCCACACAGCATGTAGGAACCAAGTGATAGGAATTGTGATTCTTTACCTCATATGAAATTGAATATGGTTGTTGAAGAAAACTACTGATGTCTTGGGATAAAGATATGATGTCTAATTCACCAAATTTGATGACATTTGTCGTGGCGGCGACATACGTATTTGGTAACGTTAATTTACATATTAGCCATGATTAACAGGACTTGAACCATTCCTCTAGTGCTTCTTATTGTAGACATCCCATTGTACAAAGCTGTGTTAAACAGATTTAACAAACACCATTTTCAGTATCATTTGTCCTCCAGTTTAATGACAATAAGAAGAATTGAAGTAAATCCTTCACATATACATTATATGTGCTACTCCAAGAAAAATAAAGTAAATAAAATGAACGACATATCAGTAATTTTTTTTTCAACAGTAAAGCACAACTTGGTGCTATATGCTCGTGCTGAATACCCTTAACAGTGCTGACCCAAAACTCAGTCCATGCAATAGTGCAACTCATCAGCCAGATTTGCTACACATTGTTGATTGTGCTAATGCAGGGCTTACTTACTAAAACACATTATTCACTGGTTACGGATATAAATCTTAGGTCAGCACTGATTTCATTGCTACTCCCATGTCAAGATTTTTGTGACGTCTACTCATCATCTTGTGAAAACTTCCAAATTTGTTTGTAATCATGCTAGAATAAATAACCTCAAATTG

>chr20:+:26864305-26866548

GTGAACTGTCTTTTATAATGGTTTTCTTCAGCTCACAAACATGTGGGGAAAAAATTCAAAACTTTTTGTGAAGAGATAAAGAGAATTTGCAGCACTCTTAAAGATAAATGTAGCAAACTGAAATACTCTTTGGACTCATTTCATAACTATAGATGTAAATACAGCACTGCATAATGGATATGACTAAATTGTTTTCATTTCTGGAATTGTAGAATTACTGTGAATTTCCCCATTTCCCAACCTAAGAAAGGTTGTCCTAGTTGTTGCATCAATGATTACTTTTTCTTACAGAAGTAAATCTTGTATGTGTGAAGGGAAACTTTAAGGCTACTTATGGCTGTTTGGTTTCAGTAATAGGATATTGCATGTATAAAAGGACTCTTAGCTCTGAATTAATCTTCAGTTTATAGACGTTTAATGGTACTACATACTTACATTGCTCTCAGGAATGTTTGTTCTCACTGGTAATTTTAGATTGTATAATAAAACCAAGTACATCATGTACTCTATATTGCTCAGTGTTATTTGGCGGAAGGGTATTAACTAAATAATCAGCTCTTGTAAATCATGACTACAGTATTTTTACATACAGTATAATTCCTCATACTATATAATGATTTTCTTTAAATTACACTTTGGTACTTCATTACATTAAATCATATGTCATAATCATGCTTCAATCATTTGTCAGTGGTTATGAAGTAGTTATTAGTTTATTATACTAATATGCTTGCAGTTTTGAAGTTTACCACATGAAACATTTATCAGAAAACTAATTTTGACCCAAATTGTTTAAAGGCCTATTTAAGCTTTAAATCCAACATACTGTAATGTATTTTGATATTCATAATGCAAGTTAAATTAGATAAGCATCACTTTTGTGTACCAAGTAGGCTACAGTGGTCAGGAAAATAAATGCATAATTTGAAGTTACTTTTCAATGTGGGAAAATTATGGCCTTAAAGGTTAAGTATATACTTATAATCCAATTTTGAAACAAAAGTTAGAAGCAATTTAAATTTAATTTTTTAGGAGTATAGTTGTTTAAAAATAATTTCCTATGAAGTTTGGAGGTCTAAGAATTGAATTCTACAGCTGTATGAAACCTTTTGCACATAAATTTTTTTTTTCTTTTGGATGAAGTCTGGTGGAAACTTGTAAAAGTTGACTTTCAGCTCACGAGGCTTTTTACATTTGTATACCTGGGAAAAATACAAATTACCTTAAAAATTTGGCCTATACATCAGTCATCAGGTTTTGTAAATTATGAAAGTAGTCACAAAGCCTTGCTCAGTTATTTTTTTTCACAGTGCTGTAAAGTGATGAAAATTTTATGCTTGTGCATGTAAACTTAACCATTTCAGACCTATTATTCATAATTACTATAAACAGTCCTTATTAGTATCTGTATGTATGGTAATAGAGGGATTTTTCCATTCTTAGCTTAGTAAGCATGACATTTGACTTGTTAAAAATTCTGCGGTTTCAGATAAATTTTGGAAAAATATTATCCACTTAAGCTTTGCCATTAACATGAGTTACAATATTTAAGTCTTATGCTTTACGAGAAAATCCATATATCAGAGGATTCTTTAGGTAGCTGACAGTATTAAGAAGAATTACAAACCATTACCTTCTTTAGGTAATTAAGTAGTACTTAATACCTTTTGATTTTTTTACATGAATAAAATATAAAATTTTTGCAAATAAATTGACAAGCATTAGGTTTTATTTTGAATATTGTGTGTTCATAAGAGTTGTAATAATATACACTACATTATGACTATCCACTTAAATATTAAAAAATAGTTATGATAACTCAATAGTGGTCATACAGTCAACTCTAAAACTGTGAGTTAATGGAGCTTTTTGAATAATAATTCAAGAATCATTTATGAACTACTGAGGCAGTAACAGGGATCTAAATAGCAGTACTTCAAATATAGTCAGAATCCTTAGACTGTAACATTATTTTTACAATATGAAAAACTATTTTGTTATTTATTTTATTTACCGATCTTACACTAGGAATGATCTTTTTTCATGAATTCAAACCCTTACAAGGCACTGAAACTTGTTTCTTGGTGAGATAAGAGGGTCAACTATGTTTGCATAAAATTACTTTCTGTGAAAGAGTTCTTCTATGAAGGCATGTTTATGTAAATGCTAATTAGAAATGAAAATTGCCCATGTTGCCTTAATTGTAAGATAAACATGTGAGTGGTAGTCTCCTGTATGTATCAT

>chr10:-:26394211-26394258

ACTACCAGGCATTTCTACTAGCTGAGCAGACCACGCACGGACTACCTT

>chr10:-:26383662-26383839

ATATAAAATGGGTGAAGATAATGCAACTGAGACCCCCAAGCGCAAGGCAGGGAGGCCTGCAAAGCCTGAAAGTGAGAAGAAGCAAAAGGTTGTTGTAGCCAAGGCCTCAGAAGCAAAGAGAGGCCGAGGCCGACCCAAGGGCAGCAAAAACAAGCCTAAGAAGTTGGTAGTAACAAAG

>chr10:-:26382802-26383467

GGAAAGCGTGGTCGTCAGGCAAAGAAAGAGTCTGCATCTGAGGAGTCTGCTGAAGATGCAGAGTAAATTTAATCATTTCATTCATGGAGCGATCAAATTGCCATCAACCCCTAGGCTGACCTAATTTCTTGAGATGTCATCACTGAAGGCTGGTTATCACCAGTTCTGGGCTTTGTAAAATATCCTTGATATTTATTTTCATTATGATGCATGCAGGATAACTGCAGTCTGCTCATCCCTCAAGCCAGGTATATTAATGCTGTACCTTGAACTTCCAGTCAAAATTATTTATTTTTATATATTAATACGTAATGGAATAGATTTGATGTCTCAAGGAGAAAGGATTTCAGACAGGGTTAGATGGTGAAGTGGTTAGGTGTTTTTTATGTGTGGTATTGTAGGTGTAACAGTGAGAAATTCATTATCATTCTCTATTCAGAGTACAAAATGTGTAGCGAGTTAAAGTTTTTTTCTTTCGTTCACATAAGATGGGAAGAAAGATCCGATGTCCCTTTTTCATTGTCATTCATGTACTGTATTAATGCATTAAAAATGGGCCTCAAGTCCTCAAGCACTTTTTCATTTAGGGACCAATCCCAGGTCATGACATTGAGGTCAGCATTTTAACATGCGATTTTTAATTTGTATTGAATAAAATTTTAGTTC

>chr24:+:77333740-77333763

CAATGACCGTCGATTTCTGAAAGG

>chr24:+:77336858-77336993

GAGTCGGGAGGCTGCGCTCAGGAAGTGAAAGTGAACAGCGTGTCAGTATGAGAGTGCATCGGTCTGGAACTTCTAATGCCCAGACACCAGAACTTTCAAGACCCACAGATAGATGTGTAGAACAATGGGCAAATAG

>chr24:+:77337496-77339872

TGTGAGTCCCCCTGTATCTGGAGGAGTCATTCGACGAGGGTCTGCAGGCAAGGAAGGAACTTTCCGATATCCGACAAAAAAGTGAGAACTAAGCAGCCAGTAGGAACTTAATCCCGGCAGTGACAGGCAACAACATGTGATATTTAAGGAGCAACTGCACAGTTGTGATAAAAGTCATAACATAGATTTGAAAGAAACATTATGGAAACTTTCTAGTGCAGTTCTTGGTCCTCGTGGGTACCAGCTCCAGAGTGAGTGTGATGAAGTCCATTGTGAAAACTTTTTTTTAATTTTTTTTATGGGCCAATCAAGCTCAAGCTCATTTAATAAAACTATTTTTGAGTGAATGTCAAGGCCTTATAAATACTAGCTTTTATGGAAATGTGTGTGTACACAAACTCAGTGAGTGTAATCATGTGATGCTGTCAGTGCATCTTTGAATCCGTGATTTAGGAATGAAAACATTTATAGGGAAGAAGTCACCTTTGTCCTTCACGAAATCCATTTTTTTTTTATTCCCTTTCACGTGGAAAAAAAAGTGTTGATTAATCATATTAAGGTTGCAGCAATACTTTCCAAGTTTCTTAAGCTTTCTATTTGTAAAAGTACTATAAACCAGTAACTTCCAACAGTTTACGTACTACATATATTACAGTGTTGCGTATCGTGAAGTTTTAACCCAGAGGTAGCTAAATATTGTGAGGTCCCTTTTCCGCAGTTACACAAGACAGTGTTTTTTTGGTTATCTAGTCCAGGAGTTACTGTGAAAGGCTTACTTACTAGTATTACTCTACGTCACTGAGAGGGGGTTTCTTCTGAACTTTGGCATCACTTCATTTACTGCATTAGCTTTATCTTTTTTTATGGAGGAAGTCTTTCAGATGACCAAAGAAAGCATTTAAGGATCTCGTCATTTTTTTAATGGTCTTGTAGCATCTTTTGGTATATTGTGCCTTTAAAACATGGGATCAAGTAGATATAATTCTTTTACGTGACCAAGCTGTATATAATTTTATTTGATATTGCAAAATAGTATTTTAATTTGAGTGATTTTGCATCACTCTGAACAAGCTTATCATTTCACTTCTTTTGACCCTCTCTGAAGTTTGTCTTAGCACTGCTTTAGCAACTGAGTTTATTCAGGTTGGTTATACTTTGGAATGTTGCTATACTCTAACAGTGTTGCCAGATATACTTTTTTTGTTCTGCAGTATTTGACTGCATTTACGTATTCACCGAACCTAACATCATCCCATTATTGTTTTATGTCAAATCAGATTTTGTAACAGGAAGAGGAAAATGCAGTGTGCAATGTTTTAAACTGGTTTTCCAAAGATATTCTTTATAATAACATATTTTGCTCTTGTGGAGTTCCAATTAACATCTCTTTCAACTCTCATTTTACGAGGTGTTTTTTCAGCAGCTGTTACAGTATTCTACATAATGTACAGTTACTTTTTACTTACTTTAACTGCTTAGTACATTATCTGAGTTACTGATTTTTTTTCCCATTTACTTCAAATGTTCATTGTTGCTGAACAAGCACCAAGATATTCTTGCTGTAAGTCTCTTACAGTAATATGCTGACTGAACTACAGGAAGACCACAAGCAGCTGAATGTACTTGGTTTACCCTTGTTGCTCTGTGATGTCGACCCAGCTTTGTATGTCAGTAGATAGTAGCCCTGTAAACCATTTATTTACTTTCTTAACTGCAGTATTCTTATTTCCATAACGCTAGCAGGAAAATTTCCAACAATCACAAATGTGCACCGCTTGAATTTTCAGTAAACTCTGTAAGTTATGAACGCTAGTTGTATAATCTGGGTATCTTGTTTAAAAACATATGCTCTTCTAGGATTTTAAATCACCTACTTTTTTTCTTTTTTTTTTCCATTTTCATTCCCTTTAATTTCACCATTTTTTATGCCAATTTCACCACTTTATAATACACCTTCTTTTATTTTAACCATTAAATTTCTTTATATAGGTTTCCCTTATTTTATAATTTAATAATCTATGCATCGTTTAGATAATTTTCCTACTTGTATAAGGGTATGCAATGTAAACAACCATTCTTTATTTTAATTGTCATTGTAAACTTGAATATATTTAAGAGGACAGTGCAACACTTGATTTTTATATGTCTTGTCAGTCTTTTATCCTTGTTTACATAAGATGCTATATTTCATCTAATTACCTTGTCCTCTTCCAACTGATTTCTAAATTCTTTGCTCTTCAGTATACTGTACAGTATGGGAAAACAGGCTGTACAATTATGTAATAATATGACTAATAAATGGTTCTAATTATCTGGCAACAATATGACATGTTCTTCCTGTTTTATTACTCTGTAGGGAATAAAGTGCCAAGACTAC

>chr24:+:2863095-2867223

CATAATGCTGAAGGAAAAAGTTCCAGGTAATAGCAGGTCGTTGGGAGTTTCCCTCCATTCAGTCTTCTACCACAAGTTAATTGCCTTGTTACCAAGTTTCAATGGCTGATTCTAGCTCATCTGAAAGATAGTTTTAGTTAGTATACCTATGAAAAACAAATTTAGAAATTTGGACCAATTGCATGGTTATATACTATTTTCATTTTAATCCATTTCACTTGGTACGCAGTGTTTTGGATACTATACTTTATATTCAGACAAATATTTAATTTTTCTGAAAAACATCTGTATTCATTCTCTACTTTGGCATAATTATCCCCATCAGCATTTCTGTAACTGTAAGTCAACTTTCAAAGTACATATTTTATTATATTTTCTAATTTTTAAATGTCTTTTGTTTGCCATATAGATCAGTGTGCGTTTTTGTTTATGCAAATCTAAGTCTTGCTTAAAATGATCTAAATTTTTACCCATCTCTTTTACATAAAAGCTCAGCATTAGTTTAGACTATGGGAATGGCAGAGATACAACGTTGGTTGTATAATCACCAGCCTTCAAAAACTATCTTATTTTAATACTAATTTTTTAATGTAAGGTAAGAGTGCAAACAGATCTTGGTTTCTTTTCTAATTTTACATAGTATTAAAACTATGCTAACTAGCTGAATGTACCAGCTCACTTATACCTTAAGGCGCAGGACTTAAAAAAAAAATCCTGTGACTTTTACCTTGGCTTGCCACATTACAGATGCTAATTTTATTTTGAGTTTTCGTTTAGTATATGTAATTTTTTTTACATTTCTGTTCCTTGAGAGTTTCCTTCCTTTGATATTTTCTTGTTCATTTTCTTCACTCCCAATTTTCTACTATGGTTATCTCCATTAGGTTTTATATGAAATGTTTGCGTGACAATCTTCCTCACGTCATACTCATTAGTATCCTTTGTTTAATGAAATATGATTTTATCGCACAGGATTTTTGCATATACCATTACTGTAACAATGATTGGTTGCTTTCAGGATTTGGAGGGCATAGAGAGTGAAATTTTTCATGTATGTGTACCCAATTCAAAATTTTGTGTTTGCTGTAGAAGCACTGTTATATGAATGGTTGTGTGGATGATGAGTGCATAGAACAAGCAGAAAAGGACTAGAACATTATCAATGAATTACTGTAACCTATTGATGGAAGGGTAAACTTGTAATCGTACAGAAGCAACATTTATATTTTTCAACAGATTTTTTAGTGTAAGGAAATTAATGTGATCATTATTGGGAGAGGTGTGAGTATAAGGTGGTATAAACTTTTGGCTAAATTTGTGTTATACTATTAGATAGTTGAATCAGACCACTTTCTATACTATTAGATAATTTGAATCAGACCACTTGATAGAAAGGGTGGGATAGAAGGTGGAATAGCCTGAGATGGTCAAGTTTATAGAAGGAAAGAATTAAATGGAAACTGCAAGATTCGAATCTTGACATTAGGAAGAGAGGCAGCATTTGCAGAGTTAGTTTGGCTTCATATCTGAAGAAAAGTGATAACGCTTTATGTTTTTTATGTTTTGATAACTTACTCTGTATGTTCCGGTGTATATAATGTATATACTTATCCAAGAATAGTATAATGATTGCACTGAAAATAATGCAAGGTTCGGGTGAGGAATACACACCAATTCAAGTTTTGATGCTTGCTACAACTTTTGACTTCATGATTTTTAGATTTTAGTTTTAGCTTCAAGGCAGTTTTATATTACGCATTCCAGACCTTTGGTCAGTTATCATTTTGTTGATTTTTTTTTTTCTGTTTTTTTTTTTTTTCTTTTTTTTTTAAAGAAAAAGTCTTTAAAGAGACTTTTCATGCACTAGCAAGAAAAGTGTTTAGTTGTAGGCCAGAGTGGAGCTTGAAAGTTTGGTTGGCATTTATTTTTTATGAAAACCTGTGATAAAGTGATTGGTTTAATACATTCCTTTGAGGATCTTTTTTTTAGGAATAGATTATATTCAGAAGTTTGTTCCTCCATATTTAGATATTTTGTGTGATCTTACACATGAAAAACTGACTTTAGTACTGTAGGAGGGATCATGACAATTCTGCCTCGGTTGCATCAGAGTAGTTTATTTGAATCAAATCAAAGGTTCTGACACAGAGGGCAACTTTATTTCTATTAACATGCAAATGAATACATCAGTATTGTGAAATTCTACTTTCTTTGAAGTGTATCATGCTAGTCATCGTTACTCTTTCATATAATTGAAAAAAAACATTTTCAGTATGTTAGTGCCATACATAGCTAGTTTTGAATGCAGTATATGTGCTTTGAAAGCAAGAGCAAATCACAAAATGGTACTTAATTTATTTTAACCAATCGTAGAAGGGCCACAAGTATGGACAGCAGATTTTATAGCTAGTGCCCTTTGAATTCATATTTTTTTGTGTACTTTCTCTAGAAAAAAGTTTAAGCATGCATAGTCTTAGTTTTCCCGAGTACAAAAGTGGTATATTTCTTTTGGGTGGAAATGTATACTGTAGCATTATTCATATATAGTAGTTCCAGTTCCTGTGGATTTAATTGATGGTACAAGTTTGTACTATAGTTTTTACAGTTATGTACCATTTTCTACTAAAGAATAGTGATACTGCAGCAACACTCCCAACCACTTTCTAGGGAGATGTTTTCAGATGTTTAAATACTGTAATCATTAAGCCAGGCAGCTCACTGATTTTTTTGTCCCAGTTACACGAAAAGCGATGTTGTGTGGGTTTAAGAGACTCACATTTTTAGGACAGAAGAGTCATAGAGGTTTTTGATTGCATTAGATTGTTATAATTGCAATTTAATGTAATCACTAAGTTACATTTCTAGAATTTTATAGGCCTTACAAGCAATTGAAGAGTTGGGCACCTAAGTTAGGAGAGACTTATGGGAGTACAGTAGACAAAGTAAAAGGCAGAAAATATTGCAGCAAAAGTATTGCAATAGCATAGAAAAAAAATTGGGAAGTAAATAGAAATTGCAAACTCAATAAATGCTCCGTAGCTAAGTTCTGCCAAAGGAATATTCAGTGAATAATACAGGACATAATAAGAATTTCTGTGTATGATTTCCATTCAGAGATCTGGGGAAGGAGAGTTTGTGGTGGGGTTTTTGAGTGGAAAATATTTCTTACCAGGTAAACTACAGGTCTTTATTTTTTTTTTTCCCAGTTTCATACATTCCACCCATTACATGTCTTCCACTTGAGAGGTTCATGCTGAAAAGTAGTTAAATCACTTAGTAAACTGATATCCTTTTTGTCCTTCGTCAGCATTGGTTCCTCTAAGAGATGCAGTACATTTGCAGCTTGGCAAATTTTCTCTCGCTTTAAGAGCGGCCAAAATCATCTGGGTCCCAGTCAAGATCATATAACCTTTTTCAGAGGCTCTGCATCCATTGTTGAGCTATAGTAATGAAAAGTTAAAGCTATAGTAGATTCACATCAACTGTGCCTCTGATGTCAAGGCCAGTCCCTTACGACGCTCCTGATTGGCTGTTTATAAGCCAATCACAGAGCTGGAGAGACTGTCTCTCGAGAGAGTTCACACAGGCAGGATGTATGTTCTGGAACATGCATCCTGACTATGTGAACTCTCGAGAGAGACTGAGTCTCCAGCCCTGTGATTGGCTTATTAGCAGCCAATCAGGAGCATCGTAAGGGACAGGCCTAGACATCAGATGCACGGTTGATGTGAATCGACTATAGTTAGGTTTGCATTCTGCAAGGTAATGCTGTTAGAAAGCACCTCTAGTATAGTTTTATCTGATGATGTCATAACTGTAACCTTTGTGATCATTCAGGGAAAGAATTCTCTTTATATAGGATATAATTGTTAGAAATTGCTTGGATATATCAAATAAGAAGCATTTTTGGCTTGATAATGTTAAGGCAAAGCAGTGAATTTTTTTCATGACTCATAAGTATGGGGAAAGGATACAGGTATTGCTTTTGTATACTGTATTTTAAATATAGACTAGCTTCTGAATATTTCACCAACCACATATGTGCACTTGCCTGAAATATACATGTATAAAATAACTGACTTTAGTCACCTTGTATAAACATGCATTAAATAAAGTCACTATTTTGATG

>chr33:+:33119485-33119551

TAAGAAGTTTGCCGGCAACATTGAGTTCAAGACCTATAGAGGCATGATGCATTCATCGTGTGAGGAG

>chr33:+:33148629-33151840

GAAATGGCTGACATCAAGAAATTTATTGACAAGTGTCTACCTCCTGTATAGAAGCATCTATGGACGTCACCAGATATCACAACATTAAATTTCTGTTAAAGTGAAGCAAGACTATCGGTGATTTTGGAAAAAGAGAAAATAGTTTCTCTGTACAAAATATCTTGGTTGCTAGAATCCTTTTAGAATTTTGTCATGTTTAGGCAAAGGTGTTACCAGGTATCTGAAAAATATAGTATAATATCAGTAAAAGTTACAACACACAAATCATTCCAATACATGATTATTGTTTATTGTACAACAAGTTTTATTTTGATGTAATATCTGGGGGTGTTGGTGACTGAAAGACATGCTTCAACTTATGCAATTTTTATTCTGAAGTTCTATAATCTTGTCTGGGACAAGATTTGGTTAAATAAATTCTCAATCATGATCATGATTACGGTAATAATGTTAGTAGAAATATAGGAGGTGATTCTCCTTACCATATACAGTATATATGAATATATGTTTGCTTTCTTATTTATATATGGTATAATCCCCACCCCCTTAATCTTGCTATCATTTGAGGGTAGTAGGCAATTGCAAGTGCTATATTTTGACACTATCTGGAGGTCATAGTGTGCAGGCAGTTGAGGCCCTCTCCCCAAAATTTATGAAAGGCAGACTATAGCTTGGATTATATGCATCTTTACACACATACTATTGATAGTATATCTGCTGTTGAGAGATTTTTGCTTCTTGGCATGAAAAGATATAGTGTAAAATAGGCATCTTGAAGAATACATGGTAGTAAGTAACATTAAAAGATGTACAAATGTCAGCTCTTTTGTTTATATAATAAATGTACCTGCCTTTTATTTTATTTTTTTGCTGAGTTGTTTGATATCCAAAATTACAAATATACAGTATACCGTTCAAATTCAAAATACTATGCTTTAGTATAATTCTGATTACTTATAAACTACTTAAGATATCATTAAAGCATTTTTAGTTTATAGAGGAAAAAATGAGAGGGTGTTACTGAAAATTAAACATTTTCATTTACAGCGTTATTTAATCACAAGGAAACCCAGTGCAACATGTATAAAAATTTAATAATTTTTAAGAATGCCTTGTAGAATAAGATCGGTTGGGTGATGGTTCTATGCTGTAGCAATAAGAACAAGGTGTTCTTCAGTACACTAATATATAAGTACAATTTTGTCACAGACTACATAATCTGGACTTACGGAAACCATGCAGTGAAACCTAAGATTTTGCATATTTGAATGCTTATTGAAAATTTTAAACATCAGAGATTGTTTCAAATGCACTTTGCATTTAAAATTTTCTGGTGTAATGATTAGCACTGTGGTATCTGGCTTAACAAGTGCCGTTAATTGTTTTAAGTGTGAAATAAAATGTTTCTTTTATGTTCGCCATGAAGAGTTGTCAGTCTTGTGTGTGACATGTTATCTATTTTAGGTGCCAAAGATACTATACACATGAAGCAATTTTGGAAGTACACCACTATCTTGCATGTTACTGGGAAGGTGTATATTTTGTTAAAACTTCTCATAAGGTTGTTCCCAGCTACTCTAGACTGTCAGTGATATGCCATTTGGCTGTAGTTTTGTCTCTCCTTTACTTACACTACTGTATCACTATCCAAGCAGTATTGTTTCCTCCCAAGATCTTTTGGCAGTCTCGTCACATTGGGAAGCAGTTTTCTTGGCAAGTCTTTAATAAATTATACAGTTTTAAACAGTGTTCATTTTATTGTGTATTACTGGAGAAGTATTTAGGGAAGTACATTTGTCATTCTTTACCAGTCCCCATTACTCCGTGTTTCATTACTCGGTGTTTTACTTGGATAGTGGTGCCCCATGATCCTTAGTTTTGTCACAAAGTAAGTACTTGTTGCATAGAAAGCCTTTCATCAAGGATTTAGTAAACACTGTAATCAAGTACTATTGTTTAACAGGTCCCTTATGTTAGTCACCCCAGGAGAGTAATAAAGGTTTGTAACCTGAAAGAAGAACGCATTAGGATGTAAACCCAAACCAGTAGCTGTAGGTCTGTTTAGAGTAAAAGGGCCTATTTGTGCTCTAGTGGGATTACCATGCCATTTTTCATTTCAGTGTATAATGCCCAGTCATCACTATGTGCTATTTTGGACTTTGTATTTCCTTTTCTAGCTCTTAATTTCCTTTTCTATTCCCAGATCTTAAGTGTTGGTGTGTCCTGATAGTTTTGTTTACTGTGATACTTCAGCATTTATTTAATTACTGGAATGTTGGATATCCTATTATGCAGAAGTTTTCATATGCAAAATGGTTTAGGTTCAGCTGTATGTAGAAATGGTATCACTTGCATTCTTACATATACATATTCAAGTTACAGTCCTTACTAACTGTTGAATTATACCTTTCAAGGAACCTTCAAGTACAGCATTTGTCTTCTGTCTCTAAAACCTTTTTCAATGCTGTTTCTCAAGTATTATTGAGCACGTGTGTTCCACATTCTTGTAGGACAACTGGACAATCTCTACACACAGACATAAAAGTCTTGCAGTTCACAAAAATGTAATCACCTTCTCGTGTATTGTTAAATTTACTTCGTGTGTTGGATGTGTTCAGTTCTCTATTCATTACACCTTTTAATGCAGTGTTAATTTAGTTGTCAGTCTTAATGACTTAGTTCTAACATTTTCATTGGCATCATACCAAATGCGATGTCTGGTACAAGTCATAATATACAAAATTATAGAAGTAATTTCTAAATGGATATTTTTAATATATAAAGCCAGTGCGTAATCTTTTATGCAGGACAGTGTATTATTACCTAAGATAAATCCTGAAGTCCCTTAGGAGTAGCCTTGACCTGTAGTTTTCCTTGTTAACAGGTTGTAGACACTGGTTCACATCAGATGTATAATCCTAATCTGGTGTGTATTCAAGTTTTGAATATACCTTCATTGGTTCCATTTACAGTCAGTAGGCAAAGTGTTGTTCAGCTAGTAGTATAGCATTATTCTACTTTCACTGTTATGCATTTTGCATTGAATGATACATAGGGTTACAGGAGCTGAATTCATATTTTTCATTGGTTAGCAATAACACATGTTTATTTCCATAAGAAAAGCATTCAGCTCTACACACTTGCAGTTATGTAATTATTTTTTCCTTATGTACGAGGTTGTAAGTTGTAAGATAACTTTCATAAAGGAATGT

>chr33:+:33149018-33151840

TGTCTGGGACAAGATTTGGTTAAATAAATTCTCAATCATGATCATGATTACGGTAATAATGTTAGTAGAAATATAGGAGGTGATTCTCCTTACCATATACAGTATATATGAATATATGTTTGCTTTCTTATTTATATATGGTATAATCCCCACCCCCTTAATCTTGCTATCATTTGAGGGTAGTAGGCAATTGCAAGTGCTATATTTTGACACTATCTGGAGGTCATAGTGTGCAGGCAGTTGAGGCCCTCTCCCCAAAATTTATGAAAGGCAGACTATAGCTTGGATTATATGCATCTTTACACACATACTATTGATAGTATATCTGCTGTTGAGAGATTTTTGCTTCTTGGCATGAAAAGATATAGTGTAAAATAGGCATCTTGAAGAATACATGGTAGTAAGTAACATTAAAAGATGTACAAATGTCAGCTCTTTTGTTTATATAATAAATGTACCTGCCTTTTATTTTATTTTTTTGCTGAGTTGTTTGATATCCAAAATTACAAATATACAGTATACCGTTCAAATTCAAAATACTATGCTTTAGTATAATTCTGATTACTTATAAACTACTTAAGATATCATTAAAGCATTTTTAGTTTATAGAGGAAAAAATGAGAGGGTGTTACTGAAAATTAAACATTTTCATTTACAGCGTTATTTAATCACAAGGAAACCCAGTGCAACATGTATAAAAATTTAATAATTTTTAAGAATGCCTTGTAGAATAAGATCGGTTGGGTGATGGTTCTATGCTGTAGCAATAAGAACAAGGTGTTCTTCAGTACACTAATATATAAGTACAATTTTGTCACAGACTACATAATCTGGACTTACGGAAACCATGCAGTGAAACCTAAGATTTTGCATATTTGAATGCTTATTGAAAATTTTAAACATCAGAGATTGTTTCAAATGCACTTTGCATTTAAAATTTTCTGGTGTAATGATTAGCACTGTGGTATCTGGCTTAACAAGTGCCGTTAATTGTTTTAAGTGTGAAATAAAATGTTTCTTTTATGTTCGCCATGAAGAGTTGTCAGTCTTGTGTGTGACATGTTATCTATTTTAGGTGCCAAAGATACTATACACATGAAGCAATTTTGGAAGTACACCACTATCTTGCATGTTACTGGGAAGGTGTATATTTTGTTAAAACTTCTCATAAGGTTGTTCCCAGCTACTCTAGACTGTCAGTGATATGCCATTTGGCTGTAGTTTTGTCTCTCCTTTACTTACACTACTGTATCACTATCCAAGCAGTATTGTTTCCTCCCAAGATCTTTTGGCAGTCTCGTCACATTGGGAAGCAGTTTTCTTGGCAAGTCTTTAATAAATTATACAGTTTTAAACAGTGTTCATTTTATTGTGTATTACTGGAGAAGTATTTAGGGAAGTACATTTGTCATTCTTTACCAGTCCCCATTACTCCGTGTTTCATTACTCGGTGTTTTACTTGGATAGTGGTGCCCCATGATCCTTAGTTTTGTCACAAAGTAAGTACTTGTTGCATAGAAAGCCTTTCATCAAGGATTTAGTAAACACTGTAATCAAGTACTATTGTTTAACAGGTCCCTTATGTTAGTCACCCCAGGAGAGTAATAAAGGTTTGTAACCTGAAAGAAGAACGCATTAGGATGTAAACCCAAACCAGTAGCTGTAGGTCTGTTTAGAGTAAAAGGGCCTATTTGTGCTCTAGTGGGATTACCATGCCATTTTTCATTTCAGTGTATAATGCCCAGTCATCACTATGTGCTATTTTGGACTTTGTATTTCCTTTTCTAGCTCTTAATTTCCTTTTCTATTCCCAGATCTTAAGTGTTGGTGTGTCCTGATAGTTTTGTTTACTGTGATACTTCAGCATTTATTTAATTACTGGAATGTTGGATATCCTATTATGCAGAAGTTTTCATATGCAAAATGGTTTAGGTTCAGCTGTATGTAGAAATGGTATCACTTGCATTCTTACATATACATATTCAAGTTACAGTCCTTACTAACTGTTGAATTATACCTTTCAAGGAACCTTCAAGTACAGCATTTGTCTTCTGTCTCTAAAACCTTTTTCAATGCTGTTTCTCAAGTATTATTGAGCACGTGTGTTCCACATTCTTGTAGGACAACTGGACAATCTCTACACACAGACATAAAAGTCTTGCAGTTCACAAAAATGTAATCACCTTCTCGTGTATTGTTAAATTTACTTCGTGTGTTGGATGTGTTCAGTTCTCTATTCATTACACCTTTTAATGCAGTGTTAATTTAGTTGTCAGTCTTAATGACTTAGTTCTAACATTTTCATTGGCATCATACCAAATGCGATGTCTGGTACAAGTCATAATATACAAAATTATAGAAGTAATTTCTAAATGGATATTTTTAATATATAAAGCCAGTGCGTAATCTTTTATGCAGGACAGTGTATTATTACCTAAGATAAATCCTGAAGTCCCTTAGGAGTAGCCTTGACCTGTAGTTTTCCTTGTTAACAGGTTGTAGACACTGGTTCACATCAGATGTATAATCCTAATCTGGTGTGTATTCAAGTTTTGAATATACCTTCATTGGTTCCATTTACAGTCAGTAGGCAAAGTGTTGTTCAGCTAGTAGTATAGCATTATTCTACTTTCACTGTTATGCATTTTGCATTGAATGATACATAGGGTTACAGGAGCTGAATTCATATTTTTCATTGGTTAGCAATAACACATGTTTATTTCCATAAGAAAAGCATTCAGCTCTACACACTTGCAGTTATGTAATTATTTTTTCCTTATGTACGAGGTTGTAAGTTGTAAGATAACTTTCATAAAGGAATGT

>chr38:-:17356940-17357142

GGGGAAGAGGAAGAGGCCGTGGCCGCGGAAGGGGCCGTGGAGGCAGAGGTCCTCGTGGATATTTCCGCAGATATTATGCTGGTTCACGTCCCCAACCTGAAGGCATGATGGGAGGACCTTCAGAAGGCGTTCACGCCGCAGGTTCCGCCGTGGTGGACGTTGGCCGTGGACGTGGCAGAATAGGTAGTGGACCAGAAACAAAA

>chr38:-:17354059-17354661

GGTGATGGACAAGGCCAGGAGGGACAGTCTGCTGGCTCTGGTCAGGCCCAGCAGGCAGTGGAGAACACAACTGCTGAAAGTTCAGCATAAGCTATCCTCATCCTCCCCTGCAATCAAGCCACCAAAGCTAACGAGGCCACTATTTAGGTTATCCAGATAAAGTAACCTGAACAGTGTTCTTAATTTAAGGGTGGATAGGGCTGCTGCTGCTGCTGTCATACCCCCTCCCAGCCCATCGGAAGCCCAGCAGGAGCTCTGTCCACCACCCGGAACTTCCAGTGACAAACAAACCACCGACAAATGAAAGGGACAGTAATGCAAGAGCATAAGCTTACAAAAACCAGCTGTTGCTCCCGGTTACCAGAAAGTCGGCTCTGACCACCTTCAGAAGGAAGCTAATGTGTCCTCTCTCCGGGTCCGGTAACAAAGAGGTCACGCAATTTGCAAAGACGAACCCACGGTCAAGGGTTGGAGTCTAAATGGCCCTTTCTTTGTCGCGCAACACCTGAACACACCATGAAACAAATGTACATTTAGGGTGGGAAAGCACAGTTCATACATAACAGATTAAATAAAAAGATTAAACAAATCACAATGGGTGGT

>chr17:-:58703476-58704683

ATGAACAATAATTTCTTCAGAAGATTCGTACATCAATAATCCTTAAGGATACCCAACTACAAACAGCCAAGAATAACAAGAGGGATAAAGATAGGTCTTTAGAATTTCTGATACCAGTATATCGCAGTTATTTGAAAAATGAAGTCTCTGGGTAATAACCACCAGAATATAAAAAAACAAATATGGCCTCGAGAAATAGCAGCTGAAGAGGTCCAATATGTACTTATTGACTGATCTAACTTTTTTAAGCCCAACGTGTTAATAGATACGGTTTAAGCCCAACGTATCATGTTAATAGTTATGGATTGATTACTAATTACCAAATTTTTATTTAGTTGGCCTTATACCTGTACAAGTGGAAGCCACATATAATGTAGTTATTATCAATCGAGATCTCAGCAATGCAATACACTCAGATGATTCCAAACCATCACGCTACAAGAGAAAATGAAACTAAATGTGCAATACAAGAAATAATCCTCATACGCTATGGATATGCCTATTGAGATTGTGGTAGTGAATATCTAAACGTGCAAAAATCTATTTATCATGCTATGTTATATCACAGCGAGTTGGCTCTTTGACTGAGTGGACTTTGAGGTAAAAATGCTTGAATATCTTTAAAAAGCCTCATTTGATGTGAAAATGCTTGCTTATCTTTAAAAAACTTTGATGTAAAATGCTTAAATATCTTTAAGAAACTTTGATGAAAAATGCTTAAATATCTTTAAGAAACTTTGATGTAAAATGCATGCATATCTTTGAGAAACTTTGTTTGATGTAAAATACTTGCTTATCTTTAAGAAACTTTGATTTAAAATGCTTGCATATTTTTAAGAAACTTCGTTTGATATGAAAGTGCTCACATATCTTTAAGAAACTTTATTTGGAGTGAAAATGATTGCATATCTTTAAGAAGAGTTGTTTTGATGTGAAAATGCTTGAATATCTTTAGAAACTTTGACTGATGTTTAAATGGTTGCATATCTTTTAAGAAACTTTGCTTAAGTGTGCAGATGCTTGCATATCTTTGAGAAACTTTGTTTGAAGGGAAACTGCTTGCATATCTTTGAGAAACTTTGTTTGATGTGAAAATGCTTGCATTTCTTGAAGGAACTTTCATGTGAAAATGCTTGCATATCTTAAGGAAACTTTGTTTGATGCACAATGCTTGCATATCTTTAAGAAATAAAAGATTTTTGACCGCT

>chr39:+:11933356-11934874

GGCTTATTTGAATTTTTTACTGACACTGAATACTCTCAAGTTGGTAAATAGTAAAAAGGTGATCAGTGCACAGATATGATACATGCCTTCAAAGTGTTTCACTGAGTTGGTAGTCTGTTCACCAAATGTTGGACTGAAAGAGCTACACCTTAGATAAGCCAATACACAGTGCTGTACTCCTTTCTTGTAACATGCAATTATTTTCTTCATTATTTGAACTCCACCACTTTTCTTGAGTACTATACCCCTTTGGAGGTTTTGACAGCATGTGTGTTAGTGATTTTGTCTTTTCTCACTAAGTAGGTCTCACTTTCTCTTACATGGTATTGTTATTTCATTAGAGGAACCCCAGTTACGTACTCTATCGACATGAAATTTGACATTACAGGTACAACAGACTGAAGTGCTAGTCTCTAGTTAATGGAAAACAATCCTAATTAAAATAATGGTTAATTGAGATGTTAGTAAAAGTTAAACTATTACTGCCTGAGGTTCTCCCAAACAGGAAACAATTCACCAGAACTTATTCATTTCTGTACAGAATTATGTCTTCTTGTTGGTTACAGCACAGTGATCCAGAACTAACTCCTCAGAATGTTGTGCATAAGAGATTGCAGATTCTAAAGCAGTCCCAAAGTTTCCTGAATTTGAAAACTACAATACCTGATATTTTCAGTTAGACCCATATAACATACAAGTTGATTAGGCAGTATAACATTAGTTGGCCATTAAGTTTACCTGGTCAGCCAACATACAGGAATTCTATAGTAGTTTAATGCCTATATAATTTGATAAAGTACAACCATTCAAAAAATGCCTTGTAAAAATAAAAGGAAACTTGCTTGAAAAATTATTGACCAAGAACACTGAAATAGTTCAGGTAACAGAGGATGATATCCTGATGCAAAACTCATTTTTACTTAGTGTTTCATATGCTGCATAATATATGGCAGTCAGTGGTACCTTCAAATTTAAATCTCTCTAAAGGCTAGCAAAAATATGCATGTATCATACAGACACGTACCGACTTATAACCAAAGAACAAGTAAATAGGTAATCGGACACTTCACTACAGTTACAGTTGGATGCATTTATGTATATGAAAAAGAATAATTTCAGAGTAGTTTGTGAAATGCTTAAAGCGTAGAGTATTAACTCTCATGCATTTTGTTAAATGCTCCTTCTTTCGGGACATTATTATTTTTTTTTTTCAATTTGCTGTATGTTTGTTATTTGCAGATTTTTATATCTTCTTTCTTGACTTGATTCACATTCTTTAGTTCTGTCAGTTCTAACTAACTTATGTAAAGTATTCTTTTTATTTTTTTGAATGATTTATCTTTCATTGTTCAACTTCTGTCATTGACAGGTGACACTTGTAGAAATATTTATAGTTATTGTGGCACTGTCTATCTGTTTCAGATCTTGTTAAGTTTATATGGAAGTATTTCATTTTGTGACATGTTTTAGCAATGCAAATGGGTATGAAAGTGAGAAATAAAAATTAGTACTTCGTTTC

>chr12:-:85471155-85471319

ACTGCGGTCGTGACTGTGAACCTGACGACGACGAAACCGTCGATACTACTCAAGGTGTTGTGTCTCCGGCACGCAGATAAATTTCTTTCCTCTATCGTTAAATATATTTAGTTTCTAATACAATGCCTCGCCGCGGAGGATTTGCCCCAAGAGCTCCACCGTAAG

>chr12:-:85467955-85467963

CCACCCCCC

>chr12:-:85467692-85467706

GCTGTTGGCTTGGCT

>chr12:-:85464701-85464703

GTG

>chr12:-:85458532-85458663

GGTCACGTAGTAGGTCATGCACTTACTAGTGGAGGAAGCAGCTCTGAACCCCAACCCCAAGCTGCCCCTGCACCAGTTGCCCAACCTCAGGGATATCCACAGTACCAAGGTTACCAACAGTACCCTCAACAG

>chr12:-:85406180-85406318

AGTGGGCCCAGTGAACCTCAAGGACCTTGTGCCTGGGAAATTAAACAGTTCTTGCAATGTGCCCAAACACAGTCTGATGTTTCTGTATGTGAAGGCTTTAATGAAGCATTAAGGCAATGCAAAGCACAGCACCAGAATC

>chr12:-:85373109-85373480

AAATGATGGTTTAGTGTACTCCAGGAAATGGATGGCAGGGAGTCGTGATGTGTTTTGTATAGTACTGATGATATAAATTGTAACTCAAAAATATCTGATAGTATAACAATGTCCATATTGTTTAGAGAATGGAATAACCTTGGCTCATATCTCATATTTTGTTTTATCCATCAAAAGTGCCCAGTATGTTTATGTAGTATATGCATACTGGTTTATATGTTTTGCTGATTCTGAGAATCCCTGTTGGCTTTTTTTCCTTTGTACATAATCAGCCGCATAAATATTACATTGTACTTCATAGGCCATTGCTGTTTTTAAGTTAAAAGTAATTTGTAAAATAACTGAACTGTTAATAAATATTTTATGTAATGT

>chr11:+:100375458-100375588

GGGAGTAGTCAGTTGGAATAATCATTAGCTGAGCCTGTGAGTGAGCCTTAAACATCTAGCAGTGTTGATAAAAGAAGTTTCAGACAAAAGGAACTTTCTATCGGCAAGCATGGGGAAGGAGGAGAAGTCAA

>chr11:+:100384414-100386055

AGCATGTTGTCTTATCGTCAACTTGTGGAGGAATTTGCTTTCTTCTTCACTGGAAACTTCGAGAAGACAATCTCTCCAGAACAGTATTATTATCATCATTATTCAGAAGATGGAGAACCATATTCATATGGAACAAGCCCATCAAAGGGGCCATTGACTTGGAATTCGAGCTTCCAAAGATTTTGGCGTTCATTAAAAAAAATGAGATGGAGATACACAGCTCCAGAAAATAAAGGAAGCCAAGTACAAGGATATATGTAGAAACAGTTAGAGGAGCTGGACACCAAGATGAAGAAAGGATGCGAGAATGGAGGTAAAGTAAAAGGCCAAAAAGGGGTTGCAGAGCTAAGGGTATGAAGTTCTGGACACACAAAGCAATTGTTTAAAGCTAAAGACTGATTTTGTTAAGGAATCTCGCTGATTCCTTCATAAAAAAAACGGTAATTATTAAGATCTTAATAACGAATATAAAAAGTCTTAATTTTCTACTTGGGGAAAGTTACTTGATTCATCGGTAAACTAACTTTCTCCGAAAATAACGCTGTGAATATTAGTTTTTGTCGCTTCTAATTCGTAACTATCCTTGGAGAAGTTGGCTCAGAGAGAAATTGTTCCTATTTGGAGAAGTTGGTTCAGAGAGAAATTGTTTTCTTTTAGGAGAAGTTGGTTCAGAGAAATTGTTTTCTCTTTGAGAAGTTGGCTCAGAGTGATATTATCTCTTTGGAGAAGATGGTTCAGAAAGAAATTGTTTCCTCTTTGGAGAAGTAGCTTCAGAGTAATTTTTATCTCTTTGGAGAAGTTGATTAAGAGAGAACTTGCTTTCTCTTTGAAGAAGTTGCTCCAGAGAGAACGTGTTTTCTTTTAGGAGAAGTTGGTTCAAAAAGAAAGTTTTCTTTTAGGAGAAGATGGTTCAGAGAGAATATTTTTCTCTTTGGAGAAGTTGGTTGAGAGAGATTTTTTCTTTCTTTTTGGAGAAGTTGGCCTCAGAGATTTTTTTTTTTTTTGAGAAGTTGGTTCAGAAAGGACTTGTTTTCTCTTTGGAGAAGTTGGTTTAGAGAGAAACTTTTCTTTTCAGAGAAGTTTGTTCAGAGAGAAATTGTTTTCTCTTTGGAGAAGTTGGTTCAGAGTAATTTTTATCTCTTTGGAAAAGTTGATTAAGAGAGAACTTTTTTTCTTTTCGGAGAAGTTTGTTCAGAGAGAAATTTCTCTTTGGAGAAGTTGGTTAATAGATAGAGAACTTGTTTTCTTTTCGCAGAAGTTGGTTCAGAGAGAAATTGTTTTCTCTTTGGAGAAGTTGGTTCAGAGAGAATTGTTTTCTCTTTGGAGGAGTTGCTTCAGAAAGAACTTTTTTTCTTTTAGGAGAAGATCGTTCAGAAAGAACTTGTTTTCTCTTTGGAAAAGTTGTTTCAGAGAGAAACTGTTTTCTCTTTGGAGAAGATCGTTCAGTTACAAGTGATAAAATTCTTATCTGATGAATGATGGCCGGTCCAAAAATATGATCATTTATTACTTACGAAGGCATTACTTTATTGCACAGTCATGGACAACAGATAAGAGTATATCAGTTTATCACAAACAAACAAAAAGCAAGCCCACAAAGACACGTCAGCTTTGAGATAATAAATACATGTTTGAGCCACGT

>chr11:+:100375458-100375588

GGGAGTAGTCAGTTGGAATAATCATTAGCTGAGCCTGTGAGTGAGCCTTAAACATCTAGCAGTGTTGATAAAAGAAGTTTCAGACAAAAGGAACTTTCTATCGGCAAGCATGGGGAAGGAGGAGAAGTCAA

>chr11:+:100384414-100385104

AGCATGTTGTCTTATCGTCAACTTGTGGAGGAATTTGCTTTCTTCTTCACTGGAAACTTCGAGAAGACAATCTCTCCAGAACAGTATTATTATCATCATTATTCAGAAGATGGAGAACCATATTCATATGGAACAAGCCCATCAAAGGGGCCATTGACTTGGAATTCGAGCTTCCAAAGATTTTGGCGTTCATTAAAAAAAATGAGATGGAGATACACAGCTCCAGAAAATAAAGGAAGCCAAGTACAAGGATATATGTAGAAACAGTTAGAGGAGCTGGACACCAAGATGAAGAAAGGATGCGAGAATGGAGGTAAAGTAAAAGGCCAAAAAGGGGTTGCAGAGCTAAGGGTATGAAGTTCTGGACACACAAAGCAATTGTTTAAAGCTAAAGACTGATTTTGTTAAGGAATCTCGCTGATTCCTTCATAAAAAAAACGGTAATTATTAAGATCTTAATAACGAATATAAAAAGTCTTAATTTTCTACTTGGGGAAAGTTACTTGATTCATCGGTAAACTAACTTTCTCCGAAAATAACGCTGTGAATATTAGTTTTTGTCGCTTCTAATTCGTAACTATCCTTGGAGAAGTTGGCTCAGAGAGAAATTGTTCCTATTTGGAGAAGTTGGTTCAGAGAGAAATTGTTTTCTTTTAGGAGAAGTTGGTTCAGAGAAATTGTTTTCTCTTTG

>chr11:+:100385173-100385342

GAGAAGTAGCTTCAGAGTAATTTTTATCTCTTTGGAGAAGTTGATTAAGAGAGAACTTGCTTTCTCTTTGAAGAAGTTGCTCCAGAGAGAACGTGTTTTCTTTTAGGAGAAGTTGGTTCAAAAAGAAAGTTTTCTTTTAGGAGAAGATGGTTCAGAGAGAATATTTTTCT

>chr11:+:100385403-100386054

ATTTTTTTTTTTTTTGAGAAGTTGGTTCAGAAAGGACTTGTTTTCTCTTTGGAGAAGTTGGTTTAGAGAGAAACTTTTCTTTTCAGAGAAGTTTGTTCAGAGAGAAATTGTTTTCTCTTTGGAGAAGTTGGTTCAGAGTAATTTTTATCTCTTTGGAAAAGTTGATTAAGAGAGAACTTTTTTTCTTTTCGGAGAAGTTTGTTCAGAGAGAAATTTCTCTTTGGAGAAGTTGGTTAATAGATAGAGAACTTGTTTTCTTTTCGCAGAAGTTGGTTCAGAGAGAAATTGTTTTCTCTTTGGAGAAGTTGGTTCAGAGAGAATTGTTTTCTCTTTGGAGGAGTTGCTTCAGAAAGAACTTTTTTTCTTTTAGGAGAAGATCGTTCAGAAAGAACTTGTTTTCTCTTTGGAAAAGTTGTTTCAGAGAGAAACTGTTTTCTCTTTGGAGAAGATCGTTCAGTTACAAGTGATAAAATTCTTATCTGATGAATGATGGCCGGTCCAAAAATATGATCATTTATTACTTACGAAGGCATTACTTTATTGCACAGTCATGGACAACAGATAAGAGTATATCAGTTTATCACAAACAAACAAAAAGCAAGCCCACAAAGACACGTCAGCTTTGAGATAATAAATACATGTTTGAGCCACG

>chr10:-:26579958-26580005

ACTACCAGGCATTTCTACTAGCTGAGCAGACCACTCGCGGAGTATTTT

>chr10:-:26575991-26576165

ATATAAAATGGCTGAAGATAATGCAACTGATACCCCCAAGCGCAAGAGGGGAAGGCCCGCAAAGCCTGAAAGTGAGAAGAAACAAAAGGTTGTAGCCGAGGGCTCAGATGCAAAGAGAGGCCGAGGCCGACCCAAGGGCAGCAAAAATAAGGCTAAGAAGTTGGGAGTAACAAAG

>chr10:-:26575143-26575811

GGAAAGCGTGGTCGTCAGGCAAAGAAAGAGTCTGCATCTGAGGAGTCTGCTGAAGATGCAGAGTAAATTTAATCATTTCATTCATGGAGCGATCAAGTTGCCATCACCCCTTGGCTGACCTACCTTCTTGAGATGTCATTACTGAAGGCTGGTTATTACCAGCTCTGGGCTTTGTAAAATATCCTTGATATTTTTTTATTATGATGCATGCAGGATAACTGCAGTCTGCTCATCCCTCAAGCCAGGTTTATTAATGCTGTACCTTGAACTTCCATAGTCAAAATTATTTATTTTTCTATATATTAATATTGGTATAGATTTGATGTCTCGAGGAGAAAGGATTTCAGACAGGGTTAGATGGTGAAGTGGTTAGGTGTTTTTTATGTGTGGTATTGTAGGTGTAACATTGTGTGAGAAATTCATTATCATTCTCTATTCAGAGTACAAAATGTGTAGCGAGTTAAAGTTGTGTTTTATTTCGTTCACATAAGATGGGAAGAAAGATCTGATGTCCCTTTTTCATCTTCATTCATATACTGTATTAGTGCATTCAAAATGGGCCTCGTGTCCTCGAGCACTTTTTCATTTAGGGACCAACCCCAGGTCATAGCATTGGGGTAAGCATCTTAAACGTGCGATTTTTAATTATTATGGAATAAAATTTTAGTT

>chr4:-:10223279-10224470

CGGCGTGTCTCTCTCAAGCCAATAGTAATAATGCTGCTGATGCTCACTTTAGATTGGGCTACATAACCCTCTCTGCCACATTATATTTCTCTCTTTCCAACATATTCTTTCTATCCAGGCAGGATGCATTGTCACTACTTTTTGGAATCTCAATCGATACCAAATTTCGTCACAATACAAAAAAAAAAACTTAAGGGAACGGCCTCGTCGTAGTAACGAGGCCGAAACTCCCCAACTCGCTGCAGTTTGTCATCACTAGAGAAAAGAACATTATGTACAATTTCATTTTCATCCCTAACAAGGATGTGTACCAACCGCTCGCTTCCTGGCGGCCGCTGGCTGGCTGGCTATAACCATTTTTCTCTCTCAAACATTATATCTTTCTTTCTTTGTCGTACAATTTTCTCCTTTCACAGTCTGACAAAACACACAGATGGATGCTGCATTTCTTTGCAGACACTCGTCCGGGAGTGGCGACAACACTGGGCAGTGCCGTCGCCGCAATGTGCGTTCAACTGGTCGATGCATCTCATAATCTTGCGATTCACATGCTGACACGCATTTTGCTGCGGTCTTCATCGACCCACGAGCCGAGCGATCCACCATTAGAAGTTGTCAAATTAGTTAGATTTCGTGCTGAAATACTAGATTACAATTTGCAAACACAAAATGTAGGATGGGCATTCTCCGCATCCCCAAAAGCCACTTGCTGCTCAAAAACAGTCAAGTCAGTCACATATTTCTCTCCAGACTAAATTCTCTCATCTCTAAATGCAATCAATGTTCAATTAATAATTCTCACACAGACAGACAGACACGCAGCATGCACTTGAATCTTTGCTTTCCTTTAAACCATTGACATGCACACTCACACAGCCCCTCTTCAAGAGCACCGTATAGAGTATACACTTGACTCTCACTTTTTTTCATTTTCATCCCTAACAAGGATGATGATACATTCAAAATCCAATATTCTTCATCCCTAACTAAGGATGGATGGATGATTGCTGCTGCTGCTGCTGCTGCTGCAGCTGCACTAAAAACAGTAACTGAACATTTTTCTAAGCAAAGTGCTGATGCCAGGCCAACCAATACCAGTTTCCGTCATCTCCAACCCTCACAAAAGCCCCTCTTCAAGAGCACTCTTGTGAGTTGAGCTTGTTATAATATAATGGCTCTCTCACCATAAAAC

>chr4:-:10222559-10222822

ATTGGCTCTTCGCTTTCGAGACGTCCCACACTGATACCAGTCCACCACATCCAACACAACAAACTATACTAGACACTATCTCCCTCCACGGACTATTACTACCTTCCTCCATCTACACCTCTAAATATCTCTCTCTCGCTCTCCAATCACAGTCCAACGTCCTCTCCAACTATGGCCATCGACAAACAGTCACACAACCATTTGAGCTTGATGGTTTGCTCTCACACCAACCATCATCGGCTCAGCAGTAGCTTTCGAGACGTT

>chr4:-:10222455-10224079

GTCGTACAATTTTCTCCTTTCACAGTCTGACAAAACACACAGATGGATGCTGCATTTCTTTGCAGACACTCGTCCGGGAGTGGCGACAACACTGGGCAGTGCCGTCGCCGCAATGTGCGTTCAACTGGTCGATGCATCTCATAATCTTGCGATTCACATGCTGACACGCATTTTGCTGCGGTCTTCATCGACCCACGAGCCGAGCGATCCACCATTAGAAGTTGTCAAATTAGTTAGATTTCGTGCTGAAATACTAGATTACAATTTGCAAACACAAAATGTAGGATGGGCATTCTCCGCATCCCCAAAAGCCACTTGCTGCTCAAAAACAGTCAAGTCAGTCACATATTTCTCTCCAGACTAAATTCTCTCATCTCTAAATGCAATCAATGTTCAATTAATAATTCTCACACAGACAGACAGACACGCAGCATGCACTTGAATCTTTGCTTTCCTTTAAACCATTGACATGCACACTCACACAGCCCCTCTTCAAGAGCACCGTATAGAGTATACACTTGACTCTCACTTTTTTTCATTTTCATCCCTAACAAGGATGATGATACATTCAAAATCCAATATTCTTCATCCCTAACTAAGGATGGATGGATGATTGCTGCTGCTGCTGCTGCTGCTGCAGCTGCACTAAAAACAGTAACTGAACATTTTTCTAAGCAAAGTGCTGATGCCAGGCCAACCAATACCAGTTTCCGTCATCTCCAACCCTCACAAAAGCCCCTCTTCAAGAGCACTCTTGTGAGTTGAGCTTGTTATAATATAATGGCTCTCTCACCATAAAACATTGGCTCTTCGCTTTCGAGACGTCCCACACTGATACCAGTCCACCAACACCAACGCAATACCTCTGCGCATTTTCTCCATCTCTCTCTCTCTCTCTCTCTCTCTCTCTCATTTCTTGCAAAAGTGTCCCCACCATGTATACTGTGTGACAGATCCATTGCCTTGTTAAACCAATCAGAGTTGTTTGCATTTTTTTCCTCGCATACTAACAAGAGCCCCTCTTCAAGAGCACCCTTGTTGTGTGTTGCTTAGACCATATTATACTGGCTCTCTCTCTCTCGCCATCAACTCTAAGAACTTTACACAAAACAACCAATCACACAAAATTATCATTGTGCTGCAATCAGGCTAACCAATACCAGTTTCCGTCATCTCCAACCCTCACAAAAGCCCCTCTTCAAGAGCACTCTTGTGAGTTGAGCTTGTTATAATATAATGGCTCTCTCACCACAAAACATTGGCTCTTCGCTTTCGAGACGTCCCACACTGATACCAGTCCACCACATCCAACACAACAAACTATACTAGACACTATCTCCCTCCACGGACTATTACTACCTTCCTCCATCTACACCTCTAAATATCTCTCTCTCGCTCTCCAATCACAGTCCAACGTCCTCTCCAACTATGGCCATCGACAAACAGTCACACAACCATTTGAGCTTGATGGTTTGCTCTCACACCAACCATCATCGGCTCAGCAGTAGCTTTCGAGACGTTTTTCAAATATTATCAATTACACTCACTGACTGACAGACAGACTGACTGTTTCAAACAAACATTGTATATTTCTTTCTCTCTTTCAAATTTCTCTCTTTCAAAAT

>chr4:-:10220131-10220148

TTCTCTCTCTCTCTCTGT

>chr4:-:10205660-10205666

CTCTTTC

>chr4:-:10205237-10205239

AAA

>chr4:-:10201806-10201809

CTTT

>chr4:-:10195215-10195223

CTCTCTCTT

>chr4:-:10188949-10188952

TCAA

>chr4:-:10188611-10188618

AATCTCTC

>chr4:-:10182320-10182329

TCTTTATAAA

>chr4:-:10180643-10180654

TTTCTCTCTTTC

>chr4:-:10175830-10175835

AAAATT

>chr4:-:10169046-10169059

CATTTTTTTCAAAA

>chr4:-:10162787-10162793

TTTCTCT

>chr4:-:10156180-10156185

CTTTAT

>chr4:-:10155179-10155198

AAATTTCTCTCTGGATAATG

>chr4:-:10115964-10116126

TTCTCTCTCTCTCACTCGGTTTCCATTGGAATAAATTCAACATCAAAATCAAAGTTTTGGTTGCATGAGGTAGCCATCAGCGCTGGCGGTAACCGCAATACGCCTGCCAAAAATACATGTCATGTATATCTGGCACTGACTCCGCAACCTTGCTGATTTTTTC

>chr4:-:10115151-10116390

CTCTCAAAGGCTTTCCTTTACTTTCTAAATCATTTTTGCTTCTACTACCACCACCAAACCAATGTCATTCCAAAACCACTCAATTTCTATTTCAAATCAATATATCTCTCTCTCTCTCCACTTCATCCTCTCATTTTATATTGTGTCTCACACGAGTTCACAAGTCACTACTAGTTTGTTGGCATCAAACACAGTTTTACAAGTAAAACTCTGTTCATCCAATCTCTCTCTCTATATATATATATGTATATTCTCTCTCTCTAGTTCTCTCTCTCTCACTCGGTTTCCATTGGAATAAATTCAACATCAAAATCAAAGTTTTGGTTGCATGAGGTAGCCATCAGCGCTGGCGGTAACCGCAATACGCCTGCCAAAAATACATGTCATGTATATCTGGCACTGACTCCGCAACCTTGCTGATTTTTTCACATGTTACCCCACAACTGCCGTGTTCCAGTTGTCATGTGTACTCTACTATTGTGGTACTGCATCAACGGCAAATTGTCTGTCGGCCATCTCTCTCCAAGCCCAAAACAATGGAAGAGCATTATTACCAGCCTGCACAAGCAATATCTCCTTCTATATCTCTCTCTCTAACGAAAATCAGTCACTCACTGATGTGACTTCATTTCCAACATATTCTTTCTCACAAAACAATATTGCTTGCACAAACCAGTCACAAGAGGCTCAAAGGCTTCTGAGCAGTCTACCACCACCTTCCAGAGACAGTCATCACCAATAATGAACTGAACTCTTATCTATAATGGAAGGCCCCGTAACATTCAACCCTAGTGTTCGTTCGCCAAAACCATTCCAAAAACACTGGGCTTGAACAGTGCCGCGCGGCGGCTGTTCACCATCAACACCCGCCTATTATTAGTGCAGCATTGGTCATTTCCGTCTCTCCGACGCCCTCACCCATGCAAACTGTGGCACTCACCATCCTATGCATGGGATGAAAGGGTAAAAAGCTTGAGCTGACTTGTTCATATAAATATCACTGTAAATACATGACTCAAGAAGTCAGCAATCCATTGGCTCATATCCATCGAGACGTAAATTCACAATACACACTTTTTCTCAAACTTTGCAAATTCACTTTTCTATAGAACTTGTCTCATCTCTCTCACAAATCCTGCCCTTCTTTCCTCCTCACTGCATTCAACTTTCCACATCCAAAAAGCAAACCACCTCCCTCTCTCACTTTACTTTCTGTATAAAACTTGCTTCATCTCTCTCT

>chr4:-:10115065-10115097

CCCAAACTCTGCCATGCCTTCTCCTTCCAAAAC

>chr4:-:10114870-10114997

ATTTTCAAATCTCTCTCTTTTCACAAAACTGCCCACTTGTCTGACCACATCCAACTTTCCACATCCAAAAAAACCAGGGCATCTCCATCATACTCCTCTCCAACCATTACCAACTCTCACTGAAATAG

>chr4:-:10113208-10113250

GTATACCTATATCATATACTCTCTCACCAACCACCTGCCCCAT

>chr33:+:16741935-16743787

AGTGGCAATCTGGACTCGTTCCATGCGAGTCACGCGCTCTTGGAACAGCAGCAGCAGCAGCGCGGCAGAGATCGGCTCCATAGCCTTAGGAGAGACAAAAGCCCTCCAAAGCATCAGTAACCGTGTCGGACATCGAAAGGAACGGACGGAGGAGGAGGGCGTCTCTTGCTTCGTGTCGCCTCCAAGAGAGGGGGAGTTGCTCTCACGTATAGATATAATACAGAAAGACAGTGACGGTTAATCCTCCGAAGGGGGGGAGATTGAACGCCCTCGCCTCCCCGTGGACACGTTCCAACAGGCGTGGTGTCTCGTCCATCCATCCAGCCAGCCGCCCCCTGACTCTACTGCAGCAGCAGCAGCAGCAGCAGCGCCCTTCCACGCCCCTCCCCATTGCCTGGTGTTATCGCCATGACGGAGGGGGCGTGGCCGTTAGGGAGGTAGAGGAAAGGAGAGGAGGAGGAGGAGTAGGAGTAGGAGGTAGTGCAGAAGAGACACGGTGGAGGAGGTTGGTGCGAGCAGAGGCCATGTGCCTGGAATCTACCGCAGCGTAATTGACAACAAGAACAACAACAGCTGGAGGGAAATAAATAATCATCTTGCACTGTCGAGGAGCGTATGGTGTACTGTAAACAAATTGACTCGGTGCTCGGAATGGGTTTTGCTGATGTCTCGTCCTTTGGAGGAATGCGCCCATGAAGAAGTCCCCCTCCCTCCCCATGGTTCTTTCATTCCCCAGGCAATTCCCATCTTCCAGCACACTCCATTTCCCTGCCTTCCCCCTTTCTCTTTCTCTCTCTCTCACTTCCTCATTCCAGTGAATAGGGGAGAGAGAGTTGGAATGTGTATTCGACGAGCCTCAGGAATCCATTAGGAATCAGAGACAGCTGTCGCATCTGCTGTTTAGCTGTGATGATGCCGTTTGTTGTGCCTGGATTGCGGTGTCGTGTCGTCATCGATTAGGAGAATCTCTCGTGAGTGTCTTGTCTGTCAGAGGCATTTTATCATCATCAGCTGTTGTTTTACTGCCAAAGCTGCTGCTTTCAGCCGTGTCGTTCCTTCCTTCGTGGCCTCGTTGATGGCAAGAAGCGGTTCGGGTATATCTAGGAAGAGTTTCTCAGTGGAAATGATCTATTTGTTCTCCGGGGTGCTTTGATACCTTGTGGCATCTGATTAGAGACATTGGAAATAACCAGAGAAAATAAATAGATTGTGGTACTGACATTTATCTGTCCCTGTGGAGAAAAGGACGAAGTATTTCGTGTTCTGTTGCTCCACAGACATGAATTGGAGGAACAGTTGAAGAAACAAAATAGATAAGAGGGTCCCCGTCCACGCCCCCTTTTTGACACCTCCAGAAGACCCGGGACGATGCATTGACGGCGTTCGTCGTATTTTTTTGCGCGAATCTTCCCAAAGTGAGGTGGAGGAGGGACGACTCTTATGTCCCCCTCTTTTCCCCTCTCTTGGCCTCACACCCAGCAACCACGAAGGTCATCGTCCCCTCCTTCATGTTCGCCGCCGTAGCGGGCACGGTGTGGCATGTGATGCGACGTAGGATCGCCAGCGAAGGGCACCACCCTCTCCAGCAGCAGGACATGGTCGAACTGAGTAGGAACTGCTGCAAAAGTAGGGGCGGACCTTGTGGATGTAAGGTGGTTGGAGGCAAGGCCACCTCGTCGTCGTCGTCGTTTTGCCCGTGCCCATGTTGGACTTGTCAGACCTTCATTGATTTCGAGTGCGTCCGCGTCTGTTGGAACTGGTAGGAGGGAAGGAAGGAAGGAAGGAAGGAAAGAGAGAAGGAGTGGGAGGTGTGTTGTGTGAAGTTGGAAGGAAGGGGTCGATCAATATATCCT

>chr33:+:27510948-27513099

AAGTTTTGGTGATATTTATTATGAAAACGTTTATACAAGCTATAAAAATTAATGCAAAAATATGTGGATGGCAGCTACTCGGACAAACATTACGAGATCGGCAAACGATACATTCAGACACTAAAAAAAGAACAAAAAAAGGTACAACAGAATGAAAGGAAGCAGATGGGTTTCTGAAGAAGTGCTGAAAAATTTCCCTTAAAAAAAAAAATTAAAAAACACTCACATTCCATAAAACTCAAATCAAGGTATATGAGAAAAACTCCACTTACTGCAGTCAATCTCTCGACTACAATAACTTTTCTTCAGGAATCACAGTGAAATGGAGAAACTGAAAAGCAAAAGGACACGAATCTCTGCACCTATAGAAAGCAGTCAGTAAGTTGCTGTGACTAATAAATCTTACGTAAATCTATAAACCAATGTTGGACTATCTTTACAGACTATTGTTATTACAAATTAGTGCTACTGTCACCATATAAAACCCTTCTACCGTTCTATTCTAGTAAGAAAAGGATAATGCATCCAGTGCATTTGTGTCTTCTGTAAAGAATGAGTAGTACACCATCGTCACGAGAAGCCTTGCATAAATGGATTCCTATAGGTAGGTTCATTCCAACAAAAACTCCATAGCTATGCAGCACTGCTGTCTCATAGAGGAAGGCTGAGTGGAAAAAAGGTTTTGGATAATGACAATTATAGTATTGATATCCTATGGCAGTGATAATAAACATAAAACCAAACTACGACCAAATTGGACACAGCATCTCCGTTCGCCTAGAATTATTTGGCAAGGTCTGTCGTAATAATGCCTTTCCAACTTATCTTCATCATGGCATGCCAATTAGTGACATAATTTCATAAATATTTTCTTTCCAGTCTTACATAATAGTCCAAGAGGTTCAAGCTGACAATGATACATTAATTTATTGTTTCCTCTTTCCCACACAATAATTTGGTCACCTTAGAAAGTATACAACTTGTCAAAATGACAATAAAAGAAAATCAGTATAAACAATCAGTTCACATTACTGTATATTTATAAAAGCTACATGCTTCTTTTTTCCCTTACACTGTACTTATCAGGTGCCTCACTGGTCAGCAAAAACAATGAACTGTCTTGGTGTGGTTCACGTTTCACTGCTGGCCAATGAAAATGCATTTTCATGGTAAGTTATAATGTAACAAGTAAAAGTCTCAGTTAAAGATATGGATTTAACATACAAACAGTGACAGAACTTAATCCTTACTCTCACTGCAAAATCATGAATTTTTCAGCAATTACAAAACAAAGGGAATGCATAATAGTATTAATATTTCATAATTTTGTACTGTGATCTGACGACAAATTTTTTTTGAGAATTACAGAATATATTAAGTATAATATCCACTATTATCCAAACAATTTTCAAAATGTTTAAATGCAATAATGACTGATTAACATCACTTCAATGTAGCATTTGCAACATGAATGCCATGACCACACATTAACTGGTATAAGTGGTGTACGAGCTAAAACATTGTAAAACATTACAAACTTAAATTACAATAAATATCACTAGTTTATCCTTTTGCTAAGCAGGAGATACCATGTCCCAGGACTAGAAATAAATCATAATTTTTACACAGATGAAGATCATATACATAACAGGTCCTATTTCAGTCGTGTTTACACTGAAAATAAAATATAGTGGCCGTTGATGGAACATTCATTATAATCAGTGCTCGGATGATACACTACAATAAACAATATTACATAACAAATAAATAAATTACAGTATTTCTTTACTTGTTTTTGTGAGTGGCGATGATTTTCAATAAAGTGCTTTCGCTGTAGAGACTTAGGATTTTTGCTTGTGTAAGGAATGTTGTTTCAATTATTTTGTAGAGAATGTTACTGGTTCTTGGAAAGGAATTCATCAATAAAAAAATACAAAATATGGGCCTGTCTATGAGATACTTAATTGTAAGGTAAACCCATTCTGTCATTACACAACTAAATAAACTTGGAAACGTCATAACAACTGATCTTCAACTGAATAAAGAAGTTAACTATAAATAAACAATTATACAATTATTCTACTACGGTACTGGACACCACAAACATACTGAGAGAAATAAGCCGTTAGAAAGTTCAGTAATTATAATTGAC

>chr33:+:44137392-44138976

ACAATGCCAACGGATGGAATTTTCGTTCTGGAATATTATTTTTTAGAAGTACTATGGTTTTCAGTGTCCACGGATGTTTGCTCCTCTTCGCTAAAATACCAAATGAGTCTGTCAGTACGCCACCACTCATTTATTTCTATTTCTGCGTCATATACTGCAGCAGCTATCAGTGTAAACAACTAATTATGCCCGGTTCAAGGTTGTCCGTCAATCAGTGTATCTAGTCAGTCATCCAGATCCATTTTACTGATTCGTTCCAGAATCTCCTGAGATGATGGTAGCTTCTTCATCATCTTGGCCAGGGTGGCATCGTCCACCTCATCATCCAGGTCCAGCTTTTCCGTAGCGGTAATGACCTTCCTGCTCCTGACGGGGCTACTGGAGGACGCAGTTGTCGCTGACAGTTTGGAGTACCGTTCCGTTTTTTTTTCGGTCTTGGACGCCGAGAAGTCGTCGTCCAAGTCCTTCCTGGCGGAGAAGGTCTTTCGCTCCATCTTGGGGGTAACGTCGAGTTCCACTCGCTTCCTTTCGAAAGTTGGGGTTCGTTTGTAATCGTCGATTGACGAGGAGGAAGCTCCGATGCCATTGACCTTAGCTGACCTTTCCGACTTGAAGCTGTAAGAGGACTCGTCACTGGTTGCTCCGGATGCAGCGGCTGACCTGTAGCGAGATGTCCTTTCGGACTTGTAGGAACTGTCCGTGTCGTTGGCGAGGGTGCGAGACATTGAGTCTCGGAGGGTGTTTCTCGAGGCCTCGGCGTCCTGTGGGAGTCCGAGATCCTCCCTCAGCCTCTTGAGCCTAGCGAGGCGTTCCTCCAGGCTCTGAGTCTCCTTCTGAGGGTCATAAGCGGGGACCGGTTTGGATTCGATTTTCAAGGTGGCGGGGAGCTCTTCCGAAGTCTCTCGAGCGAGAGAGGCCCTCGTGAGAATTCCCTCGACGGTAGAGGAAGTCTCATGGCGGCTCGAGAAAGGCTGAAGGGACTGCTCCCGACGGAGAGGCCCCGCTCGCAGGTGTGCGAATCGGTCAAGCTTGTCCTCAGACCAGTCCCTGGGACCTCGGAGCCTGTAATCCTTCTCGGCGAGATAAACCTTGTAGTTGTCTTGGTACATGCGCTCAGCATCCCTTAAATTACACTCATAGACCCTAGGCTTAGGTCTGTTCCAATACCGGTTGACCTTTATAGGCCTCCTGTATTCTGGAACATCCATGATGACACCTTTTTATAATGGAGAGACGACGAAGACGGCGGCAGCGGCGGCGATGAAGAAGGGCGCGAAGGTGGGAATGGCAGAGAGGCTAAAGAACGGCTCCTGCTGCTGCTGCCACGGCTGCTGGTGGTGGTGCCTGTAATAGAGAGACACACTCGAGTGGGCGGCCTGCAAAAAGGCGTTGGGGCAAGAGAATGGCGGAGGAGGAGGAGGAGGAGGCGCCGCTGCTGACCCCTTCTATCTCTGTCACTCACTCTCTCTCTCTCTCCCTCCTGAAGGGCTGGCTACCAGAGGTACGGTAGCAGAGGCACTACACAGCAGCTTCACAACCAGCGAACCTTCGTCGCCTACCACCACCACCACCTCCGAACGAGACT

>chr32:+:22037692-22040025

GGTTTGCAGTACTAGCATACTCAGCACAATGGTCAACCAGACACATTCCAGGATAAAGGGTCTCTGGGTTCAGTGTTCATGTAAGAAGTGTCCTAGAAAACTCCAGTAGTGGCTGCTCAACAATTGGTGGAGCTCACCCAGTTCCCTGGGATAGTTTGTTTCTTACCAATAATGGTGGTATGAAAGCAAAGTTGATTGAGATGACTGGTCTCTTTCTTTATATCTATTCCATTTCTCCTCTATAGTGATGGGCAGTGAGAAGATGGATCCATCATGCACTGAAGCTGGAGCTGGTTAGGCCTAAGTGAGGATAGTAGCCTACTGTTAAGAGTATTTTATATTCTACACGGAATTATCTTTCAGCAGCAAACCTTCCTCTCTCTAGCTAGAGGAGAGAGGAAGTCTAACAAGCACAAATAGGTGACTACCTTAGTTTAACAGAATAGATATGGCTATCAACAGCTCCTCTGATCTAAGTTCTGACTTGTTCTTGGTTCTAACTTGGTCAAGAAGCAATGTTTAGGAGCTTCAGATGGTGTAGTTACACTACTTTTGTGAGGATTGGGAGATGTAAATAGTAGTTACTGCTGCTGCTACAATTACCAGTTGATACTCAACTTGAAGATAAATATTATGATGCTCAACTTGAAGATAAATATTGTGATACTCAACTTGAAGATAAATATTGTGGCTCTAATGATGGTTCTAGGAGCCTCAATATAAGTACAGGCACTCCTTTGATTTCAATGACACCAATGTAGTCTCAGGATCCGAGATAGTCTTTCTTCAGTGTGATAGTAGTGTCACCATAGTAGTGCCATGTTGCACAAAAGGGCCTAGTGTCCTTTCATCCCCTTTGTAGCAGTACAAGTACATGTAGAGACAGAAGTACACTAAGTACAGTGACCAACCAGACTTCATAGGGGACATGAATGTATCTACATCATTTTCTTTTTCAATTATGGTAAGTCATGAGATAAGGACAGCTGATCTTGTCTTTCGGATCTCGTTCAACCTCTGAAGAAACTACATGTTCAAAGGTGATCAATGGAATAAACCATGCTCCTATGGCCCTGTGGCACCTATGATAGTATGGCATCTATGTTGCCTCTGTAAGTAGTCTTTCAACTGAATACCAACAACTTGTTCGTTAGCTTAGAGTTGACTGGGAAATGATGTCCAATGGTCATTTCTCCGACTAGAAATATGATCAAACGATCATACCCCACAACTTCCTGGTGGACATCAGTACATTGTGCACAGATCTCCCAATCTATTGCTTGTTTGTATGGTGTTTCTATGTTGCATGGAACAAGTGGCTATTCAGCAACAGGACCAACGGCTTTACGTAACTTCTATCACCAGGAATACACATCTCTCACCCCTCAGTGGAACGCCCGAGAATCGAACTTGTGGCCAGCAATCTTGATCTATTGCATTCATCTCCTTTACCTTGGAACATTGCCCTTGAGTCCTTGAACTCATTTTCATTTGATCCCATGGTGGATGCTCAAGAAGTCGTGTAGTTCAACCAGCTCTTAGGGACAGGCTGTTTCTCGCCTAAGGTGGCAGCTGCAAGGAGAAAGTGTGTGGGACTGGCCTCTATGCTTTCTCCTAGCTTCCTTCCTCTTCCAGTGGACAGAGGAGCAGTGGACAAGCCGTCACATGCTGGACTGGCATGCATGTAGGTGATCTAGATGACTGAGTATCCTATCTATAATCCAGTTTTGATTGGATTAGTAAATGCAAATTCTTCATTCTCTCTAGCAAGGGGAGAGGGACTGACTATGGGCAAGCCAGTTGGTTATTTTAGCCTTATGGTCTCCCAACACAATGGGTTCATTCAAACCTTTTCGAATCAGGGATTTGACTTACATTCCCTTAATTCAAAATGCCTGGAATTCTGATGATTGAACATCTGTTGTTCAATACATCAGAGGTATTGTCTCTTGCCTTAGCTCTTTCATGAATAAGAAAAGAGTACCAAGGTAGGCCGAACTACCAGTCAGTTCAGAGAATTACTTAGAATCCTCCCTCCAAAAGTATGTCTCATATGTAAAGACCAAGGGTTTGTATTAGTGTAAAAACAAATGACATTTTTTAAATTTATATTTTTCTTAATATACCAAACTCAGGTCTTTATCCCTCATTATTTTACCTGGGCCAAAAGTAAATTAACTAGAACTGAATGCATTCAAGGCTGGATGGGCATTACTTCTGCCCCACCTTCGGTAACTGTTACCTCAACCGTTACCTTGCAAAAAAGCTTAACGGCCAGATTCCAACTCGCTGAAAGTTGACCCCATATGGAAAGACCTCAGGCTTGTATGTTAG

>chr32:+:32636424-32637817

GGGGGATGCTGGGAGTTCACGGATCGAGGTGTTGTTTTGTTTACAATCGTTACGCAGGCGCGCAAGCGCGAATTTCTTTCTTGCCGCACTAAAAAGTATCTGTGACACATCTCGGAAATTATTTCGTCACTTTGACATAATTTTTGTACCATTTTAAATTAGCCGTTACATGGAGTATTATATATGAAAATGTGCGCATTTTTATGTAGAATACAACAAAAAAATACTCATGATTGTAGCTGTTATCAGTTTTGAGATATTTTCATATAAATAATGATAAGTGCCAAAATTTCAACCTTCGGTCAACTTTGACTCTACCGAAATGGTCGAAAAACGCAATTGTAAGCTAAAACTCTTATATTTTAGTAATATTCAATCATTTACCTTAATTTTGCAACTAACTGGAAGTCTCTAGCACAATATTTCGATTTATGGTGAATTTATGAAAAAACTTTTTCCTTACGTCCGCGCGGTAACTCTTCCGAAAAAAATCATACATGCGATTGTGGTAATGTTTGCACCATTTTAAAATTAGCCGTTACATAAAGTTTTATATATGGAAATGTGCGCAATTTCATGCACAATACAACTAAAAACAACCCATGGTTGTAGTTTTATCAATTTTGAAATATTTTCATATAAAAAATGATGTGACAAATTTTCAACCTTCAGTCAAATTTGACTCTACCAAAATGGTCGAAAAACGCAATTGTAAGCTAAAACGCTTATATTTTAGAAATATTCAATCATTTACCTTCATTTTGCAACTAACTGGAAGTCTCTAGCACAATATTTCGATTTATGGTGAATTTATGAAAAAAATAACATTTTCTTTACGTCCGCGCAGTAATTCTTCCGAAAAAATCATATGTGCGATTGTGGTAATGTTTGCACCATTTTAAATTAGCCGTTACATAAAGTTTTTTATATGAAAATGTGCGCAATTTCATGTAGAATACAACAAAAAATAATTGAAGGTTGTAGCTTTTCTAATTTTTGAAATATTTGCATATAAATCACGATAAATAGAAAAAAAACCACGTTCGGTCAACTTTGACTCTACAGAAATGGTCGAAAAACGCAATTGTAAGCTAAAACTCTTACAGTCTAGTAATATTCAGTCATTTATCTTCATCTTGAAACAAATTCGAAGTCTCTAGCACAATATTTAGATTTATGGTGAATTTAAAAATAAAAACTTTCCTTCCCTCCGCGCGCGGATTCTCCGCCACAAATCTCCGAAATGCGTACGTCCCATTCTCGGAATATTTGCTCCATTTCATATTAGGCATTTCATAGAGTTTTATATATGAAAATGTGCGCAATTTCATGTAGAATAAAACGAAAAATATTTGAAGGTTGTAGCTTTTCTTATTTCCGAAATAATTGCATAT

>chr13:+:52062-53987

GGTGAAGCAGCAGGTGAAGTTGAAAAATGAGCAATAAGTTCCAAGAGGGGAAGAAATTTGTTGCCGATAGTAGTTCAAATCCTGGGGAGAATAAAATACAAGATGTGTACCTGTTGTGATTGGAAACCTGTAACCCCCTTTTTTTTGGTGTTTTGACTCATTTTTTCTAGTGTCATCACGAGGAGATCCTTGCTTTCTCAAAACTCTTTGAAATCGATTACCAATACCCCTAACATGCCCAAAGCTTTGCATGATGTTCTATTAGGCAATTTTTCTGGTCATGGTTCAATCCTTCTACATCAAAACAGAATCAAATGGAGTAAATCCAGAGTATTCCTTGAACTACAGGTTCCCCTTCTTTACAAATAACATTCCATAGGTAATGAATACCCAAAAAAATCTAGTAGGAAGATAGTTTTTACTAGTATGTGTCCTGTGACATACCTCTCCTGTGATATGATCTTCAACCGTTCTCTGATGAAAGGTATTTATGTACTATACTTTGAAAAATGAGAGTGAAACGTGAAAAAAAATAAGAATTAAACAGCAGAAAAATGCCAGATAATACACATTGCCCGAAATAACTACTGCAAATGAAAACTACTATAACAAAAATAAATCAAGTACTACTGTTTTCTCACTTCCAGTATCTTGACATAATGGTTCGGTTCCCTACAATACAACCAGCCTCACGCACACGACTTGGTTATTTTAGCATTATATTAACATTGGCGACTGGCAATATTGGCACGCTTTTAGAAGACTAACTTGAATACTGATGGGATTTTTTTCTAGAGTAAAGCAACTCAAACTAATTTGACGTAGGTAACATAACTTTATTGGAAATCAAAATTTTCAGCAACCACATTTGCTAATACATTAGTAAATATGCATATGAGGAATAGAAGAGATCTGACATATTGCTCATGCTAAGTTGAGAGCAGTGACATATATGGAGGAAAACATGTGATCATAATAATGGTAACTCATTTTAGCATGCTTTGTAAAAGTGCTAAAATATGCTGCTATACTAGTGAGGTACTTCAAAGGTCATCATGATCAGTATAATTCTTTTTAAAAAAATATTCAGTACAACTTTTTAAGGAAAAGGGACATTACAATAACATCAAATTACAAATACTGTTGTAACATCAAATTTTGGAAAGAAAACTACAATGTCATTAATGTATGTTTTGGTTGTGTGGATAGAAAACAGTCAATTAAGAGACACAATCAAATGGAAAAATCAACAGAGTAAGTCTATAAGTAGACCTGCACTACTCTCTTCCTAACAGATCAAATTATACTGTAGTGGTAAAGAAGCTGCCACCATGGAAGGTTTCACTGAAGGCCAGCCACAATTCATTTCTCCATAACTCAAGACAAGCTTAAAATTTCATTGCAAACCTTTCGTAATGCAATTACAAGTGCATCAATAATGTAATGAACGTGCCTTTCTGCTCTACACTGTCGGATTATACTTCACATTTGCAGCCTTTCAATCACTCTCACACAATATATCCAAAAACACAGTGAAGTCCACTAACAGACAGCAATGAAAAGACAAGAAAATTTAAAAACATAATAAACATGTAAATTGGTAATATACACATTATCATGGAAGAGGTCTGACTGTACCAGCCAGTTAACGAAAATAAAATAGTTTAGCAAATATATCACAGGGTAAATGTACTTAACAGAGCACACAAACTCTCAGTAACATAATCTGAAGATGTATAAAGGAGATACAACTGACAGTTAACTTGACGACAAATAAATAAAAAAAAAGTAAATCATGTTCCTCAGTCCTTACACTTGAATCATTCTACCTAACAGCATTAGATGAATAAGACTGAAATCAAAACATATAAGCATTGCTAAAAATTGAAAAATAAAATATGCTAAAGCAAATTGCACTAGGAGGTT

>chr13:+:1994980-1996735

GGAGTGAAGGAAATTGGGAAGTCAAAAGGGGTGGGAAAAATAACAATGTACCAGATATCCTTCTTCGAGAACCATCACTACCCAAGCTTCTTCCTGAAGCTTTGCCAGACACTACAAAAGCTACGTACATGGCCTTTGGGGGACCCTTACAGCCTACTTGAACAGAGTTCAAAACATCAGATGTGATTACATTACAATTCTTTCACTTCAAAAGTAACCTTTGATCACTTACTGAGCTACTGCGAGCAGTTTGGGAAATTCTGTGTGGAACTTTCATGCAGATGTTGTAGAGGCAGGACCCTAGAGGGAAGACTTCAAAAGATACTGCAAAGAATATTCTATTCTGTTCTGATTCAAGACACCTTAGGCTAATGCCTTAAAAACTTGGGGATTAAGGAGACAATGGTAGGATGTGTCAAAAACAGCTACCAATGTTGAGCTGGATCACTTTCTTGGATAAATATGCGCACAACTGCTTCTGTTGTAATAAGAACCAACCCCTCCCTCTACTTTGGAAATGTTTATAAAGGTGAGGCTAAAATCTCCGATGCCTCATCAAGAAATGCTGCCCCGTCAGGACTTAACATTTGTATATGTTATCTAACATTGTGTACAAACACACACACACACACACGTTATTCTTGCTGTAATCAGGCGGTCATACTAAGAATGAAATAATGAGTTCAAAAGTACTTCTGCCTCACAATTAGGCCAAAGTGCTTGACTGATTAGAGAGGTCTTTGCTTCCTTGGAGCAAGAACTTGAAATCCATGTAGAACAAAGCAGCTTTATATAGCTGGAAACTGATGCAGTGGCATGCTGAGAACAAGCTTATGTGATACACTTCTAATATTGCCCAAGTTGCTGTTTTAGATTAAGCCAGCAGCATGCTGGCTCATTTGCCCTAGTTGGTTGGTTCGTATGCGCTCATTCCACAAACAGTTGCAGGCAAACTACATTGTTAATGTTAGGACAGAGCTATTCAACTTTACATGACATCACCATGAAACAAGGGTAACAGCCAGAGCCATCTGCATAGTACGTACTTTAAGAGGCAACTGCTGAGAAGCACGAGGCCATTCTAGTACAAGGTAGCTTAACCTAGATAAATAAGTGGTAGGAGAGGAAGAGCCGCTATTACAGATGAGAACATTTGTCAAAAGGAAACAAGTACGACGCAACGTGATAGAGGGGGAAACTAAGGCCACAATGGAAAACGATTCAGGGCACATAATCAACAAAGGAAGCATAGATGTGCAGCTCAGAAACTACTGGAGAAAGTGTAATACTATCTCAGAATGTAATCCCCTAGACAACTCCACTAGTCTACACACATCACTATCGCCTCCAAAAAAAAGGATGATACACGAGCCAGTAAATGTGACTGCTAGAAGACGGTTGAGAGGTGGCGAAGAGGACCCTTGGCCCCCGAGATATGCCTCGGTTCCCTGTCAGCTGAGAAGACCAATTGATGCATGGAAGTCCAGAGGGCAGGCTACAGACACAAAGGCTCTGACAGTTGAAATAGTTGATCACTTGGAAGGGGAACAGTGATTTTCACCCATAATATATACGGTCATATTTCTAACCTACAGAGGAATACTTATCATTTTTCTCCTGAACCACATTGGGAGCATAAGAAAGTGGGGCAGATTGAGAGAAAAGAATCAACCCATCATGCCAAGGAGGACCTTTTGCATCTTCCGATACTTCACAAAATGATTCATCCTCTCATCAGATCACTCCTTAATTTCTT

>chr13:+:9749551-9752195

GTGATGGCTGGGTTGGGGAGAGTATTTGTCGTTGGGGGTTGGGGGCAGGTCCATGGGGGATGTACTTGCATAAATGTTATGTTTATAATATATAATACTGGACGAATGTTATTTAGACTTTACCCCATTGATGTCCCTATTCTTGTACGTTTATTTTAACGTTTATATATTATTACAGCCTACATATCCGTTGTACATTACTGTAGATTCTTTCGTCAATAAATTATTAATTAACTAACTACGAAATTGAAGCCTCTCATATATAACAGGCGACAAGAAGGCGCATTATTGTAAATATCAACGCAATGCAAAAATAGATGAAGAGGTATTTATAGGTTCATGAGATTATTATGATTTATGAACATTTAGATGATGCTAAGCACTGGGGCACGATCAGCCATTCAACAGTGAAATGGGTCCCGGCAGAGGGATAGTGCCGTCAGTTCACCCCACACGGTGCACTGTAAGCATTACTCAAGGTTATTTGCGGCATCCCTTCAGTCCCTCGCTGCAGCCCCTCTCATTTCTTTTACTGTACCTCCGTTCATAGTATCTTTCACCCATCGTACTCTCCGCCCACTTCTATTAATTATTTCATAGTGCAACTGCGAAGTTTTCCTCCTGTTACACCTTTCTACTCTCAATTTCCCTTTCAGCGCTGCATGACCTTATAGGTCCCAGCGCTTGGCTTTTGGTCTAAATTTTATATTCCAATATTCCGACAGAGAAAAGGGAGTTTGAGTGGTTGGACAGCAGAGCTGGAGGAAAGGAAGCAATAAAAGGGGTTAAAGTAAAATTTAGTAATGCATACAGTGTACCTCGTGAGGTGCACTGACGGCACTAAATGTCTCCTCCACCGTAGGAGGATAGTGCCGTCAGTGGACCTCATGCGATGCACTGTAGGCATTAATTAAGGTTCTTTGCAGCGTCCCTTCGGCCCCTAGCTGCAACCACTTTCGTTCCTTTTACTATACCTCCTTTCGTATTCTCTTTCTTCATCTTACTTTCCCCCCTCTCCTAACAGTTGATTCATATTGCAACTGCGAGGTTTTCCTTCTGTTACACCTTTCAAACCTTTTACTGTCAATTTCCATTTCAGCGCTGAATGACCTCATAGGTCCCAGTGCTTGGCCTTTGGCGTAAATTCTACATTCAGTTCAACCCATGAAATATTGCGGGAATGATTCCTTGATAAAACACGGTGGTACTATTGATGTTAGTCAGAGACTATATACAAAACAGCTGCATGAATGTTTATACACAAGGCAGGGCCGGCTACGCGAATGTTTGGCAGAGAGGTCACGGCATGTGCGAAAATATTTATGATGATTACGCGAGTGTGTTCTGTGGAAGTTGAAAAACATTCTTACAAAACATCAGAGTAATTATTCTCATTTAGAATCAGGTGTACGTGAGTGAAATTCAGCAACATTATTAATATCCAACGATTCTCCCAGAAGACATGAACCCCATAGTGGTGTAGTGCTATCAGTGCTCCTCATGCGCACTGTGGGCCTTTCCTAACGTTCTTTGCAACGCCCCTTCGGTCCCTGACTGCAACCCCTTGCATTCATTTTACCGTACCTCCGTTCATGGTCTCTTCCATCTTACTTTACACTCTCCAATGCTGAATGACCTTATAAGTCCCAGCGCTTGGCCTTGGGCGTAAATTCTATATTCCAAATCAAATTCCAGAGGATACTGATTCTTCTTCAATTCACTCTACGATTATTCTTTTTAGTACAACATAAAAAAGATTTAGAAACAGTAAATTTTCTATTGCGAGAAAAAGATGTTAGAAAAAGTTTCAAACAAACTAAAGATAACCATTTCTTTAATAGCTAATGTGGCTATTTTATGGATGTTATCCTGCTTAGCAGATAAAGGCATTAATATATAATAATAGCTTGGAAAATGGTAAAAACATTAAACATGCATAATTGTCAACGAAAATAGGTCTGTGCTACATCAACGTCTAAAGGAACAAGTCACTGAAAGTAGGAAACTCAGTATCTGAATCCTATGTAATATTAATGTCACCCTTACAAGAACACGTATGTTGCCTAGATTCAAAGGCGCTTTAAATACCTAAATCCTATTGAATTTCAACGTCACAATTAAGGGAACACATACATGTAGGTTGCCTAAATTCTGACATTGAAAACCTCACGAAGTTACCAGCTTTCATCAGTATTATTTACTGCCTATACTGACGAAAGAACAAGAGAGTCGCAAATTAGATAATTTAACGCGAAATACACCTGTTATTGGTGGTTAATTGTCCGTCTTGAATTAATAGAAGTTTTAAAGGCTAACGGACAGAAAAGGACACCAATTTCATCTAGTTCAAGCTAATTAGTCACCTTGGATTGTAGACAACGGCATCTTCTTCTTCTTCTTCCTTTCGAAGCAACGAACGAAACTAAGGTGAGCAGTTGTCTAACTAGGTAAACAGTTGCCGAAACTAAGCTGAACAGCTGGCGAAACTAGGTGAACGGTTGCAGACACTACAATGAGCAGGCACCGAAGCTAGGTGAACAGTTGCCAAACGCCGCCACCACCAGCCAGCCAAGACCAGTAATTCACTAAGGTGTTCGAACTCCTTCCAGTGAGCTGTGTCTTTACCAAATCCCAAAAGCCGACTC

>chr13:+:33512451-33516071

GACACAGGATATATTTATGGAAAGTTTTACTCTATTACTATAACACTTGACTAGCTACATTGCTTACAATGTAATTCTGGATGTTTAAAAAGCAAGTAGACATCTTTGAATAACCACAGTGATGATATTGTGAACATCACCAATAAAACTCATTAATGCTTCTTTTAGCATTATTGGATAACACAGGGTACCTAGGTGTCATGTTTAAAAAGTATTATATAAGAATTCAGCTAATTACTACAGTCTGTAGGTCAATTAACAAGTTGTGTTATTGAAGAAAGATCTTATACTTCAGTAAAACATTAGGTCAAATAGGCATCATTAAATATGAAGCCAGTGATGAAACCTGAAGATAAAAGCATATGTTGAAATGGGTAAAAACTCTCTGATTTTGGATTTTTAATACTGTATCCAAATTAGTTTGCAAAAACATTATACAAATTTTCCAGTTCTCTTTGTATTTAATGATTCAGTTTTCATACATTCTTTTATGGCAACAGTTTGTAATCACTATTTATAATTCAAAGGTTGCAACTTCATACTGAGTAGTAGTAGTACTTGAGATAAGTATTAGAACTAAGAGTCATGCTGTCTGTCCGAAAATGTTATCAGTATAACTAAATTACAATAACAGAAAATTCTGCTATGTGAATGAAACTCTTTCAAGGTAAATTTCTGAAAATTAATAAAGTTTCATAATGCTGGTTGCTATACAAACAAGAGCAGTCTTAAGAAAACTTTTCCCATCTATGAGAGGCTTTCATGGGCCATTATTAGTCTTTAAAAATTACCTTGTATAAATTTATATTAATTACCTTTGTAAATGATTAAGTAGAACTCTGACAAATCCTTTTTTATAGTTTTGTTTTATCCAAAATTACAAAGATTTAGAGTTTTTTCCTTTACTCTTAATTACTTGGTAATATGCACAGGAACTGCCAAGGCTAGGCAGTAGTTCAAAAAACTTGTTCTTGTGCATGGGTAAGCTTTTTGTTGATCGCACATTTGCATGAATGCGTGAAAGAAATTTACATACAATTTCTGTATTCATTATAGCTACTATTGATTTATGAAGAAAATCTTTGAATTCCTATCATTTATTTCAGGTAAAAAATACATTCTAAATTTTCAAGAATTATGATTGGTAAGGTAAATTATAATGTAACCTGGGCTTCAGTAACAATATTGTTTTACTTGCACAATATGATGGAAAAATAAAAGTACATTCAACTTAAGGGAGCTTGCTAAGAAAAATTTATACTACCTTGAGTATCTTGTGGCTCATGTTATATAAATGCTGGAAAATATTGTACATGTAGAGTAACCTTTTTCTTATTCCTGGTATCTATGCTACTGTAAGTACTGCCATACCAATTACAAGGAGAACAAGATGTTTATGATGATGTTATTTTAAAAACCTATAGAGGGGGAAAATTTGGTTTATTTAGATAACACTATTTCTTGCAAAAACAATACGCAAATAAGTTTTACTTTTCACAAAAAATCGGCCTTAAACCAGAGACTAAAGCTTTGTGATAAACTAAATCTGCCAAAAGAATAGCCAGCATGTTGTAGTTTCACTGTAATTTATTATATTACCCATCAACATTGCAAAGGGTAGCTTATACTAAAAGGATTACTGAAAGTGATGTCGACTACATCTCTCAAGAGAATCAGTGTCTAGTAATAAAATTTAAAAGAATAGCTATCATTACATATTAAAGGAAAAACTACCCAAAGACAAATTTGAAAGCACTGAGATCATTAATCAATCTTCTGTTGTAGTATATTCCCAGACTTACATTGTAGAGCTTGTGTGCAAATTTGATGTTTTCTGTCCTTTGTGAAAACTTTATATTTTTATATGATTTTCAGATTTTTTAAAATCTGTGGAACAGAGAAGCCCGTGTGACAGAGCTTAAAATAATAACACTGCTCCTTTGTTTTTTAATCATACAATCTTCACACTGCTGTTTTTTCTTCGACTTCTCACACTTTTCTTCAAGTTCCTCATTTTATATTTTCCAATGCAATTTGTTAAGAATTTAATTATCTTGGGAGTTAACTTGCAAGAGAAGGGTTTAGGAATTTGTAAATTAATAAAAAAATTTATTTTGCTCTAGTCTGTATGATTGGTTGTGAGATGGAGATGTAAAAAATGAAATATATACTGTAGTAATATACAACCAATGAGTGTTTCATTTTCCATTTTAATTAGCTTTCTGGTTTGCTCAGCATTTTGCTTGTTCTCTCAGGTATTGAGGTATTACACTTATTACATTCACTGTAAAAATGTTTTTTGAGAAAAGAAGATACAAGCCACAGCCTTACATTTACCAATAGTGCATGCCCACCTATTTCATCCTGCCACTTCCCTGAACACTCTAGGGAGCCAAATTTTAATTTATGAAAAACGTTTGGTTAACAACTCCAACCACCCCGGTTTTTTGGCTAAAAATCTGACCGCAGAATTCTGTAGGGCCACAACCTGCCTCTATGACTTACTTTTCCAGCATTGTGTATTCTTCATACACTCTCTGAGAGTAAGAGGACATTAGTAGATAACAAAACTGAATTACAGGAACTGGAAGATAATTTTTCACCATGCAATCTCTCACAAAGAAACTTGAACATGGGCACTGAGGCCAAACCAAAAGAACCTAGGTCATAAGACAGTTTTAAAGAACAGAACAATATTACAAATTTGTCAAGTAATTCATACTTTTTCTAGGATACATGCCTGTGCTTCATAAATGGAGTTTCTTGCATCCCTTTCTGAGTCAAAAGCCCTTGTGATGATTTCCCTCAACCAGAAAAGAGATAATGTTCTTCAAGACCAACTCCGTTTTTTCCTTAATGACAAAGTTTCTTCTACACCTGGGTCTCAGGGAGGCTGTTCTTACATACAATCTTATGGCTCTCACACCAGGCACAGAAATACCTTGTCTCCCCTGAAAAAGTCCCTCAGGAGGGGAGGAGAACCTTTCAAACCGAGGTCGGGACAAAGGGGTCGTGGGTCTTGGCCAAAAACTGGTACAAACTGCAGGGACAGGAAGACCAGCCTTTAATGTGGGTAATTATGATTGAGAGGCCATGCAACTCCCTGACCCTCTTGGTAGATGGAAGTGCCAGGAGGAACACAGTCTTGACAGGTCTATTCCTCTCAGCCAGGTGCAAGGGATGCTCTATACACCTTGATGGCAGTTCTCAAAGGGCCTTATCAGACCTCAGGGAGTCTACTTTCTTCTTCACAGAAGTCTTCAAGTAGTCGTGACCCCTCTGACAACACCCATGACTACTCTGTTCACTTCCCCCAGTAGTGCCAATGAAAGAGGGTCTGAGAGTGTATGTGATGGCTGCTGCAAAATATCTTTCTTTTTTAATATGTGAGATCTCTCTACTTCGTCTTATCTCCTCTCTCTTCTTTCTAATGAACACCATAGTCTTTGGAATTTTGAATTTCAAATCTTGGCCCCTGTGGGCTTGTAACATATGAATAAGGGTCATATGAATAATAAATTAATAATATTAAAAGCTGCTGGATAGCCTCCAACCTTGAAGGGGGGGCAGCTACATTTAGATGATAACTTCAGCGCCAGAAGGCGGGGCCAGTTGAGGCTC

>chr13:+:35020867-35025088

ATATTCCTTTAATAATTTACAATGAATGTACTTATTCCAACATACACAAAATCATAAGAAATATATGAAAATTTAACTCCTTTATATAAACCTTATATCTTACATAAGCTACAGTACACATACTAAACATAAGAAATCTTGAAACACAACAATGTTAAAATGCAGGCCATTGTTTCACAAAAACTTAATTCTGTGGTACGGATGCATCAGTTGAGTAAATAAGAAGTACTAGGCAGTGGTACAGGATGTGGCATAAAAAGGAAGTCACTCTGAATAGGTTTACCTGCTACATCTCTGGTGTCTTGCTAGGGGCACTATTCCTATTTCTGTTTGGGAAAGGGCTTATGTGCTAAAAAAAAACGTACCATGATAATTTAAAATCATGATTCAATAATAAAATATACAGCCTTGTACTCTGTCCACTTAGGAACAATGATGAGGTATATTTTCTTTCATGCAAGTCTTTCACACAATGTATATGTGATAATAATGATGATAACAATGAGCAGATTCAATGATGATGCAACACCAACACTGTTATGCAGTTCTTGTATTCACAGTTATTACACTTACACATGAATATGTACACAAACTTTCACTAACTGGCAACATTATTGTACACAAACTATCACTAACTGGTAATATTATTTATAACAGTCTCCAGACATGGCATCAGATCATCAGATTCAACCTTGTCACAATGCTGCATAAAAGGAGAAAAAAAGTGGCAGTCCAGAAGCCTCTGTGATTTATGCTTGGGACTGGCTTTGTTTTTTCTTGCTTTTCTTGCTTTACATATTGATTTTTATAATGATATTGCCTCTGCCATTTATTAAACATTCAAGGTATGTAGCACTGCCAGGGCAGTAACATAAAAATAGTCCATGAAAGCAATGAAACGGCATAATCTCTCTACAATAATTATCCAGTGTACCCCAAAAGAGGCTAAAAGACATAAATGTACTTCATGTGGTGCACTGTAGGCAATAAATATCCAATCGAGATATGGAACCACTTTTTTAGCCTCTTTTAGTCTTTCCTCAATCTCAGTCAAACTTCTCAATACTCCTTGGAGTTGTCTCTCAGTTCCACCCTCGGATCCTTGTACTTCATCTATATTTTCTGGATCTCTTTGCCTCCCTGTCCAGCCACTCCAACTTCCTTTTTCATAGTCTTAAGTGGTGAATGGCTGAAAAAGTCAAAATGCTTGGCAGCCTAAATTTCATAAATCAAATCAGTAATACTATAACAAACTTGAAGTTTTTTGGGTCAGCTCTACTAGGTCATTTTCCATTACTCAGTTGTGTCAATTAGATCTTCAAACACTTCTCTATCCACCTTCCTTACCATGGTTACATTCTGGCAAAAGTTTTTCCTAGCATAAATTTCCTCTGGCTAAGTAGCTTTAGTGGTGCTTTAGATGATATTTATGACAATTTTATAATTTCCATACCATATTTAATGGGATTATCATTCTTAAATGTAATGGGCCTCAAATATCTTGATTAACCCACTGACAAAGGAGCCAGCTTGAATGTGTGACGAGGTAAGTGGACAATTTACACTCGTATGATTCTGTGGCAAGAAGCATTGTAAGTAAGACATCAACAGGCATGCATTACTGATGAGATATCTCTTGACTTCCTGACCAAAGCAGCTATGAAGTTACTGTAGGACGAATTTTTTGGTAGGCCATGCATTATGTATCCCATAGACTGGCATTTGGATTTTATCTTTTCCTTTCAATCCTCAAAAGTTAGCAACTGTATAAATCAAGGCTGCCCTGATTAAAAAAAAAAAAACCTGATGTTAGAACACGTTAGCAGAAGCATCAGTCTCTTCTCCCCTGCCTTAAAAGAGGACAGAACACAACAGCTGCATCAGTCTCTTCTTCTCTGCTTTAAAAGCTGGTCCTCATCTCACTTTAAGTGTCTTTGGCACCTACCAGTGTCAAGTGCCAGAAGAACCATGTGTAATTTACAAGAGCATCATGGCAAAAAAGCTGCAAAGAGCTATCAATGGTACAAGCAAGCTGGACCATAAAGATAACTAACCTTTGAGCCAGTTAGCTACCACTGCTTATACCTTAAATACTCAACTGGTGCCTCACCAAAATGCAGTAGCTTAGTGTTTGCATATAAATCACTTACATTTCATGTATGATCCAATCTACATACGTATACTGAAAATGAAACACCTTTTGAATACAACATATAAAAAAACCAGTTATTCAAGACATTAAAAAAAAAGTTATTGAAGAGCCGATTCAAAATTAAGAGCAAGAAGAGATTCCCATTTGAAGCAACAGCTAATGTGCAGGTCAGAAGTGGAACTGTAATAAAATAAGTCTTTTTCATTGAATAAACTGGTAATAACTTAGAATGTTGGATTCGAATTGCATAAGAATAGATCAAAGTTCACTTTTAATAAGACAACTTGTAAAAGTCAGACATGTTAATTACATGATGACATGTAGTACACCATTCAAGAAAAATTATTATAGATTTTGAGGCAAAAATTAAATAACGGTAAAGTAATTTTATGATGACATGTAGTACAACATTCAAGAACAATTATCATAGATTTTCTGGTAAAAATCAATTAACAGTAAAGATAATCTAGGACATTCAATATGATATAAAATCAAAAGGAGTTAGAACAAACTTATAAAACTTGACCATAAAAAAATATTTTGCATGAAACAGCATTTAACTGAAATAGATTTATTACTCTTGTATGTCAATGTAAATTGATACTGTATAAAGTATTAAATAGAAGCATATACTGTACATGCTGTTTGGAACATTAGGCTAGTTACATTGCTGTCAACCCCTTTTGTATGCTGAATACTGAAGTCTGACAGGGCACTGGTTGACAAAAGCAACAATAAAGCAGTTTGTCGAAGAATGAAAGAATGATGTAGCATTATACAGCAATTTATTTCCCTTGATTACATTAGTTTCATAATTAGCATACATTAAGTTAAAAATTAATGCCAAATATGGCATAAAAGGGTTAACAAGTTATATATATTTAGCAATTATGTACTGCCCAACTGATTCCAAAATGATACAATCACTAACACTTTTATATCCAATCAAATCTCAGGGACTGAAAACATCACAGAGTTCTTCCTGCAGGAAGACTGGATTGAGGTCTTTTGTAGGCCTTCGGCACTTTTGAAATAGTAGGCCCTGAAACAAATTCTGAAAATTTATTCAGGTAGATTTATATTTATTTACATTGGTTGTGCAGACAGCTCTCTTAATGCTGGTATGACTCTAGTCATCAAATCATAAAGAACAGTGCTTTGTCTCTGCGTGCGTATTTCAATCTACAAGATCCTGCCGATTTTTGGAAGGAAGGCACAAGGCCACTGAGCCGAGTGTCTTTTCTGGTCCTATGATACTACAGCTAAGAAGGCCATTCAATAAAACTGACTGGGAAAATCTAGTAATTTTGCCATTTGGAGATTGGTATGTTCAAAGAGTAAACCATACGATGTAAGCTATGCTAAGCTTTACTTTGAAATATATCTTAAATTACTTTGACAATGACTAAGTTTCATCACAGATAGTCTGCCATTTAAATTGCTCTATTAGGTAAGACTCAATATTATTGAGTTTAATAATCCACATAAAAAACATTATACTGTAACTCAGAAACCAATGTTTGCAATAGCTCCTGTATCAAATTTATGCTATGTCGAGAAAACATAAAATTCATTATTATGGTAAAATTTACAAATGCAATTTAAAGAAAATTTCAAAATTGCTCTAAAAATTAAAAACTTAAACCCAAATTGATTTACAGTTAACTTTTTTAGACACACTTGATTAAAGATCTCAAAAATCAAAACATTAGTAAAATTGTATAAAACTTCATTCAATGCAGTGTCTTAATGCCAAAAAAACTACTGGTAAAATTCAGATGAGTACAGAGTTCCATTTCTAACAATGTATCAATAAATATGAAAAATATAAACTGAAGGACTCATAATTCACCTGCACCTATCACTGTCATTCCTCACAATGACTGACAGTTGATTTACTTATACTATTGCTAAATCATTTAAAAATCCCCAATTCAAACACAGAAAAAGCAACTTGTCAAATCTCAATGTCTGTTCCAAAAATGTTACTTTTAATAGCGCCCCTCTGCTACAGAACTGACTTGACTGTATATGTACAGTGCTGTTTACCAAAGAATTATTGCTCGAGAT

>chr13:+:54970107-54972741

AAAGGTTAAATATTTATTACAAAAGGGTAAACTGAACATGATGTACTCGTAGCGTACATAGATGTAACTTAGAGCTACACAATAATGAACGTACAGAAATTTCTTTAATATTAAAGATACGTAGCTCAAGACACATGAAAAGCAGTTCAGCTAACACCTTGCAATACATGGTTGAGTTTCTACAAGCCACTGAGAATTGGTATAAACACTTTGTTTCTCAATACACCTATAAAACTGACATTATGCAACCTGCAAAGCCAAGTAAAGCTAACAACTTCACATCCTTTCCACATGACATGAATAGTCTATCAAAATCACAAGCCTTTCCAATTAACACAGGCCTTAGTGGTAAGAGGGCAATTAAATGCAAAAAGGAAGAGAAGGGGTTCAGAAACTACTAGCACATCACAGCACATTAATTTCAATCAATGTGCCTGTATAATTCCACGAAAATGCAATTAACCTCAATTCTTTTTCTCCAAAAAGGAAGGCTGCTTCTTCATTTTTCTCAGCAGCCTGAAGACGACTCACCAAGGTAAATTTCTTTTGTCTGGCATATACTGTACTCTCAAGCTTCCAGCTTCTGAGAAGCCTTTAATATCAAGCAGTTACACATACATATGATACAAATCAAGACGGTATAAAAAATCACTCTTGAATACGATACACTAAAGTTGCTGACCTGAATAGTAAATTACTTCAAAAGAATCCTCATGGAAATGTCAAAAGAAATTGCTTTTTAAAATTCAAAATAATTTCCAAGTAGAAAATCTAACTACATGTGGCAAATAGATTATAGTAATAGTAATAGAGACATTCTTTTTAGAGCACGAGTTACGCATCAGAACTTTCCTCACTGACATAAAAAGGAGAGCAGATGACTTTACCAAAAGAGAGAATGGGGTCAAAATAGTGTATATAAAAAAAGAAAAAAAAAAATCCTCAGCATTGGCCATGCTGCAGACAGCACTTGTCAGTTATTGCAGCTATGAAAGTCAAAACAAGACAATACACTTGAACCTCAATCCCATGGGGTTTGTAGAATTTTTCATGTTTCATATTCACCCAATTAAACACAAGGGTACAAACTATTGTATATCTCTGAAACCTTAAAGTGTGGCTTGAGGAAACACGCCTCAAGCAGGATAATACCTCAATGCAACCAAAAGCACCATCATAGTTTAAGGGCATATTCATATTCTCTTCCTGTAAAGTGTGTTCAAACAGTACTGAGATTGATACCTACACAAACAACTAACTCCATTGTGCATTGACTTTCATGACATAACAAAAAGCATTAGAATGCCACTACTTTATCAGACATAATTACTTTCAAATACAAATTGAAACTACGGCAACTAACTTCTTTCACAATGCCCAGATGTTGTTCTCAGATTCTTACATACAAATATACTGCTGGGTGACGAGTCTTCCAACCAATCACACGTTTTATCACACATATCTCAATGTATAATTCAGTACACTCAATACTAAACTCCTACTAAACACTCTTCAATTTAATCTTAAGCTAACATCCAACTTTCTATTACTTTCCATGCTGTTCAACACAAGCATTTTAATAACCACTATTTTTTTCCAATTATAATCCTCCTTCTATTGCTTGAAAAACTTAACAATACTGCCATACATATTTAACCATAAATTTGGCCCACCATTCCCATAACTTGACTCCTAGCTCTCCTCAGTACTTGGAATGTATTTCTCTTTTACTACAGGCCAGCTTCATTATTACCACTATTTTAGGACACAGTGAAGCTTTAAATGAGACTCAATCAGGAATAAAAGGAGAGTTTAAGGTATTTCGCAATAATTTACTGTTTATATATGTGTGTGTATATATATATAAAATAATTTCCATATGCAATAATTGACAAAATTCTATTAAATTTCAAAACATTTTAACACCTATTAGATGACCAAAAGATAAAGAGGTTCAAGTGTATAACTAAATATTTGCAAGAACAAGACAATGACTACAGCAAATAAAACACTAAGATACTAGAATAGAAGACTACAAAGGAACTTATATTAACAAATAAAATAATTACACAATAGCAGTGAAAGGTTTTCATTATCCTTGGATATCAAATTTGAGTTGTTTTATGCAGTATAGATCAGCTGGCTCTGTAACCAATGTCAAGTGCTGAGTTTGATGTCTGTTTATATATACAGTATTTGTATATATATATAAAAATTGGCTCACTTTAAATAATATGGATAGAACAAGATTAAGCCCAAATACCACTCATTTCAGAACTGGTCTGTTATGATCTGAAAGAGCGCACATTTTAGCATTTAAACTTGCAATATCAACATTACTGCAAGCAAGGGACTTTCTGGCAAAGTATACCCTACCTCTTAAATCTCTGCTTCATAGTTCCAACGTGGTCACCAAATATCACCATGGCAAAATACATAGGACTTTTATGCCATACCTTGAGCAGTCAAATCACAAAATCTCTTCCAAACTTAAACCACTTTAAATGTACATACAGGTATGCCACAACCTCCCTATATGCACTATTTTGTCCAAGATGGTCATCTATATCTTTCACTACTAAAAGAAAATTTCATCCTTTATAAACTATTTACACAAACCTGGTGTTACAGGCAG

>chr12:+:3686690-3688645

GGAAACCTTTGTGAATAATATGGAAAAGTGGATCCACATCATAACAAAACATAGTAAATAGTATTTTAAATTTCTATTGAATAAGAGTGGTCTCTACTTACATGGCAAATAGCATTTTCAATTCCTATTGAATAGGAGTGGTCTCTACTTACACAGCTGTGCCTTAGAGCTATAGGCAACAAACAGCTACTCAGTTTAATATTACAGTTGACAGCTTATTGAAACAAGTCAAACGTTCAGTTAATATACAAACATTCAGAAAAGAATATAAATATTTACTTAACAGCAAAAAATTGTGTATGACAAAAATAAACAGTATATGTATGTATAAAAAGAATTCACATTTGCAGCATGCATTATAAATCTGACTTGATTAAAGCTGCAAGCAAAGAGATGTTACAAAACTTCAAAAATTAAAGCTGGTTTAGAAATAATGTTCTTAAGAAACATAATTACAAATACTGTATGAATAAGCACCACAAATATCATCAATTGTTTAAAAGCCAAGTGAACAAAAGTGAAATGGAAACAAGAAAGATGCTCAGGCAAATGAAAATCAGAACCTGTTATGTCATTTAAGAGTTAAGGGTAAATATGCTAACTAAGTCTGAACAACTTGTGATCTAAACGATACAATATTTACCTTTCTTGTACTAATGTTCTGAACTTTGCATCTGTACAATAGTTACTGAGATATCTATACTGTATTATCAACCATCACCTTGTATTAGATACAAAACATAATACTCCCTAACAAACTAAAATATAACCATCACTACAAAATATATCAATAGTATTATTATCAGCCTTCAAAACATAACCACCAACTTTGCAGAGTAAATAACTGTTACACAGGGCAGATGGAAATGTACCTAAATTATAAAAGTTGATTACTACTTGGCTGTTCAACCTGTCTGAACATTAGTGTCAAACATCACTGATTGGACACTTGACTTCTTTCTGACTTTGTTAAAAGACAACAAATCATGAGCTCTTACAACTCTTCACACCTACAAGTGAAACTTCAGATACCAAATGAATGTATGTCATCAAACATCTTTAAAAATGCATAATATTAACATCATTCTTGTACAATAACCATATATACCTACCTATAAATTTTGTACACCACTCAAACTGTAAAATATTTTCTGCAATGGAAATGTTATATATTTGTTCAAATATGGGTTGCCTACACTATTTTGCTTGATTTACTTACAGAAAGAGATGCCCCACATGCTGTAAGTGGAATTTACATAAAACAATTATCATATACGTACAGTAACAGTTACGTATTACCAAGCATACATAAATTTAATCATAAATTCCTTACAATCATACAAACCATATTCTGTACTGTATATCAAAGAGAGCATTCGATATCCAGTAATTATTTACAATATGACTGGAATAACATGACTGATCCAGGAATCTTGGTAAAGAAGGTAGACACAAACAATACAAATATACTAACTATGTATTTATAATACTGAACTAAACCCTGCTAAAGAAGAGTTCAACTTAGGTAGCTATAGTATGAATAGGTAACATAATAAATAAAAGTGATTCTTCACTTATTACAATTAACTGGCACAACACAGTATGGCTTTTGGGTTAGGTGTTGACTGAGGAAAGAAATAAATAGAAATAAAAAAAATATAATTTTAAAAAGCAGCTATAATAAACTCAATACAGGAATATAGAAAGCTACGATATAAATAATTCAATTACCATATGCAGGATGTAAAAAATTGGTAGGAAAGGCAACTAAAATGCAATACGCACAGTGTTCCCCTTGTATTCGCGGTGGATGTGTACCAGAATCCGTAAATAGTTGAAACCCCATATCAATGATCAAAACTGCCTATTTTGTTGGTTAAAACTCAAGAAAAACCCACTAAAAATTTTTAAATCAGGGTTTTTTTTTAATAGTTTTATCACAAAAAGTTCATTAGG

>chr12:+:58188375-58190356

ATCCCAATGGAGAAAGCGTTGAGATCAAAGGCCGGAGCCGATGCAGAGGTGGAAGGGTAACTCGGTAGAGGTACCTCAGGAGGAGGAGAGACCGTTGACTCCGCCGGAGTACGGGGCCTCTCCTTGGAGGACTTGCTCTTGGAGGACCTAGAGCCTGAAGCAGAAGATTTACCCTTCTCTGCTCCGGGATTTGAAGCCGAAGACTTACGAGACGATGACGCCGACGAAGTCTTTTTGGATGACGACGACGCCTTCGTTAGGGTTTTCTGCACTCTGTGCCCCTTCACCTTGGGTTTCACAGAAGCGCTAGGCGTAGAATACTGGAGCTCAGCTCCTGTAAAGCCTTGGAAGGAAGCAGTAGAGATAGCGGGAGAAGAAGATCCAGAGGCGCCCAAGGGAAGCCCTTGAGCGTCCAACGTACCTACCTCGACCAACAGGTCGTCTACACCTACCATCGGTTCCACATTCAGGTCCAACGTCGCGACTTCCGCTGAGATATCCTGGCCTGGTTCCTTCATGCATGCGGCGACCTGCCGCTGGATTGCCGTCATCGTCGGGGCTGCCTCCGCCGGGTCGACGTACCCCGTCGACTTGCCGCCGGGGAACAGCAGGACAGCCATCCTCTTTTCTAGAATGTAGGGCTGTCCCTTGGCGGCGTTCTTGCCGAAGCCGCCGACCCAAGCCCTCTAAGTTGCAAGGGCGGTCTCTCTAACAGCGGCAGCCTGTAAGAGAGGGTTTCTATTAGTTTTTAGTTTAAAGTTGAGGTTCAACCTTAGACTAAAATAGAGCCTTAAGCCTACAAATTTTGGGCATATATTTCACAAAATATGGAGAACTTAAGCTTAAAAGACATATTTGAAACTTAAACTTAAGATATAATCCAGTATTTACATGCTATATATACAGCTGGAAATACTTACCCCGTCCAAAAACTGACTCACAAGATCATAGCAGATCGTACACGTTTCGTGGAACCAGACCTGCAGATTCCCGTGAGGAGTCGCGCACGGAGCATGGGACCTGCAGACTTCATGTCCACAGGGGTCCTGGAGCACGGCGTTGCACCCTGGGTGCTCACAGTTGGTAGTCTGTAAGTGGAAGGATACATGAGTATCATTATAACACTTACAGGGCTACTCTAGAACTCCGTTGCATGCCGGAGAGTGAAAAATTTTTGGGCATAACCCCTCCCCTACCGCCTTAATAGGCATAATATATAGCTCCGGTGTGAGCCGAAGTAGGAAACAGAGGGCAAGGGAGGGACCCAGCTTAAGATAACTTATCAATAACTTATGATTAACTTAAGCATAAAATAGCACAAAATTCCGGAAGACGATTCCGAATCGCGGTGGTGTCCGTCTCCGCCGGCTGCGCCGGAGAGACGGGATGGTTAAGGTAAAAGAGACCGACTGTACGCCCGAGGTCCGCCAGTAGGCGGGGTACTAGCTCCCTCCCTCGCAATCGACGGGGCTCCGGGAAGAAAACAGAGAACCGACATCATGGGGAGGGGGCGAGAGGGCGAAACAGAACTTGAGCCTACAGCGGAGCAACGGAGCAACAAAGGACGGGGGAGGCCAACCCCCCCACTTCGCCACAACGGAGCACCGTGAAGCAGAGAGACAAGGTCACTCCCAGTCCAGTGTCTGTCCAGCCTCCCCTACTCCCTCCGCGTAGGGAGAGAGGGAGACAGGCCCGGGTGGAGCGAGCAAGGACAGACCTCCCTCCCCCCAACTCTATGGGAGAGCGGGAGGAGGGCAGGGTGACTGGGACGGGCGTCTGGCTGTACCGCGATCACAGGGTGACCACGGTACGATAACACGATATAAACAACGAAATCGATAGCCTAGGTTAATGCAACCAAACTGATCAGCAAGATGCAACAACTGATGCAACAAAACTGATAGTAAGGAAGCAACAAAACTAATGGGGACCATGAGACCATAAACCTAGGCTAAGATAACGTACCAGACGCCCTAGGCTAA

>chr12:+:68468444-68470552

GTTCATGGAGAAAGCGGGCAGCGAGTCATACGGGGGAAGCTGAGATCCACTAGTGTTAGAGGTTGAGGGAGAGGCAAGGGGGGCTTCTCTTAGGCCGGCTATAATAGTATCCTGAGAAGTTATCCTATCCGACAGGCGAGATATCATATGTTCCATACTCGTCTTAAGGGAGCCTACCAAGTCGCCCACCTGTTGCAACAGGCCAGCATTGGTGTCCAGAGCCGGAGCTGCTGCGGAGGTGGAGGGGAGACTCTGAATAGGCACCAAAGGTAGTGGAACTGGTGATACTGCCGGGGTACGGGATTTCTCCTTAGTCTTACTCTTTGAGGACTTTGAGCCGGAGCTAGACCCTTTAGACCTGTCTGAGCCGGGATTACGAGCCGGAGACTTACGCGAAGAAGAAGACGAGGACGTCTTCTTCGAGGACGATGACGTCTTAGTCAAGGTCATATGTTTAGACTTGATCTTAGGCCTAACCGAAGCCTCTGGAGGAGTATATAACTCATCACCGGTAAAGCCTGGGAAGGATGGTGAAGTTGCAGGGGTAGAAGAAACAGTCACTCCCAAGGGGGACCCTTGGGTACCTGCATTACTTACCTCGACCAACAGGTCGTCTATACCTACCATAGGTTCAACATTTATATCCAGGGTTGCGACATCCGTGGAGATATCCTGGTCTTGTTCAGTCAAGGAGGCCGCCAGCTGTTGTTGGATAAAGGCAATAGAGGGGTCCGCCTCTACCGGATCGACGTATCCAGTCGCCTTGCCTCCGGGGAAGATTAACAAGCTAAACGCTTCTCCAGAATGTAGGGCTGACCCTTGGCGGCGTTCTTGCCAAAACCTCCGACCCAGGCCCGCAGGGTAGCCAATGCCGTATCTCTTACTGCCGGAGCCTGTAAGAGAGGATATATTTTAGATTTAAGAACCACTTAAAACTAAAACTTAGGATATAATTCTTAAACTAGATGCCTTAAGTTAAAGTAATGATGAAAACTTAAGAAGTAAAAGAGGCGGAGCGGCATGCGGAGATGGAATACTTACGCCTTCCAAAAGCTGGCTCACCAGATCGTAATATGGTACACGTCTCATGGTACCAGACCTGGATGTCCCCGTGCGGAGTCGCGCATGGAGCATGGGACCGGCAAACTTCGTGTCCACAGGGGTCCTGAAGTGTAGCGGAACATCCCGGATGCTCACAGTTGGTGGCCTGTAAGTGGGAAGACACATGAGTATCTTAAAGGGGTAACACTTACAGACTAAGGACAAAAGAACTCCGTTACGTGGCGGAGCTCGGAAAAAATTTGGGCATAACCCCTCCCTGCATGGCCTGAATAGGCTATAATCCCGGAGAGATCCGGTAACTACTAAGGGAGGGGTGGGGATGGTTTAAGAAACTTAAGATAAACTTAAAGATAACTTAAACCTACATGCAAGTAACCGGACTAGGTCCAGTGAAGCGGAGTGTAGTAACTCAGCAATACGGTACGGTTAGTAATGACCTATTGTATGCTCCGGTCCAACTATGGTGGACTATACCCTTCCGCTGGATACCAGGACTCCGATAGGGAGGAGCTCTAGTAAGGAGGGAAGGGCGAGATGACATACAGACTCGCGCTAACCCGGAGCGACAAGGAAGTAACGGAGGGGGGGAAGGCAGAGCCCCCCACAACGTACCACCCGGCCGAGCCGAGAAGCGGAGAGGTCGGAACCAGTCAGGGACTGTCGGACTCCCAGGCCCCTCCGTGGAGGGGAAGGGGGAGGCAGGCTCGGGCATGCTGGGCGAGCAAGGACAGACCGACCCACCCCCGCCCGACTCTATGGGAGAGCGGGAGAGGGGGAAGGGGACTGGCAGGCGCCTGGCTGACTCGTGATCACGTAGTGACCACGAGGCGGTAAACTAAGATACGGTAACCAAGCCTAGGCCAACCAACTGATCAGAGAGAAGCTATCGGGAAGCAACCAGAACTGAAAAGCGGTAGCATATAGGCCCATAGGGCGAAAACCAACTGATCAGAGAGAAGCTATGAGGAAGCGACCGAACTGATCAACGGTAGACTAGGCTCTACGAGCCAGGACCTAGGCTAAGCCAGACGCCAACTAACCTAACAA

>chr12:+:94430142-94432920

ACATTTTGTTTTTTCATTAAGCCTCATGTATTGCAGTATATATTTCAGTCTTTATTTCCCTTCAAATATATAAAAAAAGTGAGGTATATTATGAAGAAAGCCACCGTCAAATGCGATGCTTATTCAGAAGTGCTAACCGACCAGCAAGCGCTTGGTAATTGTGTCTTGACAATATTGGAAAAATCCTATATCTCTCATATATTAACTTTGCACTCAAAACAGTGCTGTAGCAAATTAAAAGGCTTTGACCCTACACTGCTTCTTAAAAGTCTTTTCGCAAATGTCATTGCCATTAAAAAAACCACTAACTATTTACACTTTGGTATTATAATATCCTTTCTGACATAGTAAGCCAAGAATGAAAAATTTTCAGGCTCTTCATTTTTAAGTATACATTATATATCACACATTACTTTCTGCAGTACTGGTGTGCCTGTTTGGGTGAAAACAAATTAATTAATACAATAACTACCAGAGCATCTATGCAAAGTACAGCTATCGTATCTCATTTAAACTATTTAATTGAAAATATATTCCTGTAAGACCCCATACAATATAGAAACCTGCCTTTCTATGCTGATATTAGTCATGAACTGGATTTACCTTTAACAGATTCTATTAGGACAACACAGTTAACTGAATTACATAGTAGTCAATGGTACAGTATATTAGAGAGTATAATAAACACCTATATACTTGAGCCACCCAAATAAAAAAAAAAAAAAAAAAAAAAAAAAAGTTGCAGTATAAGGCTTTCAATTGTTGAAGGTCACAAGATTATTTGATTTACCCAGAGTTAACCATTAAGAATCACTTATGCTTAAACATATATCAATGGATAATAAAACTTGTACATTTCCTCACTTGAACAATTTAAAAGACTTTCCCTATACGACAAGCTCCAACAATGCTGCTACATGTGATTCAGTTTCTCAGACAGAAACCCATGACAAATACTATTCGCACCAAATGAAAGGCTGATAATATTGAGGCTACAAAAGATCCACTTCATCTTCCATTGCTGTGGGAACTTTCACTTACTTGAAGTCATGACACTGGGGGAAAAAATATGCTACAACCTGTATAATCTACAGTTGAAACTTTGATAAGACAGAACAAATTATTGCTATTAATAAATTAAAAGTTTCCCATGTAATTCTAGACTAATATTAAAACTTGCTTTTGGTTTAACAAACACAACCCTTTGCATGGATATCAATCATAAGGGTCAGTTTACTTAAGAAAACTAACACAAATTTTCTCTTTGCCATTTGCTACTAAGCAATTTATTTGTATTACAAATTTTGCAGTTTTCAGTTTTATTATTTTATTAAATCGGCCTTTACTGTGCAAGGAGCTTTCCTGTGTGTGTGTGTGTAAATATACAAATATGCATTAATACAAAGTTGTACATGCATACTGCATGTACAAATACACTTAAAAGAGTACACATACTGTTCAATAGAGTACGTATATATGTATACTATATATAGGCATGTACACCTTAATGACATACAGAAAAGCAAGATATTACCTTGTACATAAGTAAATACTTTCAAGAAGTGCACAAAAAGTTATCTATGAGCAACTTGCATTGAGGAAAAGAAGTTATAAAAAGCACAGCAGGAATAGTTGGACGGAGAGGGGAATTGACAGGCTCGCGTCGGTATGTTGGAAACAAGATTGTGATATGTCTTCCAGTATATAGAAATGAAAACCCATTAAAGTGCATCTCTACAGAACTTTGAATATTAAAGCTTTGCTTTATATTAAAATATTAACAACCTACTCCACTGTTTAAACTCATTTCGTGCAAGGATTAATAATAATTATGACATATCACATTTGTCTGATCAAGACAGGTCTTGCAGTCCTTTGAAGTGACATTGGTTGAGCACACAAGCAACAGCTTTTCCGCATACTTGATATTTCAGGGGTCTTTATTTATTCTACTGTACATTTTGTCCCTCTTGGAAAATCATAACACTTGGAACTTGAAAGCACTTGCTGGAACGTTTTGTAATTTTCATAAGTATTCATTCTTATTCATAGTAGTACCTACTTTGTCATTAAGTTCTGAGTGATGCAAGATTAACCCATAAATCCAGTTTGGCACTTTTAAATTTTGTGTAGTTAGTTGCCATAATAAGCCAACAAAAACAAAAATCATATGCAAAGAGCTTAAGTGAATAATTGATCCCATGAACCACTTTGGAAACAATACCATAAACATCAATGTCTTAAAATTTCTCTCCCAAAATGATCTTAAAAACTATATAATAAAGAGGCACAAGATAAGATCTCAGAAGTTTTAGACAATCCCTACAATATTTAACAAAGGTTTTCCACAGCCATAAAAGTTAAACAATTAAAAAATAATAAATCTGTAGTGCGATATAAAATATAGGTCTAATGGATTCTCCATTACCACAGGCAAGAAAACTCACCAGAAATTAGGAGTAAACCCCATCAAAATTCTCCATAATGAGATTAAGGAATTTACTTATTAAAGATAAAAGTTTTTAATACTTTTCCTATATAACTGATATAGCGTATCATGGTCTGATAACTAATGGGGGAGGGGGTGGGCCTCATAGCAAGAGGTACAGAAGGCACATGTATGTATCTGGGAAGGGAAGCTTTCCGTTCTGGTCAACAAACCGATCTAATTCTTTTACCAAGAGATCCTAGACCAAATCAGAAAATAGCATTATCTTTGATTGCATTTGCAAACATGACTTGCAACAGTTTTGCCATTACACCTGAGGAAACACACCCC

>chr12:+:103098753-103103140

CAATTGGACAATTCGGTGTCGCTTGGCCTACAGGGTTCTTCCTCCTTGGATGGCCTATTGTCACATTTCTTGGAGGCACGTAGCCCCCCCTCCCCAGCACTCCTTTACATTGGTGGCAATGGGAGATAGCCAATGGGGAAAGAGGTAGACAGAGCCCGACCTGGTTTCCCCCTTAGCGCAGTGGTTCTTAAACTTTTCATTACCATGGCCCCGCTCTTAGAGTTGGGCCCTACCATCCATGCCCCCTTGCATCTATGAATAATATTCCCTCCCAGATTTAAAGGAAAATGAAAAAGAGAGAAAGAGAAGTGTTTCGAGGGATAGGCAGGGAGGTAAATAATTTCAAATCATGGAAGTCCAACGAGCCTAACCAAATAGTAGACTCTATGAAACTAACGTTATAAGCCAATAATTTTGTATTAGTTCCTCACTCACGTTGAGAGAATAACAAATATAATTAGAGAATTTTTAATTTTTCCCTAGGGCTCATGCCTCCCCTGGAAATTGATGATGCTCCCCAAGAGGACATGTCCCCCCCCGTTTAAGAACCATTGCACCTAGTGGGTATTTTGATGCTTCAGTTCTTTCTTAACTGCCTTCCAAGCAAGAAGATTACTCTCTCACTCAAAGCCAGTTGTTTTCAGCTCCTCCTGCCCACTTTATGCCTTGACCTTTTTTTTTTTCTGTTATATTGTGTTACCTGTGTTCTTGCATTCCCAGAAATGCAAACCTATGAATCATCTTAGCAATCCAGACTTCAGTGAAGATGCCCAGGCATTGGTAATAACCCATACCTTGTTATTTAGCCTCCTTGGATACAGACCCACATACCACTTGTACCAGATGCAGGCAGATCTAATGGTTGCAATGGAAGTAATCCTTTTTCCAAATGTCATAATTGGTTTCCAGAGCAACGGAAGAATTGGCTCCACTCCTTGCTCTACCTCTTCCATGCCAAAATCTCTTCTCTGAGATCTTGTAATTGCTGTAGCTGCTGCTGTGCGAGAGTAAGTTTCCCTGTCCCATGAGCAAGGATCCAGCTACCCCTTTAGTCTTTTCAGGCATGGATGCAGAGAAGCTCTCAGTGCTGAGCATCATTAGGCCTTCCATGGGTTCCCTCCTTGTGGGGGTCTACTGTCCCACTTCATATCTAACCATGTTTCCACAGCTCCTCCTTCCTCATCCTACAATGTCTGTCATCAACTGTGCCACCTCCATTGGCTTCCATTCCTGATCCTTGTATGTCTGCTGCTTCCAACTCTCTTGTTCAAGCTCTGTTGGATATGGTCAATAGTCACAGGTGCTCTTTTGAAGAAGTACGGTCAAGAAGCATCATAGATCTCCTCTTCGTCTTTGGCTTCCTCCTCCTCTTCATCGTCGTCTAATCGTTCCATTCCTCAGACGTTGGAAGAAAAGACTTCATTGGACCTTCACGTAAGATGATTTGCAGGTCCTCTCTGGATTCATGGTCTGCTTCTTCTACAGCCAATCATCGTTCTGCTAGACATGTAGTCTGTGTTGAACCTGTTCCTGCTGCACAGATGTGCACCTATCAAATCACACATCAGTGTCTCTTCTAATTTAGCCAATGAACATTGCCCAGATACTACTGACTTTCGTGCAGCAGCACACACATAGGGAGGTCTCCTGTAAGATCAATGAATAGGCGGATCTAACTTCGCAAGAAGTCAAAGTTATCTTGTCTTAAGTCATCCTGCTTAAGGAGCAAGTGTTTTTGCCACGGCTATGCAGGACGACAATAAGACTCCTATTAAATTTGTCATTTTTCTCAAAATTATCAATTATATTTCATGTTTCATTCTCCATCCAATGTCTTTAATAGCAAAATAAGATAAGAGGAACCTGCTGTATCATTTGCAGTTTTATTACAGTACAATACATATACAGCATTTTTTTGGTGTCCCATTACAGTATAACAGTGCTATATTGATATATTTTATATTGTTTACCAGTTAGAGGAAAGAAAGTCTGTAGTTATAAACAAAATCTATTATCACCGTAAGTATTCTTTTCCTGCTAATGTAGTAAACACATGGCATTGATTGATGGCTCCTACACAAAAAGTCAGAAATTCAAGGGTAGGTTAATTACAAAATTTTCAACTATCCATACACTTTAATAGTTCATCTCTAATATATCTATCTAACATGATCATGTTTACAAGAAATGGTATGCATGGGTCATTTTACACTTTCATCTCACTGACTATCCCTGTACAGAAATGGTAATGTTTCCTGACAATTTTAAGCTCAGTACTTGAGCTATGATATACAAGTTAAACTTAAAATGTTTTCACAAACAGAAGAAACCACACTGCATAAAAAGAACTTGTCAGTTTAATATGGGATCTTACAGTATGTATTCCCAGTTTACATTAAACTTAAGTTTGATAACTAATGTACATCTTTAACACCAAGATTCAGATTATTTATTACCTTTAAGGTTACCTTTTAAAGTCAGGTATGGTACAACCTCAGCATCTTCTCTTGTGACCATGATCGATAAAACACAAGGGAACATTTATGTACAATGTTCTGTTTTGGTATTAAATGCATATCAGATCTTCCATCATAACATTTCTCTTCAATTTCAAAATATGAAAATAAATGTATTAAAAATAACTAAGCAACAGGCAACTTGTGAACAGTAATTCAGTTATATCTTTTCAAATTACAACTAAAGATTTGATACCTCCCCATCTACTTGCACATAAATAATGTTTTTCACTAAACATCAGTAAACCACTGCAGAACAAAATACAGAAATTTAGAGACTAGTCATAGGTAGAATATGCCTCTCTCAAAATAGCTAGAGCTCACAATTTCCGGCACATTTACTCGATGCTCATGAACATACTTTCAGAAAAAAAAAAAAATATGATCATAGAATCTGAAGTAAATGATGAAATACTGTTGCTCAATTTATAAATTGTGCTAACTTAATAGGGTTTTATATACAGTAATGTCTCCCTGACCCTACACTAAAATATATTTTGTGTGAAGTAAGGATATGACTGATAAGTCATACATTTCTAAGTTATAATGTTTCATGAAGGCTCACCACACCAATATGTGCATATATTTGGGATAACTAAGCAAATGCATATCTCCCAATGAATCAAGTGTGTGAATTATCAGTCACATAATAATTCCATATTCTTGGTTATTGCTTGAGTCATGAAAAAGAGAAGTGACCAGTATAACCTAATGAAGCCCTTGCATTTGAACTGCAAGAAATGACCCCATCCTTGATTTCATTTCCCCCTCAAGCACCAACTAAGAACTTGGTGAAATAGCAAGGAAGGTCTGAAAATGACATGTGAATAAACACTGACCATGATCATGAATACATGACTTGACATAAAAATCTGGAACTCTTAAATCTCTATATACAACGTTACTTTACAGCCTTTTAATAAAGCAATTGAAAGTAAGAGATCAGGAATTAAGCAAAACAATTATTAAAGAAGAAAATATAAAATTACACCTAATGGAGTTGTTTGGTAAAATAAAGAAAACTATCTATTCGTATGCTGCACACCTTAATCTGTATAAAGGTAAAAATATCTTTAAATGCAGATTTATCAAAATTGGGATAGACCAATTCAATTTCTTACTACATTTCTGCTTTGGAACATTGTAGTACATGTACAAAAAATATCATAAAAATAATGAGATATTTTGAAAATTTTGCTTCTATACAGTGTATTTATGCTATTAAGTGGTATTGATAACTATGTCAATGCATTGTTATTTGATTAGAAAAGCATCACTTAGATGCATTATCTATTTTACAATGAACATGTCTACCAAATTATTGATTCCAAACCTAAAAGCCTTTACACTGAACACAATGCTAATGTACGGAGAATGTTTGAACACTTACTAACCCATCAATAATACTATTATAAATAAAATATCTGAGAGATCTAACTTACTGTATAAAAATGGGATTATATTTGTACATTACAAACAAGTTACTGTACAGTTCCATTAAATGGTTTCGTATGAACAGTCCACTTAAAATTTAACACAAAACTTAGGTGATATTCTATCTAATTTCCAGTACAAAATATACTGTAGCAAGACATGAATACCCCTCTAATGAAGGGTCTACAAATGAATTCACAGCACTGAAACTCCTCATCTGAAGATTATTTCAACTTGTGCAAAAGTTGGGCATCTTGTCCGTGTTATGTGGTGGTACAGAAAGCCAGATACAGCCCTGTCTAGCACTTTTACTTTGCAAATAGAAACAAATTAGTGGAAAATAACTTTTAATCTCACCTTGGATCAATGAATGACAATGATTTAATCTGTTTCCTTTAGAATATTCTTTTGTGTATTAGCTCTTTCATACTTTCATAG

>chr12:+:104438386-104440912

GGACTGCCTGAACCAACGTTTGTTGAGAAGGCTGGAAGGGAGAGAACTTCCTTGGTTTCTTCTTGTTCCTTGGGTTAGGACCAGAAGATTCCTGCCTCCGCTTTGCTACCAGTCCCCACCTGACTTTGAGGCACTGGTTGACCTTAGAAGCCTCATGAAGGACTTCTGCTACTACTGGTGCTGGAAAGGTCGCCGTTCCCCAAATTGAGGAAGCAATCAGTTTTATTCGGTTCATGGCGGATAGTAGCTTCCGCCAGAACGTGCTTCCTACAATTTCTCCTGGCTATGAGGAAGTCGTAAAGGTCACACTGCATGGTGTGCAGCAGACCTTTAGCCAAAACTTTAAATAACGCTCTTGTTGGTATACAAGAGCCGTCATCTCCGCCATAGTCATGGAGGAAAGGGATCTCGCGAGCATAGTCCCGGGCGTCGTACTCCATCTGATGAGAGTCCGACAACCTGGGGAGTCTCTCACTAAAGAGAGAGGTGGCACAGTCCGCCTTTAGTTTTCCTACTGAGAAGTAGGAACTGCCCCTTCGAAGTACGTCTCGGAGTCCTGGGACCTAATAGAGAGGGTGGGTTCCGTCTCTCGTAGTTGAGGCAGGGGCTCGTCTTTCAGGGCGGCCTGAAAGGTTGCTTCCACTACCTTAAGGGTCAGTGGTAGCGGAGTCCCTTCCACCGGAGTAAAAATGGTGAAAGGACTCCTAAAGGCTGTGATGCGGGTGTTTTCACACCCCCATTCTTCCAGACTTCTCATTAGAGGTCTGCTGTGGCCTGTTCCCTCGGAAGGATCACTGTTTCCTTGGGAACCTTGTCCTCTCTCATCATCGCTGCTTCCGTTAGTCGGACGTAGCCAATGAATGGTGGTTGAAGGTCTCTGGGGTGGAACTCGAAGTCCTCACGCCTTCGAGTTCCTATGCCTTCGATTGTCAACATTCCGTTGACAAACGGGGCATACGAAGCGATCCTCCAAGGGTTGGCCGCCTTGAATTCCGGCAGTTGGCTGGAATCTGGCATTGGAAGAGGAGAAGGTGGCACAGATAGAGGGGAACCTGCCGCAATCGAGGTTTGGATCCCGGCGATGGCATTCTCCTGAGCGGCCAGCCGTTCCGCCAGCGATTGGATCGACTGGCCGGAAGACGTAACAGAACTGGAGAGCTGGGAGAGCACCGAGCCGACCTTGTTGTCAACCAAGGCCCTACGATCACTCCCACCTGCTCCAGAATGTTAGCCGAAAAGGATTCGGGATCAAAAAGAGGGACTTGAGCCCGATCCTTACGTGATCTATGTTTGGGGGACCTCGACGCAGAGGAAGAGGCCTCCCTTGACCTAACTGATCCGGGGTGGTGAGCCGGAGTTATAGTGTCTGTCAGGATGACCGATTTCTTGGGGAGAGACTTTTTAAGTTTCTCCTCGTCATCTTAGCCTTGGGGATCACTGAAGTAGCCTTAGGACGGACAGACCGCTGCTCTTCAGTGAAGCCCCGGAAAGAAGTGGAAGTAGAAGGATCAGTAAGAAGGTGATGGAGAAAGGGAATTCAGGAAAGGGCCCTGAGAACCCACAGGAGCTGCCCCTACTACACCCTTACCTGTACCCATGGAGTCAATAGCCATGGGTTCAACGTCGAGGTTCATTGCCGCAACGTCTTCTGCAACGTCCTCTGGGAAATCCCCCTCCTGTAGGGAGATCATGACTTGAAGGTCGGCCAGCAAAGGGGCGGCCGCTATCGGGTCCACCACTGATCCTTTCTTGGCTCCTGGGAATATTAGGTCCGCCATATCCTGGGTAAGAATGTAGGGGCGGTCCCTTAGAATTCCGGCCAAAACCCCCTACCCAAGCTTTGAGAGTCGACAAAGCTTTCTTCTTTTCTTTTCATCAGAACCCTGTAAAAAAGGGTCTAGAGTTAGGGAGAGATAGGGGAGGAATGTCTGTTGTGTTGAGATTATATGAATATGTGTCTCATATGTGAAGGTATAATAAAACAACAAGTATTCCAGGTGACGAATGAATGAAGAAAGTGCTAAGAAACTTACTACTAGTGAGGCATCTCCTACTAGCAAGTAGCAGGTTTGGCAAGCTTCATGATGCCAAACGATAAAATCCTCCACTTTCACTGCACATCCTGCATGAGAACGGCATACTATATGGCCGCATAGGTCTTGGAGGATGGCACTGCACCCAATCTCCTGGCACAACATCTGTAAGTCAAAGGATACATGAGTACCAATGACAACCTCCGGTGGAATAATATGCAAACTATCCGTGGGTGCCGGAGTAGACCGACGGATAGAGATCTACGTATGAATATTACGTAGAAAAATTACGAGGGGGGAAGAAAGTCTCTGCCTCCGCCTAAGCTCACTTGTCATATGACTAAAAAATAGACTCCGTAGGGCCCGGAGTTCTCCGTATGGCCCGGAGTTAATTAATATTGAGTGAGCAATGTCAAACTTCTAACGAAGCAAGGGATAGTACGAGATGGCGGAAATAAAAACCCCCCGCAGTAACGCAAAAGATTCTAAACATT

>chr10:+:22792773-22796215

GTTTTTTGTTCGTTTTATGTGGATACCAAGAAAGTCTTGCTTAATTGGAAGAAAAATTGGTGATCATTTTGCACAAATAATGGTGGTGGGATTTCTGCAGTTTATGAAATTACATACTCATGGAAGCAGAACCTTTGGGGGACGCTCCAATTTTAATGAGCTTAATTAAGAATATGGTGCAACCTGCAAAGTTCTCCAAGAATTTGATGCATGTGTCAGAGGCTTATTCAAATTCTTCTAAAGAAGGAAACCAGTACAGAATTATGGCTTTGTTTATCATCTCGGAGTTTCTTTTGTCAGTGTCATCTTTTATTGTGCTTTATGAAAAATGAGTACTGTACTGTACCCTTTTTGTTATACACTTTGTACTGCCTTTGTCAAATGCTAATTAGTGTAAGTTGTATTCAAGCATCATCAATCCTCCTTGCTATGTATGATGATTTCTGTCTGTGGAGTATTAGTCAAGGTATAAGTTTTCTGAGTAATGCAAATGTTACATGGAGGTGAAAGTATACTGATGTGATTAATTCATTATATGCAATGCGTTTCATCTTGAATGGGAAAAATGGAATGGTTAGTGAAATGCGGAAATTACACAGAGTTCCTTGCTGGCAAGAAGCTTGATAATGTTTGTGTCTGTTGAGACTGACTTTACATTTTTTATCACATTACTATCTTAAAGCTTTTTTTTTTTTCTTTCTTTTTTCTTTTTTACCGGAATGCAAATAAACAGAAAAGGTAAGGCCTTAGTGATATAGAATATTCCCCGTGCTGACTCATTAAGTAATGTAAAGATTGTGTTCATTTTGTTTTATGTAAACAATTATGTAACAAAATGTGATAATTAGGTATATTAATTTTATCATTAGAATTATCAAAGCTACAGTATAAGATTTTTTGGCAATTACATACACATGTACGTATGTATATATATTTGGATATCAACATGCTAGCTGGTCACACAAGCCCTTGGCAGTCTCCAGTTTTGCATGTTGCTATTTATTCCTCGTATTTCCATATCATTTTTTCCCTTGCAAACTTACTGCTTTTGCGACTACTGTAAACTCAAGTCCTGATTTGGTGTCTGTTGTGGGTGGAAATTAATTTTACTGGTTTCATGTCTCTGCTTAGTTCTGATTGGCTTGTTATATACTATACTATTCACTTAGTGTATATATAGTATATATACATACATATTGTGTATGTTTATGAAAGTATATAAATGAAAACTTTAAAGCATGGCATTCTACAGTATATAATAGCTGCTGTATTCCGGATGTTATTGCAATCATTTTCATACCATTCATTCTAATTTCACAATAGGAAATGTATATAATGTATTTTATAATGATTAACTTTGTCTTTATACATTGTTAAAAAGAGGTTGGGAGGTTGTCATAACTGGCAGGTGTCTCAGTTAGACGGATCTCACGTTTCAATTGAAGGGCCTCACTACAGCCCATCACCAACTGCCGTGACTTAAGCTTAAACATTTATCAGGTTAAGATGAACTATTTTACATTGCAAATCATTATACTCTCCTTAATAGTTGCTTTGTCTTTATACTTAACAAAGTTCACATTTTTATACAGTACCATTCAGAGGTGGCTTCTTTTGAGAAGTGCATTGGATGTTGTTCATATCCCTGACTTGACGCTGTTCCTTTTTATTATTACTGAAGCTTTTCAGCTGGATCTTTTTGCTGAATTAGGTGAAAAGGTTTTTTTATGGAAATTAGGATGTGTATATCAATTCTTTTCTAAACGATCAAAACTAGCTTTCTGTGTACTGTATTGATTATAGTGGAATCTCTCAATTCAAGCATAGTTTAGATAGAAGCGTTTCAAAAATTTAAATAATGTTCATCTTCTTTCCGTTAGATTAGATTGTAAATGGTCTTTTGGGTAAATATTTTTATCGTTATTGAGGAGAAAGTAAATCTTTTTCAACTTTTCATCCTTTATCCATGTTAAGGATTCAGATAAATTCTTTTGTAATGCTGTTATTTTGATTATGGGATTTAAGTACATTTGTAATTACACTTAATTTTATTTTTGAGTTGGAGAGATTTAACTGTCATTTGTATGTTAATCATTTTTAGTTTGTATATTGTTTCATTTGAATTTAATTTAATTTTCCAGTTTATCAAAAACATATTGTGGGAGTTTTTCTGTTGGATCATAATGGAACCTTGTGTGCTTTTTGGGTTTGCTTCATTAGATACTAACTTCTAAAGGTACACATCAGAATAGTGAATGTGGTTTTTTATTGTTATTCTTATAGATTTAAAACTGCAAATTCCCCGCCCTACTCGTTGGTGTACCACAACTGTGGAAGCAGAAGTCATTGTTCTCCACTGTGATGACAACAGTCAGGATGGTGGTCATTCAGTTTCTTATAAAGATAAAGGGGCTCCAGCATGATCAGACAATGGCTACAACTTTTATTTATTTATTTTTTTTTGTGGCTTATTACCCCAAAGGCAGCTAATGCAGTTTTCTAAATATTGTACATCAAGGGGATTTCTTTCAGATTGGACCATAATGCCCCTTAGTTTCTCCAGTCTGCCGAGGGCACTTAATTCTCACAGCATCAAACGTAGAACAAAGAAAATATAGCCTTTGTTATTTTCCTTTTCAGCATATTAGACTTCTCTTTTTACAAGACTAATGACTAACCCAAGTTTTTGTTCAGTGTAGTTAGGTTTATGTTCATTGTACAGTCAATGGTGTACTAGTTTATAGAGTTAACTGTAGTATACCTATCATGTGATTTCTTTTCCCGGGATGGAGTTGGCCGTTTTGTTATGTAGGTTAATCTCTCGATTGTGGAATTCCAAGTATGTAGTACTGGATGTAACAAGCTATCCAGTACAGCTGCCTAGGGGTGAGTGGTCACTGAACTGCTGATGAAAGAAATAGTTTATTTAGATTTGTTGAGTTTTATTTTTAAAAAATTGTTTTGAATTCCATTTTGATCGTGAACAGTTTGTTTTCTTCTGCAAAAATTAGTCATGATTAGTCATTAATTTTTAAATATATATCTTTTATTAAAATTCTGACACTGTATAAAGTTGAGAATATATAACCACTGTATTTTTATTATCAGGATCTCTCCCTGTTCATCTAATGTTAAGAATTTTTTTTATTTTTTATTTATAGGTGAATAGGTTTTCATTTTTATATTTTTTTAATTTTCTGGTTCTAAGCTGTAGTTGTTTGTACAATCATTTCATCAGTCAGCATTTAGATCATTAAGGAATTCTTTAGTGGTATGGTATTTCATACTTTTCTGCAGACTTTTATCCGTTGCCAAATGTGAAGTAATCTGTAAGTATGGTTATTTGAGCTGTGCATAGCCTGTATAATCATTTACATTAGATAAAAAGGTTATATGCAAAATATTTGAGTCTAAGTTGCAATAATAAAAACACTTTTGCTCAG

>chr10:+:50106410-50110641

GGCAGCCATGAATGCAGCAGCAGGTGCTGCTGTGAATGCAGCCGCCAACGAACTCTTCTCTTCCTTTGGCTTCAAGAAGTAAACCTAGTTAATGCAAGATGATTATTGTTGAACTGAAAAAGCTTCATTTGCCACTAATGCTCTTAGGTTTTGAATATGGAAAAGTTGCTTATTTTTAATTTGGATATTGCTTTGAGGAATCACATGTATATGTAAACTTTATAACAAAGAATTGTACTGTTATCCCACATTTTTCAGGCTGTGGGGATTAGGTATCGTCATAAAGATGTTCAGTTGCTTGAGTATTAATCATTAGAGTTGAAAAGCAAGTTTAACACAGTTGCTGCTTGTAATGTTTTCTTTCAAATGTTATTTCCAGATGATGAGTTCTATGTAGAGAGAAAAATTTCTTGTAATAGTTTGTCTGTGTTGTAATTTGTTTTGGAAGTTCAGGTTGAAATATTTAGTTCTTGTTTTACCTTATATGATGCCTTATCAAGTTTGCTTTTGATGACTTGGTAAAACCTTGTGAATAACATTAGTAATTCCAATGAAGTTTAGTGTAACTGTCTTTTCACGAACTTCAGTATGTATTGCATCAGAGTGTGACTGCACATTGTTGGACAATACAGTAAATACAAAGTTCTTGGGATGGTCATTGAATATCATTTGTCTCAGCCCTAAGCTTGACTGAAATATGAAGGACAGGAATATCTTGGAATAAAGGCAAAAGCTCTGTTTTCATAAATAAGTTGCGTATGGGATCTAGGCCTAGGGCAGATTTGGTTTACTTACAGAAAACAGCTATTTTTAAAGGCCTGGGTTCTCTGCTGTAAATGCTCCTTTGTCGCTTTAAGCACTAATACCTGTCCACTCATGCTTGTACTTTTCCAGTTGTCTTCATGCGTGAATTTTTTTCCACACAATCTAAAGCCATTTTATTATTTACTGTATTTTTGGATTTTATACTTTATGATTTTGGTATACTCTGTTAAGCTTTTAAGTGTTTAAGGGTATCATGTATATAGCTGGATGAATTTATTTAATTCATATCTGTTTTACAGTTGCTTTAAGAGTCCAATAGATTGTGTGTTGCACCATAAGGGTTGTGTAGTGCAAAATACTGCAATCATTAAAATTTAAGACAATAGTTGTTGCACACACAACACAATTTCATATTGTTTTAGTTATGTGCAAGACTCTTATTAGATGCATATTTATTGAAGTTAACGAGGACAAAATGCTTTAATATTTATTTTATGAGGTTTGAACCTTGCAGCATAGCAATACAACCTTAGAGCTACTTCACCCCAGTAAATGAAGATGCCTTGAACAAAAACTTGCATGATATTTAATATCATTCAGAGTATGATATCTTGAAAAAATTTTTATTTTTATTTTTTTATTTATTTATTTTTTTTTAAGGCAACAAATACCTCTGTTGTTGGCACTTGGATTCAGCAACTCAATTTTTTTTTGGTTAATAGAAGGGTTATTGGATTGCAAGTCATTGTACTAATTCCTTTACTTTTGAATAATTTAAGTTTAAATATTTGGTTAAATCTTCATTAAATCTCAAAGTATTTCCATGCAGCGAATCCTTTCAAGTCAGTTTCCTAGTTTTAGGTAAATGAATTAGGAAATTAAAAAAAATAGCCTAAGATGGCCAGTTAATTTATTCAGGTTCAAGAGTTCCCACCAGTAGCTATAATAAAGTATCAAAAAAGTATGTAAAATGTGGATAAGGTGACTTTAATATAGACAGTTTGGTCTTAAAGTTGTTTATTTCATTGCAAAATTGTTCCTTTGATTTGCGTACTGTATATGTATGGTAAATGTAGATGAAGTAGGTTCATACTTGTGTAGAGAGAAGTTATATTTAGTGATGTAATTATAGTGCAGTAATCTTTGAATGGGAGTATATGCACTGGTGTTGTACTGCACCTTTGTTTTGGTGGCATAAGGAGATGTGATGATTCATTTTCCTGTCACTTGTTGCCTCTTTAATTATTTAGTAATGAATGAGTACTGCACAGTATTTGTTGACGTGATTATACCACATATGTGGTGCCTGTAGCTATATTGTAACGGGGTTCTTAGGCCCTGTGTGTATATATATATAGGATGATGAATATGGAAGAGTGACCATGTATTATGTTAATGCATTTGTACATGAAACTTCACATGATGTGCATGCTAATGTAAAATGACTCATTTATTGTATTACTGTGTTTATTTTCTTTTGTGTGTGTGTATCTGTCAAAGCCCAAGGCCCATCTTGGAAAATAGGATTCTGTTGCATAAAGTGAATTTTTTAAGTGAATATAATATACTGTACTACCAATAGGGCTGTATCTTTGTTGTTTCATACTACTGTATGTTGAGATTTCAGAGTTTATATAAAAGCTTGTAATAGAATGTGTGAAACTTTGTGTTATGGGGCTCATTTTTACCAATAGACGGATCATTCTTCAATTCTTGATGCTGATACTTAAAATGAGGCTTATTTCCATGTAAGAAATGGCAAGGAAAAGGCAGCTTCTTTGTGAACTTTGATGCTACTTGCTCTTTCTCTATTTTGCTGTGCACTAGAAATTAATGTTCATTATCAAGTTTAATAAGGCATTGAAAGAAACTGCTGTGACACTTGTAAAATTTAGAATCTAGATAAGTTATCAGAATTAATACTGTTGCTGTCTTAATGTAGGTGAATCGGTGACTTGCTGCACTTATTGGCTAGTAATAATTTGGTTTTTGGGTTGATATTTTCTTAAGTTTTTGATAAAGACTAAAATTGAACAGAAGTGCATTGCAATCCAACTCACCAAACTAAAAGCGAACAGAAGTGCATTTCAGTCCAACTCACCAAAGAAGTGGCTATCAGATGCAATAATATAAAGCATGTAGGCTCCTAGTATCCGGTTGGTGTCCCTCGTGTGTGCATAGTTCTCTACTCTTGTGGTACCTTGTTCTCTCATGGATATGTTTTGGCTAAATGAGAAAGCTATTTGGAGATGTTGCAAGGTTATCCAGAAATATGTTGCAGAATAGAGAGAAAACAGTGATAGATAGGGATCGGCTAGTCTACATATACTAGATAAAGTTACAGATGGTATGATTGTTAGGTGGTCTTTAATCACCTTGGTACACTAAGTTCCACATTTTATATTGGAATACTAAAGCTCTTTACCACCCATGTTAAGAAGTTACTAGCAGAAGTGGGTACTTGGCATACAGGCAGCCTGTTGTGTACATTTAATTATAACCCAGTACTTTGAAGTGCCAGAGCTCTGCTTGAGAACAGTAGCTGTTCTCCTACAGATCATTGAAACCAACAGAAACAAGTGGGTTTGCAAATTGAAACATCTACTATAACCAATAACTCAGTTTTGGGAAATTGATTTAAATATGGAAATAATTTCTCCATTATGTGGGTATTTATATCTTTTCATTCCACTCACCCCCCAAGATAGTAGGAAATATGTAATATAATATGTGAACTTTAAGCATAGAAATGGGATAATGGTGTTTTCTTTTCCCTTTGTGTAGTTGAAAATGTAGTTTCTATTTGTACTGTCTTGAATGATGACTTTCAAGGCGATATTGAGCGTTTTTGTGCTTTGTTTCTATTGTGATATGAAAGTTGGCACAATATTCCAAACTCCAGGCAATTCTGTTTTTTCAAGGATTACCAAACTATCCATTAAGGTTGAAAATAATTTGGTTTTCAGAAAAAGGGTTATAAAAGTGTCCACTAATTAGCTGCAAATTCAGACTGATAAATCTTTGTATGGTGAAAATAACCAAGTAATTTAGACCTTTCTGCTCCTAATTACTTACATTTTGGAATGTTATTTCCATGCTTTAATGATATTACAACACTAGACATTTGCATGTGCATTTTCAGATCACTTGGGTAATTATGCTCTTGTCTGGACCTAATCCAAGTTTTTATAAGTGAACAGTATGCAGTAATAAACAGTAGATGTTTTCATTTTGGAATACCTTGTGTTTGTTAAAATATACTACAGGCAAGCCAGAGCACACTGTTGCCTTTGATAAGAGAAAAAGTATCTCTCCCTAACTGTCAAAAATGCAAATACATCTCAAGCAGAGGGTTTGTTTAAGAGTCTGGAACTCCGGAGTTTGTGGTTGTCCAGGGTGTTTGTCCTTGTTACTCTGTGCTATCTTACTGTTAAACCAAGCCACTGTAAAATAAAGGCCCTAAACTT

>chr10:+:87313506-87314806

CATTAAAGTTTATCTTAAAATAAGACTATCTCAAAAGCTCAATCCTTTCGTACTAAAGCCATTTTCAAATTAATAAAAGATAGAAACCAACCTGGCTCACACCGGTTTGAACTCAAATCATGTAAAATTTTAAAGGTCGAACAGACCTACCAGCTAGACGGCTACACCTAACTGAAATTTTAATTCAACATCGAGGTCGCAACTACTCTTGTCGATAAGAACTCTCAAAGAAAATCACGCTGTTATCCCTAAAGTAACTTATACTTTTAATCCACAAAGAAGGATCACACACTAATTATAATTATTAAATTAAATAGACAGTTACACATTATATCTACATCTCCCCAACGAAATATAAGCCTAATAAAGCTACTTTTCACTCAAATTGTAAGCCAAATTAAACTTATATAAAGTTTTATAGGGTCTTATCGTCCCACTAAATAATTTGAGCCTTTTCACTCAAAAGTTAAATTCAAATTACATAAAGGAGACAGCTCATCCCTCGTCCAACCTTTCATTCAAGCCTTCAATTAAAAGACTACTGACTATGCTACCTTCGCACGGTCAAATTACCGCGGCCCTTTAAATCAATCAGTGGGCAGGCTAGACTTTATAACTTATACACACAGACATGTTTTTGATAAACAGGCGGAGATAATATTTGCCGAGTTCCTTCTAAACGCCTTCTAATACTTAAAAACTTTATTTTATTTAAGATTAAAAAAAAACTCTAATTTTACTTATATAAACATTCTATCTTTCGCTTAAACAATAAACGAAATTCTTTACTAAAAACAGCAAGATACTTTAAAAATATAAATACAAATCATTATTACTTTAACGTTATACCAAGATGGCTGACTTTAAGCCCACTTCTAATGACTTACAATAAAAGTACTTAACAAAAACAACAGATAAGAAGCCTCTTCCACCTACCTTATTACCAAATTACAGTTAAACTTGGTACAAAATTAAACTCATTTCGTAAAGAACCAGATATACAAAGACCGACTAACATATCACCACTAAAAACTTTTTCAAACTACTTTATAACGTAGAAAAGCAATAAAATTTAGCTCTCTCTGTTTCGGGAATTTACAAATAACGCAGCTATATAGTAGACCCCTGATACAAAAGGTACGAAAATTCAACACTACTTTAATAAAATATTAAATAATTCCATCACTGTACTTATTAACTATAATTTCTAAACAAAATTCTTTATCATACTAAGAAACAAAACTTATTAATTTTTTATAAAATTTAACCACAAATCAATATAAAATAATAACACTTAATTT

>chr10:+:87952957-87954001

TTGAAAAAGTTTTTAGTGGTGATATGTTAGTCGGTCTTTGTATATCTGGTTCTTTACGAAATGAGTTTAATTTTGTACCAAGTTTAACTGTAATTTGGTAATAAAGGTAGGAGGAATGAGCTTCTTATCTGTTGTTTTTGTTAAGTACTTTTATTGTAAGTCATTAGAAGTGGGCTTAAAGTCAGCCATCTTGGTATAACGTTAAAGTAATAATGATTTGTATTTATATTTTTAAAGTATCTTGCTGTTTTTAGTAAAGAATTTCGTTTATTGTTTAAGCGAAAGATAGAATGTTTATATAAGTAAAATTAGAGTTTTTTTTTAATCTTAAATAAAATAAAGTTTTTAAGTATTAGAAGGCGTTTAGAAGGAACTCGGCAAATATTATCTCCGCCTGTTTATCAAAAACATGTCTGTGTGTATAAGTTATAAAGTCTAGCCTGCCCACTGATTGATTTAAAGGGCCGCGGTAATTTGACCGTGCGAAGGTAGCATAGTCAGTAGTCTTTTAATTGAAGGCTTGAATGAAAGGTTGGACGAGGGATGAGCTGTCTCCTTTATGTAATTTGAATTTAACTTTTGAGTGAAAAGGCTCAAATTATTTAGTGGGACGATAAGACCCTATAAAACTTTATATAAGTTTAATTTGGCTTACAATTTGAGTGAAAAGTAGCTTTATTAGGCTTATATTTCGTTGGGGAGATGTAGATATAATGTGTAACTGTCTATTTAATTTAATAATTATAATTAGTGTGTGATCCTTCTTTGTGGATTAAAAGTATAAGTTACTTTAGGGATAACAGCGTGATTTTCTTTGAGAGTTCTTATCGACAAGAGTAGTTGCGACCTCGATGTTGAATTAAAATTTCAGTTAGGGGTGTAGCCGTCTAGCTGGTAGGTCTGTTCGACCTTTAAAATTTTTACATGATTTGAGTTCAAACCGGTGTGAGCCAGGTTGGTTTCTATCTTTTATTAATTTGAAAATGGCTTTAGTACGAAAGGATTGAGCTTTTGAGATAGTCTTATTTTAAGATAAACTTTAATG

>chr17:+:68628802-68631703

GGAAATTTTAACTTTATTCAGTTATCAAACTTTCACCATTTTCAAGATGAACACAGTTTTAAACCTACAGCTCAGAAAAGTGAGCAATGGAATGATTTTGTACAGGGTGCAACACACACTTTATGGTATCAAATGAATCAAAGCAGAAGCCAAAATCATATAAATAACCCACCCTACACTGTACTGCAACTATTTGTAGAATGAAAACTACAAAAATACACCAAAAGGCCAAAATCACAACTTGAATCGATTAAAAAATCTGACTTACTGCAAATTATGAAATCTGACTTACTGCAAATTATGAAATCTGACTTACTGCAAATCATAAAATAGATGTCCACTTTCTTTGCAGTTTTCCAACAAATGCTGCTGAAGGATATCAAACAAAATATAAAGCGCAATATGAAAAATAATCACATAGTTGGATGTTATCTGTACACCATTATGAAAATTAAACAACTACAATATACCAGTCAAATGAGGAGTAACTGCTTAATTAGTTGTAAAATATCTTGTAAATGACCAAACTAGAGGTATCTTCAATAAGGAAATCAGACAACTCCAACATTAAATCAAACTTAAAAGGCAATACTATACTTGCTTCCATACCACTTATTGCATTTCATCAAAGAAGAGGACTAACATTTAAATTTTTCTAAAAGATATTGTAAACAGTTTTACCCTGAAAAAAGAAATGTACCCTAATATTGTAGGTACATAATACCAATAGTCAGTTATCAAGTTTACAAAATTTGATTTGGAATACATGGTAACTCCTCAAGCTTTTGATATTTACTTAATTGGTTAAGAAAATACGGTTCAAAAATTAGTGCACAAAATCAACAATATACTGTACTAATAGACAGACATGACCATTACAAATTTTAAGACTGAAAACTTAAACAAAATAGTATTTATAGCCAAATATTTGATCAAACTAGAAAATTTCTCTATCAAATTCACAGATTAAGATATGGAGACCTATGTTATATAAAAACATCAAGTTTTTTAAAGTCTTGCTAAATGTAACCTAAACTGTTTGTGAACATGATTCTCTTCAATAGATGACTCTCTGTATTATAAACAAAACCTATGAGACTTTTTATATCAATTTACTGTACTTGACTCATAGAGTGCTCATCACTTCACTTTGAAGCTAGAGTATACTAAACCCTTCTGCCATCCCTAGATTACCTGCTTGACATTCATAACTATGAATCAGAGAATTGCTGAAAAACAGTAAAAATCATATTTTCAATTATTATGTTTTGCACATTTTTTTATTTATGTTATACCATATCAGCTTATCTTTTGTCCTATTTTACACTATAGCCTTCACTAACACCACGAATATGCTTTTTAAATTTAAGTATATCTCTAATAGACTATTACAGGATTGGAGATGACTGTAAGGGTAGTTTAATTTTTTCTCAAAATGGAATTCCGATTCAAATCTTACAACTTTGCAAACTATAGTTGTTCAGATAAAAAGTTTGTCAGGATCACCTACTCTAACAATACGTACTGTCTATCATTCAAGAGCAACTAATCAATGTTTTGGCATGTTTTGCAAAACTTTTCAATGCATATAACAAGAACAATGAAATAAATTAATAAATTTTGAAGTACTTTATATGAAAAATACATAGTCTAGGCCCATTAAACTGAAGAACACTTATGGCATAACCTGGAAATCAATGACATTTTGTCGGCAACATTTCATTTCATTTTTTTCTCTTCCATCCATAATTTACATTTAGTGAGAAAAAATATACAAAAATACAGTATATTGTATATCTGTTAGTTAAGCACTTGATTAATTAGTAGTGTATAATACATACTGATATTTGACAAAACATTTTTAAATCTTAGACTAGATAAATAGTTTCAATATTTTAAAACTGCCACAAGACAATTTATCATGTATTCAATATCTCATATCAACTTTCTCTTTAATACATGCATATTAAATTCTAAAAAGCACCACATCAAAAGTAAAAATTATGAACAAAATACCAGGACAGGATTGTATTGCACATTGTAACACCATTAAAATGAAAACAAAAATAGAAACATTATTTGAAAACTGAAAATTTTGCTCATACAATTATAATCTTACTTCATAATTTCCACTGTCACCCCATTCCTAAATGACAGTCTAGCTTTTGAAATAAAAATGATAACCAAGAAAATAAGGTAACAACTCAACATAAAACCAATGAAAGTATGATTTGTATGCTGCAACCCTTTGAAAATTACTACAATGAAAATAAAGACAGCAAACTCAAAAACTACAGTTTACTGTCAACTGTACTACAATATTAAAACTAATCAGTTACATATTGTCTTGCCAAATTACGGTGATGCATTACTTGGCTTTCATAAGCCTTACTTTGAATAGTTCTTCCAAAGTATGTACATTCTATATAAAAAAAAAATTGAGTCAGGTTTCCACTCCTATTAAGATATTCAAATCCTTTTCATAATTTGTATAAGTTTAACACTGAATCATTAGTCTGATTAAAATTTTCATGAATTACTGTTGAGCACAATTTTAATAATGATATTAATACTGAATGCTGAACAACTCACTCATTCAGCAATTCTTTCTCACATTGACTGATTGGGATATCATATTTAGGCCAAAGGCCAACCACTGGGACCTATGAGATCATTCTGCAATAAAAATCATGTTGAAACTTGACTGAGTTGTGGACATGTGCAATAATATGCAAGAGTTTATTCATGACTGCAATGTGTACTAAGTGTGTTGATGGAAATGTGCTTTCAACAAATCAGTAAGCCATGACAAATGCAATGTATCTTTAACATGTTTTATCATTTTAATATCTTACCACAGCAAGTACATCATATAAAAGGGTGACAGTTTCTTTCATCTTTG

>chr17:+:74098479-74101084

GTCAGTCGGCAGGCAGACGCGGTGGTGATAGTAGCTCGCTCTCTCTCTCCTGGAGTTCTCTCTCTCCCCCAAACACTCCCTCACTCTCCCCAAGACCCTGGAGGACTTGTACACGCGCGTTCATTTAGCAAAACGATTTTCTCTCTCGTCCTCATTTTTTATTTCTTTCCCTTCCTCTTTCCCTTCTCTTCCTCTGGCTCTCTCTCACACTCTCCCTCTCTGTCTCTCTATCTCTGACTGTCTCTTTCCTGTTTACTTCCGGAGCATCTTCGTAATCACATGACTGCTGCTCTAGGATGACAGAAGTGATGCTACGGAAAATCAGGAAGCTGGACCTGCGGTTATGGTGACCAAGAGACAAACTCTGGAAAAGTACAATGGAGAATCAATGCTTTGGGGCGTCTAGTCTCGCCTAATAAGGATTCTCGAAATCAACGGTTTTTTTGGCGGAGGGCGTCGCGAAAACATGCTTTTGTCGATCTGTCCGTCTAAAGCAGTTGGTGAATTGACGTGTATACGCTGAGTTATCTGTAGGAATGAGACTGCTTCCACCACTCAGACATTAGAAAGAAATATTTTCGCAAATCGGAAAATTGTCGAATACTGAGCGAATTGGATTATAGTGCCGTGTGAATGACTACTCGCTTCTAGTGACTGTTTGATAGTTAGATAAATAAACCTAGAGATGAATAGTGCACCTTGTAAAGTAAACTTTCGATAATAATTTTTGATAGAGACTTTACTCCTTAAAGTATCCTACCAGGACCTTCTAGCGATCACAATAAGTGAATTATAGATGTTAGTAATACAATATAATTATCTCAAGCGCAAATCTTCCACTGGGAAGATCCGTGACTCGTACATCTCTGCAGTCAGCAATTAAAAGAGTATTTATAAGGCTACACAGTGGAAATCACAGTGAATTGTTGACGTAGATTTAGTTACCGATACAAGAAGAAATTCATCTTGGAATCTGCTGCAAAGGAAAAGCAAGAAGGACAATAAACAAATATTAATAAAAAAGGGAAACTGGTTTTCGAAGAGGACGTCTCTCTCACCCGGCACACAACTAGTGTGTTCGCATCCCTTTGTGGGTCACGCCCCCTTTTCTCTCTCTCTCTCTCTCTCTCTCTCTCTCTCTCTCTCTCTCTCTCTCTCTCTCTCTCTCCGCCATGAGCCTTCTTCGAACCACAAGGGATCAATTGACTTTGCAATGCCAGCAGCAGCAGATATGTAGGAATATAGAAATGAACAGCAGCGGGAAATTCACATCGTCTGTCACGAGGTCTTAAAGAGTGAAAGAAACCCGATTCCATGATAACCACTGTGTCAAAAGGCCCTGGTCATCACCTACTTTATCTTCCAGAGCCTTCGGTGTTGAGGCGAGAACATTTCCATGTACTGTGATGTGGATTAGTGTTTGTTAGTTCCCTCATTTTCCCTTCCCTAAGAGATTCTCCTCTGCTGCTGATGCTTTGGCCTTCCCAAGAAGCAAGAGCCTCATGATAATGGTATTGATGACCAAAGGTCAGAGTCAAAAGTATCAAAAGCCCAAGTGCCCCTTTCGCCTCACTTTTCCCCGAGTCACGAGAAGGACAATAATTCTTCACGAAACGAAGGCTTCCATAACGCCTTTCTCTCTCTCTTTTCCATGACGTAAAAGAGAAGAACATTGATGATACCCAACCTTTGGCTTCCAGGTCGGCCTTTCTTTTGTATATGAATATTCATAGGTGATAACAAAAAGAAGAGAGACAATTCTTCCGTTCCCTGTCTTGTTCCCCAACGGTGAATCCGAACACATCCAAACGTCTTCCAAAAAGATCGTCTTGATGGATTTATGATCCTTTAGCTTTACGTCGACTCCGGAGGTTAACTCGAAACAGGCGCCGCCCATCAGCATTCTCTCATTTCCATCAATCAAAACGAATCAACAAATCAGCTTCAGGAAATATCACTCCATATTTGATCGTTATCAAAACTAAGTAACGTAATTTTGGTCATCATAAAAATTCAAGAAATATCACTCCATATTTGATAATTACGCGAATACATGATCTGACGTCGTCGTAAGTTTGATCGTTACACGAATAACAAAACTGTCACTCCATACTGGTAATATTAAGAATTGAAGAACAATTACTCCACATACGGTTATTAGAAAAAAAAGGCAAGAGATTTCGAACATTCATTTGAGATTAAATCCCCAACGAGTAAATCAAGAGAGAAAATATAATGAAAACTATGAGGAAAAACTCCAGTTGAATGATCTACTGCAGAGTGGAAAATATGATGTAACTACACAAGAAAAAGAGAAAAAGTGCAAATCGCGAGAGGAACAATATTTGTGAAGCTCGCCATCCAGCACCATCTGCGTCAGGTGCTGAGTCAGCACAAGTCGTGAGAGTCTGGGTGTAGTAGCTCTAAATCTCCGTGGAAATAAGGAAAGAAAGAAATAGAGAGCAAAAGAGAGAGAAAAAAAGAACAACTCATTTCTCCGCCAGAAAGCTGTTTTGTTTACCTTGTTGAAGGTCCACTGGAAGGAAGGAAGGAAGGTGGAAATCGAAGGTCATAATAACTGAAACACAAAAGCTTTCGTAGAAAGC

>chr17:+:81253555-81256565

TAGCATTTTAATAAAGCGATTTCCAGTGAATAAAAATTCTTAACCAAATAGAAACTCATCACACAGCTCTAGTACCAATATCTATACCCAATTTTTTAAGTTACTACAATACTTTTAGGAAAATACACTAACTCAACCTCTACCAAAAGGAAATGATGAAATAATTACCACAACCATCATATTAGTTCCATTACAAGTTCATTGGCAATTCTTTTGAAACCAACTTAAGAATTTAAACAAATTATGTTCCCTACAATAATCTACAACTGGAAATTGTTCTCAAAACCTGTCTACTTCTAAAACCAATATGGACATCCTCAGGTGCAATGAACCAAACAAAATTATTAATGACTAGCAAATAATATAAGCCAGACTAGGCAAATTGCACATACAAAAGTCATTAGTGAAACTACTGAAGGGAATTGCTTAGTCCACAATTGCAAATATGCTTCACCTTATAAGTACTACTGCCATTCAGGAACATAAGAAAATAAAAAAGGGGTAATTTTGTTTGGTCCAAACTAAAAAAAAATACTGATACATACTCAAGACTGCAAGGAACAATTTCTTCTCTGAAGTATCCATAAGTATTGTAGGTTTCAACTAATGTATAAAAATATACTGATTTTTTTTATTTATCCATTTATTTAAATTGACACCCACAACCTTTTTACATTTATGGAAATCTAACAAAGCCCAAAGAAAGCTGTATGTAGAAATGCATTTATGATCCCAAATAATAAACTGGAATGCTAAAGCTATGAAGAAATTTACCAACGTGGTCCTCTTGTGGAAAGTAAATTAACTACTGTCAAGCAAAACTGCTTGTTTTACATGGAAAGCAGTATGAACTTAAAAGAACATACTAAAACTTCACATTGTACAAATTAAATTTTGGTAAGGCTTCAATGTACCAGTACCTGAAATCTGTGAAGCACAAATGTTAATGGCATTTCTTGTTCTTGCTAACCAAAAAGGGAAGCAACTTTTCATAATTTTCATTATATTTACCTATTCTTAACAACTATAAAGCTTATAGGTACAGTATAAAGGTGGAAATATTTAAGCTTTACCCCCAAACATTCACCAGGGCACAAGTCTTAAAGAACTCACTATCATCTTTCATACTGTGTAATCCCCAAGTTTGAGCTACTAGATGAATATACATTTTTCTGTATAACGTAGAGGAACTTGAACTTAACTGAAAAAATAAGTTTAAAATAAGAGCGGAGAAAATTTGCTTTGCATATTTTCTGAACTACCTACAAGTTACTTAGCTCTTCAGATAAAATATAATTAGCTATCTCACCGACAATGAACTATCCCTTTTTTGTGCCCTGTGAAAGACGACATAATTATAAACTGCATGTACTTTCAGTCTATGCATCACCTCTAGTTTTCACAGCATTCACTCACCTTACATAACGATATTTTCAGAGACGAGCACTTATTCATGGCCAAAACACCTATATTCTATTTGTTACTGAAACCTGATTTTTAATCACAAAGAATTTCTTTCCAGTATAAAAGTACAAGTTCTACTTCTAATTCATCACATATGCAAAGGGAAATAAATTATTGATACCTGAAACACTATCTCATAAGTATCCACTGACACTGAGGGCCCTGACAACTTTTCAATCTGATACATGCACCAAATAAAAAACTGCACCAAATACTTTTGTTTTTTGAAAGACATACATATTGCTATTACTAAAGTTTTCATAACTTTTGTTATATGTAGTTGAAAGAGATTTGTACTCTGATGTCCATTTCGTAACTGAATAATTGCAAAGCTTCATTAAATTTTTTACATCTTAAACTTGGATCTCAGGCCATTAACCAGTAATATCCGCCAGTAAACATGAAATGTCAACTTAACAAAAGAAAAAAATTTAAAGCAGAGCATTTAAAAGCACTTTAAATTTATCTCACTTTTCAGTGACGTGATACTTCTATTAAGCAAATTATACATTTCTTACTTGAGTTTTTAAGGCTTGTTAAAATGTAAAAGAAATATAGACCAAATCAAGTCTTCCACTTTCAGTTTGTCCATAGCAAGCGAATAATCTTCTGCCTTAAGACTCGACAGTTGTGACTTCCTATAACTATATGAACTGTCAAAACTCAACATCATATATTTGTTAATTCTTATATTTTAACAAGTATCATCTAAAGGCCACTTTAATGGGTAACAAAAATATCTATGTTCGCTAGTGTTATGCAGATAAATTCTACTGAGAACAGAGTGGGTAGGTAATGAAAAATGCACTAATGTTAACACTTTAAACATTACCTAAAGAAGAACTTCATGGTAAGCATCAGACTTTTATGATGATTTAAAGAGTATAAAAGCTGAAGGAGGTAAGTTGACTTCTGTACTTATTTATGTAGCCTTAACTTCTGCTAATTTTTATTTGGTTTTATTTTCATTAAAATCTTTTTAAATATTCTCAAAACATCCTACACCTAAAGAAAACTTTTTCCACCTTGAAAATTCCATATCTCTATTACTAATCAAGCAAAACAATTGCAAGCCTTAATACAAACCATGTTAAATTGTCAAACTACATGAGTTTCCTTGCCCACAGCATATTTCTAAACATACTATATAAATATAACTTTGCATAACTTCAATGTAAATGTCAGACATAAAAGATTTCAACTATATTCCAAATACAAAGCATGAACAAATGGCAACATTTTAATTTTAAGCCCTATGGAACCAATCTAACTTTACATGTAATCCCCTGATGATTTGAGCGAGTTATATACAAACCCCACGAAGATATCCTTGAAAACCCATTTTACCATAATATACAGAAAACAAGACCCATGCAGTCACAAAAAGCTCTGTGGTCTGTATTTCTCAAAATAAACAATGCATAACTGATAGCTAACCTTGATAGTTATCAAATACTTAACAAAACAGCAAAATTCCTAAATGTTAGGTTGAAGTACCATGGCACTAACCAATTACCACAAAATTAGTACGTCCCTTATTTCTGTTC

>chr17:+:87900838-87904216

ATGCATGTAGTATTTATTCAACTTAGCTTTTATAAAAATTATCCCAATACAGAAAAAATTAGACTTAAGTATCGTACAGAGGTAATGATGATGAAGCTTGAGCAGTAATGACATTTAATAGAGCCAAGTTTCATTCCTTGATCTCTGGCTTTTCTAAGAGTAAAATTTTAAGAAATCCTCTTTGATCAAAGCAATGTTAGATAGTTCTAAACAAGTAAGAAGGAAAGAAATATCAAAGTTTGCCCTATAAGTACTTGTGGAGAAGTTATGCTTGCAATGTTGTATCACAAAATGTCAGAAGGATCAATACTTGTATAATCTCTTTTTTAATACCATCACCTTACTCTCAAGCCAGTTATTGTCAAGAGCATTTATAAGAATGCAGGTCAAAAATACTCAAGACACAGTACGTATAATGAGTACTTAGAAGTTCTATATCAACAAAAATCAAAAGAAAAGTAAGAACCCGGCCATTTGTATAAATGTACAGTATAAAAATTGGTTTGAATTTATATATTAGGGCTTCAGTCACTCTTTGGGTTCGGTATTTCACCAAAAAATGAAAATATATAATGTTATTCATCAAGTACAAAACAAATCTACCTCAAGTTGATTTTGTTTATTACCTTCTGTAAGGAGCAAAAACACAAGTGAAAACAAATATTTTTCCTAAAACTGTTTTACTTTTAGCAATACTGGTTGCATATCAAGCAATTACTTTCATTCATAAGAATAAAATTATTCAGTGAGTTAAGATTAAAAAATGTAAATACCGTACTTCTATGGTAATTGGTTGGTGATCTAAAAAAAAAAAATCTGAAAAACTTAAGGAAGTCTAAAGAGATTTTTATTTGTACCACAAAAACTCGATTGTGCATTTATTTATTTCAAATACACACCTGTGTAGATGACATGATACTATGAGTCATATACCTCTCATTTTACAGTGTATTTAGTTGAAAAAATCAAATGGAATGCAATTTACATAACACTTTACATTAACAATAAAGTGTCGTTTATCAGTACACCAGCAGGTAAAATGGTCAAACTTATTGTAGTTACAAATGAAATCATTAAAGGACAATTCAGCTCGTGGACAAGTACCAAAAAAAAAATAATTTGCAGCACTTATTACATGCCTTAATGCCCGTTAAAACTGTCTGATGTAAAAAGTTCATAAGCAAGAATAAAAGAAGGAAGTATAAAACGTATCATTTCATAAAATTTTTAAAATCTTGAAATGAGATGAAATACCTCACAGGAAAACTTACTGACAATCACATACAGTACACACAAGAAGTGTGTATGTGAAAACTTTCCTGTACTAGGTATTGCCCCCACTCTATAAACTATCTCTGGCAGTTCTTGAGCTCTACATTACTACTGTACACACTGTGTAACTCTGATAAAATATAAATATACAGACTTCAGTTAATAAGATAACATATGCAAGTCATCACATTTATATTTATATATCACTAACTCAAATATCAATGAATTATGCTAAAAAAGACTGGTTAGATTCACCTCTTATAAGTTTATATTTATACAGATGCTGTACATGTTACACAGTAGCTTTCAAATCTGTGCCAAAAGAGTCTAATTTCTAAGTGAAAATTATTCTCTCTAATTATTTGGTCAAGTATCTGAATCACAGAACCTACCTTAATGAAGACACCCACTATTTGTTACAAGAGTCCCCTTACCATGACTACTAGTCACTTCAATAATAAAAAGTTTGACACTGGTTAACATCACTGAAACTATTTTATCCAAGAGTATATTAAAATATGAAACAGTAATGAAACTAATAGTCATACCCTAAACAAGATATTTTACTTTTTGTTCTCTCAAAAAAGCACAAAGATGACAATGTGCAAGGGCCACATAAACCTACATTCTGTGATAACAAAAGATCAATGAATTTGGGAAAATGTGTAATAAATCAAGGTTGACTTACTAGGTTTTCCAACGAATTTGAAAGTCATTGTCTACAGAGATATGTAATGCAAAATGGGTATCCTCAGTCTTCAAAGCATTATAAATCAGTAAGTTCATCAGAAGCTGGTGATGCTAAACTTGGTGCCAGTTCAATGAAGTCATGCAAGTGTAAGGTAATAATGGATTAATATTGCATTATCTATTCACATTAACAGTTGAAGCATTCTCAGCACTTGCTATGCGATAAAATATTCTAGACTTATGTATCTTACACATCCTAAGGGAAACAGCAAACTTACATTATGCAAACTTTCTTGGAACAACTCTCAGTCCAAGTCGTGTTTACAGTATATGCATTCTGTAAATTACATCCACTGGATAAAACTAAGCTGATACTGTTTCCCTGGCACTGCACTGAACAACAGCACAATGCCTTTAGTATATCCAAAACAACTCTCTTTAAATATACTGATAAATATTTCATATGTAACAGAACATATATACAACCACTAATTATTTTAAATAAAACAGCACTGGTTATAAATGCATCATCAATCAAAAATCATGCTTGTCCATAAAGAAAAACAAAATACACAAATAAGTGATCTGCTAATACACAAATGCTCTAAAGGTAAACTGGTCCCAGGAAATAATGAAAATGTGTTATATATCTCTCTCATAACACAAGGGCCCTAACTAATATGACATTGAAATAATTGCTGTAGTTAAGACACTGAAATAATTCTTGTAGCCTAAGACATTACAAAATGCTTAAATGAGGCTTCACAAAGTGCTCTGCTTACATACGGCACAGAACTAATGATACATACCTAAGCAAACACTAATAAGACAAAAAATTGTAGGTTGACATAAAATGGTTACTATCTGGATAGCAATATATAGTGTATTTTGAGTAACAATAACATTACCATAATTCAAACTCAACTAGTACATACTGGCACTACAGAGTAAAAATATCACTTAGGACTCTGGCCAGTAATTCTTATTCTAAGATAAAATAAGTTGTGCAAAAAATTTTCAATTCAACTACAGTATAATAATCAATTTTATGAAAGAAACTAATTTCTAACCTGCCTTAACAAAAAACAAAATCTTAAAAGAATTTGTTACAATATATTAACTGAAACCAATAATTCCCTTACAACAATATTTATCTCAGTCTAGTCATTACCTACATCTTAACTATATCTGTACATGCACCTCATAATTTCCTTTCCCAATGAGTTATAGCACAAAGAAAGGTTTAAACCAGAAGCATAATTCAAGAAGTGGACAATTAATTGATACATATTAACCCTCTACACTTAGCAAACTTTAAACAAAATGTCAACTGAATGCAATTCAAAATAAATCTGCAAAACTATCTTACATCACACATTGCAGAGAAAAATTACTCCTGGATCATCTTGCGTATCAT

>chr16:+:105052-107633

ATATGCTAAACACAAGTTTTATTATACTTTATAAGATATACAGTAGCAAATAATTTTGCAATTTTAATAAATCAGTTACATTATTTTACTTTACATAAATTTCATGGTTACCAGTCAACTAACCTTGCAGAGCACTTGCCATCATCATCTTCAATAATGTAAAATTAATAACAAAACACATGAGTGAGCCCCCAGACCATATATAAGAAAGTAGTATGCGTAACACAAGTCAACGAACATATATGGGGATGCACTAAAGCAACCTACTGGTATTCAAGAAAACAAGATTTTAGACAAGCAATCAGCTAAAATCAATAAGAAAAGTGTTAGTGAACTATAAAGAAAAAAATATTGCCCTTTGGAAATGAGACTATTCCTGAATTAAATAGAATAATGCACTTTATATCTGCAGTCTGGCTAAACTACCTAATAAATATTTAAATAGTAATGGTTTTTCTTTTGAGACTGGTAAACTGGTATACTTCCAAGAGTCAAAAAAGCAGATATACTGGATAAGAAACAATGAAAGATTACATATTCAAATAGCAAAGCATTACAAAAGTTAGCTGATTAGCACCCATACAAAGTCCTGATGTGAAGTATGAATACCCTGTTTAGTTTTCTTTACAAGCCATGGAGAAATCATAACTAAATCTGTAAGGCTCCCTTTATTAGTGCTAAAAATATCATTATGACAAGACCTGAAATGCAGCTTCCCCCAAAAACTAGGAGTAAAGAATGTGAAGAAATGACTTTATCAGTCATCGATTACTGCCACTAATTATAGGAACATTCCAACACTGAAAAAACATATCAACTGCTATATATCACATCTGAGTGTAAAATTGAACAAAAGTGAGAGAGAGAGAGAGAGAGATACTGTACAGTACTGTAATTTACTATTTGTAATATTTCATTTGTAAGCTATACAGTATATTAAAAATCAATGACTGCATGTTTTCCGGAAACAAGCGGAAGTATTTTAAGAGTAAAACTTAAGTCATTGAAGAATATACATTTCATATTAACTATTATATCCTAATTAGAAATATAAATATCAAAAGTTACAATTTGAAATTATCACAGGCTATATCAAGCCTTGCTCATCAATAGCTAGGTTCTGATTTTCTGCATCATAGATAATACAGCAAAACTTCAAACTACCAGGACTTAGGGCACGTAAGGTTACAGCTAAATTTCCCCAGATAGATTCTCTCCCGTTACCACCTTTTATTCAGTTAAAATGATGGGCTCAAATCCTACTTAGTAAAAATGCAGAACAGCAAGGTACAACATCATACAGAGCAAGGATAAGCAACACATGAGTGAAGCATAACCATACTAAGTATTGAAAAAGATTCAACACTACATTTCAAAGAGGGAGCTCGTAAGGTTTTCAAAAATAATTCTGACATGGAGAATAAATATGGTAATGGAAATTCCAATGAAGTGATAAAAATGAAAAGAATAACTGAATGAACTCTACAAGTTTAAGCATTACCTTTCCGATAAGTTTCAATCAGATAATAAACACCAAACATGTCAGAACCACATAGTACCATGTACCCTTTATATAGGTGGAAGGCTGAAATATTATAGCTAAAATGTTGCTTTCCAAAACTTCAATCCTGAATTTTAATTTACCCACATGCATAAATAATATCCATAAAGACCTGTTTGAATTTTAATTTACCCACATGCATAAATATAATATCCATAAAGACCTGTTTGGTGTTACAAGCTATCTTATGGTATTAAACAATACAAACTAGTAAATAACCCATAAAATAAAATAAGGCACTGTACAACAACCAGAACAGCTTTAAAAGTTGCTTGAGAAAGCACCCTTTAAAGAATTCGTAGTACTATACAGCAAAATAAAATATTCTTAAACGGTACAGAAATCTCAAACGTGAACAAAATCCTACATGTCCAACACGAGATATTTTGGATTACATTACAGTCTCTATAATCGTCTATTAGAATTTGTACATTTATTTTTTTGATTCATCGCTCAACATGAATTAGCGATGACTCCAAAATCTAGTTTACAGCAGATTTACAATACATACAGTGCAAGACTGAAGTTACTCCATCTCTTATATCTCATAAATCCCAGACTAAAGAAATATCACTGACTAGGATGCCTGAGTGTAATATCCCATGTAAAAACCTTTGGACCTAAATGGTGCTACTAAAACAGGAAATAACAAATAAAAATGCTTCCTTGAAAATGATAGTATACACTTTCCCTTCAAATTTTTAATTATGCATCTCTTCGCGACTTTGACAGTTTTTAAAGCAAGGTATAAATTTAAAATGTGCAAAAATTAACAGTGAGTTAGCAATTTGAAGTGAGCAGCCATTACTCGCGCTTCAGAACACTACTCTAATGATCTAACATGTTTATATGTAAATAAGAAGACTTCAGTTTCACATGATATTCACAATATGAAATTCAAATTGGTATGAATTGATTTCCTTTCACAACACACACGCACACCTCATACTTATTCGTAACCATATCAATCTAATAACAACCTGACTTTCTTCATTTTAAGCATTCTAAATGAACCGTCTAC

>chr16:+:69266562-69270042

TACTAAATAGTTTATTCAAGAACGTCTATAATGAAACCGTATAAAGCGGGCATTTGCTCACAGGCTTCCTTTTGCATATCGAACATAAAAAACCTAGAAATTACAGACAACTGGCTTTCCTGTTTCTGGCACCTTGTTTTATATGAAAATACAACTCTACCTGCCAACATGGAATCTCTAATTCTTGACACATCTGATGAGATCATACCAAACTATACCAGCTCTTCTGGAGTCAATAGGAGGAAAAATAAAATGAACTGGAAAATCAGAAACTCAAAAGTTTTCAAAACTTAAGACTATGTAGAAGTAAGACTAATTGGGTTTTAAGACTGTTTCAATCTACATTAAGGAAATCACTTTTCTGAGCTACCAAGAGTTTATTTTTAAATACAGCTAGGAAAATGGCAACATCAGATCGTTCATTCACTTTTCACTGTATGTTCCACTGTATGTGTAAATTCATTTCAAACTATGCTTTTCACTTTGTCAAGTCCATAGTTTTTCTGTCATGCCAATCATAATTCATAAACAAGGTTGAACTGTGATACAAGCCTGTTTTGTAAGTATGATTTGGTCTTTTTCCACATTCACTAGAAGATTATAAAACAGGAGTCTACTTTCTCTTATTCTTTAGAAGGGTACAGGTTTACCCAAAATAATGGGGTCATGTAGAAAATGCAGGCCACTCCACCAGTAATATTAAAGAAGTGTAACTAACACTAATGAAGTGAAATTCAAATTACCTCACCAGTACCCCTAATGAAGTTGTTAGCTAAACTCCAAAGCTCTTGATCACGTACTATCTGGTCTAAACCAAATTATTTTGTCCAACCTTAAATGTATCCATTCACCTTCGAATGAATATGAAAACAAAACTTGGCTACCTTTGACTGCAAATAAATTATAATCAATGGCAGTAACCTTTTTGGCATTTTGGAATGGATATCCAATTATTAAATGAACTTTTAGACACACAGGAATATCTAGAAGTGTACAATCCTAGATTAACAGAAGTCTAAATCCATACTCTGGTTTTTATGATAATAGACAGCCATTAAAGAGACACGAGTATTTGTGTATTACAGTATATTAACATCTTGGCACCTAAATATCAGATAAACAGAAAAAGAACAAAATGACACAACACATTCACTCATTCTCTCTTTCTTAAATTAAGCAAGTTTATCTGAAAGACATCAATAGCACAATTAAATATCAAATCATACTTGTTAGGATCATTTGAGCAAATTAAGGCACAATATTGTAGCGATCTTACTCGTCATTGTGTCCAAGTGTTCTCTCGTTTCTACTTTCCTCATGTGGTTTTCCAGTAGGTCTCAATAGTTGTGGTATATTAGGAGATGCAAAAGGGGATAAGCAGGAATACATTTGGATACAAAATTGAAGTATAGCTATATGCAGAGAGGCACTGTTTATGAAAGGAATACAAAGGATGTCAAAAGGAAAGTGAGAGTACAATGGGAAAATAGGAAGCAGGTACAAAAGCAATAATTTAGGAAGGCCAAGAGAATTAAGTATGTAAAGCAGGAAGTATATGTAAAGGAGGGATACAAGAACCCTAAAAGGTGAAAGCCGAGAGAAGACGACAAAGTTTAGAAAAGAGGAAGAATACAAGATGATGAAGGAACCTCAATAAGGATGGGATGGCCCATGTCTTTCAGCATTCTACGAAAGGACTAACTGCTACCGTTCAGCTGTACATTTCTTTTCTGTAAGGTGGCAAAAGAAACAGAGCTGGGCGGATAAAACTACACACAAACTCTACAAATAACAGAAAAAATTCTACTTTGTTCCCTCAACAGGGTTTACTTCACTTTGTTCATGGCACATAATTAAGCCTTTTTTCCCTTTTGTTTTGTTGATATTTTTGGTCTCCTTTGTGGAAACATGAATAAAAAAAAAAAGCCACCACACAGTAGAGAGCTCTGAATTTCCTCCCTAATGTACAGTACATAAGAAAACTTCCTATACAAAAGAGGTCAGGGATGTGGGGTCCTCATAACATACTTCACTCCCTTGGGTGTAAACAGAGGGGCAATTGTTTGCTCTCTTGCACTTGGAAATATTGTACTAACTGACCCTCATCCCTCAGGATACAATATTGACTATAAAAATATACAGAGCTCAAAACAGATTATCAAATACCAACATTGTGTACTCACATTACATCAGAGTAAGAAAATATAACACTTTTTAACTTACAAAAAAACAGTACTACTGTTCAGTAAATAGTCAGAAAGCACCAGGATTTCTCCAATCTATGTACTATAGACTTTCTACCCAATATTAAGGTAAGGTTGGTGTCAAACGATAATGATTTCTGGTATCTTTTACCATCTTGCTGCCCAAAGTAGCATACATTCAATTTCTCCCTTATAGTAGCACTAAACTTCAAGTTACTAGTATGCAAAGTTTGATGGCCATGATGTTCATAGGTGCCCTGAGGACAACTTATTTATTCCTGGTAGGTATACTTGATAGGTTTAACATTGTTAATAATATTGAGAGAAATGCAGGTTCTCTTGGAATATTCCATTGCAGCCTTAATTCTGAGAACTAATACAAAAATATATTTTACAGCATTTCTTAGAAAGAGGGATTGTAGTAATGGTAACAACTGTAGGAAATAAACGTAGCAAGAAATGAGTATAACAAAAACAAGCTAGTATTTTCAGTAACTAATGCATGATAAAAAATGGCCTTTATAAAAAAGATACTGTCAACTGAGTGATTGTCTTGCTACTTATATAAACAGAATATAAATTAATACATCAGTAATCTATACAAATTAACATTTAAATGCAGTTAAGAGGCACATCTGCCCACTCCCAAACGAACTAATGGAATTCACCAGTTTCCTTTACCTCTGCTAGGTAACGTGAAGTGAAAAATTCTTGTCAAATAAGTTCATAACAACAACAATACACTTATTCGTATGACAAACAAATACTTTATGAAGGAGAATTGCAAGCAATGGACTGAACATGGCCATGAGATGAAAAGCTTCCAGATTTCTGTTCGTAAATCCAACAGATTCTAACTTTAAAACAACTGTCCAATCATATGATTTATAGAGACTAAGGATACGAAAAATTGCACGACTAATGGATCATTGCAAATGACCTCGGTCACGCCAAAACCACTGTGATTGAGATGGCAATGCTTAGATTATCTTTGTAACTAATTCTTGGGAAGCATAACTAAACTGGAATCCTTTTCAAAATGTCACTGGACTCAAACTGTAATACTCAAAAAGCTAAAGCTTGGGACCTCGATGTTGGAACTACTGGGGGCCCAAGCCAGACTTAACTTAAAACCATTGGTCCCTATGCTAGAATTCTCAACATATTGCCATTTGGATACTAAAAATCCCATAGAGACGCCATGCTAGAATTTTCATGTTGAACCCCAAACTACAATTCTCATCATAAACCCATTGGAATTCATGCTGGAATCATAC

>chr15:+:3053266-3055710

GAAAGATCTTGCTTAGAATCAGATTTTCTTACTTTATCTTTTTTATAAGAACTTCTGTCACTTTCTGAATAAGATTTATCAATCTTCACATGACCTGTAGGTTTTTTAGAATTACTTATATCATGCTCTTGATATGGTTTTTCGTATGATGACGACCTATGAGACTTTTCCTTACTTCGAGGAACAGAACTTCTAGAACTTCTACTCTCCAAAGAACTACTTCTTGAACTATTTTTGGAAGTACCACTACTGCTCTTTCCACTAGAAGGTTTCTCAGTACTGCTGTGATGGTCTTTACTCTTACTGAGATGTTTTTTACTACTTTTATCACTTGAAGAAGCTTTTCCACCACAAGAATTTTTAGATGCTTTCTGTTTATTTTCATTAAGCAAATTGTTTTTTTCTGAAAGAGCCTTAGGATCAGACATAGGAAATGTACTTTCACCAATTGAAGAATGACTACTTGTTTTATCTACAATTTTACTATTCCCTTTTTCCTTGCAAGAGCCTTCTTTGCTATTTTCAATATCAATTTTTCCATTATCATCCAAGCTTCTACAAGTTTCAGATTCTACATAAGAACCATCTTTATTTTTGCATGATGTTATGGCTTTATCCTTTATACTTCCATCATTACTGGCCCTAGTTGTTTCATTCACCACATCAGAATTCAAAGTTTTTGAACATGACTCTCTAGCTTCTTTAAACACTGCAGCAGTACCGTCAATATTGGAAGACCATTCTGTAACAGCTAAGTCTTCACTGACACTGTGCTCCTCATTTACAACTGATTTCTTTATTGCTAGATCTGCACAAGACCCAGACTGACGGACCTTTTCTGTACCTAAAAATGCATCCTTTGTCTCATCTAAATTACACTTTTGAGAGTCCCTATGACTACACTCACTGAAAGTATGATCCACAGAAGTTGAGGTTTCTGGTTTTTCACAATCAATTTCCAGTGCTGCATGCATTTTGGAAACAGACCTATTTGCACTATCTGAAGCAGCAGGAGGCTGTGGCTCTACTGACACTGTTAAGCCACTGCCTTGGGCAGTCACCTCTTGTCCATCACTGAAAATGCTAACTTTAGTGCTCTTAATATTTTCAATGTTGACCAGATCTCTTGAAATGGTTATCTGAAATGGTCCACTATTAACTTGTGCCAATGACTTATCCTCAAGAGAGCTCTTGTCCTTTCCACCTGTTTGAGGTAAACTTCCTAATGCCAAGTCCACCTCCACCTTCTTCTGAGCTATCAATTTGCCATCCTGCTCTTCTGAGTTGGCTTCTTCACAATCATATGTCCCCTTTTTCAGCTCATCATTACTTACTTTACTGTGACTTTTCTCATTCAGAATATTAACACTAGACTTGTCTGCTTTATCTACAGAGCTTTCAGTTGGGTGCTTGGTTCTGGCAATTTTGGAATCTCTCTTCCAGACTTACTATACTCCTCCTACAAGATGTTGGTTTAATTTCTTCAGGATCTTTATAGGTTACAGAGGACTCATTATTTGATATTTCTGACAATTCGACTTTATCAAATTGCTCATAAGGCACTTTCTTTGTTTGCGATGAGGATTCCCCTTCAGTGTGTTTTGAAGCATCAGGTTTAGACAGTACACTGTCACTCTTGTGCAGCAAGGGGCTTTCTCTGTCATATGTCTGTTCATGTGGTTTAGTCTTGTCTATACATTTGTCTTTATCAATTTTGGAAGTATCCATATCTGCTCCATCTGGTTTCTTCATTGATGTGCTGGAATTGCATTTAGTTTGTTCACTGGAAACACATTCAGAAGTTGCTGCTTCTTTGAGAAGTTCCCTCTTGATCTTTGCCAAACTTTGATTTGATTTATTATCAGGTTTTTTCTCCTTTTTCTCTTCATTAATTTGTTCATCTAAGTAAGATTTGATAGAATCTTGCTTCTCATCAGTTTTTGAATAATCAGTGACTGAGGTGTCTGACAGCCTCTTGGACAAGTTATGCATGCTTCCTTCGACTGCTTCTATAGCATTAATTTTCTCTATGCTTGAACTGTGAGATGGAGTTGGAGGTAAACTTATATTCAACTCAGATGAAACCTCTTTTCTTGAATCCACAAGTTCATTTGCACTTTTATCTTTCCCATTTCTGCTTTTTGTTCCTTCTTCTTGGAGCTGATTTCTAGTCTCCAACAAACATTCTTCCCCGGTCTTAAGGGAGTCTTTTGCCATTTCATGGTCCTTATCATTTTCTTGGTCCTTCCCTTTTGCCTCTGATTCCAAAGGACTATTACACAGGCCAACAGAACTCTTATTTTTTGGAGAATTTCCACTACTCCTTAGAATGTCATCTATTCTAACAGGTGATGGTAACACTTCTTTTACTAATTTATCTTTGTGATGGTCTATCTTCAATCGTTTAGAGGAATCTTTACTGATGTTATTAGAGGATTTTTTCCC

>chr15:+:16314589-16317904

CTGGTTTACATATTTATTAAATGATACGTATTTACTATAACAAATGCTCCCCTATGAGAAAACAGGAAAAATCACAATGAATTTAATTCCCATTTACCAATTCATCATTTCTCATGTGCACATTATTGTAATGCAGTCAATTATATAAACAACACCAAAGATCATCTGCATAGCTGAATTTACTTGATGAAATACTCATTTAATCCTGGAGCCCAGTATAAAATAATACTTAATGATTCAATCCCCACACTATCAACTTCCATGTACAAAAGAAAAATAGATCTATTGAAGTGCACAGAAAATGTAGACACCCTTTGACATGTGGAATGCTCAATACCAGTATAGTCACCTAAATTTTTGCATTTCCCTTCTGGTTCATCAGATCACTGTCTGAAATAAGTTTATAATCAAACTATCTAAACTACAAAAGATGCTAAGAGCTATTCACAAAAAAATTCCAGAGGGGAGCACAGAGTAAAATTAATGAAGTGTAGGGGAAGAAGCAGTCCTGGAATAGCATTATGAGGATTGTGAAGGCAATGCAGGGTTACCCTAACCATCCATTTGTGGGGACCTTATTCTTTATTATGCACAACAAAAAGGTGATCTGGCTATGCTTGGAATAAACAGTCCATTTCTGTTCAATGTTCTGCAGTATGAACTTGTCCTTAATACAAAAAATTTGCATTCATAACATGGAGAACTTAAGATGGCAGACTGTCAAGCAACCAAATTTTTTCTGCACAGCCATCAAAACACTCATGTTTGAATTTTCACTGTATAAAAAGAAAAAATATGACTGCTTCGCTACTCCCTGAAGGAGTTCCCTTCCACTCTTTGGGGATGAACCAACACAGAGAAGTGACACGGAAAAATTGGGTGGGCCTTCTCTGCATATTACAAACCACTAAGACACAAATCACAATAGAAAAGACATGAAGATCTAAAGCTAAATACTAGATATGCCACCATATTGTGCTGTGGATATTTCACTCCTACGCTAAACCATAGGATTTTATACTCACTTTGAAAAGGCATCTCCAGTCAAAATAGCAGGGTATACTCTACATTTGTAAACGGTCATTTACAACTGCATTTAACCCTGGACCTACAAAACTTTCAAGGATTATTATACAGTTCACAGGAGGAGACAAGATTCATGTTCATAACCATCTGTTGCCACATGCTCTTTGAATGAACATCGAATTGGGGGAATTTCTCTTAATTTCTGGTAATGCACATGGAACTCTTGGTTCTTCCACTGCAATCTGAAATGTTATATCCTTGAAGTACTATCTTTAAATGCCACCTCCTAGTTAACAAACTACCTTTCTCTTATTCACCATAAACCAGTGTCAGTTAAATCAATATAGGTGGCATGTGAATAAGATATATAAAGTTGCAACGACAACAATCTAGCACTGCAACAAATCATTCTTCAAAAACTTTAGGTGTACCAAGGCATGCAAATTTGTGGAGCATTTCTTATTAGCCTTTATACCACTTTACTATGACCTGTTAATTTGACTTGAATTAGCTGTGGACTTATCTGCTAAAGGAGGAAAGAAAATACAAAAACACAATTTATTTGGTGCCTACCCAAAATGCCATGCAAAAAGTAGTACTATGAAAATGTCCTACGAAAACATCAACACTTTCATTAGTTTTGATATGACCTAACACATGAATCTGTTATTTGCATGATTAAACCAAAAGAGAATATCACAGCTTACAACCATGTGTCACGGTGAGGAAACACCTGCTTGTACAAAATCTACACCAATCTTAACCATTACATAAACATTTGAAAGTCCATGAGTTTATCTGCATTCAGTAGCCCTAATACTGATAGTTTTAAGATAACAATGTAAAATTTAAGATTCAAGAACAGATGATCTGAAAATATACCAATATTTTATTTGGCCCAAAAGATTATTGAGTTGAGCACAGCATGATTGAGCATAACTGCCTCATTAATGGGCACACACAGAAATCTTCAAAAACATTTTCTGAATAATGATAATAATACATACCAACAATTTAAGGCAGTTGCCTTAGCAAATCTATGCAAGCCAATATGAGAAACTTCAACTCATTTCTGTAGTTTTAAATTCTATCAGCCATAACGTTTTGCTCAACAATAACTATGTTCCACTTAATTTTTTAAAAGAAACAAAGGACAATTTTGAGTAACATTACTGATAATATCAATATTTTCTAAAAAATACATTACCTAGGTATTGATGAATATACAAGCTCCAATTTGTACATGCAAGTAGTAAAAATGTTCTACCTAAATTTTGGATTTACTTTTCATAACACTCCTAGCTATTTGAGTATTATTATTTTATTATTATTATAAAGGATTAGTTTACCATGACCTCTGAGCCTAATAAAGGCTATAGCTATTTGAGTAACCCTACTATCAAGAGATGTTGGCCTCAGGCCTAGATTTGCTAAGGGTACAACCTTATAGTATTACCCTAACAAATGAAATGTGCAATATTGTCTTTATTAAATGATACAGGTAATTTCAACAACTGACATTTGGGTAATGTATTACCACCAAGTTATTAAGTGACAGAAACCATGTCTGAGCCTGAAATGATCTTTTATAGTAAATTTTTGATAAAGGAAGGCTTCCACTTTTCAAAACTTACATACCTGTATTGAACTATTTGGCATACTCTCAAACAATGCACATGACTAAGTACATCATAAATGAGGCATTGAAACTTTATCAAATTCAACTTTCTATGAAAAAGAATGAGCTCAAAGAACTAGTACACTAAGCTGACTGTAACCAGTTTTAATCAAAAACTTACATCTAATATTTTGTAACAAACACATGATTCAGATTCAAATAATTTCCACAACAGCCCATACATACTATATCAAATTGGATCAGTTTTTGTTTTAACAAGGTTAAACTATTCAGTATTGTATCAATATTTTCTTTTATTTTTAACAATTTAAAGAATGCAAAATAATGTAAATGTCTGAGCAAAGCACTGTCAAGTCCTACGAAGTAATATTAATTGTTTGCCAAAGTAAATCCAGTATATGTACTTTTGTTTGGAATTTATTGTAAGGCCTCACAACAAATTTTGATACTTTTAATATCAAATTTTTAACTGCTTTGCACTTCCCTAACCCCTCTCTACTTCTTCATATATAAAAATGTTCAAATTCTGCTCCTAGTTATCAAAATTCAAAACGAAGAAATGCTTTAGCATCTAATCCATCTTGCCAAAATGCTCAGTGGGAAATAACACAAGGGGATCTTCAGTTCCAAGGAGTTCAGTTTTGTAGTAAG

>chr15:+:29354946-29358415

GGGTAAGATTTGATGTGTGCAAGGATGTTGAAATCCTTTGATGGTTTTACATGAACTAGGGTCATAACTCTTACCATGTATGTGCAATAAAATAATTTGTAGCTGAATGTAATAATGATCCTTGATAAAAATAAATTTACTTTAAAATTACGTATGTTATAACTTCTCAAGAGCTCAGAATGAGATTACAGTACATACATGTATCGAGGAAAGCAATGGGTACTTCTCATTTTATAATGGAAATTTATCTTTCGCAAGACTTCATAATTTTGGATGAAATATTCACATTATTATGATGATATTGATACATCATGATACCTCATGCAATTAAAAGTAGCCAAGAGCTCAAGGGAAAAATAAAGGCAACCTGAGAAAATGTAGACTGCTTCGTGTTATTTTTTTCATCAGTGGTTCTAAGCAATACCATAAATGAATATTGATTTCTTAAGCAATACTATGTCTTAATAAAAACTGACTAACTTTTCTGACAAGTCTTTGGGCAGCCTTACGAGAGTCAAGTATTTTGATTTGCTGGTTTGGTTACACTAACCAATGATGCTGGTAATTCCAACTGATCTAAAACCAAACTACATAACAAAGACTATTTGGTCAAAGGGTAAGCTGACCTCTCCTAAAAGAAAAGACACAGTTGTGGCTGCATTATGCAAACTGAAGGTGATTCCATATCTGTTCTTGCATAAGTTACATCCTTCCTTAGTCTTTAGGGATCCTGATTTGTGTCCCCCTTTGGGTGGAACCACCTCCTTTAGCAGGAAGACAATTTCCTATTATTAATTATCATTAACAATAATAAACTGTCTTGCTACTAAAGTTAGTGCTTCCACCTAAAGGGGAACTGGATAGTCTACAGCAACTAGCAGTTTCCTCAGATCTTGGTTGGCTATGTACATCAAGAGGGCCCAGGGTACTGGCCTATGGTATGACAGAGGCACAATAACAACCATGTAATCTAGCCCATCCAACCAAGTTGTGCAAACACTATAAACAGCAGTGACTGATGAAGTCATCATTCAACCATTTATGATACAACACAATTTCTCTAGTTTAGTCTGTTTAGTGTGTCATTTATTAAGTCATGAGCAAATTATATGAAATATCGAAATCAAATAACATCATTACAGTATGTAATACAAACTCTCAGTTGAGGTTACGGGATTAATGGGGACCAACATGAACTACTGTATGTTATCTAACACAGAACTTGAGACACAAATTATGATTTTACTGATGCTTAAAACACAAAACGGAAATAATTTTGGAGTCAATGTTATTTATGAATATTAAATTAAGGGATTTCAGCAATTTATTCAACAGTCATTGGGCTCATTTATAGTCATACATACTGAACACAGCTTTGACGATGACACAGACTTGAGTTTAACATGAGATGAAAGATATACTGTATGGTAAAAAAATGGGAAATGAAAATACTTGCTGCAAATACATACCAACTTAATATGACACTTTCTTGATATACAAGTAAATTTAATAACAGGATTTCAAATAATGCCATATTACCTACCCATTACATACCTACAAACAATTACAACAGCATGAGTGAGAATTAGACATGCCACAGAGAATTAAAATTCTGAAGTTAGTTGCTGGAGTTACCTCAGTATTTTGCCCACTTTCATCATACCCTTAATATCATTTACCTTCATCTCGGAAGCACTCATATCCTATGTTCTAAAGGACTACTATGATCCTTCAATTATCAACCTATTACTCTTGTAGGCACAATATTTGAAACTCGAAAAGATTATCTAATATAAAACTTGGAATTTTAAAGGGCTATTCTCATCAATATTTTTACAAGTTATACAGCAAGACTCATACAGCGTCACGTCACATAATTACGGTTAAGAGTAGTTTAAAGAGACATGAAGTAACATAGCTAGACTAGACTCTCACAGGGGACTGATAAATAAAGACAATCTCATAGGTCAGTCACAGTTATGACTCTCATACAGCTCATCAGGAGACTATTCATAAATGAGAAACGAAAATTGTACACAGTAGGGTCGCAGATGTCAGTCAAGCAAAGAGGCCAATGAGAGTGAAGAAAAGTAAAAGGCATAGGGCAATGTAACTGAGGCTCAGGGTATCAATAAGGCAAAAACCTTCAGTACCATCTGTACTGTTCTGCACAAGGCACACTTTAAGCCTTCTCTATCAAAATCAGCATTGTTTCCATAAGGCAAGATTAAACGGTGATCTGTTCTCCCTTAAATCCCTTATAGGCATCCTCTACCACATACTGAGGAATCTTTTATCATTCATTCATCTTGACGTTTGTAAGGCAAATTACAGTCTGGCATAAGGTCACATTAACCCTACTCCATATTTACTTGTCAAACCGGATTTGTTGTGTACCTTACCCTTTGTCTTGTTTTCACTGACTTCAAGTACCCTCTCATTCTACCCATTTACATGTAAATGATTCTCCTTGACAAATAATCTTTCCTTTTAGCTTATATTATCATGACACACAAGGCCTTTATTATCCTAAGCAAATGACATATAACCACTGCTAAAGAAAATGGAGATTCTGAACAGGCCTCATAACAGATTGCTAAAATACATGGCTCTTACTATTATCTAGTAATTTACATTTGGAAACCAAATTCTGAAACTGCATTCGAAAGTAAGTCTAGTATGATGAATCATGTTATGAAAATGAATCAATCTAAAGCTTTTGTTGATCTGAGAATAAAGCTTTACTGAAAATATCAGGAGTCAGATGGCAGAATAGTTATAAATGACACATCAGGCAAATTACAGAAGCTTCATGTAAGCATGAGAGACTAATGAATGGGGAGATGGAAATTACTTGGACATGTCTTTCTCAGAACCCTTAGTAAAATAGTAGTATATGATAGTGTTCGCTGGACTCCTGCGGGTACCAGATTAGTTGGAAGACCCAGACCTACTTTAAAGAGAAGAGAGGCAGGAAATGAGATTTGTGGAAGATAGAGCACATGAAAGACATGAGTGGGAGAGTTTCACAGAAGCCCTTTGCATTATGTAGTGATGGAGGTGATGATGATGAGCTTAACCATGCCACAGAGTTAAATAAGATTCATGGTCAAAATGCAGTGGAAAAATTATACTAAAAATGACGATGCTGCAGAGGAATAGGGTGTTCTGCAGATAGAAACCATACCTAGATCTCAGTGGCAGATGGTTTTGAATCTAAATTACGGTTTTCTTAAGAATCAGATAAATATTCTTCAGTCTGCATTAGATGATTCTCATAGTCATCATCAGTCTCTACATTGAGTGGTAATAATATCTCTTCAGGCAAAAGGTTTTTCTTTCACTTTGGAACATTTAATTATGCAGATAAATTGAATCTAATAAAAAAGAAATCAACCATTTACTAATGAACATCTGGAGTGTACCGAGAAGTGAGAGGTAGTTCACCTGTCAGACTGTTTTCATGTTAC

>chr15:+:33115057-33116819

GCTCCTCAGTGCCTGTCTCGACGTGTGCGCCCCCCCAAAACACCCCCCCCCCCCACGGACTCTCCGTTGCCCTTTTAAGATTTTCGTATTTTTAGAACTTAAAGTGCTTTAGGGGTGTTGTAACACTTGTATTGGTTACGAAGCCGTACTTCAATTTTTTATCCGCTACAAATATATAAGTTTTTGTTTCATGACAAATTTGTATACATGATTTTTTTATATAGTTCTTTAAGAATTGAAATGTTTTGGTGGTATGACAACATTATATTTGGTTACGAAACCGTACTTCAATTTTTTCTCCGCTACAAATATGTTTTTTTTTAATATATAGCAAATTTGTATACATGATTTTTTTTATATAGTTCTTTAAGAATTGAAATGTTTTGGTGGTATGACAGCACTGCATTGTTTATGAAATCGGGATACAACTTTTTTTTTTTTATAGCAAATATATGAGTTTTTGTTTTGTACCAAATTAGAATACTTATTATTAAAGAAACGGTAGAAGGCTGTCACTATTTGCTTTTATATTTCATCGTAAAATTGCGAAAATGAGAAAGATCGTTCTGATATCTTCTTTGTATATTATCTTTGATTTATACATTAGGTTAACTTTGTTATGTTAGTTTTCATTTTTTAAATATATTATTTTAGGCAGTTGAAAAAACAGAGGACACTGCTACCTGTTTTACGAAAGGTTCTTATCTCCCTCACCCTCTTTTTTTTATCTTTCTTATTGTTATATCTTATATTCTTACACCTGTAAGAATTAAAATGCTTTTGGAGTGTCACGGCATCTCTGCATTAATCATGAATTTTCGATATTAAAATTTTATTTTTAATAAAAAAAAATTACTTTTCATCAGTTTTTCAAGTCATGATCTGGATCTATTGTTGTTTTGTAAAACGTGATTCTTAACTATTTCCAACACTTAGAGTAAATTTGGCTGATCTTCGTTTTGATATTTACGTTTGCCCTATTTCATGATCTTTATTTTTTTAAATTCAATTTTTAAGCAATGGGAAATATAGAGAATGATGTCCGTTTTGTTAGAAGATTAAGATCTTACTCTGTTCTTGATGTTGATAGCGTAGAGACGCCCTCAGTTTTTTTATCACACATCATCTTATATTCTTGCTACTTTGCTAAACTTATATATTCTATTCTTGCCACTTTGCTAACATTAGCGTTTTCGAGCCCTTCCTCTCCATCTTAAAAGTCATTTTCATCCACCATTTTTTTCGGCAATTTTGGCTCGATATCTCATTGTTCTTTAATGCCGCACCTTCAGTTTTTTAATTGTTTTTAAGCAATGGGAAATACAGAGAATGGTGTCCGTTTTGTTAGAGATTTAATATTTTACTTCGTTCTTGATCCTGATAGCGTAGAGACGCCCTCAGTTTTTATCACACATTATCTTATGTTCTATTCTTGCTACTTTGCTAAACTTATATTCTGTTCTTGCTACTTTGCTAACATTAGCGTTTTGAAGCCCTGCCTCTCCATCTTAAAAGTAATTTTCATCCACCAAGACTTTTTTTCGACAATTTTCGCTCGATATCTCGTTGCTCTTTGAGTTGCACCTTCAGATTTTTATCATTTATATTATTTGTAATGACCAGGCTTCATTTTTCTGTTGTAGGTACAATATTAATCTCATGCAAAAGCTATTTATTTTTAATTTTGTAACTTTCTGAACATTTTGGGTTTACAGCTTTGTAAAAATATTGACTGATGTAATAAAAACGTATTCTCACTTCTC

>chr15:+:64958879-64962073

AACAATGTAATATTTTACTGTCCAATAACTTTGACTTGGGTAGAATTTAGAAACTGCACAATATAATGTACACATTGTAATAATAACTTGGACATAAAACACACACTGACTTTTGTTTTACTACCTATTTAAAATGTTAATATAGTCTTTTATAGTTTGTGACCGCGAGTTACTATCGTGACTCCAGCGCCATCATTTCCACTCAAGCATTTTGAATTTAAAAAAGCTTACATTTACTATAATGCCCATTTGCTAATACCATATTGGCACAACATAAATATTCAAAATACTTGATCACGTCAAACTGTCACTCCTTACATCTAGCTTAAGTAGGGGATGAAATTTCTGAAAATGTCTGAATAAATTGCTTTTCCTTTATATATGATATGCAACACTACAGCATTGCTTCAAAGTAACTAACCACTAATACCATAGATGTAATCTCACCTGCTAGGATGACCAGCAACGACTGAAAAGTATAAGAGAAGCAATACAAATGTGACAAAACACTATCCCCATGCTATCTAACGAGTTTAAAATATCCATCTGCATGAAATACAAAATCCTTTATCAAGGATTATGATCTTACAAAGCTTATAAACACAGGATTTCACATTATTCATAACTACACATACTCCAAATATTTTCAAAACTAACACACCATACCTTTGACCTGAAGTCTAGATGGTCTCTTGGGTTAGTACAATGGATAGGAGATGGTCTCAGAGAAGTATGATGATTAAAATTACCATTTCAATACACTATTTTGGTGATACGGTGTACTACACTGACAATATATGTACCAGATACCATGAAATACCTGGAGATACAAATCCATAAGCATTGTACAACATTACACCTAGTACGTATTGGTAATAACAGGTGTAGATTTTTTTATATGAGCGAGATCCCATTTCAGACAATGGAACATACAACTCTTACTCATTGCACACAGAAAAAGCAGTATAGTATTTAACATTGTCTATACTAACTCAACCTCTTTACATGAATACAGATATACTCATCTTAAACATGTTATATTTGTGTTAAAGATATGTAACTTTCGTACAAAACTCTGTCTAAATGTGTTATATTTGTGTTAAAGATATGTAACTTTTGTACAAAACTTTTCCTTTTATAAATTTCCAAAAACTATATCGGCAGCATCTTCAGCTCAATGAATCTGGCAGTATATTGTCATCTTATGGTTGATGTAGTAGTAGTAATGTGTCCTTTTCAGATTACAATGAATCTGGCAGTTTATTGTCATCTTACGGTTGTAGTAGTAGTAATGTGTCCTTTTCATATTAATACTAAAAGACAACACTACTGAGATAATTAAATTCTTTCCTCATACTACCAACCTACTTTGCAATGTTCTGTTACTTCCAGTACATTTAACTTTTAACTGTATGCAAATCATTATTCAATGAAACCTCAAACAATATTTAATAAAATTCACCTACGAGGTATTTTTCATATGACAGCTCTTTGGTAGAACCCAGAGAAAATTTTCCCAAGGCTTGCATATGCAAAAATATATACTGTACAGCCATTAATAAGCTAAACAAAATGAAAATCCAAGTAATCAAGATCAAAACAAACTACAGTCAACCATTTTTTTTAGATTTACATCTCTTCACCTTCTTATGTATGCTTCAAAATTGTAGAAAGTGAAACTTACGAGCTTATAGTATATAATCCTGATTCTTGTGAAAGCAGTGTGCTTTGAAATAAAAAACACATCCACCAATTATTCTTCAGTACTTATGTAAAGCAAAACAAAATCAGTTAATGACAAGGGATATCAATGAATACAACTTTACTGAAAGTAACATGTTGAATAAGATATTGCATATAACCAGTGAGAAATCAAATATAATTTCAATCAAAAACCAAACTAACTTAGGCTTCAGTTGGCCTGCTCAATAGCTATTTAAATTATTTTTTTCATGTTGTACATGCAAATTACTGACTTCTACTGTTCACTGCTGTGTAAAAACTGAAGAAAGAAAGAAGCTGACAAACGAGCAATATGGCCAGCCATATGAGTGACCACACAAACATTACACTGATTTTTCAAGTTGACATTTTTGATTTGTCACTTACCCAGGTCAAATAATAGTTAATGATTCAAAACAAAACAGCAGCTCCTACAAGAGATTACACATTTTTAATACAATTTTATCATTGTTCTTGAAACATTTCTTCAAGGTTCCATCAGCAAAGGTTTCATTTGTAATTCAAGTTATTTTAAGAATTAAAAATAACCCAAGACCATCCTTCCACACTTCTGAATTATTTTATATTCTCAATGTACGTAGACGCTTCTATGGAATCTTCATGTTTTTCAGAATTGGTGTAGTTAGCAAGTGTTCTGATATCAAATAATGATAGTAACTTCAGTATCTAATGAAAATTAACCCTAAGCAGAATATATGAAGTAAGCTTGCCCTGTATATGAGAAGCTCTCCTACACTGTAAATATATTGACAACTGATAAAGGATTTGTGTCTACTATGTCTTATAAAACTTTTAAAAACATCCCCTGTTATACAGTAAATACAATGAAGTGGGGTCTTGGAACATCTAAACACCTAGTTCCAATATTATGAAACAAACATTAATATGTGAATACAACAATACTTCACACAACACTAACAAAGCATTTACAGGAATTCAGACCAGTGGTATATGTACTAAATATGTGAAAAATATTATAAGCAAATTCTAACCTTTCAACCAAAGTAACAAAATAAGTTTTCTATTTTAGCAAAGGTTTAGGTCTACAAACTGGCACAAACTATCTAGGTAATATGAAAAACCTGATCTACATTTTGAATAAAACTACAGCATTTCTCAACTCTTACACGATATAAGGGTCATACCTCCCTTGGAAAATAGTTACTGCTTGTCTTAAAATCATGTTCTCAGAAAATAGAAAATGATAAAATTAAGTGGTATGATGTAGTAGACCCCATTCTGAATACTGACCGATAGAATTCTCAATAATCCTGATACAGTTCTGTATCAAGCTCCTTTAAATAACTGCTAAAATAAGTTACACCAATACAAAAAATACAGCACAGAGTATGCTCTAGTTTTTGACTGAGCAAAATAATGAAATTACCCTAGACCCAACCCATTTACCCTGTAGTAGTAAATTAGTAAGGTTAAATACAACCTATAAGGTCT

>chr15:+:74772848-74776202

GGCATGCCATGGCATTTTAAAATTCAAGAATAACTGTGCTTATGCTTGACAGTTCTCTCTTTTTGCCTAATTGAGTGAAAATGTCTCATGCAACTGTGTAGTGGTTTGTGACCCTTCAGGCACCTAAAAAAGCTATTACATAAATTTTAGCCCCAGTCTTGCCCAATTGTATTTGCCCAGAAGAAATTTATCCTGTTTTTCCCCTTGCTCTGTATGAGGGGACTCATATGTTTTTAATATTTTCCACTTATGGTTGCATAATTGTGACTACTATTGATTACTTTTTTATCATTCTCATCATTACTAGTAATTGGTATTAAGGATGAAGTTTGTCACCCTTTTGGAGCTCTTGTTTGTCCTAGACTCTGTGCCTTAAAGTTTAAAAAAGTATTCCCCCTCATAAGTCAGAATTTGTTTGCTCTTATATGTGTGAACACATTGCATGCCTGTGTTTATTGCGCCATTAATACAAAATGTTATGGCTGGTAAAGAGAGGTTGCTCCAATAATCTTTTGGTGTTAATTGCACAATCTCTCTTGTTTTCTTGGAGGCTCTCCTCCAAATTGCACCTTGTGAGTCATCCCAAGCAACTAATCTCCACCTCTGTATCTGAGAGAGCAACCTGGAGTCAGAATTTTAGAAGTGTTCAAGATGGTTAGGAATGAGCATTGTGTGCAAGATTACATAGTATGGTGAGTGAGGTGGATAAGTTTGCTGCCAAGAAAATTAAACTTGATCCATTTGTATTAATATTGTTGCGAAGTGATTCTCATTTATGTGATAGGCCCAGTCAAATGACTATGCATTATGATAGGAAAATTTGCTAGGCTATATAACAGGAGTCATTTGGCAGTTAGGATATTCTCTCCGTATATCAAAGGTTACCTTAGCTACTGCTAGGAAAATTGTTAGGCTCCATTTCTGATATCGGAGATTTTCTTTTCTTCCTTCCATAAATTACTATGGCTAGGTTGATGATAGGTTGTAGGAGAAAGGGAGGGATGGAGTGGCTAGAAGTATAGGAATGGATAGGCAAAAGTTAGGAGCCGGGTGGGTGCTCATTGCATTCTAGTGTTGTTATTTTTCTATCTGAGCAGTATTTCAGTGTTCTTTAGAAAGAGAAATAACAGCAATGTGTGCATTTAATTTTATTTATTATATTCACTCTGGGAGTGAGGGTGTTTTGAGTGAATGACAGCTTCTTAATGTTAATTCTTTGACAAGTTCGTGCATACGAATTTATTTTAATGCGGGTCCTCTCTCTCTTAGTTTCTGATCATGCTCAGTGATGATACTAGAGTTATTATATAGTTATGGTCTTAACTAGTCATTAATATGTTAGGGATACGATTCTTCGCTATTTTTATTATATTTTTCAGAGATGTTATTAGGCGTTAGGTTAATTATATCCTATTTTTGCTCATTTGTCAATGTTCTAGTACAAATATTGTTATTGATATATCAGTTTTAATTGTTTTTGTTATTTGGAAGTGATCAATGGGAAAACTGACTTTCTGATTCCAGGTGTGCTGACTCTCTTCAAAATTTTTTTTCAAAAACGTGTGTGGTTAACCACTGTACTCTTCAAGTAGTCTGCCCAAGGATGTTTTCTTGTACAAATTTTTTTTTCTTTTTTTTGCTGTGATAAGTGTTGAATCCTAATTTGTATACAATTGTGATAAGGTCGCTGCACAAAGTTAGGCTCGCAATGTAGGTACTCCTTATTATTAGTACTTATCTGCTATTACCCAAACCCACTGTCTGGGCAAGTGTGCGCTAAACTACAACATAACCATCATTAACCTAGAATAATTGCATGTCAAAGGCTGCTGTTGCTAGGTTTGTTATTCTGCCATGTACTTGGTATATTGTGGTTAAATTGTAGGATCCAGTTGTTATCAGTAACTGAATAAAAGATTGATTAGCTGTTGCCATACATTGTATATGCGTTGTAAATGATATTGTACATAGGGAGATATATTTTGTTTTGATATAGATTCCTCTCCAGATCTGTTACCTTTGTGTGTATGCTTTTTTAAGTCCTTTATCATAGTCCTTGCATTCATATATAGGGCCTAAGTACATTGTTACAAATTAGTCATAGAAAATTATAGTTTTCACTTCCCATTGTTTGTGATATTCCTTGAACTGAAACACAAACCAATCATGCATTATGAGGATAGAGATAGTCTTTTCACCAGGAAATTTTTCCCATTGAATGTAAATAAGTTTTTGTGGCAAATGGTGTGATAAATGACACAAATTGGGTGAAGAAGTATTGTACTAATGATATTAAGATTAAATGTAATTAAAAGTTACATAAGATTTAATACTCTATTTTTTACCTTTATTTAGGTTCAGGAATTTACGCTCTTAAGAAATTTTCTTACCTTAAAGTTAGATTGTCATTCCCTTACATATAGAGGTCATTGCTTTCATGGTTTGTGTGTAAACTGTAGCAGCTAAAAGGTTCCCTTGGCCTATGGTTAATCAGATTTGAAATTTAATCTGGTGAGGTGACTAGAATTTTAAGTTTCTAGGGTATTTTGTGGTAATTTTGTCCCTATATGAGAAAATATTTTGAGAGTATTGTACTACAATTCTTGAAAGCTTATCTATATTGTTATCTTTTATACTAAAGGTAGTTTACATAGTTTGTGAATAGCCTTGTACTTTGAGAATTCCATTTTACCCTTGGGCAGTACTGAAGTTTACATGGTATTTGTTTTTAAATAATAGCTATAATTTGTATGTAAAAATAAACACTGCTGTGTGCTAGGACCTTCTAAATATTGTCCTTTGTAAAGGTGCAAAGGTGGGGTTAATGCAAATAAAAAAAACTGCAGATTTAAAGTTAAGAAATATTAGATTTTGTAACTTCCAACCAAAAGCAGAAAGCAGCGTTTTGGCTGAACGTCAAAATGAAGCTTTAAAGCCATTATATTATCTGAAAGATAAATTTTCAAGTGACAGTCTGCAGACTAATTTTAAATTACTGTTAAGGTGCAAAACCAGTCTCGTTCATGTTAATTTTGCTATGGTACAGGTGTGGTGTGATGTATGTCCTTGCAAATAGAATCTTGGAAGATGAAGATTAATCTTGAAGTTTAAAAGTAAAATCTCAGGGATGGTACGTATTAAAAGATACAATGGACTAAGCATATTTTTTCCGAGACCTTTCTTCTGTCAATTTACTGTTGTGTATTCTGAAGGTATGAAAATTATCAAATTTTGATATTTGTTTTTGGGGGGAGCAAGTCATGATTAATCCTTGTCTGTCTGCTAGGTCATATACAATCATATCTGATTAGAACTTTTGTTAATTAGTGGTGCAAAGTATAAATGGTATGGTGAT

>chr14:+:38096780-38101039

GATTTTAAATGTTAGCAGTTAAGTTGTAGTGTTTTTATAATAGGAATCATCTACAGAGGTTTATAATCCTTGTAGACTTTTCTTGTAAACTTTAAAAGTAAAACTGCAAGTACTTTCCAGGTATATTAAACTGCAATTGTATTCTTACAGATTTGATTTTTTAAGTATATACTGTACAGTAAGTAAGATTTAAGATTCCTTATTTTGAAACACATGATCATATGCTGAAACTGTGTAAGTCATTCTTGGTTAACAGTGTCATTTAGGCATACTGTCTTATTTGCATTATGTATGTTAAGCTTTGCAGACCATAATATTTTCTATTTTTCTGTCCCAGATGTGACAATAAGTTTCTGTGTACTGTAAAAGTACTAAAACAGCTAAACCTATCGTTGTTAAAACAGGCATTGTTAGTATAAATATTAAATGTACCAGTATCTTATCTGTAAAGTGACAGGTTTTATATTGTGTGCAGTAATGTTCCATTGGGAAAAGTATGTGCTTATGGCTTTCTTAATTAATATGTAATTACTTGTCCTAAAAACATTGTGATCATTAGATTTTGACCAGGAGCTTGAGATATCGTTTTTCACAATTCTCAGTATTTAGAGGAAAAATTTGTGGTTGTTCTGTGTTGTAAATTGATAGCTCCCAAGCTTTCTTACTGCAAATAATAGATAGTGATGATAAATTTGATTTATGGTTTTATAGTGATGATAAATTTGATTTAAGGTTTTATAGTACCATTGCTTTGGTATGTCAATGCAGTTTTTATGTTCTTTGTTGTTATTAAAAAATGCTGCAGTCATATTTCTCCTCCATGCATACTTTACCACTTGATTTTATATGAATCTCTGAGGCTGTTTTATTTACAGTAATTTATCACTGCTGTATTGCTAGTCACGTTTGAAATAATGCTGATGAATTTAGGTATTAGTACATATAAGGTCATTGTAAATCATTATCAGTAAGATATCACTCATTTTCCAGTTTGTTTTGAATTAAAGTGTATTTTTAAAAGGTCATTGGCATTGGGACCAACAAGAGCTTCATTGTCATATGAGCCTACTCCTATTTATTTGGTATTGATCATTCATTTTTGCCATAGAATAATTGGAGATGAAACACATCACAGTTGTCTGCTATTGTCTTAGACATATTTTAGTTGATACAGAGGTATGCTAGATGGCAGTAGTATTAATTTTTGAATAGCTAGTAAAGATACTTACATTGTTAAAACAGTAGTAATTTTCAAATAACTATACTTACTAGAAGTGCATTACTTTCTTGTCCATTTAAGAGAGTTGCATCACCGTGCATGTGTAAAAGTTTGGTTTGTCATATATTGTCATTTTTTTAGCATATATTATCATTAATTCTGGTGCTGCCTATGGTGTGCCTTATGCAATTCACTGTAGACTGTACTTAAGGTTTGTAATTTTCATGTTTCCTTTGGCCTTTTCCCACACATCTGCCCACCTTTGACCTTGCCTTCATCTAATATCCACATCAATCTGTGACCATGGAATTGCCTGAATGGGAAGTGGAGATCACTTCAGATTTGGAGCTTATCTGTAGTATACCTGAACCTGAATATGAGAATGATGGAGAGAATCTGACTTCACATTGCAGAAACTATACTGTATCTCTGTTGTGGATGTTGGGAACCATGTTCATCTTGGCTGTACTTATTTTACCCTTTTCTCCAGAACTGCTACAAGAATGTAATTGGATGTAAACATTTGTTTCATAATTAGATAGAAAATGGGCCTAAACATAGTTTGGTCTGCAATTCGAGGAACAAGTTGAATTTTATAAATATAGATTCAGAGGTAACTCTACAGTGATTACTAAGTAAGACTGGTTAATATTCTCAGCTATAAGGAATGGTTATTACTGTAAGCAGCATAGTAAACATTGAGTGCGCTTTTGGTTGAAATTGTTCCTAGCACACCACATTGAGAACCCAAGATTTAGAGCCAAAATGATTTTTCTGCCTACCATTTGTTTGAAGATTAGAGTAGTTAAACTTAATAGATGCCATTGGTTTCTCTCAAACAAGAACAACTACAATTAGAATCAATGTAGACACTGAAAAAGATTGATAAAAAGCTTGTTAAGACATCATTACTTTGGAGGATATTTTACTGGAACATGTTTGTAAAAACAGGAGTACAGTATTAAGTATTCATCCACAACAGCCTTAGGGGTGTTAGTCCTATTAGTGACCTCTTGTGATGCATTGTTGGCATTACTCCTAGCTGCACCCAGTTTTTAGCTTTGACTACCGACCCCCAACCCTGCTAACCATTAACTCTTACTGCAACTGTAGAGTTTTCTCCTGGTTTCACTTTAGATCATTGTACTTTATCTCCTTTGTTTTCTTAATGTCTCTGTTCAACCACTAACTTTTTCCCTGTCTAAAGCGCTGAATGGGTGAAAGTGCTGTAGTGTTTCCAGATGTTGAATTCAAGAGCTATATGTAATTGGTTGTCTTAATAGTAATACAGTACCTTTAGTGCTGTCCAGATAAACCTGAAAGCTATCATATGCTTGATACACTTCAGTGGATTAGGAAAATTATGGAATCTTAAAAAAATGAGACATCTGGTAATACCAGGCTGACAAAAATTTAGAGATCTTGTGTGTAGAATTTCCATTAGTCAGTAAACCAGTGCAGTATCTGCATATCTCCCTGAGTAATTGGGTAGTAGATATAATTTACTGAAAACTTGCCTGACCATTAGTTACCTCAAATGGTATGCAGTTGTATTTCTTTGATTGATAGCACCTCATATATAAGATATAATGAAAATGATAGTACAGTGCAGTTTGTGGGAGAGGGTAAGCACCACCATCCTTAGAAATAGTTATTCCTTTCAAGGTGAATCACTCTTCAAGAAGTGCTGTACTGATTTTGACATAAATTTGCTATCTTTACCTAAAAATATACACATGAATGTTTCCATTTCAAATTGAAACTCAATATGTCAAAAGAGAAAGTTCAAATTAGATAGCCAAATGGATGTTTTCTTATCTAGAGATTATTTCACATTCTACACACCATGCTTTTGGGTTAAGCACCACAACAAGGAGGCTATACTATTATAACCATTAGCAAGATCTGTGTAATGTAATGTAATGTTAGCTTAAATTATGCCACAGTCTTGATGCTACATGTACTAATAGCTTTTTGGGCTGCTTAGATCAATCAACAGAAATCTGAGCAAGTGTTACTTACTGATCATATGTAATATTAGAGTAGAAAGGACAGCACTGATGGTGACAACCAGGGTTAGTGCAGCATCACTAACATTTCCAGGTGTAATAAACATTTCATAGTTATTCAGATGCAATATTTTTTATTTATCACAACTCAACCTAACATGAAAAAATGTTGATGCATATTACCTGTAAAGTTGCATTTCACAACTCTTTCTACCTTTGCTCATACTGAACTATTGTATTCTTATCTGTAGGGGATTAATATTTATAGTGTACCTGTGTGGCACAGTGCAGACATTAAAGGTTCATTGCAGTGCACCTTCATCCCAGATGCATTGCTTTCTTTCTTTCTCTTTTCAGCCATTTCCAAGGTTTTTTCCACCTAGTGGTCTTAACTTCTGTCATACTTTATTCTGTTTTCCACTCTGGTTTAAGAACATTTTTTGGGTGACCTCATTAAAAGTCTAAACAGTGTGATCATTGAACCATTTTACATTAGTATATCAACCCACTGACCATTAAAGGAGTGAACAAGCAGTGTAGGTTACTCTTACTACACCACAACTAATATGGGGTGAAGTTTGAATACTATTTGATATAGTATTGTAGTGTGCTCTACAGTTTCACCCAGTGTCCATTTCAGAATAGGCCTGTAAATTGTGATATTTTTATTTTGGTTTGCAAAGCTTTGTGTGGTTTATGTAAAAAGTTGGTGGTATTATTAGGTATATTAAACCTTTACTGTAATAATAATGTGTAAATGTGATTATAATACTGTACTGTATATTGTGTATATTCCAGCGGAACTGGTTGAAAGTAAATTATCGTTTTACACATGAAATTAGAATTTGGTAATTTATCTGTGAAAAATATTTGCTCTATGTGATGTGCTATACTCCATTTCATATTTAGCTTGTTGTACAAGATGTATGTTTTTATGTATTGTTGGTCTTACTTTTTGTCACCAATTTTACACATCTGTGATATTCTTTAGAACAATTTTTCATTAAGCTTCAAATACACTTTTGCTATATG

>chr14:+:56221399-56223317

GATTGGGTCAGATGGCAATACTGTATAATTCTAGTTTGGTAAACAATAATTTTTACATTTGGTCATTAGTGTTTAGTAAAATTGCTAAGGAAAGATGTATTTCATGATTTGCTGAGTTATCAAGTAACATTCTTTTAACTGCCTTTTGTAGAATTTACTTACATATAACGATCAAAAATTATATTTTAAACATGTTTGCCAGTGAATTTTCTGTAAATGTTTAATTTTACTCACATTGTTTCAGCGCATACTGTCTCATTACAAAACATTTCCTTCCTAAAAAAATTGCCAAACTTAAAACCGTGAAATTCTGTAGCACCTCCTCTTGGAGTGTCTTTCAGTGTAAGGCCTGTCAGTAGACTTTGATATTAGCATAATCTTTTAGTTTTCTCATGGTATTGCTAGGGATTACAAAATGTTCATGGTAGGAAATAATGTTTACTGACATAGTTTTAGTTTATAACAATAATAAAAAGATCAACCCCTTCTTTCCACTAAGTTCATTTTTTCTTGTCACATGGGTTTTTATAGTTTTTGTTTAATGATGGTAATGGAAGAATTGCAGAGCTATATTAATAGAATATTGACAAATTTTAGTTTCGTGTAGCTCTTCCGTATGATTATTAAATACATCTTGGAACACTGATGTCTGTAGATAGAGCTACATTAGTACTGACTCATTACATCATTTCATCATCATGATCTTAGATTTCATACATTTGCCATTTACATAAGTCCCAGCTTCTTGAAAGAAAGACTACACGTCAGCTTTGGATAAACTACATGGATGTTTCATATACTGATTTGTAACCAGACCTAACGCTTTTCAAAACTCAAGTCCTTTTCTTTCCTTACGTCATAATTCTAAAACTCCATCATTAGCAGGATTTTTCTTCATAGGGTATGGTATAAACTCTGCTATCTACACATCAGCTCTTATGAAAGCTCAGGTATCCGCATTATTATCTGCTGGGCAATAACTGTACTGTAGTTGCATGCGATAGATGTCATTTTGTGTAGGCGACCTATTTTCACTCGTATTATTTTGTCAGTGATCTTTTATGCTCAAGTAGAAGTAGATACAGCAGACATTGTGTATATCCTGTATTACACGAAGTATGTACAGTTCATGGATATGATTTTATTTAGCCAGATGAATGCAGTAGTCTTTAGATATGCTGGAAGTCTCATGTTTACCATGACTCAATGATTTTAGAACAGTGTAGTATTTTTCATTTAGTGTAGGTTACATTTCGTGTAAAGGATATTGTTTGTGAATTTATTAGTGGTACACAATTGACATTGATATTGCATAGAGGGCTGTGTTTTACCAGCATTTATCAATGAGTGCGCAGTATCTCTGCCATTTAACTGCACAGTAATTTCAGTGAAACTTGTGTGTGCTCAAATCTCAGTACTGGTATGAGTGGATGATATCTGTGCAGATGGTAGAGTTTGTACTCCATTCCTAAATATCAAGCCACTTCAGGTTATTTTGCATGTATCTGCAAGAGTCATTGGAAATGCATATGAAAATGAAATGAGAAATTGTAAGCTGTGCAATACTTCGGAATTATAAGGATTGTTGTCAGGATAAAAGAGGCTCTATTTGTTCTTCATGATGATTGCTATTTGCAAAAATAAATTAATTTCAATTTTTATGTAGTTTCAGGCTTGCTTTGTATTAATGAACCACTTAGTAATCATTGGGTTACCTCACTTTGGCGTGCCAATGGATGATGTAAGGTTAAGCTGTGTGCGATGGAGGGTATTCTTTTATTCCTACTTTGCAGTCTATATTTTTTGTGCATGTGCTGATATTTAATATTGAAACATACATCAAACTGTACGTGTGCGTTCAATAAACCTGATTTCGATGCATTACAGTGTATTAAAAATCATTCTGATGAATACTCTTT

>chr14:+:67907223-67911393

AAAGATCTGGTTTTATAATTTCCAAAACAACTAGAATTAGCTCTGGCAAATTTCTACAGCTCACTGAATATTCATAGAATATTCGAGACCAGCATAAAAATAAAACATTCCAGTTTTCTAAAACTGTTAATAACATTTCCGAAGATGTTTCATGAAATGCATCAATACAACTTTACTAGCAATCCCATCACATGTACACCAGAGAGATAACACTTCCACGAAAAGAAACTTTAGAGGACTGAACTAGATGGATGTAAATGGGAGTGCAACAGGTTACCCTCACATGAAAAATAGAAGGTACCAGCCTTCTCTGTCAACCATACCTGTAGCCTGATGTAATAACATTACAGCCATGGTCCTGTTACCTACTTTTTGGTGACATTCCACAGAATCTTAAAAGTTCTCAATAATCTCTGGGTAGACAAATTCCAGGTTAACAACCTATGATAAAGTGAACTTGTGATAGTACTGATAAACCTGTACAACCAAAAGTATTCCACAGGAAAACAAAACTTTAGAACTACACATCACAATAATGACATTACCATGATTCATGTTAATTGGCATCAAACAGCAATTTCCTAAAATTCTGATGAAGTAAAAACAGCCCAGGTCTACTTTCTTAAAACTCCACTTACAAGAATTGCAGGCAACAAGCACCTTTCATGTGCAGAGGCTTAAGAGTTCATACCATCATCACAAACAAGACAAGGGAGCATACGGCATTGGCATGCAATCAACTAAAGTTTATCGGCACCTTAGTTCTTCCAGAAATACTCTAGCCTTGTAATCAAAATTAGTCCAATACAATATTCTAAAACTTCATATCTGTGTAATTACAAAAATGCAAATTGCTACAGTTCATCAGCTGATTAATACAAGTATGAAGAACCATTTTTTCTAAATGAAATATGCCAGAATTTCATAACAGTAGTTAAATGTATAACTACAATACTTTACAATTTATCTAAAATATGTAATCATGTTTGTAGCTTTTATCTTCAACAGCAACTATGTTTCCTATAAATGTTTTGAATAAAATGAGATAAAAAAAATTACTGACCATGGAAATGCTGGTGAAGATACTATTTGCCCCATTACTAATGTAGCTTACTAATAACGTCCTTATTAAATTCTGAATACAACCTCAATACATTTTGTGAAAACTAAACTTCATCAAACTAATTATTATTCATTGTTCATAAAAAAATAACAATTCATAACTAACAAATTAACTATTCACCCTATTCAACACCATAATAAATGTTGAATGAACTTTCACCTTAAACTAACTGAACTTATTGTGGTACTATATTTTCTAAAACAAGTCTGCTACTAATACACTCTGCATCACACTTTAAAGTGGGGAAGAAATTGTTAGATTTAATAGCTGAACTAAATGCAGCAACAGTCCTTTATGTGACATCACTAAAATGAGACAACAAACTGTAGATGTCACACCCATATTATGAATATGTAATCACCACTATACCAAACTTCATGAACCCTTAGAACCAAATTTGTATACATCAAAAGTATCAATATAAACACTGTTAACATATATACGTATAATGAGTATAAATGAGTCACAAGAAAATCCCATAATGCAAATTATATTATATTTTACATAACTTACATAACATTAATTTAACAAGACAGTTCACACATCTTAAACTGTGAATTTCAAACATTACAAATGATGAATAAAAACTAATGCTATCCCAAAGGAGCTGGAAAAAAATCTGCAGACCCAATTAACTAATATGACCTATGAAGTTTTGTAATGATTTTACTTGATGTCTGGTGTACATTTGCACTGTATGAGAATGCAGTTTGGCCAGAAAATTAGTTAGATCCATGAGTAATCCTAGGAAATCATTTCAAGAATTAAAACTGATTTTTATCACATAAATACTCTAAAATTCAACTCTACTGTATATGTACTGGGAATCTTGTCAAATTCAGATTCCAAAATTCAGCTGACATACTATCTTAGATCTGGTCTGAGACTGAACACAAGGCACAGACTGCAATATGTATCCTGAGTCAAAATGAATTTGATTCAATGATCTTACAATATCACCCTAGTTAAATACCAGCAAGTTTTTATTTGATAGAACTAAATAGTGAAAGAGACGTTCACTAATTGGAATTTTTGCTTGCAAAATCGCTGTGGTAAATTTTTCATGCCTATTTTGGGACCATTCATATAAGGGATGACCACTGAATAATATCCTAAATTTCACTTTGTTTTCCATGTTCTCTTTACATACACATCATCTGAGTTCTTTGCAATACTCATTCTTTAAATATACAACAGTAATGGGAAAACTTACCAAATATAAATCCATAATGGCAGAATATTTCTATACTGCATTATGAAGTAACTATTTATACAGTACAATATGTATCCTTTAGATATATCTAAATCAGGTCAATAGAGCACTGGATGCTCTTAGACTTCACATGAAAATTCGACTTCCTTACAACTTTGTAAAGTGTCATTATCGTTAGCACAATTATTATTATCACTATTCAGTGTTCTTAGACCACTGACTTATGGTTTTTACCTCTTGAAAATTATCTTAAGGGTTTGCTCTTATAGTACACTGGCATAGTTTTGAAACATCAATACATCTGCATGTGTAAGTCAAGAGTCAACAAGCTGATTTTGCAGAAAAAAAAAAAAAAAGTTGAAAAGAGCACTTAACATAGCTATCGTATATTAGTATAAAATAGGACACTGCTACCAGCCTACCAAAGGCAAAATGCAACAAAGAAACCAAAACATTATGCTATCAAATCAAATTTGATATGCTCAGCATTATGCCATTAAATTTGATAAAACAGTCTACAATACAACAGCACCACCATATAATGTACCAAAATAGCTGTGAATTGAGGATTAAATTTTCATATCCTTTGTGCTTTTTTTTTTAATTCTCTTTTAACCACAACAGAATCGTTTTTTTCTCTCTCTCTTTTGAACACAACTGAATCAAAAGTAAATTTGGTCAATGACCCTGGAATAATCTAATTTCTAGGTGCCTTTCATTTTTTCATCTGCAGGATAACCTTTTCACCTAAAATAAAAAATGTATGGTTTCAGTCAACATTTTGTTCAGGTAGGTAGTCGCTCCTCAACTCCAGCAGGTATATATAAGCTGCCTGTTTATTCAGTGCAGCAATTACTTTGTGGATATCATTTATTGCTAAACTGTTTACTTTAGTCACAGCTTCCTTTCTACTGAAACCATTACTTTGAATTCTCTCCCCAATTTCCAGTTCTTGAACCCTATGATATTTAATTAAGAAGATCTGTAACACTGGCATTCTTGCTTCCCATCCTTTATGATTGGACAAGCACACTGAATTGGCGTGCACATAAAAACAGACAGCGCAAAAATACTACACTTGGGAAAACACAAAGAATGTCTTCCAAATACATATAAAAGCAGAAGAAAACATATCTAAATGCTGCAAGATGTAAGAAGCTGTCACAATAGAATACTAGTTACTTGTCCTAGAAATTAAAGTGAAAACTTAAGAACTTTTCTTATACAACTGACATCAAGGCAAGACACTGTATGCATGAACTTCTGCAACTGCCTTACACTTGCTACTTAGCTGCATTAGAAACCTAGATCAAAGTGGTGGTCACATGATAATGAATTTTGTACAATACAAAAACCGCTGGCAACCACCACTTAATATTTCTCTCTAAAACTCAAAGCAGGCTAAGAAGTTTACATGAAAAAGGAAACTTAACTCAAAGAACAGACAAGAAAGCAAGGAAGTTGTTAAAGACTGCGTTTTCAAGTTACACTTTCCAAAGGCAAACCAACAAACCTTTACAGAGAAAGAGCAAAATAAAAAACCCAAACAGTCTATTGCATTTTTACGAACCAATCTGATATACGTTTTCTCAAATATTACAACCATACAGTGATGCATTATATGGCTTGGATGAGACCTGAATTCAGCGACTTATATATACTACCATACAATGAAAGAAGCTGGAGGGAGAAGTATTTGCTCATGCTAAGTAAGTATTGTGTTTATTATCTGGTAGCTTAACAAGAGGATCAAATCCATATAATTGACTTCCATGGGGCTGGTGTGATGGGTTTGTCTTTGATAAACTGTGCTCTTAAAAAACTAATTAGTACTG

>chr19:+:352304-354575

GAATTTTGAGTTGCTTTATACATGTATAATTCATAACAAAAGTAGTTTGTTTCACAGGTACACATTAAAACTCGGTCCAATGGTAATACAGTAATTGGAAAATATAATTCTACCAAATGGATAAAACAAAAAAAAGGTCAACAAAACAAAAAGAAAAATTATCAAAATATTTTCATGCTGAAAATAATTTTGAAAGATACACTTCAATTAGAATTACCTCCACATTCTGCAATGTATTTCTGGAATGTGCTGGAGATAAATGTTCTTCCTTAGCAGTGAGTGTATTAGTTTACCAAAATAACTGCAAATCAGACTGTGATTTTGCTCACCATTTAACATTTAATGTAAACATCTCGGGGGGGGAAATTTTATATATAGCACAGCAAGTTATGAATCGGTTCTGAAAAAATAGATAAAATCCCACCTGAATCACACACGGAACTGAAAAGAATGGCGCATTCTATCTGAATGACAGACAAGTGGCCTAAAGAAATGTAGTTGGGTCATGATACTCACTTAACCCTTTACGTTCATAGTACTTGTTGAATGAAATAATATTCACTACATTAATTTTCATATGCATTTCTTTATTGGCCATATGCCATCAAAGCCTGTCACACTTACTTTTGTTTCAGCAGACCACTCTGTTAAAAGAATGAATGCAGGTAACATCTACAAACCATTCCATTTAGACTTTATACTGGAAGATTTCATATACGGACAATTATACATCCAAGCAAGGGGTTATACTCACATTGCAGATACAATTTTATGACATGCAATTATTACCTAACTTCAGCAATCACAAAAATAACGGCTTTGGAAATCTGAAAACAGTAACTAAATTTTCCCCAAGTGACACTAGTCTTCTATCAGAAAAGATAAGTTCCAAAATTGCATTCAAAGAAATATTCAAGTTTCAAATGTACAGCTCCTGTCATGTAATGTCAAAACTCATGCATATTACACACAACAGCACCATACTTCAATAAGGTTCAAATCAATCACTTGACAACTCGCAGTCAGTCTGTTGAAAAACATTATCATTCAACCCTCCTGTTATTACTATTAACTTAATCAGACCACTGAGAAGAGCATGACAACTGAAAAATTTAACACTATGCTTTGATAAATGCACAAACCAAGACAAAGTAAAATCATTTGTATAATTTTACAATGGTTTCTATAATGCCCTAGGCCAATCTGAAATTTGCTTTCCACTTTTCAAATACCTTGCAATGTTTATTCCCTCAAACTTCAATTAGCATAAATACTGCCTTACTAAAAATAAATTAAAATTACCTTGATTACAAAAATAAAATAATGATATTCACTTCTAAAAAGAAGACCAACTTTCTTTAATTTCTAAACACATATGGATGTACTTTTAAAATTATCTTATTATACTCCTTTCAATCTTGGTCTACCAAAGTTCATTATCCTTCAAGCAAAGATACTGCGAAAGTACTATTCAAATGAATTCAATATTTCAGTGATCTCATACACTACAAGCTACAAACAAAAACTAGCTGACGGCACTTTGAATAATACTGCATGTCATGATACTCATGAAGCTTTGGATGCACAGCGTGGCTGTGCTTAGAAATTACATACCTGGTTAGCCTGCCAACAACTTTGGTAAGTCTCTTTAGTTACGGGGTGGGGGGTGTGTGTTTACCATGCAAATATTTTTTTTAGAACTGCAAAGATGTAAATCCCTCACTGGCTTTTACAATGGTGGCCACTGAAAGATTAGAGTTATATAAATACAGTTTTCCATCTACATTTAACACAATTATAGCATTACCTATAATTCTCTAGAGAAGCACTTTCTTTTTATATGTACTACATACAGACTATGTACGCTTGTATTGTAAAAGGCTTACAAATAAAATACATCACTTGGAGTAAAAACTACAGCAAAGCACACCTAATGAGATTCATACACAGGAAGCTTGTTACCCCAAAAACTGCACATTCAACATTGTGCACACTTTCAAAGTATCAAAGTTTCATACCAGCTGGAGAAGAAATAACTTTCAAAAGCCTCTGAATTTCTCTAGCAATGATTTGGGATGGATAACACAACCAGAATGCAAACTATTGAATCTAGAACTTCAAACGCATGTGAATATACATTACGAGTACATATGGAAAAAAATTATAAATTTTAAGGTGTCTTATCCTTTTTTAAATAAATTAGCATGCTATCAAAATTTAGAGGAAAATTACACTTATGGAAGAGAAAAATTCAGTATTTCCTAGATCACCC

>chr19:+:900602-902000

GATTTGTCATTTTTATTAGAACAACAGTCATAACACGGACATTGTAAAAGCAAATAAATTGTATATATTTTTTTACCACACCATTTGAAAGCTCAAAACATAACACTATTTGTCCCCAATGAACTAAGCTATAGGTAAAGAGGATGTAAATGTTTTTATGGTAACACTGAATACTGTGAAAATTTGTGTGGTAATACTGAATACTGTAAATATTTGTATGGTAACACTGAGTACTGTAGATATTTGTGTGGTAACACTGAATACTGTAAATATATGTATGGTAACAGTGAACACTGTAAATATATGTACGGTAACACTGAATGCTGCAGTACAAGACTATGATATTTCCCTTATTTACTAAGTAAATATGTCTTCTTAAATGTCCTACTCATTCTTCCCTAAAGTGCAGCTAATGCAATACAAAGGTTGACAGTGGTAAATTTGATTTAATATAGTAAACTATGTCGATGGTAAAGCAGCAATTAAGTATGAAGCTCCCTTTTAAATTTATAAGGAAAATAATGAACACCATATTCTTTGGAAGCTTGAATTTCAAGTCAGTGGTCCTGAGGGCCTGTTCGATATGAATTATTAGCAGCTGAATAATAATTATGATAATGATTGATATGGACAGCTGAAAGTGAAGTTACTATTGTATTTCTATATTTTAAATAATTGTCCTCTATATTTTAAATGATTATCCTTTAGATTGTACGTGGTTATACTCAATTTTAAGTGGTTATCCTCTAAATTTAAAGGATTATCCTCTTATTTTAAATAGTTACTCTCTATTTTAAATGGTTATCCTGAATTTTGATGGGTTATCCTATATTGTAATTGGTTATCCTCTATTTTAAATGGTTATCCTCAGTTTTAATTGGTTATCCTTCATTTTAAATGGTTATCCTATGGTGCCAAACAGAACTCTGAAAAGCATCCACATAGTACACCTTTTTCCCAGCCGGCCAATTGCACAAGATTTGTGTACGCGATCTTTTATTGTTGGTATCGCAATCAAAGCCTTAGCAGCTACGATGTTCTTGCATGATCTCTCTTCTAAGAAGTTTGCAGAGTGGGCTTCTGATAAGACTGAAGGTACTGTGGGTGGTTGTCAGTGTAGCTCCTGTTAATTATACCTTCTCATACCACTGCTGGGTGCAGAGTAAGGATCCCTTGACCTAGGTTCACCTCTTCCATCATCTCTTGAAGGCCCAGGAGCAGAGTAGTAATCACTCTTAGGCCTGGAATAGTAGTTGTCACCTGCAGGGGAACCTGGCCTGGAATAGTTGTTTCTTGGAATTGGGGAGATTTGGGGACGCCTGGATGTTAAGGGTTTGGCATAGAGATCTTCCACTGGTCTTGTTTGTGGACGTCTGTAGGGATCTTTGGCCAGAGGTGC

>chr19:+:3854148-3855637

AATTTATTTTCAGATTAGAATGTTTGGTTGGAGGTTGTCCAAGAATATATTTTTATCCACAATATATAATAGGTGTTAAACCTGGTGGTCTTCGCGATCAACCAGACTTTTCAACCACACGCTTGCCGTAGTTTCTTGTAAACAGCCTTCACCTCGCTTTTTTGTAATCTTGACAACTTCTTTATATCTCGTTCACTCATATAAAAATTCCCCAGAATTCCCATCATCACAATAATAATATTCAAAACAGCAGTTTATGTTCATGAGCTAGAATATATTTTTGTATTGATAGTAGTGAAGATCTAGACCTATGGGGGGAGAGCAGCACGTCCACATCATTATATGGAGAGAAAAAACTGAAAACATTGTCCAGAGAGGTTGGGTCTGACTGAACATTCTGAAGTGCATATGGAATCACTGGTAGATAGGTATCTAGTACTTTTAGTGAAGGAGAAAGGAGGATGGACTTTGCCAAATTGGTAACAGCAATAGCATTCTCTCTATTATGGTTACCACACTGATAAAGACTGCTGGGTACTAGGGGCCAAGATATACTTTATATATATTGTCTATATATGAGAAAAAGAGAGGTCAACTTTTCAATATCTTGTCAAACCATTTTTACACCATTTCAAATGAAGATGCATACAATAAAAATCAAAGAGTACAGCAGATAACACCAAAGAGAAGAAAACATAACCATATTGCACTAAAGAATTTTTCATATTCCTACATACACAGACAAAAGGAAAAAATGCTCTTCCAAAACAAGTGAGATTATGTTGATCCAGACTTCCATCAAATGAAAACAAAACATTTGAGTAACCCCTTAGTATCCAGCACTCTTCTACCTAAGGAAAAAAGTCCCCTTTGCCACCAAACCTCTTCATTTCATTTTTCAACTCCTTGAAGTTTGTCCACAGAGAGAGAGAGAGAGAGAGAGAGAGAGAGAAATTCCTGGGGATGGACCGATCTCGATCTCGACGCTCTTGAGGGAAATGTAATGCTAAATTCAATTTTAACACAAGTTACAATTTGAAAAATGCCTGACTACTCTTTAGTAAGAAACAAAGCTAATAAAAAGATGGCAAGGAAATGACATATGCCAGTGCAATACTAGTCGTAACTTAACGAGTAATAAAAAATAGGACTCTCAATGCATGCAAAGATAAATAGGCTGCCAAAAACAGCAAAGGGAAAGTTTACGAAGCTTTGAAGTTACAAAGATCTATTGAAATAATAAAGTTTTAATTCACCTATATTTTTTCCAGTATAAATATTAAAAATGCTTGCACAGGAAAATTTATTTTTTATTTCTTTTTATATAACTCATGGCGTACTACAGTACATTAACACAATAACTTCACTTTCCTTCACCGCTTTCATATCTAATGATGCATCAGTGGGTATATGACAGGTAGACAAGGGGGCTGGAGTTTATATAATAAAATTCTCTTCTTCATTTAGACGGTATATAATTCTCCATATAC

>chr19:+:21624520-21624732

GGCAGTAATAGGAGAGGGCAGGAGAGGAGAGAACCAAGCCTTGTCTCCCTCTCTTTCTTTCTCTCTCTCCTCTCTCTCTCTCTCTCTCGCTTTCTCCCTCCCTCACTCTCTCTCTCTCTCTTTCTCTGCCTCTCCGCCGTGCATTAGTTGACCTCCTGGACGCCGACCCTCCGCGTTCGCGCGCATCGCCATGCAGCAGCAGCAGCAGCACAG

>chr19:+:21637194-21638008

AACTGCCATCCTCTTCATCTACACATCCATCCTTTTAGTAACAACTAAAGCCCAAAGAAGTCACCATGATAACCACAGAATTGATGCATATGACTCATTTCATGACAAGGAATTAGAACAAAACTTAAACAGCCTTCAGCATGAACAACTGTTTCTACCTAAGAAAGATCATTTATCAAGTGTATCTTCTGAGCTCAATAAAGACAAACTGGAATGGATAACTTTGCCAAGGATGAATCCTTCACAACACAGTGACAAATTAAATAAAAACCTTCCATCAAGACAGCCTCCTTCCATCGATAAAGATCATAAACAGCCTGAACATTTTACACCAAAGAAAGATCATTCATCAAGTCTATCTCCTGAGCTCAATAAAGACAAACTGGAACGGATGACTTTTCCAAGAATAAATCCTTTAACACGACGAGACAGACCACAAAAAATGAATCACTCAAGACTGCATCACCCCACCAACAAAGACCATTCAAAGAGGATAAAACTAAGAAATACGCAATCATACAAGAGACGTGCTACAAGGGGTTATGGGGGAGTGTCTAATCAAATGGAGCAAGATTCACAGCATACATTACAACGACAACTGCACCCCATAACTCCTGATATATATCCAAGGAAGAGGAGGATTTCAAGATTCCAACTGGGGAGAAACAAAGGAGGCAACAGAGAACATGGACGTTCCCAGGGAGTTCAATCAAAGAAAAGAAGGAAACACATGAAAAATGGAAAATACCCTAAGAGCCTGCCAAAAAGCCAACACAGGTTAGACCTGGGCTCCCATCAAAGTCATGAACAAAAAG

>chr19:+:21638347-21639926

GACAACATAGAAGTGCAAGAAGTAGCTGCACAGGAGGTAAAGTCTGGAGGAGGGGTCAGTGTCGATGTCCTTCCTTGTCAAACTGGGATGAGAAGAAATCAGAATGTGTCTGCATCTATGGAACATACAAAGATGCTAACGGTAACTGCCGATCATTCATTTAAATCCACTAAGATTTGAAGTTTGAAAGAAACCAGACAAATAAGATTGGACAACTTTTGTAAAGTACAGTCTTTCATCCTCAAGGACTGCACATCACATTCTGCATAAAAGCACTTAAATACAATTTTAGTTTATAAATGAAAAATAACAAATATCTCTCTAACCTCTCAAGATTATTAACAAAACAATAGAATTGTTTTTTTTGGAACAGGCTTCTGATGTGTAAACAGTGAGATCTAACACCCAGAACCTGACAAAACAAATTTAATCATAAATTACCTGTCCTTCTCTATTATGCAACTAGTTGCAGCATTTGTTTTGTGAACTTTGAATTGTATAAGGTACATGTGCTGTACCTAGTCTGTCTTATCTTTCTGTAATATGCATGAAAGGTAATCCTAACAAAGAAAATATATCAAAGTGGCCAAAAAAGAAATTCCACCAAAATTAAAGTATTTCTGTAACACTTAGTCACTTGACAACACAATATGTACTTGGATTTTGCTTTATGTAGTTTTAGAGAGCAAATACCCTCCAATAATGTTATCTTTGAAAACTGTTGTTGGGAGAGAAAACAGTACAAACTTAGCCCGCTAAAAATAAATATCCAAATGGACAATTACAGTACTACAAAATGTATGTTTAGAATATCTTGCAGAAGATTAATAAATTACAAGGAATTACTTCAAATTATCTACCATAAGTGTAGTTCATAATGATTACTGTGTGATCTATTGTATTGGCTCTATCTAGAGTTCTTCAAAATAAATATCTAATAACAGTCCCTTCTCACTGCAGATGAACTTAGTTGTCCAAAGATAACCGAGGAGTTCTATTTGTACCCAGAAGGTAACAAAAAGACCGGGTATTAAAAATGCACATTAAAAGGCATCACTGTAGTTATAAGTAAGAATAAGCCTTGCATATACTTACTAAAATAACAAAAAGTTAGTTACCATAAGCTCATTACTAACATTCTTCTATAAATAAACTATCAGCAAAATGAAAACCTCTTCAACTGGAATACTTCATTCACCCACAGTATATTTTCCCCAACACATACTCATATACAGTATACAGTAGTTATACACAATCCTACAAGACTAGTGAACAATATCTGATAAATATAAGTTTACCCTATAATGTTACAGTGCTACTGTAACTTCAGAGTTAAAACAGCAATGAAATACCCAGTAAGAGAAAAATACTTTCCAGTCACTGTACCTGGAAAATGAAAAGCCCACTACACTAACTTATATAAAACATGAAACTTAGCAACAAAACAACACTTTGACAAATTATTAACAGGATACTGCATGTAAATTGGATATACAATGCAGACAAGTATACTGGTCCCTTTGTTTCCTTTATTAACTGTAAAATACTGTATGGATAAAACTAAAAAATTATAAACTATG

>chr19:+:74207725-74210539

AATTGGCCATTCTTAAATTTAGTCTATTTTAGATAGTATATACATATTTAGCTCGGATTGTGAGGGTTAAGTATAACCTGTGAGACCGACTTGCAGATGCTAGGATCACTCCCTCAACGAGAATGAGATGGTATCCAAGTCACTATTTGCTTCCCTCTCTATGGCCTTGATAGCAAAATGGGGGGAAAGAAGCGAGAAGAAAAAAGAGAAATAAGACCTTGCTAATTTAGATGAAAAAACAACACAAATCTGAATAACTAACAGTTAAGAATTTGGTGTGTTAATGATGTAACAGTAAGGGGCTCCACTTGCAGAGCCTTTAATTACCCGGGAGAATACTGGAGCCCATTTTCATTTAAGAATGACTATCTCTCAGATATCCAGCCACACTGCAAACAATATTGCATTACTATACATTTGGTAAAAGCTACATCCCCTCTCTTCAGTTACAATTTCCAAATAACTGGAGTATCTATTTTTTGACTTATGAGACGTTTCAGGTGTCATGTATAGATGTCTGACACCAAGTGATAAACATTAGGCTGTGACTTAGCCTTTGTATGATAGTTCTATTCCAGTCTTGGATCTGAATTCTCATGCTTGAAGGTATAACATACAAGACCGCTTTAATCATTTAATTTTTAACTTCCTGTTGTTTTATTCACTTACTATGCTGTTATTTACAATAAGATATACAAGGTTTTAATTGGTTCTTATCTTCTCTGTTTGTTTTACATATAATTATGGGGTTCCAAACCTGCGAAAGCTGGTTATGCAAGTTCCAGATTTGGTTGCTTGTATCTAAAGAATAAATGGTCTTATGAGCACTATACTGTACTACTACTCTAATAATAATAAGTACATGCATACACACATACATACATGCACAATTCCTGTTACAGACATTAATACAATAATAATAATGGATATCTTACTGAAAAGAAAACAAATCAATAAAAGAATTTCTCACCTGTCTTCTTGGTAAGTTCTCTTCCCAATGATACTTGAACAACCTACATCAGAAGGTACAATTAATGGCACTGGAGCTCCTTGTTTAATACTGGCTTCCATCATGATAGGAAGATCAATTCGATTGTCCCAAGTGCCCAGTGCATCAAAATTGAAGTCTTTCTTCCTCCCATTATTAGAATCACTTATCTCCTTTGAAGATGCATTCCCATCAGTGTTTTCCACTTGAACTTTAGTACAGAAAGCTCCGACAGGGAATCTTCTGGTTACTTTTTTACCTGACAATGTTTGAGCAAGTGTTGCAAACCTTCTCTCGTGACCTTTACTGACATAAGTTGAGGTATTACAATTTAATATTGGTGCTTGACAAAATGACTTTGCTTCCAAGTTGCCAAAAGTTCCTAAAAACACATTTTGCAGAGTTCTCCTTTGGATGAAAAACGATTTATTCATCCTAGGTTAATTCCTTACTGTGATGTCGCTTTCCTGCAGAAAAAAAAAAATAATACAGTACTGTAATTAGCAACTTACAGTATGATGTTCCTATGTTGCAATACATTAGGTATAGTAATTATAGATGTCAACAAAAGAGGAGGTACTATCGACTTGGAAAGAATGTTACTTCGACACCAAGGTTCCAATAAAATTGATAAAGATACTTAGTCCACGGGCCGAGGACTGATTTTTCTCATTCTGATATGGGCCATTAAGTTATATCAAAATAACTGTACAGGTTTTTATTTGGAAAAGAATATTGTGTCCAAACAGGAATTTGACAAAAGGATATTTCCAATGTGGTTGCTTAAATCACATTCAACAGGTTTTTAAAAATATAATTAAAAGAACATTAATTAAAAAAACAAACTGCCATACTAAAAGCAGGGCTGACCTAACACTAGTTAAAATAATGGATACTACCGTTAATAAACTACAACCAGGAAGGTAAAACTGATGGCTACTATGTAGTACGGCAGCCTGGTTCCTGCCTCTCAGACTGGAATACTATTTACCACCTTTTGTTTATATTTCTTTTATTCTTGTATAATCAAAATTACCATTATTTATTTATGCTCCTACATTTATCCCCCAAGGGCTGGTGCTAAAAGAGGCAGCTCTTCTGTGTGTAAACTGGTAGCTACTGAACCAGAGGGGCGTGGCAGTACCTAGTAGTAGTCTTCGGTTTAACCACCAGAGGTATTTCACTGGGGGTAGTAGGCTGTGAGTGTATCTGAAGGGGAAGGTTAGGTCAGGGTTATTCCCTGTTGCCTACCCTAGGCCTGTTTCACTAGTTTTGATATTCCCTAGTACCTAGGTAGCCTAGTATATCCAAAAGCCACAGTTTCACGCTACCTACGGTATGGTCCTCCACAGTCAAAAAGAGATTATGAAATAAGCTAGGCTACATGTTAAATGGAAAACAAATCACACTCCTACCTACCTATTAGTCAAAGCATCCTACAGGCCTACTTTACAACTGCACTCGATTAGGTAACCAACACGTTAACGCCTACATTAGCCTAAGCCTAATCAGGCTTGCTTTTAAATGCAATCAAACAAAACAGGCTAGGAAAGGACTAGCTAACCTTAAAAACCCATCTACTATTTACCTAAGACTGTAAGGTACCTTCAGGTAACAGGAATGGAACCAGCAACTTAGCCTAATACTACTAAATTAACCGAAAGCACCTTTGAAATACGGCGGCCTACAAAACTTAGGCCTCAGTCGGCTCACTAAATCCTTGACAGCAAACCTACGTCTATTGTGAAGGCCTCTTCACCCTTTAAGACAACTAATGCATAAAATAAAAAAATATGTGGCCAACCGGGTTGACAACAGACCGCAATT

>chr18:+:17488409-17493008

TGTTCCACTTTTAATATCTTTTAAGGTCATACTGTACAGGACTGGACAGAAAAGAAAAACAGGTGTATTTTCACTTTTTTGATTAAGACACTAAAAAAAGTACACTTAATTTTTATCAGCATTTTAAGGGAGACAAGGACATACGTACTTTTCACTGTGGCAAAACAGTACTATTTAGAATTTTTTCTGGATTTCCCAACATTTCAGTTACTCTTCCATAATATCCATCTTTCAAAGGCAAGACTTCAAAATACAAATTTCCCAAAATATTTTCAAGAGCAAACAAAATAATTAACAACAGGATTTGGCAATGAAAATACACCATGACCACAACAACCACACATGGACCCCTATAGAAAAATATGTAACGTACTCACTGAACGTAAACTGATACCCTGAACAAAGTAGACAGCTTTGGGATTATAGCAGAACAATACCATTCACAAGTTAAGAATAGAAGATGGTTCAACAAGTAGCCACCAACCGTGCTATAAAGGAGCATACCATTCGCAGAAAGGATTTACGATTTTCATGAATGGGAATGCTAAATCTGATCAAGTTGGCAATGCTAACTAACACTCAAAAATAAATCTTTTTTTTCAACTAAATTTCTGCCTACCTTTATTACACACTTTCAATAAATACTACTCATAACTATATATGTATACCTTCTATGTAACAATAAATTCGATAAAAATCAACTATACATAAATATACATAGCCAAAGGGGAATATGGTCCTTTGGATTTCGGTGCAATGGATATGCAAGAGTTACCGACTGGAAATAAGTGGTACTGTAATTCCAGAGACACTTCTAGTCATCACTGGAAAGACTGAGGATGAGTAAACAAACAGCAATTTGGAGAGATCTAGCGAGTCAATTTCCTATCACTTTACTATTACACAATGTTTAGTCCTTACTTTGCACGTAACTTAATTGCATATGATACAGAAGAATAAACTGAGTAATTACTCATTCTCATAAAGTTGTTTTCTTCAAATAAAGTCAAATTTTTACAAAAGAATAGTTTTACAAATATTTCTCATCTCATTCTCAAAATATAGAAGACATACTATTACTGCTTCGCATAATTATGATTATGGTATGGGTCAGAAATGTGGCTCCACTATAAAAATATGTCATTCTTTCATCAAAAAAAAGGTACCCAATACCATTGTAAGTGCCTGAAATATAAACTGTGCCTAAAGATCTTTTCTTACTCATAACTCACCTACTTTATCGTTAAATGGTGCTTAAAAAAAAGCCAGGAAAGACGGGATATTATCATTTGAATGACCTGCAGTTCAATGAGCTGACCAATTGATTCTCTTGTGATTTACTCGGCTAACATAAACTGTGATGAAATGTTGCCATCACAATAAGTTAAGTCAATAAAATTACTAAATTATCAAATTAACAAAAGCAAGCTTCTTGCAAAGCCTTCAAATACAAGTTCCCCAATGACAGAAGTCTTGTTGAATGTTCCCAAAGGTGGACTGCAGGCTTTAAACATTCATTTTCTTAAAAGTAAATGAGAAAGCAATGTTGCAAAAGTTACTTAGGTACAGAGAAACAAAATAATCACACGAGCAGTGCCACTTGAATGGAATTTACATTTCAATGTATTTATTTTCATCTAATCTAGTGATTCTTACACGAGTTCATTATATTCAAAAGAGAAGCCATTGAAATTTTTGTAACGAAAGTCTCTACAGAACATCTGAATTCTTATTACATTCCATTGATTTAATGTTCAGATGAAGCCACTTCATATTCCATTCATTCTTTTTAAGGATGAGGTGTATATGGTCTAAAATCCTTTGTAGTATATCTTCCACACAACTACTTGTTATTCACCGCTAAAATAATGTACGTTATATATTTCACTGGTTAAATTCTACAGTGTTCTGATATGAGAGATAACTGGCAATGGTAAAAGTAGTTTCCAGCAAACACTACATATTATCTAAATGTATAATACTGAAACAAAAACCTGATCAAATGAAATTACAGTGCTAAACTACGTGGCGGAAAGTGAAAAATCCAACAAGATATATTAGTGTGGAATGTTAAATATTTCCATGTACTATTCATAGGAAGAACAAATCAATGCTTAACTAGGACCTGTCACTATATTACACAGGGACAACCAACTAAGAATCTACCAAGAAGATCATCCTGTTCTTCAGTGGATAAAATAGGCCCATCTTAAACTTCTGCCAAACAAAACTCTTTTCTCCTCATTATGCATAACAATAGAACAGCAACTTTCTAAAGAAGACCTCATTTTAAAATCACGCTCTAGCCCTCACTGACAGGCTCTAATGAGCCTGCCTCTCAGCATGATGTCTAATAACTAGGACCACACGCAAGCAAGCAACATTTGCATATCAGTCACTGTACACATTTCCCAGGAAGGCTTTAGTTATTACAAATATCATGATGCCTTACAGTTCAACACTTGTAGCATACCTAGGGAACTGAGGCGGCACTGAAACCCTGACTCCTTGCATGGTAACTGCATTTTGCACAGAGCAAAGTTCGTGTAAAATTCAGCCAACTCCTAACTTCATTCCAGCCACCAAAAGTTCTTATCTTTGTTGAATGCCAAATGAGGTCTATTTTATGCAACTGGAGCCTCCCATCTCGTTGCTATGCCTAATCATTGCTTTTCTTTTTTGCAACCAGCAGCTTTGAAGGTGGATGCTATGTTGCAATCTTAGGATGGTCTCGGGGGTCCACATCTTTCCTCGATAGTGGTTCAACATGACTGATTCGTGACTCATCTTAGACCTGATGTTAACGTATTCCAACAAGTTGTGCACTCTTATGCTTTAGTGTTTTCCTTCTACCTTCTAAAACAGGGGTAAAAGACAACACTACCCGATGTTGCTCCAAGTACAACAAGGGCCCACCCTCAAATTTTGCCACGCCAACAGGATAGAGGTCTGTAACTTGCGTGGCAGAAAGTCACTATCTCCTCTCTCTGCATTTCTTCTGTAAAGAGCATAGTTCTTCATAAATCTCTTACATGGTATGCATACACGAGTGCGTACTTACTAGATTCTACCTTTTTCTGATTCTATCCCACACTGGCTGTCCAACTGCACTTCTTTTAGACTTAGCCTTTACACCCACTAACACAGCCCTTGGACTAGAACTTGGTAGTCAAGTAGTAATCGTTAACATTGCCTGTTAAAACCCAGGTGCCTGGTCTCCACAGATATCACTCCAAACGATTCACTATCCCTTCCCTTTCAGACTTACAGGGGACTGACAGAAAGAAGTTTATCCTGTGTTCTGTTAGGGCTCTAAACAGCGATATTGCCACCTAGATTGTGAGAATACCTCTAACAATGCTTATTTATTAACCCTTCATCTTATTGCTGACCAATCTCCACAAATTTAGCAGCTTTCACCTTCATACGAGAACATTAAGCTTGGGGTCTGACTAAGTCATAATTACTAAACAACTTCAACCCATTAATGAAGTCAAACATACTGTGAAAGGATTAGTTCATCTCCTTCAGGACTAAAACTTAATGCACTCTTTGGCCTTCCTATGGAATATAGTAACTTTACTTTCAATTCCTACCCTTAAAATTTTTCTGTCCACCTTCAACTAATCTGCCAGCTTCTAAACCAGCCTATGCCAAGCTAGAAATGCCTATTGCAGAAAGGTAACAATCAAGCTATACAACATTTTCTTTTAACAAATTGTCAATTTTCCTGATTCAGGTAAGACTGTATATCATTTTCTCACTTAATAGCATCACTTTATTGGAGAGCTGCAATCTATTCGAACTGCCTCCTCCCAAAATATATACTTGACTCAGTATATATTTCTATTTACTCCTTCCTGTAAATATTTAAACATTTTTTTGGAAACAAATTTTGTATTTTCTAGATTCAAACCATTGTCTTACACTGAAGTCTTACTTTCCTCCCTAATGACTGGGGCATATACAGATCAACTCGAGACTAATGCTTCACACAAAATAAAAGGGTGAGAGATTTGAGGTGACCTAACTTTCAGGCATTTCTCATAACCCCCTGAAGAACACAATCTTCTTGGTAATTTCTTAATTGCTTCTTCATGATGAAGATAGGCTCTAGTAAGGCAGCAGTCTGCATCAACTAAACATTTTTTATTTGCATTTTTTAAACTCTCAAATCTCACATGCAGGCTTCAGCTACAGAAAGTTATGTTTCCGGTTTTCTTTTATCATCAACAACTTGTTTCTAAAAACTACAATAACATTGTAGCATTACTAACCACATAAATCAAATAAGTATGTTAGTCCCACAGGACCTATGTTTCTTTGTGTGTAATGAGTCAATTCCAAATAGGCAAATACAGTAATATGATACTCAGGAAGCCACTTAGTTTCAGAATTGTCATTAATCTGATGAGTTTGTCAATTTCCCCAAAATAAATACTCTTATAATTACTCAAAAAATTCATACAATTACTCAAAAAATTGAAAGACTGATAATGTAACGATTTTGAACAGTTGCATGTGTGATATACATACTGTAATTTAGTTTACGAACTCAACTTGATTTTTTTCTAGAGACACACAATTCTATTTTTACCTC

>chr18:+:82227219-82230741

AATATTACAGACTAGACTTCTGGCCACATCATGAATATGGTTATGCAAAACTTGTAGCAAAATAGGTAACAAACACTTCCATTATAATTCTATAAATATCTGAGTCTACTCCACAGCATCCTTTCTCTAGGGAAAAAGGGAATGAGGCCTGCTTCAACTGTCTAATATAGTACAGAGAAGTCTGAAGCATAATTCAAGTTGAAATGGTCTTTTTCTAGCGGATTTGAAAAAAAATACAACTGGAAGAATGACACAAGGCACTTTCATACCTTCCCATAAGTCTTACACAATATACAAATATTGAATATATTTGCGATATTTGCGAGTCACAAATACAGTACAATAGCATCTGCGTATATAAAATTTCTTCACACCCTTTGATTTTATTACTTAGAAGGAAAATACCATAAACTTGTAAGCATCATGATACGTATACATAGCTGTTGCTTAGCGACGTAAAACCTCTGGGAAAATGCTACACCCTTCTTTAATGTTTTCCCAACATAAATTAACAAAAATACTTCATAGTTGCTGGAACTGACTGCCTTCACAAGTCCTTACTATTCTTTCAACAAAGGGCGATGCCCTTTCTTACTAAAGGTCTCAGTCACTGCATGGTGAAGGAGAATTATACAAAGTGGAAAAACACCCAAACATCTAGTGTCAGCAAGCTACAATAAAACAAACTGTAGCATATAAGTGAAGTTGTACACTTTGACAAAAGACTTGGATGCAAATTCTATGTTTACTCAAATTAAATATGATTTCCATGGGCACAACAATTCAATCATGACTCTAGTATTTGAAATCAATGTCTTTTAAAACATGCCAATGTAAATTTTCTTGATATGTGAGGGGAATAAACAAATACGTATTACCAATATCACTGTGAATAAATAACATACATACAATAATGAAATACCTTGAGACTCAAATTTGATTACAACAGTCTATTGCGTTCTTCCTAAACAGCAGCTCAACTTCCACCAGAAATCAGTGCAATACTAAGTGTATGGTTAGTATGTTAACAACTACTATAACTTTTAAATGGTCAGTGTCAGCAACTGAAGATAATATTCATCTCATTACTTCACACAGTAATCTATCATCAGTATGCATAGAATGACTGTAAAAAGATGCAACTGAGCTCCACACCACATTCCTTTATCCAACAAATATATCTTGATATTCTATTACAGTACATACACTGCGTCTGCCTTTCACATTTCTTTATTTGAAACCTCCAAAAAAAAAATCACACAAAAGATTTATTATTGACAGGAAAATAAATACTACCTCATACACGATCATTTGAAAATAGCCCTAGCTACCATGCTCTTCATTTACAGCATGTTACATCAAAAGTTAATGTGCTGACGCTAAGTGTATTAGAATAACTTAACCATTTAAATACATGATATTCTACTATAAATATACAATGACAATAGGTGCAGGTGGTCTGCCTCATCATCAGTAGCACAAATATAACAAAATGTCAAGGTTCATGTTAACTTGAAAATCTTCCTGACCACCACCTAGCAAGCACTGCGCACTGGCCTGGGGTGTGACAAAACTGAGAAAAAGGTACCCTTGAATATTATTTTGACGTAAGTGTTCTCGATCACAATGAAAAACATAATGTTATTAGTGTAATTAAGTAGATTAACCTAACCACTGAATTATTTCAGAGAGAAATCAAAACTTCATGATATGGTACCCAATTTTGTGTGATGTACAGCAAATATAATTAGACTATGGAATAACATGTATCATAACACAATAACAGCTGCAGCGAATATATAGGTTGAAATGACATATGCCTTAATATATAGTATATGTATATACATGCATTTATTTGTAAAACAGAAAGCTGCAATGTAAATTAATCTTTTATTTCTGTATTAGTAATTATAACATCACTAATCATCAAAACAAAAGATACGTGTAATACTACTTTCAAAATGTAACATGACCACTCAAAGTATGTGATGCCCATACTGTATAACAAACACTACATTCTGATAGATGAAAAAGAATCCTTTCCAGGCACTTTCATATACTTATGGTGTACACAAACACCTGGGGAAGGATTTTTTTCTATCATCATGTGAAGCAAGCAAAGCAATCTCAAAATGCAGTATTTCAATAAAGAATACAAGTTTGACATTATGAATAACAAACTCTTAATTTTTAAGTCATAAACCCATAGTTTTACACAACCCTTAATCCAACTCAATCGCTTGATGAAGACAATGCGATACAACATAATTCATATCTCTGCAACTGAAGATTCATGTCTACAGCAGATACCCAACCCAGAAAGAAACTTCAAGTTGACTGATGCCAATCAGTAAGAAAAGTGACAAAGAGTTGGACAGTATACCTTTTAACATTCCTGGTAGTAATACTGGTCAACTGGTGCTATAAGAAATGTTCCCTCGATGTGAATAACAAAGATAAAAGTGAATATATCATGAATGAACATTTACACATCCTTAATAAGTACCCATTTTACCCTTTATGACTTTGCAAAAGATTCAGGAGATAACACATTCAGAAACAAGCTATTACAGCACTGTCACTTGACACTGACAAGTTGCAATAAGGGCAACGGAGTTTGCTCATGATCAAGCGAACAAAAGATCGTGACCTGCCTTCCATGAACTTCTCTGAGAATGACAGATAACGAGAATTTGTCAGTTACAACTCTGTAATCTCCAGTGCCTCAAAAAGAGAGGTTACTAGCTCACTTATGAATCAGATGACTCTGAGGTCAAATAAAGACACTTAGTAATGCGTGAACAGTATGGTACCAACATATGATGATTCAAGGAAGCCAAAACTTCCTACACATACAAAGTAACCATTCTAATTTAAGAAATTATATGAGCAATTATTAATTTTGTTAATAATCAGAAGATACTACACTTTTGATCCAGAAGGGAAACAGATTCATAAACCAATTTGAAAGGTTAGAACCACAGATGTGGATTCATGACTGTGGTAACTTCTCCTTCAGTTAACTCCAAAATTCTTCAGAAAAACACCTTAACAAAGAGTCTTCCTTCCAGAAACTGAGAAACTGAGCACTGCACAAATATTCTACTCAGTTTGGGTCCAAGATGACATTCACTCAGCGTAGCTGAACATCATGTCCTGATTAACATACGTAGCAGGAGTTATATCAAGAAAAAAAGAACATGTAGATAACAATACGGAATGAAAAGCACTTCCAAGAAATTTTATTCTAAATGAGCAAAAATCCATAAGGAACAAGTCTAAAATCCACAAATTCCTATGCATCCTGCAAAAATACAAAAGAAACAGCATCTTATGAAAACAAACAAATGTGATGCCAGCTCTGGATTTTTTATTCACAGCAGTACAACCACCAATGCATAGGAACACACCAACCTAAAAATGCATGCCATCCACCAGCAGGTATGCAAGTATTACTAATTACTGGATACTGTCTATCAAGGGTACCAATATAAGGCAGCTGGGTTCTCGTGTAAGAGCAAAC

>chr18:+:82230863-82231546

TTGAGATGCATAATAACTCTAAAACATGGAGATGGAAGAACAGAGGGTTGCAAAACAAGTAAATTAAAATACTGACATGGACTAGAGAGTAATAGTGAACTATACAAGCAATTATAAACTGCATAAAACTATGATTTATCAAAGCAAATAAACATGTAAAACTAGCTTGAAAGTCAATGGAATGTCTTTATATACAAAGAAAGAGATAAATTCATTCTCACTAATCTTAATTGAAAATCACAAAGGGACTTGTACCGCATAAAACCTGCCAAAGCTATGCTTGCAGAAAATACAGCGCCAGACTACCTGGGAGATTTCCGTGTTTCTAGATCAGTACTCTTCATTCTGGTGAAAGGCTTAGCTTCTCAATATCAATAAGACATCATCAGTGGAGAAAACAGTTGATTCACAAGTTTAAAACAGAGACCCCCAGTCATTAGTATACGAGGATTTCACAATTCAGAGGAAAAGTGACTAACTCGGGTCATTTCCCGCACTGCACAAAATAGGATTGTGTGTAGTTCCCTATTAGAAGCAAAGATAATATACATATATACTATGCATATATACATCAAAAACTGTCATTCATTAACAAATCTATGGTTTGTAATTAGACACTGAATGGCACTTCAAAATGATCATGAACAAAACCTCGGATGCACAGTACGATTTCCATCTTGCCAC

>chr18:+:85142054-85142231

GGCAGTAGCAGCTCTTAGCTCGAAGGGGAAGTCAACTGAGCCTGTGGAAGAGAAGTTACACAGTCCAGAGGAAAGTACGACTCGGCGAGGACACTCAGAATCATCAACATGTCTTCCTTCAAGCGATTCCCACCAGCTCTCTTGGTCTGCTTGATTCTGGGATTTGCCTGGGCGCAAG

>chr18:+:85143751-85143804

TGAATGGCCAGACTCCGACAACATCTATGAGCTCGACACCATCTCTTCCAATGG

>chr18:+:85145329-85145421

GGTGCTGCCTTGGAGACTACAGGTTCCCCGCCCGGTGCAACTACATCTTTGATAACGAAACGTTCACATGCGACGATGGGGTATGGGAGCCAA

>chr18:+:85146327-85146946

GGGATCCCCCGATGATGGCATTAGCTACTTGGGTCGGTTAAGGCCTTAACCAATCTGGTCGAGGAAGAGGTTTATCACCGGAGATGAAATTAGACAAGTGTATTGTGAGACCATCATATGTCAACACTAATTAAGAACATGAGGTGATGATATCGCAGATGCTGGGTTATTAAAATTAAAAGGGAAATGTAAGCATATTCTTCACCTTAGAGAAGTGAAGAATATGCTTAAATAAGACATGGAATATTAATTTTCAAGTAATTAAAAATGTTTCCAGTAAAGTTAATATAAAATTTTCATGTGTTGCTTGCAAATAAATTGCCCTGTGTACTGCACTGCTTTGTAAGGCGTGAGAATCAGCAAGCATTTATTTTGATTGTTTTTCCAACGGTTAACCATTCATACTTATTCAAACAATTGTATAATCAATTATTTCTTTCATGAGTGGAGAATCACTTTATTATCCTTTGATTGCAGTAGATTCAACCATCTAGATATGAACTTTAATGTTATCACTGTTTTCCAACGTTGGTTTTCATAAGATGAATTGATGATTTTGAGTTGCTATTTCACTCGTGTAATCTTGATTGGTAATAGGATGAATAATAAAAATAATTGGC

>chr39:+:1294973-1296423

CAACTTTTAAATTTATTCGAATCGAGTATAAAGTATGCAGACTACTGGAGGCTACAAAGATAACAGAAATAACAATATGTAAATAAGATAACAAAAATAAAAAACATTTCTCTGAAGAGACTAGAATAATACCTTTTACCCGATTAACAGGCTGAAAAAATTACCAAGAATTTTATATATAATTTAAAGGTAAATTTCCTCATACAGTGGATTGCTCAAGAGAAATTATACAATGGAAGGCATCTAACTAGTATTTTCATCATCATTGATGAGACTGTTAACAAGATGCTTTTCCTTTAAATAGCTAGACTGCCTTTTTATAAGAATGAAGTGTTGAACATCATGAAAATAACTATCAAATTGCATTGATTTTTTTTTAAATCTTTCTAGGAAATGGGATGAGTGGTGATACTGAAAATTCTCAAGTTGCATTTGTAAACACATCTTCAGGAAACTTTGAAAAGTATACTGTTAAGAAAAAATTAGGAAAACAAATTACCAGAGTTTTTCATCTCTTCAAATAACTGATGACACTGACATTATATAAGTGGGTCTATTTCTACAGCACTGTTAAGAAAAAGTTAACAAATTTCAAGAAGGAAAAAAACAAATGTGAAGAACTGAAGAAACAAAAACACATAGTATTGCAGTTTGTCACTGCAATCAACTAAAAAAAAAGAAAGAAAAAAAAGTATTTACAGAACAATAATAGCGAACCTTACCACTGCTAGTGTATCAGAAAAATAAGGAATATAAAAAACATTAAATTGATCCAAGTGAGGCTAGATTGTTCTTTTCCAAATTCTGCAGTAGTTTTAAATAGTGATCTCAAAAAAAAAAAAAATCAATATTCAGCATTGGTACGAAGAACTTGGCTCAACAAGAACAGACCTCAAGGCATTGCTCATATCCTAAATATTATTTAATCCTAAACATGCTCATCCATTTTTTCTTCATAAAATCTATAAGTATGAACATTACATCCTAAAAGGCACCTGGAAGAGCAACAACTGAGCAGCCTCATATACAAAAAATGCTATTACATATTTATGGAAATATAAGGGCTTAACACATGAGTAACGTAATGAGAGAAGTTAACACATTTCAAGCACAAATGTGACCAATTAACTTACAATATGCATTATGACAATAAATGTAGATATCTATGAAAAACACTAAACCTAACATACTTTTAGATGCAATACACTTTAGGCAACCCAATAACTGTATTGAAAAAATTGAATATCATGCATTACGGAAGAGCTTGCCAATCATATAATTCTAGTAATGGACCATTTTTATGTATCAAAAAGGCGTATAAAAACTGTTTGAAAGGAAAATGAGATTCAATGGACCCTTTCATAAGCCACTCTGACCTAGGAAATAAAACTTTTCTATAGAATATCATCTGGCAAAACTTATCACAACCCTCTCTATGCAGCATTGTTACC

>chr39:+:1431584-1433751

CAGAATCAAAATTTTTATTCTACAAAGTTTGACATGAAATTCATTGACAGTAATTATCATTAGCACACGTCTGTACAAAATAAAAGGTTTAAGGCATTTCAGGTCATTTGGGATAAAACATGGATTGTAATTCACACCCAAAATGGTGATCAAAGCCACATCTTATCTCAAGAGTTTAAAAGGGAAGTATTTTTTTTCTTTAAAAAAATCACATTAAAGAAATGAAACACCACTTTTCTTTATTATGTGAGCTATTGCAAAATATCCATGTTTCTACGGAGCACATTGCTAATTGCAGCGGTGTGTGTGTTGAAGGTGCCGTGATGGTCATTGTGAACGGAATGAGAAAGGGATTTTCATGCTGCCAGGGATGGCAAGCATCTTTTCCTTCATACAGAGTAATAAGCATAATTACGCATTCATTCATATCTCATTCAAAAAAATAAATGGGTTTTCTTCAAAAGACTACATCTATCATCCAAAAATGTGAATTAGTAACGTTAACATTTAACTGATAGCAATTTTGCTCAGCATTCCAAGATACAATACCAAGTTAGCAACAACAACAAAGACAATTTTTTTCCATTTCTGTGCCAATAGTTTTTAATGGCCTCTTCCCCAGGAAGCACAGCCATTTCCCTAATTACAGTATCTTTTTTTTTCTAAAAATACATCTGTAGCATTTACTAGTTATGAAAAGGATATCAATACTTAACAATCTTTTAGAGCGATGCCATAATAAATATATACACACATACATAAATAAAGCCAAGACAGGCAGCCCAAAAGTAGTTGTGGCAATGGACTAGATTACTTAAAGCATTCTCGTTACACATTCAAGCTTTTAGTTGGGAAATGTTAAGACAGTCACCAACACTACTCTCTATGTGTTCTTTCAAGAGCCCCTGGTTTTACATGGAAGCTCTGTTACACTTCTGGTAAACCACAACTCAAAATTTGGCCTGATCAGTCAATCACTACCAGTATATCAAAGCAATATGCATTGCAACTATTAATCTGAACAGTGATGAGCAAAAACATTACAAGCCAGAAATTTAAAAACAGAACATTTGATAAATTTCCAACAGCATTAGCAGATGCAGTGGTAATGAGTATATGTGTTGCAAGATAATAATGAAAGACAAAAGATTATCATTTATGAACCACTGACCTAGATAAACTTAAGTTTAATAAAAAAATGTACAGAGCCTGAAACAATTATAAGTGAATGCCATTAAAGTTGGTTCTATAATGAGTTGGACCTACAAATTCCACAAAGTTGAGCTGTCATTATCAAAATGTTATAAAGTAGTATAAAATTAGTTTCACAATATTTGTTTAAGGCTGTCAGGTTTTATCATTAAAATATTAAGTTATCATATGTATGGAGAACAATGATTAAATGCCTAATACTAATGTACCTATATCATGATATTTTGTGAGGTTTTATTACAGTTGTGTAACACTTATCACCTGTAATATTGTGGTATTTTTTTTTTCATTTTACAATAACTATGTTAGCTGAAGATGTACATCATGCTGAAAAAATTTCAAGATTATATAGGCCCTAAGACAAGGAATATAGTTTCCAGAGTTATAATACAGAAAACTGTATTAGTACAAACCTTAAGAGTGAAATTATATTGCTGGATTAATGACTATACAGTAGTTAAACTGATATCTTACTTTTTTTCTATAGCTATATTAAGTCTGTATTAAGTTGTTCCAAGTTTTTTTTTCTTTTTCTAAGGCACCAAAATTATGTAGCAAATGGTCTTCACTTCCATAAAAATTGTTTGAGACAAAAGTATCACTAGAAACTCACTTGAGTACCAGAAATTAATTCTTATACAAAAATATTAGATCTCTTTTTAAAGATAGACCAAAATTTTCCAGGAACTTTTAAGAAGATGAGCAAACAGTGACACTGTGTAGATGGAGACATTACTAAGCCCAGATGAGACTGAATAGTTGGATTAGAGTTGAAGTCAAAATAAAAACACAAGACTACTCATGAACTACAAAACAAGTTTCTTTATCAAATTACAGATTACAGAAAAAACTGTAAACTCCAACTGTTCAGCCAAAAGAGGCCCAGCAATGTTTTTCTGCCGTGGAAGGTCAGTCCATCAGGGTCCAAAAGTGCACATTTAAATATAAAGATGACC

>chr39:+:49026807-49030498

GGCTAAGTTTAAGCGGGACAAGTCTAAGATTACCCTTCTTTTTTGTGAGCCTTTCTTTGGCACGCTGAACAAGCGACCTTGAAATTTTAATCTCTTGACTCTCGCTATAGCTCCTTTTGAAGGAGGTCGTCTGCGTACTCTGTCAATTCCTTGGAAGGAAGTTGACGGAAAGGTCTGGATGGAGGTGGGTTCGTCAACCAGCTCCAACCCAGGCCTTTTGACACTATGCTCTGAGCCCATTGGCTGAAGTTCCACCGGTGGCGAAAGTGAAACAGCCTCCCTCCTACCTGAAGTTCTTCATTGGTTCTGGTAACCGCCTCGGCCTCCTCTGAAGTGCTTTCCCCTGTTAAAGGGGCCTCCCGATCCTCTCCCACGAAAGGAACGCTTACCTCTACCTCCCTTACCCGAGCGGTCATACTTCTGAGAAGCTTGACTCTCGAAGGCTTGATTGTAAGCTGGAGAGATGGCGTAAGAGGTGGAGGGCTGAGGCTGAGGGGATATCACTAAATTGGCTGTGATTGACCTTTAGAAGTGGAGGGTTGGGCTGTCTGCACTAACGGTACTGTTGGAACTTGTTGAGGAAAGCGTAGTTGCTTCTTCTGGTAAGGTTGGAAACGCCTGGGTTTCTTTTGTCCCTTACCCTTAGGAGTCAGGTCTTGCCTCCTCTTAGCCGAAAGGCCCCAACGATCCTTAAGGCTCTGGTTTAACCTGGTAGCCTCTGACTGGACCTCCTTAACCATAGCTTCTGGGAAGAGATCTGCTCCCCAGATGCTAGACGAGAGTAACCTATTCGGTTCATGCCGAATGGTTGCCTCTTGTAGGACATGCTTCCTACAATTCGTCCGAGCAGTGGCAAACTCAAACATATCTGACTGTACCGTTTGAGTCAGGGCTTTGGTCATGAGCTTAAAAATCGGTTCTGATCCATAAGCCATGGTAGCTACCTCAGACATAGCCATAGAATTGATGGATCTGGCTAGACGCGATTTTGCATCAAATTCAGCCTGAATAAGGCTATCTGGGAGCCTGGGTAGCTTTTCACCAAACTGCTCCATAGCACAGTCCGGTTTGAGTTTGCCAGCTGAGAATGTATTCGGCAAGTCTTCCCACAATTCTCCAATGAGGGGAGCAACGGAGATGTGGGATCCGCTTCCTTCAACTGCGGTATAGGTTCCCCCTTCTGGGCCGCTGGGATGGTCGACCTCGCGATCTTGGTTAGGAACGGGAGCGGGTACTCTCATCCATTGTAAAAATGGTAAAGGGACTCTTAAATGGTTGTATCTTGGTGTTAGAACAATCCATGTCCTCTAAACACCTGAGCCATTCTCTCTGAGCCTGGTCTCGTGAGTAGAGGACTGTCTCCTTAGGTACCTTATCGTCCCGCACCATAGCCGAAGGCGTAAGCCTTGCGTAGCCTATGAACGGAGGCTGGAGGTCTTCCGGGAAGAACTCGAAGTCCTCTATTCTTCGAGTCCCAAATTCCGGTATAGAGATGAGACCATCCTGGAAGGGGGCGTATGACGCTACTCTCCACGGATTGTTCATAGAGAACTGAGGTAGTGAGTCATACGGAGGAAGTTGGGATCCACTCGTGTTGGACAGTGGAGGAGAGGCTAGAGGAGCTTCTCTCAGCCCGGCTATAATGGAGTCCTGGGAAGTAATCCTGTCCGACAGACGAGAGATCATTTGCTCCATGCTACTCTTTAAGGACCCAACCAGGTCACCCACCTGTTGCAACAGGCCAGCATTGGAGTCCAAAGCCGGAGCTGCTGCGGAGGTGGAGGGGAGGCTCTGAATCGGAACCAAAGGTAGCGGAACTGGTGATTCTGCCGGAGTGCGAGATCTCTCTCTGGAGGATTTACTCCTCGAGGACTTAGAGCCGGAGCTAGAAGCTTTAGACCTGGATGCTCCGGGATTCCCAGCCGGAGACTTACGGGAGGAGGATGAAGCCGAAGTCGTCTTCTTAGACGACGACGACGTCTTAGTCAAGGTCATCACCTTAGACTTGACCTTAGGTCTAACCGAAGCACCAGGAGGAGAATATATTTTCGTCACCCGTAAAGCCTTGGAAGGATGGAGAGGTTGCAGGATAGAAGAAACAGTTACGCCCAAGGGTGACCCTTGGGTGTCCACCGTACCTACCTCGACCAACAGGTCGTCTATACCTACCATAGGTTCAACATTAATATCTAGGGTCGCGACATCCGTAGAGATATCCTGGTCCTGATCAGTTAATGAGGTAGCCAGCTGTTGTTGGATGAAGGCGATAGTCGGGTCCGCTTCTACCGGGTCGACGTATCCTGTCGCCTTGCCTCCGGGGAAGATTAGTAATGCCAACCGCTTCTCTAGGATGTAGGGCTGTCCCTTGGCGGCGTTTTTCCCCAAAACCGCCGACCCAGGCCCGCAGGGTAGCCAGTGCGGTATCTCTTACCGCCGGAGCCTGGAAGAGAGGGCGTAGTTTAGATTTAAGAATCACTTAAAACTAAAACTTAAAGTATAACTTAAATTAATTGCCTTAAGTTAATAAATGATGAAAACTTAAGCGCTAAAAAAGAAGCGGAGCAGCTACTGGAGATGGAAAACTTACCCCTTCCAAAAGCTGGCTCACCAGATCGTAACATATGGTACACGTCTCATGGTACCAGACCTGGATGTCCCCGAGCGGAGTCGCGCATGGAGCATGGGACCGGCAAACTTCGTGTCCACAAGGGTCCTGAAGTGTGGCGGAACATCCCGGATGCTCACAGTTGGTGGCCTGTAAGTGGGGAGACACATGAGTATCTTAAAAAACATCACTTACAGTCTAAAGGACAGAAGAACTCCGATGCATGCCGGAGCTCGGAAAAAATTTTGGGCATAACCCCTCCCTGCATCGCCTGAATAGGCTATAATCCCGGAGAGATCCGGTAAGATATGTAAGGGAGGGGGGATGGTTTAAGGTTCTTAAGATAAACTTAAAGATAACTTAAACCTAACACACAAGCAAACCGGACTAAGTCCAGTGCGTAGCGGAGTGTAGTAACTCAGCAAAACGGTAAGGTTAGTAGAGACCCACTGTATGCTCCGGTCCACCCTACTGGGTGGACTAACAACTTTCCGCTGGATACCAGGACTCCGATCAGGAAGGAGCCCTAGTAAGATGGGAAAGAGCGAGAAGACATACAGGCTCAAGCTAGCCCGGAGCGACAAGGTAGCGCGGAGGGTGGGCAAGGCCAGTACCCCCCACTCCGTACCAACCGGCCGGACCGAGAAGCCGGAGAGGTCGAGACCAGTCTGGGTCTGTCCCGACTCCCTAGCCCCCCCGCCTGAGGGGAGAGAGGGAGGCAGGCTCGGGTAGAGGAGCGAGCATGGGCAGACCGACCCACCCCCGCCCGACTCTATGGAAGAGCGGGAGGGGGGGGAAGAATGACTGGACAGGCGTCTGGCTGGCCCGTGATCACGAAGTGACCACGAGGCGGTAAGACCAAATACGACAACTAAGCCTAGGACAACCACTGATCAGGGAAATGCTATCGGGAAGCATACTGAACTGAAAAGCGGTGGCAAATAAGCCCACTGGGCCAAACCAACTGATCAGAGAGATGCTATAGGGAAGCAACCGAACTGATGAGCGGTAGTATAGGCTCAAAGAGCCAGGACCTAGGCTAAGCCAGACGCCTAACTAACCTAACAATAATAAAATACACAGTATA

>chr39:+:51278959-51281046

GCATTATGTGCTGAATTTAATACTCAATGACACACAAAAAATCAAGTGACTAAAAATTACTATCACAAAAATTTTCTACACAAACTGAGGTTCACAATCATACAAAAAACTTAGCTTATACAGTCATAGCTACATTTTAGTTTTGTTTTTTATCACATTTGTTTTTAAAAGTCATTTTATTGTGTGTGATTTCATGATATATAGACATAAATATGATATTTAAGGTAGGCACTGATAAACTGCATGTACTCATACAAAATGGACATGACAGGCGCATGGGAAGAAACAAAAGTATAATTATCGTCATCATAATGTGTTTGCTACAACAAGAATGGAGTTGAGATGTCTGTGAGGTATCTTTTCCTTTAAGAATTTGTGCAAAAAGGAAGTACAAATAGAAAAGAAAAACAACATACTCTTTCTCAAATTTTCTCATCTGAAATATACATGGAGAAAATTACAGAGGACTTATAAGAAAAATGAAATAGAAATTCTTGAATCCAGCTCACCACCTGCTCTACCAAACACATTTGAGAAAATATATAAATTAAATAACTTCATTTTTTCCATGTGATGGCAGCTTAGGCCAATAGTGAGAGTAGCCTAAAAGAATACCAAAGTTTGTCCGCTCAATACTGCTCATGGTACAGTAAAAAAAAAAATTAAATAAAAAAAAAAATAAATCATAACAAAAACCATCTCTTGAGCTTTGCCAGTTCACATACCCGATACCTCCTGGATTTGTATTATTAATCTTCCGTTACCATGTTGTGCTGCAAAACTGCAGCAGAATCCAGCAATAATATAATCTTGCATCAAAAATTATCATAAGTAATCATAAATATGGTATGAAAAAAAAAGGTATTCTCATTTTAACTTTAAAGCTATGTTGTTATATTGCAGCCTGATTGTAGCCAGTGTTATCTTCAATTTCCCAATGGCAACATCTCCAATAGGATTCTCTAGGTTAAACACTATAAAACAATGAAAATTTCAATATCCAATGAATGAGAACACACTTTACTGCACCAATGCTCATTTGAGATAAGTGAACTTAAAAAATACAAGAAATGGGGTTTCCTTTGAAAGGCAACATAAGGCTACAATGCCGTGAAAAGAAAATGCATTTTTTGACCTATTACTCCAGAAAAAGCATACATGGCAATACTTTCCACCACTCCTTTCTTCCAAATGACTAGGATGATGGTTCACAATTAAAAAAAAATGAAGGGTCCTCCCCCAAATTCTAAGCCCATTAACAACACTAAATTTCCTCCACACTGTAGAAAAGGTAAAACAATGATGAGAAAACTCATAACTAACGAGATCAATTAATGGACTTCACAATGCTGAGAAATAAGGAAAAGAATGATGAAGGAAGCCTTCCACACGAATCATTTGTCACGCCTTGATGTGGTGGTGCCCGCAATCTCCTAGTTCTCCAGTATGCCCAATCTTGTTAACTTCACTCTCTTGCTAAAACTGAGACCAGCTGATTCAAGCAAAACCTGCTTGTTCATGGAAAATATAAAGTTTTGTTTTATTATCACAGTTCACTTTCCACAGTCACTTCACCAAAGTCTTTAAGTCTGAAAATCATAAGGCTATTACAACGAAAGCACTTATAGTAAGAACACTGTCAAATTTCATCACTTTGGGAAGCATAAACATTCTTCAAACTTTTAATATTTGTCTCGGAAGCAGACAATACATACTGACATGTACAGAGTTCAGCGTTTTGTCCACAGGTTAAACATAAACTGCAATAAACAAGTAGCAATGATGAGAAGGGCCGTTAACATTGGAGAACTTTTGAAAATGCTACCAGGCACATGAATGCTTCTCCAGGAATGAATATAAATCACATGTCTGCTCATAAATGCTTGCCAAATTATGTCTAAAACTTACTTTTCCATACCCTTTCACTTCACCTTTATATTTAGGGGACTGCACCAAATAAAAAGAACGATAAATAAAGATAAAAAAATATCACAATGTGAGGCCCCAAAAAACTGGTGGGTTGAGCATAATTTTATCCCCCTTTTATTTTAGGAAAGGTGGAAGCTATGCTGTCCTTCTCTGGCAGCA

>chr38:+:18034156-18036906

TATAGGAAATAAGTTATGTTTAGTGATGTCCTCCAGAAGTGCATCACACACGCATTTAGGATGATGTCTTTTACTCGGAGGTTTATCTTACACCTCTGTCCTCTCCATACATTAGTGATTACAACCAATTGTACCGTCCAATTCTGCCTTCATCAGTGAAATATTTCCCGTCTTGATCTTGTTACAGTATGTATTTATTGATGATCAATTTTCATGTAAATAGTTGGAAGTTATTGTATATCGTAATTAAAAAAGTTACACTAGAAAGGCATTAATTTTTTATTTCTTAATTGACAGAGGCAGCTGTTCTCAAAGTGAATGTTAATTACTACCCCATACAGTGTGGGGAGTCTTTTGATCCTTGTTTGAATGCACTCAGTGCTCCTTTTTATTTGCGACACAGTGGAGCATTCTGTTGTTTGCATTTTATTTGTTGAATTATTTTCATTATGTTGGCTGTTATGCTCCATTACATCTGTGGCTACCCTAGTTCCTGTTGAGTGATCAATATGAAGCAAAGGTTGAGCTTTGGCCTCTAAGGCTTAGTTGTTAACTCTGTGCATATATAAAGAGGCCACAGTACACTTGTTTCATAGTTCATCTTCCATTATTGTGATAGCAAATGCACAGTCGTTACTGTAGGATGATAATTTGTAAACCATAAATATGCATGTAATATATACAGTTAAGTTTGAAATAACTGTGTTCATTTGCAAAGGATTATTAAATATAATACATAATACAATACGTATAGCCATTGTTTATAAGTCTGTAAGAGTACAGTACAATGAGAATTACTTAGGGCTGAAAGTCTAAACTCATAGTGCAGTACAGTACTGCAGTTTTATAAATTCTAAATCAAACAAGATGATGGATAAGGTGGACGGCTTTAAGTGTGATTTTAAAATTTGATTTTATTTCATTTTTATTTTATGTCAAAATGTATTCTGGAAAAAATAGGTATTCACTATAAGATTATTATGTGCCTTACAAATGAAAAGTGTCTTAAAATATGGAAGGCAATTCTAAGTAACAGTACCAGGAACCAAGGTGATAGCCAGTGAGTTATCTAGCCTTAGTGAACAATGAATGTACTTAAATAACAACATTAAAGCTAGGTAAATGAGTCCTTTAAGCAACAATACTTCACCAAACTTGATGTACCTGTGGGTGATTTTTCAATGTAATTAGCAGGTGCATGGATTCGTCCCCTTGCTATGGATGCCAGCTGGTACAAAGGATAAACAAAAGATGTTTTAATGTGGAAATGATTTCCTCGGGGACACGCTCTGTATCCTGAGAGATACCCTGGTCATATCAACAGTATTTCAGATCCCCACATCCATCTACAGTCTGCTCAAGTGATTTATTGGATGTTCCAAGTGCCTTTGTCTGCAGCTGTTTAGTGACCATCTCTTGCTTCTTCCTGGGCAGTATGGCTTGTCAAAGGTCTTGAGCCCAGGTTGTATGAGCATTAGCTAGGCCATTGTTGCCTGACCTAGGTCTTTAAAAAGAGGTCACTTTGGCAGCTCCCAGGCCACAAGTTGAATGTGGCAATAGGTTTAAGATTCATGTTAAATATTAAGTGAACAAACACTGAGAGTGCATTTTCACACTTAGTGTTTGTTCTTATATTATCAATAATAATGTTATTATTACTACTATTGCTCCAGTAGACATGAATTTTTATTATATCAGGAAATGTACATGTTAATTTTTTTATGTTAATAGTATGGTGTCTTTTGTGATATACTACATCTAGCATGCAATGTTTTTTAGTTTGTCTTATAGTGGCCATATTTGTTAGTTTTACATTGTGTAGTTAGACTCAGCAGCAAGGCTCTTTGTTTTTTCAATCTTTTTAATGTGGTGAAGGGTTCCTAGTGTAGAATAATTAAAATATGGATTTCCAATATTCATAAAGTTTCGTAGAGGAAGCATTCAGTGGTAAAGTTTTATACTAATAGATTTAAGAAATGCTCCCAAGATTAATACTTGAGAGTTGTCTTGGGCTGTCCTTCCCAATCTTTCTTGCACAAGTTTATGAAGTTCAGTTAACCCTACATACCTCAGAATAGTAATGCTATTATATTAGTACAATGGAAAAAGTAAGGAAAAAAGTTCATCATTTCTACAAAAAATTACTGCTATAGTTTATGATAATGTTGCATTGCTGTTTTTTTGAGGTCTTGTCTTTCCATGATCCAGGGGTTAGTAATTTCATAGACTATAATCAATGTTGCCTTGCTTTATAAGGTTCTATCTCAGTCCTAACAATATTTTTTCACTGTATAGTTTATGTGGGACTGGAATGGGAGTAACCCATGAAATTGGATAGAAGCTAAGCAGTGTCTTTTGAAATATGTGAGATTGGGTTACCATTGCTAGCTGGTCACCCTTTCTTTGTCATGTCGTATCAGCACACCATGTACATCTATCCTCTTGCTAGAAATGGTTTAACCTAATCCTCAGAAATGGACATTGCTTGCTTCAATCCTGAACTATGGTATTAGCTTATAGCTCACATTGAATGGTTCACTGCCACTTGCAGCAAAAGGGGGAGCCGATGATTTCTCTTGACAGTACTCATAATCCCTTAACAATGATTAGTTTCTTTTCACCAATAAACTGCTCTCACTTTTGCTGTATTTGCATGTGTTCCTTTAGTGCTTTTATGCAGATGGGGAATGAAGAACCCCATGAAATTAAGTGTATGCTGAAGCAAATGTAAATAAATTTTATCTTTAAATG

>chr6:+:101152465-101156080

ATAATGAAGTATTTTATTATGAAACTGATAGTTACAATACTCTAAATATACAATACCATAGTTATAACCTAAATACAAGCACACAGAGTGGACATTGTGCTTAAGCTATTGGGTTTAAAATTGGTGAAACATACTACAAGAATGTATAACTTGGTATATTATTTAAACTTTCATTCCTTCACATTCAGCACACTCAGCCTTTAAGGAATTTCATTGAGTGAAGCACAGATAAGTTATAAAAGAAAATGTGATTCCTGACAGTGATATTCACGGGTGGAGTAGAATTTACTTTTTATGTCAGCAGAAGCACTAATGCTCTTGCCTGCACAGCACAATTGAAACAACCTCTCTAATCCTGAAATGGCAATCTAGGAGTTAATGAATAAGACTTATACATTAATGTCAATAGTACTCTGCTTGTAAAATTAATCTCCATGGTTAACCTGACATTGCACACAAATTGACACAATTGACAAAGAACCAGATAAAGAGTAATCATGCCTAATAGCTAGGCCAAAAACTGTCAAAAAGACAACCTCAGGAGTTAAATAAAATAGTCACTGGTGTCCAATACTGTGCCCACAAAGCATATTTACAATATCTACATCATGTTAGATGCTTAGTTTTAAGTTTTCTTCTCTTGAAAATTCAATATACTATATTGGGATGAAAAGACTACTTTTAATGAGTAAACTCTGCAAACACCTTGTGACAGGTAAAAGTAAATAGACTGTTCAATAGGTATAAGAATAAAGAGAAGTAAACTAATGTGACACATAAGAAAACTGTATACTCATGGAATGAAAGAGAGAAATGTACTTTTTCAAACACTGCTGTTCAAAAGATTCTTGCAGAAACTTAAGATGCAAAATGGTGACTTACCTTATACTGTAATAGATAAATTCCATCAATAAAATAATTTTAAAGTCTGCATTATTTACTACAGTTTTTCTAAATATTTTAGACCAGATTCTTAGCAACAAAGAAAAATAAAAATTCTCTCCTTTTAGGAGTACCAAGTGCAGTACAGGCAGAGTTGCCAACAGGACGGATCTCTGGACTGGTAATATTATTCTTCAAAGGTGTATTTTATATACTGAGTCATTATATACTTTTAATTATGAAATCTATGGATCATGGTTTATGTTCACCAATACAAATCCTGGAGAGATCAGTTGCTTAAAAAGTTCCCAATACAGTTGTGTCATAAAATAAACCTTTGAGGGAAAGACTAGGTCAAGGAATATTGTATTTAAACACCAAAACATCTACTACTGATGAGTTTGAAACAGTGCCACAATCTATTAACAAGAGTGACAGTGCAAAAACAACAGTCTATGCTGCTGATTACTAAATGGTAAAAGAAGAAAACACCCATATAACGATTTGAATGGCATCAAAACTGCAGTTTGTATAGAGTTAATCCCAAATTGAAATGTATAGTAATACTGGTAATATCCCATTCCTTTGAAGTCAGTATTATACGACATGAAATTTTAAGACCCCAACCACAACCTAGCCACCTCTAAAGTTTATTGATAATAATAATGAGTTACCGGCTGAGTTTCCTTACACCAAACTTAAATACAACATTCATATCAGGCAAAAGCTCAGTCATGCATGTATCCATTCACTACATTTAAAAACATAGACACACATTTTCTGATAAGTAGACATAAAACTCCTAAGTAACTGACAAAGAGGACCTGTAAGAAATATAATAACAAACAAGGAGTACCTTCTCTTCTTACAATAAATTAACATCATTATACATAAAAATAATGAGAGTGAAGTCTTGAGCTTCAAGACAAGCAAGCTAACAAACATACTGTATATCACAGTTTCAACTTAATTCTCATTTAAATTTATATAAAAGAATCACAATCCAGGGAAATTTTATGATGAAGTAAATTTGGTAAGATTTATAAATTAATTTTATATGTGCTGTACACCTCAAAGGTAGTGGGGAACTTAAAGCGATTTAGGATATAAGTAATCACTTGTAGAACAAAGATTATTTTAATGTACACCAACATATGGTATTCATTTGTGTTTTGTTCCTAAGGAGAAACAACCTGTTTTAAGATAATTTTCTTGGTGAAAAGTATCAAATACTTTGAAAATTATCCATAATCATACTCTCCCAAAAATAATTCTGAAATTTATAAAATGTGCAGTTACTTCCAACTTGTTAACTACTTATTAGCAGTTCATAAACTTGCAGAAAATGTGCTGAGATTTAAAAAAAACTTAAAAAGATTTGAGTAATATGTAATGAATATATATATGTATGGTAGTACTGTCCAACCATAGGTTATCCAATGAATAAAACAAACACTGCACTGTTAAATTCTAAATTTACATACATTAGTAATGAATAATGAAATTATAAACACTAATAAAAATACAGGATAAACTCTTCGGGTCTTTGGATTTGAGTTACAGCATTATTCAGAATTAAGAACATTAACTTTGAACTATTTATGAACAAGTTGTCAGTTGAAACTTACAAGCCGTATAAAATGACACGGACTTATTTTACAACATTCAATTATTCTTTACTGAATATGTGTCTTACAATTTATACATAAATGACAGCACTGGTAGAAAAGCACACAGACAAAATGAAATTCACTAAAATTCTAGTTGCTAATTACTACCAACTAGCCAAATTAATTCTTATTCATATATAAAGGACTAGAATGGGTAATTTTTTGCCATTGCTGTGACCATCAAACATTTGAAAGTTATATATATTACAAAGGTCCCAGTTTTAATGATTCAGGGAATCTAAGGTAACTGTTTGTCATAACTAGGTTCTGCAGAGTGAAGTCACATGATACAAATACTATATGCTAGGCCTTTCCCCACATCGTCATTATTTTTTCTACACTTAGTGCAGCAGATACAGAAAATGTTAAAACTTGGCACTGTGAATGATACTGTTCGGTCCTGTGTCACAATTTTCTGCAGGAAAGTGACAGCTTAATCAACATTAATCACTATTGATTTGACTTAAATAATTATGCAGAGATTTATGATGAAACTGACAATAGACTTCCTGGTTATAAGATGTAAATGTCACAAGGAGTTAATTGTATAATTACATAAAAAATCCTTCAAGATTTGTCTATTGCTAAGAAACTCAAAAGCACTGCATAATCAAAAAAAGACCAAATCTTAATTAAAACAAGCCCTTGTTTAATTGTTATTGTGCGATAAATTTTTAACCCTAGAAACTGAAGAAGCATTCAGCATTTCTTGGTATAGCTCTTATAAAAATTAACACATACCCTACAACAAAACTATACAGCATTTAATGAAAAGTTAAACAAGCCTTCAGCTTTTATACCTGCGAGTGTAGCAGTGTATGCTTCTGTAAATTTATAATTCAGTATAATATATATTTCTTTAGTATAATAGGTATAACACTTTGGGACATAACTCCAATGTAGTTCAGGTAAGGATGTCTGTGACATTAAGTTTGTAAATATTAATTATAGTTACTAATTGCAAAACCTTTCATATTTTAAATTAAAATCTTAATTTATTAAAATATCTTTTCTCATTCTAGTATAAAATGGTTATGGGGTAATATTTTTTCCATGGTTAAAAC

>chr6:+:107801696-107805992

AATGACTAGTATATTAATAAACAGTTTAATACAATAAAATACATTTTCTAACAAAATCTTTAGGAAAATTATAATTTCAGGATGCTTACATCAAAAATTAAAATTACTGACACAAAATTCAGCAAATATAACTCTTAACATTACTGCAAAGCTAGGTATAAAAGGAAAAATAAAACTCTACTAAATACAAGGTTACTAAAAGCTATTACTGCCTTGTCTGCTTCATATTGTGACAACAATGCATGAAAAAATATCATTACAATGTATTACTTTTAAAAGTAAATTGTATTTTTTCTAGGTTTACAAAGCAAAGCCTTTTATATAGGAGCATCCTTCAGTGTGAGCTGGAATTGGCCACTGAAAACTTGGTAACAAGGCAATTAATAGGTGCAGATGAGGGTGACTGGGGGAGTATCCTTTACTTGCTGTCATGCTCACTTTTCTTTTGGCCCCTGGGGACTAAACAGGGCGGTTGGGTGATTAAAGTACAGTATATCAACTCCCTTGTTTGAATACCTATGAAAAATAAAAATATAAAAAAAGCCAGGGCTGCCTCACTGAAATACTCAGCCCATGAATGAGTGAAATAATCTCCTTGAAGAGCTGATTCAACTTTGCCTGTTGGGATTCTAGATGAACAGAACTACAGAGTCCCATCAGATCCTTCCCCAAGAGGGGAGAGTTCATGGAAGTTGTATGCTTCTTCTTGGGTCTGGCGATGAGGAAAGGAAGACAGTCCAACAATAACAAAGAAGTGCTGAAAGTGCTTCTTCTCATCCTCTCGACAGTCCTATTCTCGTTACACACTGAAGGGCTGGTTGAAGCTGCAATTGAAGGTGAAGTCACACCTACCAGACGATCTGCTACAAGAAGAATTACTGCACCAATAAGATTCCCCTTCTTTGCAGCAGTTGCCAAAGCCAGTTCAGCTGTGAACACATCAGCAACTACCTTAGATGCAGAAGGCTCAGATGTTGGCTTCCTCCTCAATAAGGTTATCAAGGAAGGCTGCAAAAGGTCAACCTGGAATATTCAAATAGGCAAAATCCTTCTAAGGTACTGCAATGATGTTGATACCTTATGTGTACTTGGCGTGTAAGAGGGCAAGACAGCTGCTAAAAGAGCACCTCCCTCAGATGTTTCATTCTCTGGGGAAATATCATGATCAGATTTAATAGGCCTATCCCTCCTGGAAGGGGTAGCACTTACAGGAGGTGGACGAGGAATGGAAGACAGATGAGGGAGGAACTTCCAGGATTCAACACCATCACTGAGGTGTATCCCTCCACACACCTCCTCCCTTTAACTAAGTCAAGTGAATGTTTCTTATTCTTCCTCCTTCTTTTCCCAAACTTAAGGCACCGAGGAAAATCCACACACCACATTCCCTAAAAAATTCCATGTACAGAACTTCCTCCTTCAAGCAGTACAGTAAATGAGGGTTAATCAATACAGGTGGCATAAACTGCCTACAAGGACAACCATGTCCCTCGCAACAAAGTTGCTCATCTTTTTCCCCATACAAATTAAAAAAACCAACAGCAAGGCAACAATAAAAGAAAGGAAAAAACACACACAATTAAAAAACAATGATCAAGTGCTCATCCAAGTTCTCATAAAACAAGCAGGGACAGAAGAGTGCAGCCTGTCTGTACTCTACAATTGCTGAAAGAAAAGTGAGTGCAATGCTTAACGAGTGATGGATGTTCACCTATTTTCACCCCTCTACACCTATAAAGACCTTGTTACCAAGTTTTCAGTTACTAATTTCAGCTTGCGCTTGAGGATACTCCTATATAAAAGCCAAAGGCTTGTATTTTTGTAAGAACAAATAGACTGATTCCTCTTGATAATTATGCCTTAGCAACAAAATATTTCCATAAAATCTGGTACTTTGTAAGACAATACTTCTTCAACACAACTCTAACACACATGTATTTGGGGAAAACCAAATGTTCTTACATTACTCATAATACATTTCAGAAATGAAAAGAAAAACATGCTTTGCCAAATGCCATCAAAACTAGTTTATAATTCTTAATTCTGTGTGATAACTTTGCAGCTAGTCTTAATTACCAATAATCAATACTGTGAAGATGAACTAATTCATAAATACACAAGAGCAATCATAAACATGATTTTCACAAATGTAAACAGTGTTCATGTACAATATAAATATAACTCTTATGCTAATCAGGAAGCGAGGTTTGAGTAATGCTTTTGAAATCATGGCAGCTCTGCCTTAGCAAAAGAAGCTTGTTTTCGGCTGGAATTTAACAAAGCACTTTCACTTCTGCAAGAAGTAAAGTCATTACAAAATAGACTTATGTCACCTCTTATTTGTACCAATGTCATGACTAGGCACAGATAAAAAAAAGATGAGAAAAGCTGCTTAAACAGAAGTATTGACATTTCTCTGTGAAATGGTTACTAGTATTCTGCTTTTAATCCAGATGTGTTGAAATGTAATTTACAAAAATTTGGGTATCTTTATCTTATTTTAAAACATACAGTAACATGCCATAATACAGTAAAGTGCTTGCAAAGAAATATTGGTACAAAACTAATATATCTAAGTCTTTGGAATATACAAAATAGTATCTTACACGTAATAATTATACAATATACTTCTACAAAGGTTTAAGAAATTGCGAAGCAGCTACACTTGAAGCATTCCTATTGGCATTATATTGGTTCAAGCATTATTCTATTTTGAGAGGACAGCATGTAAACAAAATTAATAACTACTTTGTTTTATATTATAAAACTAATCACAACATTAATCTGTGTAAACACCTTTAATCTTTAAACTGATTTTTACACAAAAATATAAAGCAAAACTGGTTTTAATCAAACAATAATGATATAAAAAGTAGAAACAGCACGCAGAAATTAAACAACAAAAAATAAGGAAAATGAAAAATGCATGCTGTACTGTGCATTGAGACTAAAAGTTAAGAAATGCATGATTTGTGTAGCTCTCTTTCTCTCTCAAGGGTGACAGACTACTTAAATATAACATTAAGTATGGACAATTATTGTACACAAGACATACAGTGACATTACTCTAACATAACAGATGTACATCAACAATAAGCTGGTTATCTAAGTAATTTGTCATATATGATAGAGGCCTACCAGTCAAGAAACCTCTTCGAAATATCAAAATCTCTGGTTATGAAGACAATTACTGTGTATGAAGTCTATGATACAAAATTTCCTACAGTTCAGTAGCCACATCACACTGCAGTAAAGGAAGGCTTACCAATGTGTGTTTTATATACAGAGGATAACTGATGAAACTTAATCAACATTTCTTCCATTGCCAAAAATTACAGGATAATCCTGCAAAAATGCACATGCAATAAAATTACCACATACTTAAGGATATTTAATCTACCCATAATAGAGTATAAAAAGGTTATGGAAATTCTCCAAATTTATCTGATCTTACTTTCACGAAAAGAAAATGAAATAGATATTTCAAGTTCCTGGATACTCCATATATTCAAGTCACTGACTTGTAAGGCAATTCAAATATATCTACTGACCTTCACTTTAACATTACAAAATTTTTAAACAGATTTCCTAAACCAACTATAATACACAAAGTAAAGTACAGTACAATGGTCAGTTTATAGCAATATCCACAAACTGTTACATGCACAGAAATAATCCATCTTTATATTAATTCTCCTGTTACATAAATTGCTAAACCTTTCTTGGCTTACATGTATCTTTCAATCATCCCTTCAGTAACAAAAGCCATTATGAAGAAAATAAACTTTATTACATACCAAAAGTTCAAAAAGCTAACATTCTAGTGCTAAGTTTGAAAATATAAAATGTAAAATGAAACACTTTACCTCTGCTAACAAGAATTCTGTTTTCAAATCTAGGTGTGGTATAACAGGTGACAAATTATTCTTGCAAAACTCCTGGGATGAATCGCAACTTTTCAGGGACTTGAGCTATACTAAAGAAGAGTACACAGAGACCAAAGAAATGAAATGGTTTTCTGTTAGAAAATTATTGGCAAACTTTAATAACAACAAGCAAGTTCTACATAAGATGTTTAAATTGTCGGTGGATGTGCCAACAAATTTCAAGATTTACGAGTACAACAACACTTAAAAAAATATCTGGGTTTAATTAGAAAAATAAAGCTAATGCTTCATCATCCCAAGTGTTAAATCAATAAAATACTAAAATGCTGCATGTCTGCAGAATATAATAAATGGCTGTGACATCATGTTCAAACATACCTTGTCACTGATTGTGTTATACTCGTATCTCC

>chr6:+:113285147-113288214

CTACTGTAACACATTTATTACAGAGACTTGGTTACATCAGCATTAGTATTATTGTGCGCCACCAAGCTCACACAGATAGCACCGTCCTCACAGCATCACTCCACGAGATATATTACATGAGATATTGTGATGCAACTTTCCAATTGTTGGTACATTAGGCAAGTGGTTCCCAGCCTCTCTTAGATTTCAGTACTGCTCAAGAGTACATTTATTTAGTGCTATTTCATGTACTGTGTACCACCTGTGTGCTATTCTTTAACCTTGTCAGCTAATATCAAGTCTCCTTTGGAAGGTTGCTATGCATCACTAGTGATACTAGGACGTGGCATACATTTAGCCAAAGGTTGGGAACCACTTCAACAGACAGTCAAAATTCTATACAATGCTCAGGTGTTCTTTAAATCTCGTTGAGTCTCTGACTAATGTCCAAAATTGTTGGGCTTCACCACAATGGATGTTTATCGCCTGAATAACCGTATTACATGATTCACAGGTCACTATCCTACCAGTAAATGAGCCAAGACTACTGATAAACACTGTATTAACAATTTTAAATTTAAATCTAATCTTTCAGGTGGATAAAATACTTTGGATTTTTTCAGACATAAACTCTTTGCTATATAAACAACAGATTTTCCAGCTACTTTAATTTCAGAAAATGTAAACAAAATGTAAACAACAGTTTAACTTGGAAAGATACCAGATCACATCTATGACCATATCATCAAGAATACTCAGTTCTATAAAAGACAGACATTAACTCTAGTTCACTACTAATGACAGCAATCCTTTCAAAACCCAAGAATAAACAAACGAAAGTTGTGGTGAATCTATAACTGTTCTGTACCTCTATAACACACTATTGTCAAATTCAGATTTCCTAACACAAGGCATGTCATTGAAAACTTAAGATACTATGCATGAAACTGAAGACACAATGATAAATGAATGAACTGACATTAATAAAAATGAAAAATAGAGAGATATATGGAGACAATAGGAAACACTCCATTACTGGCAGGATTGTAATAATAATGACAATAATAGTAATGATAATTTAATTCACCTATTAGCCTAACAATTCAAACTGCCTTGTTGTGAAAAAAATAAAAACAAAGGAAGAGAATCGCACAGCGACAGAGCGGTGTTCAATAATCTTTACCACCCATCACAAAATGTATGATAAGTGATAACATTTTGTTATTAAGCAAGCTAGGTTTATTTCATTATATACATAAATATAAATCAGCTTATAAATGATAAAAGAACAAAGACTCATTTGTTCTGAATGGCACCGTTTCAAAGTCTTTTCAGAATGTTTGCAAATATTTTCCACATCAGTAGTAAATTTCACATCCTATACAGCTTTGCAAATTTTCAAGCTTTAAGGATGACAGATGACCAAACATATGCTGCTTCTTTTTGGACAAAGTAGCATTTACAAACCTTCCTGAAAGCACTCTGAGGTGGCCCATTCAACTCTAATCTAAGCATAATTGTCAAGAATATATCTATGCAAGAATATACTACAATATTAAGTTAGGGTAACGCTATTAAGGCAGCAGCATTGATGTTCGGTTACCTGACACCTCCACCTCCTGAAAAATGGCACTTTCTATTAGCCAGGAAATCGAACACTGCCAATGCAAGGTCACTTTACTACAAATCTACAGTCCAGCCCAAAGTTAGGTCATAGTTTTATAAGAAATAAAATTAAGGCAACAAAATAAAATACACTGGATTGGAGCAACATAAAAATATAATGTAAAGAAAATACAATCTGCTAAATTCCAGCAACAGCATGGGGCTAACTCTGAAACACCTGGTAGACACTCATTTTTGGAGTGTCTAACAGATGGCGTCATATTAGTACGGTGCTTTTTTCACGCATTTTTTTCCACGTTAGGCAATGATGGAGAAAACAGTGTCCTCGAAATCACAGTTTTGCACAGCTGTGATGTTGGCTCAACCATCATCTTGCGACATTAACTCAGAATGGAAGAGCCTCTACTTGGTAGAACTGAAGGTGGCAATTAATATTCAGTAAAATCTTATTCCAGTGCAACTGAAGGAAACAAAGAGACATCTTGTTTTCACATCCAAATAAATAAATAGATCAACTTTGCTTCTGATGTACTTATTTGCAAAACTAAGTGTGAGAAGGTCATAACAATGGCAGTGAAATCCATCAAAATATACATGAATATAGAAAGACACTATGATCAAAGGCATTGAAATTTGATATCAATTTATTGGACTGGATTTGTTTTTCTCTTGGCTGACAGCTCCTTTGAACAAACATATTTTTTGGCAATATAAAAAAACTTGTGCATCACCAATGACTCATATATATATTCTTCAGTTTTGTGATCAATCAACTAAGAAGTGTTAAAATTTCATGCTGAAATTGAAACAATTTACACATTTTCAATACATTACAAGAGGACCAAAAAGTCCCATATATTATCAAATAATGAGCTAGATTTTACGTTACCAACATTTGCAAGTTACGTCAATGAGATAACAAATGTCTGTCATAAAATTAGGGAAATAATGACTTTGCACCACGCATACATCATCTACTAGAGTCAAACAGCATACAATTCAAGATGTGCAAGTGGAATTTTCAATGCGTTCAATTTTTTAAAGATTTTAATTGTGGATGTAAACACAGATCCATAGAGAGAAAAACATGGAAACAGTATATAACGTACCTTTACTTTTTTTTAGAATTTTCCTAAAACCTACAAAAAAGTGTTTCAAGTCTCAAAGATCAATAAAACTACAGGCACTTATTTGCAGAGACTGAATATCAGAAGGTAGATTGTCTCCATGCTTACTTTTTGACGAGTAAATAAAAGATGTGACTATAAATACAATCTTGTTACCATCAACGTTGAATTGGAAATAAATTCAAACATATTGTATGGATCCATATGAGAAATTTGTTTATTTGTGTGATGATTAGTAGTTGCAGTAGGTTCTGGTGGCAGCGGTGGGATTGGGGCCATTTACATGAGCTTTTGCTTGGCGCAGTCGGGGCAGAGGATGTCTGCCCCGTCGGTAAT

>chr40:+:37851841-37853749

CTAAAAGGTTACATATATTGAGGAACATAAATACGTTTGTATACATAACTGTGGTGTATGTGCCTATAATAAGAAATGCCAAATATGTCTACAACACCAATGCTTTAAGCTTTAGGGTCCCAAGTGTACAAAAAGCTAGGGTACAAACATTATCTCATACACTCAAGGGTCTAGAGCAAGACTTCCAGGATTCCTCTAGCCATCACTAGCAATTAAGAGCAATTTTTATTTTTACAATTTGGACTATGCGCAAGGCTACAAAGTTCATTGTATTAATGGCTTACTTCTATTTAGCACTTCTTACAAAACTGGTTTGTTTTGGTATCACAGCACATTAAGACCGAAAATACTGTGCAAGCTCCTTTTTTTTGTATAAAATTCTTCTTTACAAAGACCATATAATTCACTATCAGCACTTCCAAAACTTTCTTTCAAAAGTCCTTGTGCCTTACTTGGAATTTTGATTATACGGCACCAGGTCCTCTATAATTTTGGTGTCGTATATGTCTTGGGGTCTTACAAACTGCCAAGCATAGTCTACATGAGGTCCTTGTATGAATGAATTTCTCGAGTCGTGGACACCTCAAAAGGTTACTCTGACCCAATTCACAGTTGTGCTTGGTCTTCCTGACTTCGGTAACATGTTTTCTACTTCTACTTCAGACCTATTTTCAAAATGATTTTCAGCCCTACAATTCAGTTTCATCAATGCTTGTACAGGGTAGAAGAGATGACTATTTCCAATAACAGTTCCAAGCCAGGTGGTGTTTCAGGTCAAAAACCTCAGATAGGTTATGGACTTGGCCATGAGCATCCTATCACAAAAAGCGGTGGGAGTCTGTCTTCCCTCTGGTATCAAAGAGAACCTATAGATTGAGTATTTTCATTATTACAAAATAACTTCCTGTGTAAAAACTATCAAAATGTTCTAGATGCCTTATTAAAATCTAGGAATGTACAAACTAATAGTACAATAAGTGTTTAAAAAAGAAGAAATAAAAATTTCATTGTCATGGAACATTACAGGTATAGCTAACTCATTTCCCATGATAATACTGTTATTAAAACTCATTCAACTCAGAATAATCCTAAAAGCTATCTTCATGCCACACAGAAAATTTGACTACCAGTTCAAACCTTCAACTTGTCTGGCAATATCTAAGGTTTATACAGTTCTGTATATAAATCTGGTCATGTTCATATAAGAAAATGAGCAGTTTGGTTAAAACTACTAAAACATCTGCTGAGACCAAAAAAATCTACAAATGAGTGGCTGGTAACAGTTCAAACAGCAAGGTAAAGGTGGTCTCAGTAAAGCTAATTTTTATTAATTGCTGCTCAATTCAACTGAACAACTGCAAATGACACACCCTTCCATTTAGTCTTATGTCCTTTACTGTTTGAACTCTCCCATACATGAAGGTAAGAACGAGCTCCTTTCTGATAGTGGCCATCGAAATGTTAACGACTATGTCGGCACAGGGAGGTGCTGTGTTCCACGCTAACAAGATCTATACAAAGTGTATCAAGCAAAAAAGCTTTCAAACAACATTTATCACATATATTACAAATAGATTTGGTTCGACTGCGCATTCAACAACGTACTTAAGTTTAATATCTCTACTTTTTCTCTTCTTCTTTTTCTTTTTCTTCTGTCTTGTTGTCTTTCTTGTTTTCTTTTTCTCCTTCCTTCTCATCCTTTTGAGCCTTCTCTTTTTCAGAATCATCCTTTTCTTTTTCATCCTTCTTGTCTTCCTCAGTCTTTTCAGTCTTCTCAGTTTTTTCCTCTTTTTCTTCAGCAGGTTCTTCCTCCTTGGGTGGTGGTTTTGGTAGATGAGAATTCAAGTCAAGATTGGAGCACATTTCATCCCAATCTGGGAAAGCACGCTCTTTCACCCTGTTGCAGAAC

>chr40:+:43482912-43484897

AACATATCAGAAGTCATAACTCTTAGGTTTCTTTTGATAGTCTTTTGTTTTAAAGGAGAACACTCAAATGTGCAATAAACTGCAACTTTTGGGTCTCCAGTTTTGCATTTAGGTCAATATGAGAGGACAGTCTGTGTTAGATCTTTTATAAATCAGTTAGGAAAAGAAATCCTTTCCAATTTAACTTCAGTTGATTGACTTCTAACATTGGCTCATTCTCACAGTGGAAGACAAGTTTTCCTGATTCATATTTTGATTATATGATTGTATAAATCAGTGTTTTAGCTGTAAATTTAAAATAGCTTCTTGTCTGACAGTGTGAGGGGAGCAATAATTAGGTTCTTTTGTGGTTGGTATATATATACATATACATATATATATATCGGAATAATGACATAGTTGGCCTACTCTTAGGAAAGGTAGAACATACCTTCACACTTAACAAGTCATTTTGTCAAATTTCATAATTGCTAGATCATTTGATGTCAATTGTTAATCATTCAGATTATTTTGAGGAACTATTGTTAAGGTATGTTAGCTGAAGCACTTTGGGAAGGCCTTCTATATTTTGTAAATTCACTTATGTACAATGATTCCCATCGTATACCTTTCCTACGTCGCTTCTCAATTGTACTGTAATCCTGACGTTCATATGGCAGCATATATTTGCCAGGATATCTCCAAGCTGTTAGGTAATAACAATCAAAAGATTATCCTACATGTAAATGTGGAGAGACAACTGGTGACTGACAGAGCATCCCTTAGGTTCTGGGTGCAGCATTTCCTACTTAAAACCTAGAAATGCTTCTTGCAGAAATTCTAGGAGACTCTTGTCATTCATCACCCATAACAGTTTGAAACATCCAGTACAGCATGTGTTATTTTGCAAGTTACAAATTTTTTTATAAAAAACACAAAAGTTTAAATATCTTCCTCACTGTTCAAAATTCTCTCTAATTTTTTAGTTCAGTCCTTGGGGGCATGGTGTTTACTATCTAATACCTATACCTGTGTTCACTTATTCAAGATTTGCACATGTTGCAGTGGCTGTTTAACTTGTTATTTCATTTATAAAATTAGTGTTCAGAGTGTTGATGTAAAAAAAATGTAAGTATGGATAATCTGAAAGAAGTCAGTAATTGACCTGTAACATTCCGTTGTAGATGATGACAGCTTTTTTGTTGTTCTCAGCCATAGATTTAAAGGACCAGGTTTTTGTTTTAAAAAGCGGCACGCATCCCTCCCTTTGAAAACCAAAATTCTTACTAATTGCTTCAGGTAGAATTCTAGAGCAGCATGCAGCCATTGCCTGACAAGAGAAAAAGTAAACTCACAATTTCCATAACAGCTCTTTGTGCTTCATTGCCTTGTTGCTTTGTTTTAATTTACAAGTGTCCAGTAATGCTTCTTTCAGCATAAACAGTTTTGTTAGAACCTCATAAATATAAATCATGATAGTTTTATTTTGATACCTTTCTGCTGGGAATTTTGTAATGCAGCCATTTGTAAAAGGAAAGCTCTGCACCAGTGACACTAATCTACCATACAGAAGTTGCACTTTGTCCAAAAAAAGTTCTAGTTCATTTCAGATAATGAGTGCAAATAAAGAGTCAAAGAACCCATTCCCCATTCTCTGGCAACATATCAATCCTTAAAAGTGAGCCTTATGTTTTAATAATATACTTCAAAACTGAGTTCATACAACAATTTACAGTTTAATTGTACTTCAAGTACAGTACAGGGTACCTTTTTCATTGGTGCTGTATATCCGGAAAGTTATAGGTTCCATTGGATGAAGTAATGCAAAAGATTCTTTTTCATTCAAAAGTCTTGATTTCCTTTCAAAAGATTTATTGATAGGTTTAGGTGCCGCTGGTGGTCAACATTATGTACACAGGGTTGGGATGGCGCTGGCCGCTGGGTGTAGGCAGTCTGCACAAAACCCCTGGCTTCTGTAATCCGTCATCAATAAACACCTACACCCAC

>chr40:+:49898852-49900845

GACAGGAAAATTTATTGTAAAAAATTTGATGAAAACAAAATCTAAGAAAAGGTCTGGTTAAACATTTGAAGCATTACCATTAACACTACTTGATTTACAATGAAATATTAGCTTTTATGATCTTCAGATATGAGTAGATAGGAAAAAAAATAAGTTTTGAGTGACAAAAAAGAAAAAAAATGCAAATTTCACATATAACAATATCTAAACCAAATACCATGTCACAAACGCTACACCGTACAATAATGTACAAAATTCATACATCGGGGAATGGGATACAATAAAAATAGTAATAAATATTTAATACTATTAACATTCTAAAGGGCCTATCAAACATAGCTTTTAATCTTATAGGCAACTGAGCCTGGAGATTAACTGGTAACCAAAGAACATAGCACATCAAACTATTCCTTGCTTGGAAAGAAAGAGAAAAAACAAATAGTTCTTTTCCCAAACAATTAATATCAGTTTGTGTCAATTACATATTCCAGACTTTTAAATACTGCCGACCAAGAAAATGCCTACCTCCTTACATTTTCACCCTCAAAAAACTAAGGCATTTTATTCAAATGAATGCAACTCCAAGGAGATCCTTCTACCTTCTACAACATAAACTGAAGCTACAACTTCAGATTAAGGCTTTCAATGTCAATAACTTCCAAAAAAAAAGTAATTAGTCATAGGCCCTGAAAAACATTGGAATTCTAGCAAGTTACAAATGAATTTAAAATGGAAACGAAATTATGATTTTTGAAGGGATCTGATGATAAACTACCCCGCCTAAGGATGTGTACCATCAAAATCAACATAGTATATCAATGACTGAATCTTGGGACTTAGGAGGAGGGGAGGGAGGGAGGGGGTGGGTGGGGCAGCCCCAAATCTTTAACAATGATTAACAGCACCTAAGGATATCGGATCTAAGTATAGCTGGCCAGTTATTTACCTAGGTTTGTGAGGGCAAAGTTTCTAATTCTATCTTTAACAAGCAGCAGACCAGTTTTCCTCAATACACAGCTATTAAATATGCATATAAAACTTGCCTATATGAACGACTGACAATTGCCAATCAGTTCTACTTTTTGTTCATAAAGAAACCATTCAATAAACCATCATGCCATCCACCAAAGACAAACTTGTATGGATTTAATCTGAGTAATCACCATTTAACATTATCCTTAATTTGCTATATGCAAGGATGGTAGATAATTGTTAAATTCGATATACAAAATTTTGAGCAATTTCTATAAAATATGGGGTTTAAGCATTTCTTTGCTACATGCTAAAACAAGCCATCATTCATATAGGGCTGAATAACTGCAATGCTTTGATCAAATATATAAAAAATTAACTTAGATTCACAAGGGAAATTCTTCCTTAACAATGCAATTCATTACTAACCCATATGTTAATGTACAGTTTTCCCATTAATAGTGAGACGGGCACAATTCCTTGCATCTGCCTTCAATTTATACCTTAGTACGTACTTTGACTTTTATTACCTACTAAAAAGAGAATAAGCTCTTAAACCAAAACTAAGGAAACCCAGAATTAAGCAAACTTCAAACCCAACAAATATCTCAATTCATTCAACATCACTAACTTTAGCAGTGCTGTATCTAGTCAAGCTTTTGCTACTTAATCCTTAATGCTATTCATCATTTTCTAACCTGTTTCCTTGGTAAATTTCATATCAATACAAAGACCTAGCAGTCTACAGAGTAATTCTGATCATGCTTCCAAATGGAAAGTATGCAGAAAATCATACTATGAAATATTGAATGGAAATTGCTCAAATCATTCCAGTGATATATTGTAGAAACACAATCTACATACAGTATACCCTTTTCATCTTATGATGAAAAGCCCTTTACTTTTTGTGATTGAAGAATTAAAAAAAAAAGAAAATATTTAAAACCTTATGAGCAAATTCTGGTTCCTTCCAGCAAATAGAAGTGACTAACTTCAAGAAACATACAACAAAATTGCTGAAC

>chr41:+:5102597-5104410

GACACTTCCATTATTATATTTTGGTGTTGTTAATACTATTGTCAACGTATAATTACAGAAAGCAGACAAATAAATTAAGAACTAAACTGAAAAATTAGGAATGATCCAACACTTGGACATTCACAAATGCTAAATCCTCACAAATTCACCATTTAACATTAGGTTTCAGCTTCTAGCAACATTTTTAGACCGTTATACCTCTTGATCTGATGAATATGCCACTGACTCTACAGTTTTCTCAGTTATATGAATTCAGCTTAAATCTATCTAGATGCTATCTTTAAATGGCAAACACTTGTGGCCATTTTTAAGCAATGTGAACTAATGTTAACAATCTGTAATAACATTACTACATAATGTATACAAATCACACATATATAGATATATAATGGTACACTACACACTTTTAAGGAATATTTTTGAACTAGAAAAAAATTTATTGTTATAAACAGTATGCACTGGAATGGAATAGTATAGAGTTTAGGCCGTGTTCTGGGATCCATAAAGTCATTCAGCGCTGGAAAGGAAATTGAGAGTAGGTAGGTTTGAGAGATGTTAGAGGAGGAAAACCTCACATTTGCACTAAGAAATAATTGTTAGAAGGTGGATAGAAATAGAAAAATAAAGAATTGAAATTGAATGGAAAAAATACCCATCAAGAACCAGGTCATACCATATGGTGCTATGTAATGCTATTTTCAGATAAAGTATGGTCAAAGTTAGTGATTGATAGGTCATATTTGGTGAAGAATGTTTAGTTGTTGAAGGAAGTAGATTACACTTTCTATGGAGAGGTGTTCTGCTTCAGCGAGAATATCTTGAAGGCTTTAAGGTGGACGGGAAAGAGAAGTTAGGCTTGGAAATGCTCTGCTGCGTGCGACTGTGTTGGGGGCAGGAAAGGATTATATGGGATACTGAAAGAGGGCTATGACAAGTTGGACACAATGTTGGAGGAGATTCACTCATTAAACGGGAGTGACTGATAGGTAAGCAAGATGAAAGAAAGATAATATGAATGGAGGTACAGTAAAAAGTGATAAAAGGGGCTGAAGGGACACTGCATTTAGACTGAGTTAAGCAATATATGTAGTATCTATTATGAAAAAATTAATTTAGCTTGTCATTTAGTTATAAAACTAACAGGTCTTAACATCATTTAATGTCTACATGAATAAATCTGCAAAGCTAGTGTACTGTAGACAGTACGTATACCATAGCTAATATGGTTACAATTATAACTTCTGTGATACCACGCTATTGTTACAAAATAGGGGTAAGCAGGGTATCTAACAACTGGAATGAAACTAATGAGAATCCATCCAAATACTGAAATTGCCTCTAGTACACCTGTTCATTAGCATATCAAAACTTCTGATATCACAACATCAACTGAGATATCTATGAATACATACAATTAAACTTCTAAACAATATCAAATCCATTTTGCCCCAACAGTATTGTAAAATGCAACAAAGTATCAAAAAAATAATTCCCACTATAAGCAAGATAGTACGTACGTTAACTAAGAATACTACGTAGCTAATGCTTGCATGTTAAAAGAACATGTAGTAAAATAAAGCAGAATTCCTTTACTAAAGCATGTTCAAAGTACGTACCCATAAATAACAAAAGACATAATGTTTCTCTTGAGAATTCCATACAAAGATACAAAAATTCTTCTGAGCTACCTTCACTATATTACAAAAAAAAAAGAACATTAACCCTCACTACTTACAACTTTTTTTCTTACTTATATGAACATAATTCTACAGTAAGAATTTTCACAGTTTATGACTGATGTACTGGAACTTTTC

>chr41:+:5165880-5168544

GTCAGCTCCAGAAATCTGTCAGTCCTCTGACAGTTCCACAATGGCGAAAGGAATCCACTCATATTCCCGATATCATACTAGTTCTGGTTCTAAAAGATATTCCAGTTAAAGTCTGAGTTTACTGACAACTTTGACCGCTATTTCATGGCAGTGAGCAACTCAACCAATGAGAAATAAAAATTCCCAATGCTCCTTTTGAAAAACTGAAAGGTTAAATAAGAGACTGGTCATTAGTCTCAGTGTTATGGATTGAATAATTTATGAAAATTAGGCGACAACGTTTTTGTTACACAACAGTTTTGAAAGTAAAGGAAAACAGCAGCTGTTTGTGGCATACAAGGAGCTGGAAAAAAAAACCCTTATGATAGATATGTGATGAAAGTACGGCATGTGACCATATGAGGTGATGTAAGAGAAACAGACCTATATTGTCTATGGAAGCCATAGCTGGAATGCTAAAAGGAATTGCTGAGCCAACTCTCCTCTATGGAAGCAAAGTGTGCATGTTAAATGTGAACGAAATAAAAAAGAATAAGGCTGAAGATATTGCGATGAACTATTTGCATGATATATGCAGCATTAGATGAGTGAGAAATATGATGCGTGCATTAGTCATAAAAATGTTAGTACAGGCAAGACGAGGGATTATACAGTATTTTGAGATGGTTTGGGTCCTAAAGACATAATAAAGGACTGGGTTGGTGAAAAGGGATGGAGAATAATGGGACGTTTACGAGAAAGATGACAAGAAGACATAAAAAAAAGGTGGGGGGGTGGAAAGATGGCTGATGAGAGTGCGCTGGATTGTGGAACAATCTCCTTGAAGATGTCGTGCAACTAGAACCTTCCCCTTGTAGTATTTTAATGGTTTGCTTACATTTGATCAATTTATTTATCAATTTGTTCGTTTATCTTTTATTTCCTAATAACCGATTGCTTCTTTCTGCATTCCTTTATTCCTATTACCTTCCGTTCCACCTTTTAAATGAATGCCATATTCTTTGGAAGCTTGAATTTCAAGTCCTTGACCACCAGTGACCTTGTTCATTATAAATTGGGTTTATCTTCTGAATAATAATAATAATATACTTAGGAAGCAGACCCTCTCTCAAGCATACTTTATTAAAATTAATGGCTACTTCAACAGCGTTACACTTGTAGAGATTCTTCTCTATTTTACGAATACCGGCTTTTTCCGTGCTACTTAAACAGTAGAGCGCCTATAGAGCTCATGATCAAGTACAGTGTCAAAATAGGTGTATATATTTGAATTTTACAAATAAACTATGATACCATAGTCATTAAAGTCATTAACGATTCTCTCTCTCTCTCTCTCTCTCTCTCTCTCTCTCTCTCTCTCTCTCTCTCTCTCTTTTAGACTCATCTAACACTGTCAGTTATTGCGTCTCTGTGCACTCTGGAAACCTACCGACATGAGACCCATTCTAATTCTCAATTATTGCGTCTCTGTGCAATCTGGAACCTTCCAGCATTAGACACACCTAACTCTGGAATCTACCAGCATTAGACTCATCTTACTCTGTCAATTATTACGTCTCTGTGCACTCTGGAACCTTCCAGCATTAGACTCATCTAACTCTGGAATCTACCACCATTAGACTCATCTTACTCTGTCAATTATTGCGTCTCTGTGCACGCTGGAACCTTCCAGCATTAGACTCATCTAACTCTGGAATCTATCAGCATTAGACTCATCTAACTCTGTCAATTATTGCGTCTCTGTGCACTGTGGAGCCTTCCAGCATCAGACTCATCTAACTCTGGAATCTACCACCATTAGACTCATCTAACTCTGTCAATTATTGCGTCTCTGTGCGCTCTGGAACCTTCCAGCATTAGACTCTTCTAACTCTAGAATCTACCAGCATTAGACTCATCTAACTCTGTCAATTATTGCGTCTCTGTGCACTGTGGAGCCTTCCAGCAACAGACTCATCTAACTCTGGAATCTACCACCATTAGACTCATCTAACTCTGTCAATTATTGCGTCTCTGTGCGCTCTGGAACCTTCCAGCATTAGACTCATCTAACTCTGGAATTTACCAGCATTAGACTCATCTAACTCTGTCAATTATTGCGTCTCTGTGCACTTTGGAACCTTCCAGCATTAGACTCGACTCACTCTGGAATCTACCAGCATTAGACTCATTTACTCTCAATTATTGTGTCTCTGTGTACTCTGGAACCTTCTAGCATTAGACTCGTCTAACTCTGGAATCTACCAGCATTAGACTCATCTAACTCTCAATTATTGCGTCTCTGTGCGCTCTGGAACCTTCCAGCATTAGACTCATCTAACTCTGGAATCTACCAGCATTAGACTCATCCTACTCTCAATTATTGCGACTCTGTGCGCTCTGTAACCTACCAGCATTAGACTCATTCTAACTCTGTCAATTATTGCGTCTCTGTGCGCTCAGGAACCTACCAGCATTAGACTCATCTAGCTCTGTCAGTCACGATTCTGAATTCCTCTCCTTCATCCCACCTTCAGAGGAACCAAAAACAAAAGCATTATATCAGGTCATACTCTGAGAATCCGACCACTCACCTCAAATTCAGGGAATTGGTGAATAACTTGAGTACACTGATAAAAATCTACTTGAGTCTCCAACAGCTCTCGACAACTCTACCTCTCCGTAT

>chr41:+:12610466-12614124

GATAAAAAGTCAACAATGTTTATTGTTGCTCATGATATAATGTGCGACACCTAATTTCAGGCAATGTAACTTGTCTTTTTCGTGAATACAAAAATAAGTTTCTGCATGTATATTACATTGTAATAATAAGGTATCCAACTACCAGTAGATATGATACATGTACAGTACAACTAAGAAACTTAAGGTATATATTGCCCCAAAATACAAAATAAACTTCTGTTGTATAATTCCCAATAACATAAAATATACAGTAATTACATTCATATTCTGTTGGGTGAGTATAGATACTATATGTTTAAAAATAGCTAACAACTTAAAAGTTACTAAAGTACAATACACAAAAGTAAGGGATGGCAAAGATACGACTCTCTAAGATCTGAATCTGTCTCTTATTAATATTTCAAAATGAATCCTTAATTCTAAGAACAGCATACTTTCTCTTTTAAACTGCTGTGTACCTAATTGCAGACAAATCACATTTCTAACTAAACTATTAGAAAGCGGGTAATACTGGTAATCCTTTTATTATATCAATTAACAGCTATAAATCAAAGTGTACCAACTGTTTAAAATGTTAATATATAAAACTATCTTGTTTCTATGTCAGTTATGAGCAACAGTAGACTAACGGGGATGTTCAAGTATAATCGGTTGGTAGATCAAGAAAGCATTAGTATTTCAAGATTTCCAGCCTTCTATACAGTTTAACGCACTGCACAGCACAGTAGTTATACATCAAAGTCCTGTTAAACAGAGCAGTTTCACAAACGTCATCCTGCTAATAGCAATATTCAGCCAATGACTGGTTATTTAAGAAAGTGAGTGAAAAAGTAACAGAGAGAAGGATAAAATTCATGTGACTATTTTCTATACATCTACATCACTGTGGAATTGTTACTGAATCTCTGAGGTTTTATTTTTTAATGGTTCATGCCCAGCACAATCATATTATCCTTAGATTATCATATATCAGAACATACAACCCTCGTATTTTTGGCTTTCCTTATGCTTAAACACCACACACTACTGTAATGGAAGCAAAAAAAGGAAAATTATCAAGGGCAACGACCAAATGAGAATTTAAGATAAAGTGAACAGGACCAAAGAGCGGTGAGAACTCGTTCCACATCTAACTTCAGAAAATGAAAATTTGGCATTAAAAGCTTGATTGATAAGTGAATCTAATACCACAGTCTAAATCACATAGGAATGTGAAAAATAACACAAATAAACTAGGATTAATCCCCATACCTTATACTTTACTAGCAAAGAACAGATGGACTATTTGGCATAAGTCTCAAGGTTCTGTATGAACAGAGATGAAGTGCATAAAGAGAAGCATGAAAGATGTACTTAGAAAAGAAAAAAGCTGAATTTAAGGACACAGAACTAAAGTGAAGGGCCATGGTATTCAATTGGATAACAAGATTGTCTGGTGCAGCTAAGAGATAAGTGTAAGGGAAGGGGGAAGCAGAGGGCAATTGCTTGTGAAAGTCCAAATCATGCCTAGTTGTGTTTGGATATGGAGAAAGGATACGTGCGCAGAAGGAGAAAGGTTGAGTGTACTAGTCGATATGAATGCAAGGGTTATAAACAGACACAAGGAGGCTTGAGTCCTGGGAATGTAATAATGAAAGAGAAAATTAAGGGGTATAGTTTCTTAGATAACATATTAATTCAGAGTAAACAACTGAGTGGAATATGGCTATGGAAAAATTTGCTCAAGTAAGCTTAATAAGAAGGAATTTTGTGGGATACCTGTAGGGAGTGTAGGGAAAATGGGAATGGATTAAGGAGACAGCAAACCAGTAGGAGAGATGGTGAGTTTGAGCTGCATGCAATCAGTAAGACTGCAAGAAGCACATAAGAAGGTGAAATAAAAGTTCATTGCATGATAGTGAATGAGGTACAGAGACAAGAAAAGAGAAATTGTAAGTGTATTTGCAAAATAAAAGTATACAGCCAATCAGACACATATAGAGGTAAACGGTACAGATGAAAGAGGTGAAACAAAAAAGGCAGGAGCTCAGCATAAAGTCTGGAAGATAAATGTCAAGGGAAGCAAAGCTGGGATGTTTTAAGTGATTGTTGCATCAAATCTCTTTTCCATGAGGCAAATGTGGGTTTTGAAGGCAAATGAATGAAAATAAGCTGGAAACTCTTGAGATGAATATGTATCAAAGATGGTTGAAAAGGTAAGAACTGTATGCAAATAATGGACACAGTAAAACAGCTGAGCAAAGATAAAAAAAATTAGTTTTTGCAGGTGGTCTGGTCCTGTGACCAGAAAGGAAGAGGACAGGTTGGTGAAAAGGCAGCATAATTCAGATATGTAGGGCGAATGAAGGAAAGGGAGAATCTAGAAAGTGCTGGCTAGACTGGCAGAGGTGTTAGAATGAACTTTAATATTCAGAATGTGTTTGTGTGCAATAACTGAGACATTTTTAAACTGTTTCCTTTAAAGCTAATATTGTCTGACAGTTTAATGAGAGGACAGCATGTGTGTGTAAAGACTTACAGAGGATGGAACCTTTTGAAATACCAATTTAAAATCCTTTATGACAGTCTTACTAAAAAAGATCTTTTACTATTTGATGCTGGTATGTCTCATTTCATGTCTACACATAAGTTTTGAGCTTTCTGTAAAAGTACACTTTATACAAAACTGTTAAGCTAATAAAATTGCACACTTACAGTTACAAGAGAAATTTCAAATGAGATGTCTATGTAAGGTGATCACACATGTTGTGTTCAGTCACTTCAATACCATACCTAAGACACTAATCAAAAGAAAAGTTTCACCTGCACACTAAATATTTATAGTTTATGGCAACTATTTCCTGATTACAATGGTAATTTGCCATACATGGTATTGAAAGTGTGAATTATCAACACTGCAAAAATTACCCATTTGCATATATAACAGAACGTACACCTGGGCTTTCCACTACTAAGAGTAATGCTCATAGTATTTGCCCACTTTGCATGTGCTTGACAAAGTTCATAATCAAAATCTCAACACTTTTTCATTGACATAAGTCTTAATGTTTCAAATTCCTTTTTTTGCACACACTGCCTTTGTAACCTCTCAATATTCACAATTTTCTTATCCTATCTTTGACTATTTATCCTTCTTCACTAATAAATGGCCCCAGGAAAATTCAAAATAGCTTGAATAATGTTACACTATCCATAACATCTCCGTTTTTAAAACATTTCCTCTCTTCATAACTTCACAAAAATTCTTCATAGTTCTACTAAAACACACAGAACTCAAAATATTGATCATGGTTATGGGCTGAAATTTTGCTACCAAGTAAATGATGGTCCCATGGTTGCAAATAAGCTACAGTTACCATAATAAACTGACTAAACCCTTACTCGAATGAGTAGAAACAATTTTTATAAGCCAGACGAAGGTAGACTTTTACCATAGTCTCTAATCTCATGACTTTTCACTCTTTTAAGATATAGCCAATGGTCAATATAAAAAATGCATGTCAGTAAATGTAACTCTTATACATGAATATGTTTCTCACACTGGAATCAAAATACAAAAACCAGGTGGAGCCACCAAAACATAACGAAATTAGAAGTGAGTATCAATAGCTTGTTACTCATGAAATGAGAATTTTATGTGTGTAATCTTATCGATATAT

>chr41:+:18974189-18975595

GGGAGGCGGGCGTAAGGAACCATTCCCATTTTCTGTCAGATATTTTCTGTCGCCGGTGCAGACAACAAGTGTTTTCTGCACCTCCGTCATTGGATTTTGGAACTCTTTTGGTCACTTGAGTATCCCGATTGATTTTTGGTTATTTGATTAGGATCGGTGGTTAGGCATACGCTAATTGTGGATTGTTTTTATTTTGGCTTGATTTTTCCTTAAACTTACGATGTCTGGATCTAGTTCGACTAGGCCAGAGTATGTTGTGTGGAAGAATGCAAGGTTAGGCTACCGAAAACTTCGGTAGACCCTCATACAGTGTGCGCGAATTGTAGGAAACATGTTTGTATGTTTGATGAGCGCTGCAACGAGTGTGAAAATATGTCTGATTCTGCGTGGAAGGCTTATGAATCATAGGTACGTAAACTAGAACGTGATAGTGTAAGGAGGTCTTCCTCCAGGCGTGTAACACAAGTTACCCGTCCAGTAGAATTTATCCCTCCAAAGACCTGTGATGCCTTCGGGCCCTACAATAGTGTCTTTGGAGGGTAATACCCTATCTGTGATTCTTAAATCTCTGCGTAGCCTGGAATCATGTTTGCATTGGAAAGTTATAGTAGTGTAGTGAAGAATAGTGATAATGCCATGAGTGTTCATTCTATCGCGTAAGCGTATAATTTCTCATTGACAAAACACTACCGCGTGATGGCTCTCCCTACCTCGGTGAGGCAGAATGTAGTAGGGAGGTAGCTGTCCAAGTGTCTAAAATACTCTACGTTTGTTCATAAAACTTACCAGTCAGATATATGTATATATATATCTGCCGGGTAAAGGTGAACAAGCGATGTTGTATCATAGTAATATTATTTTTGCCCTTACGTCATATTACTATCAAACTGTTGTATGGTTCAAGTTATATACTCATACCATAGTTACTCCTACGGAGGACAACCGAGAGTACGATTATTCTTATTACATTTAATCGGTTCTCCCTGCAACTATTCAGGGGTTGCCGGTTTCCCTATTTTTAAACATTTAAGGTATTGTTATGACAATACCAAGTTAGCCTTTTATATTTAGCAAATTCAGTTCGCTTAAATATACTAGTTTGATGGATTTTGGTTTCTGCTCTATAATGATAGTAAACCTATTCATTCGTAGAATGGTGTAGGTGGCAACTCAGGCAGAGCAGTGCGAGAACGACGAACGTTCTCAGAGTCTGCTGTCACTAATGCGTAATGCCAGTGCTCAGTTCCATCTGATTGTCTGCTATGCGCAGTAGGTTACGTCTCTCTCTCTCCTGCGGGATTGACGGACTAACCGTATTTCTACCCTACAATCACGGCCTTAGCCTCGGGTTGAAGGGAATTCTAGCATACATGGCTGAACATCTTCACTTTGCGAGAATTTTTTCAT

>chr41:+:19100908-19103427

AACCCAGATAATTTATTTGATAAAACCTTAATACTATACACTTCTCTTTATTTGGACAGAGCTAATCTCATAACATTAATGCTCGCATATTAATTTCAAACAAAGCATGGGTTATATTAGTAAGCTGTAGCTCTTAAGTAAAATTACAATGCTTTGTAGATTTTGATAAATATATATAAAAAGAGAGGAAACTTAAAGAACTAGCAAAACAAATCACAAGTAATGCCCTTCTACAGGAACAATGTAAAGTACTTTTAATCAGGTCACTTTCATACTGAAGTAAATCCAAATAATAAGTTCTTATTATAACCTTCGATGTTACTCATTTATGTATGTATTTAAAGTTTACAGCAGAAAAAATGCTTCTATGTCACAAGAATAACAAACGCAATCACTTGAAATGGTATTTTAACAATATGCGAGGTGGCTGTAGATTAAGCTAATGATTCTAAGTCAATGAAATCTATGAAAAATGGTCCCAACCCAAATGCAATTGTATAATGATGTAAAAACATGAAATTTTGGTTAAAATAAATTCATGAACCTTGCTAATAAATTTATTCTATCACGAGATCAAAAGAAAATATATGACGTAAAAGATATACGTAGTACTATCATGTCAGCATAAATGACATCAATATGAAGGACAACAGAAATTATTTCTGCCAAATCCACACCCACGAAGAAGTGAAAACTCTCCAATACTTTATTGTTTACTTCTTGCTAACACTAAATATTGTTGAAGGTAATATAATCCTTTAAATACGACGTCATAGGGACTTGGTGCACTGAAAGCCACTAAATGCTGAAAGTAATGACGACTGTACATGCATATTTCACAAACACATTGACTGATAAATATTTTTAAGACATTTTACAACCTTAATCTTTTTCAAATTAACTTCTCTTATTTCAGTTTGATATTTTGCCACTCTTACAACACTGAGGATGCCACCAATTCTTGGGGATTCCCAATAGTAGGAATGTCCTAAACCTGCCTTGTGTGAACAAGTGCTACAATAACTTTTAAACATCAATTTCCAGTGGTGAAAATACTTTCTTCTTAACTATGATTATACTTGTAATAAAACTCTGGCCAGAAACAACTTATTGTTAATAGAAAATATCAACTTACTTAGGGGATCTTCTCTCAAAATTATATTTAAACTATTCTGTAGTGTCTTTACTGAATGATGCTCTAACATGTATGACAGTGTTGACATACATTGAAAGTCTAGCGAAAAAAATGCGGTCAAATATTAAATGGCTTCTCTGATAGCCACATATGCAGTGATCAATATGTGATTAACTTAACATTACCATTAAAATGTCCACATGAAAGGACAAAAATTCTTCATTTTTTGTTTAAATATATCCAAACAGAAGCATCACTTGTATCAAATATACACTATATCATTGATATAATGATTAGATTCTCAGTTAAATAATAGAAGCTATACTAAAGTATACGGATACAACTTGGCTCATGGTTTAGAAAAAAGATCTTCACTAGAGTAAATTGAACTACGTAATTAGGTTGTTTCACTATGGCTACATTCAAGCTTCACGTTAACTTTTGTGACCAAATGAGGAGAATGGTGAAGATTAAAGATTAATCATAATGTATGTGCTATACTGAAATTAAGTACTGTATGAGCAGCTGGTACACAAGTACCTATATAAAAAAAAAATAATTTTGAGCTTAAAATTAAATGACACTTTTTACATTCGTACGAGGCTTTTATTGGAAATGGTACTCCTGGTATGTGGAACTAAAAAAAAATTAAATATTTGCATTACACAGTACATAATAGTACTGAATAATATTAGACGAACAATGTTGTTTCACACGTCTGCCTCCCATAGTTAAAAAAAAAGCTGTGAACTTTAGTAAATGGATTGCGACTCATTCTAAAATCTGAAACCCTCATTTAATTCCACTCCCTGTCCTATTATGCACTACAGACAAAACACTGACTGAGCTACATGGTAAGATGAGAAATCTCACATTAAAAAAAGAGGTTTAATAAAGAATAACATGATGCACAGCACAAAGATTATCATTCCTTGCTTGGAAAGAAATATGTTCATAAACAATCATTTGCATCTTACAGTATAACAATGTCAGCACATAAATCATCAACAATAAATGCACACTATACAAAGGATGCATTGAAAGAAATGCACTCTGTGGAAGTTAAGATGTGATATAAGGACTTTTTAAACATTAAACACCTAAAAGTTCAATGTAAGCGTCATCGTCATTTTAAATCTATAATAAACCAGATATGGCCATAAAATCTTCCAAAGGCCAAGAATCTTCAGGAATGATATGCTACTATAATCTATGACAGCTTATCATCATTGCCTGCACCCTCACAGTCTCGTACTAACAAATACCTACCATTTCCATATCTACCTCAGCAGGTAATTGACATCACAATCAAGTCTTGCATATATCCTGCCAGTATTTCCAACAACTCTGACCCACCATCCTATTTCATAAGTCAAGAACAGGCC

>chr41:+:21885322-21889652

GCTGCATGCAATAATGTAAATTTTTATTTTTTTTTCTTTTATTGTTCAGTTACACCACTCCTAGCAGAGGTCGCAGCTCTGGGGAAGGGTTCCTGCTCAGGACTAATCACAGGATTACAAAATGTTCATAAGGGTTAGAGAGAGAGAGAGAGAGACAGAAAGTGTGTGTGAAAGAGAAAAAAAAACAAAAAAAACAAACAAAAACAGGAACCCTTTGTAAAGAAATATCACAGGGTATACAATATGGACAAACTTATGTATAAATGCCAAGCACTTTTAGTCGTCTCAACACCTTGATCTTACCATTTTCTCAACACTAAACTCTTCCAAAAGCATTAAGACCATGAGGCACCACAGCTTCACAGTAGTACCAGCAATACAAGGACTGGACGAGGCACTGCTTGTGGTAAAACTGCATCAATTGCCGCTAAATTTGCATAGACAATCTGTCCTAATCTGACTTTCACATTTCCTTTCTTTGGTAAAATCAAAAGATTTACTGGTCAATGTAACAACTGAAGTCATTAGCTCTCCATAGGACTGGAGCTGTTGATGCCAAAACTACTCGGATAATGTAGACTTGCCCATCACTGAAAGACCAAAATCTGTCCTTGACCAAGTAGGGCACCACTACTGATGAAGCAATATCTATGGCAATGCTACCAACTTTTAAGAAGTAATGTGTAGGGAGTAAATTAGGAGAGAGCTGGGCATGTCAAAGTGACACCCATCTTCCATCCGGCAGCCTCATGTACCCCTCTACATCACACCAAAGAAGTAAGCAGCAGGTTCAAATGCGCACCACACCTACTCGATTAACCATCATCTTGTTCTGGACAAGTAACTTTACAAGGAAATTCCATTTTTGGACGACAACATATGGTCGGTATTAAGCTTTGTTTTGGACAAATTATTTGTCTTGACTGTAAATATTTGGACATTTCCAGCTATTGGAACTAAGTGGACACTGGACCATTAGAATTTCCTGCTATGGACAAACTTTTCCCTATTTGGCCACTAACAGTGGATTGCTAAATTTTACTTTACAAAACCATCTTAGCTCTTTGGATTGGAAAATGCTAACATTTTCAAAAAATAATTAAATTAAAGATTAAAAGCTAAAATGATTTTTTTGGTCAAATCAGGGATTTTAAAATACACTATTTCAAATGTCTTTTAGAGGATTCTCCAGCAGCAGATGTCATCACTATGATGTTTGTAGCCTTTTTCTTAAAGCTTTTCCATATGAAATTCCTCTCAAAGAATTTCAACAACCCAGGTGATGTTTTAACTGTGCATAAAGCACTTCTGAGTCAGATCACACTTACTATATTCAAGTACAGACCCAAAACAGATTTGCTATACACAGCTGTACAAGAACAATTCAATGTCAAGTGTCTGACTAAAGAAGTGCTATAAAACAGCACTTATTACCAAAACTGTGATGGCGTTTCATTCTTGGCATAACAAATATGCATGAAAACACTAATTTCTAGTTGGCATCATAGCAGGGATAGCTAGCTGCATGTGCAGAACATATTTAAACATATTTTTTGCATTTTAACATAAATGTTATTTTTATTTTCATTTTATTTTCCTTCACACCAATACCCCACTTCTCAATATTAAACCCCTATAATATTCGGATATAAATTAAAGCATAAAATAACAATAATCATAAGAAGCTCAAGAAAACTGTACAGTAATAGTGGAAGCATGGAGAGGCCTGGCCGGGCAGAAGTCGTCAACGTTAGGACGCCTTGTGTGGTATACAGATACCTATTAGGTTTCCAACTGCACACCATTTAATTACATTATGGACACTCGATAAATTTCACCTTTTATCAAGGGGTAGTGCCTCTTTCACAAAGGCACAAGTTTACATACCGACAAATGGCAATGCCACAATGATATTGCCCATAGGCAAACAATGAAGTTAAAAATTTCAAAAACCATGACTGAACACTTAGCTCCTAATACACATGACCAACATATCATTAAGATATGAAGACCTAAAACTTCCACATTCTCACTGCTAAATCATGACTGTACTCATTTGAAAATAGAAGACAAAATATATTCACGGCTCTAAAAGAAAACTAAGTATTCATGCCAATGACTAACTTGGGAACTTGGCTTACATTTTCAGTGACAATAACTGTTGCCTGTACCATTGTTACTCGTGCTACTATGCCAACAATACTGTAGTTTAAAAATAAAAAAAGAAAATAATGTCTGAACATAGTTTAAGGTCCAAGTGGCACTATATGATAGCTAAGTACACACCAAAGAGTAGGTCTTGAGTCAGCCTTGGAAATTAGACTGCTGCCAAGATGCCTTCACTAACGTGTTTGCATACTTCCCAACACTACCCGTTTTAATTCTCATCACAAAAACAAACAATTACTAACAAGAACAACCAGATTGTGAAAAATTTTCCACAATTTATCACACGTTTACTCAAAGTAGGTAACTTCACTAAACTTTGTAGACAAAAGAATTAAAACTTTATCTAAATAAACACAACATGAGAGCAACTCCAATATGAACAAACAAATGAATGTTCAATTTGTCTCCTCACAAAGCATTTTCAAGCTCAAGAGATTGAGAATTACCTTAAATGATGGAATACAACTTGACCCATCTCCGCTTTTGTGACTTGTACGTATTTCCACATATTGACAGCAGCCTTGTAAACCATGACAGAAGTTTGTGCACAGGTCTAGTCACTCCCATCAGTCCCTTATTCAGTCTCAACATATTTACTAAATATAAACTTTAAAAGTTCACTGACTAAATTATTTTTGGATTACAGTATTGTGTTAACGTGTTTATCAAATCTCAACTTCAAAAATGGTTTTGTGATTCAATTAGTTTACCATAAAACCCTGCTACCATGCATTCATAAAAAAAGTTGCAACAGATAAATTTCCTGTGACAAGTGGTGTCAGTATTGTATGGCACCTAAGGTGAGGAAAAACAAGACAGCAAAACAGGTTTCATGAAAAGTACAGCAGAAAGAAACTTGATCAGTCAAGCACAAACTAGATTTTTCAAAAACTGAATAGATAATTTCGTACTTCTCGGATAGGAAAGCGGCAGTTTCAAGAAGTGAGAAAGTCTAGTCCTTTTTGTCAAGGTCACGCAAGTCCAAAGTCATTTGTCTACCAATATTTATTTATGCATGTTCTATGAGCAAATACGTTCTTTTCATATTATATATGTATACTCAAACATTTATTTAAATACAAAGTACATTATACTGATGGACTCCACAATCTACAAGTTTGATGCATGCCGGGAAATATACCTTTAATGTTTATGCCCTTCAATAATCATAAAAATGCACATGAATATAACAAAATCAGTTGTGTTGGAATGTTCACTCAAGCAGCCACAATAAATTCATGGCACACAGCACAACAAATAGTACTGCACTTCAATTGTAAATTCCTGCATCAGTGCAAAACCCATTACCAATAGGAACTGATAATGAAAATTTTCTTAACACCATAACTACCCAAGAAGAGTAGTTCTAATGTTTTACAACCTAAGCTACATACATACTCTTTAAATACAAAAACTGGTGAGAAATGACAAAAATTCTAAGTGATTCTCCAGTATCAATCATGCTTAATTCCAAGTAAGAAAAAATGAAAGTAAGTCATTCACAGTGACTAGGAATCAAACTGCATAAGAACCTGCATCTCTGGATAAAGTGGCTTAATTGAAAACAGCATTTCAAAAAAAATTAAAATACCTCTCTTCTTTGGGTAACCAGGATAGTTATTACAGAACCAATTCCCCCTCAAAAAATAAACAAACCATAAAAGGCGAGATTAAGAAAGACTAGGAGATCAAGTATCATTTCTAAAAATATCTTTTTCATAGAATATGTACTTAAAAATATCAACCGCTTTTCATACAAGATGCATGATCTTAGTCCAACTAGATGACCTGTATCACAGCCACATATAATAATATTTCAATGGCCAACCAACAGATGCATAGTTCGTGAAGGCAGTTCATTTATCAATAATGCATCCAGATAAAAGATCAAATTTATCATCTTAGCACAACCTCAATGGCAAGGTCTATCATCATCTTGAATATGACATGGCAATGACATGAAACACTCATTAATTTAGATACCATTTTAAATCATATATACTATGAATTGCTGCTGTTTTTTTTATTTACACTTCAGAAATGAAAAATACAAAAAGTGTGCCAAGGGCATGACCTAGTTTGCAGAAAAATTTTGGATACTCCTACTATGCAAAATAATAAAATTTATAACAAGTTACCATTACCCCTGCACAAAACCTATG

>chr41:+:21889758-21889818

AAAAAAAAAAAAAAAAAAAACATTGGCTAGTTGGTCTCAATAATCCTAGAGACTAGGTCAC

>chr41:+:23593198-23596229

ATACAGATTCAGTGTATAAAACTGAGTGAATTTATGAAATCCTTTATACAATATTAATTCATCTTTTATCCATTTGCTAAGTATGTGACAGTGAATGTGTGATATATGAAGTCCATAATACAATATTAATTCATTTTCAGTAAATACAATACAGTACTCATGTATTTTCTCTGAAAAGGTATGTCAGAAAAGCATATATCTTAAATTCATTGCCACCCATCAGAACCCTGAACTGCAGATCACAAGTATTATTTTGAATTTGATGTTCTACTATGTGTAAGGCTGGGAGGGATATTGTAGTATTTGCCTGTCAGTTTTTTAAGTACAGTTCTCAGGATATTCCGTGATGTATAGTTACGCTACCTTTCTTTGCCATTCTTTCTGTTCCAAAGTTTATGTTGATTGGTAAACATATTGCAAACCCTATCTGGTGTTATGTTTGGTAGAGATTTAAAGATCTTTTGCGATAAAGATTTAATTTGTGAGAAAAATAACTTTTTCTATTTTGGCAGTGATTCACTATGTAAATATTTTTTTATTTAGATCTTGTCTCCAGTGCAGACTTTAGTGGCATTGCATAGTATCTAGCACTTTAAAGACACTTGAAAGTTATTCCCTATTCTCTATGCAGCTCATTGTTTGAGAGGGTAGTAAAGTAAATCTGATATTAGGGCAGTTGATCTAATTGCACTTACAAAGAATTTTATTTTAAACTCTCTCTCTCTCTCTCTCTCTCTCTCTCTCTCTCTCTCTCTCTCTCTCTCTCTCTCTCTCTCTCTCTCTCTCTCTCTCTCAGATTTATTGCCTGTATATCTCAGAGAAGCCCTTTTTCTCATTTCTTGCCTGTGCCTCTCATAGCTCAATGTTTTGGGTGTAAGTGAACTGGGGTTGCATAATACATACTCGTTATCCTGATTTGTCAGGGCCTCATCCCCAAATGATTAACTGTTTGTGATGTCTGGCAAAAGCTTCTCTTTCAAGTTCCTGCTCAATGTTACTTTCATTTGATGTAATGTAGAGAGACCGGCACTTGGAAGTGGTGGGTATCAAATAATGAATATTACCTTTCACAACGATTTTTTTGATTGGGTAAACATCTAAGAAGAGTTATTAAAAAGGGACAGATGTGTCCTTATATGCACATAGATATGCATATCAGCACTCTTTAAACAGTTTGCTGATAATTATGTTAAAACAGTATGTATTTTTTATTGGCATTTGGGGATGAAGAAGAAAAGCTATTAAGTTTTCAGGGTTGCAAAATATTGAGTAAAGTATGTGTGAGAGATAGGAATTTTAACTCCTAGTTCTGGGCAAAGTATCTCATTGTAATAATAATAATAAAAGAAAATTGCTGTGGTTGGCATACATTAGGTTTTTTTAGATTTAAAGGTGTGTCAGAGAGATCAAGTTGTTATTTTAAAAAATATTGCAGGTTAGGGTTTATCTGAGAGAACATGATTGTTTCAGATAAATAATTTAAATACACTGTTTATTATCATAATCCCATCAATCATGTACTACATGCCCACAGTCATGGTTGCAAATATCCATCTGCAATAGTACCCAACAATCTCTTTTGTACTAAACAGTAGAGGAATGGTAGTCTCTCAATGCATGCACCATTTATACCCTCGGGTATGTGTGATAGACAAAACACTGTTGAATACAGGGACATTTGAGACTGATATATCAAAGTAAAAATATGGGATTGATGGGTTGCTTTTAAAGTTGGTTTTGCCTTATACAAATTTAATTTCCTGTTTTCTATTTTGTTTTCAGATTCAGTCCTAGTACCTGAAAATGTGTTTTAAGTTTTATTTCAAATCCTTAAAGCTAGCTACAGTTTATAAGAATTTGATATTCAATTGTGTAGCTGCTAAACTATGAATTAGTCATCATACGCCACTTCTTGATTTCAGAAATTTACTGTTCATATTCACCAAAAATGTGGAACTACATGGATCATAGAATGAACTTATTAACTTCTCAATCGTCCCTCTCTCTTTTTTTTTTTTTATAATGCTGAAATCTTTGTCTTTTGGAATGCTTTACAACATTCAAGAATTTATTCTGCAATTTAAATTTATATTACTATAGTACCAGTAAAATTAACTTATGTTTAGAAATTTCTGTCACCCTCCCTTATCCATTCCTTTTTAGTTTGTCCCTTCACTTTAAGGTTCTCCTGTGATGAGGAAAATGGCCATGAACTAATCCCCGCTACCAAAAGTATCTGTCTAGTAAAGTTAATTTAATTTACTAAAGTTTTTATAGAGACTTTTATTTACTAGTACATATTATTGATATTAGTTTTTCAGTTGCTTCAGTTCTATTTACTGTATTTTATTTAAGCAGGCTAGCCAATAAATGAAGAAAGAAATCAGAAAAACATTAATACTACTTCCATGTTTTTTTATAAGTATGGCATATAATAATAAGAATGTGAATACAAAGCATTACACATATAAAAGTTATTTGTTTCATAGTACCGGGGAATATTCGAAAACCACTCCTACAAACCAAAGCATGTGTATTTGACCAGTAGATATATGGTGTGTTAAAATACACTACTGCAGATGGCAACATATAATATTGAAAAAATGCACGAAGATTGATTGATACCAATTGAAACTAAATTGCCAAGATTAACGTGGAGCTAGTAGTCTCAAAACCTTGTTCCAAGTAGCATTGTAAGACATCTGCCCACTTTTGTATTTGAACAGTTTGAATTTTGTATGATTTTTTATGGTACAATTTAAAATGAAATTTTTTGCAAGCTAATGATATTAAGAAGGTAATTTAATTTTTGAAGGTACCAATTACAAGGTCGTTATACCATATCGGTTCCATTCATGTAGTGACTAGTAGTACTATAGGAGAAATATGTATTTAATTTTTTTTAACTGTAAAACGGAGAAGGATCACAATGAGGATTTTGGAATGAAATTGATGTAGTTGATGTTCATGGATCATAATATGTATAAATGCTATAGTCATATTCCAGCAATTAAAAATTTTTACCTCAAACC

>chr41:+:42195068-42198267

GTGATTGAGCTTTAGAAGTGGAAGGTTGGGCTGTTTGCACTAAGGGCACCGCTGGAACTTGCTGAGGAAAGCGTGGCTGCTTTTTCTGGTAAGGTTGGAAATGCCTAGGTTTCCTCTGTCCCTTACCTTTAGGTGTTAGGTCTTGCCTCCTCTTAGCCGTAAGGCCCCAACGGTCCTTAAGGCTCTGGTTCAGCCTCGTAGCCTCTGACTGGACTTCCTTCACCATAGCTTCTGGGAAGAGATCTGCTCCCCAGATGTTAGACGAGAGTAACCTATTCGGCTCATGCCGAATGGTTGCCTCTTGTAGGACATGCTTCCTACAATTCGTTCTAGCAGTGGCAAACTCAAACATATCCGACTGTACCGTTTGAGTCAGGGATTTGGTCATGAGTTTAAAAAGCGGTTCTGAGCCATAGGCTATGGTTGCTACCTCGGTCATAGCCATAGAGTTAATTGACCTGGCTAATCGCGATTTCGCGTCAAATTCAGCCTGAATAAGGCTATCTGGGAGCCTGGGTAGCTTTTCACCAAACTGCTCCATAGCACAGTCCGGTTTGAGTTTGCCAGCTGAGAAGGTGTTCGGCAAGTCTTCCCACAATTCTCCGGCTGAGGGGAGCAACGGAGATGTGGGATCTGCTTCCTTCAATTGAGGCATGGGTTCCCCTTCTGGGCCGCTGGGATGGTAGACCTCGCGATCTTTGTCAGGAACGGGAGCGGGGTTACTCTCATCCATTGTGAAAATGGTAAAGGGACTCTTAAAAGGTTGTATCTTGGTATTAGAACAATCCATGTCCTCTAAACACCTGAGCCATTCTCTCTGAGCCTGGTCTCGTGAATAGAGGACTGTCTCCTTTGGTACCCTATCATCCCGAACCATGGCTGAAGCTGTAAGCCTTGCGTAGCCTATGAACGGAGGCTGGAGTCTTCCGGGTAGAACTCGAAGTCCTCTATCCTTCGAGTTCCAAATTCCGGTATAGAAATGAGACCGTCTTGGAAGGGGGCGTATGACGCTACTCTCCATGGATTGTTCATCGAGAACGGAGGTAGCGAGTCATACGGAGGAAGCTGAGTTCCACTCGTATTGGAAAGTGGAGGAGAGGCTAGAGGGGCTTCTCTCAGTCCGGCTATAATGGAGTCCTGGGAAGTAATCCTATCCGACAATCGAGAGATCATTTGTTCCATGCTACTCTTTAGGGACCCAACCAGGTCGCCCACCTGTTGCAACAGGCCAGCATTGGAGTCCAAGGCCGGAGCTGCTGCGGAGGTGGAGGGGTGGCTCTGAATCGGAACCAAGGGTAGCGGAACTGGTGACTCTGCCGGAGTGCGAGATCTCTCTCTGGAGGATTTACTCCTCGAGGACTTAGAGCCGGAGCTAGAAGCTTTAGACCTGTCTGCTCCGGGATTCCCAGCCGGAGACTTACGCGAGGAGGATGAAGCCGAAGTCGTCTTCTTGGACGACGACGACGTCTTAGTCAAGGATTTCACTGCTTGACCCTTGACCTTAGGTCTAACCGAAGCGTCAGGAGGAGTATACAATTCATCACCTGTAAAGCCTTGGAAGGATGGAGAGGTTGCAGGAGTAGAAGAAACAGTTACGCCCAAGGGTGACCCTTGGGTGTCCACCGTACCTACCTCGACCAACAGGTCGTCTACACCTACCATAGGTTCCACATTAATATCTAAAGTCGCGACGTCCGTGGAGATATCCTGGTCTTGGTCAGTTAATGAGGCAGCCAGCTGTTGTTGGATGAAGGCGATAGTCGGGGCCGCCTCTACTGGGTCGACGTATCCTGTCGCCTTGCCTCCGGGGAAGATTAACAGCGCCAACCGCTTCTCTAAGATGTAGGGCATACCCTTGGCGGCGTTCTTCCCAAAACCGCCGACCCAGGCCCGCAGGGTTGCCAGTGCGGTATCCCTCACTGCCGGCGCCTGGAAGAGAGGGCGTAGATTAGATTTAAGAATCACTTAAAACTAAAACTTAAAGTATAACTTAAACTTAGATGCCTTAAGTTAAAGTGAATGATGAAAACTTAAGCACTAAAACAGAAGCGGAGCAGCTACCGGAGATGGAATACTTACCCCTTCTAAAAGCTGGCTCACCAGATCGTAACATATGGTGCACGTCTCATGGTACCAGACCTGGATGTCCCCGTGCGGAGTCGCGCATGGAGCATGGGACCGGCAAACTTCGTGTCCACATGGGTCCTGAAGTGTGGCGGCGCATCCCGGATGCTCACAGTTGGTGGCCTGTAAGTGGGAAGACACATGAGTATCTTAAAGAATATCACTTACAGGCTAAAGGACAGAAGAACTCCGTTGCATGCCGGAGCTCGGAAAAAATTTTGGGCATAACCCCTCCCTGCTTCGCCTGAATAGGCTATAATCCCGGAGAGATCCGGTAAGACACGTAAGGGAGGGGGGAAAGGTTTAAGGTACTTAAGATAAACTTATAGTTAATCTAATAACACTTAAACCTAAAACTCAAACGAACCGGACCAAGTCCAGTGCGTAGCGGAGTGTAGTAACTCAGCAATACGGTAAGGTTAGCAGGGACCCACTGTATGTTCCGGTCCACTCTACTGGGTGGACTAACGTCTTTCCGCTGGATGCCAGGACTCCGATCAGGAAAGGAGCCCTAGTAAGGTGGGAAAGAGCGAGAAGACATACAGGCTCGAGCTAACACGGAGCGACAAGGAAGCAACGGATGGGGGGAAAGGCCAGTACCCCCCACCACATTACCAACCGGCCGGACCGAGAAGCCGGAGAGGTCGAGTCCAGCCTGGGTCTGTCCCGTCTCCCTAGCCCCTCCGCCTGGGGGGAGAGAGGGAGGCAGGCTCGGGTATGTGGAGCGAGCATGGGCAGACCGACCCACCCTCCCCCGACTCTATGGAAGAGCGGGAGGGGGGGGGAAGGTGACTGGGCAGGCGTCTGGCTGTCTCGTGATCGCGTAGTGACCACGAGGCGGTAAGACCAAAACAAGACAACTAAGCCTAGGACAAACCAACTGATCAGAGAGATGCTATCGGGAAGCAACTGAACTGAAAAGCAGAAGCATATAGGCCCACTGGGCCAAAACCAACTGATCAGAGAGATACTATAGGGAAGCAAATGCCGAACTGATGAGCGTAGTATAGGCTCAATGAGCCAGGACCTAGGCTAAGCCAGACGCCTAACTAACCTAACCATAACAAAATACATGG

>chr42:+:6099046-6099169

AGAAACGGCCGGGCGAAAGCCGTGGGGGTTCAGAACAGATGGACTCCTCTACGTCATCGGTTAAGCCCCGCCCATAAACATCCCGGCCGAGATACCAGCTTCCTCTTCACGTGGCCTTGATGAG

>chr42:+:6101478-6101757

ACGTTTTCCGAGAACTTCGCACAACAGTTCCTCATCACCAATGGCACCGACGAGCGAGTGATCGATTTATCGAGCTGAAAGACGACACGAAAAATAATTTTAAAAAATGACGTCATCGGCCTGGAAATGGGCTGGATTCCTTCGAAGTTGACCTGATGTTCTCGGCGATGGATGACCTCGACCCAGGTCGAAGAAACAGGACCGGATCACCTCAAGGTCGTAATCTGTCATTGGGTACCGTTGGGCCTCCAACGCTGCCTTCAAATTCTACCAAAATTTC

>chr42:+:6099046-6099169

AGAAACGGCCGGGCGAAAGCCGTGGGGGTTCAGAACAGATGGACTCCTCTACGTCATCGGTTAAGCCCCGCCCATAAACATCCCGGCCGAGATACCAGCTTCCTCTTCACGTGGCCTTGATGAG

>chr42:+:6101478-6103421

ACGTTTTCCGAGAACTTCGCACAACAGTTCCTCATCACCAATGGCACCGACGAGCGAGTGATCGATTTATCGAGCTGAAAGACGACACGAAAAATAATTTTAAAAAATGACGTCATCGGCCTGGAAATGGGCTGGATTCCTTCGAAGTTGACCTGATGTTCTCGGCGATGGATGACCTCGACCCAGGTCGAAGAAACAGGACCGGATCACCTCAAGGTCGTAATCTGTCATTGGGTACCGTTGGGCCTCCAACGCTGCCTTCAAATTCTACCAAAATTTCTCTTCTCTCAAAAAAAAAAAAAAAAAAAAAAAAGTCGAAGGTTTTGAGCCCAACATCAATGGAAGCCTAATCATCGTGTCCTCTTCAAGATCCGTCTTCCAGCATTCTTCTGAATCACACAAGTCCACTTGGAATGTGTCAACCTTAAAATTGAGAAGGTACCTTTGTGCCTCTGTTTAATTTTTAGTCGTCTGGGAGCACACCTCTTGCAATAATTGCGTGATGCTCAATAAAGTGAGGTCGGTTGAAACATTAGCATCACTATCAAGGTCATACTTTCTTCTCGTATCTCAAAACCTTCGTCAAAAGGCTGTGTTTAATGGTTAATCATGACAGTTTCTTTTTCAGTTTCATAAAGAGACTAAATTAGAGAATGAATTCAATCAATGATGTGTTTGCACTTTCTTATAGCAACTGTTAGCTTAAAATCAAAGCAGAGTAAAGATAGGTCATTTGGTCAAGGTCAAGAAACGTAGCTATATTTTTAATTAATTCAGAATTTACTTATGTTCTTTTTTATTTTTTGATCATAACTCATTAAAAATGGTTAAAGAATTTTCAAGCCAAAATACCTTCATTTTCAGGCAATCTATTTAAAGGTTTATCAAAATGTGGGTCTTTTCCCTAAAAGGCTTTTATGCAGACTTAAGATTTATCACAATAATTCATGTAAGTTCATTTCAGTTATTCAAACTTTCTTTAATGCTTGTAATATGGATCAATTCTTTTATATAGGTTGCAATTCCTATACCATTACTCACCACTCATAAGTAACTGTATATTGATTAATTCCCATTACAGGCTTTTATGCCTAAAACTTATCATCATCAAAAGGCTTCTAAGAATGGATCAACTTTTTTTATACAGGCTTCAGTGCCTAAAACATTAATCATCACTCAAAGGCATCTGAGTATTAATCAATTTTCATTATAGGCTATGACATCATCATCACCAAAAGGCTTCAAAGAATGAATCAATTCTTCTATACAGGCTTCAATTTCTTTGCATTATTCATCACCAAAAGGCTTCTAGGTATGAATCAATTCCCATTACAGGCTTTTATGCCTAAAACATTACTTATCACCAAAAGGCTTCAAAGAATGACTCAATTCTTTTATGGAGGCTTCAATGCCTTAACATCATTCATCACCAAAAGGCTTCTAAGTATGGATCAATTCACATTACAGGCTCTTATGCCTAAAACATCATTTATCACCAAAAAGGCTTCAAAGAATGAATCAGTTCTTTTATACAGGCTTCAATGCTTTTACCTCATTATAAATGCACCATTCATTACCAAAAGGCTTCAGAGAAATATTCAGTTTTTTATACAGGCTTCAATGCCTCTGCATTATTCATCACTTAAGGCTTCTAAGTATGGATCAATTCCTATTACAGGCTTTTTGACTAAAACATCATTTGTCACCAAAAGGCTTCAAAGAGAATAAATTCTTTTTATAAATGTTTCAGTGCCTTTACATCATTCATCACCATAAGGCTTCTACGTATGGCTCAATTCCTATTATAGGCTTTAATGACTAAAACATTCATTATCAAAAGGCTTCTAAGAATGGATCATTTTTTTTTTATAGAGCCCTCAGTGTAGGATTTCATGAATGCATATCACTCATCACCAGGATCAGTCTTCAAATTCAGGTTACACA

>chr42:+:6103913-6103947

CCTGCCTCATCAATCATCACCAAATGGCTTCTAAG

>chr42:+:6104389-6104411

AAAGGATCAATCTTCAAAAGCAG

>chr42:+:6105586-6105826

GATTTTATGCCTGCCTCATCAATCATCACCAAATGGCTTCTAAGAAAGGATCAATCTTCAAATGCAGGATTTTATGCCTGCCTCATCAATCATCACCAAATGGCTTCTAAGAAAGGATCAATCTTCAAATGCAGGATTTTATGCCTGCCTCATCAATCATCACCAAATGGCTTCTAAGAAAGGATCAATCTTCAAATGCAGAATTTCAATCCTGCCTCATCAATCATCACCAAATGGCTTC

>chr42:+:16860577-16863114

GGGCCGCTGGGATGGTAGACCTCGCGATCTTTGTCAGGAACGGGAGCGGGGTACTCTCATCCATTGTGAAAATGGTAAAGGGACTCTTGAAAGGTTGTATCCTGGTATTAGAACAATCCATGTCCTCTAAACATCTGAGCCATTCTCTCTGAGCCTGGTCTCGTGAATAGAGGACTGTCTCCTTTGGTACCCTATCATCCCGAACCATGGCTGAAGCTGTAAGCCTTGCGTAGCCTATGAACGGAGGCTGGAGGTCTTCCGGGAAGAACTCGAAGTCCTCTATCCTTCGAGTTCCGAATTCCGGTATGGAAATGAGACCGTCTTGGAAGGGGGCGTATGACGCTACTCTCCATGGATTGTTCATCGAGAACGGAGGTAGCGAGTCATACGGAGGAAGCTGAGTTCCACTCGTATTGGAAAGTGGAGGAGAGGCTAGAGGGGCTTCTCTCAGTCCGGCTATAATGGAGTCTTGGGAAGTAATCCTATCCGACAGACGAGAGATCATTTGTTCCATGCTACTCTTTAGGGACCCAACCAGGTCGCCCACCTGTTGCAACAGGCCAGCATTGGAGTCCAAGGCCGGAGCTGCTGCGGAGGTGGAGGGGTGGCTCTGAATCGGAACCAAGGGTAGCGGAACTGGTGACTCTGCCGGGGTGCGAGATCTCTCTCTGGAGGATTTACTCCTCGAGGACTTAGAGCCGGAGCTAGAAGCTTTAGACCTGTCTGCTCCGGGATTCGCAGCCGGAGACTTACGTGAGGAGGATGACGCCGAAGTCGACTTCTTAGACGACGACGACGTCTTAGTCAAGGATTTCACTGCTTGACCCTTGACCTTAGGTCTAACCGAAGCGTCAGGAGGAGTATATAATTCATCACCTGTAAAGCCTTGGAAGGATGGAGAGGTTGCAGGAGTAGAAGAAACAGTTACACCCAAGGGTGACCCTTGGGTGTCCACCCTACTTACCTCGACCAACAGGTCGTCTACACCTACCATAGGTTCCACATTAATATCTAGAGTCGCGACGTCCGTGGAGATATCCTGGTCTTGATCAGTTAATGAGGCAGCCAGCTGTTGTTGGATGAAGGCGATAGTAGGGGGCCGCCTCTACTGTGTCGACGTATCCTGTCGCCTTGCCTCCGGGGAAGATTAACAGCGCCAACCGCTTCTCCAAGATGTAGGGCATACCCTTGGCGGCGTTCTTCCCAAAACCGCCGACCCAGGCCCGCAGGGTTGCCAGTGCGGTATCCTTCACTGCCGGCGCCTGGAAGAGAGGGCGTAGATTAGATTTTAAGAATCACTTAAAACTTAAACTCAAAATATAACTTAAACTTAGATGCCTTAAGTTAAAGTGGATGATGAAAACTTAAGCACTAAAACAGAAGCGGAGCAGCTACCGGAGATGGGATACTCACCCCTTCTAAAAGCTGGCTCACCAGATCGTTAACATATGGTACACGTCTCATGGTACCAGACCTGGATGTCCCCGTGCAGCGTCGCGCATGGAGCATGGGACCGGCAAACTTCGTGTCCACATGGGTCCTGAAGTGTGGCGGCGCATCCCGGATGCTCACAGTTGGTGGCCTGTAAGTGGGAAGATACATGAGTATCTTAAAGAATAACACTTACAGGCTAAAGGACAGAAGAACTCCGTTGCATGCCGGAGCTCGGAAAAAATTTGGGCATAACCCCCCCCTGCCTCGCCTGAATAGGCTATAATCCCGGAGATATCCGGTAAGACATGTAAGGGAGGGGGGGAAAAGGTTTAAGGTACTTAAGATAAACTTATAGTTAATCTAATAACGCTTAAACCTAAAACTCAAACGAACCGGACCAAGTCCAGTGCGTAGCGGAGTTTAGTAACTCAGCAATGCGGTAAGGTTAGCAGGGACCCACTGTATGTTCCGGTCCACTCTACTGGGTGGACTAACATCTTCCCGCTGGATGCCAGGACTCCGATCAGGAAAGGAGTCCTAGCAAGGTCGGGAAGAGCAGAGGAGGACATACAGGCTCGAGCGAACCGGAGCGACAAGGAAGCAACGGATGGGGGGAAAGGCCAGTACCCCCCACCACATCACCACCGGGCGGACCGAGAAGCCGGAGAGGTCGCGTCCAGTCTGGGTCTGTCCCGTCTCCCTGGCCCCTCCGCCTGGGGGGAGAGAGGGAGGCATGCTCGGGTATGTGGAGCGAGCGTGGGCAGACCGACCCACCCTCCCCCGACTCTATGGAAGAGCGGGAGGAGGGGGGGGGAAGGGGACTGGGCAGGCGTCTGGCTGTCTCGTGATCGCGTAGTGACCACGAGGCGGTAAGACTAAAATAAGACAACTAAGCCTAGGACCAACCAACTGATCAGAGAGATGCTATCGGGAAGCAACTGAACTGAAAAGCGGTAGCATGGAGACCTACTGGGCCAAAACCGACTGATCAGAGAGATGCTATAGGGAAGCAACCGAACTGATGAGCGGTAGCATAGGCTCGGAGCCAGGACCTAGGCTAAGCCAGACGCCTAACTAACCTAGCCACAACAAAATACATGGTAT

>chr42:+:20955151-20956293

GAAAGAAATTAGCATTAAAGAAGGAACATACGATGAATCTTGAAATAACAGAGAACACAAGATTGGAGTGGTGACCAATTGCGTATTTTCTCGGGTCTTTTCTTTCGTTGTGAGTGTATGTAATATATTGTGCGCTTGTAATGTAGACGTGTTGATGCCTATGTATGGTTGAATATATATATATAAATATATATACAGAAATATAAATGTGGTTGCCTTCGGAGATAGACGTTGAAAGTGTGTCTCTCTTGGATGATGGTGAAGAATCGTCCATGTTTCTTTTGTTTTATGGTGTTGCGCGCCCCGAGCCCCCTCTGTTCAAGTCCTCTTGAGACCGAGTTCGTCTTCTTCTTCTTCTTCTTGCTTTCTGGATGGATGATGTCCTCTGACTCTCTCTCGATGAGTGTCCCTCGGCGGCGTTGTTCGAAAGGCTTCCGTCCGACAGCTCCTGCCTGTGTAAAAGTCTTTTCCAAGAGCCAGGGGAGGGGCGCCGCGGAGGGCTGGGGAACCGCCGAGTCCCTCCCGGGCCCTGACGAGAGAGCGCTGTTGTGTCCTTGTGTGATAACGTGCGTCCTTCTTATCGTCAACCGAAATCTTGGGAGGCAGGAATGATGCAACGTTTTCCTCTGTCCGTGGGGAGACGAACGAAGGGTGGGGGCGTCATCGGGCACCACCGCATGGGGCATTAAGGGGGAGGGGACACTTGATGCAGAGGCCTTCCTTGGATGACTTCCGAGGCTCGTCTCTTGTGTATACGCGGAGGGCGGGGAGGGGAGGCGTGGCTTCCGCCCTCGACCTCGCCACCTGCTGCCAACATTAGCAGCAGCAGCAGCTGGTGGTGGTGGCCTCTGCATCCTCACGAGCAGCAGTCAGTAGCAGTAGCAGTGGCAGTAGCAGGAAGGAGCTGAGCGTTCTTCGGGAGAAGAGCAACGCGGGTTCTGCCTGCCGGCCACTGCCTGCCCCGTCGACGGATGGATGGATGTTCTCCGGGGAGCTATTGACGAGTGTTGATTCCAGTAGTAGTAGTAGACCTACTACTATGTCGTTTAAAGCGTTATCCAAAGCCATGATTTGAACGTTGAGTGGTCGTGCAAAAGGCGCTGGCGCCTCTCCACCACGTTTTTCAAAAACTAAAAACTAAAC

>chr42:+:49924920-49926881

GGATGCAGATAGCAGCATGGTCAGCATTGGAAAGAAGGCCACAGCAGTCTTAAACCCAAATTCTCTCTTCATCAATACATTCCTAACTGTCAGAAAGTAAGGCGAATATGAATACGTATAAGCATGTCCAAGAGAAAGAATGAAACAAGGGTCCTAACCACATCTTCCACAGGTGGAAAACCGTTAGCTAAAAAGCCCAGGCACCATCATGAAAATAATGACCAATACTGTGTTACTACCTATATTCAAAATGCTATTTCTGAGGGACTATTCCAGTAACAAACCATGACAGAGAAAGGGAAATAAACTAGTTTCCATAAATATATTTTTCTTAGTGAACACTTTTTAAAAGTAGAAACAACCATACAGCAAAAACTTATTTGGGTACTCATTTTCTTAGTCTTTGAGTACCAGAAAAGCTTATTTCTTTACCATTTTGAAATCAATTCAACTGGTTAAATTATATGGCTTACTACCTTATAATTTACATCAAGAATGTAACTCTCTCAACAGGACTCCCAATGAACCCACCAGCTCATTCTACCAATATGAATTTCTGCCTCAAATAGAGCCCACATTTTTATCTACATGTTTTAACAATCTAAAACAGTTGCCAAAACCTTATGATTTACATTGCTCTTTTCCTCAAAATCAAATTCCTTTCCTATCATCACTTTCAGTACTCATGGAACTAAAATCCTCTCTACCCACAACTTGTGCTAAATATCTCATGGTCCTAATGAATCATTTTCACATTGGAAAGTGAGTCTTCAGTTCCCCTCCTGGCAAACTCCCTCTCAAGCAGCACCCAGGTGCTACTTTGTTTTCTTCTGGGCATCCAGACCTATCACAGGGTTTTTCATTCAATTAAAAGGCCACTTTGCAGAAAAACATTCCAAGCTGGCATAAGACCTGCTTAGACTACAGCAGCAACAAAACATCCCGATATCATTCTAGGAGAAAAAGCGAAAGAAAATTTGTGACATCGGTGTCAAGACCTTGGATGAAAAAGTTTTTCAATTTCACTTATGTATCAGATTGACGAGAGGGACCTCCCACATTTACTCAGAGCAGGATCAAAACAGTAAAAAATGCATTTCTTCAGCGCAATCTAGTTGTCTGTAAAATGTATACTGCTGTATGAAACTCTCAGCAACAGCCCATAAAACTCTCAGCCATGGCTTAGTGGGGGCCTGTGTTGTTGGCACTTATAGCAATGCCAAATGCCTGATCATGGGTAACTTTTATAAAATAAAAATTACAGAGGCTAAAAGGCTGCCATTTGGTTGGAGGGTGGATGATTGACATACCATAGTTTCTAAGATGTGAGGGCGGATGGACACAAAGCCATCTCAGCAGTCTCCTTTACAGAAAACTAAAAGGAAGCAGGGGACCACTACGCATGCCATATCAGTTTAGTCAGATCTTTACTGTCCCCTCATTCAAGTTGGAGTCATCACCCCCACCAAAAACAAGCAATAGCACAACACTCACTCTGAAAGTATATAAATGAGCAAAATGGAACAGATACCATTCAAATCCAACTAAATAGGGTTAGGGCATTTCACACATGATGGTTTCAGTTGAAACTGATCAACCAAACTCCTAACAGCCCTCTTAAACAAGTCTATTGTGGGTGCAAAGGTAGTTGAGTAACCTGTGGAGCAGAAACTCATGTCCAGCAATGCCAAGTTCAAACTTGCATAAACATAAAGATGTTTCAAAAAAGATCTGAAAGCTCACTACAACCTTCTCAATTAAAAGTTGATCTATTTGATCCCACTAGCTTGACATAATTTGACATGTTGTAGAAACATCAATTTAGCTTCATTTATTTGTCACTGACTATGAATCAACACGTCATACCTCAAACTCAGCCAAACAAAACATTCAGGCCCCTAAAATCAAATTGTTTCTAGGTCAACCAGCTACTGAAATATTACAGTACCTAAAGACATTGTA

>chr43:+:38379001-38380428

GTCGTGATACGTTTTACTACAATCAGAAATTCACACTGTATTCCAGATATCTATGAACGAATCCATCTGAGAAATTCCAATTACTTCGGACATATATCATGTTTTTAAGTCTGAACTGTTTTGTTTTGTAGTGTTAACTAATATAATTTGCATTGTATTTTCATATTCTAGAGTAAATTTAGTGAGATTATGTCTTTTCAGTAAAATACAAATGGGAAATTCGATTAACAAAAGCTAATGATAACTGTACTATCTTCACTTTTCTTGCTGATTGTACATTATCAAATACATAGTAAGTCATAGAAAGACTAAAGTTTGATACATGAGATAACTGGTAATTTCATCTCTCACACTGAGAGAACTTGCCCCAAGTGAAATAAGTTTTAGTGCTTTCAAATGTAGGATCAGAAGGACTTTTTAATTCCTGTTATTTGTGAACATAATTAAGGTTGTACATAATTCAATTCCAATTAAAATGAGAGGGTTTTGGATCAGGATGCAGTAGTTGTCCCATTCCCATGTTACTTTTAAAGTTTGGAAGCAATTGTTGAATAGCAGCCAAGGTTTCTTGTGCTCCACAACAGAAGTGGAGTTGTGAATGTAAGTACAGACACCACCCTACTTACAAACGAGTTATGTTCTGGACAGCCGTTTGTATGTTGAATTGCTTGTTGGTTACTGTACTCTTCAATCAACACAATGCTGTACATTTTTTCATCCTAAAACAATACATTGTATATACAGTACTGTATGTACAGAATTGGTGCTGAAAAAATTTGAACTTATGGGCAGCAAAGAGGAGTGCTCAGAACACAACTTCAATTTGTGATCATGTTTGTTTGTATCTCTGAATGTTTGTAAGTTGAATGTTCATAAGTAGGATGGTGTCTACTGTAGCTTTTCTGAATTACATTATTGGAGACCAATGAATTTTGTCATTTTTTGTTGCCCCTGCTTTACATCTTTATTTGGTCTCTGACCCTCTGTAAGTTTACCAGATAAAGGCAGTCCCTGGTTATCGGCGGGGGTTCCGTTATCAGCAGGGTGCTGATAAGAGGAACCAGCCATTAACCAAAACTTGGTGATTTATGGTGCTTATGGCGCCATGTTTCGGTTAATGGTGCCTCTGTTAGGAATGTTTAGGTATGTTATGGCGCCATAAGTCCATCATCAGAACCTCATGGCGCCAATAACCGAAACTTGGTCCATTATGGCGCTAGACAAGTGCCTTAAAACCAGGTTGCCATTAACCAAGTCCGCCAATAACCGGGGACTGACTGTACATGCAACAGAAATTACAGATTCAACCTGAATAGGAAAAGAAACTCCAGATTTTAGTCCCTCAATTATCTGTGGAATATTGCAGGATTAATGATCTGGATCCTATAAGAAGTTGGTAGTGTCATTATTACAGCAATCCTAATAATC

>chr43:+:44560388-44562227

CATATTCTTGACTGGATTTACTGATACGATCACAGAACTATAAACATACAAATCACTTTTATCATACAGGAGTAATATATTAAAAACATATGGCGCACAATCATACTTGCTGGATGATACAATAAAAAAACAGAAACTCACTAGTACCAGTTGCATTTCTTAAAGTGCTTTGTCTTCAAGAGTTTGAGTACCACTAAAGAACTGTACATTATGAAACTTTGACCAAAGTATTCCCCAGTTGTTAATACAAGATTTTCTTATGTTAACTTTTGACTTTTACTTGCACAATTTCTGCCAGACAACTACATATATTAATTACAACAAAAATTCACCTGCTGTCAAAATACACTTAAGGAATCCTTATCACTTTCATGTGTAGGTGGATTTACTTAGTGCAATATTGGCATAATATTAGGCTTACATTATCTAGCCATCTGAAATATTATTATTATTATTCCTCAAAAAAAAATGCAGAGAGATGCAAATACACAGATAGCATTAAAGGCAAGCAGCTTGGGTGTCCTGATAGAACAGAACAATTTAGAGAACACTCAGGAAGCCAATGACGAAAAGTCAGCTTTTAAAGGTATTAATCAACTGCAGTTTTACCTCTAACAGCAGCATTCAAGCCTTTTAAAACATACTTTAAACTCCTAAACAGAGACCACATCAGCATTTAATCTTTTTTTTACATTACACAACATTAAAACCATAAACAAGTGATTTACAGTTATCCACGTGACTGTGTATAGCAACTTCATTCCTGCATAGTGGAAAAAAAATGCAGGATTGTAACACATTAAATGTACTGCAAAAGTCCTGTTTTCTGCTGTGCCATGAACTTGAGTTGCTAGTACAGTGCTGTAACAAACTTTTTCACTTCCAATAAAGCTCACATTCTTTAAGAAGAAAGATCACTTGATTAGTACATTATCATCAACTGACTTTTTAGGGGGGAAGAGTCAAAATTTACCTGGCAAGTATTTGCACAAATTACTTTCTCTATGTACTCTATAGGAACTTCAACATGGAAGGATTTGCCTAACTAATGTACTGTAGTCATCTACAGAAGTCTTTCCAAGGAATGTAGTTTGATTTCTTTTGTACTATCAATGCAATAAAACACAAAATTAGCTAACTCATTTGGTTACCAGACATACTACCAACAGTGCAATAAACTCAAGACACAGCAAAGCAGGACATAAAATGCAATATTCTAAGTAAAAAAATTTTCAGTTGGAAGGAGGCTTTGTAGGCAAAATAAATTCAATTGTCAAATGTTCACCTTCTTTCTCAAAAGAAATTAAGAATTTTATATTTATGAATGGTAATGTAATTAACTAGCCAGTTATAGCTGTGGTAAGGAATTACAGCTCAATCATTCTTTGCCAAAGATGAATATTTTTCCTGAGTGTTTTACTTTGTAACTTCTAATTTAAATGCAATGTAAATTAAGTGAAGCAGTTACTCAACTCTCATAAACTGCCGAGGCTTAACACGAACAAATAGTTAACCTCAGATTCACACATATGCGTTTGTTATCCTTATTCCACACATTAAATGGATGACTTTCCTTTTACATATCAATAGGTTCATCAAACAAATTACATACATAAATTACATCACAAACATTGGTACAAAATCTGTATATGTAACCTCGTCCAAATTCTACATAACACCCTACAAATATTTCTTCAAATTACAGAAGATCATTAGCATAAAGAAGGTTGGCCCACACATTTAAGCGTAATTAATTGCACAGTATATTGTGAAATGGAACCTTTGTTACTGATACATTACACTGGAACCCAAGGAAAACTTTTCTAAATTATTCCTTAAC

>chr44:+:19965046-19967308

AACATTCTGTAAAATTTATTGAATGGCCAAAGTACAAAGTTAGTATTAAAATCCAACAAGAAAATATAAATACAAATCTAATATTTAAAAACCTCTCTAATCCATTTGGCTACATCTTTAGTAACTTTCTAATTGTGCTCAAGGAATGTCTACTCACTAAGTACCACACATACGCTGTTGCAGTATTGTGCCACTCAATCCTCACTGAGAATCTATGTACTACCTATGGATATGTTCATTTTACATCTTGTGACGCACTACCTTACTCAAATTAGGATAATTATATTGTATGGCTCTTTAAGGCAATAAAAAGACTTCTTCACAACACATTTCAAATGTTTTAGCACCTGAGAAACAGTTTCGTCATCTCATGCATCAGACACTTATATAAAACTTCATTGGTCGTCAGATTTCGTCCATTTCTTTCATCACAGCTGAGAATAATTAACCAACAATCATTCATTTTGAAAGTTTCGCAGTCACACCTGTAAAAATACCTATTGCAGCTGCATTAATCACTGATGCATTCAGTTACATGACCTACGTCATATGCTCGTCATAACAAAAATGATCAGCATAACCTGCATGTTTTACTTTTAAAGGGATAAGGGCTCAACATACTTGCACGTTTTAAGATATAATATCCACAATGGAAGCCACAAGAGTTTTGATTATGAATTCCAAGCTACAAATAACTTTCCTTGAAGTCTTTCGAATATGAAATACTTCAATTTTATGTTGGCTTTACGGGCTTAAATATATACTTATTATAGTTGTAAAACAATCTCAATAATTGGCTCTCAAAATAAAGCTGTACAGTGTATGGTCTGTATATATATCACTTTTTCTGAAACGAAACTGCTCCCGATAAAACAACGCCATGATACAGTTACCGTGCACCAAGGAACAGATCTTCTCACTAAGAGGAAGTCTGCCATAATGAAACAAACCACCATCATCTCACTATCACCAAGTACACAGCCATTCACATTATCACACGAAGCTGGTTTTATCATCCTTTAAACATATCACATTTAAACACAAATGATATCCTCATACCGAATGCTCGAAAGATATATATAAACATTTTTACACTAAAATCAAACGTTTTCCTCAACAACTATCAGTTTCGTCAAATTGCTAAAACCAATTGGAGCAGCCTTGATGCATGAAGCTGTGAATAACAGTGAGAAAGTAAATTAATCCCCGGCTATTTACCTAATCAGTTCTGCGGAATTCAGCCTGATATCTCTTAAAGACCGAAATTAGTACTACTACTATTACTAAAATACAGAAACTAGTACTCCTAGCACTAAAACAAAGCTCTGTCAAGTAGAAAACATCGTCCCAAGGGATTGGCTGAAGATCCGCGAGTGACCAAGACAGAAGACCCCTAATATTCATCCAGTACCACAAGCTAAAAACAATGATTTTTGCTGTTTTCACTTACAAAACAGGCTTACTGCAGTCTTTAGTAACTAAAAACCATGCTAACAATTATTGTGGGGGTAAATAGTCCTCGTGTCAACAATATACTCCTCTTAAAATATATTATAAAGCATCGTATAAAATATAACAACTGCTCTCAAAATTCAGGAAAGGGATAAAATGGTTGTGAATAGGATATCGATCAACATTTTGCAAATAAATATAAAACTATATTTGTGTGTTAGGCCTGCACGTTGACAAGGCCTAAAATCACAGTCCCATAGTAACCAATGATAGACCATATGCCCTCCTAGTTGGACGGGCCTTTGATAAAGCTTTGTGAAGGGAACACACACCATCAGAAAAAATGAATGGTAAGAAGCGCACTAGGTTCATTCTGAATCGTTACGTTTGACACAAATTGCTATTGAATATTATGACGATGAAATTAATATTATTTGCGACCTGTATGCCTCCCCAAAACACATGATGACAGAGAAAATATCCTTGAATGTCCTGGCCAATTATTTCTTGATGAATTACCATCAAATGAAATTTAATTACTTTTTACAATAATTTTTACTTTATCAGTTATTTACGAAATTGTGATTATAAGAAAAGAACTAGAAGTACATTAAATACCCTTTGAGCAAGTTCACTTCTATAAAACAGAATTTCCATGAGTTCATGTCCACGATATTCATTCCTTACAGGAACAATGAGAGACTATTCACGATATTCCTCACAGGAACAATGAGAGATTAAACTCACTCTTCCCAAGAAGTCCCAACATACAGTTATTCAATTCTGGACATTTCCTCGTCTGGGTCAGCTC

>chr44:+:36021563-36023841

GACTGACCATCACATTGAAACATCTGCTTATTTTTTTAGCATTAATTTGAACAAGAAAACTGAACAAAGAAAGATGTGTAGAGCATAGGTGGGTGGTTAGCATAACAGTTTTGCTGTTACAATTGTAAGTTTTGTGAAGAGTGCTCTCTATCATTTGCCAAGTGATACTCACACAAGAGTAGGCGACTATCCCATGCTCTCTGAAAGTGCTAACCCAGGTGCGTGTCCCAAGTGCTTCCCAGTTCAGACAGTGCCTTGTATTTGCAGTTCACCACTGCCTACTAATGTTTTATTGGCAGTTTCACTGGAGTAATGACCTAGTATTCAAAGCCATCCATCTTTTGCTGCTGTTTTTGCTAAAGAACCCAATGCTGTTGGGCCTCCTACAGTGATCAGCTTCCCTTGTAGATAACCTCAATTGTATCAAAATCACACCATGTTCCTCCATTACTACTTCCTGCCTCAACACATCCTGTAACCAAGAATGGTAAGGCTCATATGCAGAATCCTTTCATCTTCACTTTCTTGCCTTTTCTTTCTTCCATGGGAAGTAATAATGGAGATCTCAGAAGCCCTCTTGTAAAAAGTTCTCTCAGACGTTCAAGTGAGCACCATCCTTTTTGTGGGAGTTGTTGACAGGTTTCACTCTCCTTTGGGTAGGACGTCTGCCACAGATAGGGTGGGGACACTAGGTTCTCTTAGCATGCATGTGGACCAGAGAGAGATGCTTCTGTTCTTAGTAGCACTTTTGATCTCCCAAGTTTTTCCTAGCGTGAGAGTTCGGACACATCTAACTTTGGCTACTTTGACTCAGGGACTGTTACAAGAGTAATTGTTCCCTGCTCTCAAACCCAGAAGATTCAACTTGCCATTTAGTTGAGTGACTATCCTCTCACCTGAAAAACTTTTGATAATTCTCATAAGAACAAATAAAAGATTTTAAAGTAACTTGTTCTTCTCTTTGGTATTACAAACCAGAACCTTCAATCCCAATTCTATCTCAGCCACCCTGTGTCATCCCTGATGCTGAAGGAAAAAATAGTTGATTATTGCAGGTTAGTATAGGGTTAGCTCTGCTTACATAGGTATCTACCTACATTGAACTACTTTGTTACCAAGTTCCAATGAACATTGCCACTCACACTGAAGAATGCTTCTATACAGAGGCTCTTGTTCATAGACCTAAAAAAATTACAGAAGGAAAATGACTATTGGGGTTTAAGGTGTCTACTCAGATGTGGAGACTCCTTCCACAAATATCAGTTGAGAACGCTGGTCACGAGTAGGTTTAGGAAAGTGCCGAGACACTGACAATGTCTCTTGTCTTCAGTTTCTGTGTAGATCAGATCATTTATGTAGTTAATGTCCTTGAATCATGAAAGGATACCGACTTGATATGTAAACATTTTTGGGGTATCTGTCTCTTCCCTTTAAATTTCTCATTCTGCTGAAAGCTCTTGTTCAATCAGCATTAAGAACTCTGGTCACTATCAAGCAACTTGGCAATGTTCTCTTCACCACCCTCAAACTAGAATCAGTTTCATGATCCTACAAATGCTGTCAATTCTTAGTATGTGGTACCTTATCCCTCTTTACTTTGACAGAACATGCATCACTTTCAAATATGACAAAATACAAAATACAATCTTGCATATGCTAGGCAAGAATTTCTCAAAATTTACAGTATAATAACACTTGAAGAAAGTCATGCAATTACAGTCACTGACTTTGAATGAAGAAAATCTTGCAGTTACACTCACTGACTGAATAATTGCATTGCAGTTATTTCAATAACCTGTGCTTGAAGATATGGTTAATGTCTGTGCACTATAAAATTTACCATATATTTAGTCATCCAAATTTTTCAGAAATATCTTAACATACCAAATACTAAAAGATTTAGTCACAAATATCATATAATGCTGATGAAGTGTTGTTGCACTACCCAAGATTTGATGACCAGCAGTAAAGGTCTAAATTCTCTACCTCTGTCAAAACACCTAATGGCTGAAGAACACAATCCAGTGACCTTATCAAGCAAATATTGTGTATATATATATAAGAAAAAAAAATAGACCAAACAATTGGTAACCCAGGGTTGATTGTACATAAATGTAATTGCTACTGCTGTTATGAAATATTCACACTCCCACCAACTTCCAAAGTTTATCAAAGACCAAATACCAGATGATGTGAATATAAAATCAGCATCGGGGTATATATTTTCACCTAGTCATTCCAAATGTATACAGTAGGTACTTTTAATAAAATTTGCAAAT

>chr45:+:28344064-28347408

AAAAACATTTTATTTTGGCAAGAATAATACATTACAATTAATACCATAATGAAAAGTTATAGGTCAATTATGACAGCTAAACATTATACCTGTATGGTAATCCATTTGTTATTTTAGTGCTCTGCACGTGAAATATATCATTACCCTACACAGGGAAACTTTTAACGTCAACTTATCTATAAAAATAAAATATTTAACATTTCTTTTTAGTTTAGCATGTATTATTAGGCCCAACTTTTCAGATATTTTTTTTATTCCGAAGACATTATTTTGGTTTACACAAAGAGTAGAACATGTTACTGTATATACCTAATTGAATAAATAGCAAAAGCCCTAGTAAGGTAGTACGTAAATTAGAATTTTTATAGGTTCACCAACAGAATCTAGACTTTTTAAACTTTACAAGTATTGATTTATTAATGTAGTGTATAACATTACAGTAGGTATGTATCTCCGAAACATTATCTTAATGAAGAATAAATTGAGAGTTAATATTAGTTTAAAATGCATTTATGCTTCTAGCAAAGAGAAAATGAGCCTAAAACCCTAGGAATTTCCTGAAGTAAAAGGCATTTTCATATTCTTCCAACAATTTAAGTGAAAACTACACCATGTGAATTTTAGTATAAAAATTTACCAACCCTAAATTCTAGAGCATATTTTATACAGCATAAAAGGTATAGAATAAATTTAGATAAAGGCTATCAAGTCAACAATTACATAATATTCTTTTCCTTTTACCTTGCATTTCATTGTTACATCTCCTGAATTGTACAGTAGTTTACTGTACTTTCCTTCTGATGGCTAATCATAAAATTCATATCCATTGTATTAAATTCAAAGACGGTTCAAATTACTATAATCATACTGTACTTTTGATTTACAGCACAGCAGTGTAAAGTTTACAAATAGCTTTATACATCCAAATTCACAAAGTGTCATTTTTATTGCCTCTTTGAAGTAATCATACCCACTTTTGATTGTAGCCTATCCAGTACACTTTGGCTGTTGAAAATCATTAATGATGATATTTATTTTTGTACAAAACCATTTTGATATTTTACAAGTGAAACCGTGAAGCTCTGACATCATAACTCTCATCGGCTTAAATTAAGGCACGCAAGAGTAACTACAAAACTATTATAAACCTATTTTCCTTCCAGAAAACCACAAATTAAAAGCAAAGTACTGTACTACAAAATGATACTTTTATGGGAACATACAGAAGTGAAAACTAACAATATACGTAGTCTGCTACCAGATCTCACAAGTATCTACATAGTCCTTCAAGACCAGATGCAGGTTGATTAAAACTGTCTCTCCTTGTACCCCAGATAATTTGCCATCCAATATGGAAGCAAATTAAGAAAATATTTATGAACACTTTCTGTAAATCTGAGGAAATCCTTGCCTCAGAACCTGAATAAGCAGTAAGAGAATATATAGATAATGTAAAAGAGTCTAATGGCTATGTTATATTGAATAATATCAGACAGACCAGCAGCAGTTAAAAATAGAAAATAAAAACTATATGTATATATGGAAGATTTTCATCATAGAGATCCCTGAAAAAATGAAAAAAAGACAGATATAGGTCTATCTACATTTTAAAAGGATTTTTCAGGCATATTACCTGTTAAAAGTAACAGGTGCCATGTTTCCCATTATACCAACAAGTAGCAAATCTCACACATAACAATTAATACTCCTATGTAGTTTAAGTACTTGTAATGTACCTAATGTATGTGAAGTGCTGATATGAGCCGATAATTACACCAACTTGACATAAAAGCATTGGATATTTACAGTCATATTGTGTTCCTGTGTACAGAAGTTTAAAGTGACTGTATAGTACTTGTACACAACTAGGGTATATTAAAGGTAAAAAATAGTGTTAAAAATTTGACAGAGAATAAATGTGGCTGAATTACATTTCGTAAATAATCACCTCTAGAATTTTCTCGCTTACAATTTATAAATAAATTTACAGTGCCATTGTGAATTGGCTATATCAAACCTTATCTTGGTGCCCTCTGAACAATGCAGGATACTGTACAGTATGTTAAACTAAATCAGTACTATGTAACAAATCAATACTGCTGGCAATTTCATTGCTTCAAGTAAACAAGAGCTCACTTTTCTTAGTCAACAGTTTAACTGAAAATCTTTCAAAAATTGTAATAGTATAAAAGACTTCACATTCTAAATGTTACATTACATTAACCTTTGAATTATTAACCATTAATCTTTCAATTACTAACAACTAATTTGATATGAAATCCTACAATTATTTTCCAATTATAAAATATCTGAGAGAAACATTCAAGTGTTTTGCCATGCATTCAAGCATTTTATACACAAATTTTACAAACCCTATCATTCCAGTAGTTAGAAATGTAGTATTGCTAGCCAGCCATATACATATTTGCAAATACTACCTGTAGTACATCCTTTATCTCCAAAACAGATACTACTAATCTGTAATTGCCAGTATTAAATGCCATTCAAAAACTCTGTCATTAGCAGCAATACTATATTACCTTTATTATAAAATAGACACATCACAGAATCAACATCTATAGCAACCCCTGTAATATCCTATGGGGCAACCTTTGCACAATAAAGCTCTTATGAGGTGAAAACATATAAGCGTTCATAACTTTTAATTTTCTTGCATTGTACTTGGTTAGAATGCTTATCTCAGTATATTAATTAACTTCTTCATTGTGGGCACACAATTTCTTCCTTGAGATTGTATCTCTGTTGTTCTTAGCATTTTGTTACTTTTTTGTCACAAACATGAATCAACTACTTATCACAGTAGTGCGTAGTTTCTGCAAAAATTAACATATTTTGAACAGAATCTAGTGTCTACCATGATATAATCAGTCACAGTCTATGGTTAAGACACCAAAATATAAATGATAAGTAGATTTCTTTCATCCTGCTCTGGTGCCTAATGCTGAAAGGTTTCTACCCATAAAACAAAATAAAAATTATGCAGAAATTTTTTAAATAGCTGCAACTTTTTCCACCAAAAAGAAATGATTGAATTTCACAAAATCCGGGAAAGAAATTTTAGTGAGCATCGATGTCATTTGCAAAGTGTGTTCAGCACTGAGCAAATGCAGAAAACTAAATGAAAATTTATTCTCACTGCCACAAAGACAGATGCTGTAAGACATCCTTACTTAGTTTGCTGTACAATTTTCCTTCCCTGTCACTGACAAAAAATTATGAATAGGCGCATGACTAAACAAAGTAGTTGGGATTTTAATAAAAGAAAAGTAATGTCTAGAGATATTTTAGCTCTCCAGGTGACAGATGGGAAGTCTACTAGCATAT

>chr46:+:4514708-4517587

AATTTTATGCAATTTACTAATCCAATATTACAATTATTTTAAGCCAGCCATTCACTTAACAAATTTTTGTCATAATCATAAAACCATACATTATACGTAACATTTATAGTTACTTACATATAAAAAGTGTCCAATATAACAAACAAAAAGATTTTAGAATATAAAAATTTCTGGGTTCGCTTTAGCTGCAAATTTATAAATGATCTGAACTGGTTCAAATAAAATAATGTCTAAAGAGTATTACTAAGAATGGTCCCTTAATCCAGGTGCAGCCATATCAACAACTTACAATTACCGACATATAGCACTTAGACTACTACATGATATTTGGACCTTGGAGACTACTACATTATATTTGGACCTTTTCTAAGAAGATAAAAATATGCAAATCAACAAATATCCCAAAATGAAACAAAATTTCTTTTCAAAACTTTTATCCTAATAAAGTTTGAATCATATTAATTTTTTTTACAAAAACGTGTTCAATAATTTGAGTAAAATGGGAACATACTGCAAAGTGCTAACATTTTATATCTTCTATATGAACCTAGACAAACATCAGAATATCATACCATGAAATTTTTAACAATGTCCATAAAGTGTTGGGATACATTTTCCTACAAGACTATCCAAATGAACATTTGATTCTAAACAAGTTAAGTTGTAGACCTTATGAAATTTTAAAATAATGTCCATTAAAACAATCAAATAAATGAAAATTGTATGATGTACCCACATACAATGTTGGAATACATTTTCCTACAAGATTAGCAAAATGGACATTGATTCTAAACAAGTTGTAGACCTTAAAATTACTCTCTAAATGGTCATGCTACTTAAGTAGATACCTATATTCAATAAGGGTACAATTAATATAGGCATTAAATCATAGTGTGTATGAATCAATTGCAACCTCCTTTAACAACCTTAATTTCTTTTATTACATTTCAACCTCCTTTAACAACCTTAATTTCTTTATTACATTTCAAGTTAAAATTTATATTACAACTTTGTTCTTATAAAGTGTCGCAGTAACATACAACCAAAAGTCTAATTAATATATGAAAATATTATACAACAAAGGGAGTATTCATTTTTATTGTAACAAGTACCATGAGACAAAATTGCAGACACTTCATAATATTCAATGTCCATGCTTACTTTATTAATTATTGAATTTTCAAATGTAAGCTGCACTAAAGTATAAAAATTTCAAGTTGCCAGTGGTACTGGCAAAAACTTAAAACTAAGTGGCAAAGATTCTTAATGCATGCAATTTCAAGCATATGTAATACATAAAGCCATGTAGAGTATTAAAGATAAAAAGCATACACATAATATATTGTTGCTATCATTAAAAACAGCACATCTAACCTTGAACATCTCATATGAAAACTTAAAGATAACAGACTTCCATTTCAATCTAAATCATGTAAATATTTTCTCTGCTTTATGTTGTCTGGTGTTTACCTTTCAATGACTTACAGAACATAATCGTATACCTTTTCAACAATGAACCATACAGTATGATTGTAAATGCAGAAGCCATACCTAAATACTGCAAATTGTTGCCTTACATTCAAACTAGCAATGTTTATACAAGATAATATATCACATATAATCAACCTACACATTTAGTATCAAAGACATCCATCCAATATTAAATTTGGATCTTTGTTCTGCAATTGAAAAGATGGTTAATCCAACTATTGTTAACCTTCCAATAGGCATGAATATCCCTTTAACCTTTCAATAGTATAAGACAATTATACATGGATGTTCCATATCCAGGTGGAAATAGTTGCCACAGAATTCCAGAGTTTGGGTGTTTTCTTATTTAATTATTACCTTTATTCTTTCATTTAATTATTCCTGTATATAACAAAAAAACTGTCAATACACAAAGTGCTGATAGAATTACAAGGTGAATTGGCTATTTTTCCAGTGGGTGAGGATTATATGTATACATTTTGATTGTTCTAAATGCACTGGCTATACGAAGATACATAAATATGGATTCAAAAGAAGACCCAGGAAAAAGGAGCATGGGTTAATTGGCCAAAATATGCCTGGTTTTCAAGTTTGACTAAACTATATTTTTACTTTTTATCTTTTATGAGAAATAAATGGGCTTCAACACATTTTATGGAGTTTGGAAATTAAATAGTAGTCAGGTATTAGTCCTCAGTGGTCTAAATGTGAAAAACAATGGGTTTACTTTACTTTTTATAGACTGAAAATAAATTTGGTGCTGGATTTAAACAATCATAATAGCTATTTAGAAAATAATTTAACTCTACTTAGCTCTGTTATCTTACATGGTGGTTGATATATTTGGTACTAATTAAGCTCCAAAGCTGATATAAAAACTTATAATCTCTTATACCAGTCTCCAAGCTCTGGTATTTTCACTGTGATGCCCATCAAAAATAAAACAATGAAAAAGGAACTAGGAACAGAATGATGCCTGCATTTGGTGAAAAACTCTTCTAAAGCCAATGCAGTACAACACTTACAGAAAACAGAACTCGCAGTCTGCAGTTTTAAAATAATTCTACTGTTTATGATTTCAGTTTATTGATATCACTGTGGTGATAGAATCTGTATTCCGTTTCCATATTAATATTCTTCGGGTGATGCAACTAAGTAATTATATTATCAATAACACTTCAATGTACTTTTATGCAACACAGTAACCTATATAATGCATGGTATTCCTAACCCTAAAGCTATATGTATCTGTAAACTGGGACTTTGAAAATGGAAGTTTCTTTACATTAATTTCCATTTTCCACAAAGGAGCTGTTATGGGTATACTTACCACATCCAAATAAATCTTTAGATTCCTCAGACTTAACAGGAGTAAATTCTGACTCCTTG

>chr46:+:19230396-19231921

ATGGAAAGAAAACTTTACTGAATTCCTATGTACAGGACTGTACAATATAAACTAGAGATATAAAGTTGATAAACATACCTAACATCCTAATCATTTTCACAATACAATTCATACGTTAAACAAATGATATGGGAACTTACATACGAGACATTAGAGAATAAATTTATCAACATACATAATGACAAAATGTTAGTACTGTAATTGGTGAAGTTCCCTTTCCATATTCCTTAACATATACAACTGTGATAATTCATCCGAAAGTTCATCCATTGAAAGCAAAGCTCTGTGAGAAATGCAACTGCCAGACAAGCACTGAATTCTTGCACACAGTCTTTATAACAAATCCAGCATGCAAGCCCTTCTATAGTCAAATATGGTACTTGTTACGAAACTCATCGCAACACTTAAGAGAGAAACATAGTATATAAAGGAGTGCTACTGTCCCTATCACAGAATGTGTTAGATTAGTTGCATGTAATATAGCCTGAAAGCCAAAAAAGATGTAAAGTTTAAATCTGTTATAACTGTGAAGAGTGGATGAAGTAAATATTTCATGTAGCTTGGATTTGCAAGTGATGCGATGTAAATCAAACAACAATCAGGTTCTGGTGGCAAAGGCTACATTACAGGCAACACAGATTAAGCACTCACAACTCCGCACTAATTCTGAGCTGAATATAAAGACAGAATTCATGAGCTTAAAACACCGGTAAGAATTAAAAAGACAATTACAATAGTACAAAGTTCTGCATTTTGCAGTACAATTAACATTAAAATAGTTTAATTCTTTTTTAATGACATTTTTGTTGATAATCCATTTGAAGCAGTGTTCAAAAAGTAATGCCTAACGCTCACCACATTACATGGAGAAGCAATGAAATGCAAAATTTACTTAAAAACACTATTAACACATTATATCAATGCTCATAAAAACTGTGGGAGTCACATGTTTGAAGTGAGAGATCCTTTAAATTTCTGTCATATATTATTATAATAAAGTCCCAAACACTGACTGATTGACAGCTGGCAGTGATCAGAGGAGCACCAGTCTTGACTCCCATATATAATAAATCTTTTTTTCTAATATTTAGTGCAATGACTGACTTTATCAAATCAGCCAAAATAAGCAACATAAAGCTTCATTAAGAAGTGCTTTCCAGATGTTGCATAGATGAAACGTGAAGTACATAAAAAACTTTGAATGAATAATGATCATGGAAAACAGTCCTCAAGGAAGTAGTTTGTATGGCTAATTTCATTCTAAAGACTGGATAAATCTTTTCTCTTGGCTTTGACTACACAGCTACACACTGTAAAATCAGTGTCCATACATCCCCAGAATTGTTGTCATCATAGTACAGTGTTTTAAAAACTTTCTACACGAGTAAAATATATTTTCATAAACCTATACTTATTCACAAAAAAAATTACCTCTTGGACTAGGTATAAATGATAATGCTAAAAAAAGTCTGACAACCTTAATAGATAAATAAAGAATAAACCCATCCATTCAATTTGTGCCCTGT

>chr46:+:19230396-19232594

ATGGAAAGAAAACTTTACTGAATTCCTATGTACAGGACTGTACAATATAAACTAGAGATATAAAGTTGATAAACATACCTAACATCCTAATCATTTTCACAATACAATTCATACGTTAAACAAATGATATGGGAACTTACATACGAGACATTAGAGAATAAATTTATCAACATACATAATGACAAAATGTTAGTACTGTAATTGGTGAAGTTCCCTTTCCATATTCCTTAACATATACAACTGTGATAATTCATCCGAAAGTTCATCCATTGAAAGCAAAGCTCTGTGAGAAATGCAACTGCCAGACAAGCACTGAATTCTTGCACACAGTCTTTATAACAAATCCAGCATGCAAGCCCTTCTATAGTCAAATATGGTACTTGTTACGAAACTCATCGCAACACTTAAGAGAGAAACATAGTATATAAAGGAGTGCTACTGTCCCTATCACAGAATGTGTTAGATTAGTTGCATGTAATATAGCCTGAAAGCCAAAAAAGATGTAAAGTTTAAATCTGTTATAACTGTGAAGAGTGGATGAAGTAAATATTTCATGTAGCTTGGATTTGCAAGTGATGCGATGTAAATCAAACAACAATCAGGTTCTGGTGGCAAAGGCTACATTACAGGCAACACAGATTAAGCACTCACAACTCCGCACTAATTCTGAGCTGAATATAAAGACAGAATTCATGAGCTTAAAACACCGGTAAGAATTAAAAAGACAATTACAATAGTACAAAGTTCTGCATTTTGCAGTACAATTAACATTAAAATAGTTTAATTCTTTTTTAATGACATTTTTGTTGATAATCCATTTGAAGCAGTGTTCAAAAAGTAATGCCTAACGCTCACCACATTACATGGAGAAGCAATGAAATGCAAAATTTACTTAAAAACACTATTAACACATTATATCAATGCTCATAAAAACTGTGGGAGTCACATGTTTGAAGTGAGAGATCCTTTAAATTTCTGTCATATATTATTATAATAAAGTCCCAAACACTGACTGATTGACAGCTGGCAGTGATCAGAGGAGCACCAGTCTTGACTCCCATATATAATAAATCTTTTTTTCTAATATTTAGTGCAATGACTGACTTTATCAAATCAGCCAAAATAAGCAACATAAAGCTTCATTAAGAAGTGCTTTCCAGATGTTGCATAGATGAAACGTGAAGTACATAAAAAACTTTGAATGAATAATGATCATGGAAAACAGTCCTCAAGGAAGTAGTTTGTATGGCTAATTTCATTCTAAAGACTGGATAAATCTTTTCTCTTGGCTTTGACTACACAGCTACACACTGTAAAATCAGTGTCCATACATCCCCAGAATTGTTGTCATCATAGTACAGTGTTTTAAAAACTTTCTACACGAGTAAAATATATTTTCATAAACCTATACTTATTCACAAAAAAAATTACCTCTTGGACTAGGTATAAATGATAATGCTAAAAAAAGTCTGACAACCTTAATAGATAAATAAAGAATAAACCCATCCATTCAATTTGTGCCCTGTACCTGTATCCTACCCAGAAACTGTTGTACAGCAATTTTGTGAGACAAAGGATATAATAAATTGTACCAAATAAACTACTAAAAGAACAAAACAGGAATACTACAATGACTTGGCAACAAAAGGTAACTGGTACAAAAGCTTTTTTTTTTTTTATTTAGCATGAGTCATAGGGCAGCCCAACATTCATTCACAAAGGTACAGGTATTGTGAGTGACTGGGTAATTTTGAATTAGGGGATTGTTTGGAGATCAACAAAAAAAAATTAATGAATAATTCCGGAATATGACTTGAGGACGGCGCAAAGAAGGAAGGGCATTTAGAGGAGGAAGAGAAAGTGGATCATACAGAGGCCAGTGATACTTCACATCACTGTATCAATGCTGTTTGTCAGCACAGATATCCCAACACAGCTATAGCTTAGGTTGAGTGGTACATAAATTATTTTTGTGATTAAGAAAAAGCAAGCCTAAATAAAAATACTATAAACTGGTCTCTTAAAATAATATTTATAAGCTTCCATATACAAGTTAATGAACTATCACATTTACAGTGGTCATAAGAGTTACAACTGATATGTGGGTGGTAGCCTGGTTGGTTGGGTAGGATAGTACAACATAAGCTTCGTTTTCAAGACAGAAAAGGTCAGTATTCTGCCTCATCATCATCTTCCTCC

>chr46:+:36066994-36067199

ACAGGAAAAGCTGGACCTAAAGATAAACAGTCATAGATAACAGTGCTATGAATTGACTACTGTAATATTTATTTGAAGGGCATTAGTTACTGTCACAGTTTTCAACAGTAGATCTAATTAAGGTGTGCCTTAGTATATGGCACTGAACTTGGCCTTTGTTCCTCCAGTAATACAAACCATTGCCTGGAATTGGTTGTTGAAATTAG

>chr46:+:36068245-36068301

GATCTGACAGACACATATGGAACAAAAATTATCAGGATTTGAAGAAACATGAAATAG

>chr46:+:36070998-36073791

ATGCCACAAGTCAAAAAGTAGTAGGATGGTCATGAACTCTCCAGTTTTCTGCTTTCACCTTTTGGACAAACTATTGATGAATCATGACTTATGCCACCATGAGGGTGAGGACACACAAAAGACTTGGTTGATACCTCTCCATCTTGTTAACGAATTCTCACTACAGATGGAAGTAGTAAGAAAGATGTGGTCCAGTTTAAATGCTCTTCTTACCTCAGGAACCATTCTGTGCAGAGAACTCGACATACACCAGTTAGATCAGTAACGACTATGCCTTCCAGAACATTGATCTTTAGATAGCCTAATCCACACTTTAATTTTTAAAATTAAATTTTTCCTTTATAGCCAAATACTTTTATCTAGAAAGTCCATCACCCAACCTTGTTGTGCTTTTGCTCCATTTCATTCATACTGAAAATTGAGCTGTTTTCCTATTGAGTACTTGCTTGACCAATCATGTGTACCTGCTAAGAGGGTTTTCTACTAGATCTACTTGCTAGCCAAATTACAGTGTCTAGGATTTGAGCAGATGCATAAATAGACTAAGTACAGTAGTAGTGAGTTTATATGGGGAACATCAGTTTTATAAAAAAAAAAATTATTGTCCCTTTTATATCTTTGATATAGGTATTGTCTATAGAAATAGAATCTGTTCACCTGTACTTTTGAAGCAAATAGTCATAAACAGCCTTACTCAGTATGAGATGGACCCAGCTCATCAACTTGATAGACCATGAAAGGAGGAAATTCCCACAGTGCAAACCTTAATTGCATTTCACTGTCTTGAAAGGCAACACAGTCATACTTCTTTGACTGCTTCAAAATTGGTTTATGCTGCTTATGCTTCCCAGGATTAATGTTATCCTGTTTAATTTAATCACTTTCAGGTGTGATATTTGAATTTTTAGTTTCCTTTCAGTTGATGACTCACTACATTGTCATCACATTACTGTTAATACTCTCATTTTAGTTTTCAATCTTTTCATGTGATTTAGGTTTGGTATAAAATTCTTGATTTCAAGTTTTCACCTTGTTTTAAAATTAGCTGTGTCAATTTTTGTTTTTGCATTGGCTCCTTTTGGGTTTTCTTTTCTCAGTAAATATTTTGCTTTTTGATCAGTTTCAGACTCAGCTGTTCATTATTGACATTTTGGTTAGGATTAAGTGATTTTGTATCTTTATTGTGAACTTCCCTTTTTTGTGATAGCTTTGTTTGTGGTATTTTTACTTTGCATGGAACCAGTGGTTATCCAGCAACGGGACCAACGGCTTTACCCGACTTCTGGGAATCATGTCTCGGCTTGCTGGTATGGGCAGGGTTATTAAGTTCTCTCCACTTGAAGTGTAGCACCTGTAGACCCAACAGTGTTAAGTGTGTGTGTGTGTTCCCCCAAAATGTGTTAGTGTGTGTGGACTGGTCCATTGATTAATGGTTCCTATACACTCAGTAGTACATTTAAAAACATTGTACTTCAGTGCTGCCATTAGTGATTCCTTGCTCTCTTCTCATTGCACCGCCATCACAGATGTCAAATTTTGAATCTTTTCTTATGAGGCCGCCTCCTCTGTGGTGCTGCCTGTCCCTGCTGAGAATGAGCATTGCGAGTGTCATCAACCATCTCCAGTCTCGACATGCTCCATCCTCTCACCTTTTCTTACACGGATGCTCGCCTTTCCACACATTCCTTTATTGATGAAAAAACTTCTTTTTGACAGAAAAGGCTTCCACAGCCAGACTCAAGATGAATGTTTTGTCTGTCTTACAATATCAGTTCCTTATTGTAGTCATTGTTAGTGCTCCCTCCTACTCCTGAGTCATACATCGAGACCGCTGTTCCCATGTATTTGGTGACTTCCACTTTGGTTATTCTTACATTGGCAAGTACCTACTTACCAGTCTGGCTCAAATTCTGTCCTCATGCACTTAACTACTCTCTTGTATTCAACTACTTGCGTTGAAAGGCACCCTTGCACAGTATATCTGGTTCCTGGCCATGGAAAGTGCTTTGCAGTTTTCTTGGTAGCAGGGCCAACAAAATCACTGAAGGAATTACTATGGTTCTACCTAGTCCCAATCTCCTGATGTTTTTTAGTCTAAAAAGCTTTGCTGGGGGTTCCTAGTATTTGAATTTTCAACAAAAGGATATGGTTCTAAGAGGAGAGTCACAGGTATGTGAACTCAAAGAACAGCAGGTGGTCCTATGAGAAGTTCTAGTGGCATTTCAGACACCAGCAGATGCTCCAATGATTCATTGCCTCCTTGTGGCAGCTGCAAAAGTGGTTCTAGGACCTCCTACATCTTGTTCTAGTCTCAGGCTTCCTCTGACTTGGGGTGTTTAAAAGCAGCCCCACTCCCACCAGTTTAACAATCATTTGGAGTCACTGCATATTCATACATGTTGATTATTGAGGGTAGTCTGAGAGAGAATAGCTTATCTGGAAGTGAAGTAGCAGTTGCCAATATGTCCCCCTTTCTCCACCATGAGTTAGACTTAGGCAGTGGTAACATCATTTATTATCTGTTTTGTCCTTTTCTCTCTTGCATGGACCACTATTAAACCTTTTGGGTGGGATTTTACCTTTGAGTTGCACTGCTTCATTATGGCCCCTTGATGAAGTTGTCAGATGCTTCCTTCCTGTATTTGACCCCCATGATTAAGTTCCTGATTGCCCTTGCCTCCTCTGAGCATGTTAGTTCAGGGTAAATCATTGGTCTTTTGTGGATCTTGCCTTTGCCATGACTGTGTTTTAGTGAAAGTTCCTTGTTCTTGAGCTCTTTGCTATTACTGCCTTG

>chr46:+:36073875-36073983

TCTGTTCTCTCACCGAGTCCTTAAGATTTTTGGTGTTCCCAACTTTGTGTAGGTTAGCATAATGACTGTCTATGAACACAGCAATGATTCTGTTGTACTACTTTAGTGT

>chr48:+:6457058-6459884

TATAACAGACATGTTTTAATAAATATTATATAGGCTATGAAGAAACATTACATTAATAATACATTTGACAAAACAGCATTAAAAGATCACAATAAAATAGGTTTTCAGAGGTCCTTAAAAACCATTGTTTTGCAATATTGTCAAGAGAAATATATATACTATGCTCTAGTCATCTTTAAAAGTGCAGAAGGTTTAGGAAGGCCCATGCACAGGATATGACAATCTACAGGGTAAAGAGGCACATTAACATAATTGCCATTTTCTGTAACGAGTCATTTTCTTGTGAAAAAGAAAAGCATTGACCATTTTTTATATTCAACACTTAGCTTTACAACTATTTCATGCTATTGCTAAACAGTTTAGTTGACTATACAACTATTTTATGCTACTGTTTAGGCAGTTTAGTTGTAGTTTGGCTATACAACTATTTTATGCTACTGTTAGGCAGTTTAGTTGGCTACAAGACTCATTCATTGGAAGAAAATTATTTAGGAAGTTTTTGGAATGCATACTTTATTTTGCATGATTAGTGTTACCACTGTCACTACAGCTTAAATATTGAAAACAAATAAAGCTGCTCATGTCCAGCCTTTTCCAAGTTGCATTATGTGCAGTTTTCCTTTACCATATTTTTAGGTATATCCCGAATTGATTTCCATCAAGTTCTTGTCTTCATGATTATTAATATAGTATCTACTTTCAGGAGGAAATGACTAAATGAAGATCATATTTTACACGCTGCTGCAACCCCAGTCTTGGACAGCCACTGAGCAAGCTGACAAGCAGGAGGAGAGAATTCCTTTGGCAAAAGAAATTAGTGGGGAAATAGGGTGCTATTGGGCTACAAGGTCATGGACAGGATTAAAGAGCGAGAGTAGCTGGTTAATGGCTCTCAACTCTTGGACAAGCGAGCTGCAGGTCAACTAATGTTCAGAATTGTCTCTTAAGGTGAAGGATCAAATCTGCTTGGGAGTTACAATTGGAACAAGGTCTACAGCTGCTTAGAACTCATCTCACGTCGGAATGAAGCAAACTGGCAGAGTTCTTCCCTTCAACAGTTTTTAGCAATGATAGCCATAGTCCTACCCTTCAACAGCGGTGCAATTGTAGCAAGTCGAAGCAACCCTACCTTGGACATAAATTGTAACAGGTCAACATAGCCAGTCCTTCAGAAGATATTGCAGGAGGTCAACAAGCGAATCAAAGGTTGATGGACCATAAAATGTAGAGTGAAGTCTACAGACAGAGATGAAAGGAGAACTGATTCTTGAGCATATAATGTTAGTCGTTGGTAAGACTGAAGTGAAAACCTGCAGACTGCCTAAGGAGACCATCCAACATGTCTTGAGAACATAAATATGAAAACGTACTTCAATCAGGCATTGCCAATGGCAAATTATTACCAGTCTGGGAGCAAGAGACTACCATCTTTTGCATAATTTTTCTGAAGTTGATCACAAGACATGATCAAAAGAAGAAAATAAAAACAGCATCCTTGAAGTTACTTCACAGGGCAGGCTCTTCTGGTGAAAGATTATGAAGGGAAATCACTTGACAAACTTTATGCTCTACAAGATGGTGTCTCTGATGCTTCCTTATTAGTTCTTGATTAGAAGTATAGCCTACGTTTGCTTCAGTCATGAACTCTCAAAGTTTTGTTTGGCTCTGAAAACACTAGATTGGTGCCTTCCAGATATCAGTCCATTCAAGGATACCATCTCTTGGTCCTTCAGTCTGTCAATATCCTTGGCATGGCTGGGCCTTTTGCAAACTCTAGTTGAGATGACAAGGTTGACTTAACATAACCAACCACTTGTAGGCTGCTGACATTGACTAAAGCAATGGAGCTTTTGAGAACTGAAGATAACTGCTTGGCTTAAAAAAATAACATTGTGGAAGTAGCAAGACAAAAACCGTGAAGTGGCCTGGAAGATACAGGAAAAATTTAGCTGAAGAATTGTACTAGGTAGAAAGTACAAAGACAAAAAATGTGGAGATAGTGTATCCCATTCATCCCCGCAAAGATAACAATGACTAGAGTGCATCATTTCATCTTGGACAATAAGGTATGACATTTATACACAGAAAATATTAAAATTTCAAAATAAGAACATTTTGTGTCAACTGGAGCACATTCATGAATTCTACATAAATTTCAAAAGAAAGAAGTGTAATTTCTTTTCAAAAGTAGACTAGTTAGTTCCACTATCATTTAAGTTTTGAAAGTGATTAAGCAGATGTCAAAACTCCTGTAAAGTTGACTATAAGTAACAAAATTTGCATAGGTATACAAATTTAATTATATTTACTCCAGGAACTGATTAACACAAACAATAAAAACATAAAAATAACTGTGCTTATGATTTCTGCAGTTTTGAGCTTATAACTGAAACAAAATATAATGTAGTCACACAAAAGAAAAAAAAATTAAATTCAACAAGGGTTCAAAAGTATGACTAGATAGGAGACAGGAAATTGAGTATAAAAACTGATAAATATGTTTGGTAAAGTTAAGTTGAATGGAAGTAAAACATTAACATTTAGAAAGGACCCAACACTAACTAACTTGCACTGTAACTTAGCCAATACAGAACAAACTAAAGTATAATTTAACATATAAAGTGACACTTAAAAGCTAGTACCCAATTTGTGCAGACAAGCTATTTGAATTAAAGCAAAGGAAAAATCAACGTATTTAATTCTCTAACTCTACAATAAACGTATCTGATTTAAAACATAACAGGTCATTAGCTATTTGACATAATCTTATATTACCTGAATAGGTAAATGGTAAAAATTAACTGCACCTTATGACTCAAACCATAAT

>chr48:+:6560683-6562740

TAACTGTAACTAGACATGTATTTTATAAAATATCACAAAATTTACATAGTCTTTCATGGCATTTGATAAAGAGTAAAATGAACAGCCTACAAAATATCTCAAACTCAAACATTGCCATATAATAGTAAAGTCATGTAATAAGGAGAAAATAACTCCTGTAGTTATCTTAGCTGACCTACAATTACAGCAATACAGTAATGCATTCAATGAAGGTGTTTTTGCAGGTTCATACATCATAAGTACCTTTAACACCAGGCTTTATTTTAATAGATTACTTTTTAGTTTAATAATCAAAAGAGAAAGCATCTTGCAGCTTATCTCAAGACCTTTTTCATTTGCGGTGCCACTGAAGAGAGCATTTCCAGTGTTTAAAAGAAGTACTATTTTAATGATTAAAAATCAAGGTATACTATAATGTACTGAAATACAGTTTACCATTCTTGCAAAAAAATGACACTACACAATGACACTAAACAAAATAAGTACAGTAAGAATAATGGCATTCTTCTTCTTCCCAAACAATACACCAGGAAATTAAGGCTTGGCAAATGACAAGAATTATGGCAAAGAGGTTTTTCTTTTGTGTGTTACAAATACGTTAGGGCGTGAATATATTTTTGCACGCAATTTTGAGACATCTTGTCTCTTTAGTAATAAATTTTACAGCAAATTTGAAAACACATTTCCCACAACTCCTCTTATTTGAAATTGACAGACGAGCTCCGACAGGTCGACTTTACTTGCAAACAAAAGACCAAAGCTGTAGCACCATTAAAAGTACCAAAAAATATAAGACACAAATCAGCTCTGTGTGACAACAACCGAATCTAAAGCTACAACCAATACTTTAATTTACCAAACCATCAATTCAACCTACAGGTCTGAATCATTTAGGAAATTGTTTCCAAATGAATTTACGTCACTCAATCTGTTGACTGGGAGCATATTCATAATATATTTATCATACGTAGTAATTATGTAACTACTATTCTTGAAACAACCTTCAGCCTTTCCAAATTAATATTCGTACACAATACAAATATATAGACCAATGGTTATATGTTTGACATAAGACAAAACTACAAAGGTGGTATCCTTCTAAATTCAACTTAGGCAAACACTGGCAGAGTTAACTTTGAAGTGTGAACAATTTACAGACAAAGAAACAATTAAATTACAAATTGTGACATAAATAAAAAAAAAGAACCTAGCAATTTAAAACTGGCTTCTGAGCCCTGAAAAATAAAATATGCAAAAGTTTGATTACATGCTGCTGTATCTTGCATAGATGAGTAATCATTTCCCACACAGTGAATACAATAAAAACCTTTGCTACAAATAATATATGATGCGAGACTACAGTTCGTTTATCTCGATAGATGCTTTGTGTACTGTAAATCTGGGTCAACGTACGTCAACCATGCTCTATCCTGGAATGACTACTGACTGAATATGCAACCTTGACTATGGTATGTCACAGCTAACTTTATTCCTCCACACAAATGTGTAAGGTACTTCTCCCTAGGTGTCGAATCTAAATAAAAGGCAACGCAATCCAGCAATATAAAAAAAAAATTTGGAGTAAAATCCCACATGATATAGACTTTTGGTGCCATTTAAAACTATAGTTTCTAATTGGTATAACTAAACATTTCAATCACTGTTGTTTTGCTATGCATTCCTGTACTAATAGAAAGGAATAAGTTTGTTTCTGTCTATTGTTAAATTCTTTCAAATGAACATATCACTTTCTGAGGAATATTGCAAATATAAACTAGTATTGCTTGTTGTCTAATTATTTCGTACTGAACCATATATATACCAAGCTATTCAAACAATTACAAAAATAATCTGGCCCTACGCACTAAACAATGGCCTACATCTACGTACAATCACAAGCTACAACAATTTACTGTTGTACTACAATATGAAAAGAAGGAATTCAAAATTATGGAAAACAGATTATGGAGAATTTTAAATGATGCTTTTTCCTACTACAACAGGGCTAGAATGAGGCATTAATTGTCATAAAATCCATGGTAGTTTTCCACTTAAGCA

>chr48:+:20083734-20086208

TCCTTTGCCATTTTCATTTTATTCTGATAGTACATCTTTCCAGCCTTAGGTGGCCAGAAAATGGCATACAAAAAACCATGGTTTACAATTTTTTTTTTTTTTTTAGTCAAAATTGACTTATGACTGTTGACCATACACGAGAATACATAAAGTATAACTACATATACTTTATGGAAAATGTCTACGATGATTAATGCATAACTACACATACTTATAGGAAAAAGTCTGAGATGATTAACACAGAATAAGCCATGTCATGCTATCCCTAACAGCTTACAACCCTTCAACATGAATCCTCCAACTACACTGATGTTTATATCGCTCGAAAATACACCAAACTTAATAATTATCTATTACTGATTGCTACTTACAGTGATACAAGTTTCTAAATTAACCTTTTCTTCATAAATTAAAAGTGGATTAAAAATTAGTTTCTTATTGTCAAAGCAAATACTTCATTCTCAAATAATAAAAAAAAATGAATTGTCTATCCAACCTTCACACCACGGCCATTGCTAAATAACATACCATTTTCCATTATTAAATTGTCCTATTTTCCATTAAATGAATCCTACGCTGGCCATTCAGCTAAACTTCCTTTCATAACAAAAAAATAAAAAACCAAATCAATAGGTTTTGGTGGGAGGTTCCATGCTAGTTGTGTTGAAATAAAAACTTTTCTGCTTAAAAGCATCCATTAACTATCCGTAACTGCAAGTGACACCCAACTAAAAACAAATGCTCAGGCTCAACACAGGATATTTATCTTTCCGTGTTCTATACTCTACTGCAAATCAAAATAAAACAATTCCCTAATTTACAAAAAAAAAAAAAATTGCACTTTTTGTGGTTAGCATATTTATATTTCATTAGTCAATCTTCTCTCAGCCATATCGCGTGGCAGCCTACTGAATGGTATCAAAATCCTTGCATAAAGTAATAAGTGGACTTACTGTTGATATTCGAACATTTGGTCATTAAATATGGGGCCCAAATAGGTCAGCATGCAGATGTCTCTGTCCAAGGAACACTTCGCACTACATACAGAGCGAGATATGATCCACAGACAATAGGGTACATGCAAGACTTCCCTAACTGATGCTTCTATTTAATGACCAATTATCAAACAGGGTGCATCCTATAATATCAAATTATGATACGATATATTAAACTTAACACCAATCAATGATACTATATATTAAACTTCCACCAATCAATTTTAAAAACTATAAATAACCTACAGCCGTCCACACTAACACTGAATATTTCACGTGACCCTGACACGTAACATCTCACAGCCACATCACCAATGTACACGAGTAAGAAAATTTCAAACTCGGCTGGTTACATTATCACCATTGCACTTCACTGTTGCACAAATCACTTTCATACCCCATTGAGTTCGTAAAAAAAAAAACAAGAAGCCATATTCTTTGTTCATAAAACAAAGGGGGGGGGGGGGAGAGAAGCCACATTCTTTCAATTTACCTCCCTCTTTCCCAACCATCATTGTCGCCCTTTAACTGAACACTGCTAAATCTAGCTACCATTCACTGCCAACCTTTCGTTCAGGGGAGAGTCACCCCCCCCCCCCCCCCCCCCCCCCACAAAGACCGTTCTGGAGAGTGCTCCCCCCCCCTTCCCCGGGGGCCCTTCCCTATCTGAACACTGCTAAAACTAGCTACTAATCACTCCCACTACAATGGCGGATGGGCAATTTGCCAAGCTTTCGTTCGGGGGAAAGGCGCCCCCCCTCCACACAAACCCCTTTCTGGGGAGTCCCACCCCCCACGGGGTCCATGACTGTTCCCCGTTCCCCCTCCCCCCTTTTCCGGGGAGTGGCTCAAGAAGGTCGTACAACTTCCACTTTGTCCAGCCTGATTTCACCTTATCTGGGCTGGTGCAATACGCATAATGCGCAATTACTGGCAATTTAACATACTATGAATGAATGGCTAATTAAAACTACGACCACATGAATTGAAACTACAACCTAAAATATTATGAACAATAACATTCTACTTTCACACACTAACTGTGACCTTCGGCCATTTGCCATCAAGCAATATCCTACAATAATAAATTTATATTAATATTTTTTTAAAGTCCTATGCAAGGTCTAATCCCTGTTATTCACTATAAAAAAAAAAATAAGTTTGGTATACAGTAACGTTACTTAAGACAGTAATACCTAATAGATAAAAACTAAGCAATAAATAATTTGACAAAATCAGCTGATTGCTACAACACTAATAAAAAAATAAAAATAAATTACCACCGGCGTCACAACATGGCTTATTTCTTACCGTCAATCTTCACGAAAACTTTCAAAATAAATAAATTCACTTTAGAAAAAAAAATATACAAAATAATTCAACGTCGGGATACAAAACAATCGTTGAAAGTGGCTGGCTGGATGGAGTTCCGGAGAGTGAGCTGGAGAGATTCTTTCACTTGCACGGTCGCTGCTCGTAT

>chr48:+:20083734-20084159

TCCTTTGCCATTTTCATTTTATTCTGATAGTACATCTTTCCAGCCTTAGGTGGCCAGAAAATGGCATACAAAAAACCATGGTTTACAATTTTTTTTTTTTTTTTAGTCAAAATTGACTTATGACTGTTGACCATACACGAGAATACATAAAGTATAACTACATATACTTTATGGAAAATGTCTACGATGATTAATGCATAACTACACATACTTATAGGAAAAAGTCTGAGATGATTAACACAGAATAAGCCATGTCATGCTATCCCTAACAGCTTACAACCCTTCAACATGAATCCTCCAACTACACTGATGTTTATATCGCTCGAAAATACACCAAACTTAATAATTATCTATTACTGATTGCTACTTACAGTGATACAAGTTTCTAAATTAACCTTTTCTTCATAAATTAAAAGTGGATTAAAA

>chr48:+:20084327-20086208

CTTCCTTTCATAACAAAAAAATAAAAAACCAAATCAATAGGTTTTGGTGGGAGGTTCCATGCTAGTTGTGTTGAAATAAAAACTTTTCTGCTTAAAAGCATCCATTAACTATCCGTAACTGCAAGTGACACCCAACTAAAAACAAATGCTCAGGCTCAACACAGGATATTTATCTTTCCGTGTTCTATACTCTACTGCAAATCAAAATAAAACAATTCCCTAATTTACAAAAAAAAAAAAAATTGCACTTTTTGTGGTTAGCATATTTATATTTCATTAGTCAATCTTCTCTCAGCCATATCGCGTGGCAGCCTACTGAATGGTATCAAAATCCTTGCATAAAGTAATAAGTGGACTTACTGTTGATATTCGAACATTTGGTCATTAAATATGGGGCCCAAATAGGTCAGCATGCAGATGTCTCTGTCCAAGGAACACTTCGCACTACATACAGAGCGAGATATGATCCACAGACAATAGGGTACATGCAAGACTTCCCTAACTGATGCTTCTATTTAATGACCAATTATCAAACAGGGTGCATCCTATAATATCAAATTATGATACGATATATTAAACTTAACACCAATCAATGATACTATATATTAAACTTCCACCAATCAATTTTAAAAACTATAAATAACCTACAGCCGTCCACACTAACACTGAATATTTCACGTGACCCTGACACGTAACATCTCACAGCCACATCACCAATGTACACGAGTAAGAAAATTTCAAACTCGGCTGGTTACATTATCACCATTGCACTTCACTGTTGCACAAATCACTTTCATACCCCATTGAGTTCGTAAAAAAAAAAACAAGAAGCCATATTCTTTGTTCATAAAACAAAGGGGGGGGGGGGGAGAGAAGCCACATTCTTTCAATTTACCTCCCTCTTTCCCAACCATCATTGTCGCCCTTTAACTGAACACTGCTAAATCTAGCTACCATTCACTGCCAACCTTTCGTTCAGGGGAGAGTCACCCCCCCCCCCCCCCCCCCCCCCCACAAAGACCGTTCTGGAGAGTGCTCCCCCCCCCTTCCCCGGGGGCCCTTCCCTATCTGAACACTGCTAAAACTAGCTACTAATCACTCCCACTACAATGGCGGATGGGCAATTTGCCAAGCTTTCGTTCGGGGGAAAGGCGCCCCCCCTCCACACAAACCCCTTTCTGGGGAGTCCCACCCCCCACGGGGTCCATGACTGTTCCCCGTTCCCCCTCCCCCCTTTTCCGGGGAGTGGCTCAAGAAGGTCGTACAACTTCCACTTTGTCCAGCCTGATTTCACCTTATCTGGGCTGGTGCAATACGCATAATGCGCAATTACTGGCAATTTAACATACTATGAATGAATGGCTAATTAAAACTACGACCACATGAATTGAAACTACAACCTAAAATATTATGAACAATAACATTCTACTTTCACACACTAACTGTGACCTTCGGCCATTTGCCATCAAGCAATATCCTACAATAATAAATTTATATTAATATTTTTTTAAAGTCCTATGCAAGGTCTAATCCCTGTTATTCACTATAAAAAAAAAAATAAGTTTGGTATACAGTAACGTTACTTAAGACAGTAATACCTAATAGATAAAAACTAAGCAATAAATAATTTGACAAAATCAGCTGATTGCTACAACACTAATAAAAAAATAAAAATAAATTACCACCGGCGTCACAACATGGCTTATTTCTTACCGTCAATCTTCACGAAAACTTTCAAAATAAATAAATTCACTTTAGAAAAAAAAATATACAAAATAATTCAACGTCGGGATACAAAACAATCGTTGAAAGTGGCTGGCTGGATGGAGTTCCGGAGAGTGAGCTGGAGAGATTCTTTCACTTGCACGGTCGCTGCTCGTAT

>chr48:+:20083839-20085001

GTCAAAATTGACTTATGACTGTTGACCATACACGAGAATACATAAAGTATAACTACATATACTTTATGGAAAATGTCTACGATGATTAATGCATAACTACACATACTTATAGGAAAAAGTCTGAGATGATTAACACAGAATAAGCCATGTCATGCTATCCCTAACAGCTTACAACCCTTCAACATGAATCCTCCAACTACACTGATGTTTATATCGCTCGAAAATACACCAAACTTAATAATTATCTATTACTGATTGCTACTTACAGTGATACAAGTTTCTAAATTAACCTTTTCTTCATAAATTAAAAGTGGATTAAAAATTAGTTTCTTATTGTCAAAGCAAATACTTCATTCTCAAATAATAAAAAAAAATGAATTGTCTATCCAACCTTCACACCACGGCCATTGCTAAATAACATACCATTTTCCATTATTAAATTGTCCTATTTTCCATTAAATGAATCCTACGCTGGCCATTCAGCTAAACTTCCTTTCATAACAAAAAAATAAAAAACCAAATCAATAGGTTTTGGTGGGAGGTTCCATGCTAGTTGTGTTGAAATAAAAACTTTTCTGCTTAAAAGCATCCATTAACTATCCGTAACTGCAAGTGACACCCAACTAAAAACAAATGCTCAGGCTCAACACAGGATATTTATCTTTCCGTGTTCTATACTCTACTGCAAATCAAAATAAAACAATTCCCTAATTTACAAAAAAAAAAAAAATTGCACTTTTTGTGGTTAGCATATTTATATTTCATTAGTCAATCTTCTCTCAGCCATATCGCGTGGCAGCCTACTGAATGGTATCAAAATCCTTGCATAAAGTAATAAGTGGACTTACTGTTGATATTCGAACATTTGGTCATTAAATATGGGGCCCAAATAGGTCAGCATGCAGATGTCTCTGTCCAAGGAACACTTCGCACTACATACAGAGCGAGATATGATCCACAGACAATAGGGTACATGCAAGACTTCCCTAACTGATGCTTCTATTTAATGACCAATTATCAAACAGGGTGCATCCTATAATATCAAATTATGATACGATATATTAAACTTAACACCAATCAATGATACTATATATTAAACTTCCACCAATCAATTTTAAAAACTATAAATAACCTACAGCCGTCCACACTAACACTGAATATTT

>chr48:+:20085073-20085349

GCTGGTTACATTATCACCATTGCACTTCACTGTTGCACAAATCACTTTCATACCCCATTGAGTTCGTAAAAAAAAAAACAAGAAGCCATATTCTTTGTTCATAAAACAAAGGGGGGGGGGGGGAGAGAAGCCACATTCTTTCAATTTACCTCCCTCTTTCCCAACCATCATTGTCGCCCTTTAACTGAACACTGCTAAATCTAGCTACCATTCACTGCCAACCTTTCGTTCAGGGGAGAGTCACCCCCCCCCCCCCCCCCCCCCCCCACAAAGACCG

>chr48:+:20085478-20086208

GCGCCCCCCCTCCACACAAACCCCTTTCTGGGGAGTCCCACCCCCCACGGGGTCCATGACTGTTCCCCGTTCCCCCTCCCCCCTTTTCCGGGGAGTGGCTCAAGAAGGTCGTACAACTTCCACTTTGTCCAGCCTGATTTCACCTTATCTGGGCTGGTGCAATACGCATAATGCGCAATTACTGGCAATTTAACATACTATGAATGAATGGCTAATTAAAACTACGACCACATGAATTGAAACTACAACCTAAAATATTATGAACAATAACATTCTACTTTCACACACTAACTGTGACCTTCGGCCATTTGCCATCAAGCAATATCCTACAATAATAAATTTATATTAATATTTTTTTAAAGTCCTATGCAAGGTCTAATCCCTGTTATTCACTATAAAAAAAAAAATAAGTTTGGTATACAGTAACGTTACTTAAGACAGTAATACCTAATAGATAAAAACTAAGCAATAAATAATTTGACAAAATCAGCTGATTGCTACAACACTAATAAAAAAATAAAAATAAATTACCACCGGCGTCACAACATGGCTTATTTCTTACCGTCAATCTTCACGAAAACTTTCAAAATAAATAAATTCACTTTAGAAAAAAAAATATACAAAATAATTCAACGTCGGGATACAAAACAATCGTTGAAAGTGGCTGGCTGGATGGAGTTCCGGAGAGTGAGCTGGAGAGATTCTTTCACTTGCACGGTCGCTGCTCGTAT

>chr26:+:5496738-5498127

TAAGTGCCCTAAATCCTAACACCAAAGACATAATAATTGGGGTAAAATTGACAAAAATTGACACCATATATGTTGGCAATCTTTTGACACCACTTTACCATCATTAATAGCGTCTGAATAGGGGCAATTTGTACCAGCAACACAAGGGGTACAATTTTGGGTTCTTTTGGCATCTTGGTGCCCAAAAACTTGACACCAAAGGCATAATAATGGTGGTAAGATTGACACCATATATGTTGGCGTTGTAACACCACTTTTACCATCATAGTAGCCTCAAAATAGGGGCAGTTTGAATTTAGTGACCTTAAAATCTTGACACCAAGAGCATCATAATGTTGGCAAAGATGACGCCACACATGTTGGCAATGGAGTGACACCACTTTTACCATCATTAATAGCCTCTTAGAACAATCTAATGAAAATTTTAACCAATAACACAAGGGAACAGTTTTAGCATCTTTTGGCATCTTGGTTCCCTAAAAGAAACTTGGCACCGAGGCCTTAATAATGGTGGCGAAATTGACACCGTATATGTTGGTACTTGTATGACACTATTTTTGCTATCATGAATGATAACCTTTTGATGAATCAAAATAAGGCCAGTTTGAACCAGTGACACAATGGGCAAACTTTTGGTATCTTTTGTCCTGACAACTTGGCACCAAGGGAAAATATTGGTGGTAACAGACACCACATAATGTTGGCACTCATGCGACACCAATTTCACCACTAATAATAGCCACTTTGGGCAATATTCAATAGGGAGGTGCTTTCTGCCATAGGAACTTCTCCTTTCCCAGAAGAATGTCTAAATACTGATTTCATGAAAGTTCATTGCCTCCATCTGTTGCTTGAATAAAATTGGCATTATCTGTCATTCGTATTCAAAAAGCTTTTAATTTCCATTCAAAAATCCATAAACTATCAATTGTATACTCCAAAATGCTAGAATTATTCCTTGTTTTTATGTAATCTTTCAAAGCTGGTAAATTTCCCATAAACAAAAGCTTATAATTTCCATTTCAAAATCTGTATACAATCATTTGTATATACCATAAAGTTAGGAATATTTTTTTATGTAATCTTTCAAAGCTGTTAAACATCTAGTGAATGTATGCCTGCAGAAAAAAATCTACTATATACAATCATTTGTATATTACAGAATATATAAATATTCTTTGTTATCTATATAAATTTCAGTGCTGCAAAACATCTTTTCGTGATTGTATTTAGAGACTGCTTTTCATATATAAAGGTTTGCAATGTACTTTTGGGATGCGTCTGTCCTATGAATTGTGAAAATCAGACTGTTTGTTTATTGTGAATCAAGTCTCTATGCTTGCCGATTGTCCTGTATCTTTTTGTACAAAAATATACACAAACAATTCAT

>chr26:+:18534777-18537386

GTACAAGTGACTTTACTGAAAATGAACAACATACTTTATATGAACTCTACAAAAAGTGCTAATATTACATACTGTACTTGTGACTTATATAATGAGGTTAACGGACATGTTCTATACCACAGAAAACTGACTTCTCAGACTGATGTATAAAGGCAGTTCAAACTCATAGGAAACAGGGTCATGCATAAAAAGCCTTTCCCAAGCTTAATATGAAAGAAACCTTTTTCATGTGAAGATGAAAAATATATCGTACGGAGTAAACCAGAAATACAAGTAAGTTTATACATGACAAAATATTCAATAACAAGGGTCCTATATATATATATGTATATATACTACAGAAATATGGCAGTATACAACTCCGTCTTTATACACTCCGGGCCACACAGACCAAGACCATCTGTTGTTTTATGTACAAGAAAACAAAAACTAAGACAATAATTTCTATACATGACTTTCATTCATAATGGAGTTAAGGGGGGATGTTGACAGTGCAATACCAGATAGGTGACTAGGTCCTAAAATCCTACGTGAGGTGTCATTCAGGAGGGTCATGAGAGGTATTTGGCAACTGCATATATACAAATATTTGTAAATACAATTCACATTTCCCTTTCAGACACGTCTCTGAACCTGAAATGGCAAAATCCAGGTACTGTACACTTCACATAGGAAAAAAAAGGTGAAAACAGAAGCAAGTGGCCAAAGCAACAGGCCTTCCATTTTGCCAAATAATTTCAATGGTTAACTGACTGACTACCCCAATTGGTATTCATCTATAAAATTCATTAATTGAGATGGTGTAACCAATATGCTATTAAGAACAAAAGAATCATGAAATAAAGGCACAAGAAACACAAATAGGATACAGCATTAATAAACATTCATTATCACAGATAAAAAAACCAGGTACGGCAACATATTGGCTACACCTATAAACATTTCATATAGTGCCAAAACAGGTGAATCAAAGTAATATGGGCTGACACAAAGTATACTCCTCATACCAGCACATAGGACTTGCTGCCTATACATATGGTACATATATTTATCATGTATGATTTTAGTTTCAACAACTTTCACAAGAAGTTATGTCATTGTTCAAAACAATATTGAGCTTGTTTGACCATTACAAGCCTTTAATTGCTTTAAATAAAGTAAACTACTCAATTAAAATCTAAACTGCTCACATTGTCCATGCATGTCCAAACAAGTCATGACATCTAAAATCTGAAATCAGCATATCCTCAAAGACAACTACGTTCCATATGACTGGTTCTTACAAGGCAGTTCCACAGGATAAAAGATGTTATTCACTCTAACAAATGAGCCCCTTGACAGATGTGGAAGACTAAGCTTTGTAACTGCATCTATCTCCACTACACCTATGGAAGCAGTTTAATCTCACTCAAAAGATGCTTTCTGCACACATGAATCAATGTCAAACAATTAAGATTCTTTGTCAGACTTCATCCAGTGTTCAATTCAGTCTTCCCTGGCAACCAGTTCTTGACTTAAATCCTGTAAAAATTTCCTCTCCTTATTTATCTTAAGGACTATGTCAGAACTTTCATGCTCAGTACTCATTCTCAACATCTTAATAAAATATACAGTATGGTGAAATGGAAGTTACTGTTAAAGTTGTTATTTATCTTATATAATGTAATAAAACTTTCATTTCCAAGACTTTTATTTATATATATGGTATTAAACTGTTCATGCTTTCCAAGACACGATTTCATGTGAAAACACCCACAATGTTAGAGAAAGAAGAGAAGAAATGTTATGCTTTAGAAATCTTCCAAAATTGCAGAATCTTTTCAAGTTTTCTAATCATAACACCTCGAGTATACATTTTGGTACTAAATCATTTCTTTTAAAATAGCACTATGACACTTATGGACGATCCTGTAATATGCTGCTGAACAAACTGCACAAGTGTACCCCACATATTCCTTCCTATTTTTGCTGTGAAGTCTGTTCAACACAAAATTCCTCCCTACATGATACACAAAATCCCTTCATTTGATTTTCTAAATATTAGTATTTGCATAACATCTTTTAACCCCTTTTTCCTACAATTGTATTTATTGAAATTTCACACTAAAATAACTATTCTACATCATGGAGACTTTGGAAAATGAAGAGAAGAATTCAAGATATTCATCTTCCATCTTGTCAAATACCTAAACTTTCTCAAAGAATTTCTCGAGTTAATTTTCTGTAATAAGATACAAACGTATCTAGTACAGGACTTATACAACTATACTGCATCATTACTTTTTAACTCCAGTGATAATATGAATTTCCAAAAATGAATTTGAAATGTTTATGGTTACTGCATATCCACTGAAATACTGTACACAGTTTTTCGCATTCTCAAACGCTGCAAAACTTACTCAAAGCAGTACAATTTGGCTACTTGATTATAAAGAATGGTGTTGTGAATACTTTAGCTTTTTCTCTCTCTACATTCCCTTTCTAAATAACGCTATGCCTTAAACTGTATTTAAACCTTTAGTCTTAAAAGCTAAGAATTTTATTAAATACTACCAATATACAGTAACACTTTCACCAGTTGTGACTTTCTTTTCATCAAAAAGTTGCCAT

>chr26:+:22454889-22455824
[truncated: 295,127 more chars]
